# Single-molecule, full-length transcript sequencing provides insight into the extreme metabolism of the ruby-throated hummingbird *Archilochus colubris*

**DOI:** 10.1093/gigascience/giy009

**Published:** 2018-02-15

**Authors:** Rachael E Workman, Alexander M Myrka, G William Wong, Elizabeth Tseng, Kenneth C Welch, Winston Timp

**Affiliations:** 1 Department of Biomedical Engineering, Johns Hopkins University, Baltimore, Maryland; 2 Department of Biological Sciences, University of Toronto Scarborough, Toronto, Ontario, Canada and Department of Cell & Systems Biology, University of Toronto, Toronto, Ontario, Canada; 3 Department of Physiology and Center for Metabolism and Obesity Research, Johns Hopkins University School of Medicine, Baltimore, Maryland; 4 Pacific Biosciences, Menlo Park, California

**Keywords:** Pacbio, single molecule sequencing, Iso-seq, transcriptome, liver; metabolism; hummingbirds

## Abstract

**Background:**

Hummingbirds oxidize ingested nectar sugars directly to fuel foraging but cannot sustain this fuel use during fasting periods, such as during the night or during long-distance migratory flights. Instead, fasting hummingbirds switch to oxidizing stored lipids that are derived from ingested sugars. The hummingbird liver plays a key role in moderating energy homeostasis and this remarkable capacity for fuel switching. Additionally, liver is the principle location of *de novo* lipogenesis, which can occur at exceptionally high rates, such as during premigratory fattening. Yet understanding how this tissue and whole organism moderates energy turnover is hampered by a lack of information regarding how relevant enzymes differ in sequence, expression, and regulation.

**Findings:**

We generated a *de novo* transcriptome of the hummingbird liver using PacBio full-length cDNA sequencing (Iso-Seq), yielding 8.6Gb of sequencing data, or 2.6M reads from 4 different size fractions. We analyzed data using the SMRTAnalysis v3.1 Iso-Seq pipeline, then clustered isoforms into gene families to generate *de novo* gene contigs using Cogent. We performed orthology analysis to identify closely related sequences between our transcriptome and other avian and human gene sets. Finally, we closely examined homology of critical lipid metabolism genes between our transcriptome data and avian and human genomes.

**Conclusions:**

We confirmed high levels of sequence divergence within hummingbird lipogenic enzymes, suggesting a high probability of adaptive divergent function in the hepatic lipogenic pathways. Our results leverage cutting-edge technology and a novel bioinformatics pipeline to provide a first direct look at the transcriptome of this incredible organism.

## Introduction

Hummingbirds are the only avian group to engage in sustained hovering flight as a means for accessing floral nectar, their primary caloric energy source. While hovering, small hummingbirds, such as the ruby-throated hummingbird (*Archilochus colubris*), achieve some of the highest mass-specific metabolic rates observed among vertebrates [1,2]. Given their specialized, sugar-rich diet, it is not surprising that hummingbirds are able to fuel this intense form of exercise exclusively by oxidizing carbohydrates [3,4]. This energetic feat is also remarkable in that the source of sugar oxidized by flight muscles during hovering is the same sugar ingested in nectar meals only minutes prior [4,5]. In addition, hummingbirds seem equally adept at relying on either glucose or fructose (the 2 monosaccharides comprising their nectar) [6] as a metabolic fuel for flight [4]. In doing so, they achieve rates of sugar flux through their bodies that are up to 55 × greater than in nonflying mammals [7].

Hummingbird flight is not always a solely carbohydrate-fueled endeavor. Lipids are a more energy-dense form of fuel storage, and fasted hummingbirds are as capable of fueling hovering flight via the oxidation of onboard lipid stores as they are dietary sugars [5]. Lipids are likely the sole or predominant fuel used during overnight periods [8]. Just as flux of sugar through the hummingbird is extremely rapid, the building of lipid stores from dietary sugar is also rapid when needed. For example, ruby-throated hummingbirds can routinely increase their mass by 15% or more between midday and dusk on a given day [9]. The ruby-throated hummingbird (*A. colubris*) completes an arduous annual migratory journey from breeding grounds as far north as Quebec in Canada to wintering grounds in Central America [10]. Hummingbirds are constrained to fueling long distance migratory flights using onboard lipids. In preparing for such flights, some individuals rapidly build fat stores prior to departure or at migratory stopover points, increasing their mass by 25%–40% in as few as 4 days [9,11,12].

The ability to switch so completely and quickly between fuel types means these animals possess exquisite control over rates of substrate metabolism and biosynthesis in the liver, the principal site of lipogenesis in birds [13]. While hummingbird liver does indeed exhibit remarkably high activities of lipogenic and other metabolic enzymes [14], the mechanisms underlying high catalytic rates (high catalytic efficiency and/or high levels of enzyme expression) and control over flux (the role of hierarchical versus metabolic control) remain unclear.

Despite long-standing recognition of, and interest in, their extreme metabolism, the lack of knowledge about gene and protein sequences in hummingbirds has limited more detailed and mechanistic analyses. Amplification of hummingbird genetic sequences for sequencing and/or cloning is hampered by the lack of sequence information from closely related groups, making well-targeted primer design difficult. Only 2 genes have thus far been cloned from any hummingbird: an uncoupling protein (UCP) homolog and insulin [15,16]. These 2 studies offer limited insight into what adaptations in hepatic molecular physiology underlie extreme energy turnover or its regulation. The UCP homolog was cloned from pectoralis (flight muscle), and its functional significance *in vivo* is unclear. The amino acid sequence of hummingbird insulin was found to be largely identical to that from chicken; however, birds are insulin insensitive and lack the insulin-regulated glucose transporter (GLUT) protein GLUT4, making the role of this hormone in the regulation of energy homeostasis in hummingbirds unknown [17–19].

Recently completed sequencing of the Anna's hummingbird (*Calypte anna*) genome provides a powerful new tool in the arsenal of biologists seeking to understand variation in metabolic physiology in hummingbirds and other groups [20]. Despite their extreme catabolic and anabolic capabilities, hummingbirds have the smallest genomes among birds [21] and, in general, have among the smallest vertebrate genomes [22]. Thus, it seems likely that understanding of transcriptional variation, overlaid on top of genetic variation, is crucial to understanding what makes these organisms such elite metabolic performers.

To this end, we produced the liver transcriptome of the ruby-throated hummingbird. Because many of the proteins involved in cellular metabolism are quite large, we collaborated with Pacific Biosciences to generate long-read sequences as these would enhance our ability to identify full coding sequences and multiple encoded isoforms. The primary advantage to the PacBio Iso-seq methodology is the capability for full-length transcript sequencing, rendering complete mRNA sequences without the need for assembly. This has been demonstrated in previous studies to dramatically increase detection of alternative splicing events [23]. Additionally, full-length sequences greatly enhance the likelihood of detecting novel or rare splice variants, which is crucial for fully characterizing the transcriptomes of lesser studied, nonmodel organisms such as the hummingbird.

## Materials and Methods

### Sacrifice and sample preparation

A wild adult male ruby-throated hummingbird (*A. colubris*) was captured at the University of Toronto Scarborough using modified box traps on 23 July 2013 at 8:15 AM. At the time of its capture, the bird was aged as an “after hatch year” bird, meaning it was at least 1 year old. Standard aging techniques make more precise aging of hummingbirds more than 1 year old difficult [24]. The bird was housed in the University of Toronto Scarborough vivarium and fed NEKTON-Nectar-Plus (Nekton, Tarpon Springs, Florida) *ad libitum* and sacrificed after *ad libitum* feeding at 1:22 PM on 16 July 2014 (being 2+ years old). On arrival, it weighed 2.68 g and at the time of sacrifice it weighed 3.11 g. Tissues were sampled immediately after euthanization using RNAse-free tools. Liver tissue was dissected out and homogenized at 4°C in 1 mL cold Tri Reagent using an RNase-free glass tissue homogenizer and RNase-free syringes of increasing needle gauge. We used 100 mg of tissue per 1 mL of Tri Reagent (Sigma-Aldrich, St. Louis, Missouri), and chloroform extraction was performed twice to ensure quality. RNA was precipitated with isopropanol, centrifuged at 12,000×g for 10 minutes, washed with ethanol 2×, vacuum dried at room temperature, and eluted in RNAse-free water [25]. DNAse I (Life Technologies) digestion and spin column cleanup were performed (Ambion Purelink RNA mini kit, Life Tech). RNA concentration and RNA Integrity Number (RIN) were determined with the RNA Bioanalyzer (Agilent). The sample used for Illumina sequencing was harvested using the same methods but from a different animal. The bird was captured as described above on 22 August 2011 at 10:50 AM. At the time of capture, the bird was aged as “hatch year” and it weighed 2.93 g. It was housed and sacrificed as described above on 25 January 2016 at 10:50 AM (being +4 years old). Sampled individuals were captured under the provisions of a Canadian Wildlife Service permit (CA 0258), and all procedures were performed under the auspices of a University of Toronto Animal use protocol (20011649).

### Sequencing library preparation

Pacific Biosciences's Iso-Seq sequencing protocol was followed to generate sequencing libraries [26]. Briefly, Clontech SMARTER cDNA synthesis kit with Oligo-dT primers was used to generate first- and second-strand cDNA from polyA mRNA. After a round of polymerase chain reaction (PCR) amplification, the amplified cDNA was size selected into 4 size fractions (1–2kb, 2–3kb, 3–6kb, and 5–10kb) to prevent preferential small template sequencing, using the BluePippin (0.75% agarose external marker, Sage Sciences). Additional PCR cycles were used post size-selection to generate adequate starting material, then SMRTbell hairpin adapters were ligated onto size-selected templates. Each of the 4 size fractions was sequenced on 10 SMRT Cells, for a total of 40 SMRT Cells. Sequencing was performed by the JHU HiT Center using P6-C4 chemistry on the RSII sequencer. Illumina sequencing libraries were generated using Lexogen mRNA sense v2 Illumina library preparation kit and sequenced on a single rapid-run lane of Hiseq 4000 2 × 100 bp paired end, yielding 153M reads.

## Analysis Methods

### Data processing, isoform clustering sorting, and quality control of liver transcriptome

**Figure 1:**
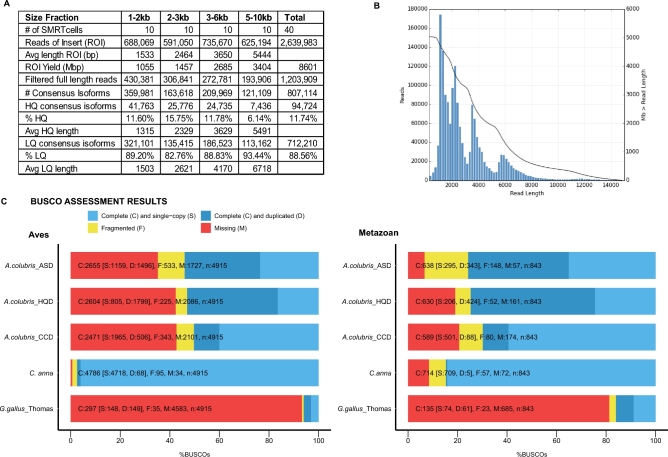
Transcriptome dataset quality control reveals good throughput, read length, and transcriptome completion. Average read lengths and isoform counts for 4 sequenced size fractions given in (**A**) and read length distribution for all sequence data (ASD, all sequence data, high quality (HQ) and low quality (LQ) isoforms) on *x*-axis vs read counts on *y*-axis plotted in (**B**) with black line representing Mb data greater than read length. For example, at 2000 bp, 4000 Mb of sequence data was larger than 2000 bp. (**C**) Benchmarking universal single-copy ortholog transcriptome assessment results displayed for *Archilochus colubris* (ruby-throated hummingbird, ASD, HQ sequence data HQD), Cogent-collapsed data, *Calypte anna* (Anna's hummingbird), *Gallus gallus* Thomas (chicken single-tissue transcriptome [26]) illustrate transcriptome completion relative to predicted single-copy ortholog datasets for both the Class Aves and Kingdom Metazoa. Abbreviations: BUSCO: benchmarking universal single-copy ortholog; HQ: high quality; LQ: low quality.

**Figure 2:**
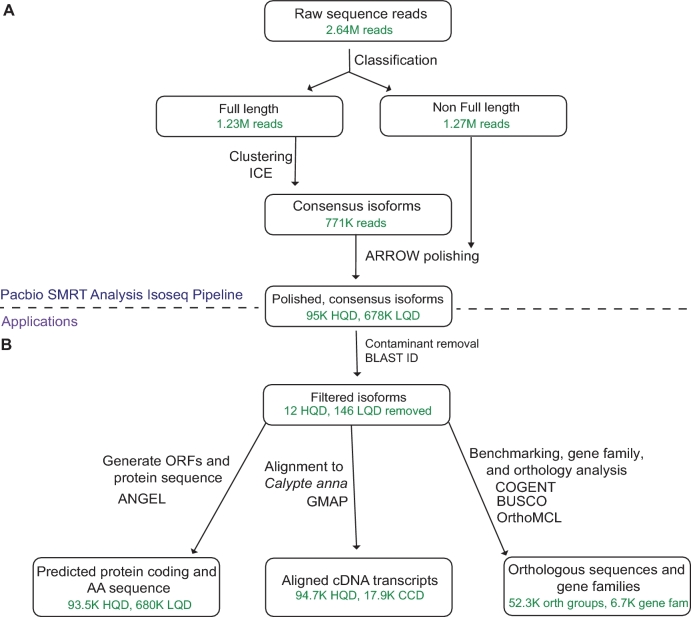
Analysis pipeline details, as well as amount of data present at each step (in green text). (**A**) Raw sequence reads from a Pacbio RSII sequencer (bax.h5, bas.h5) were sorted into full- and non–full-length reads of insert using a classification algorithm that identified full-length reads with forward and reverse primers, as well as a poly-A tail. Iterative clustering for isoforms was performed on full-length reads, and non–full-length reads were recruited to perform ARROW polished on the consensus isoforms. Polishing sorted reads into high- and low-quality bins, and high-quality data (HQD), all sequence data, or both sets of data were carried on to further applications (**B**). Applications include open reading frame and protein sequence generation from HQD and low-quality data consensus isoforms, alignment to *Calypte anna* reference with GMAP of both HQD and Cogent-collapsed data, detection of orthologous sequences (orth groups) using OrthoMCL, and prediction of gene families (gene fam) using Cogent. Numbers of available reads at each analysis step are displayed in green in each bubble. Abbreviations: HQD: high-quality data; LQD: low-quality data; ORF: open reading frame.

We performed initial data processing using SMRTanalysis 3.1 Iso-Seq pipeline using a DNANexus interface. From 40 SMRTcells, we produced 440.75 Gb of raw data, which was classified into 3.4 Gb of non-chimeric circular consensus (CCS) reads. CCS reads comprised 1.23M full-length, 1.27M non–full-length reads; reads were considered full-length if both 5΄ and 3΄ cDNA primers as well as the polyA tail signal were detected. Of the 4 size-selected bins, our average CCS length was 1533, 2464, 3650, and 5444 bp, respectively (Fig. 1B). The Iso-Seq pipeline then performed isoform-level clustering followed by final polishing using Arrow [27] to output high-quality (predicted accuracy ≥99%), full-length, isoform consensus sequences. The Iso-Seq pipeline produced 238Mb of high-quality consensus isoforms (HQD, 94,724 reads), and 2Gb (712,210 reads) of low-quality consensus isoforms (summary statistics Fig. 1A). BLAST searches were then performed to remove putative contaminants, and coding sequence and protein translation were performed, resulting in 93K HQ and 680K LQ protein sequences. A summary of the analyses performed is displayed in Fig. 2A-B, further details and settings can be found in Supplementary Methods, and data can be found in our GigaScience and Zenodo Databases [28,29].

### Assessing transcriptome completion

To estimate the completeness of our liver transcriptome sequencing, we used both subsampling and gene diversity estimation, as well as benchmarking universal single-copy orthologs (BUSCO) (BUSCO, RRID:SCR_015008) [30,31]. BUSCO checks for essential single copy orthologs that should be present in a whole transcriptome dataset for any member of the given lineage. We used both Metazoan and Aves lineages (ortholog sets) to examine transcriptome completion (Fig. 2C and Supplementary Table S1). To ensure that completeness tracked across multiple data processing steps, we analyzed all sequence data (ASD), high-quality data (HQD), and Cogent collapsed data (CCD). As expected, *Gallus gallus* and *C. anna* genome predicted transcriptomes were nearly complete for both Aves and Metazoan BUSCO sets, and our *A. colubris* transcriptome only captured around half of this diversity, likely due to our sample being a single-tissue, collection time point and individual.

Our subsampling approach to estimating transcriptome completeness involved pulling subsets of the circular consensus reads dataset and BLASTing against the predicted *C. anna* gene set. We found that the number of unique genes detected began to saturate when reaching a 90% subset of our data, suggesting that additional sequencing would not substantially contribute to transcriptome completion (Supplementary Fig. S1). Lower expressed genes may not be detected, but that vast majority of annotated liver expressed genes are likely represented in our data.

### Agreement with established Anna's hummingbird genomes reveals general clade conservation

We aligned transcripts to the *C. anna* (Anna's hummingbird) genome using GMAP (GMAP, RRID:SCR_008992) [32]. In order to validate transcript coverage and alignment throughout the multiple processing steps, we aligned using not only high-quality isoforms (HQD) but also the full consensus isoform dataset (ASD) and gene families predicted by Cogent (CCD, methods in Supplementary Methods and below).

*Calypte anna* and *A. colubris* are close relatives within the North American Bee (Mellisugini) clade of hummingbirds [33]; *A. colubris* is a member of the Caribbean Sheartails subclade and *C. anna* is of the Calypte subclade, which diverged from the ancestral Mellisugini around early to mid Pliocene [34]. Given this fairly recent divergence, we expected alignment to perform well. We found an average alignment identity of 94.8%, with 87% transcripts uniquely mapping to the reference. Of the uniquely mapped, 73% covered >90% of the query sequence (alignment length and statistics, Supplementary Figs. S2A, S2B), demonstrating high fidelity of aligned reads to reference. When ASD reads were parsed by number of reads of insert supporting each consensus cluster, it was found that, generally, alignment identity was high regardless of number of supporting reads. A clear increase in mean alignment identity was found when 2 or more supporting reads were collapsed (Supplementary Fig. S3).

When GMAP was performed using only high-quality isoforms (filtered for 2+ full-length supporting reads), alignment percentage was 95.7%, with 93.4% of transcripts mapping uniquely to the reference. The average mapped read length was 2411bp (HQD, 2617 bp ASD), while the average predicted coding sequence (CDS) length for *C. anna* was 1386 bp. This being said, reads mapped with GMAP contain Untranslated Regions (UTRs). When we predict just the CDS sequences for *A. colubris* using ANGEL [35], the mean length was 981 bp. When we BLASTed the unaligned reads to the whole National Center for Biotechnology Information (NCBI) database, they largely mapped back to *C. anna* (53%). This result suggests that our mapping parameters were too stringent to map these reads, error rate prevented alignment, unaligned regions are divergent enough between both hummingbirds to preclude alignment, or a combination of the above.

### Putative gene family prediction and reduction of transcript redundancy reduces data load while maintaining transcript diversity

**Figure 3:**
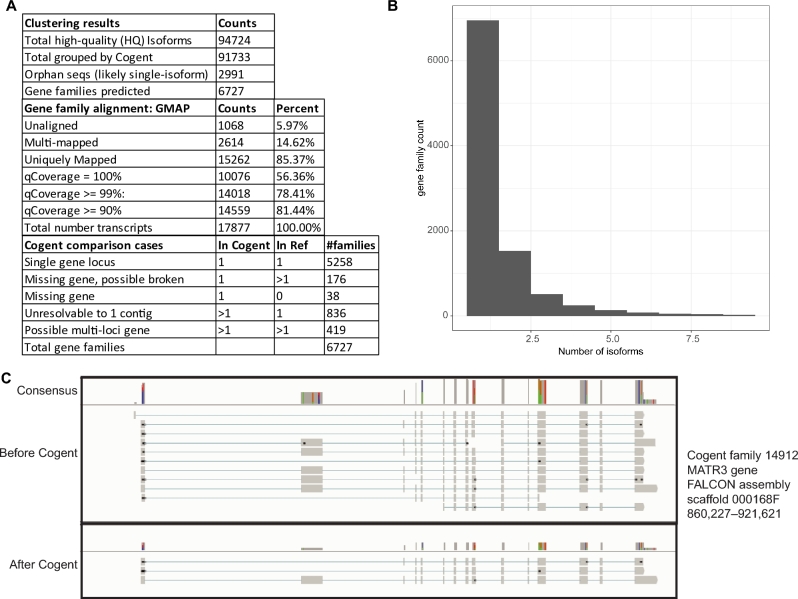
Reducing transcript redundancy and predicting gene families using Cogent software. (**A**) Gene families predicted and classified by relationship to *Calypte anna* genome assembly shown, along with statistics for alignment using GMAP software that show excellent alignment to closely related hummingbird reference species *C. anna*. Cogent comparison cases highlight the relationships between predicted gene families and *C. anna* reference (column captioned “In ref”) and demonstrate the additional information given to an assembly by transcriptome information. Number of isoforms predicted per gene family (unigene) given in (**B**) shows relatively low isoform diversity in this tissue. (**C**) Alignment of the MATR3 gene demonstrates the redundancy-reducing capabilities of the Cogent software, which was reduced from 11 semiredundant reads to 3 unique isoforms using this pipeline.

To assign transcripts to putative gene families, as well as cluster and eliminate redundant transcripts to produce a unique set of gene isoforms, we utilized the newly developed Cogent [36] pipeline. Cogent is specifically designed for transcriptome assembly in the absence of a reference genome, allowing for isoforms of the same gene to be distinctly identified from different gene families, which are defined as having more than 2 (possibly redundant) transcript copies. Of the 94,724 HQ consensus isoforms, 91,733 were grouped into 6725 multitranscript gene families (Fig. 3A). The remaining 2991 sequences were classified as putative single-isoform genes, or “orphans.” Reconstructed contigs were then applied in place of a reference (or *de novo* clustering) to reduce redundant transcripts in the original HQD dataset. With this approach, we were able to reduce our HQ dataset to 14,628 distinct transcript isoforms and 2990 orphan isoforms, for a total of 17,618 isoform sequences (18% of the original). Due to the use of HQD only transcripts (2 full-length reads and estimated accuracy >99%) and constraints of transcript collapse, a number of additional isoforms were likely lost in filtering and collapse, reducing transcript diversity. However, without sufficient supporting data, the trade-off between gene diversity and reliability led us to choose reliability. Future studies should examine whether transcript “rescue” from low-quality datasets is possible with Illumina validation or additional consensus generation strategies.

Cogent collapsed data is further summarized and most abundant transcripts are detailed in Supplementary Table S2. An average of 1.53 isoforms was found per gene family (Fig. 3B), with 2624, or 27.4%, of the gene families having more than 1 isoform, including “orphans.” While other studies have found more isoforms per locus, e.g., 6.56 in *Zea mays* [37], that study multiplexed 6 plant tissues, whereas a lower complexity is to be expected with single tissue analysis. This dataset (CCD) was also mapped onto the *C. anna* genome assembly [38] to demonstrate the effectiveness of this method in reducing transcript redundancy and classifying isoforms (Fig. 3C). Cogent gene families were polished using Illumina short read RNAseq data and the error correction algorithm Pilon [39] (Supplementary Methods) to obtain higher accuracy reads.

### Orthologous gene pair predictions and gene ontology annotation show putative unique hummingbird orthologs

To examine protein sequence similarity and divergence between *A. colubris* and other avian species, we used OrthoMCL (OrthoMCL DB: Ortholog Groups of Protein Sequences, RRID:SCR_007839), which generates reciprocal best hits from comparison species using BLAST all-vs-all, then clustering to group orthologous sequences for each pair of organisms [40]. OrthoMCL protein sequences were predicted using ANGEL [35], and 119,292 high-quality sequences were put into this analysis. We compared our ruby-throated hummingbird, *A. colubris*, to 5 other birds: *C. anna* (Anna's hummingbird), fellow member of the bee clade of hummingbirds; *Chaetura pelagica* (chimney swift), the closest available outgroup species to the hummingbird clade; and other bird species for which relatively well-annotated genomes and/or transcriptomes are available, *G. gallus* (chicken), *Taeniopygia guttata* (zebra finch), and *Melopsittacus undulatus* (budgerigar), as well as *Homo sapiens* (human) and *Alligator mississippiensis* (American alligator). Algorithm parameters and data accession numbers are presented in Supplementary Methods.

**Figure 4:**
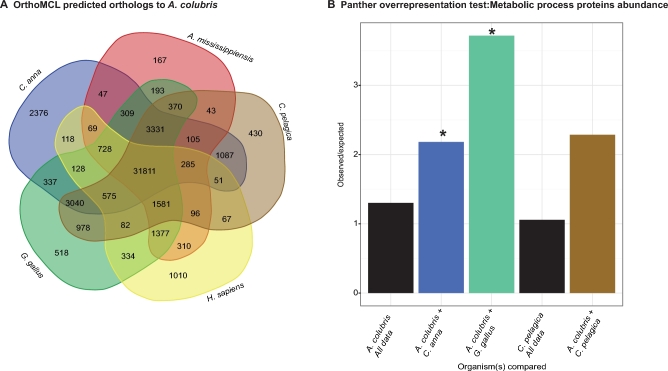
Orthology analysis. The proteomes of 5 birds (Anna's hummingbird: *Calypte anna*, zebra finch: *Tinamus guttatus*, chicken: *Gallus gallus*, swift: *Chaetura pelagica*, and budgeridger: *Melopsitticus undulatus*), 1 mammal (*Homo sapiens*), and 1 reptile (*Alligator mississippiensis*) were compared against *Archilochus colubris* using OrthoMCL to detect homologous sequences. A Venn diagram illustrating sequences with best reciprocal blast hits between the given species and *A. colubris* is shown in (**A**). Bar chart illustrates observed/expected ratios of metabolism enzymes (gene ontology group: metabolic process 0008152) for comparison groups (statistical overrepresentation test) for selected OrthoMCL groups using Panther. Datasets input either include the entire proteome of target species (swift all, anna's all) or distinct set of homologs shared 2 groups (Ex. *A. colubris + C. anna* are homologs shared between these 2 species but not any of the other comparison groups). Asterisks denote significant overrepresentation of metabolic process proteins relative to expected baseline (*P* < 0.05) (**B**).

A matrix of ortholog pairings, with duplicate ortholog hits removed, shows the number of orthologous sequences for each species pair (Supplementary Table S3). Orthologs shared between ruby-throated hummingbird and a subset of the other species analyzed are illustrated in Fig. 4A. Unsurprisingly, the largest amount of orthologs that pair closely to only 1 species, i.e., 1:1 orthologs, were found between Anna's and Ruby-throated hummingbird. Surprisingly, the second-largest set was between chicken and ruby-throated hummingbird, as opposed to its closest outgroup species, *C. pelagica*. This is likely due to the completeness of chicken transcriptome annotation, as chicken is the most well-studied avian species. Of the 596 unpaired *A. colubris* protein sequences, 190 paired most closely with *C. anna* when compared using BlastP and the majority of matches output (559/594) were less than 50 AA, only a fraction of the average sequence length.

In order to more closely examine the identity of orthologs in related hummingbird species, gene ontology (GO) annotation was performed on the set of orthologs that was shared between *C. anna* and *A. colubris* but not by the other birds included in the OrthoMCL analysis. This set of 2376 protein sequences was examined using BlastP and GO analysis performed by Panther [41,42]. Additional datasets used for GO comparison included 1:1 orthologs for *G. gallus* and *A. colubris* (518) and for *A. colubris* and *C. pelagica* (430), as well as whole transcriptome data from *C. pelagica* and the CCD from our transcriptome (Supplementary Table S4, Fig. 4B).

As the initial impetus for our investigation centered on the exceptional metabolism and energetics of hummingbirds, we focused our investigation on orthologs tagged as part of the “metabolic process (GO:0008152)” grouping. Of the 1444 orthologs identified in *A. colubris* as part of this process grouping, 236 (16.3%) were unique to hummingbirds. Within this top-level grouping, the largest number of genes group under “primary metabolic processes (GO:0044238).” Of the 1240 orthologs identified within this grouping, 204 (16.3%) are identified as uniquely shared by our hummingbird species. Six GO biological processes are defined under the “primary metabolic processes.” Of these processes, the process with the highest proportion of identified *A. colubris* orthologs hitting as unique to the 2 hummingbird species is “lipid metabolic processes” (GO:0006629; 33 of 114 orthologs, 28.9%), which is significantly enriched relative to the comparative orthology databases of both chicken and human (statistical overrepresentation test, Panther, [41], *P* values given in Supplementary Table S4). Because we considered it likely that an enrichment in lipid metabolic genes could be a result of our dataset being from liver tissue, we compared enrichment with that of the entire Cogent predicted gene set from the ruby-throated hummingbird transcriptome and found no significant enrichment using the same tests (Supplementary Table S4). Because 1:1 hummingbird orthologs are relatively more abundant in lipid metabolic genes than the sequences that were found to be highly homologous to 1 or more of the other species compared using OrthoMCL, we predict that lipid metabolic genes are more divergent from the other examined species than other classes of enzymes. Though this alone is not direct evidence of greater selection on proteins within that pathway, it is suggestive. If neutral sequence divergence is assumed to be randomly accrued throughout a species’ genome, then greater divergence in enzymes making up “lipid metabolic processes” suggests that closer examination of these proteins for evidence of functional, or even adaptive, divergence is warranted. A phylogenetically informed analysis of ortholog divergence among taxa is necessary to establish a selection signature, which will become possible in the future with the advance of the B10K project [43] and larger numbers of avian species in GO databases.

**Figure 5:**
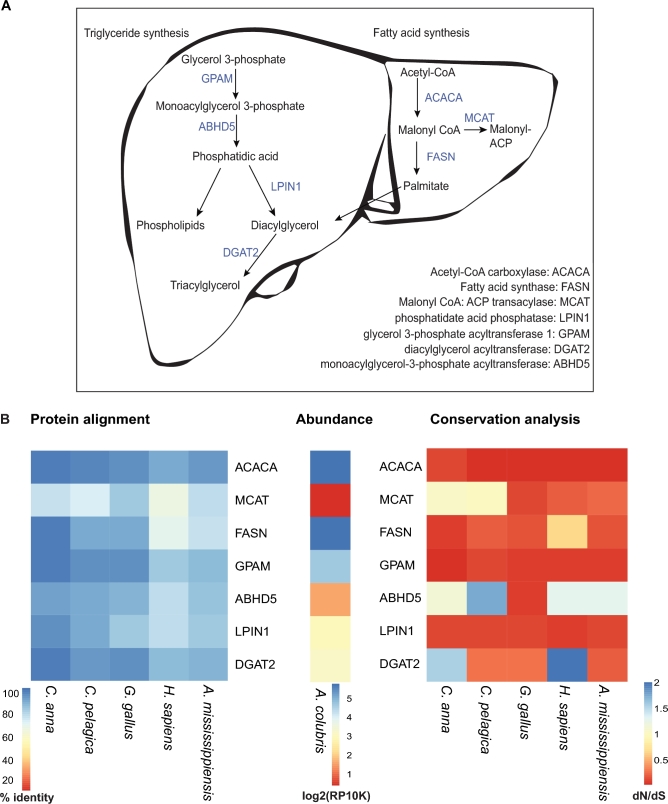
Pathway analysis of key enzymes in hepatic lipogenesis. (**A**) An overview of the relationship between the investigated genes and their roles in triacylglycerol, phospholipid, and fatty acid synthesis. (**B**) Heat maps illustrating percent amino acid identity of these proteins relative to *Archilochus colubris* predicted sequences, abundances (log2 [reads per 10000] transformed) of their transcripts, and dN/dS (ratio of synonymous to nonsynonymous gene mutations). Taken together, these illustrate the complex relationships between target proteins and identity, conservation, and abundance.

Given the apparent sequence divergence among enzymes involved in “lipid metabolic processes” hinted at by orthology and ontology analyses, we elected to more closely examine enzymes that comprise the lipogenic pathway. In liver, fatty acids can be synthesized via the *de novo* lipogenesis pathway using acetyl CoA as substrate. These newly synthesized fatty acids can then be esterified onto the glycerophosphate backbone to generate triglycerides via the glycerol-3-phosphate pathway of lipid synthesis. We predicted that key enzymes involved in these 2 pathways (Fig. 5A) would be divergent in hummingbirds given their extraordinary metabolic demands. Eight enzymes involved in this pathway were examined for *A. colubris, C. anna, Gallus Gallus, C. pelagica, A. mississippiensis*, and *H. sapiens (*accession numbers and details given in Supplementary Table S5). Pairwise protein alignment scores are given in Supplementary Table S6 as well as illustrated in a heat map shown in Fig. 5B, and alignments in Supplementary Data 1. Interestingly, enzymes involved in *de novo* fatty acid synthesis share a higher degree of identity between examined organisms, whereas enzymes involved in triglyceride synthesis tend to be slightly less conserved (Fig. 5A). Figure 5B also shows normalized abundances of the enzymes of interest in our liver transcriptome dataset, revealing a high expression level of the rate-setting enzyme involved in *de novo* lipogenesis (*ACACA*; acetyl CoA carboxylase). In contrast to the cytosolic *ACACA* enzyme that uses acetyl-CoA as substrates for fatty acid synthesis, *MCAT* encodes a mitochondrial enzyme that uses malonyl-CoA as substrates for fatty acid synthesis. Much less is known about the MCAT-dependent pathway of fatty acid synthesis in mitochondria. Interestingly, MCAT has the lowest relative abundance in ruby-throated hummingbird liver. The relative hepatic expression levels of triglyceride synthesis genes (e.g., *LPIN1* and *DGAT2*) are also much lower compared to genes involved in *de novo* lipogenesis (*ACACA* and *FASN*). It is important to note that most metabolic enzymes are tightly regulated. The relative levels of hepatic lipogenesis enzymes may vary greatly depending on the time of day and the physiological states (fast vs fed) of the animals.

In order to further investigate the degree of conservation between key hepatic lipogenesis enzymes in hummingbirds and comparative organisms, we performed conservation analysis and determined the ratio of nonsynonymous to synonymous codon changes (dN/dS) as a metric of positive selection, using pairwise alignments followed by the CodeML module in PAML4 [44]. These ratios are given in Supplementary Table S6 and plotted in a heat map in Fig. 5B. A dN/dS score >1 denotes genomic regions putatively undergoing positive selection. We found, in general, good conservation of these enzymes among species, with the exception of the 3΄ and 5΄ ends of alignments. These often had an extended or retracted coding sequence in the case of hummingbirds and *C. pelagica*, which could be related to post-translational modification or selection on pathway regulation [45]. Surprisingly, terminal sequence length was variable even between *C. anna* and *A. colubris*, which both belong to the closely related Bee hummingbird taxon [33]. Variation in 5΄ and 3΄ length may also be an effect of the different methodologies used to produce these sequences, RNA sequencing for *A. colubris, G. gallus*, and *H. sapiens*, and open reading frame (ORF) prediction from genomic data for the other organisms examined. For example, we note in our analysis that *MCAT* appears more conserved between *A. colubris* and *H. sapiens* than between *A. colubris* and *C. anna*, which could be due not to *A. colubris* actually being more similar to *H. sapiens* but rather to ORF prediction oversights.

The averaged dN/dS values, while useful for comparison, can be misleading when considered over the entire gene, as 3΄ and 5΄ variation can overshadow conserved motifs, and pairwise comparisons (Supplementary Data 1 and 2) are limited in scope. This type of analysis is ideal for very divergent sequences but less informative for pairs of sequences that are highly similar [46]. Despite this, conservation analysis is still valuable and provides insights that connect nucleotide to amino acid information that alignments alone can miss. For example, lysophosphatidic acid acyltransferase (*ABHD5*), which functions primarily in phosphatidic acid biosynthesis, has reasonable protein alignment scores to all comparative organisms but also shows positive selection acting upon this gene relative to *C. anna*, swift, human, and alligator, but not chicken (Fig. 5B). This led us to more closely examine the coding sequence alignment, where we found that the bulk of differences in coding sequence were attributable to exon 1, with alignment largely becoming synchronous (with the exception of *H. sapiens*, which is widely divergent) by exon 2 and continuing through to the end of the transcript. Although the primary AB hydrolase-1 domain is very well conserved between species, these differences in exon 1 could be functionally significant, and honing down to regions of differentiation between comparative species gives us interesting starting points for future investigations, including the cloning and enzyme kinetics studies of *ABHD5*. Additionally, pairwise comparisons provide interesting observations, such as coding strand elongation in the 5΄ region in *A. colubris GPAM* (Supplementary Data 2). This information can be leveraged for future studies examining enzyme structure, function, and evolution.

### Transcriptome resource mining could provide functional genomic insights

Access to the transcriptome informs the investigation of biological processes and enables the formation of new hypotheses. This is exemplified by the serendipitous observation that hummingbird glucose transporter 2 (*GLUT2*) lacks a N-glycosylation site due to an asparagine to aspartic acid amino acid substitution. This missing glycosylation site was also seen in the available Anna's hummingbird genome. All class 1 glucose transporters studied in model vertebrates contain 1 N-glycosylation site located on the large extracellular loop of the protein [47]. In GLUT2 the associated glycan interacts with the glycan-galectin lattice of the cell, stabilizing cell surface expression [48]. Removal of the N-glycan of GLUT2 in rat pancreatic β cells results in the sequestering of cell-surface GLUT2 in lipid rafts, and this sequestered GLUT2 exhibits a reduction in glucose transport activity by approximately 25% [48]. This reduction in transport is thought to occur through interaction of the *GLUT* with lipid raft-bound stomatin [48,49]. In mammals, GLUT2 serves a glucose-sensing role in the pancreatic β cells and is required for the regulation of blood glucose through insulin and glucagon [50]. The lack of N-glycosylation of GLUT2 may contribute to the observed high blood glucose concentration in hummingbirds [51].

Another serendipitous observation was the highly abundant chitinase-like transcript noted from Illumina sequencing results. While humans express chitinase in the gut, but not the liver, chickens express the enzyme in both gut and liver, and other mammals (cows) express the enzyme only in the liver [52]. Suzuki et al. hypothesize that the ancestral state is expression of chitinase in both tissues. While the gut chitinase is used for digestion, expression in liver is believed to contribute to serum chitinase levels and to act as a defense against chitin-containing pathogens [52]. The chitinase-like isoform in our dataset is highly homologous to the chicken liver chitinase-like transcript.

### Reuse potential

In conclusion, our results have leveraged cutting-edge technology to provide a compelling first direct look at the transcriptome of this incredible organism. By using PacBio sequencing, we have been able to generate full-length cDNA transcripts from the hummingbird liver. Transcriptome data generated using the Iso-seq methodology, when coupled to recently developed sophisticated gene synthesis techniques [53], will allow simple generation of relevant isoforms for biochemical experiments. Some of the key metabolic enzymes identified from our work as being unique to either *A. colubris* or at most common to *C. anna* and *A. colubris* can now be quickly cloned and expressed. Follow-up studies will allow for biochemical studies of proteins generated directly from our transcriptome data, measuring their enzymatic properties, e.g., k_cat_ or V_max_, as compared to other avian or mammalian analogues [14,54,55]. Expressed proteins may also be used for structural biology studies, applying either X-ray crystallography or cryoEM to generate structural maps of the proteins, then examine how the structure compares to other analogues.

## Availability of supporting data

Supporting datasets can be found on GigaDB [28]. Filtered fastq files of clustered CCS reads are deposited under SRA accession number SRP099041. Predicted Cogent gene families, coding sequence and annotations, and peptide and untranslated region data are available via the Zenodo data repository [29].

## Availability of source code and requirements

**Project name**: Ruby_isoseq

**Project home page**: https://github.com/reworkman/hummingbird

**Operating system:** Unix

**Programming language:** Bash, Python, R

**Other requirements**: BUSCO, GMAP, Blast+, ANGEL, CLUSTAL, Cogent, and their dependencies

**License:** MIT

## Additional file 

Supplemental Figure S1. Full-length nonchimeric circular consensus (CCS) read dataset, comprising 1,219,580 reads, was randomly subsampled by percentage, and the resultant sequences were blasted against the *C. anna* gene set (16,000 genes). The number of unique gene hits was plotted against percent subsampled and demonstrates that transcript diversity neared saturation with our depth of sequencing.

Supplemental Figure S2. Aligned lengths of all sequence data (ASD) dataset by count demonstrates clear benefit of size selection and the efficacy of alignment at longer read lengths. Alignment statistics for high-quality Arrow-polished data (HQD), Cogent-collapsed data (CCD), and ASD given in B.

Supplemental Figure S3. Alignment percentage increases when number of reads supporting a consensus cluster increases. Alignment mean, median, and cluster count given by number of supporting reads in table.

Supplementary Figure S4. Errors corrected per round of pilon correction plotted on a semilog scale. Using the Illumina RNA-seq data to correct the Cogent gene families dataset. The number of errors corrected per round of pilon drops dramatically on subsequent rounds of pilon.

Supplemental Table S1. Benchmarking universal single-copy ortholog (BUSCO) results. For Metazoan and Aves lineages, gene sets from *Gallus gallus* (single tissue Pacbio Iso-seq), *Calypte anna* whole genome predicted coding sequence, and *Archilochis colubris* high-quality data, all sequence data, and Cogent collapsed data, number of complete, fragmented and missing BUSCOs are given, as well as percentages of total BUSCO groups searched.

Supplemental Table S2. Cogent results. Gene family prediction statistics given in (A), and comparison to *Calypte anna* genome in (C), with number of contigs predicted by transcriptome data compared to those predicted by genomic coding sequences. Of the 6727 gene families, 5472 were reconstructed to a single contig, and 1255 were resolved to 2 or more contigs (B). After gene family prediction, reconstructed contigs were used to collapse redundant reads in the high-quality dataset, bringing read count from 94,724 to 17,618 unique putative isoforms. Most abundant transcripts predicted by Cogent are listed in (D).

Supplemental Table S3. Number of unique ortholog pairs (A), co-orthologs (B), and paralogs (C) for 8 species compared using OrthoMCL. Number of input reads and orthologous groups of each dataset given in (D).

Supplemental Table S4. Enrichment for lipid metabolism gene ontology (GO) terms for avian datasets *Chaetura pelagica* (whole transcriptome and 1:1 orthologs between *C. pelagica* and *A. colubris*), *G. gallus* 1:1 orthologs, *C. anna* 1:1 orthologs, and *A. colubris* whole liver transcriptome. GO terms are procured from genes in both *G. gallus* and *H. sapiens* databases. While both whole transcriptome datasets are not enriched for lipid metabolism genes (*P* = 1), datasets of 1:1 orthologs between *G. gallus, C. anna, C. pelagica* against *A. colubris* all exhibit significant enrichment. Significant *P* values in bold.

Supplemental Table S5. Amino acid and mRNA National Center for Biotechnology Information accession numbers of hepatic lipogenic enzymes for use in conservation and alignment comparisons.

Supplemental Table S6. Conservation score (dN/dS) and protein alignment identity (Clustal %) given for key enzymes in the hepatic lipogenic pathway for *Calypte anna* (Anna's hummingbird), *Chaetura pelagica* (chimney swift), *Gallus gallus* (chicken), *Homo sapiens*, and *Alligator mississippiensis* when compared against ruby-throated hummingbird *Archilochis colubris* sequences. Transcript abundance (in both raw and transformed counts) also given for *A. colubris* show relative abundance of featured enzymes.

Supplemental data 1. Alignments of amino acid sequence for hepatic lipogenic enzymes for compared species [canna = *Calypte anna*, swift = *Chaetura pelagica*, gallus = *Gallus gallus*, alligator = *Alligator mississippiensis*, human = *Homo sapiens*].

Supplemental data 2. Alignments of coding sequence for hepatic lipogenic enzymes for compared species [canna = *Calypte anna*, swift = *Chaetura pelagica*, gallus = *Gallus gallus*, alligator = *Alligator mississippiensis*, human = *Homo sapiens*].

## Author contributions

K.C.W, W.T., and G.W.W. conceived and designed the study. A.M.M. performed specimen sacrifice and extracted nucleic acid for sequencing. R.E.W. performed sequencing library preparation. R.E.W., A.M.M., E.T. and W.T. implemented the data analyses. R.E.W., A.M.M., G.W.W., K.C.W., W.T., interpreted the results and wrote the manuscript.

## Abbreviations

ASD: all sequence data; BUSCO: benchmarking universal single-copy ortholog; CCD: Cogent collapsed data; CCS: chimeric circular consensus; CCS: chimeric circular consensus; CDS: coding sequence; GLUT: glucose transporter; GO: gene ontology; HQD: high-quality data; ORF: open reading frame; PCR: polymerase chain reaction; UCP: uncoupling protein.

## Competing interests

W.T. and R.W. have received travel funds to speak at symposia organized by Pacific Biosciences. Bulk of reagents for IsoSeq were provided by Pacific Biosciences.

## Supplementary Material

GIGA-D-17-00088_Original_Submission.pdf

GIGA-D-17-00088_Revision_1.pdf

GIGA-D-17-00088_Revision_2.pdf

GIGA-D-17-00088_Revision_3.pdf

Response_to_Reviewer_Comments_Original_Submission.pdf

Response_to_Reviewer_Comments_Revision_1.pdf

Response_to_Reviewer_Comments_Revision_2.pdf

Reviewer_1_(Original_Submission)_(Attachment).pdf

Reviewer_1_Report_(Original_Submission) -- Sandeep Chakraborty 02 May 2017 Reviewed

Reviewer_1_Report_(Revision_1) -- Sandeep Chakraborty 03 Nov 2017 Reviewed

Reviewer_2_Report_(Original_Submission) -- Robert Ekblom 23 May 2017 Reviewed

Reviewer_2_Report_(Revision_1) -- Robert Ekblom 20 Oct 2017 Reviewed

Supplemental material
