# Supplementary material for: Single-molecule, full-length transcript sequencing provides insight into the extreme metabolism of the ruby-throated hummingbird Archilochus colubris
Source: Gigascience. 2018 Feb 15;7(3):giy009. doi: 10.1093/gigascience/giy009 (PMC5869288; doi:10.1093/gigascience/giy009)
Supplement: GIGA-D-17-00088_Revision_1.pdf [file giy009_giga-d-17-00088_revision_1.pdf]

# GigaScience

## Single molecule, full-length transcript sequencing provides insight into the extreme metabolism of ruby-throated hummingbird Archilochus colubris

--Manuscript Draft--

| | |
|---|---|
| **Manuscript Number:** | GIGA-D-17-00088R1 |
| **Full Title:** | Single molecule, full-length transcript sequencing provides insight into the extreme metabolism of ruby-throated hummingbird Archilochus colubris |
| **Article Type:** | Data Note |
| **Funding Information:** | Human Frontier Science Program (#RGP0062/2016) — Dr. Kenneth C. Welch<br>Natural Sciences and Engineering Research Council of Canada (CA) (#386466) — Dr. Kenneth C. Welch |
| **Abstract:** | Hummingbirds can support their high metabolic rates exclusively by oxidizing ingested sugars, which is unsurprising given their sugar-rich nectar diet and use of energetically expensive hovering flight. However, they cannot rely on dietary sugars as a fuel during fasting periods, such as during the night, at first light, or when undertaking long-distance migratory flights, and must instead rely exclusively on onboard lipids. This metabolic flexibility is remarkable both in that the birds can switch between exclusive use of each fuel type within minutes and in that de novo lipogenesis from dietary sugar precursors is the principle way in which fat stores are built, sometimes at exceptionally high rates, such as during the few days prior to a migratory flight. The hummingbird hepatopancreas is the principle location of de novo lipogenesis and likely plays a key role in fuel selection, fuel switching, and glucose homeostasis. Yet understanding how this tissue, and the whole organism, achieves and moderates high rates of energy turnover is hampered by a fundamental lack of information regarding how genes coding for relevant enzymes differ in their sequence, expression, and regulation in these unique animals. To address this knowledge gap, we generated a de novo transcriptome of the hummingbird liver using PacBio full-length cDNA sequencing (Iso-Seq), yielding a total of 8.6Gb of sequencing data, or 2.6M reads from 4 different size fractions. We analyzed data using the SMRTAnalysis v3.1 Iso-Seq pipeline, including classification of reads and clustering of isoforms (ICE) followed by error-correction (Arrow). With COGENT, we clustered different isoforms into gene families to generate de novo gene contigs. We performed orthology analysis to identify closely related sequences between our transcriptome and other avian and human gene sets. We also aligned our transcriptome against the Calypte anna genome where possible. Finally, we closely examined homology of critical lipid metabolic genes between our transcriptome data and avian and human genomes. We confirmed high levels of sequence divergence within hummingbird lipogenic enzymes, suggesting a high probability of adaptive divergent function in the hepatic lipogenic pathways. Our results have leveraged cutting-edge technology and a novel bioinformatics pipeline to provide a compelling first direct look at the transcriptome of this incredible organism. |
| **Corresponding Author:** | Winston Timp<br>Johns Hopkins University<br>Baltimore, Maryland UNITED STATES |
| **Corresponding Author Secondary Information:** | |
| **Corresponding Author's Institution:** | Johns Hopkins University |
| **Corresponding Author's Secondary Institution:** | |
| **First Author:** | Rachael E. Workman |
| **First Author Secondary Information:** | |
| **Order of Authors:** | Rachael E. Workman<br>Alexander M. Myrka |

Powered by Editorial Manager® and ProduXion Manager® from Aries Systems Corporation

Elizabeth Tseng

G. William Wong

Kenneth C. Welch

Winston Timp

**Order of Authors Secondary Information:**

**Response to Reviewers:**

Below please find our revisions to the manuscript entitled “Single molecule, full-length transcript sequencing provides insight into the extreme metabolism of ruby-throated hummingbird Archilochus colubris”.

We thank the editors and reviewers for their thoughtful comments on the initial submission. We have addressed all of the reviewers’ concerns and are submitting what we believe to be an improved version of our manuscript. The revised manuscript includes careful reanalysis as well as a new Illumina RNA-seq data set for quantification and correction of errors in the PacBio data.

Reviewer reports:

<<<<<

Reviewer #1: Workman et al. have presented a manuscript on a very relevant topic - third generation sequencing on an fascinating bird, the ruby-throated hummingbird. While I wholeheartedly agree that long read sequencing will address several assembly, and downstream, problems - resulting in a better understanding of several genetic aspects of any organism, there are several inaccuracies in the current manuscript that need to be addressed before publication.

1. There are several transcripts (about 155) from viruses - SRR5237173.540969 is one such example. It encodes a 823 long ORF, which has Identities = 702/824 (85%) with a FBS protein (NP 955606.1) from Fujinami sarcoma virus (FSV). The relation of FSV to avian genomes has been long known (http://www.pnas.org/content/77/4/2018). This should modify, however minimally, the statement 'resulted in 119,292 HQD and 1,061,147 ASD peptide sequences'. Similar techniques should be used for eliminating bacterial and fungal transcripts. Methods for quickly detecting metagenomic transcripts have been elucidated in http://biorxiv.org/content/early/2016/10/04/079186. Also, ORF-based annotation help in filtering bacterial transcripts from PacBio reads (http://biorxiv.org/content/early/2017/01/17/100974).

=====

Thank you for performing this check. We have performed BLAST searches to filter out these contaminating reads (details in supplemental methods). We have removed contaminating sequences from the HQD and LQD consensus isoforms set, then re-run ANGEL to produce the corresponding 93,469 HQD and 679,956 ASD peptide sequences. The resulting lower counts than previous runs were a result of both contaminant removal and changing minimal acceptable protein length settings (details in supplemental methods). Filtered data has been reuploaded to Zenodo [DOI:10.5281/zenodo.781592].

<<<<<

2. Figure 5A, and the associated analysis ('poor pairwise protein alignment between A. colubris and all examined species, such as with DGAT2, is suggestive of misannotation or splice variation in our transcriptome, cases with variable alignment identities provide interesting targets for further investigation') is incorrect. The DGAT2 from C. anna (XP 008493408.1) is 358 aa long, and is 357 aa identical to the ORF encoded by SRR5237173.336808. The color coding in Fig5A suggests about 60% identity, based on other transcripts (SRR5237173.185657, SRR5237173.22637, etc - there are 200 homologous transcripts). So, there seems to be two genes for DGAT2, mapping to two different scaffolds in the C. anna genome - an interesting observation is that one gene has very low expression (a single transcript), while the other has several.

Powered by Editorial Manager® and ProduXion Manager® from Aries Systems Corporation

=====

Yes, it appears the initial alignment was based on a sequence that is “DGAT2-like” and not DGAT2, and that they do in fact map to different scaffolds in the genome. When substituting the SRR5237173.336808 sequence for MSA, we obtain much higher percent identities as expected. The heatmap plot has been updated correspondingly.

<<<<<

3. The PAML numbers, and their evolutionary connotations are not properly explained. Finding the number of genes (ACACA seems to have only one), and possibly quantifying them (roughly, ACACA has about 400 homologous transcripts: some complete - some fragmented) would provide interesting insights in the pathway.

=====

We have added language in the main text to further explain PAML analysis and the implications of it [“In order to...metric of positive selection”]. We have also added a heatmap of relative abundances of transcripts [Figure 5], which has indeed provided interesting insights that we touch on briefly in the discussion [“Relative abundances of enzymes of interest...of the animals”].

<<<<<

Reviewer #2: This manuscript describes the sequencing of full length cDNA (RNA-Seq) from a hummingbird using PacBio technology. In general I find this a well written manuscript describing an important avian genomic resource. It should, however, be noted that the format of this manuscript is not following the journal guide lines for a "Data Note". I have a few questions and suggestions for improvement as outlined below.

Throughout I'm very confused by the mixed use of "hepatopancreas" and "liver" to describe the tissue being sampled and sequenced. My understanding is that the term "hepatopancreas" is mainly used for invertebrates and fish. I thus suggest changing to "liver" throughout.

=====

We agree and have updated to “liver” throughout the manuscript.

<<<<<

The main methodological novelty with this work is the use of PacBio data only, for a transcriptome characterisation in a non-model organism. While I applaud this initiative, and especially the detailed description of the downstream bioinformatics pipeline, it also raises some questions regarding data quality. The standard way of using PacBio data for a species without a reference genome (or transcriptome) is to complement the long reads with a substantial amount of short read sequences (for example Illumina) in order to correct the high level of sequencing errors in the PacBio reads. It is unclear (and not well described in the current manuscript) how the lack of such error correcting affect the quality of the resulting transcriptome sequence. This is especially problematic for inference of variable sites (SNPs and InDels) and the molecular evolution type analyses presented at the end of the results section here. The dN/dS analyses in particular are especially sensitive to sequencing and alignment errors that may be abundant in this dataset. I suggest to investigate occurrence of sequencing errors more formally and to omit any molecular evolution analyses until the transcriptome sequence variation has been validated using complementary sequencing.

=====

Our pipeline includes several error correction methods to address the inherent high error rate associated with single molecule sequencing.  Specifically, the circular consensus reads generated by Pacbio are the result of consensus generation/error

Powered by Editorial Manager® and ProduXion Manager® from Aries Systems Corporation

| | |
|---|---|
| | correction, and the following Arrow polishing step of our data analysis pipeline is purported to produce reads accurate to 99.999% with 50-fold coverage. However, due to the sporadic coverage nature of RNAseq data, not all of our reads will be this accurate. While we are confident the accuracy of our data as sufficient for tasks such as homology identification and redundancy reduction, we agree that Illumina validation should be employed before making claims that involve single nucleotide resolution.<br><br>So, to determine the level of remaining error, and in response to the reviewer's criticism, we performed Illumina sequencing on liver mRNA, and used pilon to determine and correct errors which remain at the end of our pipeline. We found a relatively small number of errors were corrected, and repeated our dN/dS analysis with the corrected dataset and updated Figure 5 to reflect this. The dN/dS values did not change substantially, with the exception of the chicken value which was significantly reduced. The manuscript and methods sections have also been updated accordingly.<br><br><<<<<<br><br>It is repeatedly stated that this is "the first high-coverage transcriptome of any single avian tissue". This is a pretty bold statement, given the large amount of transcriptome studies of several model bird species (such as chicken, zebra finch, flycatchers, crows and others). It is also completely un-necessary in this context. This study is interesting as it is, without any need to try to exaggerate the novelty with this kind of dubious statements.<br><br>=====<br><br>This has been changed, thank you for the suggestion.<br><br><<<<<<br><br>With transcriptome sequencing (RNA-Seq) it is possible to get information on relative transcription rates for the identified expressed genes (through read depth quantification). I'm puzzled why there are no such inferences reported anywhere in this manuscript.<br><br>=====<br><br>We compared overrepresentation of genes in specific GO pathways using Panther; additionally, we have included abundance estimates for our hepatic lipogenesis pathway analysis to address this concern. Also, we have included information regarding the most abundant transcripts in our liver dataset in supplemental table 2D and reference briefly in the main text [Data is further summarized...Supplemental table 2].<br><br><<<<<<br><br>The results section is full of rather detailed methods descriptions. I would have opted to keep these in the methods section only.<br><br>=====<br><br>In revising our MS to a data note format, we have pooled methods and related results into Data Description, and moved detailed methodology to a Supplemental Methods section.<br><br><<<<<<br><br>In the fifth section of the results, it would have been useful to have some information of the divergence time between the Anna's Hummingbird and the focal species.<br>=====<br><br>We have added in this information along with the alignment section of data description.<br><br><<<<< |

Powered by Editorial Manager® and ProduXion Manager® from Aries Systems Corporation

| | |
|---|---|
| | Last section of the fourth results page: "a higher degree of divergence within this class of enzymes than would be predicted statistically". Please explain what statistical test was used here and report the test statistic, sample size and p-value.<br><br>=====<br><br>In order to test for overrepresentation of lipid metabolic process enzymes, we used the statistical overrepresentation test employed by Panther and described in Box 3 of their paper (http://www.nature.com/nprot/journal/v8/n8/box/nprot.2013.092_BX3.html). These results (sample size, p value) are reported in Supplemental table 4, and a more detailed explanation of this was included.<br><br><<<<<<br><br>First section of the Methods: How many bird samples were sequenced? In the first sentence it only says "ruby-throated hummingbirds" (plural without any specific numbers). Later it says that tissue was collected from one bird. Please be more specific here. Also please provide more specific information about the one bird individual sequenced (age, sex, time and place of sampling etc.). A lot of effort have been made on this one individual - it is important to include as much meta data as possible for this.<br><br>=====<br><br>We sequenced a single sample from one bird. Standard hummingbird aging techniques only allow us to resolve the age of wild-captured hummingbirds as either a “hatch year” individual (i.e. one born within the last 12 months) or an “after hatch year” individual (at least one year old, though maybe more). Given the bird was sacrificed approximately one year after capture, and was found to be “after hatch year” when captured, we can only confidently conclude that it was 2+ years old at the time of sacrifice. We have amended the text to explicitly state this age range.<br><br><<<<<<br><br>Data Accession: It would be very useful to also have analyses scripts and pipelines placed in a public repository for future reference.<br><br>=====<br><br>It has been added to our availability and requirements section.<br><br><<<<<<br><br>Legends to figure 1 and 2 are in the wrong order.<br><br>Please check format of reference list.<br><br>=====<br><br>Reference list has been updated to Nature format and links have been incorporated into citations.<br><br><<<<<<br><br>Figure 1. Details here need to be much clearer explained in the figure caption. For example please provide detail about abbreviations used, and axis lables. For B I think "5000Mb of sequencing data was larger than 2000bp" should read "4000Mb of sequencing data was larger than 2000bp". Or am I reading the figure wrong?<br>Figure 2. Very clear and useful description of the work flow and analysis pipeline. Maybe you could add details about the amount of data in- and outputted at each stage of the analyses?<br>Figure 3. Again the caption is lacking in clarity and detail. The reader should not need to be familiar with the specific software and output terminology in order to understand |

*Powered by Editorial Manager® and ProduXion Manager® from Aries Systems Corporation*

| | |
|---|---|
| | what is done. The figure with caption should also be understandable without having to read the main text.<br>Figure 4. Not sure how important this information is (maybe better placed in a supplement). Also it is unclear what kind of statistical analyses that is being presented in 4B. Please elaborate on what was done here. What does the stars represent?<br>Figure 5. Again caption is unclear. What does the right heat map in 5B represent?<br><br>=====<br><br>Figure headings, captions and legends have been updated for clarity. Data into and out of each stage of analysis added to Figure 2. |
| **Additional Information:** | |
| **Question** | **Response** |
| Are you submitting this manuscript to a special series or article collection? | No |
| **Experimental design and statistics**<br><br>Full details of the experimental design and statistical methods used should be given in the Methods section, as detailed in our Minimum Standards Reporting Checklist. Information essential to interpreting the data presented should be made available in the figure legends.<br><br>Have you included all the information requested in your manuscript? | Yes |
| **Resources**<br><br>A description of all resources used, including antibodies, cell lines, animals and software tools, with enough information to allow them to be uniquely identified, should be included in the Methods section. Authors are strongly encouraged to cite Research Resource Identifiers (RRIDs) for antibodies, model organisms and tools, where possible.<br><br>Have you included the information requested as detailed in our Minimum Standards Reporting Checklist? | Yes |
| **Availability of data and materials**<br><br>All datasets and code on which the conclusions of the paper rely must be either included in your submission or deposited in publicly available repositories (where available and ethically appropriate), referencing such data using a unique identifier in the references and in | Yes |

Powered by Editorial Manager® and ProduXion Manager® from Aries Systems Corporation

| | |
|---|---|
| the “Availability of Data and Materials” section of your manuscript.<br><br>Have you have met the above requirement as detailed in our Minimum Standards Reporting Checklist? | |

Powered by Editorial Manager® and ProduXion Manager® from Aries Systems Corporation

Manuscript

Click here to download Manuscript 171002_MS_isoseq_hummingbird.pdf

# Single molecule, full-length transcript sequencing provides insight into the extreme metabolism of ruby-throated hummingbird *Archilochus colubris*

Rachael E. Workman[1*], Alexander M. Myrka[2*], Elizabeth Tseng[4], G. William Wong[3], Kenneth C. Welch Jr.[2+], and Winston Timp[1+]

[1] Department of Biomedical Engineering, Johns Hopkins University, Baltimore, MD, USA
[2] Department of Biological Sciences, University of Toronto Scarborough, Toronto, Ontario, Canada and Department of Cell & Systems Biology, University of Toronto, Toronto, Ontario, Canada
[3] Department of Physiology and Center for Metabolism and Obesity Research, Johns Hopkins University School of Medicine, Baltimore, MD, USA
[4] Pacific Biosciences, Menlo Park, California, USA
* Co-first author
+ Co-Corresponding author

## Abstract

### Background

Hummingbirds oxidize ingested nectar sugars directly to fuel foraging but cannot sustain this fuel use during fasting periods, such as during the night or during long-distance migratory flights. Instead, fasting hummingbirds switch to oxidizing stored lipids, derived from ingested sugars. The hummingbird liver plays a key role in moderating energy homeostasis and this remarkable capacity for fuel switching. Additionally, liver is the principle location of *de novo* lipogenesis, which can occur at exceptionally high rates, such as during premigratory fattening. Yet understanding how this tissue and whole organism moderates energy turnover is hampered by a lack of information regarding how relevant enzymes differ in sequence, expression, and regulation.

### Findings

We generated a *de novo* transcriptome of the hummingbird liver using PacBio full-length cDNA sequencing (Iso-Seq), yielding a total of 8.6Gb of sequencing data, or 2.6M reads from 4 different size fractions. We analyzed data using the SMRTAnalysis v3.1 Iso-Seq pipeline, then clustered isoforms into gene families to generate *de novo* gene contigs using Cogent. We performed orthology analysis to identify closely related sequences between our transcriptome and other avian and human gene sets. Finally, we closely examined homology of critical lipid metabolic genes between our transcriptome data and avian and human genomes.

### Conclusions

We confirmed high levels of sequence divergence within hummingbird lipogenic enzymes, suggesting a high probability of adaptive divergent function in the hepatic lipogenic pathways. Our results leverage cutting-edge technology and a novel bioinformatics pipeline to provide a first direct look at the transcriptome of this incredible organism.

### Keywords

Pacbio; single molecule sequencing; Iso-seq; transcriptome; liver; metabolism; hummingbirds

## Data Description

*Background*

Hummingbirds are the only avian group to engage in sustained hovering flight as a means for accessing floral nectar, their primary caloric energy source. While hovering, small hummingbirds, such as the ruby-throated hummingbird (*Archilochus colubris*), achieve some of the highest mass-specific metabolic rates observed among vertebrates [1,2]. Given their specialized, sugar-rich diet, it is not that surprising that hummingbirds are able to fuel this

intense form of exercise exclusively by oxidizing carbohydrates [3,4]. This energetic feat is also remarkable in that the source of sugar oxidized by flight muscles during hovering is the same sugar ingested in nectar meals only minutes prior [4,5]. In addition, hummingbirds seem equally adept at relying on either glucose or fructose (the two monosaccharides comprising their nectar [6] as a metabolic fuel for flight [4]. In doing so, they achieve rates of sugar flux through their bodies that are up to 55× greater than non-flying mammals [7].

Hummingbird flight is not always a solely carbohydrate-fueled endeavor. Lipids are a more energy dense form of fuel storage, and fasted hummingbirds are as capable of fueling hovering flight via the oxidation of onboard lipid stores as they are dietary sugars [5]. Lipids are likely the sole or predominant fuel used during overnight periods [8]. Just as flux of sugar through the hummingbird is extremely rapid, the building of lipid stores from dietary sugar is also rapid when needed. For example, ruby-throated hummingbirds can routinely increase their mass by 15% or more between midday and dusk on a given day [9]. The ruby-throated hummingbird (*A. colubris*) completes an arduous annual migratory journey from breeding grounds as far north as Quebec in Canada to wintering grounds in Central America [10]. Hummingbirds are constrained to fueling long distance migratory flights using onboard lipids. In preparing for such flights, hummingbirds rapidly build fat stores prior to departure or at migratory stopover points, increasing their mass by 25-40% in as few as four days [9,11,12].

The ability to switch so completely and quickly between fuel types means these animals possess remarkably exquisite control over rates of substrate metabolism and biosynthesis in the liver, the principal site of lipogenesis in birds [13]. While hummingbird liver does indeed exhibit remarkably high activities of lipogenic and other metabolic enzymes [14], the mechanisms underlying high catalytic rates (high catalytic efficiency and/or high levels of enzyme expression) and control over flux (the role of hierarchical versus metabolic control), sensu [15], remain unclear.

Despite long-standing recognition of, and interest in, their extreme metabolism, the lack of knowledge about gene and protein sequences in hummingbirds has limited more detailed and mechanistic analyses. Amplification of hummingbird genetic sequences for sequencing and/or cloning is hampered by the lack of sequence information from closely related groups, making well-targeted primer design difficult. Only two genes have thus far been cloned from any hummingbird: an uncoupling protein (UCP) homolog and insulin [16,17]. These two studies offer limited insight into what adaptations in hepatic molecular physiology underlie extreme energy turnover or its regulation. The UCP homolog was cloned from pectoralis (flight muscle) and its functional significance *in vivo* is unclear. The amino acid sequence of hummingbird insulin was found to be largely identical to that from chicken; however, birds are insulin insensitive and lack the insulin-regulated glucose transporter (GLUT) protein GLUT4, making the role of this hormone in the regulation of energy homeostasis in hummingbirds unknown [18–20].

Recently completed sequencing of the Anna's hummingbird (*Calypte anna*) genome provides a powerful new tool in the arsenal of biologists seeking to understand variation in metabolic physiology in hummingbirds and other groups [21]. Despite their extreme catabolic and anabolic capabilities, hummingbirds have the smallest genomes among birds [22] and, in general, have

among the smallest vertebrate genomes [23]. Thus, it seems likely that understanding of transcriptional variation, overlaid on top of genetic variation, is crucial to understanding what makes these organisms such elite metabolic performers.

To this end, we produced the liver transcriptome of the ruby-throated hummingbird, *Archilochus colubris*. Because many of the proteins involved in cellular metabolism are quite large, we collaborated with Pacific Biosciences to generate long-read sequences as these would enhance our ability to identify full coding sequences and multiple encoded isoforms. The primary advantage to the Pacbio Iso-seq methodology is the capability for full-length transcript sequencing, rendering complete mRNA sequences without the need for assembly. This has been demonstrated in previous studies to dramatically increase detection of alternative splicing events [24]. Additionally, full-length sequences greatly enhance the likelihood of detecting novel or rare splice variants, which is crucial for fully characterizing the transcriptomes of lesser studied, non-model organisms such as the hummingbird.

## Methods

*Sacrifice and sample preparation*

A wild adult male ruby-throated hummingbird (*Archilochus colubris*) was captured at the University of Toronto Scarborough using modified box traps on July 23rd 2013 at 8:15AM. At the time of its capture, the bird was aged as an "after hatch year" bird, meaning it was at least 1 year old. Standard aging techniques make more precise aging of hummingbirds more than 1 year old difficult[25]. The bird was housed in the University of Toronto Scarborough vivarium and fed NEKTON-Nectar-Plus (Nekton, Tarpon Springs, FL, USA) ad libitum, and sacrificed after ad libitum feeding at 1:22PM on July 16th 2014 (being 2+ years old). On arrival it weighed 2.68g and at the time of sacrifice it weighed 3.11g. Tissues were sampled immediately after euthanization using RNAse-free tools. Liver tissue was dissected out and homogenized at 4ºC in 1 ml cold Tri Reagent using an RNase free glass tissue homogenizer and RNase free syringes of increasing needle gauge. We used 100 mg of tissue per 1 ml of Tri Reagent (Sigma-Aldrich, St. Louis, MO, USA), and chloroform extraction was performed twice to ensure quality. RNA was precipitated, centrifuged down, washed with ethanol, vacuum dried and eluted in RNAse free water. DNAse I digestion and spin column cleanup were performed. RNA concentration and RIN were determined with RNA Bioanalyzer (Agilent).

*Sequencing library preparation*

Pacific Bioscience's Iso-Seq sequencing protocol was followed to generate sequencing libraries [26]. The Clontech SMARTER cDNA synthesis kit with Oligo-dT primers was used to generate first and second-strand cDNA from polyA mRNA. After a round of PCR amplification, the amplified cDNA was size selected into 4 size fractions (1-2kb, 2-3kb, 3-6kb, and 5-10kb) to prevent preferential small template sequencing, using the Blue Pippin (Sage Sciences). Additional PCR cycles were used post size-selection to generate adequate starting material, and then SMRTbell hairpin adapters were ligated onto size-selected templates. Each of the 4 size fractions was

sequenced on 10 SMRT Cells, for a total of 40 SMRT Cells. Sequencing was performed by the JHU HiT Center using P6-C4 chemistry on the RSII sequencer.

## Analysis Methods

*Data processing, isoform clustering sorting and quality control of liver transcriptome*

We performed initial data processing using SMRTanalysis 3.1 IsoSeq pipeline employed using a DNANexus interface. From 40 SMRTcells, we produced 440.75 Gb of raw data, which was classified into 3.4Gb of non-chimeric CCS reads. CCS reads comprising 1.23M full length, 1.27M non-full-length reads, and were considered full-length if both 5' and 3' cDNA primers as well as the polyA tail signal were detected. Of the four size-selected bins, our average CCS length was 1533, 2464, 3650, and 5444 bp, respectively (Figure 1B). The Iso-Seq pipeline then performed isoform-level clustering (ICE) followed by final polishing using the Arrow algorithm to output high-quality (predicted accuracy >= 99%), full-length, isoform consensus sequences. The Iso-Seq pipeline produced 238Mb (807,104 reads) of high quality consensus isoforms (HQD, 94,724 reads), and 2Gb (712,210 reads) of low quality consensus isoforms (summary statistics Figure 1A). BLAST searches were then performed to remove putative contaminants, and then coding sequence and protein translation was performed, resulting in 93K HQ and 680 LQ protein sequences. A summary of the analyses performed are displayed in Figure 2A-B, and further details and settings can be found in Supplemental Methods.

*Assessing transcriptome completion*

To estimate the completeness of our liver transcriptome sequencing, we used both subsampling and gene diversity estimation, as well as BUSCO [27] [28]. BUSCO checks for essential single copy orthologs which should be present in a whole transcriptome dataset for any member of the given lineage. We used both Metazoan and Aves lineages (ortholog sets) to examine transcriptome completion (Figure 2C and Supplemental Table 1), and to ensure that completeness tracked across multiple data processing steps, we analyzed ASD (all sequence data), HQD (high quality data) and CCD (Cogent collapsed data). As expected, *Gallus gallus* and *Calypte anna* transcriptomes were nearly complete for both Aves and Metazoan BUSCO sets, and our *A. colubris* transcriptome only captured around half of this diversity, likely due to our sample being single tissue, collection time point and individual.

Our subsampling approach to estimating transcriptome completeness involved pulling subsets of the circular consensus reads dataset and BLASTed against the predicted *Calypte anna* gene set. We found that the number of unique genes detected began to saturate when reaching a 90% subset of our data, suggesting that additional sequencing would not substantially contribute to transcriptome completion (Supplemental Figure 1). Lower expressed genes may not be detected, but that vast majority of annotated liver expressed genes are likely represented in our data.

*Agreement with established Anna's hummingbird genomes reveals general clade conservation*

We aligned transcripts to the *Calypte anna* (Anna's hummingbird) genome using GMAP [29]. In order to validate transcript coverage and alignment throughout the multiple processing steps, we aligned using not only high quality isoforms (HQD), but also the full consensus isoform dataset (ASD) and gene families predicted by Cogent (CCD, methods in Supplemental methods and below).

*Calypte anna* and *Archilochus colubris* are close relatives within the North American Bee (Mellisugini) clade of hummingbirds [30]; *A. colubris* is a member of the Caribbean Sheartails subclade and *C. anna* is of the Calypte subclade, which diverged from the from ancestral Mellisugini around early to mid Pliocene [31]. Given this fairly recent divergence, we expected alignment to perform well. We found an average alignment identity of 94.8%, with 87% transcripts uniquely mapping to the reference. Of the uniquely mapped, 73% covered >90% of the query sequence (alignment length and statistics, Supplemental Figure 2A, 2B), demonstrating high fidelity of aligned reads to reference. When ASD reads were parsed by number of reads of insert supporting each consensus cluster, it was found that generally, alignment identity was high regardless of number of supporting reads. A clear increase in mean alignment identity was found when two or more supporting reads were collapsed (Supplemental Figure 3).

When GMAP was performed using only high quality isoforms (filtered for 2+ full-length supporting reads), alignment percentage was 95.7%, with 93.4% of transcripts mapping uniquely to the reference. The average mapped read length was 2411bp (HQD, 2617bp ASD), while the average predicted CDS length for *Calypte anna* was 1386bp. This being said, reads mapped with GMAP contain UTRs. When we predict just the CDS sequences for *A. colubris* using ANGEL[32], the mean length was 981bp. When we BLASTed the unaligned reads to whole NCBI database, they largely mapped back to *Calypte anna* (53%). This result suggests that our mapping parameters were too stringent to map these reads, error rate prevented alignment, unaligned regions are divergent enough between both hummingbirds to preclude alignment, or some combination of the above.

*Putative gene family prediction and reduction of transcript redundancy reduces data load while maintaining transcript diversity*

To assign transcripts to putative gene families, as well as cluster and eliminate redundant transcripts to produce a unique set of gene isoforms, we utilized the newly developed Cogent [33] pipeline. Cogent is specifically designed for transcriptome assembly in the absence of a reference genome, allowing for isoforms of the same gene to be distinctly identified from different gene families, which are defined as having more than two (possibly redundant) transcript copies. Of the 94,724 HQ consensus isoforms, 91,733 were grouped into 6,725 multi-transcript gene families (Figure 3A). The remaining 2,991 sequences were classified as putative single-isoform genes, or "orphans". Reconstructed contigs were then applied in place of a reference (or de novo clustering) to reduce redundant transcripts in the original HQD dataset. From this approach, we were able to reduce our HQ dataset to 14,628 distinct transcript isoforms and 2990 orphan isoforms, for a total of 17,618 isoform sequences (18% of the

original). Data is further summarized and most abundant transcripts are detailed in Supplemental Table 2. An average of 1.53 isoforms was found per gene family (Figure 3B), with 2624, or 27.4% of the gene families having more than one isoform, including "orphans". While other studies have found more isoforms per locus, for example 6.56 in [34], that study multiplexed six plant tissues, whereas a lower complexity is to be expected with single tissue analysis. This dataset (Cogent collapsed data, or CCD) was also mapped onto the *Calypte anna* genome assembly [35], to demonstrate the effectiveness of this method in reducing transcript redundancy and classifying isoforms (Figure 3C). Cogent gene families were polished using Illumina short read RNAseq data and the error correction algorithm Pilon [36] (Supplemental Methods) to obtain higher accuracy reads.

*Orthologous gene pair predictions and GO annotation show putative unique hummingbird orthologs*

To examine protein sequence similarity and divergence between *Archilochus colubris* and other avian species, we used OrthoMCL, which generates reciprocal best hits from comparison species using BLAST all-vs-all, then clustering to group orthologous sequences for each pair of organisms [37]. OrthoMCL protein sequences were predicted using ANGEL, and 119,292 high quality sequences were put into this analysis. We compared our ruby-throated hummingbird, *Archilochus colubris,* to five other birds: *Calypte anna* (Anna's hummingbird) fellow member of the bee clade of hummingbirds, *Chaetura pelagica* (chimney swift) the closest available outgroup species to the hummingbird clade, and other bird species for which relatively well-annotated genomes and/or transcriptomes are available, *Gallus gallus* (chicken), *Taeniopygia guttata* (zebra finch), and *Melopsittacus undulatus* (budgerigar), as well as *Homo sapiens* (human), and *Alligator mississippiensis* (American alligator). Algorithm parameters and data accession numbers are presented in Supplemental methods.

A matrix of ortholog pairings, with duplicate ortholog hits removed, shows counts of number of orthologous sequences for each species pair (Supplemental Table 3). Orthologs shared between ruby-throated hummingbird and a subset of the other species analyzed are illustrated in Figure 4A. Unsurprisingly, the largest amount of orthologs which pair closely to only one species, i.e., 1:1 orthologs, were found between Anna's and Ruby-throated hummingbird sequences. Surprisingly, the second-largest set was between chicken and ruby-throated hummingbird, as opposed to its closest outgroup species, *Chaetura pelagica*. This is likely due to the completeness of chicken transcriptome annotation, as chicken is the most well-studied avian species. Of the 596 unpaired *A. colubris* protein sequences, 190 paired most closely with *Calypte anna* when compared using BlastP and the majority of matches output (559/594) were less than 50 AA, only a fraction of the average sequence length.

In order to more closely examine the identity of orthologs in related hummingbird species, gene ontology (GO) annotation was performed on a specific set of orthologs which were shared between *Calypte anna* and *Archilochus colubris*, but not by the other birds included in the OrthoMCL analysis. This set of 2,376 protein sequences was run using BlastP and GO analysis performed by Panther [38,39]. Additional datasets used for GO comparison included 1:1 orthologs

for *Gallus gallus* and *A. colubris* (518), and *A. colubris* and *Chaetura pelagica* (430), as well as whole transcriptome data from *C. pelagica* and Cogent-collapsed dataset from our transcriptome (Supplemental Table 4, Figure 4B).

As the initial impetus for our investigation centered on the exceptional metabolism and energetics of hummingbirds, we focused our investigation on orthologs tagged as part of the “metabolic process (GO:0008152)” grouping. Of the 1444 orthologs identified in *Archilochus colubris* as part of this process grouping, 236 (16.3%) were unique to hummingbirds. Within this top-level grouping, the largest number of genes group under “primary metabolic processes” (GO:0044238)”. Of the 1240 orthologs identified within this grouping, 204 (16.3%) are identified as uniquely shared by our hummingbird species. Six GO biological processes are defined under the “primary metabolic processes”. Of these processes, the process with the highest proportion of identified *A. colubris* orthologs hitting as unique to the two hummingbird species is “lipid metabolic processes” (GO:0006629; 33 of 114 orthologs, 28.9%), which is significantly enriched relative to the comparative orthology databases of both chicken and human (Statistical overrepresentation test, Panther, [38], p-values given in Supplemental table 4). Because we considered it likely that an enrichment in lipid metabolic genes could be a result of our dataset being from liver tissue, we compared enrichment with that of the entire Cogent predicted gene set from the ruby-throated hummingbird transcriptome, and found no significant enrichment using the same tests (Supplemental table 4). Because 1:1 hummingbird orthologs are relatively more abundant in lipid metabolic genes than the sequences which were found to be highly homologous to one or more of the other species compared using OrthoMCL, we predict that lipid metabolic genes are more divergent from the other examined species than other classes of enzymes. However, the proportion of identified genes within a biological pathway classified as orthologs unique to hummingbirds should not be taken as direct evidence of greater selection on proteins within that pathway. Yet, if neutral sequence divergence is assumed to be randomly accrued throughout a species’ genome, then greater divergence in enzymes making up “lipid metabolic processes” suggests that closer examination of these proteins for evidence of functional, or even adaptive, divergence is warranted. A phylogenetically-informed analysis of ortholog divergence among taxa is necessary to establish a selection signature, which will become possible in the future with the advance of the B10K project [40] and larger numbers of avian species in GO databases.

Given the apparent sequence divergence among enzymes involved in “lipid metabolic processes” hinted at by orthology and ontology analyses, we elected to more closely examine enzymes comprising the lipogenic pathway. In liver, fatty acids can be synthesized via the *de novo* lipogenesis pathway using acetyl CoA as substrate. These newly synthesized fatty acids can then be esterified onto the glycerophosphate backbone to generate triglycerides via the glycerol-3-phosphate pathway of lipid synthesis. We predicted that key enzymes involved in these two pathways (Figure 5A) would be divergent in hummingbirds given their extraordinary metabolic demands. Eight enzymes involved in this pathway were examined for *Archilochus colubris, Calypte anna, Gallus Gallus, Chaetura pelagica*, *Alligator mississippiensis* and *Homo sapiens (*accession numbers and details given in Supplemental Table 5). Pairwise protein

alignment scores are given in Supplemental Table 6 as well as illustrated in a heatmap shown in Figure 5B, and alignments in Supplemental Data 1. Interestingly, enzymes involved in *de novo* fatty acid synthesis share higher degree of identity between examined organisms, whereas enzymes involved in triglyceride synthesis tend to be slightly less conserved (Figure 5A). Figure 5B also shows normalized abundances of the enzymes of interest in our liver transcriptome dataset, revealing high expression level of the rate-setting enzyme involved in *de novo* lipogenesis (*ACACA*; acetyl CoA carboxylase). In contrast to the cytosolic *ACACA* enzyme that uses acetyl-CoA as substrates for fatty acid synthesis, *MCAT* encodes a mitochondrial enzyme that uses malonyl-CoA as substrates for fatty acid synthesis. Much less is known about the MCAT-dependent pathway of fatty acid synthesis in mitochondria. Interestingly, MCAT has the lowest relative abundance in ruby throated hummingbird liver. The relative hepatic expression levels of triglyceride synthesis genes (e.g., *LPIN1* and *DGAT2*) are also much lower compared to genes involved in *de novo* lipogenesis (*ACACA* and *FASN*). It is important to note that most metabolic enzymes are tightly regulated. The relative levels of hepatic lipogenesis enzymes may vary greatly depending on the time of day and the physiological states (fast vs. fed) of the animals.

In order to further investigate degree of conservation between key hepatic lipogenesis enzymes in hummingbirds and comparative organisms, we performed conservation analysis and determined ratio of nonsynonymous to synonymous codon changes (dN/dS) as a metric of positive selection, using pairwise alignments followed by the CodeML module in PAML4[41]. These ratios are given in Supplemental Table 6 and plotted in a heatmap in Figure 5B. A dN/dS score > 1 denotes genomic regions putatively undergoing positive selection. We found, in general, good conservation of these enzymes between organisms, with the exception of the 3' and 5' ends of alignments. These often had an extended or retracted coding sequence in the case of hummingbirds and *C. pelagica*, which could be post-translational modification or selection on pathway regulation [42]. Surprisingly, terminal sequence length was variable even between *C. anna* and *A. colubris,* which both belong to the closely-related Bee hummingbird taxon [30]. Variation in 5' and 3' length may also be an effect of the different methodologies used to produce these sequences, RNA sequencing for *A. colubris, G. gallus,* and *H. sapiens*, and ORF prediction from genomic data for the other organisms examined. For example, we note in our analysis that *MCAT* appears more conserved between *A. colubris* and *H. sapiens*, than between *A. colubris* and *C. anna*, which could be due not to *A. colubris* actually being more similar to *H. sapiens*, but rather to ORF prediction oversights

The averaged dN/dS values, while useful for comparison, can be misleading when considered over the entire gene, as 3' and 5' variation can overshadow conserved motifs, and pairwise comparisons are limited in scope. This type of analysis is ideal for very divergent sequences, but less informative for pairs of sequences that are highly similar [43]. Despite this, conservation analysis is still valuable and provides insights connecting nucleotide to amino acid information that alignments alone can miss. For example, lysophosphatidic acid acyltransferase (*ABHD5*), which functions primarily in phosphatidic acid biosynthesis, has reasonable protein alignment scores to all comparative organisms but also shows positive selection acting upon this gene

relative to *Calypte anna*, swift, human and alligator, but not chicken (Figure 5B). This led us to more closely examine coding sequence alignment, to find that the bulk of differences in coding sequence were attributable to the exon 1, with alignment largely becoming synchronous (with the exception of *H. sapiens*, which is widely divergent) by exon 2 and continuing through to the end of the transcript. Although the primary AB hydrolase-1 domain is very well conserved between species, these differences in exon 1 could be functionally significant, and honing down to regions of differentiation between comparative species gives us interesting starting points for future investigations, including the cloning and enzyme kinetics studies of *ABHD5*. Additionally, pairwise comparisons provide interesting observations, such as coding strand elongation in the 5' region in *A. colubris GPAM* (Supplemental Data 2). This information can be leveraged for future studies examining enzyme structure, function and evolution.

*Transcriptome resource mining could provide functional genomic insights*

Access to the transcriptome informs the investigation of biological processes and enables the formation of new hypotheses. This is exemplified by the serendipitous observation that hummingbird glucose transporter 2 (*GLUT2*) lacks a N-glycosylation site due to an asparagine to aspartic acid amino acid substitution. This missing glycosylation site was also seen in the available Anna's hummingbird genome. All class 1 glucose transporters studied in model vertebrates contain one N-glycosylation site located on the large extracellular loop of the protein [44]. In GLUT2 the associated glycan interacts with the glycan-galectin lattice of the cell, stabilizing cell surface expression [45]. Removal of the N-glycan of GLUT2 in rat pancreatic β cells results in the sequestering of cell-surface GLUT2 in lipid rafts and this sequestered GLUT2 exhibits a reduction in glucose transport activity by approximately 25% [45]. This reduction in transport is thought to occur through interaction of the *GLUT* with lipid raft-bound stomatin [45,46]. In mammals, GLUT2 serves a glucose-sensing role in the pancreatic β cells and is required for the regulation of blood glucose through insulin and glucagon [47]. The lack of N-glycosylation of GLUT2 may contribute to observed high blood glucose concentration in hummingbirds [48].

*Re-use potential*

In conclusion, our results have leveraged cutting-edge technology to provide a compelling first direct look at the transcriptome of this incredible organism. By using PacBio sequencing, we have been able to generate full length cDNA transcripts from the hummingbird liver. Transcriptome data generated using the Iso-seq methodology, when coupled to sophisticated recently developed gene synthesis techniques [49], allows for simple generation of relevant isoforms for biochemical experiments. Some of the key metabolic enzymes identified from our work as being unique to either *A. colubris* or at most common to *C. anna* and *A. colubris* could now be quickly cloned and expressed. Follow up studies will allow for biochemical studies of proteins generated directly from our transcriptome data, measuring their enzymatic properties, e.g. $k_{cat}$ or $V_{max}$, as compared to other avian or mammalian analogues [14,50,51]. Expressed proteins may also be used for structural biology studies, applying either x-ray crystallography or cryoEM

to generate structural maps of the proteins, and examine how the structure compares to other analogues.

*Availability of supporting data*

Filtered fastq of clustered CCS reads deposited in SRA accession number SRP099041. Predicted Cogent gene families, coding sequence and annotations, peptide and untranslated region data are available at 10.5281/zenodo.781592. All other data available upon request.

*Availability and requirements*

Project name: Ruby_isoseq

Project home page: https://github.com/reworkman/hummingbird

Operating system: Unix

Programming language: Bash, Python, R

Other requirements: BUSCO, GMAP, Blast+, ANGEL, CLUSTAL, Cogent, and their dependencies

License: None

**Acknowledgments**

Pacific Biosciences for reagents and SMRTcells as well as technical support. M. Schatz, E. Jarvis, J. Korlach, Y. Guo for discussion. HFSP grant #RGP0062/2016. Natural Sciences and Engineering Research Council of Canada Discovery Grant (#386466) to KCW.

**Disclosure Declaration**

W.T. and R.W. have received travel funds to speak at symposia organized by Pacific Biosciences. Bulk of reagents for IsoSeq were provided by Pacific Biosciences.

**Figure legends**

**Figure 1.** Transcriptome dataset quality control reveals good throughput, read length, and transcriptome completion. Average read lengths and isoform counts for 4 sequenced size fractions given in **A**, and read length distribution for all sequence data (ASD, is all sequence data, high quality (HQ) and low quality (LQ) isoforms) on x vs read counts on y plotted in **B**, with black line representing Mb data greater than read length. For example, at 2000bp, 4000Mb of sequence data was larger than 2000bp. **C**. BUSCO transcriptome assessment results displayed for *Archilochus colubris* (ruby-throated hummingbird, all sequence data ASD, high quality sequence data HQD), Cogent-collapsed data (CCD), *Calypte anna* (Anna's hummingbird), *Gallus gallus* (chicken) Thomas (single-tissue transcriptome) illustrate transcriptome completion

relative to predicted single copy ortholog datasets for both the Class Aves and Kingdom Metazoa.

**Figure 2.** Pipeline details analysis steps, as well as amount of data present at each step (in green text). **A** Raw sequence reads from a Pacbio RSII sequencer (bax.h5, bas.h5) were sorted into full and non-full length reads of insert (ROI) using a classification algorithm that identified full length reads with forward and reverse primers, as well as a poly-A tail. Iterative clustering for isoforms (ICE) was performed on full length reads, and non-full length reads were recruited to perform ARROW polished on the consensus isoforms. Polishing sorted reads into high and low-quality bins, and either high quality data (HQD), all sequence data (ASD) or both sets of data, were carried on to further applications (**B**). Applications include ORF and protein sequence generation from high quality (HQD) and low quality (LQD) consensus isoforms, alignment to *C. anna* reference with GMAP of both high quality data (HQD) and Cogent-collapsed data (CCD), detection of orthologous sequences (orth groups) using OrthoMCL, and prediction of gene families (gene fam) using Cogent. Numbers of available reads at each analysis step is displayed in green in each bubble.

**Figure 3.** Reducing transcript redundancy and predicting gene families using Cogent software. **A.** Gene families predicted and classified by relationship to *Calypte anna* genome assembly shown, along with statistics for alignment using GMAP software which show excellent alignment to closely related hummingbird reference species *Calypte anna*. Cogent comparison cases highlight the relationships between predicted gene families and *C. anna* reference (In ref), and demonstrate the additional information given to an assembly by transcriptome information. Number of isoforms predicted per gene family (unigene) given in **B** shows relatively low isoform diversity in this tissue. **C** Alignment of the MATR3 gene demonstrates the redundancy reducing capabilities of the Cogent software, which was reduced from 11 semi-redundant reads to 3 unique isoforms using this pipeline.

**Figure 4.** Orthology analysis. The proteomes of five birds (Anna's hummingbird:*Calypte anna*, Zebrafinch: *Tinamus guttatus*, Chicken: *Gallus gallus*, Swift: *Chaetura pelagica*, and Budgeridger:*Melopsitticus undulatus*), one mammal (*Homo sapiens*) and one reptile (*Alligator mississippiensis*) were compared against *Archilochus colubris* using OrthoMCL to detect homologous sequences. A Venn diagram illustrating sequences with best reciprocal blast hits between the given species and *A. colubris* is shown in **A.** Bar chart illustrates observed/expected ratios of metabolism enzymes (GO group: metabolic process 0008152) for comparison groups (statistical overrepresentation test) for selected OrthoMCL groups using Panther. Datasets input either include the entire proteome of target species (swift all, anna's all) or distinct set of homologs shared two groups (Ex. *A. colubris* + *C. anna* are homologs shared between these two species but not any of the other comparison groups). Asterisks denote significant overrepresentation of metabolic process proteins relative to expected baseline ($p<0.05$)(**B**).

**Figure 5.** Pathway analysis of key enzymes in hepatic lipogenesis. **A** An overview of the relationship between the investigated genes and their roles in triacylglycerol, phospholipid and

fatty acid synthesis, **B** heat maps illustrating percent amino acid identity of these proteins relative to *Archilochus colubris* predicted sequences, abundances (log2(reads per 10000) transformed) of their transcripts, and dN/dS (ratio of synonymous to nonsynonymous gene mutations). Taken together, these illustrate the complex relationships between target proteins and identity, conservation and abundance.

**References:**


1. Suarez, R. K. Hummingbird flight: sustaining the highest mass-specific metabolic rates among vertebrates. *Experientia* **48,** 565–570 (1992).
2. Chai, P. & Dudley, R. Limits to flight energetics of hummingbirds hovering in hypodense and hypoxic gas mixtures. *J. Exp. Biol.* **199,** 2285–2295 (1996).
3. Suarez, R. K. *et al.* Fuel selection in rufous hummingbirds: ecological implications of metabolic biochemistry. *Proc. Natl. Acad. Sci. U. S. A.* **87,** 9207–9210 (1990).
4. Chen, C. C. W. & Welch, K. C. Hummingbirds can fuel expensive hovering flight completely with either exogenous glucose or fructose. *Funct. Ecol.* **28,** 589–600 (2014).
5. Welch, K. C., Jr, Altshuler, D. L. & Suarez, R. K. Oxygen consumption rates in hovering hummingbirds reflect substrate-dependent differences in P/O ratios: carbohydrate as a 'premium fuel'. *J. Exp. Biol.* **210,** 2146–2153 (2007).
6. Baker, H. G. Sugar Concentrations in Nectars from Hummingbird Flowers. *Biotropica* **7,** 37–41 (1975).
7. Welch, K. C., Jr & Chen, C. C. W. Sugar flux through the flight muscles of hovering vertebrate nectarivores: a review. *J. Comp. Physiol. B* **184,** 945–959 (2014).
8. Powers, D. R., Brown, A. R. & Van Hook, J. A. Influence of normal daytime fat deposition on laboratory measurements of torpor use in territorial versus nonterritorial hummingbirds. *Physiol. Biochem. Zool.* **76,** 389–397 (2003).
9. Hou, L., Verdirame, M. & Welch, K. C. Automated tracking of wild hummingbird mass and energetics over multiple time scales using radio frequency identification (RFID) technology.

*J. Avian Biol.* **46,** 1–8 (2015).

10. Weidensaul, S., Robinson, T. R., Sargent, R. R., Sargent, M. B. & Poole, A. Ruby-throated hummingbird (Archilochus colubris). *Birds of North America Online (A. Poole, Editor). Cornell Lab of Ornithology, Ithaca, NY, USA. http://bna. birds. cornell. edu/bna/species/204* (2013).
11. Carpenter, F. L., Hixon, M. A., Beuchat, C. A., Russell, R. W. & Paton, D. C. Biphasic Mass Gain in Migrant Hummingbirds: Body Composition Changes, Torpor, and Ecological Significance. *Ecology* **74,** 1173–1182 (1993).
12. Hou, L. & Welch, K. C., Jr. Premigratory ruby-throated hummingbirds, Archilochus colubris, exhibit multiple strategies for fuelling migration. *Anim. Behav.* **121,** 87–99 (2016).
13. Hermier, D. Lipoprotein metabolism and fattening in poultry. *J. Nutr.* **127,** 805S–808S (1997).
14. Suarez, R. K., Brownsey, R. W., Vogl, W., Brown, G. S. & Hochachka, P. W. Biosynthetic capacity of hummingbird liver. *Am. J. Physiol.* **255,** R699–702 (1988).
15. ter Kuile, B. H. & Westerhoff, H. V. Transcriptome meets metabolome: hierarchical and metabolic regulation of the glycolytic pathway. *FEBS Lett.* **500,** 169–171 (2001).
16. Vianna, C. R. *et al.* Cloning and functional characterization of an uncoupling protein homolog in hummingbirds. *Physiol. Genomics* **5,** 137–145 (2001).
17. Fan, L., Gardner, P., Chan, S. J. & Steiner, D. F. Cloning and analysis of the gene encoding hummingbird proinsulin. *Gen. Comp. Endocrinol.* **91,** 25–30 (1993).
18. Welch, K. C., Jr, Allalou, A., Sehgal, P., Cheng, J. & Ashok, A. Glucose transporter expression in an avian nectarivore: the ruby-throated hummingbird (Archilochus colubris). *PLoS One* **8,** e77003 (2013).
19. Braun, E. J. & Sweazea, K. L. Glucose regulation in birds. *Comp. Biochem. Physiol. B*

*Biochem. Mol. Biol.* **151,** 1–9 (2008).

20. Polakof, S., Mommsen, T. P. & Soengas, J. L. Glucosensing and glucose homeostasis: from fish to mammals. *Comp. Biochem. Physiol. B Biochem. Mol. Biol.* **160,** 123–149 (2011).
21. Jarvis, E. D. *et al.* Phylogenomic analyses data of the avian phylogenomics project. *Gigascience* **4,** 4 (2015).
22. Gregory, T. R., Andrews, C. B., McGuire, J. A. & Witt, C. C. The smallest avian genomes are found in hummingbirds. *Proc. Biol. Sci.* **276,** 3753–3757 (2009).
23. Hughes, A. L. & Hughes, M. K. Small genomes for better flyers. *Nature* **377,** 391 (1995).
24. Abdel-Ghany, S. E. *et al.* A survey of the sorghum transcriptome using single-molecule long reads. *Nat. Commun.* **7,** 11706 (2016).
25. Pyle, P. Identification Guide to North American Birds, Part I: Columbidae to Ploceidae: Peter Pyle: 9780961894023: Books - Amazon.ca. Available at: https://www.amazon.ca/Identification-Guide-North-American-Birds/dp/0961894024. (Accessed: 1st October 2017)
26. Thomas, S., Underwood, J. G., Tseng, E., Holloway, A. K. & Bench To Basinet CvDC Informatics Subcommittee. Long-read sequencing of chicken transcripts and identification of new transcript isoforms. *PLoS One* **9,** e94650 (2014).
27. Assessing genome assembly and annotation completeness with Benchmarking Universal Single-Copy Orthologs (BUSCO). Available at: https://gitlab.com/ezlab/busco.
28. Simão, F. A., Waterhouse, R. M., Ioannidis, P., Kriventseva, E. V. & Zdobnov, E. M. BUSCO: assessing genome assembly and annotation completeness with single-copy orthologs. *Bioinformatics* **31,** 3210–3212 (2015).
29. Wu, T. D. & Watanabe, C. K. GMAP: a genomic mapping and alignment program for mRNA

and EST sequences. *Bioinformatics* **21,** 1859–1875 (2005).

30. McGuire, J. A., Witt, C. C., Altshuler, D. L. & Remsen, J. V., Jr. Phylogenetic systematics and biogeography of hummingbirds: Bayesian and maximum likelihood analyses of partitioned data and selection of an appropriate partitioning strategy. *Syst. Biol.* **56,** 837–856 (2007).

31. Licona-Vera, Y. & Ornelas, J. F. The conquering of North America: dated phylogenetic and biogeographic inference of migratory behavior in bee hummingbirds. *BMC Evol. Biol.* **17,** 126 (2017).

32. Tseng, E. Robust Open Reading Frame prediction (ANGLE re-implementation). Available at: https://github.com/PacificBiosciences/ANGEL.

33. Coding Genome Reconstruction using Iso-Seq data. *Elizabeth Tseng* Available at: https://github.com/Magdoll/Cogent.

34. Wang, B. *et al.* Unveiling the complexity of the maize transcriptome by single-molecule long-read sequencing. *Nat. Commun.* **7,** 11708 (2016).

35. Zhang, G. *et al.* Genomic data of the Anna's Hummingbird (Calypte anna). (2014). doi:10.5524/101004

36. Walker, B. J. *et al.* Pilon: an integrated tool for comprehensive microbial variant detection and genome assembly improvement. *PLoS One* **9,** e112963 (2014).

37. Li, L., Stoeckert, C. J., Jr & Roos, D. S. OrthoMCL: identification of ortholog groups for eukaryotic genomes. *Genome Res.* **13,** 2178–2189 (2003).

38. Mi, H., Muruganujan, A., Casagrande, J. T. & Thomas, P. D. Large-scale gene function analysis with the PANTHER classification system. *Nat. Protoc.* **8,** 1551–1566 (2013).

39. Mi, H. *et al.* PANTHER version 11: expanded annotation data from Gene Ontology and Reactome pathways, and data analysis tool enhancements. *Nucleic Acids Res.* **45,**

D183–D189 (2017).

40. Zhang, G. *et al.* Genomics: Bird sequencing project takes off. *Nature* **522,** 34 (2015).

41. Yang, Z. PAML 4: phylogenetic analysis by maximum likelihood. *Mol. Biol. Evol.* **24,** 1586–1591 (2007).

42. Jacob, E. & Unger, R. A tale of two tails: why are terminal residues of proteins exposed? *Bioinformatics* **23,** e225–30 (2007).

43. Kryazhimskiy, S. & Plotkin, J. B. The population genetics of dN/dS. *PLoS Genet.* **4,** e1000304 (2008).

44. Joost, H. G. & Thorens, B. The extended GLUT-family of sugar/polyol transport facilitators: nomenclature, sequence characteristics, and potential function of its novel members (review). *Mol. Membr. Biol.* **18,** 247–256 (2001).

45. Ohtsubo, K. *et al.* N-Glycosylation modulates the membrane sub-domain distribution and activity of glucose transporter 2 in pancreatic beta cells. *Biochem. Biophys. Res. Commun.* **434,** 346–351 (2013).

46. Zhang, J. Z., Abbud, W., Prohaska, R. & Ismail-Beigi, F. Overexpression of stomatin depresses GLUT-1 glucose transporter activity. *Am. J. Physiol. Cell Physiol.* **280,** C1277–83 (2001).

47. Thorens, B. & Mueckler, M. Glucose transporters in the 21st Century. *Am. J. Physiol. Endocrinol. Metab.* **298,** E141–5 (2010).

48. Beuchat, C. A. & Chong, C. R. Hyperglycemia in hummingbirds and its consequences for hemoglobin glycation. *Comp. Biochem. Physiol. A Mol. Integr. Physiol.* **120,** 409–416 (1998).

49. Kosuri, S. & Church, G. M. Large-scale de novo DNA synthesis: technologies and applications. *Nat. Methods* **11,** 499–507 (2014).

50. Suarez, R. K., Welch, K. C., Jr, Hanna, S. K. & Herrera M, L. G. Flight muscle enzymes and metabolic flux rates during hovering flight of the nectar bat, Glossophaga soricina: further evidence of convergence with hummingbirds. *Comp. Biochem. Physiol. A Mol. Integr. Physiol.* **153,** 136–140 (2009).

51. FernándezM.J., BozinovicF. & SuarezR.K. Enzymatic flux capacities in hummingbird flight muscles: a ‘one size fits all’ hypothesis. *Can. J. Zool.* **89,** 985–991 (2011).

A]

| Size Fraction | 1-2kb | 2-3kb | 3-6kb | 5-10kb | Total |
|---|---|---|---|---|---|
| # of SMRTcells | 10 | 10 | 10 | 10 | 40 |
| Reads of Insert (ROI) | 688,069 | 591,050 | 735,670 | 625,194 | 2,639,983 |
| Avg length ROI (bp) | 1533 | 2464 | 3650 | 5444 | |
| ROI Yield (Mbp) | 1055 | 1457 | 2685 | 3404 | 8601 |
| Filtered full length reads | 430,381 | 306,841 | 272,781 | 193,906 | 1,203,909 |
| # Consensus Isoforms | 359,981 | 163,618 | 209,969 | 121,109 | 807,114 |
| HQ consensus isoforms | 41,763 | 25,776 | 24,735 | 7,436 | 94,724 |
| % HQ | 11.60% | 15.75% | 11.78% | 6.14% | 11.74% |
| Avg HQ length | 1315 | 2329 | 3629 | 5491 | |
| LQ consensus isoforms | 321,101 | 135,415 | 186,523 | 113,162 | 712,210 |
| % LQ | 89.20% | 82.76% | 88.83% | 93.44% | 88.56% |
| Avg LQ length | 1503 | 2621 | 4170 | 6718 | |

B]

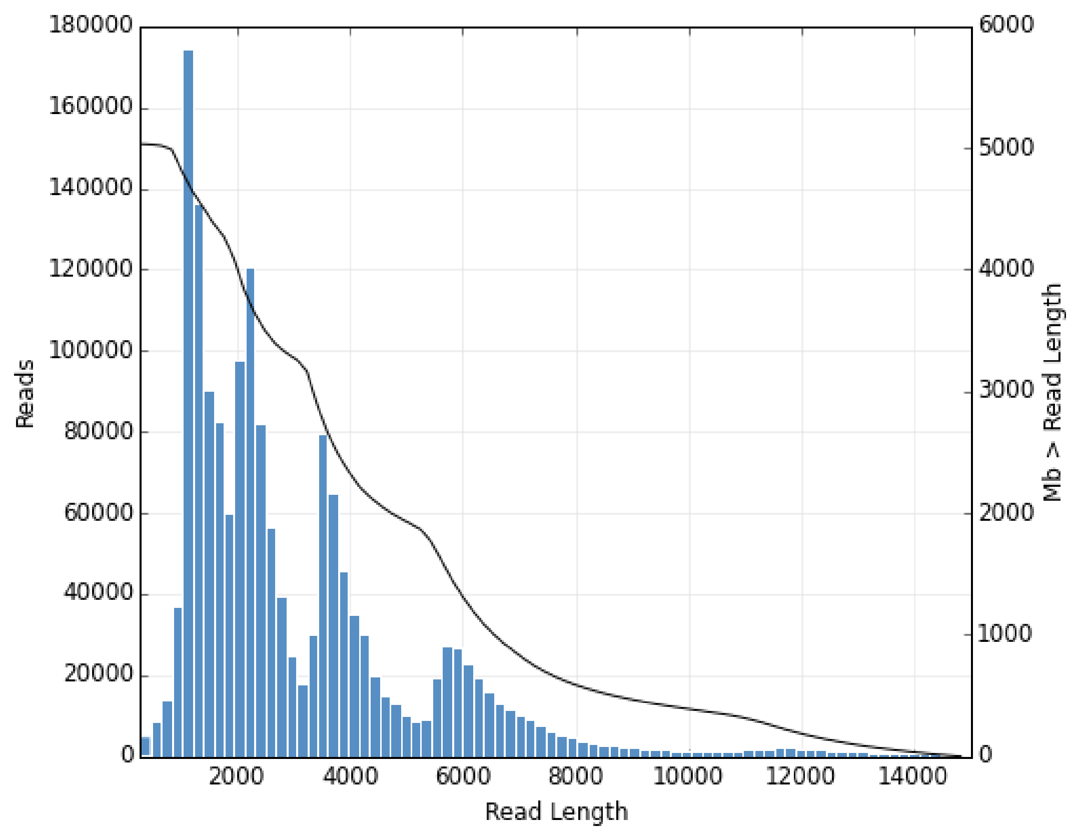


C]

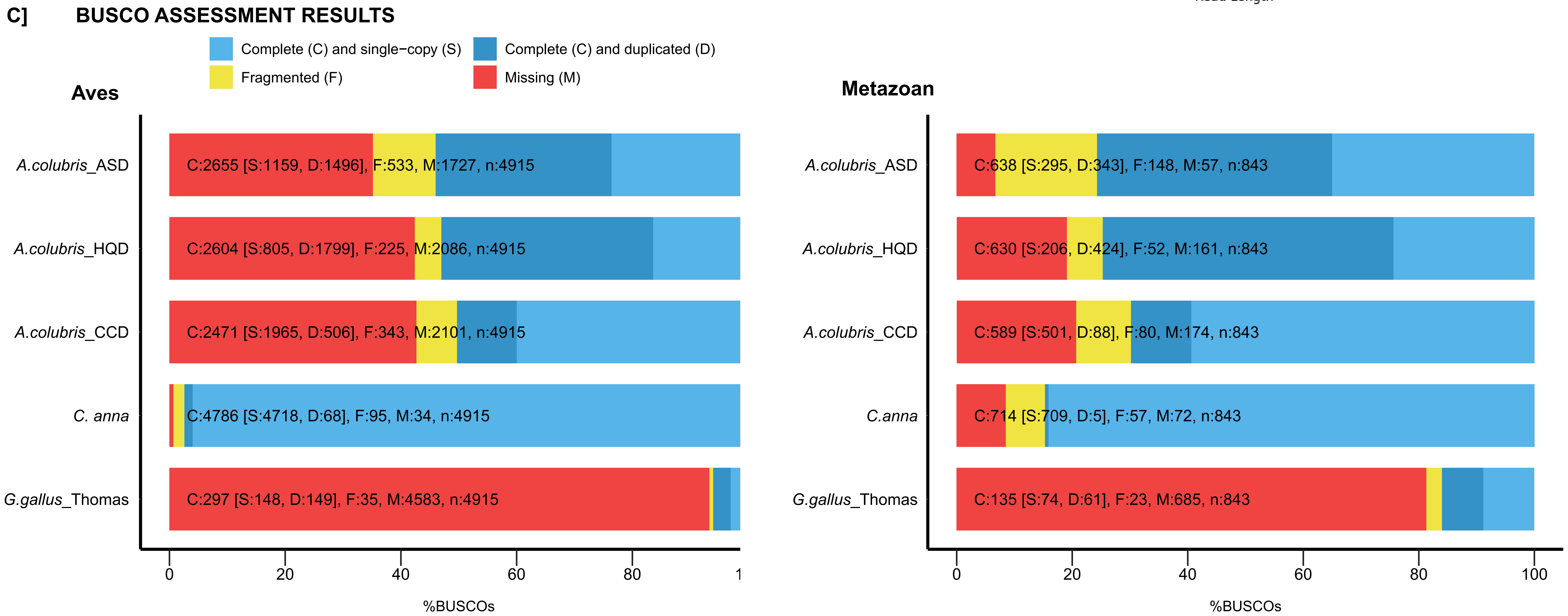

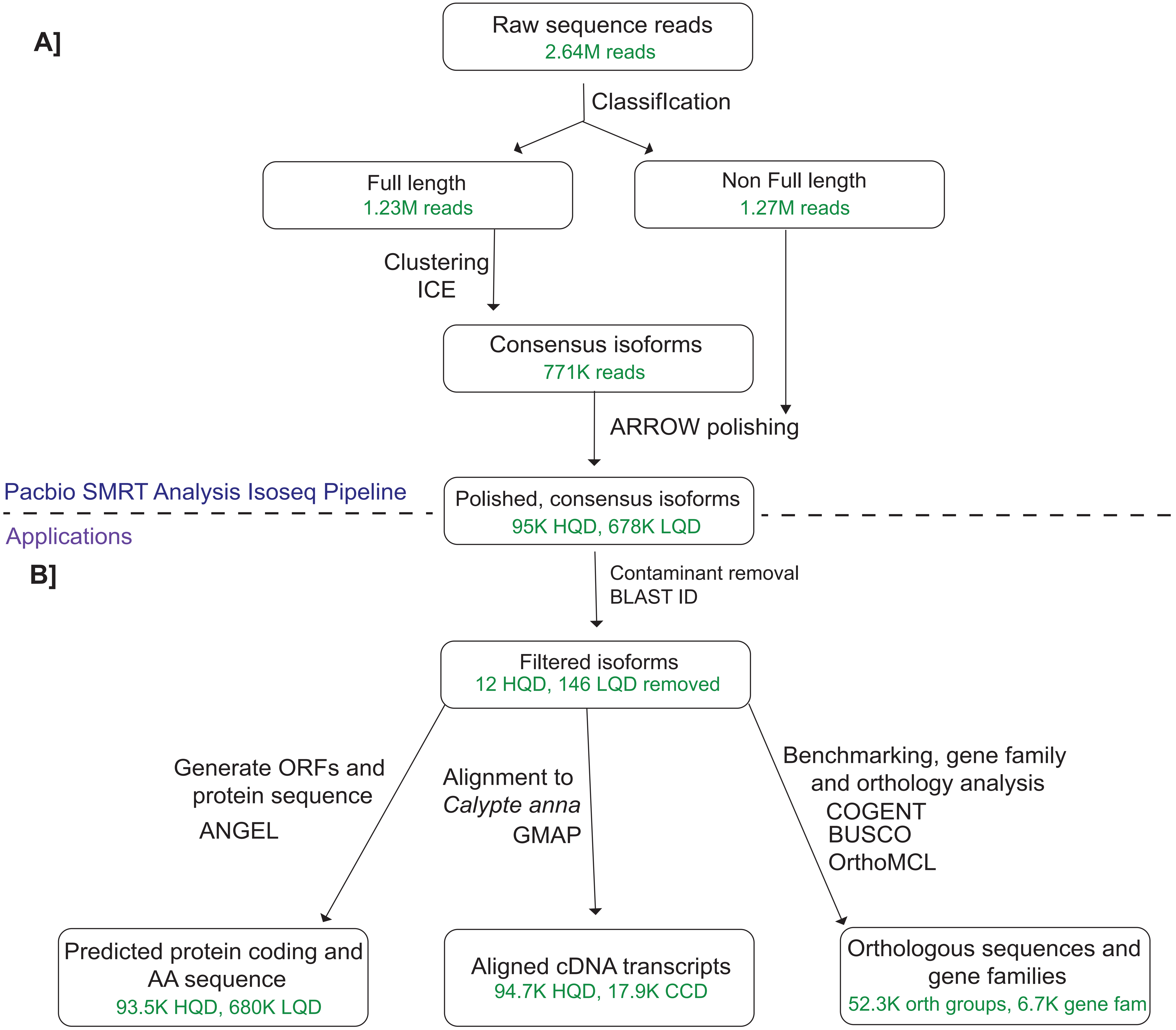
A]
Raw sequence reads
2.64M reads
ClassifIcation
Full length
1.23M reads
Non Full length
1.27M reads
Clustering
ICE
Consensus isoforms
771K reads
ARROW polishing
Pacbio SMRT Analysis Isoseq Pipeline
Polished, consensus isoforms
95K HQD, 678K LQD
Applications
B]
Contaminant removal
BLAST ID
Filtered isoforms
12 HQD, 146 LQD removed
Generate ORFs and
protein sequence
ANGEL
Alignment to
*Calypte anna*
GMAP
Benchmarking, gene family
and orthology analysis
COGENT
BUSCO
OrthoMCL
Predicted protein coding and
AA sequence
93.5K HQD, 680K LQD
Aligned cDNA transcripts
94.7K HQD, 17.9K CCD
Orthologous sequences and
gene families
52.3K orth groups, 6.7K gene fam

A]

| Clustering results | Counts | | |
|---|---|---|---|
| Total high quality (HQ) Isoforms | 94724 | | |
| Total grouped by Cogent | 91733 | | |
| Orphan seqs (likely single-isoform) | 2991 | | |
| Gene families predicted | 6727 | | |
| **Gene family alignment: GMAP** | **Counts** | **Percent** | |
| Unaligned | 1068 | 5.97% | |
| Multi-mapped | 2614 | 14.62% | |
| Uniquely Mapped | 15262 | 85.37% | |
| qCoverage = 100% | 10076 | 56.36% | |
| qCoverage >= 99%: | 14018 | 78.41% | |
| qCoverage >= 90% | 14559 | 81.44% | |
| Total number transcripts | 17877 | 100.00% | |
| **Cogent comparison cases** | **In Cogent** | **In Ref** | **#families** |
| Single gene locus | 1 | 1 | 5258 |
| Missing gene, possible broken | 1 | >1 | 176 |
| Missing gene | 1 | 0 | 38 |
| Unresolvable to 1 contig | >1 | 1 | 836 |
| Possible multi-loci gene | >1 | >1 | 419 |
| Total gene families | | | 6727 |

B]

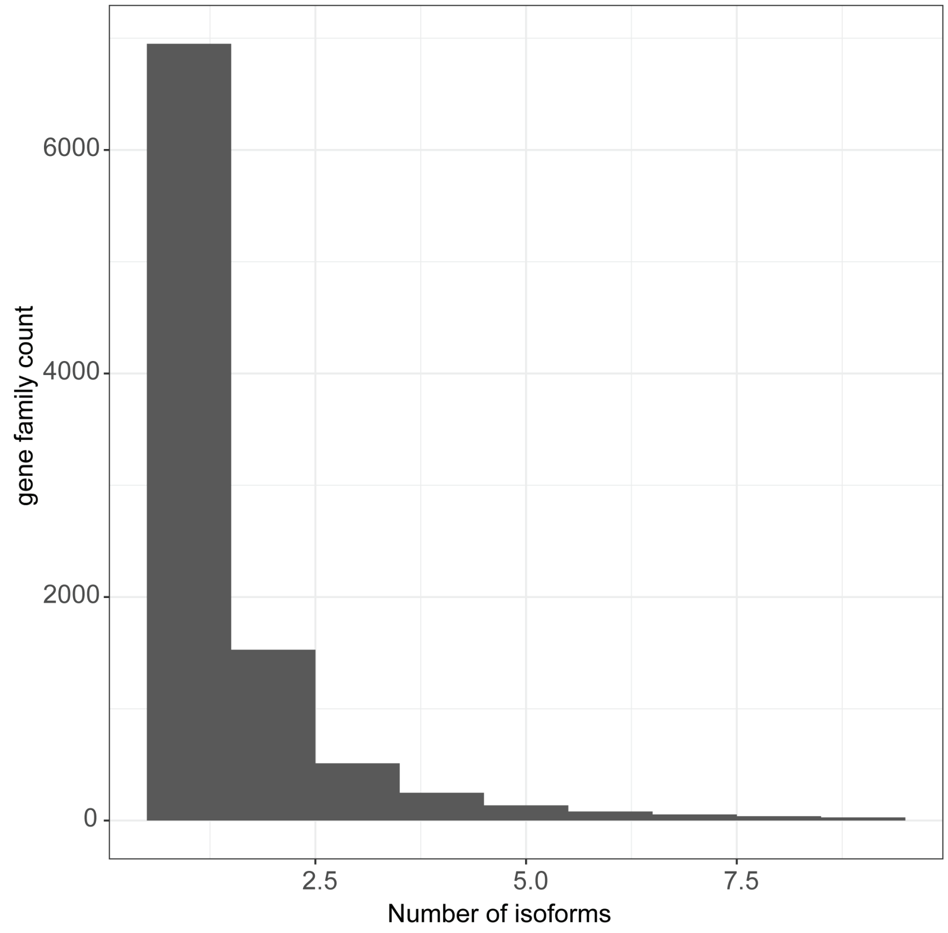


C]

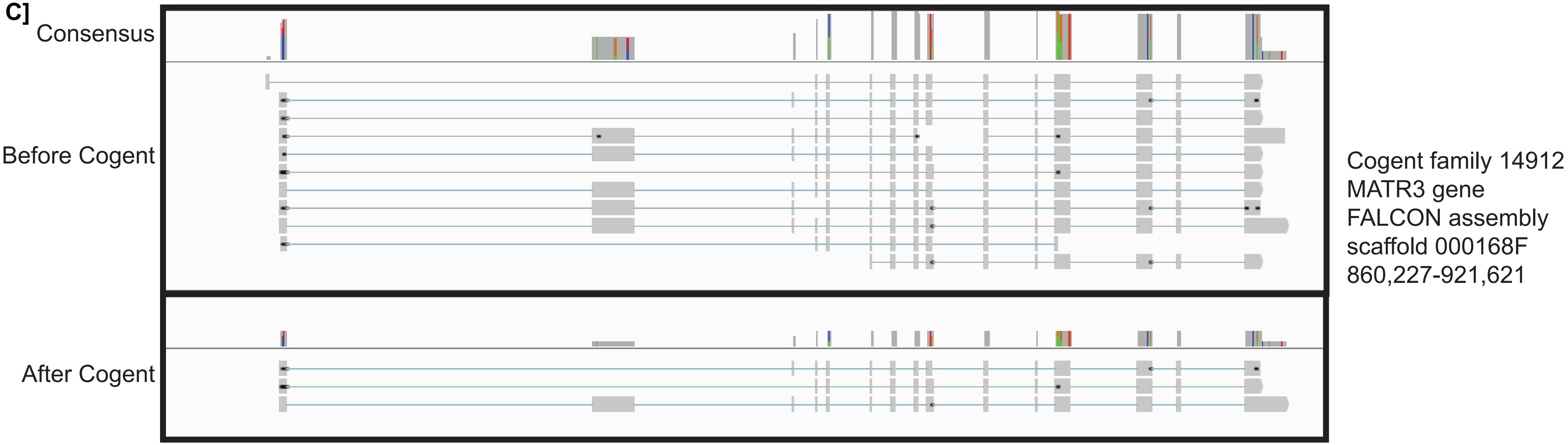

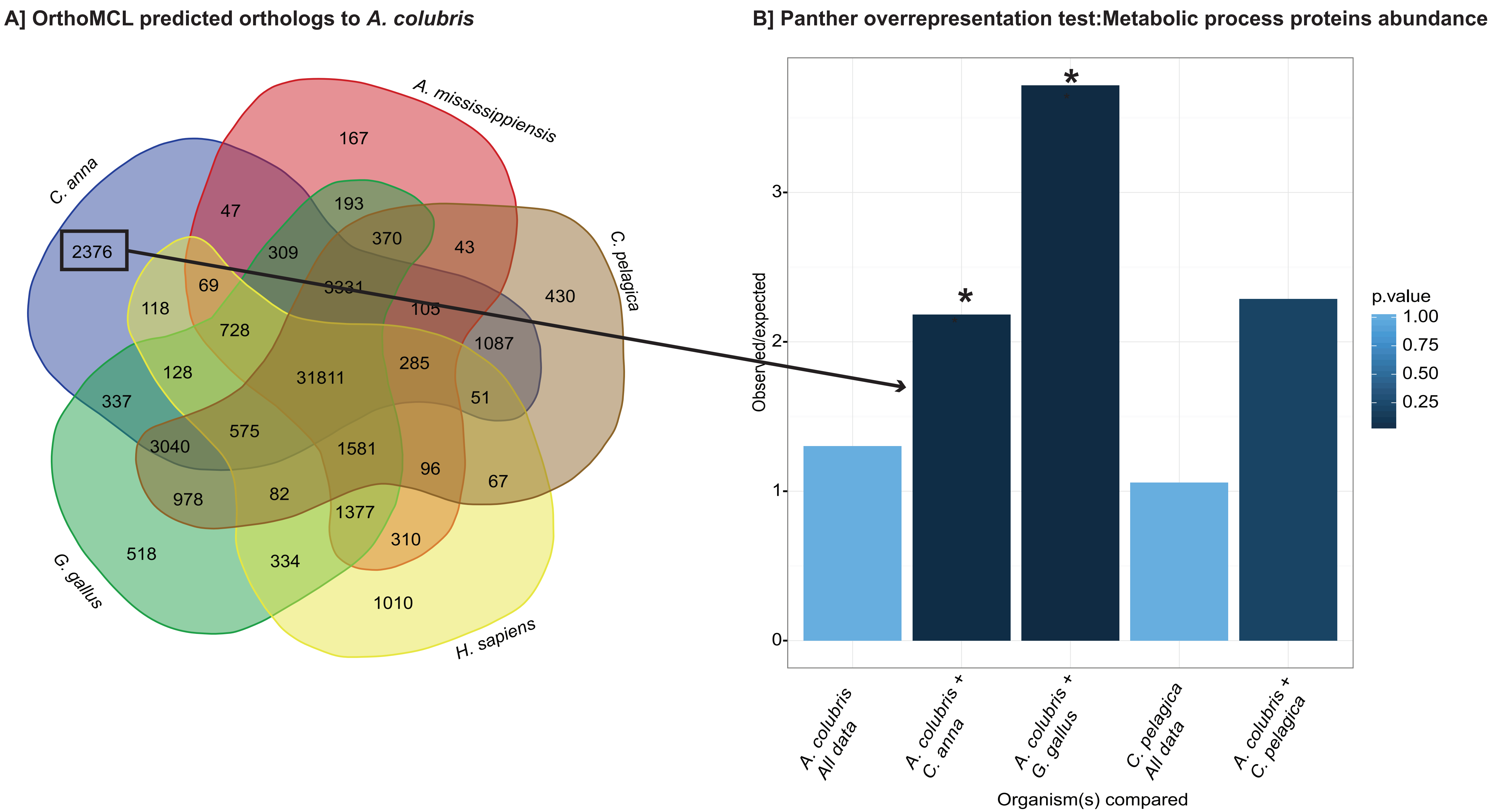
A] OrthoMCL predicted orthologs to A. colubris
B] Panther overrepresentation test:Metabolic process proteins abundance
C. anna
A. mississippiensis
C. pelagica
G. gallus
H. sapiens
167
47
193
370
43
2376
309
69
3331
430
118
105
728
1087
128
31811
285
337
51
575
3040
1581
96
67
978
82
1377
310
518
334
1010
Observed/expected
p.value
1.00
0.75
0.50
0.25
0
1
2
3
A. colubris All data
A. colubris + C. anna
A. colubris + G. gallus
C. pelagica All data
A. colubris + C. pelagica
Organism(s) compared

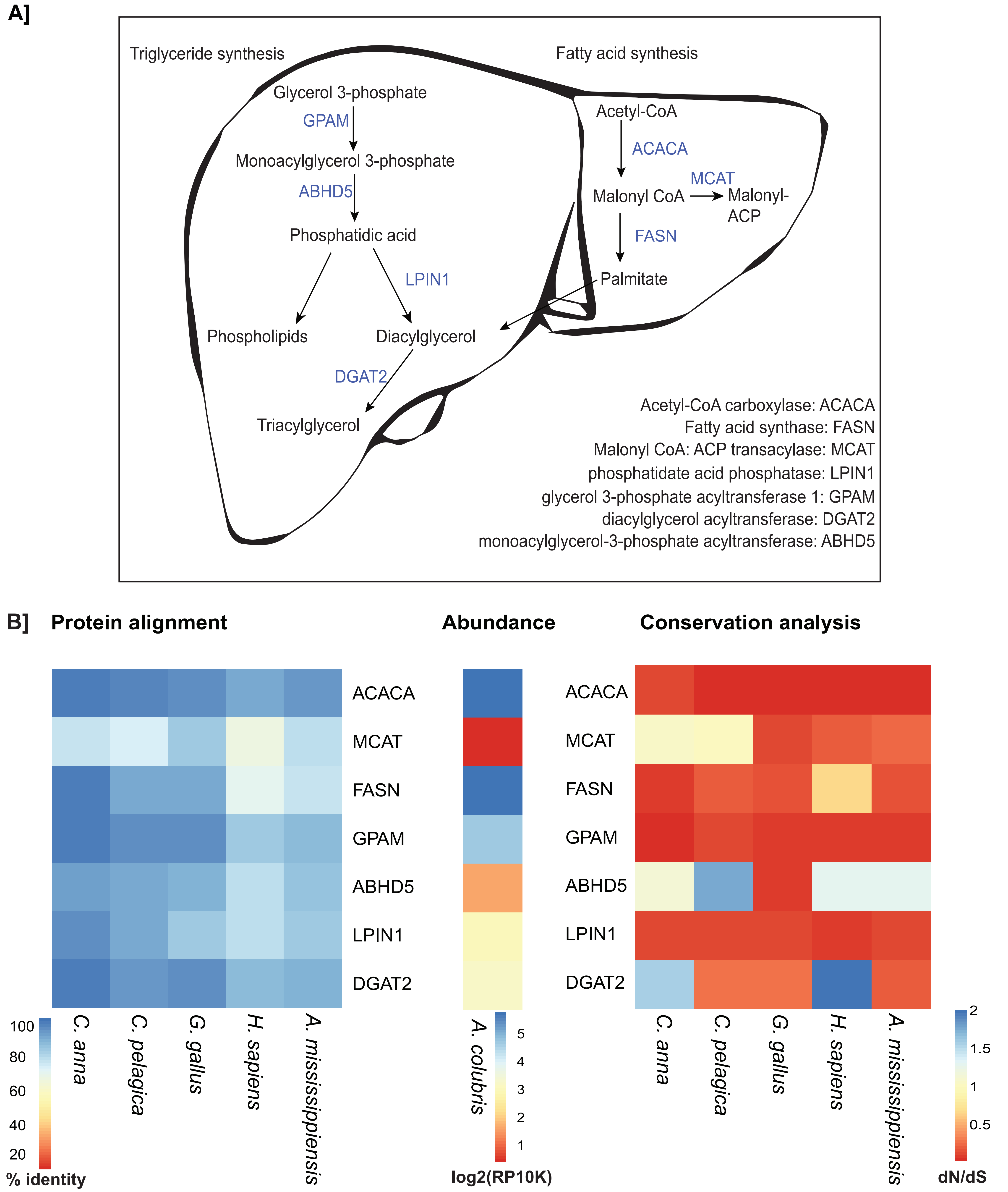
A]
Triglyceride synthesis
Fatty acid synthesis
Glycerol 3-phosphate
GPAM
Monoacylglycerol 3-phosphate
ABHD5
Phosphatidic acid
LPIN1
Phospholipids
Diacylglycerol
DGAT2
Triacylglycerol
Acetyl-CoA
ACACA
Malonyl CoA
MCAT
Malonyl-ACP
FASN
Palmitate
Acetyl-CoA carboxylase: ACACA
Fatty acid synthase: FASN
Malonyl CoA: ACP transacylase: MCAT
phosphatidate acid phosphatase: LPIN1
glycerol 3-phosphate acyltransferase 1: GPAM
diacylglycerol acyltransferase: DGAT2
monoacylglycerol-3-phosphate acyltransferase: ABHD5
B]
Protein alignment
Abundance
Conservation analysis
ACACA
MCAT
FASN
GPAM
ABHD5
LPIN1
DGAT2
C. anna
C. pelagica
G. gallus
H. sapiens
A. mississippiensis
A. colubris
100
80
60
40
20
% identity
5
4
3
2
1
log2(RP10K)
2
1.5
1
0.5
dN/dS

Supplementary Methods

Click here to access/download
**Supplementary Material**
Supplemental methods.pdf

Supplementary Data

Click here to access/download
**Supplementary Material**
171009_suppdata.pdf

JOHNS HOPKINS
UNIVERSITY

Department of Biomedical Engineering
Clark Hall 118A
3400 N. Charles St.
Baltimore MD 21218
wtimp@jhu.edu
www.timplab.com

Winston Timp
Assistant Professor

10/9/2017

Dear Dr. Zauner:

Below please find our revisions to the manuscript entitled "Single molecule, full-length transcript sequencing provides insight into the extreme metabolism of ruby-throated hummingbird *Archilochus colubris*".

We thank the editors and reviewers for their thoughtful comments on the initial submission. We have addressed all of the reviewers' concerns and are submitting what we believe to be an improved version of our manuscript. The revised manuscript includes careful reanalysis as well as a new Illumina RNA-seq data set for quantification and correction of errors in the PacBio data.

Please feel free to contact us with any further concerns or issues. Thank you for your kind consideration of the manuscript.

Sincerely,

Winston Timp
Assistant Professor
Department of Biomedical Engineering
Johns Hopkins University
