# Supplementary material for: Single-molecule, full-length transcript sequencing provides insight into the extreme metabolism of the ruby-throated hummingbird Archilochus colubris
Source: Gigascience. 2018 Feb 15;7(3):giy009. doi: 10.1093/gigascience/giy009 (PMC5869288; doi:10.1093/gigascience/giy009)
Supplement: GIGA-D-17-00088_Revision_3.pdf [file giy009_giga-d-17-00088_revision_3.pdf]

GigaScience

Single molecule, full-length transcript sequencing provides insight into the extreme metabolism of ruby-throated hummingbird Archilochus colubris

--Manuscript Draft--

**Manuscript Number:**	GIGA-D-17-00088R3
**Full Title:**	Single molecule, full-length transcript sequencing provides insight into the extreme metabolism of ruby-throated hummingbird Archilochus colubris
**Article Type:**	Data Note
**Funding Information:**	Human Frontier Science Program (#RGP0062/2016) — Dr. Kenneth C. Welch<br>Natural Sciences and Engineering Research Council of Canada (CA) (#386466) — Dr. Kenneth C. Welch
**Abstract:**	Background<br>Hummingbirds oxidize ingested nectar sugars directly to fuel foraging but cannot sustain this fuel use during fasting periods, such as during the night or during long-distance migratory flights. Instead, fasting hummingbirds switch to oxidizing stored lipids, derived from ingested sugars. The hummingbird liver plays a key role in moderating energy homeostasis and this remarkable capacity for fuel switching. Additionally, liver is the principle location of de novo lipogenesis, which can occur at exceptionally high rates, such as during premigratory fattening. Yet understanding how this tissue and whole organism moderates energy turnover is hampered by a lack of information regarding how relevant enzymes differ in sequence, expression, and regulation.<br>Findings<br>We generated a de novo transcriptome of the hummingbird liver using PacBio full-length cDNA sequencing (Iso-Seq), yielding a total of 8.6Gb of sequencing data, or 2.6M reads from 4 different size fractions. We analyzed data using the SMRTAnalysis v3.1 Iso-Seq pipeline, then clustered isoforms into gene families to generate de novo gene contigs using Cogent. We performed orthology analysis to identify closely related sequences between our transcriptome and other avian and human gene sets. Finally, we closely examined homology of critical lipid metabolism genes between our transcriptome data and avian and human genomes.<br>Conclusions<br>We confirmed high levels of sequence divergence within hummingbird lipogenic enzymes, suggesting a high probability of adaptive divergent function in the hepatic lipogenic pathways. Our results leverage cutting-edge technology and a novel bioinformatics pipeline to provide a first direct look at the transcriptome of this incredible organism.
**Corresponding Author:**	Winston Timp<br>Johns Hopkins University<br>Baltimore, Maryland UNITED STATES
**Corresponding Author Secondary Information:**	
**Corresponding Author's Institution:**	Johns Hopkins University
**Corresponding Author's Secondary Institution:**	
**First Author:**	Rachael E. Workman
**First Author Secondary Information:**	
**Order of Authors:**	Rachael E. Workman<br>Alexander M. Myrka<br>Elizabeth Tseng<br>G. William Wong

Powered by Editorial Manager® and ProduXion Manager® from Aries Systems Corporation

	Kenneth C. Welch
	Winston Timp
**Order of Authors Secondary Information:**	
**Response to Reviewers:**	As per editorial instructions, we have:<br><br>1) Included citations to the GigaDB and ZENODO datasets wehre appropriate<br>2) Removed the sentence "All other data available upon request"<br>3) Added the MIT license to our github repo<br>4) Added a statement to the manuscript involving the approval for research with animals (Candian Wildlife Service permit and UToronto AUP).<br><br>2/5/18<br>5) Fixed the references 28, 29 and 38
**Additional Information:**	
**Question**	**Response**
Are you submitting this manuscript to a special series or article collection?	No
**Experimental design and statistics**<br><br>Full details of the experimental design and statistical methods used should be given in the Methods section, as detailed in our Minimum Standards Reporting Checklist. Information essential to interpreting the data presented should be made available in the figure legends.<br><br>Have you included all the information requested in your manuscript?	Yes
**Resources**<br><br>A description of all resources used, including antibodies, cell lines, animals and software tools, with enough information to allow them to be uniquely identified, should be included in the Methods section. Authors are strongly encouraged to cite Research Resource Identifiers (RRIDs) for antibodies, model organisms and tools, where possible.<br><br>Have you included the information requested as detailed in our Minimum Standards Reporting Checklist?	Yes
**Availability of data and materials**<br><br>All datasets and code on which the conclusions of the paper rely must be either included in your submission or deposited in publicly available repositories	Yes

(where available and ethically appropriate), referencing such data using a unique identifier in the references and in the “Availability of Data and Materials” section of your manuscript.<br><br>Have you have met the above requirement as detailed in our Minimum Standards Reporting Checklist?	

Powered by Editorial Manager® and ProduXion Manager® from Aries Systems Corporation

Manuscript

Click here to download Manuscript 180206_MS_isoseq_hummingbird_7-2-18.docx

Single molecule, full-length transcript sequencing provides insight into the extreme metabolism of ruby-throated hummingbird *Archilochus colubris*

Rachael E. Workman[1*], Alexander M. Myrka[2*], Elizabeth Tseng[4], G. William Wong[3], Kenneth C. Welch Jr.[2+], and Winston Timp[1+]

[1] Department of Biomedical Engineering, Johns Hopkins University, Baltimore, MD, USA
[2] Department of Biological Sciences, University of Toronto Scarborough, Toronto, Ontario, Canada and Department of Cell & Systems Biology, University of Toronto, Toronto, Ontario, Canada
[3] Department of Physiology and Center for Metabolism and Obesity Research, Johns Hopkins University School of Medicine, Baltimore, MD, USA
[4] Pacific Biosciences, Menlo Park, California, USA
* Co-first author
+ Co-Corresponding author

Abstract

Background

Hummingbirds oxidize ingested nectar sugars directly to fuel foraging but cannot sustain this fuel use during fasting periods, such as during the night or during long-distance migratory flights. Instead, fasting hummingbirds switch to oxidizing stored lipids, derived from ingested sugars. The hummingbird liver plays a key role in moderating energy homeostasis and this remarkable capacity for fuel switching. Additionally, liver is the principle location of *de novo* lipogenesis, which can occur at exceptionally high rates, such as during premigratory fattening. Yet understanding how this tissue and whole organism moderates energy turnover is hampered by a lack of information regarding how relevant enzymes differ in sequence, expression, and regulation.

Findings

We generated a *de novo* transcriptome of the hummingbird liver using PacBio full-length cDNA sequencing (Iso-Seq), yielding a total of 8.6Gb of sequencing data, or 2.6M reads from 4 different size fractions. We analyzed data using the SMRTAnalysis v3.1 Iso-Seq pipeline, then clustered isoforms into gene families to generate *de novo* gene contigs using Cogent. We performed orthology analysis to identify closely related sequences between our transcriptome and other avian and human gene sets. Finally, we closely examined homology of critical lipid metabolism genes between our transcriptome data and avian and human genomes.

Conclusions

We confirmed high levels of sequence divergence within hummingbird lipogenic enzymes, suggesting a high probability of adaptive divergent function in the hepatic lipogenic pathways. Our results leverage cutting-edge technology and a novel bioinformatics pipeline to provide a first direct look at the transcriptome of this incredible organism.

Keywords

Pacbio; single molecule sequencing; Iso-seq; transcriptome; liver; metabolism; hummingbirds

Data Description

Background

Hummingbirds are the only avian group to engage in sustained hovering flight as a means for accessing floral nectar, their primary caloric energy source. While hovering, small hummingbirds, such as the ruby-throated hummingbird (*Archilochus colubris*), achieve some of the highest mass-specific metabolic rates observed among vertebrates [1,2]. Given their specialized, sugar-rich diet, it is not that surprising that hummingbirds are able to fuel this intense form of exercise exclusively by oxidizing carbohydrates [3,4]. This energetic feat is also remarkable in that the source of sugar oxidized by flight muscles during hovering is the same

sugar ingested in nectar meals only minutes prior [4,5]. In addition, hummingbirds seem equally adept at relying on either glucose or fructose (the two monosaccharides comprising their nectar) [6] as a metabolic fuel for flight [4]. In doing so, they achieve rates of sugar flux through their bodies that are up to 55× greater than non-flying mammals [7].

Hummingbird flight is not always a solely carbohydrate-fueled endeavor. Lipids are a more energy dense form of fuel storage, and fasted hummingbirds are as capable of fueling hovering flight via the oxidation of onboard lipid stores as they are dietary sugars [5]. Lipids are likely the sole or predominant fuel used during overnight periods [8]. Just as flux of sugar through the hummingbird is extremely rapid, the building of lipid stores from dietary sugar is also rapid when needed. For example, ruby-throated hummingbirds can routinely increase their mass by 15% or more between midday and dusk on a given day [9]. The ruby-throated hummingbird (*A. colubris*) completes an arduous annual migratory journey from breeding grounds as far north as Quebec in Canada to wintering grounds in Central America [10]. Hummingbirds are constrained to fueling long distance migratory flights using onboard lipids. In preparing for such flights, some individuals rapidly build fat stores prior to departure or at migratory stopover points, increasing their mass by 25-40% in as few as four days [9,11,12].

The ability to switch so completely and quickly between fuel types means these animals possess exquisite control over rates of substrate metabolism and biosynthesis in the liver, the principal site of lipogenesis in birds [13]. While hummingbird liver does indeed exhibit remarkably high activities of lipogenic and other metabolic enzymes [14], the mechanisms underlying high catalytic rates (high catalytic efficiency and/or high levels of enzyme expression) and control over flux (the role of hierarchical versus metabolic control), remain unclear.

Despite long-standing recognition of, and interest in, their extreme metabolism, the lack of knowledge about gene and protein sequences in hummingbirds has limited more detailed and mechanistic analyses. Amplification of hummingbird genetic sequences for sequencing and/or cloning is hampered by the lack of sequence information from closely related groups, making well-targeted primer design difficult. Only two genes have thus far been cloned from any hummingbird: an uncoupling protein (UCP) homolog and insulin [15,16]. These two studies offer limited insight into what adaptations in hepatic molecular physiology underlie extreme energy turnover or its regulation. The UCP homolog was cloned from pectoralis (flight muscle) and its functional significance *in vivo* is unclear. The amino acid sequence of hummingbird insulin was found to be largely identical to that from chicken; however, birds are insulin insensitive and lack the insulin-regulated glucose transporter (GLUT) protein GLUT4, making the role of this hormone in the regulation of energy homeostasis in hummingbirds unknown [17–19].

Recently completed sequencing of the Anna’s hummingbird (*Calypte anna*) genome provides a powerful new tool in the arsenal of biologists seeking to understand variation in metabolic physiology in hummingbirds and other groups [20]. Despite their extreme catabolic and anabolic capabilities, hummingbirds have the smallest genomes among birds [21] and, in general, have among the smallest vertebrate genomes [22]. Thus, it seems likely that understanding of transcriptional variation, overlaid on top of genetic variation, is crucial to understanding what makes these organisms such elite metabolic performers.

To this end, we produced the liver transcriptome of the ruby-throated hummingbird, *Archilochus colubris*. Because many of the proteins involved in cellular metabolism are quite large, we collaborated with Pacific Biosciences to generate long-read sequences as these would enhance our ability to identify full coding sequences and multiple encoded isoforms. The primary advantage to the PacBio Iso-seq methodology is the capability for full-length transcript sequencing, rendering complete mRNA sequences without the need for assembly. This has been demonstrated in previous studies to dramatically increase detection of alternative splicing events [23]. Additionally, full-length sequences greatly enhance the likelihood of detecting novel or rare splice variants, which is crucial for fully characterizing the transcriptomes of lesser studied, non-model organisms such as the hummingbird.

Methods

Sacrifice and sample preparation

A wild adult male ruby-throated hummingbird (*Archilochus colubris*) was captured at the University of Toronto Scarborough using modified box traps on July 23rd 2013 at 8:15AM. At the time of its capture, the bird was aged as an "after hatch year" bird, meaning it was at least 1 year old. Standard aging techniques make more precise aging of hummingbirds more than 1 year old difficult [24]. The bird was housed in the University of Toronto Scarborough vivarium and fed NEKTON-Nectar-Plus (Nekton, Tarpon Springs, FL, USA) *ad libitum*, and sacrificed after *ad libitum* feeding at 1:22PM on July 16th 2014 (being 2+ years old). On arrival it weighed 2.68g and at the time of sacrifice it weighed 3.11g. Tissues were sampled immediately after euthanization using RNAse-free tools. Liver tissue was dissected out and homogenized at 4ºC in 1 mL cold Tri Reagent using an RNase free glass tissue homogenizer and RNase free syringes of increasing needle gauge. We used 100 mg of tissue per 1 mL of Tri Reagent (Sigma-Aldrich, St. Louis, MO, USA), and chloroform extraction was performed twice to ensure quality. RNA was precipitated with isopropanol, centrifuged at 12000xg for 10 minutes, washed with ethanol 2x, vacuum dried at room temperature and eluted in RNAse free water [25]. DNAse I (Life Technologies) digestion and spin column cleanup were performed (Ambion Purelink RNA mini kit, Life Tech). RNA concentration and RIN were determined with RNA Bioanalyzer (Agilent). The sample used for Illumina sequencing was harvested using the same methods, but from a different animal. The bird was captured as described above on August 22nd, 2011 at 10:50AM. At the time of capture, the bird was aged as "hatch year" and it weighed 2.93g. It was housed and sacrificed as described above on January 25th, 2016 at 10:50AM (being over 4 years old). Sampled individuals were captured under the provisions of a Canadian Wildlife Service permit (# CA 0258) and all procedures were performed under the auspices of a University of Toronto Animal Use Protocol (# 20011649).

Sequencing library preparation

Pacific Biosciences's Iso-Seq sequencing protocol was followed to generate sequencing libraries [26]. Briefly, Clontech SMARTER cDNA synthesis kit with Oligo-dT primers was used to generate first and second-strand cDNA from polyA mRNA. After a round of PCR amplification, the amplified cDNA was size selected into 4 size fractions (1-2kb, 2-3kb, 3-6kb, and 5-10kb) to prevent preferential small template sequencing, using the BluePippin (0.75% agarose external

marker, Sage Sciences). Additional PCR cycles were used post size-selection to generate adequate starting material, and then SMRTbell hairpin adapters were ligated onto size-selected templates. Each of the 4 size fractions was sequenced on 10 SMRT Cells, for a total of 40 SMRT Cells. Sequencing was performed by the JHU HiT Center using P6-C4 chemistry on the RSII sequencer. Illumina sequencing libraries were generated using Lexogen mRNA sense v2 Illumina library preparation kit, and sequenced on a single rapid-run lane of Hiseq 4000 2x100bp paired end, yielded 153M reads.

Analysis Methods

Data processing, isoform clustering sorting and quality control of liver transcriptome

We performed initial data processing using SMRTanalysis 3.1 Iso-Seq pipeline employed using a DNANexus interface. From 40 SMRTcells, we produced 440.75 Gb of raw data, which was classified into 3.4 Gb of non-chimeric circular consensus (CCS) reads. CCS reads comprised 1.23M full length, 1.27M non-full-length reads; reads were considered full-length if both 5' and 3' cDNA primers as well as the polyA tail signal were detected. Of the four size-selected bins, our average CCS length was 1533, 2464, 3650, and 5444 bp, respectively (Figure 1B). The Iso-Seq pipeline then performed isoform-level clustering (ICE) followed by final polishing using Arrow [27] to output high-quality (predicted accuracy >= 99%), full-length, isoform consensus sequences. The Iso-Seq pipeline produced 238Mb of high quality consensus isoforms (HQD, 94,724 reads), and 2Gb (712,210 reads) of low quality consensus isoforms (summary statistics Figure 1A). BLAST searches were then performed to remove putative contaminants, and coding sequence and protein translation was performed, resulting in 93K HQ and 680K LQ protein sequences. A summary of the analyses performed are displayed in Figure 2A-B, further details and settings can be found in Supplemental Methods, and data can be found in our GigaScience and Zenodo Databases [28,29].

Assessing transcriptome completion

To estimate the completeness of our liver transcriptome sequencing, we used both subsampling and gene diversity estimation, as well as BUSCO (BUSCO, RRID:SCR_015008) [30], [31]. BUSCO checks for essential single copy orthologs which should be present in a whole transcriptome dataset for any member of the given lineage. We used both Metazoan and Aves lineages (ortholog sets) to examine transcriptome completion (Figure 2C and Supplemental Table 1), and to ensure that completeness tracked across multiple data processing steps, we analyzed ASD (all sequence data), HQD (high quality data) and CCD (Cogent collapsed data). As expected, *Gallus gallus* and *Calypte anna* genome predicted transcriptomes were nearly complete for both Aves and Metazoan BUSCO sets, and our *A. colubris* transcriptome only captured around half of this diversity, likely due to our sample being a single tissue, collection time point and individual.

Our subsampling approach to estimating transcriptome completeness involved pulling subsets of the circular consensus reads dataset and BLASTing against the predicted *Calypte anna* gene set. We found that the number of unique genes detected began to saturate when reaching a 90% subset of our data, suggesting that additional sequencing would not substantially

contribute to transcriptome completion (Supplemental Figure 1). Lower expressed genes may not be detected, but that vast majority of annotated liver expressed genes are likely represented in our data.

Agreement with established Anna's hummingbird genomes reveals general clade conservation

We aligned transcripts to the *Calypte anna* (Anna's hummingbird) genome using GMAP (GMAP, RRID:SCR_008992) [32]. In order to validate transcript coverage and alignment throughout the multiple processing steps, we aligned using not only high quality isoforms (HQD), but also the full consensus isoform dataset (ASD) and gene families predicted by Cogent (CCD, methods in Supplemental methods and below).

Calypte anna and *Archilochus colubris* are close relatives within the North American Bee (Mellisugini) clade of hummingbirds [33]; *A. colubris* is a member of the Caribbean Sheartails subclade and *C. anna* is of the Calypte subclade, which diverged from the from ancestral Mellisugini around early to mid Pliocene [34]. Given this fairly recent divergence, we expected alignment to perform well. We found an average alignment identity of 94.8%, with 87% transcripts uniquely mapping to the reference. Of the uniquely mapped, 73% covered >90% of the query sequence (alignment length and statistics, Supplemental Figure 2A, 2B), demonstrating high fidelity of aligned reads to reference. When ASD reads were parsed by number of reads of insert supporting each consensus cluster, it was found that generally, alignment identity was high regardless of number of supporting reads. A clear increase in mean alignment identity was found when two or more supporting reads were collapsed (Supplemental Figure 3).

When GMAP was performed using only high quality isoforms (filtered for 2+ full-length supporting reads), alignment percentage was 95.7%, with 93.4% of transcripts mapping uniquely to the reference. The average mapped read length was 2411bp (HQD, 2617bp ASD), while the average predicted CDS length for *Calypte anna* was 1386bp. This being said, reads mapped with GMAP contain UTRs. When we predict just the CDS sequences for *A. colubris* using ANGEL[35], the mean length was 981bp. When we BLASTed the unaligned reads to whole NCBI database, they largely mapped back to *Calypte anna* (53%). This result suggests that our mapping parameters were too stringent to map these reads, error rate prevented alignment, unaligned regions are divergent enough between both hummingbirds to preclude alignment, or a combination of the above.

Putative gene family prediction and reduction of transcript redundancy reduces data load while maintaining transcript diversity

To assign transcripts to putative gene families, as well as cluster and eliminate redundant transcripts to produce a unique set of gene isoforms, we utilized the newly developed Cogent [36] pipeline. Cogent is specifically designed for transcriptome assembly in the absence of a reference genome, allowing for isoforms of the same gene to be distinctly identified from different gene families, which are defined as having more than two (possibly redundant) transcript copies. Of the 94,724 HQ consensus isoforms, 91,733 were grouped into 6,725 multi-transcript gene families (Figure 3A). The remaining 2,991 sequences were classified as putative

single-isoform genes, or “orphans”. Reconstructed contigs were then applied in place of a reference (or *de novo* clustering) to reduce redundant transcripts in the original HQD dataset. From this approach, we were able to reduce our HQ dataset to 14,628 distinct transcript isoforms and 2990 orphan isoforms, for a total of 17,618 isoform sequences (18% of the original). Due to the use of HQD only transcripts (2 full length reads and estimated accuracy >99%), and constraints of transcript collapse, a number of additional isoforms were likely lost in filtering and collapse, reducing transcript diversity. However, without sufficient supporting data the trade-off between between gene diversity and reliability led us to choose reliability. Future studies should examine whether transcript “rescue” from low quality datasets is possible with Illumina validation or additional consensus generation strategies.

Cogent collapsed data is further summarized and most abundant transcripts are detailed in Supplemental Table 2. An average of 1.53 isoforms was found per gene family (Figure 3B), with 2624, or 27.4% of the gene families having more than one isoform, including “orphans”. While other studies have found more isoforms per locus, for example 6.56 in *Zea mays* [37], that study multiplexed six plant tissues, whereas a lower complexity is to be expected with single tissue analysis. This dataset (Cogent collapsed data, or CCD) was also mapped onto the *Calypte anna* genome assembly [38], to demonstrate the effectiveness of this method in reducing transcript redundancy and classifying isoforms (Figure 3C). Cogent gene families were polished using Illumina short read RNAseq data and the error correction algorithm Pilon [39] (Supplemental Methods) to obtain higher accuracy reads.

Orthologous gene pair predictions and GO annotation show putative unique hummingbird orthologs

To examine protein sequence similarity and divergence between *Archilochus colubris* and other avian species, we used OrthoMCL (OrthoMCL DB: Ortholog Groups of Protein Sequences, RRID:SCR_007839), which generates reciprocal best hits from comparison species using BLAST all-vs-all, then clustering to group orthologous sequences for each pair of organisms [40]. OrthoMCL protein sequences were predicted using ANGEL[35], and 119,292 high quality sequences were put into this analysis. We compared our ruby-throated hummingbird, *Archilochus colubris,* to five other birds: *Calypte anna* (Anna’s hummingbird) fellow member of the bee clade of hummingbirds, *Chaetura pelagica* (chimney swift) the closest available outgroup species to the hummingbird clade, and other bird species for which relatively well-annotated genomes and/or transcriptomes are available, *Gallus gallus* (chicken), *Taeniopygia guttata* (zebra finch), and *Melopsittacus undulatus* (budgerigar), as well as *Homo sapiens* (human), and *Alligator mississippiensis* (American alligator). Algorithm parameters and data accession numbers are presented in Supplemental methods.

A matrix of ortholog pairings, with duplicate ortholog hits removed, shows the number of orthologous sequences for each species pair (Supplemental Table 3). Orthologs shared between ruby-throated hummingbird and a subset of the other species analyzed are illustrated in Figure 4A. Unsurprisingly, the largest amount of orthologs which pair closely to only one species, i.e., 1:1 orthologs, were found between Anna’s and Ruby-throated hummingbird. Surprisingly, the second-largest set was between chicken and ruby-throated hummingbird, as

opposed to its closest outgroup species, *Chaetura pelagica*. This is likely due to the completeness of chicken transcriptome annotation, as chicken is the most well-studied avian species. Of the 596 unpaired *A. colubris* protein sequences, 190 paired most closely with *Calypte anna* when compared using BlastP and the majority of matches output (559/594) were less than 50 AA, only a fraction of the average sequence length.

In order to more closely examine the identity of orthologs in related hummingbird species, gene ontology (GO) annotation was performed on the set of orthologs which were shared between *Calypte anna* and *Archilochus colubris*, but not by the other birds included in the OrthoMCL analysis. This set of 2,376 protein sequences was examined using BlastP and GO analysis performed by Panther [41,42]. Additional datasets used for GO comparison included 1:1 orthologs for *Gallus gallus* and *A. colubris* (518), and *A. colubris* and *Chaetura pelagica* (430), as well as whole transcriptome data from *C. pelagica* and the Cogent-collapsed dataset from our transcriptome (Supplemental Table 4, Figure 4B).

As the initial impetus for our investigation centered on the exceptional metabolism and energetics of hummingbirds, we focused our investigation on orthologs tagged as part of the "metabolic process (GO:0008152)" grouping. Of the 1444 orthologs identified in *Archilochus colubris* as part of this process grouping, 236 (16.3%) were unique to hummingbirds. Within this top-level grouping, the largest number of genes group under "primary metabolic processes" (GO:0044238)". Of the 1240 orthologs identified within this grouping, 204 (16.3%) are identified as uniquely shared by our hummingbird species. Six GO biological processes are defined under the "primary metabolic processes". Of these processes, the process with the highest proportion of identified *A. colubris* orthologs hitting as unique to the two hummingbird species is "lipid metabolic processes" (GO:0006629; 33 of 114 orthologs, 28.9%), which is significantly enriched relative to the comparative orthology databases of both chicken and human (Statistical overrepresentation test, Panther, [41], p-values given in Supplemental table 4). Because we considered it likely that an enrichment in lipid metabolic genes could be a result of our dataset being from liver tissue, we compared enrichment with that of the entire Cogent predicted gene set from the ruby-throated hummingbird transcriptome, and found no significant enrichment using the same tests (Supplemental table 4). Because 1:1 hummingbird orthologs are relatively more abundant in lipid metabolic genes than the sequences which were found to be highly homologous to one or more of the other species compared using OrthoMCL, we predict that lipid metabolic genes are more divergent from the other examined species than other classes of enzymes. Though this alone is not direct evidence of greater selection on proteins within that pathway, it is suggestive. If neutral sequence divergence is assumed to be randomly accrued throughout a species' genome, then greater divergence in enzymes making up "lipid metabolic processes" suggests that closer examination of these proteins for evidence of functional, or even adaptive, divergence is warranted. A phylogenetically-informed analysis of ortholog divergence among taxa is necessary to establish a selection signature, which will become possible in the future with the advance of the B10K project [43] and larger numbers of avian species in GO databases.

Given the apparent sequence divergence among enzymes involved in "lipid metabolic processes" hinted at by orthology and ontology analyses, we elected to more closely examine

enzymes comprising the lipogenic pathway. In liver, fatty acids can be synthesized via the *de novo* lipogenesis pathway using acetyl CoA as substrate. These newly synthesized fatty acids can then be esterified onto the glycerophosphate backbone to generate triglycerides via the glycerol-3-phosphate pathway of lipid synthesis. We predicted that key enzymes involved in these two pathways (Figure 5A) would be divergent in hummingbirds given their extraordinary metabolic demands. Eight enzymes involved in this pathway were examined for *Archilochus colubris, Calypte anna, Gallus Gallus, Chaetura pelagica*, *Alligator mississippiensis* and *Homo sapiens (*accession numbers and details given in Supplemental Table 5). Pairwise protein alignment scores are given in Supplemental Table 6 as well as illustrated in a heatmap shown in Figure 5B, and alignments in Supplemental Data 1. Interestingly, enzymes involved in *de novo* fatty acid synthesis share a higher degree of identity between examined organisms, whereas enzymes involved in triglyceride synthesis tend to be slightly less conserved (Figure 5A). Figure 5B also shows normalized abundances of the enzymes of interest in our liver transcriptome dataset, revealing a high expression level of the rate-setting enzyme involved in *de novo* lipogenesis (*ACACA*; acetyl CoA carboxylase). In contrast to the cytosolic *ACACA* enzyme that uses acetyl-CoA as substrates for fatty acid synthesis, *MCAT* encodes a mitochondrial enzyme that uses malonyl-CoA as substrates for fatty acid synthesis. Much less is known about the MCAT-dependent pathway of fatty acid synthesis in mitochondria. Interestingly, MCAT has the lowest relative abundance in ruby throated hummingbird liver. The relative hepatic expression levels of triglyceride synthesis genes (e.g., *LPIN1* and *DGAT2*) are also much lower compared to genes involved in *de novo* lipogenesis (*ACACA* and *FASN*). It is important to note that most metabolic enzymes are tightly regulated. The relative levels of hepatic lipogenesis enzymes may vary greatly depending on the time of day and the physiological states (fast vs. fed) of the animals.

In order to further investigate degree of conservation between key hepatic lipogenesis enzymes in hummingbirds and comparative organisms, we performed conservation analysis and determined ratio of nonsynonymous to synonymous codon changes (dN/dS) as a metric of positive selection, using pairwise alignments followed by the CodeML module in PAML4[44]. These ratios are given in Supplemental Table 6 and plotted in a heatmap in Figure 5B. A dN/dS score > 1 denotes genomic regions putatively undergoing positive selection. We found, in general, good conservation of these enzymes among species, with the exception of the 3' and 5' ends of alignments. These often had an extended or retracted coding sequence in the case of hummingbirds and *C. pelagica*, which could be related to post-translational modification or selection on pathway regulation [45]. Surprisingly, terminal sequence length was variable even between *C. anna* and *A. colubris,* which both belong to the closely-related Bee hummingbird taxon [33]. Variation in 5' and 3' length may also be an effect of the different methodologies used to produce these sequences, RNA sequencing for *A. colubris, G. gallus,* and *H. sapiens*, and ORF prediction from genomic data for the other organisms examined. For example, we note in our analysis that *MCAT* appears more conserved between *A. colubris* and *H. sapiens*, than between *A. colubris* and *C. anna*, which could be due not to *A. colubris* actually being more similar to *H. sapiens*, but rather to ORF prediction oversights.

The averaged dN/dS values, while useful for comparison, can be misleading when considered over the entire gene, as 3' and 5' variation can overshadow conserved motifs, and pairwise

comparisons (Supplemental Data 1 and 2) are limited in scope. This type of analysis is ideal for very divergent sequences, but less informative for pairs of sequences that are highly similar [46]. Despite this, conservation analysis is still valuable and provides insights connecting nucleotide to amino acid information that alignments alone can miss. For example, lysophosphatidic acid acyltransferase (*ABHD5*), which functions primarily in phosphatidic acid biosynthesis, has reasonable protein alignment scores to all comparative organisms but also shows positive selection acting upon this gene relative to *Calypte anna*, swift, human and alligator, but not chicken (Figure 5B). This led us to more closely examine the coding sequence alignment, where we found that the bulk of differences in coding sequence were attributable to exon 1, with alignment largely becoming synchronous (with the exception of *H. sapiens*, which is widely divergent) by exon 2 and continuing through to the end of the transcript. Although the primary AB hydrolase-1 domain is very well conserved between species, these differences in exon 1 could be functionally significant, and honing down to regions of differentiation between comparative species gives us interesting starting points for future investigations, including the cloning and enzyme kinetics studies of *ABHD5*. Additionally, pairwise comparisons provide interesting observations, such as coding strand elongation in the 5' region in *A. colubris GPAM* (Supplemental Data 2). This information can be leveraged for future studies examining enzyme structure, function and evolution.

Transcriptome resource mining could provide functional genomic insights

Access to the transcriptome informs the investigation of biological processes and enables the formation of new hypotheses. This is exemplified by the serendipitous observation that hummingbird glucose transporter 2 (*GLUT2*) lacks a N-glycosylation site due to an asparagine to aspartic acid amino acid substitution. This missing glycosylation site was also seen in the available Anna's hummingbird genome. All class 1 glucose transporters studied in model vertebrates contain one N-glycosylation site located on the large extracellular loop of the protein [47]. In GLUT2 the associated glycan interacts with the glycan-galectin lattice of the cell, stabilizing cell surface expression [48]. Removal of the N-glycan of GLUT2 in rat pancreatic β cells results in the sequestering of cell-surface GLUT2 in lipid rafts and this sequestered GLUT2 exhibits a reduction in glucose transport activity by approximately 25% [48]. This reduction in transport is thought to occur through interaction of the *GLUT* with lipid raft-bound stomatin [48,49]. In mammals, GLUT2 serves a glucose-sensing role in the pancreatic β cells and is required for the regulation of blood glucose through insulin and glucagon [50]. The lack of N-glycosylation of GLUT2 may contribute to observed high blood glucose concentration in hummingbirds [51].

Another serendipitous observation was the highly abundant chitinase-like transcript noted from Illumina sequencing results. While humans express chitinase in the gut, but not the liver, chickens express the enzyme in both gut and liver, and other mammals (cows) express the enzyme only in the liver [52]. Suzuki et al. hypothesize that the ancestral state is expression of chitinase in both tissues. While the gut chitinase is used for digestion, expression in liver is believed to contribute to serum chitinase levels and to act as a defense against chitin-containing pathogens[52]; perhaps the second animal had an infection? The chitinase-like isoform in our dataset is highly homologous to the chicken liver chitinase-like transcript.

Re-use potential

In conclusion, our results have leveraged cutting-edge technology to provide a compelling first direct look at the transcriptome of this incredible organism. By using PacBio sequencing, we have been able to generate full length cDNA transcripts from the hummingbird liver. Transcriptome data generated using the Iso-seq methodology, when coupled to recently developed sophisticated gene synthesis techniques [53], will allow simple generation of relevant isoforms for biochemical experiments. Some of the key metabolic enzymes identified from our work as being unique to either *A. colubris* or at most common to *C. anna* and *A. colubris* can now be quickly cloned and expressed. Follow up studies will allow for biochemical studies of proteins generated directly from our transcriptome data, measuring their enzymatic properties, e.g. k_{cat} or V_{max}, as compared to other avian or mammalian analogues [14,54,55]. Expressed proteins may also be used for structural biology studies, applying either x-ray crystallography or cryoEM to generate structural maps of the proteins, then examine how the structure compares to other analogues.

Availability of supporting data

Supporting datasets can be found on GigaDB [28]. Filtered fastq files of clustered CCS reads are deposited under SRA accession number SRP099041. Predicted Cogent gene families, coding sequence and annotations, peptide and untranslated region data are available via the Zenodo data repository [29].

Availability of source code and requirements

Project name: Ruby_isoseq

Project home page: https://github.com/reworkman/hummingbird

Operating system: Unix

Programming language: Bash, Python, R

Other requirements: BUSCO, GMAP, Blast+, ANGEL, CLUSTAL, Cogent, and their dependencies

License: MIT

Acknowledgments

Pacific Biosciences for reagents and SMRTcells as well as technical support. M. Schatz, E. Jarvis, J. Korlach, Y. Guo for discussion. HFSP grant #RGP0062/2016. Natural Sciences and Engineering Research Council of Canada Discovery Grant (#386466) to KCW.

Disclosure Declaration

W.T. and R.W. have received travel funds to speak at symposia organized by Pacific Biosciences. Bulk of reagents for IsoSeq were provided by Pacific Biosciences.

Figure legends

Figure 1. Transcriptome dataset quality control reveals good throughput, read length, and transcriptome completion. Average read lengths and isoform counts for 4 sequenced size fractions given in **A**, and read length distribution for all sequence data (ASD, is all sequence data, high quality (HQ) and low quality (LQ) isoforms) on x vs read counts on y plotted in **B**, with black line representing Mb data greater than read length. For example, at 2000bp, 4000Mb of sequence data was larger than 2000bp. **C**. BUSCO transcriptome assessment results displayed for *Archilochus colubris* (ruby-throated hummingbird, all sequence data ASD, high quality sequence data HQD), Cogent-collapsed data (CCD), *Calypte anna* (Anna's hummingbird), *Gallus gallus* Thomas (chicken single-tissue transcriptome[26]) illustrate transcriptome completion relative to predicted single copy ortholog datasets for both the Class Aves and Kingdom Metazoa.

Figure 2. Analysis pipeline details, as well as amount of data present at each step (in green text). **A** Raw sequence reads from a Pacbio RSII sequencer (bax.h5, bas.h5) were sorted into full and non-full length reads of insert (ROI) using a classification algorithm that identified full length reads with forward and reverse primers, as well as a poly-A tail. Iterative clustering for isoforms (ICE) was performed on full length reads, and non-full length reads were recruited to perform ARROW polished on the consensus isoforms. Polishing sorted reads into high and low-quality bins, and either high quality data (HQD), all sequence data (ASD) or both sets of data, were carried on to further applications (**B**). Applications include ORF and protein sequence generation from high quality (HQD) and low quality (LQD) consensus isoforms, alignment to *C. anna* reference with GMAP of both high quality data (HQD) and Cogent-collapsed data (CCD), detection of orthologous sequences (orth groups) using OrthoMCL, and prediction of gene families (gene fam) using Cogent. Numbers of available reads at each analysis step is displayed in green in each bubble.

Figure 3. Reducing transcript redundancy and predicting gene families using Cogent software. **A.** Gene families predicted and classified by relationship to *Calypte anna* genome assembly shown, along with statistics for alignment using GMAP software which show excellent alignment to closely related hummingbird reference species *Calypte anna*. Cogent comparison cases highlight the relationships between predicted gene families and *C. anna* reference (column captioned "In ref"), and demonstrate the additional information given to an assembly by transcriptome information. Number of isoforms predicted per gene family (unigene) given in **B** shows relatively low isoform diversity in this tissue. **C** Alignment of the MATR3 gene demonstrates the redundancy reducing capabilities of the Cogent software, which was reduced from 11 semi-redundant reads to 3 unique isoforms using this pipeline.

Figure 4. Orthology analysis. The proteomes of five birds (Anna's hummingbird:*Calypte anna*, Zebrafinch: *Tinamus guttatus*, Chicken: *Gallus gallus*, Swift: *Chaetura pelagica*, and Budgeridger:*Melopsitticus undulatus*), one mammal (*Homo sapiens*) and one reptile (*Alligator mississippiensis*) were compared against *Archilochus colubris* using OrthoMCL to detect homologous sequences. A Venn diagram illustrating sequences with best reciprocal blast hits between the given species and *A. colubris* is shown in **A.** Bar chart illustrates

observed/expected ratios of metabolism enzymes (GO group: metabolic process 0008152) for comparison groups (statistical overrepresentation test) for selected OrthoMCL groups using Panther. Datasets input either include the entire proteome of target species (swift all, anna's all) or distinct set of homologs shared two groups (Ex. *A. colubris* + *C. anna* are homologs shared between these two species but not any of the other comparison groups). Asterisks denote significant overrepresentation of metabolic process proteins relative to expected baseline ($p<0.05$)(**B**).

Figure 5. Pathway analysis of key enzymes in hepatic lipogenesis. **A** An overview of the relationship between the investigated genes and their roles in triacylglycerol, phospholipid and fatty acid synthesis, **B** heat maps illustrating percent amino acid identity of these proteins relative to *Archilochus colubris* predicted sequences, abundances (log2(reads per 10000) transformed) of their transcripts, and dN/dS (ratio of synonymous to nonsynonymous gene mutations). Taken together, these illustrate the complex relationships between target proteins and identity, conservation and abundance.

References:

1. Suarez RK. Hummingbird flight: sustaining the highest mass-specific metabolic rates among vertebrates. Experientia. 1992;48:565–70.

2. Chai P, Dudley R. Limits to flight energetics of hummingbirds hovering in hypodense and hypoxic gas mixtures. J. Exp. Biol. 1996;199:2285–95.

3. Suarez RK, Lighton JR, Moyes CD, Brown GS, Gass CL, Hochachka PW. Fuel selection in rufous hummingbirds: ecological implications of metabolic biochemistry. Proc. Natl. Acad. Sci. U. S. A. 1990;87:9207–10.

4. Chen CCW, Welch KC. Hummingbirds can fuel expensive hovering flight completely with either exogenous glucose or fructose. Funct. Ecol. 2014;28:589–600.

5. Welch KC Jr, Altshuler DL, Suarez RK. Oxygen consumption rates in hovering hummingbirds reflect substrate-dependent differences in P/O ratios: carbohydrate as a "premium fuel." J. Exp. Biol. 2007;210:2146–53.

6. Baker HG. Sugar Concentrations in Nectars from Hummingbird Flowers. Biotropica. [Association for Tropical Biology and Conservation, Wiley]; 1975;7:37–41.

7. Welch KC Jr, Chen CCW. Sugar flux through the flight muscles of hovering vertebrate nectarivores: a review. J. Comp. Physiol. B. Springer; 2014;184:945–59.

8. Powers DR, Brown AR, Van Hook JA. Influence of normal daytime fat deposition on laboratory measurements of torpor use in territorial versus nonterritorial hummingbirds. Physiol. Biochem. Zool. 2003;76:389–97.

9. Hou L, Verdirame M, Welch KC. Automated tracking of wild hummingbird mass and energetics over multiple time scales using radio frequency identification (RFID) technology. J. Avian Biol. Blackwell Publishing Ltd; 2015;46:1–8.

10. Weidensaul S, Robinson TR, Sargent RR, Sargent MB, Poole A. Ruby-throated

hummingbird (Archilochus colubris). Birds of North America Online (A. Poole, Editor). Cornell Lab of Ornithology, Ithaca, NY, USA. http://bna. birds. cornell. edu/bna/species/204. 2013;

11. Carpenter FL, Hixon MA, Beuchat CA, Russell RW, Paton DC. Biphasic Mass Gain in Migrant Hummingbirds: Body Composition Changes, Torpor, and Ecological Significance. Ecology. Ecological Society of America; 1993;74:1173–82.

12. Hou L, Welch KC Jr. Premigratory ruby-throated hummingbirds, Archilochus colubris, exhibit multiple strategies for fuelling migration. Anim. Behav. 2016;121:87–99.

13. Hermier D. Lipoprotein metabolism and fattening in poultry. J. Nutr. 1997;127:805S – 808S.

14. Suarez RK, Brownsey RW, Vogl W, Brown GS, Hochachka PW. Biosynthetic capacity of hummingbird liver. Am. J. Physiol. 1988;255:R699–702.

15. Vianna CR, Hagen T, Zhang CY, Bachman E, Boss O, Gereben B, et al. Cloning and functional characterization of an uncoupling protein homolog in hummingbirds. Physiol. Genomics. 2001;5:137–45.

16. Fan L, Gardner P, Chan SJ, Steiner DF. Cloning and analysis of the gene encoding hummingbird proinsulin. Gen. Comp. Endocrinol. 1993;91:25–30.

17. Welch KC Jr, Allalou A, Sehgal P, Cheng J, Ashok A. Glucose transporter expression in an avian nectarivore: the ruby-throated hummingbird (Archilochus colubris). PLoS One. 2013;8:e77003.

18. Braun EJ, Sweazea KL. Glucose regulation in birds. Comp. Biochem. Physiol. B Biochem. Mol. Biol. 2008;151:1–9.

19. Polakof S, Mommsen TP, Soengas JL. Glucosensing and glucose homeostasis: from fish to mammals. Comp. Biochem. Physiol. B Biochem. Mol. Biol. 2011;160:123–49.

20. Jarvis ED, Mirarab S, Aberer AJ, Li B, Houde P, Li C, et al. Phylogenomic analyses data of the avian phylogenomics project. Gigascience. gigascience.biomedcentral.com; 2015;4:4.

21. Gregory TR, Andrews CB, McGuire JA, Witt CC. The smallest avian genomes are found in hummingbirds. Proc. Biol. Sci. rspb.royalsocietypublishing.org; 2009;276:3753–7.

22. Hughes AL, Hughes MK. Small genomes for better flyers. Nature. 1995;377:391.

23. Abdel-Ghany SE, Hamilton M, Jacobi JL, Ngam P, Devitt N, Schilkey F, et al. A survey of the sorghum transcriptome using single-molecule long reads. Nat. Commun. 2016;7:11706.

24. Pyle P. Identification Guide to North American Birds, Part I: Columbidae to Ploceidae: Peter Pyle: 9780961894023: Books - Amazon.ca [Internet]. [cited 2017 Oct 1]. Available from: https://www.amazon.ca/Identification-Guide-North-American-Birds/dp/0961894024

25. Chomczynski P, Mackey K. Short technical reports. Modification of the TRI reagent procedure for isolation of RNA from polysaccharide- and proteoglycan-rich sources. Biotechniques. 1995;19:942–5.

26. Thomas S, Underwood JG, Tseng E, Holloway AK, Bench To Basinet CvDC Informatics Subcommittee. Long-read sequencing of chicken transcripts and identification of new transcript

isoforms. PLoS One. 2014;9:e94650.

27. Genomic Consensus [Internet]. Github. Available from: https://github.com/PacificBiosciences/GenomicConsensus

28. Workman RE, Myrka AM, Tseng E, Wong GW, Welch KC, Timp W. Supporting data for "Single molecule, full-length transcript sequencing provides insight into the extreme metabolism of ruby-throated hummingbird Archilochus colubris". *GigaScience* Database 2018. http://dx.doi.org/10.5524/100403

29. Workman RE, Myrka AM, Tseng E, Wong GW, Welch KC, Timp W. Single molecule, full-length transcript sequencing provides insight into the extreme metabolism of ruby-throated hummingbird Archilochus colubris [Data set]. Zenodo 2017. http://doi.org/10.5281/zenodo.311651

30. Assessing genome assembly and annotation completeness with Benchmarking Universal Single-Copy Orthologs (BUSCO) [Internet]. Available from: https://gitlab.com/ezlab/busco

31. Simão FA, Waterhouse RM, Ioannidis P, Kriventseva EV, Zdobnov EM. BUSCO: assessing genome assembly and annotation completeness with single-copy orthologs. Bioinformatics. 2015;31:3210–2.

32. Wu TD, Watanabe CK. GMAP: a genomic mapping and alignment program for mRNA and EST sequences. Bioinformatics. 2005;21:1859–75.

33. McGuire JA, Witt CC, Altshuler DL, Remsen JV Jr. Phylogenetic systematics and biogeography of hummingbirds: Bayesian and maximum likelihood analyses of partitioned data and selection of an appropriate partitioning strategy. Syst. Biol. 2007;56:837–56.

34. Licona-Vera Y, Ornelas JF. The conquering of North America: dated phylogenetic and biogeographic inference of migratory behavior in bee hummingbirds. BMC Evol. Biol. 2017;17:126.

35. Tseng E. Robust Open Reading Frame prediction (ANGLE re-implementation) [Internet]. Available from: https://github.com/PacificBiosciences/ANGEL

36. Coding Genome Reconstruction using Iso-Seq data [Internet]. Elizabeth Tseng. Available from: https://github.com/Magdoll/Cogent

37. Wang B, Tseng E, Regulski M, Clark TA, Hon T, Jiao Y, et al. Unveiling the complexity of the maize transcriptome by single-molecule long-read sequencing. Nat. Commun. 2016;7:11708.

38. Gilbert, T, M, P; Jarvis, E, D; Li, B; Li, C; Mello, C, V; The Avian Genome Consortium; Wang, J; Zhang, G (2014): Genomic data of the Anna's Hummingbird (Calypte anna). GigaScience Database. http://dx.doi.org/10.5524/101004

39. Walker BJ, Abeel T, Shea T, Priest M, Abouelliel A, Sakthikumar S, et al. Pilon: an integrated tool for comprehensive microbial variant detection and genome assembly improvement. PLoS One. 2014;9:e112963.

40. Li L, Stoeckert CJ Jr, Roos DS. OrthoMCL: identification of ortholog groups for eukaryotic

genomes. Genome Res. 2003;13:2178–89.

41. Mi H, Muruganujan A, Casagrande JT, Thomas PD. Large-scale gene function analysis with the PANTHER classification system. Nat. Protoc. 2013;8:1551–66.

42. Mi H, Huang X, Muruganujan A, Tang H, Mills C, Kang D, et al. PANTHER version 11: expanded annotation data from Gene Ontology and Reactome pathways, and data analysis tool enhancements. Nucleic Acids Res. 2017;45:D183–9.

43. Zhang G, Rahbek C, Graves GR, Lei F, Jarvis ED, Gilbert MTP. Genomics: Bird sequencing project takes off. Nature. 2015;522:34.

44. Yang Z. PAML 4: phylogenetic analysis by maximum likelihood. Mol. Biol. Evol. 2007;24:1586–91.

45. Jacob E, Unger R. A tale of two tails: why are terminal residues of proteins exposed? Bioinformatics. 2007;23:e225–30.

46. Kryazhimskiy S, Plotkin JB. The population genetics of dN/dS. PLoS Genet. 2008;4:e1000304.

47. Joost HG, Thorens B. The extended GLUT-family of sugar/polyol transport facilitators: nomenclature, sequence characteristics, and potential function of its novel members (review). Mol. Membr. Biol. 2001;18:247–56.

48. Ohtsubo K, Takamatsu S, Gao C, Korekane H, Kurosawa TM, Taniguchi N. N-Glycosylation modulates the membrane sub-domain distribution and activity of glucose transporter 2 in pancreatic beta cells. Biochem. Biophys. Res. Commun. 2013;434:346–51.

49. Zhang JZ, Abbud W, Prohaska R, Ismail-Beigi F. Overexpression of stomatin depresses GLUT-1 glucose transporter activity. Am. J. Physiol. Cell Physiol. 2001;280:C1277–83.

50. Thorens B, Mueckler M. Glucose transporters in the 21st Century. Am. J. Physiol. Endocrinol. Metab. 2010;298:E141–5.

51. Beuchat CA, Chong CR. Hyperglycemia in hummingbirds and its consequences for hemoglobin glycation. Comp. Biochem. Physiol. A Mol. Integr. Physiol. 1998;120:409–16.

52. Suzuki M, Fujimoto W, Goto M, Morimatsu M, Syuto B, Iwanaga T. Cellular expression of gut chitinase mRNA in the gastrointestinal tract of mice and chickens. J. Histochem. Cytochem. 2002;50:1081–9.

53. Kosuri S, Church GM. Large-scale de novo DNA synthesis: technologies and applications. Nat. Methods. 2014;11:499–507.

54. Suarez RK, Welch KC Jr, Hanna SK, Herrera M LG. Flight muscle enzymes and metabolic flux rates during hovering flight of the nectar bat, Glossophaga soricina: further evidence of convergence with hummingbirds. Comp. Biochem. Physiol. A Mol. Integr. Physiol. 2009;153:136–40.

55. FernándezM.J., BozinovicF., SuarezR.K. Enzymatic flux capacities in hummingbird flight muscles: a “one size fits all” hypothesis. Can. J. Zool. 2011;89:985–91.

Figure
Click here to download Figure 180102_workman_figures.pdf

A]

Size Fraction	1-2kb	2-3kb	3-6kb	5-10kb	Total
# of SMRTcells	10	10	10	10	40
Reads of Insert (ROI)	688,069	591,050	735,670	625,194	2,639,983
Avg length ROI (bp)	1533	2464	3650	5444	
ROI Yield (Mbp)	1055	1457	2685	3404	8601
Filtered full length reads	430,381	306,841	272,781	193,906	1,203,909
# Consensus Isoforms	359,981	163,618	209,969	121,109	807,114
HQ consensus isoforms	41,763	25,776	24,735	7,436	94,724
% HQ	11.60%	15.75%	11.78%	6.14%	11.74%
Avg HQ length	1315	2329	3629	5491	
LQ consensus isoforms	321,101	135,415	186,523	113,162	712,210
% LQ	89.20%	82.76%	88.83%	93.44%	88.56%
Avg LQ length	1503	2621	4170	6718	

B]

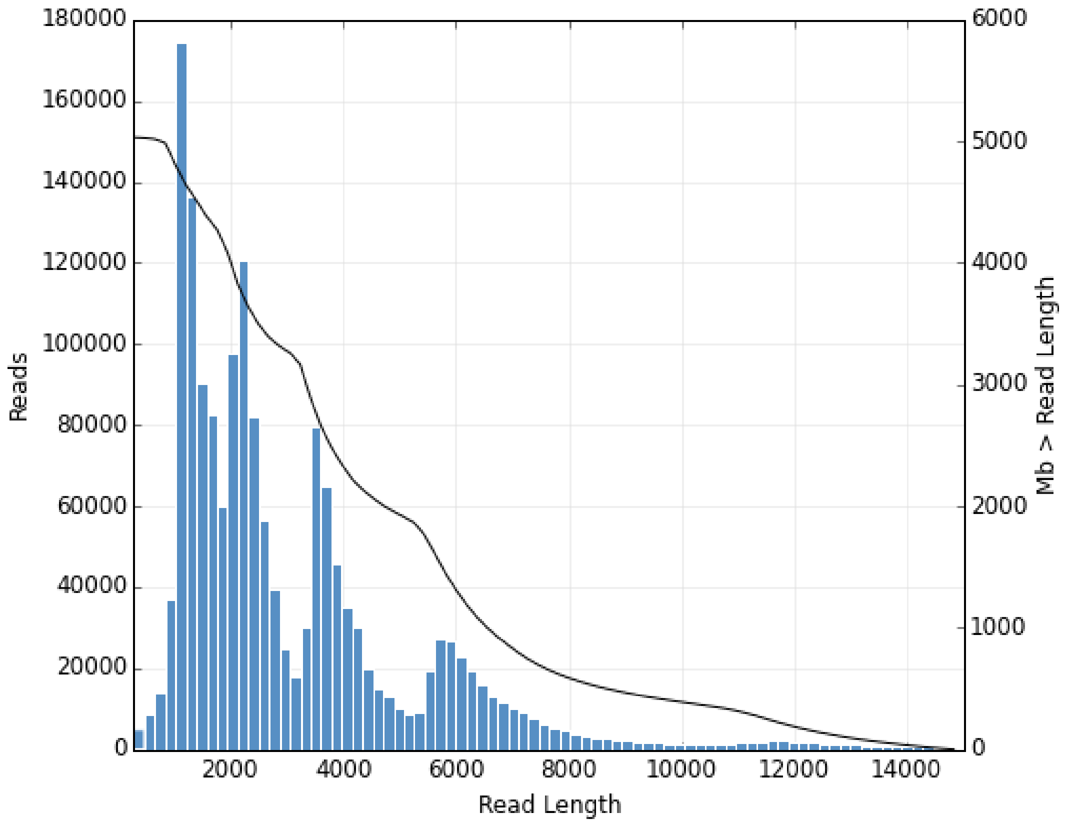

C] BUSCO ASSESSMENT RESULTS

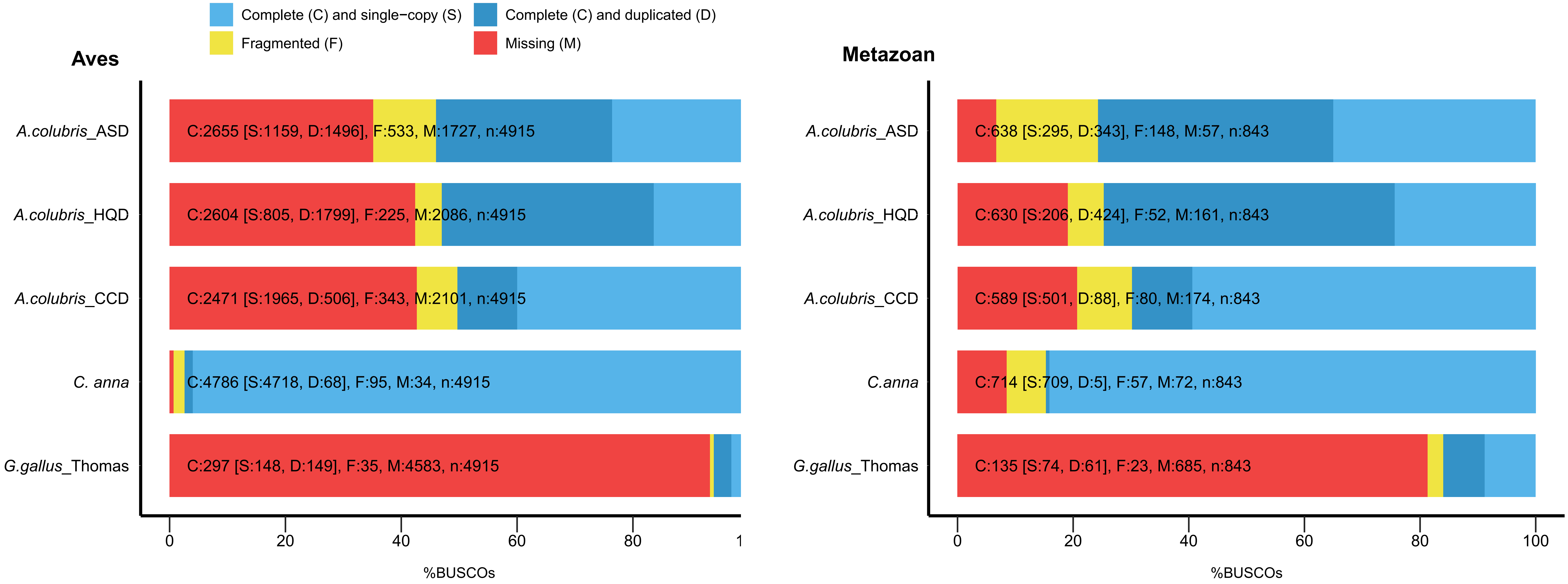

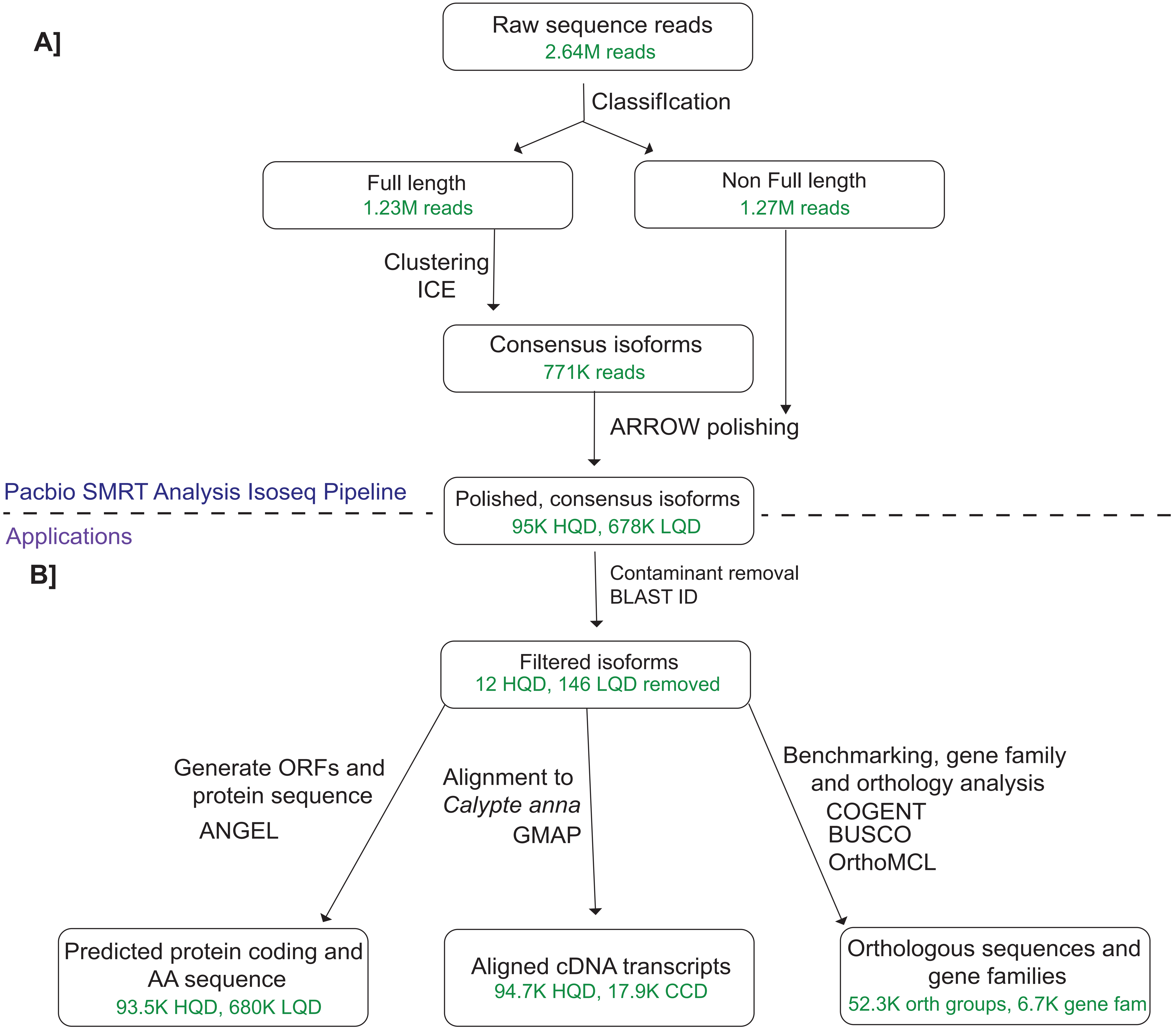

A]
Raw sequence reads
2.64M reads
ClassifIcation
Full length
1.23M reads
Non Full length
1.27M reads
Clustering
ICE
Consensus isoforms
771K reads
ARROW polishing
Pacbio SMRT Analysis Isoseq Pipeline
Polished, consensus isoforms
95K HQD, 678K LQD
Applications
B]
Contaminant removal
BLAST ID
Filtered isoforms
12 HQD, 146 LQD removed
Generate ORFs and
protein sequence
ANGEL
Alignment to
Calypte anna
GMAP
Benchmarking, gene family
and orthology analysis
COGENT
BUSCO
OrthoMCL
Predicted protein coding and
AA sequence
93.5K HQD, 680K LQD
Aligned cDNA transcripts
94.7K HQD, 17.9K CCD
Orthologous sequences and
gene families
52.3K orth groups, 6.7K gene fam

A]

Clustering results	Counts
Total high quality (HQ) Isoforms	94724
Total grouped by Cogent	91733
Orphan seqs (likely single-isoform)	2991
Gene families predicted	6727

Gene family alignment: GMAP	Counts	Percent
Unaligned	1068	5.97%
Multi-mapped	2614	14.62%
Uniquely Mapped	15262	85.37%
qCoverage = 100%	10076	56.36%
qCoverage >= 99%:	14018	78.41%
qCoverage >= 90%	14559	81.44%
Total number transcripts	17877	100.00%

Cogent comparison cases	In Cogent	In Ref	#families
Single gene locus	1	1	5258
Missing gene, possible broken	1	>1	176
Missing gene	1	0	38
Unresolvable to 1 contig	>1	1	836
Possible multi-loci gene	>1	>1	419
Total gene families			6727

B]

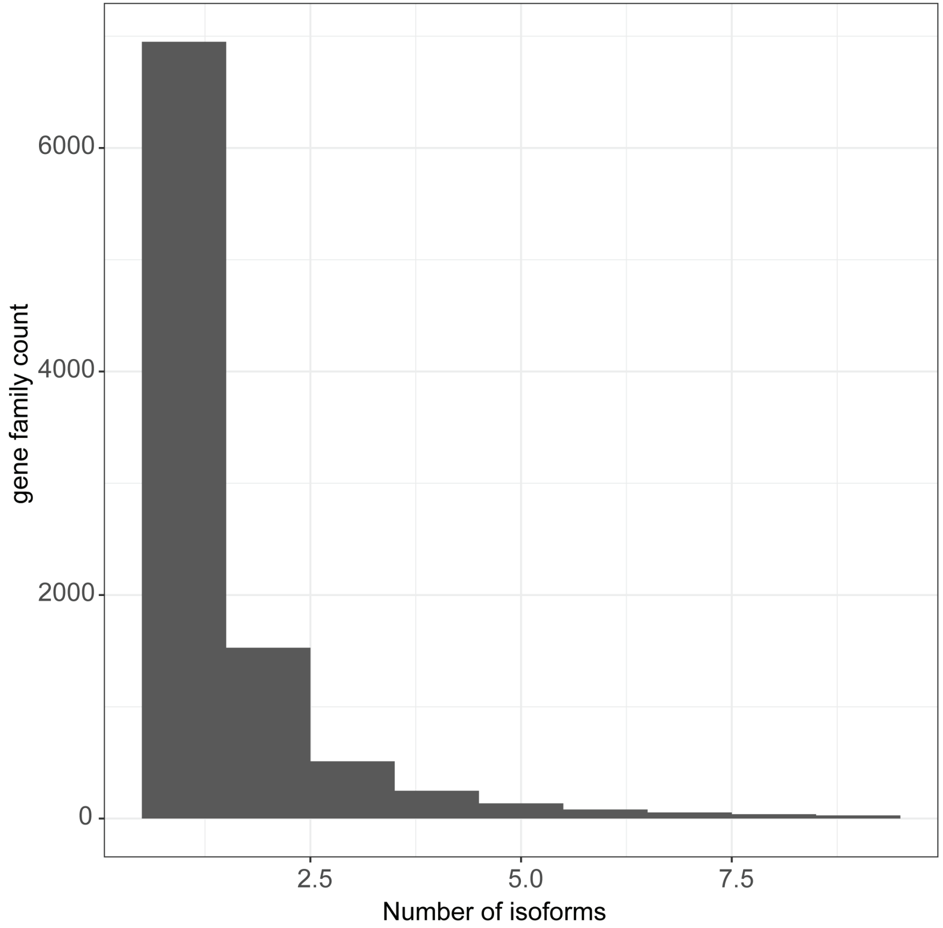

C]

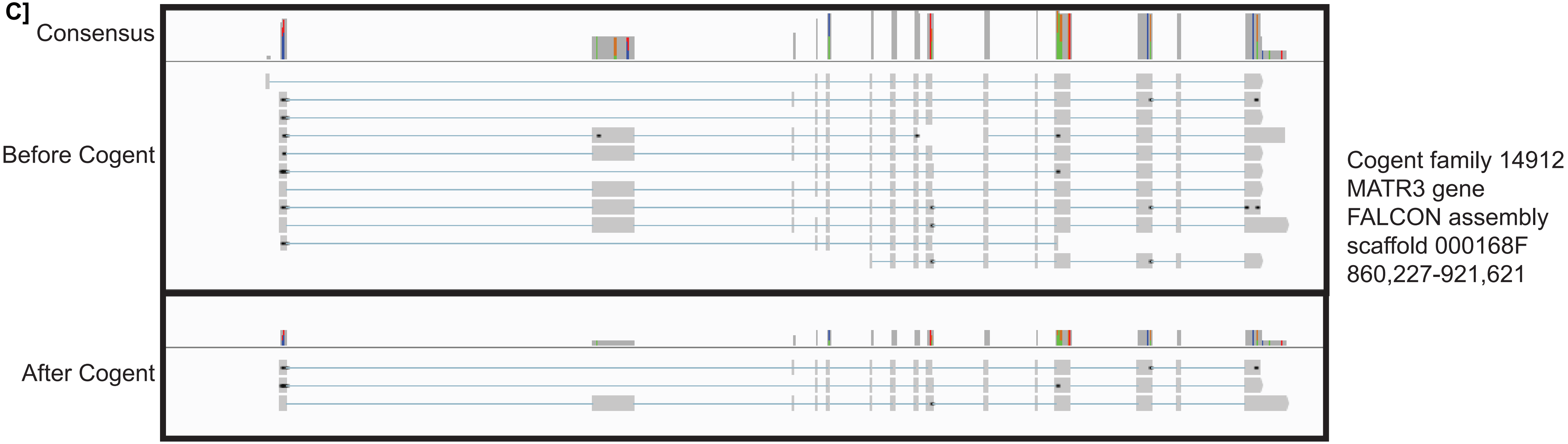

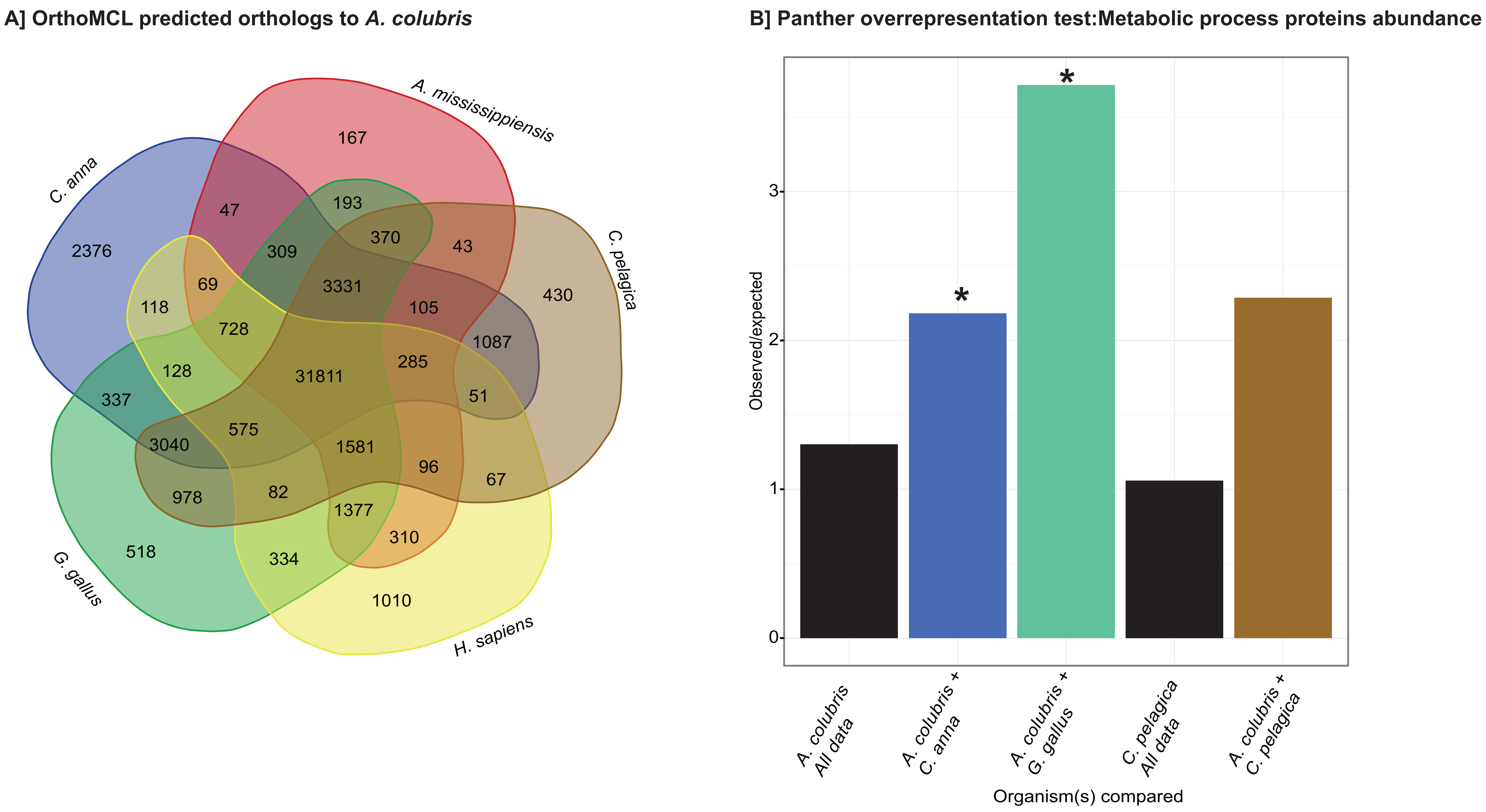

A] OrthoMCL predicted orthologs to A. colubris
B] Panther overrepresentation test:Metabolic process proteins abundance
C. anna
A. mississippiensis
C. pelagica
H. sapiens
G. gallus
167
47
193
370
43
2376
309
69
3331
105
430
118
728
1087
128
31811
285
337
51
575
3040
1581
96
67
978
82
1377
310
518
334
1010
Observed/expected
0
1
2
3
*
*
A. colubris All data
A. colubris + C. anna
A. colubris + G. gallus
C. pelagica All data
A. colubris + C. pelagica
Organism(s) compared

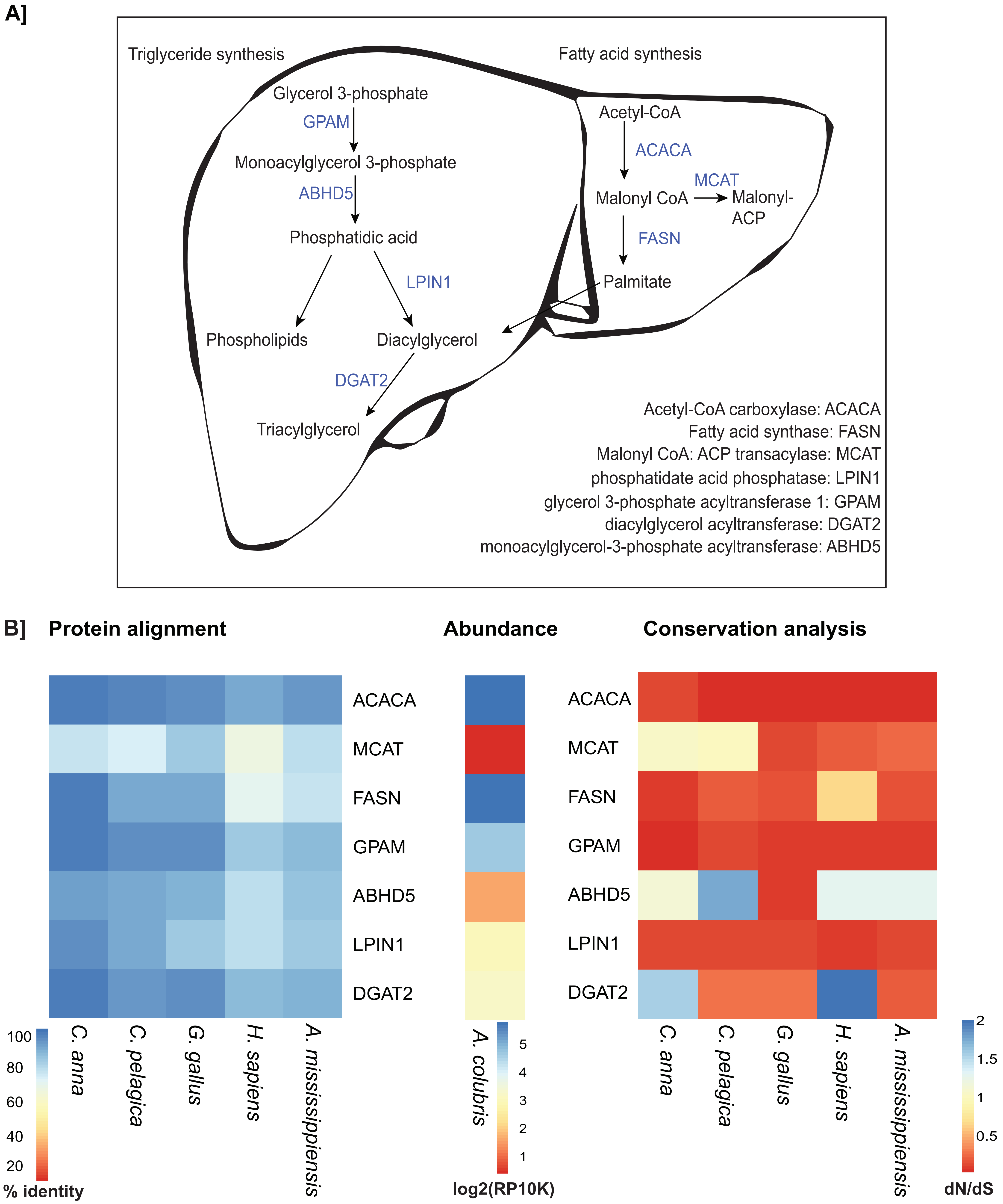
A]
Triglyceride synthesis
Fatty acid synthesis
Glycerol 3-phosphate
GPAM
Monoacylglycerol 3-phosphate
ABHD5
Phosphatidic acid
LPIN1
Phospholipids
Diacylglycerol
DGAT2
Triacylglycerol
Acetyl-CoA
ACACA
Malonyl CoA
MCAT
Malonyl-ACP
FASN
Palmitate
Acetyl-CoA carboxylase: ACACA
Fatty acid synthase: FASN
Malonyl CoA: ACP transacylase: MCAT
phosphatidate acid phosphatase: LPIN1
glycerol 3-phosphate acyltransferase 1: GPAM
diacylglycerol acyltransferase: DGAT2
monoacylglycerol-3-phosphate acyltransferase: ABHD5
B]
Protein alignment
Abundance
Conservation analysis
ACACA
MCAT
FASN
GPAM
ABHD5
LPIN1
DGAT2
C. anna
C. pelagica
G. gallus
H. sapiens
A. mississippiensis
A. colubris
100
80
60
40
20
% identity
5
4
3
2
1
log2(RP10K)
2
1.5
1
0.5
dN/dS

Click here to access/download
Supplementary Material
Supplemental_methods_workman_180102.pdf

Supplementary Data

Click here to access/download
Supplementary Material
171009_suppdata.pdf
