# Supplementary material for: Single-molecule, full-length transcript sequencing provides insight into the extreme metabolism of the ruby-throated hummingbird Archilochus colubris
Source: Gigascience. 2018 Feb 15;7(3):giy009. doi: 10.1093/gigascience/giy009 (PMC5869288; doi:10.1093/gigascience/giy009)
Supplement: Reviewer_1_(Original_Submission)_(Attachment).pdf [file giy009_reviewer_1_(original_submission)_(attachment).pdf]

Workman *et al.* have presented a manuscript on a very relevant topic - third generation sequencing on an fascinating bird, the ruby-throated hummingbird. While I wholeheartedly agree that long read sequencing will address several assembly, and downstream, problems - resulting in a better understanding of several genetic aspects of any organism, there are several inaccuracies in the current manuscript that need to be addressed before publication.

1. There are several transcripts (about 155) from viruses - SRR5237173.540969 is one such example. It encodes a 823 long ORF, which has Identities = 702/824 (85%) with a FBS protein (NP_955606.1) from Fujinami sarcoma virus (FSV). The relation of FSV to avian genomes has been long known (http://www.pnas.org/content/77/4/2018). This should modify, however minimally, the statement 'resulted in 119,292 HQD and 1,061,147 ASD peptide sequences'. Similar techniques should be used for eliminating bacterial and fungal transcripts. Methods for quickly detecting metagenomic transcripts have been elucidated in http://biorxiv.org/content/early/2016/10/04/079186. Also, ORF-based annotation help in filtering bacterial transcripts from PacBio reads (http://biorxiv.org/content/early/2017/01/17/100974).

2. Figure 5A, and the associated analysis ('poor pairwise protein alignment between A. colubris and all examined species, such as with DGAT2, is suggestive of misannotation or splice variation in our transcriptome, cases with variable alignment identities provide interesting targets for further investigation') is incorrect. The DGAT2 from C. anna (XP_008493408.1) is 358 aa long, and is 357 aa identical to the ORF encoded by SRR5237173.336808. The color coding in Fig5A suggests about 60% identity, based on other transcripts (SRR5237173.185657, SRR5237173.22637, etc - there are 200 homologous transcripts). So, there seems to be two genes for DGAT2, mapping to two different scaffolds in the C. anna genome - an interesting observation is that one gene has very low expression (a single transcript), while the other has several.

3. The PAML numbers, and their evolutionary connotations are not properly explained. Finding the number of genes (ACACA seems to have only one), and possibly quantifying them (roughly, ACACA has about 400 homologous transcripts: some complete - some fragmented) would provide interesting insights in the pathway.

Based on the above mentioned issus, my verdict will be "major revision".
