# Supplementary material for: Single-molecule, full-length transcript sequencing provides insight into the extreme metabolism of the ruby-throated hummingbird Archilochus colubris
Source: Gigascience. 2018 Feb 15;7(3):giy009. doi: 10.1093/gigascience/giy009 (PMC5869288; doi:10.1093/gigascience/giy009)
Supplement: Supplemental material [file giy009_supp.zip › 171009_suppdata.pdf]

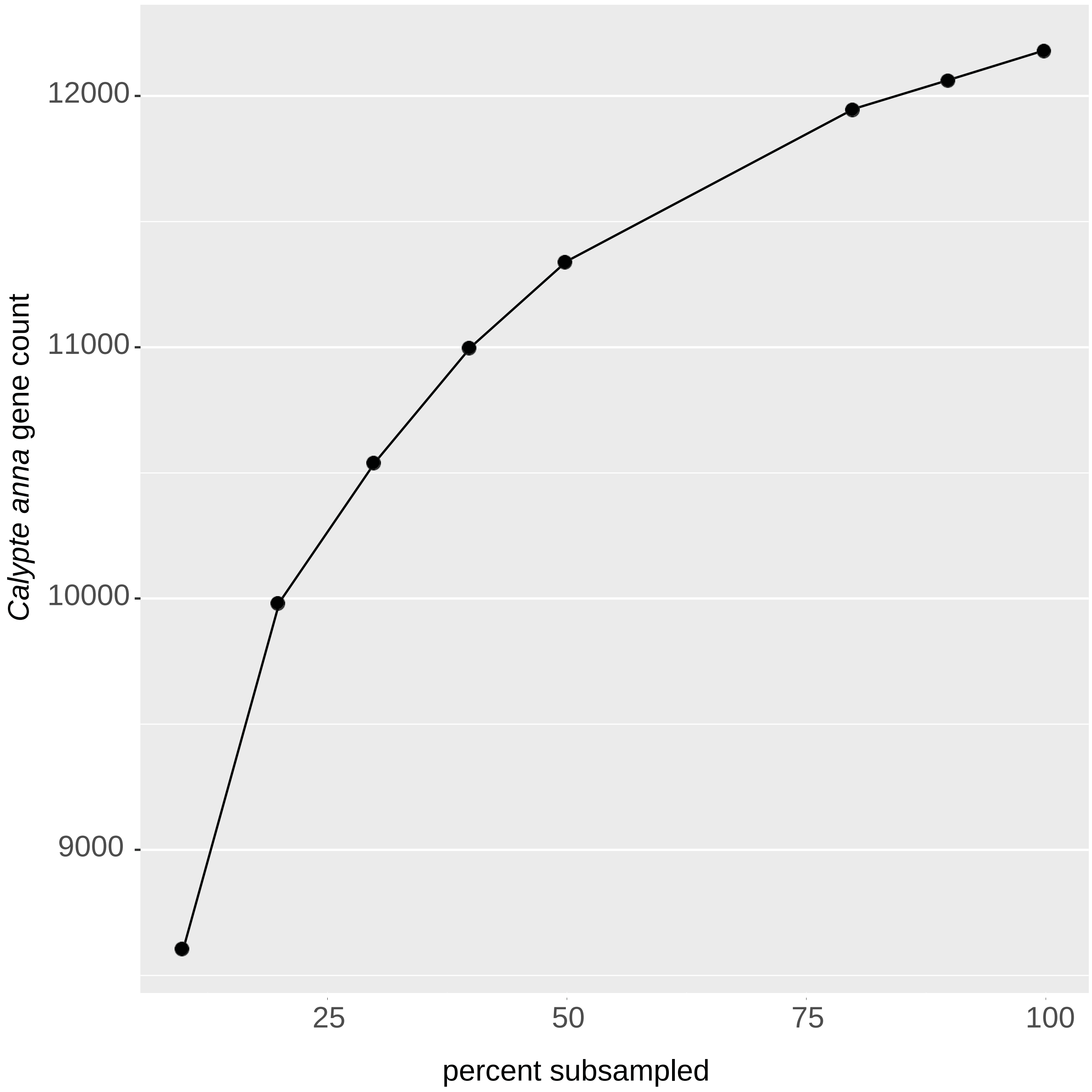

Supplemental Figure 1. Full length non-chimeric circular consensus (CCS) read dataset, comprising 1,219,580 reads, was randomly subsampled by percentage, and the resultant sequences were blasted against the *Calypte anna* gene set (16,000 genes). The number of unique gene hits was plotted against percent sub-sampled and demonstrates that transcript diversity neared saturation with our depth of sequencing.

A]

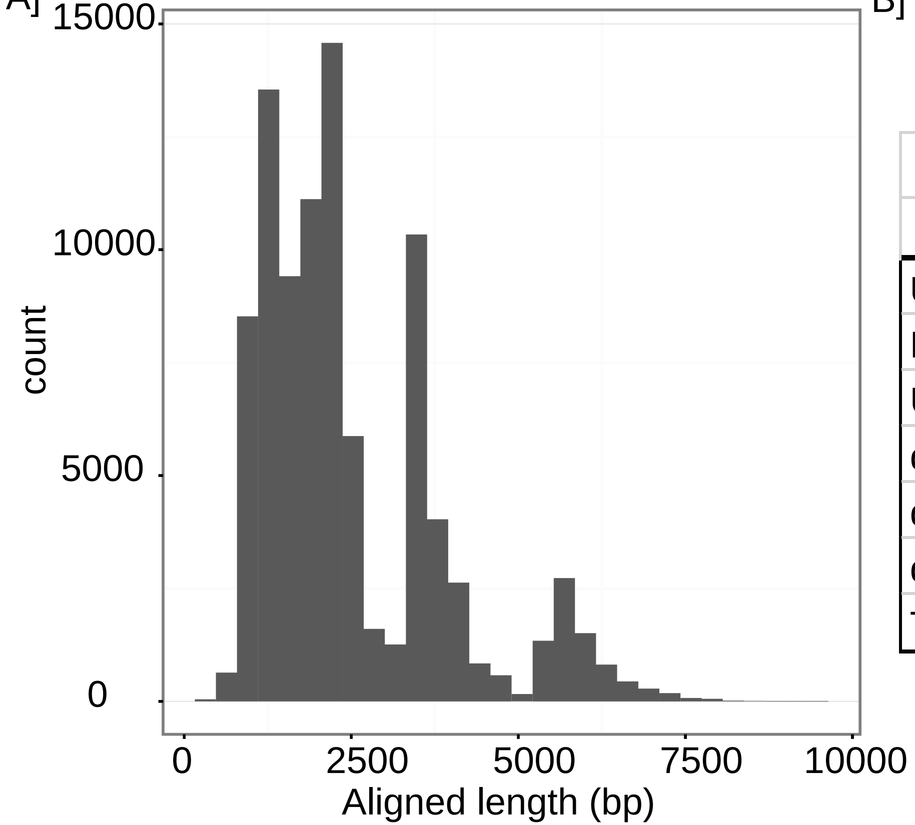

B]

	GMAP alignment to *Calypte anna* FALCON assembly					
	HQD #reads	%	Cogent #reads	%	ASD reads	%
Unaligned	2059	2.17%	1068	5.97%	38641	4.45%
Multi-mapped	4200	4.43%	2614	14.62%	123090	14.17%
Uniquely Mapped	88464	93.39%	15262	85.37%	706928	81.38%
qCoverage = 100%	63466	67.00%	10076	56.36%	419145	48.25%
qCoverage >= 99%:	84108	88.79%	14018	78.41%	594820	68.48%
qCoverage >= 90%	85987	90.78%	14559	81.44%	632313	72.79%
Total number transcripts	94724		17877		868659	

Supplemental Figure 2. Aligned lengths of all sequence data dataset (ASD) by count demonstrates clear benefit of size selection and the efficacy of alignment at longer read lengths. Alignment statistics for high quality Arrow-polished data (HQD), Cogent-collapsed data (CCD), and ASD given in B.

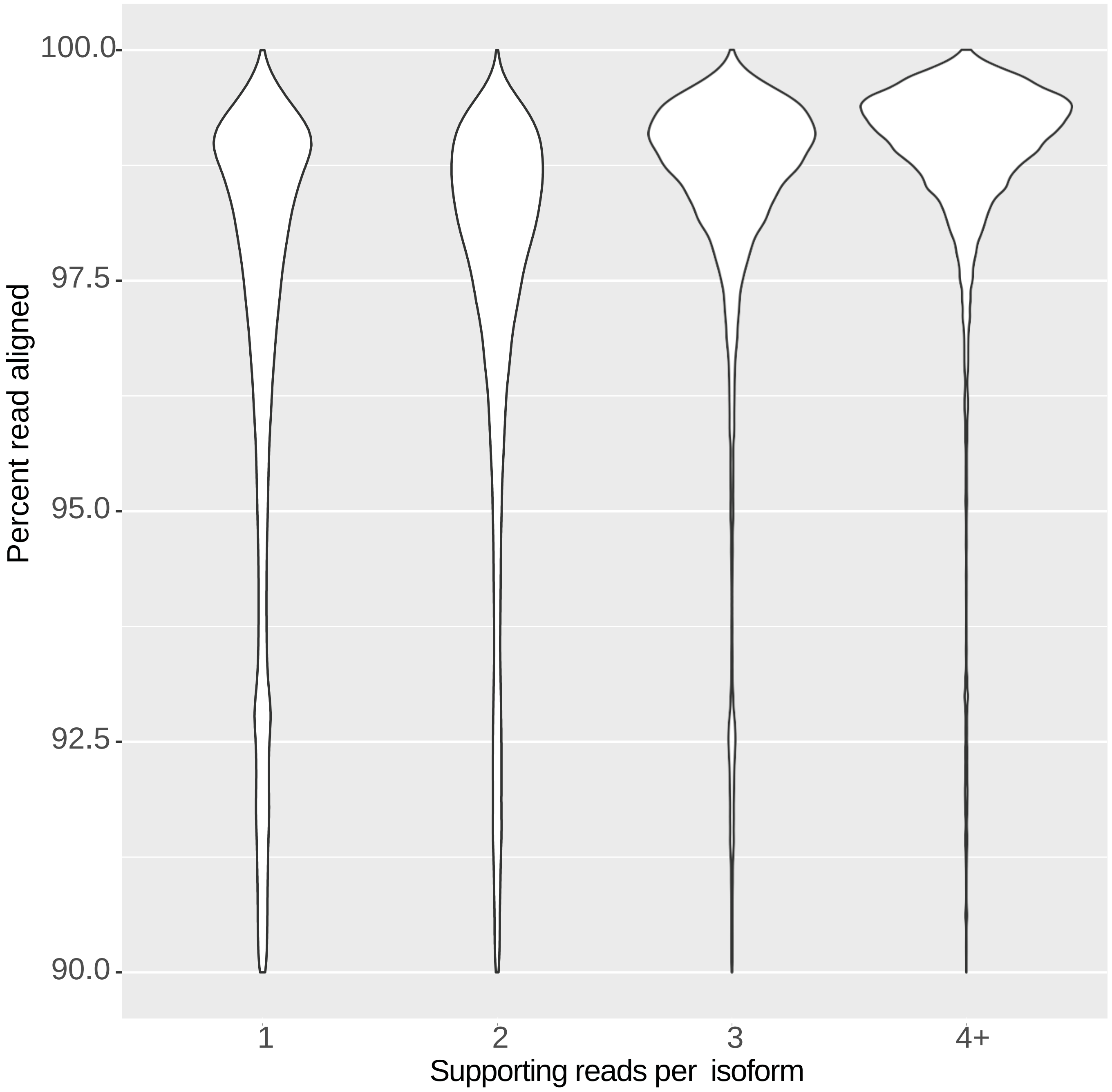

Sup reads	Mean	Median	Count
1	89.7	97	724745
2	95.05	97.9	53873
3	96.7	98.8	24205
4+	96.9	99.1	27195

Supplemental Figure 3. Alignment percentage increases when number of reads supporting a consensus cluster increases. Alignment mean, median, and cluster count given by number of supporting reads in table.

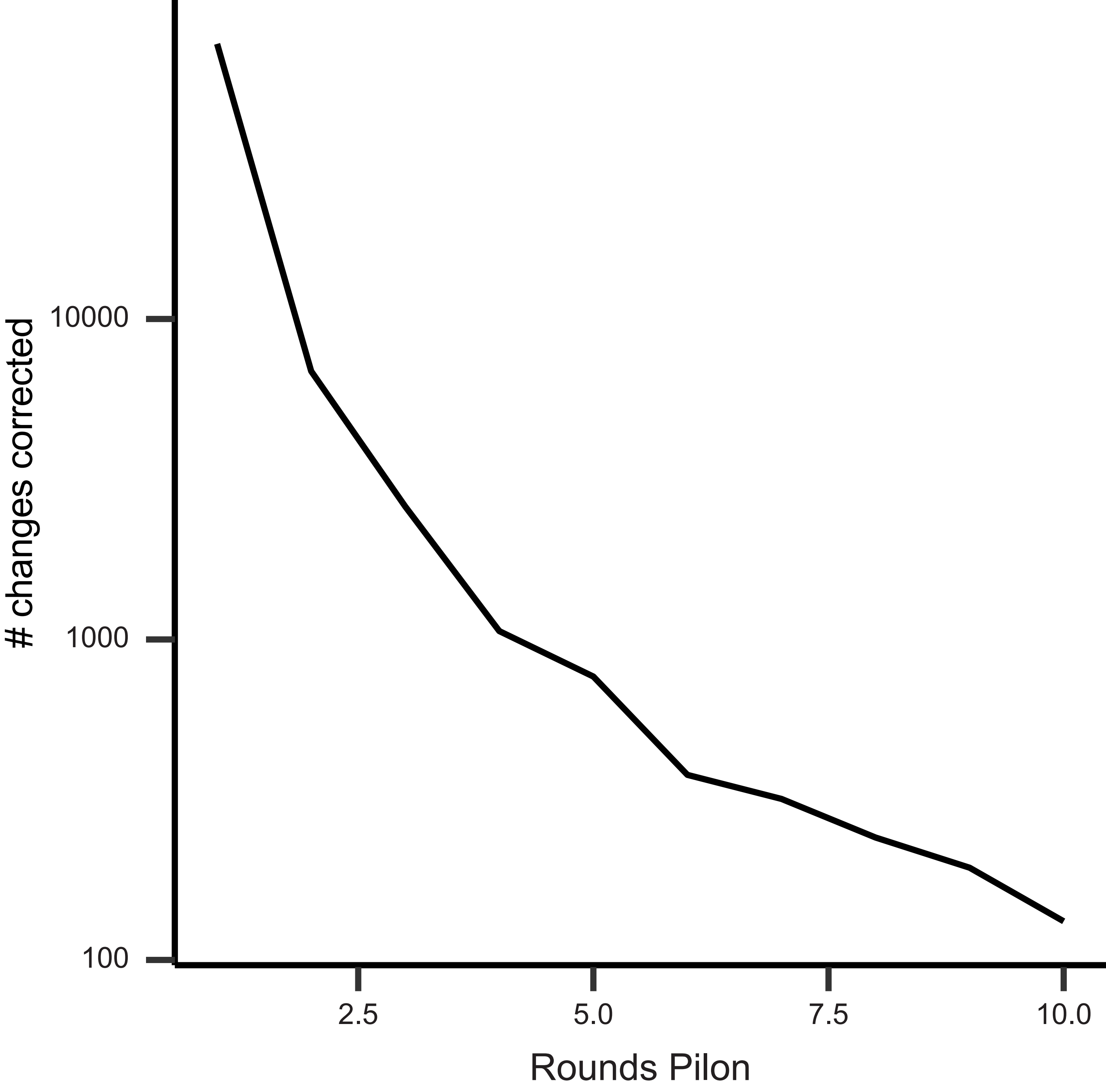

Supplementary Figure 4: Errors corrected per round of pilon correction plotted on a semilog scale. Using the Illumina RNA-seq data to correct the Cogent gene families dataset. The number of errors corrected per round of pilon drops dramatically on subsequent rounds of pilon.

Lineage	Organism<br>***A. colubris*** **(ASD):**	***A. colubris*** **(HQD):**	***A. colubris*** **(CCD)**	***G. gallus*** **(Plos One)**	***C. anna***
**#seqs searched**	**807114**	**94724**	**17618**	**1849778**	**16000**
**Metazoan**	**C:75%[D:40%],F:17%,M:6.7%,n:843**	**C:74.7%[S:24.4%,D:50.3%],F:6.2%,M:19.1%,n:843**	**C:69.8%[S:59.4%,D:10.4%],F:9.5%,M:20.7%,n:843**	**C:16.0%[S:8.8%,D:7.2%],F:2.7%,M:81.3%,n:843**	**C:84.7%[S:84.1%,D:0.6%],F:6.8%,M:8.5%,n:843**
	638 Complete BUSCOs	630 Complete BUSCOs (C)	589 Complete BUSCOs (C)	135 Complete BUSCOs (C)	714 Complete BUSCOs (C)
	295 Complete and single-copy BUSCOs	206 Complete and single-copy BUSCOs (S)	501 Complete and single-copy BUSCOs (S)	74 Complete and single-copy BUSCOs (S)	709 Complete and single-copy BUSCOs (S)
	343 Complete and duplicated BUSCOs	424 Complete and duplicated BUSCOs (D)	88 Complete and duplicated BUSCOs (D)	61 Complete and duplicated BUSCOs (D)	5 Complete and duplicated BUSCOs (D)
	148 Fragmented BUSCOs	52 Fragmented BUSCOs (F)	80 Fragmented BUSCOs (F)	23 Fragmented BUSCOs (F)	57 Fragmented BUSCOs (F)
	57 Missing BUSCOs	161 Missing BUSCOs (M)	174 Missing BUSCOs (M)	685 Missing BUSCOs (M)	72 Missing BUSCOs (M)
	843 Total BUSCO groups searched	843 Total BUSCO groups searched	843 Total BUSCO groups searched	843 Total BUSCO groups searched	843 Total BUSCO groups searched
**Aves**	**C:54.0%[S:23.6%,D:30.4%],F:10.8%,M:35.2%,n:4915**	**C:53.0%[S:16.4%,D:36.6%],F:4.6%,M:42.4%,n:4915**	**C:50.3%[S:40.0%,D:10.3%],F:7.0%,M:42.7%,n:4915**	**C:6.0%[S:3.0%,D:3.0%],F:0.7%,M:93.3%,n:4915**	**C:97.4%[S:96.0%,D:1.4%],F:1.9%,M:0.7%,n:491**
	2655 Complete BUSCOs (C)	2604 Complete BUSCOs (C)	2471 Complete BUSCOs (C)	297 Complete BUSCOs (C)	4786 Complete BUSCOs (C)
	1159 Complete and single-copy BUSCOs (S)	805 Complete and single-copy BUSCOs (S)	1965 Complete and single-copy BUSCOs (S)	148 Complete and single-copy BUSCOs (S)	4718 Complete and single-copy BUSCOs (S)
	1496 Complete and duplicated BUSCOs (D)	1799 Complete and duplicated BUSCOs (D)	506 Complete and duplicated BUSCOs (D)	149 Complete and duplicated BUSCOs (D)	68 Complete and duplicated BUSCOs (D)
	533 Fragmented BUSCOs (F)	225 Fragmented BUSCOs (F)	343 Fragmented BUSCOs (F)	35 Fragmented BUSCOs (F)	95 Fragmented BUSCOs (F)
	1727 Missing BUSCOs (M)	2086 Missing BUSCOs (M)	2101 Missing BUSCOs (M)	4583 Missing BUSCOs (M)	34 Missing BUSCOs (M)
	4915 Total BUSCO groups searched	4915 Total BUSCO groups searched	4915 Total BUSCO groups searched	4915 Total BUSCO groups searched	4915 Total BUSCO groups searched

Supplemental Table 1. BUSCO results. For Metazoan and Aves lineages, gene sets from *Gallus gallus* (single tissue Pacbio Iso-seq), *Calypte anna* whole genome predicted CDS, and *Archilochis colubris* high quality data (HQD), all sequence data (ASD), and Cogent-collapsed data (CCD), number of complete, fragmented and missing BUSCOs are given, as well as percentages of total BUSCO groups searched.

Supplemental Table 2. Cogent results. Gene family prediction statistics given in **A,** and comparison to *Calypte anna* genome in **C**, with number of contigs predicted by transcriptome data compared to those predicted by genomic coding sequences. Of the 6727 gene families, 5472 were reconstructed to a single contig, and 1255 were resolved to 2 or more contigs (**B**). After gene family prediction, reconstructed contigs were used to collapse redundant reads in the high quality (HQD) dataset, bringing read count from 94,724 to 17,618 unique putative isoforms. Most abundant transcripts predicted by Cogent are listed in **D**.

A

**Cogent results**	Count
Total HQ Arrow Isoforms	94724
Grouped by Cogent	91733
Orphan seqs	2991
Gene families predicted	6727
**After collapse**	Count
Orphan seqs	2990
Putative isoforms	14628
Total predicted isoforms	17618

B Reconstructed contig counts

**# contigs**	**Count**
1	5472
2	955
3	176
4	59
5	20
6	18
7	14
8	2

C Cogent comparisons

**In Cogent (#contigs)**	**In *C. anna* assemblies**	**#families**	**Case**
1	1	5258	Single gene locus
1	>1	176	Missing gene, possible broken
1	0	38	Missing gene
>1	1	836	Unresolvable to 1 contig
>1	>1	419	Possible multi-loci gene
		6727	Total

D **Most abundant transcripts**

**Cogent family**	**Count**	**Predicted identity**	
Cogent0_PB.2.1	3471	ALB	Albumin
Cogent88065_PB.1.1	3139	APOB	Apolipoprotein B
Cogent8195_PB.2.1	2909	ACLY	ATP citrate lysase
Cogent90112_PB.9.1	2806	APOA4	Apolipoprotein A-IV
Cogent32771_PB.6.14	2522	GPD1	Glycerol 3-phosphate dehydrogenase 1
Cogent75779_PB.3.1	2485	INSIG1	Insulin induced gene 1
Cogent45056_PB.4.1	2408		Mitochondrial transcript
Cogent16387_PB.7.1	2278	APOA1	Apolipoprotein A-1
Cogent79876_PB.2.4	2244	SLC25A1	Solute carrier family 25 member 1
Cogent8194_PB.4.1	2023	GAPDH	Glyceraldehyde 3-phosphate dehydrogenase

A Ortholog pairs

	A. colubris	*C. anna*	*C. pelagica*	*T. guttata*	*M. undulatus*	*G. gallus*	*A. mississippiensis*	*H. sapiens*
A. colubris	**52355**	7304	8219	7551	7400	20447	13683	20032
C. anna	44397	**14047**	13722	13319	12691	33195	21011	30365
C. pelagica	43932	12294	**15235**	13754	12526	34771	22054	31875
T. guttata	41407	12822	13754	**15044**	12861	33736	21419	31278
M. undulatus	43660	12643	13935	13375	**14344**	34483	21849	31990
G. gallus	45692	12695	14686	13469	13384	**38741**	23809	35096
A. mississippiensis	40822	12414	14396	13034	12988	35902	**26370**	36957
H. sapiens	38622	12163	14099	12794	12793	34512	24067	**41115**

B Co-orthologs

	A. colubris	*C. anna*	*C. pelagica*	*T. guttata*	*M. undulatus*	*G. gallus*	*A. mississippiensis*	*H.sapiens*
A. colubris	**36660**	2747	3462	3360	3034	10749	7430	16436
C. anna	11893	**8516**	941	1176	1012	6089	3247	12426
C. pelagica	13677	824	**9019**	1404	966	6354	3343	13177
T. guttata	14262	1045	1404	**9297**	1320	7654	4014	14013
M. undulatus	11563	966	1021	1312	**8354**	5811	3082	12970
G. gallus	16781	2563	2827	3199	2537	**26604**	6158	19810
A. mississippiensis	19230	1920	2196	2463	1804	8891	**18456**	19604
H. sapiens	29410	5552	6596	6396	5739	21136	13442	**29319**

C Paralogs

A. colubris	84805
C. anna	13410
C. pelagica	3252
T. guttata	3264
M. undulatus	2537
G. gallus	30160
A. mississippiensis	14451
H. sapiens	60437

D Input reads

	Input #reads	Orth groups
A.colubris	119292	52355
C. anna	16000	14047
C. pelagica	15942	15235
T. guttata	18204	15044
M. undulatus	16204	14344
G. gallus	46346	38741
A. mississippiensis	30380	26370
H. sapiens	102251	41115

Supplemental Table 3. Number of unique ortholog pairs (A), co-orthologs (B), and paralogs (C) for eight species compared using OrthoMCL. Number of input reads and orthologous groups of each dataset given in (D).

	Lipid enrichment						
**Dataset**	**Type of data**	**GO terms from**	**Total db GO terms**	**GO terms for workspace**	**expected**	**observed**	**p-value**
C. pelagica	Whole transcriptome	*G. gallus*	15789	3179	84.16	89	1
		H. sapiens	20972	3899	99.5	109	1
G. gallus	OrthoMCL pairs unique	*G. gallus*	15789	293	7.8	29	**4.46E-07**
		H. sapiens	20972	348	8.88	29	**9.43E-06**
C. anna	OrthoMCL pairs unique	*G. gallus*	15789	502	13.29	29	**2.39E-02**
		H. sapiens	20972	617	15.74	33	**1.83E-02**
A. colubris	Whole liver transcriptome	*G. gallus*	15789	2869	76	99	1
		H. sapiens	20972	3519	89.7	114	1
C. pelagica	OrthoMCL pairs unique	*G. gallus*	15789	314	8.31	19	**2.00E-01**
		H. sapiens	20972	388	9.9	20	**6.53E-01**

Supplemental Table 4. Enrichment for lipid metabolism GO terms for avian datasets *Chaetura pelagica* (whole transcriptome and 1:1 orthologs between *C. pelagica* and *A. colubris*), *G. gallus* 1:1 orthologs, *C. anna* 1:1 orthologs, and *A. colubris* whole liver transcriptome. GO terms are procured from genes in both *G. gallus* and *H. sapiens* databases. While both whole transcriptome datasets are not enriched for lipid metabolism genes (p=1), datasets of 1:1 orthologs between *G. gallus*, *C. anna*, *C. pelagica* against *A. colubris* all exhibit significant enrichment. Significant p-values in bold.

Pathway	Organism	Enzyme	Gene symbol	AA NCBI Accession	Full accession name	mRNA NCBI Acc	Full accession name
HLP	canna	Acetyl-CoA carboxylase	ACACA	XP_008489475.1	PREDICTED: acetyl-CoA carboxylase 1 isoform X2 [Calypte anna]	XM_008491253.1	PREDICTED: Calypte anna acetyl-CoA carboxylase alpha (ACACA), transcript variant X2, mRNA
HLP	swift	Acetyl-CoA carboxylase	ACACA	XP_010007346.1	PREDICTED: acetyl-CoA carboxylase 1 isoform X1 [Chaetura pelagica]	XM_010009044.1	PREDICTED: Chaetura pelagica acetyl-CoA carboxylase alpha (ACACA), transcript variant X1, mRNA
HLP	human	Acetyl-CoA carboxylase	ACACA	NP_942131.1	acetyl-CoA carboxylase 1 isoform 1 [Homo sapiens]	NM_198834.2	Homo sapiens acetyl-CoA carboxylase alpha (ACACA), transcript variant 1, mRNA
HLP	gallus	Acetyl-CoA carboxylase	ACACA	NP_990836.1	acetyl-CoA carboxylase [Gallus gallus]	NM_205505.1	Gallus gallus acetyl-CoA carboxylase alpha (ACACA), mRNA
HLP	alligator	Acetyl-CoA carboxylase	ACACA	XP_014463551.1	PREDICTED: acetyl-CoA carboxylase 1 isoform X1 [Alligator mississippiensis]	XM_014608065.2	PREDICTED: Alligator mississippiensis acetyl-CoA carboxylase alpha (ACACA), transcript variant X1, mRNA
HLP	canna	diacylglycerol acyltransfe	DGAT2	XP_008493408.1	PREDICTED: diacylglycerol O-acyltransferase 2 [Calypte anna]	XM_008495186.1	PREDICTED: Calypte anna diacylglycerol O-acyltransferase 2 (DGAT2), mRNA
HLP	swift	diacylglycerol acyltransfe	DGAT2	XP_010005498.1	PREDICTED: diacylglycerol O-acyltransferase 2 [Chaetura pelagica]	XM_010007196.1	PREDICTED: Chaetura pelagica diacylglycerol O-acyltransferase 2 (DGAT2), mRNA
HLP	human	diacylglycerol acyltransfe	DGAT2	NP_115953.2	diacylglycerol O-acyltransferase 2 isoform 1 [Homo sapiens]	NM_032564.4	Homo sapiens diacylglycerol O-acyltransferase 2 (DGAT2), transcript variant 1, mRNA
HLP	gallus	diacylglycerol acyltransfe	DGAT2	XP_419374.3	PREDICTED: diacylglycerol O-acyltransferase 2 [Gallus gallus]	XM_419374.5	PREDICTED: Gallus gallus diacylglycerol O-acyltransferase homolog 2 (mouse) (DGAT2), mRNA
HLP	alligator	diacylglycerol acyltransfe	DGAT2	XP_014456982.1	PREDICTED: diacylglycerol O-acyltransferase 2 [Alligator mississippiensis]	XM_014601496.2	PREDICTED: Alligator mississippiensis diacylglycerol O-acyltransferase 2 (DGAT2), mRNA
HLP	canna	fatty acid synthase	FASN	KFP01543.1	Fatty acid synthase [Calypte anna]	XM_008494574.1	PREDICTED: Calypte anna fatty acid synthase (FASN), mRNA
HLP	swift	fatty acid synthase	FASN	KFU85956.1	Fatty acid synthase [Chaetura pelagica]	XM_010008649.1	PREDICTED: Chaetura pelagica fatty acid synthase (FASN), mRNA
HLP	human	fatty acid synthase	FASN	NP_004095.4	fatty acid synthase [Homo sapiens]	NM_004104.4	Homo sapiens fatty acid synthase (FASN), mRNA
HLP	gallus	fatty acid synthase	FASN	NP_990486.2	fatty acid synthase [Gallus gallus]	NM_205155.2	Gallus gallus fatty acid synthase (FASN), mRNA
HLP	alligator	fatty acid synthase	FASN	XP_006038234.1	PREDICTED: fatty acid synthase [Alligator sinensis]	XM_006038172.2	PREDICTED: Alligator sinensis fatty acid synthase (FASN), mRNA
HLP	canna	glycerol-3-phosphate acy	GPAM	XP_008488364	PREDICTED: glycerol-3-phosphate acyltransferase 1, mitochondrial [Calypte anna]	XM_008490142.1	PREDICTED: Calypte anna glycerol-3-phosphate acyltransferase, mitochondrial (GPAM), mRNA
HLP	swift	glycerol-3-phosphate acy	GPAM	XP_010006217.1	PREDICTED: glycerol-3-phosphate acyltransferase 1, mitochondrial [Chaetura pelagica]	XM_010007915.1	PREDICTED: Chaetura pelagica glycerol-3-phosphate acyltransferase, mitochondrial (GPAM), mRNA
HLP	human	glycerol-3-phosphate acy	GPAM	NP_001231878.1	glycerol-3-phosphate acyltransferase 1, mitochondrial precursor [Homo sapiens]	NM_001244949.1	Homo sapiens glycerol-3-phosphate acyltransferase, mitochondrial (GPAM), transcript variant 1, mRNA
HLP	gallus	glycerol-3-phosphate acy	GPAM	XP_015144456.1	PREDICTED: glycerol-3-phosphate acyltransferase 1, mitochondrial isoform X1 [Gallus gallus]	XM_015288965.1	PREDICTED: Gallus gallus glycerol-3-phosphate acyltransferase, mitochondrial (GPAM), transcript variant X1, mRNA
HLP	alligator	glycerol-3-phosphate acy	GPAM	KYO40569.1	glycerol-3-phosphate acyltransferase 1, mitochondrial [Alligator mississippiensis]	XM_006277962.3	PREDICTED: Alligator mississippiensis glycerol-3-phosphate acyltransferase, mitochondrial (GPAM), transcript variant X1, mRNA
HLP	canna	glycerol-3-phosphate acy	GPAT4	KFP06716	Glycerol-3-phosphate acyltransferase 4, partial [Calypte anna]	N/A	NA
HLP	swift	glycerol-3-phosphate acy	GPAT4	KFU86340.1	Glycerol-3-phosphate acyltransferase 4, partial [Chaetura pelagica]	NA	NA
HLP	human	glycerol-3-phosphate acy	GPAT4	NP_848934.1	glycerol-3-phosphate acyltransferase 4 [Homo sapiens]	NM_178819.3	Homo sapiens glycerol-3-phosphate acyltransferase 4 (GPAT4), mRNA
HLP	gallus	glycerol-3-phosphate acy	GPAT4	XP_015152890.1	PREDICTED: glycerol-3-phosphate acyltransferase 4 [Gallus gallus]	XM_015297404.1	PREDICTED: Gallus gallus 1-acylglycerol-3-phosphate O-acyltransferase 6 (lysophosphatidic acid acyltransferase, zeta) (AGPAT6), transcript variant X1, mRNA
HLP	alligator	glycerol-3-phosphate acy	GPAT4	KYO28476.1	glycerol-3-phosphate acyltransferase 4 [Alligator mississippiensis]	XM_006273518.2	PREDICTED: Alligator mississippiensis glycerol-3-phosphate acyltransferase 4 (GPAT4), mRNA
HLP	canna	malonyl CoA:ACP acyltra	MCAT	XP_008488495	PREDICTED: malonyl-CoA-acyl carrier protein transacylase, mitochondrial [Calypte anna]	XM_008490273.1	PREDICTED: Calypte anna malonyl CoA:ACP acyltransferase (mitochondrial) (MCAT), mRNA
HLP	swift	malonyl CoA:ACP acyltra	MCAT	XP_010001330.1	PREDICTED: malonyl-CoA-acyl carrier protein transacylase, mitochondrial [Chaetura pelagica]	XM_010003028.1	PREDICTED: Chaetura pelagica malonyl CoA:ACP acyltransferase (mitochondrial) (MCAT), mRNA
HLP	human	malonyl CoA:ACP acyltra	MCAT	AAH42195.2	Malonyl CoA:ACP acyltransferase (mitochondrial) [Homo sapiens]	BC042195.1	Homo sapiens malonyl CoA:ACP acyltransferase (mitochondrial), mRNA (cDNA clone MGC:47838 IMAGE:6052380), complete cds
HLP	gallus	malonyl CoA:ACP acyltra	MCAT	XP_015146784.1	PREDICTED: malonyl-CoA-acyl carrier protein transacylase, mitochondrial [Gallus gallus]	XM_015291298.1	PREDICTED: Gallus gallus malonyl CoA:ACP acyltransferase (mitochondrial) (MCAT), mRNA
HLP	alligator	malonyl CoA:ACP acyltra	MCAT	KYO45338.1	malonyl-CoA-acyl carrier protein transacylase, mitochondrial [Alligator mississippiensis]	XM_019481712.1	PREDICTED: Alligator mississippiensis malonyl-CoA-acyl carrier protein transacylase (MCAT), mRNA
HLP	canna	monoacylglycerol-3-phos	ABHD5	XP_008502627.1	PREDICTED: 1-acylglycerol-3-phosphate O-acyltransferase ABHD5 [Calypte anna]	XM_008504405.1	PREDICTED: Calypte anna abhydrolase domain containing 5 (ABHD5), mRNA
HLP	swift	monoacylglycerol-3-phos	ABHD5	XP_010005913.1	PREDICTED: 1-acylglycerol-3-phosphate O-acyltransferase ABHD5 [Chaetura pelagica]	XM_010007611.1	PREDICTED: Chaetura pelagica abhydrolase domain containing 5 (ABHD5), mRNA
HLP	human	monoacylglycerol-3-phos	ABHD5	NP_057090.2	1-acylglycerol-3-phosphate O-acyltransferase ABHD5 [Homo sapiens]	Y09565.1	H.sapiens mRNA for 1-acylglycerol-3-phosphate O-acyltransferase
HLP	gallus	monoacylglycerol-3-phos	ABHD5	NP_001265074.1	1-acylglycerol-3-phosphate O-acyltransferase ABHD5 [Gallus gallus]	HQ896422.1	Gallus gallus alpha/beta hydrolase domain-containing protein 5 (ABHD5) mRNA, complete cds
HLP	alligator	monoacylglycerol-3-phos	ABHD5	KYO26977.1	1-acylglycerol-3-phosphate O-acyltransferase ABHD5 [Alligator mississippiensis]	XM_014603193.2	PREDICTED: Alligator mississippiensis abhydrolase domain containing 5 (ABHD5), transcript variant X1, mRNA
HLP	canna	lipin1	LPIN1	XP_008493249.1	PREDICTED: phosphatidate phosphatase LPIN1 isoform X1 [Calypte anna]	XM_008495027.1	PREDICTED: Calypte anna lipin 1 (LPIN1), transcript variant X1, mRNA
HLP	swift	lipin1	LPIN1	XP_010000011.1	PREDICTED: phosphatidate phosphatase LPIN1 isoform X1 [Chaetura pelagica]	XM_010001709.1	PREDICTED: Chaetura pelagica lipin 1 (LPIN1), transcript variant
HLP	human	lipin1	LPIN1	NP_663731.1	phosphatidate phosphatase LPIN1 isoform 1 [Homo sapiens]	NM_145693.2	Homo sapiens lipin 1 (LPIN1), transcript variant 1, mRNA
HLP	gallus	lipin1	LPIN1	XM_015276089.1	PREDICTED: Gallus gallus lipin 1 (LPIN1), transcript variant X1, mRNA	XP_015131575.1	PREDICTED: phosphatidate phosphatase LPIN1 isoform X1 [Gallus gallus]
HLP	alligator	lipin1	LPIN1	XP_006258935.1	PREDICTED: phosphatidate phosphatase LPIN1 isoform X1 [Alligator mississippiensis]	XM_006258873.3	PREDICTED: Alligator mississippiensis lipin 1 (LPIN1), transcript variant X1, mRNA

Supplemental Table 5. Amino acid and mRNA NCBI accession numbers of hepatic lipogenic enzymes for use in conservation and alignment comparisons.

			dN/dS nucleotide CDS					Protein alignment identity					Tx abundance: *A. colubris*	
**Pathway**	**Enzyme**	**Gene sym**	***C. anna***	***C. pelagica***	***G. gallus***	***H. sapiens***	***A. mississippiensis***	***C. anna***	***C. pelagica***	***G. gallus***	***H. sapiens***	***A. mississippiensis***	**Raw counts**	**log2(RP10K)**
HLP	Acetyl-CoA carboxylase	ACACA	0.1365	0.0354	0.0281	0.0252	0.0325	98.6	96.9	94.7	88.7	92.1	501	5.78
HLP	malonyl CoA:ACP acyltransferase (mitochondrial)	MCAT	1.0671	1.0193	0.119	0.1807	0.2371	74	68.6	80.4	61	74.5	3	0.40
HLP	fatty acid synthase	FASN	0.0643	0.215	0.1489	0.6633	0.1802	99.1	89.5	89.5	65.6	73.5	500	5.78
HLP	glycerol-3-phosphate acyltransferase 1 (mitochondrial)	GPAM	0.0371	0.1163	0.1019	0.0681	0.0857	99.9	95.3	94.1	80.6	84.6	235	4.72
HLP	monoacylglycerol-3-phosphate acyltransferase alpha	ABHD5	1.1147	1.7631	0.0923	1.2777	1.3026	90.8	88.9	87.9	74.7	83.4	19	1.61
HLP	phosphatidate acid phosphatase	LPIN1	0.1141	0.1267	0.1378	0.0792	0.1325	95.9	88.6	82	74.8	80.4	62	2.94
HLP	diacylglycerol acyltransferase	DGAT2	1.5459	0.2667	0.2947	2.0057	0.2045	99	93	96	85	87	76	3.20

Supplemental table 6. Conservation score (dN/dS) and protein alignment identity (Clustal %) given for key enzymes in the hepatic lipogenic pathway for *Calypte anna* (Anna's hummingbird), *Chaetura pelagica* (chimney swift), *Gallus gallus* (chicken), *Homo sapiens* and *Alligator mississippiensis* when compared against ruby-throated hummingbird *Archilochis colubris* sequences. Transcript abundance (in both raw and transformed counts) also given for *A. colubris* show relative abundance of featured enzymes.

Supplemental data 1. Alignments of amino acid sequence for hepatic lipogenic enzymes for compared species [canna= *Calypte anna*, swift = *Chaetura pelagica*, gallus= *Gallus gallus*, alligator= *Alligator mississippiensis*, human= *Homo sapiens*].

```
Reference sequence (1): ruby_abhd5_i6_HQ_rubyallcluste
Identities normalized by aligned length.
Colored by: identity +property


                                         1 [         .         .         .         .         :
.         .         . 80
1 ruby_abhd5_i6_HQ_rubyallcluste 100.0%
MAGAAAVVLSFAAPPATADPPLTAPGRRAAAMAEEETSSEGLGWLFSWLPAWCPTSLLHLKEAENKMLKCIASTYNKRYV
2 canna_abhd5_XP_008502627.1      90.8%     -----DFVMVMLREDVNKEALILGLNR-----------
TWLGWLFSWLPAWCPTSLLHLKEAENKMLKCIASTYNKRYV
3 cpelagica_abhd5_XP_010005913.1  88.9%     --------------GTSCTPGRFRLDT-------------
RLGWLFSWLPAWCPTSLLHLKEAEDKMLKCITSTYNKQYV
4 hsapiens_abhd5_NP_057090.2      74.7%     ----------MAAEEEEVDSADTGERS--------------
-GWLTGWLPTWCPTSISHLKEAEEKMLKCVPCTYKKEPV
5 gallus_abhd5_NP_001265074.1     87.9%     -AGATAVALSLAALPAAAPLPAPAAAMAEEEAS----
-SERLGWLFSWLPAWCPTSLLHLKEAEDKMLKCITSTYNKRYV
6 amiss_abhd5_KYO26977.1          83.2%     -----------------SDGETASEGS--------------
-GWLSGWLPAWCPTSMPHLKDAEEKILKCITSTYSKQYV

                                        81         .         1         .         .         .
.         :         . 160
1 ruby_abhd5_i6_HQ_rubyallcluste 100.0%
YISNGNKIWTLTFSPDVSHKTPLVLLHGFGGGVGLWALNFEDLCENRTVHAFDLLGFGHSSRPQFHTDAREAENQFVESI
2 canna_abhd5_XP_008502627.1      90.8%
YISNGNKIWTLTFSPDVSHKTPLVLLHGFGGGVGLWALNFEDLCENRTVHAFDLLGFGHSSRPQFHTDAREAENQFVESI
3 cpelagica_abhd5_XP_010005913.1  88.9%
YISHGNKIWTLTFSPDLSHKTPLVLLHGFGGGVGLWALNFEDLCENRTVHAFDLLGFGRSSRPHFHTDAREAENQFVESI
4 hsapiens_abhd5_NP_057090.2      74.7%
RISNGNKIWTLKFSHNISNKTPLVLLHGFGGGLGLWALNFGDLCTNRPVYAFDLLGFGRSSRPRFDSDAEEVENQFVESI
5 gallus_abhd5_NP_001265074.1     87.9%
YLANGNKIWTLTFSPDLSRKTPLVLLHGFGGGVGMWALNFEELCENRTVHAFDLLGFGRSSRPHFDTDAREAENQFVESI
6 amiss_abhd5_KYO26977.1          83.2%
YISNGNKIWTLTFSQDLSLKTPLILLHGFGGGVGLWALNFEDLCENRTVYAIDLLGFGRSSRPHFDTDAEEAENQFVESI

                                       161         .         .         .         2         .
.         .         . 240
1 ruby_abhd5_i6_HQ_rubyallcluste 100.0%
EEWRKAMEIEKMILLGHNLGGFLAAAYSLKYPSRVKHLILVEPWGFPERPDNAEHERPIPIWIKALGAILSPFNPLAGLR
2 canna_abhd5_XP_008502627.1      90.8%
EEWRKAMEIEKMILLGHNLGGFLAAAYSLKYPSRVKHLILVEPWGFPERPDNAEHERPIPIWIKALGAILSPFNPLAGLR
3 cpelagica_abhd5_XP_010005913.1  88.9%
EEWRKVMEIEKMIILGHNLGGFLAAAYSLKYPSRVKHLILVEPWGFPERPDNAEHERPIPIWIKALGAILSPFNPLAGLR
4 hsapiens_abhd5_NP_057090.2      74.7%
EEWRCALGLDKMILLGHNLGGFLAAAYSLKYPSRVNHLILVEPWGFPERPDLADQDRPIPVWIRALGAALTPFNPLAGLR
5 gallus_abhd5_NP_001265074.1     87.9%
EEWRKEMGLEKMILLGHNLGGFLAAAYSLKYPSRVKHLILVEPWGFPERPDNAEHERPIPIWIKALGAILSPFNPLAGLR
6 amiss_abhd5_KYO26977.1          83.2%
EEWRQAVGLDKMIFLGHNLGGFLAAAYSLKYPSRVKHLILVEPWGFPERPDN-EQERPIPIWIKALGAMLSPFNPLAGLR

                                       241         :         .         .         .         .
3         .         . 320
1 ruby_abhd5_i6_HQ_rubyallcluste 100.0%
IAGPFGLSLVQRLRPDFKRKYSSMFDDNTVAEYIYHCNVQSPSGETAFKNMTIPYGWAKRPMLQRISQLDQDIPITVVYG
2 canna_abhd5_XP_008502627.1      90.8%
IAGPFGLSLVQRLRPDFKRKYSSMFDDNTVAEYIYHCNVQSPSGETAFKNMTIPYGWAKRPMLQRISQLDQDIPITVVYG
3 cpelagica_abhd5_XP_010005913.1  88.9%
IAGPFGLSLVQRLRPDFKRKYSSMFDDNTVAEYIYHCNVQSPSGETAFKNMTIPYGWAKRPMLQRISQMDRDIPITVIYG
4 hsapiens_abhd5_NP_057090.2      74.7%
IAGPFGLSLVQRLRPDFKRKYSSMFEDDTVTEYIYHCNVQTPSGETAFKNMTIPYGWAKRPMLQRIGKMHPDIPVSVIFG
5 gallus_abhd5_NP_001265074.1     87.9%
IAGPFGLSLVQRLRPDFKRKYASMFDDNTVTEYIYHCNVQSPSGETAFKNMTIPYGWAKRPMLQRIPQMDQDIPITVVYG
6 amiss_abhd5_KYO26977.1          83.2%
LAGPFGLSLVQRLRPDFKRKYSSMFDDNTVAEYIYHCNVQSPSGETAFKNMTIPYGWAKRPMLQRISLMDQDIPITVIYG

                                       321         .         .         :         .         .     ]
376
1 ruby_abhd5_i6_HQ_rubyallcluste 100.0%     ARSCIDGNSGSTIQSLRPNSYVKTIAILGAGHYVYADQPEDFNERVKDICDSVD--
2 canna_abhd5_XP_008502627.1      90.8%     ARSCIDGNSGSTIQSLRPNSYVKTIAILGAGHYVYADQPEDFNERVKDICDSVD--
3 cpelagica abhd5 XP 010005913.1  88.9%     ARSCIDGNSGSTIQSLRPNSYVKTIAILGAGHYVYADQPEDFNQKVKDICDSVD--
4 hsapiens abhd5 NP 057090.2      74.7%     ARSCIDGNSGTSIQSLRPHSYVKTIAILGAGHYVYADQPEEFNQKVKEICDTV---
5 gallus abhd5 NP 001265074.1     87.9%     ARSCIDGNSGSTIQSLRPKSYVKTIAILGAGHYVYADQPEDFNQKVKDICDSVD--
6 amiss abhd5 KYO26977.1          83.2%     ARSCIDGNSGSTIQSLRPKSYVKTIAILGAGHYVYADQPEDFNQKVKEICDSVD--
```

MView 1.60.1, Copyright © 1997-2015 Nigel P. Brown

```
Reference sequence (1): ruby_acaca_i10_HQ_rubyallclust
Identities normalised by aligned length.
Colored by: identity +property
```

```
                                              1 [         .         .         .         .         :
.         .         . 80

1 ruby_acaca_i10_HQ_rubyallclust 100.0%      ---------------------------------------------------------
---MEDSAEQ--SQEMRYHMLQ-
2 canna_acaca_XP_008489475.1      98.6%      ---------------------------------------------------------
----EDSAEQ--SQEMRYHMLQ-
3 cpelagica_acaca_XP_010007346.1  96.9%      ---------------------------------------------------------
----EDSAEQ--SQEMRYHMLQ-
4 hsapiens_acaca_NP_942131.1      88.7%
WWSTLMSILRARSFWKWISTQTVRIIRAVRAHFGGIMDEPSPLAQPLELNQHSRFIIGSVSEDNSEDEISNLVKLDLLEE
5 gallus_acaca_NP_990836.1        94.7%      -------------------------------------
EESSQPAKPLEMNPHSRFIIGSVSEDNSEDETSSLVKLDLLEE
6 amiss_acaca_XP_014463551.1      92.1%      -------------------------------------
EDSSLPAKHLELSAHSRFIIGSVSEDNSEDETSSLVKLDLLEE

                                             81           .         1         .         .         .
.         :         . 160
1 ruby_acaca_i10_HQ_rubyallclust 100.0%      ------------------------------
-RPSMSGLHLVKQGRDRKKVDVQRDFTVASPAEFVTRFGGNKVIEKVLIA
2 canna_acaca_XP_008489475.1      98.6%      ------------------------------
-RPSMSGLHLVKQGRDRKKVDVQRDFTVASPAEFVTRFGGNKVIEKVLIA
3 cpelagica_acaca_XP_010007346.1  96.9%      ------------------------------
-RPSMSGLHLVKQGRDRKKVDVQRDFTVASPAEFVTRFGGNKVIEKVLIA
4 hsapiens_acaca_NP_942131.1      88.7%
KEGSLSPASVGSDTLSDLGISSLQDGLALHIRSSMSGLHLVKQGRDRKKIDSQRDFTVASPAEFVTRFGGNKVIEKVLIA
5 gallus_acaca_NP_990836.1        94.7%
KERSLSPVSVCSDSLSDLGLPSAQDGLANHMRPSMSGLHLVKQGRDRKKVDVQRDFTVASPAEFVTRFGGNRVIEKVLIA
6 amiss_acaca_XP_014463551.1      92.1%
KESSLSPVSVCSESFSDLGLPNIQEGLASHMRPSMSGLHLVKQGRDRKKVDLQRDFTVASPAEFVTRFGGNKVIEKVLIA

                                            161           .         .         .         2         .
.         .         . 240
1 ruby_acaca_i10_HQ_rubyallclust 100.0%
NNGIAAVKCMRSIRRWSYEMFRNERAIRFVVMVTPEDLKANAEYIKMADHYVPVPGGPNNNNYANVELILDIAKRIPVQA
2 canna_acaca_XP_008489475.1      98.6%
NNGIAAVKCMRSIRRWSYEMFRNERAIRFVVMVTPEDLKANAEYIKMADHYVPVPGGPNNNNYANVELILDIAKRIPVQA
3 cpelagica_acaca_XP_010007346.1  96.9%
NNGIAAVKCMRSIRRWSYEMFRNERAIRFVVMVTPEDLKANAEYIKMADHYVPVPGGPNNNNYANVELILDIAKRIPVQA
4 hsapiens_acaca_NP_942131.1      88.7%
NNGIAAVKCMRSIRRWSYEMFRNERAIRFVVMVTPEDLKANAEYIKMADHYVPVPGGPNNNNYANVELILDIAKRIPVQA
5 gallus_acaca_NP_990836.1        94.7%
NNGIAAVKCMRSIRRWSYEMFRNERAIRFVVMVTPEDLKANAEYIKMADHYVPVPGGPNNNNYANVELILDIAKRIPVQA
6 amiss_acaca_XP_014463551.1      92.1%
NNGIAAVKCMRSIRRWSYEMFRNERAIRFVVMVTPEDLKANAEYIKMADHYVPVPGGPNNNNYANVELILDIAKRIPVQA

                                            241           :         .         .         .         .
3         .         . 320
1 ruby_acaca_i10_HQ_rubyallclust 100.0%
VWAGWGHASENPKLPELLHKNGIAFMGPPSQAMWALGDKIASSIVAQTAGIPTLPWSGSGLRVDWEENDFQKRILNVPQE
2 canna_acaca_XP_008489475.1      98.6%
VWAGWGHASENPKLPELLHKNGIAFMGPPSQAMWALGDKIASSIVAQTAGIPTLPWSGSGLRVDWEENDFQKRILNVPQE
3 cpelagica_acaca_XP_010007346.1  96.9%
VWAGWGHASENPKLPELLHKNGIAFMGPPSQAMWALGDKIASSIVAQTAGIPTLPWSGSGLRVDWQENDLQKRILNVPQE
4 hsapiens_acaca_NP_942131.1      88.7%
VWAGWGHASENPKLPELLLKNGIAFMGPPSQAMWALGDKIASSIVAQTAGIPTLPWSGSGLRVDWQENDFSKRILNVPQE
5 gallus_acaca_NP_990836.1        94.7%
VWAGWGHASENPKLPELLHKNGIAFMGPPSQAMWALGDKIASSIVAQTAGIPTLPWNGSGLRVDWQENDLQKRILNVPQE
6 amiss_acaca_XP_014463551.1      92.1%
VWAGWGHASENPKLPELLHKNGIAFMGPPSQAMWALGDKIASSIVAQTAGIPTLPWSGSGLRVDWQENDLQKRILNVPQE

                                            321           .         .         :         .         .
.         .       4 400
1 ruby_acaca_i10_HQ_rubyallclust 100.0%
LYEKGYVKDADDGLRAAEEVGYPVMIKASEGGGGKGIRKVNNADDFSNLFRQVQAEVPGSPIFVMRLAKQSRHLEVQILA
2 canna_acaca_XP_008489475.1      98.6%
LYEKGYVKDADDGLRAAEEVGYPVMIKASEGGGGKGIRKVNNADDFSNLFRQVQAEVPGSPIFVMRLAKQSRHLEVQILA
3 cpelagica_acaca_XP_010007346.1  96.9%
LYEKGYVKDADDGLRAAEEVGYPVMIKASEGGGGKGIRKVNNADDFPNLFRQVQAEVPGSPIFVMRLAKQSRHLEVQILA
4 hsapiens_acaca_NP_942131.1      88.7%
LYEKGYVKDVDDGLQAAEEVGYPVMIKASEGGGGKGIRKVNNADDFPNLFRQVQAEVPGSPIFVMRLAKQSRHLEVQILA
5 gallus_acaca_NP_990836.1        94.7%
LYEKGYVKDADDGLRAAEEVGYPVMIKASEGGGGKGIRKVNNADDFPNLFRQVQAEVPGSPIFVMRLAKQSRHLEVQILA
6 amiss_acaca_XP_014463551.1      92.1%
LYEKGYVKDVDDGLRAAEEVGYPVMIKASEGGGGKGIRKVNNADDFPNLFRQVQAEVPGSPIFVMRLAKQSRHLEVQILA

                                            401           .         .         .         .         :
.         .         . 480
1 ruby_acaca_i10_HQ_rubyallclust 100.0%
DQYGNAISLFGRDCSVQRRHQKIIEEAPASIATSTVFEHMEQCAVKLAKMVGYVSAGTVEYLYSQDGSFYFLELNPRLQV
2 canna_acaca_XP_008489475.1      98.6%
DQYGNAISLFGRDCSVQRRHQKIIEEAPASIATSAVFEHMEQCAVKLAKMVGYVSAGTVEYLYSQDGSFYFLELNPRLQV
3 cpelagica_acaca_XP_010007346.1  96.9%
DQYGNAISLFGRDCSVQRRHQKIIEEAPASIATSVVFEHMEQCAVKLAKMVGYVSAGTVEYLYSQDGSFYFLELNPRLQV
```

```
4 hsapiens_acaca_NP_942131.1      88.7%
DQYGNAISLFGRDCSVQRRHQKIIEEAPATIATPAVFEHMEQCAVKLAKMVGYVSAGTVEYLYSQDGSFYFLELNPRLQV
5 gallus_acaca_NP_990836.1        94.7%
DQYGNAISLFGRDCSVQRRHQKIIEEAPASIATSVVFEHMEQCAVKLAKMVGYVSAGTVEYLYSQDGSFYFLELNPRLQV
6 amiss_acaca_XP_014463551.1      92.1%
DQYGNAISLFGRDCSVQRRHQKIIEEAPASIATIAVFEHMEQCAVKLAKMVGYVSAGTVEYLYSQDGSFYFLELNPRLQV

                                          481         .         5         .         .         .
.         :         . 560
1 ruby_acaca_i10_HQ_rubyallclust 100.0%
EHPCTEMVADVNLPAAQLQIAMGIPLHRIKDIRVMYGVSPWGDGDIDFENSAHVPSPRGHVIAARITSENPDEGFKPSSG
2 canna_acaca_XP_008489475.1      98.6%
EHPCTEMVADVNLPAAQLQIAMGIPLHRIKDIRVMYGVSPWGDGDIDFENSAHVPSPRGHVIAARITSENPDEGFKPSSG
3 cpelagica_acaca_XP_010007346.1  96.9%
EHPCTEMVADVNLPAAQLQIAMGIPLHRIKDIRVMYGVSPWGDTSIDFENSAHVPSPRGHVIAARITSENPDEGFKPSSG
4 hsapiens_acaca_NP_942131.1      88.7%
EHPCTEMVADVNLPAAQLQIAMGIPLYRIKDIRMMYGVSPWGDSPIDFEDSAHVPCPRGHVIAARITSENPDEGFKPSSG
5 gallus_acaca_NP_990836.1        94.7%
EHPCTEMVADVNLPAAQLQIAMGIPLHRIKDIRVMYGVSPWGDGSIDFENSAHVPCPRGHVIAARITSENPDEGFKPSSG
6 amiss_acaca_XP_014463551.1      92.1%
EHPCTEMVADVNLPAAQLQIAMGIPLHRIKDIRVLYSASPWGDTPVDFENSAHVPSPRGHVIAARITSENPDEGFKPSSG

                                          561         .         .         .         6         .
.         .         . 640
1 ruby_acaca_i10_HQ_rubyallclust 100.0%
TVQELNFRSNKNVWGYFSVAAAGGLHEFADSQFGHCFSWGENREEAISNMVVALKELSIRGDFRTTVEYLIKLLETESFQ
2 canna_acaca_XP_008489475.1      98.6%
TVQELNFRSNKNVWGYFSVAAAGGLHEFADSQFGHCFSWGENREEAISNMVVALKELSIRGDFRTTVEYLIKLLETESFQ
3 cpelagica_acaca_XP_010007346.1  96.9%
TVQELNFRSNKNVWGYFSVAAAGGLHEFADSQFGHCFSWGENREEAISNMVVALKELSIRGDFRTTVEYLIKLLETESFQ
4 hsapiens_acaca_NP_942131.1      88.7%
TVQELNFRSNKNVWGYFSVAAAGGLHEFADSQFGHCFSWGENREEAISNMVVALKELSIRGDFRTTVEYLIKLLETESFQ
5 gallus_acaca_NP_990836.1        94.7%
TVQELNFRSNKNVWGYFSVAAAGGLHEFADSQFGHCFSWGENREEAISNMVVALKELSIRGDFRTTVEYLIKLLETESFQ
6 amiss_acaca_XP_014463551.1      92.1%
TVQELNFRSNKNVWGYFSVAAAGGLHEFADSQFGHCFSWGENREEAISNMVVALKELSIRGDFRTTVEYLIKLLETESFQ

                                          641         :         .         .         .         .
7         .         . 720
1 ruby_acaca_i10_HQ_rubyallclust 100.0%
QNRIDTGWLDRLIAEKVQAERPDTILGVVCGALHVADVNFRNSVSNFLHSLERGQVLPAHTLLNTVDVELIYEGRKYVLK
2 canna_acaca_XP_008489475.1      98.6%
QNRIDTGWLDRLIAEKVQAERPDTILGVVCGALHVADVNFRNSVSNFLHSLERGQVLPAHTLLNTVDVELIYEGRKYVLK
3 cpelagica_acaca_XP_010007346.1  96.9%
QNRIDTGWLDRLIAEKVQAERPDTMLGVVCGALHVADVSLRNSVSNFLHSLERGQVLPAHTLLNTVDVELIYEGRKYVLK
4 hsapiens_acaca_NP_942131.1      88.7%
MNRIDTGWLDRLIAEKVQAERPDTMLGVVCGALHVADVSLRNSVSNFLHSLERGQVLPAHTLLNTVDVELIYEGVKYVLK
5 gallus_acaca_NP_990836.1        94.7%
QNRIDTGWLDRLIAEKVQAERPDTMLGVVCGALHVADVSFRNSVSNFLHSLERGQVLPAHTLLNTVDVELIYEGRKYVLK
6 amiss_acaca_XP_014463551.1      92.1%
HNRIDTGWLDRLIAEKVQAERPDTMLGVVCGALHVADVSFRNSVSNFLHSLERGQVLPAHTLLNTVDVELIYEGRKYVLK

                                          721         .         .         :         .         .
.         .         8 800
1 ruby_acaca_i10_HQ_rubyallclust 100.0%
VTRQSPNSYVVIMNNSCVEVDVHRLSDGGLLLSYDGSSYTTYMKEEVDRYRITIGNKTCVFEKENDPSILRSPSAGKLIQ
2 canna_acaca_XP_008489475.1      98.6%
VTRQSPNSYVVIMNNSCVEVDVHRLSDGGLLLSYDGSSYTTYMKEEVDRYRITIGNKTCVFEKENDPSILRSPSAGKLIQ
3 cpelagica_acaca_XP_010007346.1  96.9%
VTRQSPNSYVVIMNNSCVEVDVHRLSDGGLLLSYDGSSYTTYMKEEVDRYRITIGNKTCVFEKENDPSILRSPSAGKLIQ
4 hsapiens_acaca_NP_942131.1      88.7%
VTRQSPNSYVVIMNGSCVEVDVHRLSDGGLLLSYDGSSYTTYMKEEVDRYRITIGNKTCVFEKENDPSVMRSPSAGKLIQ
5 gallus_acaca_NP_990836.1        94.7%
VTRQSPNSYVVIMNSSCVEVDVHRLSDGGLLLSYDGSSYTTYMKEEVDRYRITIGNKTCVFEKENDPSILRSPSAGKLIQ
6 amiss_acaca_XP_014463551.1      92.1%
VTRQSPNSYVVIMNNSCVEVDVHRLSDGGLLLSYDGSSYTTYMKEEVDRYRITIGNKTCVFEKENDPSILRSPSAGKLIQ

                                          801         .         .         .         .         :
.         .         . 880
1 ruby_acaca_i10_HQ_rubyallclust 100.0%
YVVEDGGHVFAGQCFAEIEVMKMVMTLTAGESGCIHYVKRPGAVLDPGCVIAKLQLDDPS----RVQQAELHTGALPQIQ
2 canna_acaca_XP_008489475.1      98.6%
YVVEDGGHVFAGQCFAEIEVMKMVMTLTAGESGCIHYVKRPGAVLDPGCVIAKLQLDAAS----RVQQAELHTGALPQIQ
3 cpelagica_acaca_XP_010007346.1  96.9%
YVVEDGGHVFSGQCFAEIEVMKMVMTLTAGESGCIHYVKRPGAVLDPGCVIAKLQLDDPSRVQQKIQQAELHTGALPQIQ
4 hsapiens_acaca_NP_942131.1      88.7%
YIVEDGGHVFAGQCYAEIEVMKMVMTLTAVESGCIHYVKRPGAALDPGCVLAKMQLDNPS----KVQQAELHTGSLPRIQ
5 gallus_acaca_NP_990836.1        94.7%
YVVEDGGHVFAGQCFAEIEVMKMVMTLTAGESGCIHYVKRPGAVLDPGCVIAKLQLDDPS----RVQQAELHTGTLPQIQ
6 amiss_acaca_XP_014463551.1      92.1%
YVVEDGGHVFAGQCFAEIEVMKMVMTLTAAESGCIHYVKRPGAALDPGCVIAKLQLDDPS----RVQQAELHTGTLPKIQ

                                          881         .         9         .         .         .
.         :         . 960
1 ruby_acaca_i10_HQ_rubyallclust 100.0%
```

```
STALRGEKLHRIFHYVLDNLVNVMNGYCLPEPFFSSKVKGWVERLMKTLRDPSLPLLELQDIMTSVSGRIPPNVEKSIKK
2 canna_acaca_XP_008489475.1      98.6%
STALRGEKLHRIFHYVLDNLVNVMNGYCLPEPFFSSKVKGWVERLMKTLRDPSLPLLELQDIMTSVSGRIPPNVEKSIKK
3 cpelagica_acaca_XP_010007346.1  96.9%
STALRGEKLHRIFHYVLDNLVNVMNGYCLPEPYFSSKVKGWVERLMKTLRDPSLPLLELQDIMTSVSGRIPPNVEKSIKK
4 hsapiens_acaca_NP_942131.1      88.7%
STALRGEKLHRVFHYVLDNLVNVMNGYCLPDPFFSSKVKDWVERLMKTLRDPSLPLLELQDIMTSVSGRIPPNVEKSIKK
5 gallus_acaca_NP_990836.1        94.7%
STALRGEKLHRIFHYVLDNLVNVMNGYCLPEPYFSSKVKGWVERLMKTLRDPSLPLLELQDIMTSVSGRIPPNVEKSIKK
6 amiss_acaca_XP_014463551.1      92.1%
STALRGEKLHRVFHYVLDNLVNVMNGYCLPEPYFGRKVKDWVERLMKTLRDPSLPLLELQDIMTSVSGRIPPNVEKSIKK

                                                961       .         .         .         0         .
.         .         . 1040
1 ruby_acaca_i10_HQ_rubyallclust 100.0%
EMAQYASNITSVLCQFPSQQIANILDSHAATLNRKSEREVFFMNTQSIVQLVQRYRSGIRGHMKAVVMDLLRQYLKVETQ
2 canna_acaca_XP_008489475.1      98.6%
EMAQYASNITSVLCQFPSQQIANILDSHAATLNRKSEREVFFMNTQSIVQLVQRYRSGIRGHMKAVVMDLLRQYLKVETQ
3 cpelagica_acaca_XP_010007346.1  96.9%
EMAQYASNITSVLCQFPSQQIANILDSHAATLNRKSEREVFFMNTQSIVQLVQRYRSGIRGHMKAVVMDLLRQYLKVETQ
4 hsapiens_acaca_NP_942131.1      88.7%
EMAQYASNITSVLCQFPSQQIANILDSHAATLNRKSEREVFFMNTQSIVQLVQRYRSGIRGHMKAVVMDLLRQYLRVETQ
5 gallus_acaca_NP_990836.1        94.7%
EMAQYASNITSVLCQFPSQQIANILDSHAATLNRKSEREVFFMNTQSIVQLVQRYRSGIRGHMKAVVMDLLRQYLKVETQ
6 amiss_acaca_XP_014463551.1      92.1%
EMAQYASNITSVLCQFPSQQIANILDSHAATLNRKSEREVFFMNTQSIVQLVQRYRSGIRGHMKAVVMDLLRQYLKVETQ

                                               1041       :         .         .         .         .
1         .         . 1120
1 ruby_acaca_i10_HQ_rubyallclust 100.0%
FQHGHYDKCVFTLREENKSDMNAVLNYIFSHAQVTKKNLLVTMLIDQLCGRDPTLTDELINILTELTQLSKTTNAKVALR
2 canna_acaca_XP_008489475.1      98.6%
FQHGHYDKCVFTLREENKSDMNAVLNYIFSHAQVTKKNLLVTMLIDQLCGRDPTLTDELINILTELTQLSKTTNAKVALR
3 cpelagica_acaca_XP_010007346.1  96.9%
FQHGHYDKCVFTLREENKSDMNAVLNYIFSHAQVTKKNLLVTMLIDQLCGRDPTLTDELINILTELTQLSKTTNAKVALR
4 hsapiens_acaca_NP_942131.1      88.7%
FQNGHYDKCVFALREENKSDMNTVLNYIFSHAQVTKKNLLVTMLIDQLCGRDPTLTDELLNILTELTQLSKTTNAKVALR
5 gallus_acaca_NP_990836.1        94.7%
FQHGHYDKCVFALREENKSDMNAVLNYIFSHAQVTKKNLLVTMLIDQLCGRDPTLTDELINILTELTQLSKTTNAKVALR
6 amiss_acaca_XP_014463551.1      92.1%
FQHGHYDKCVFALREENKSDMNTVLNYIFSHAQVTKKNLLVTMLIDQLCGRDPTLTDELINILTELTQLSKTTNAKVALR

                                               1121       .         .         :         .         .
.         .         2 1200
1 ruby_acaca_i10_HQ_rubyallclust 100.0%
ARQVLIASHLPSYELRHNQVESIFLSAIDMYGHQFCIENLQKLILSETSIFDVLPNFFYHSNQVVRMAALEVYVRRAYIA
2 canna_acaca_XP_008489475.1      98.6%
ARQVLIASHLPSYELRHNQVESIFLSAIDMYGHQFCIENLQKLILSETSIFDVLPNFFYHSNQVVRMAALEVYVRRAYIA
3 cpelagica_acaca_XP_010007346.1  96.9%
ARQVLIASHLPSYELRHNQVESIFLSAIDMYGHQFCIENLQKLILSETSIFDVLPNFFYHSNQVVRMAALEVYVRRAYIA
4 hsapiens_acaca_NP_942131.1      88.7%
ARQVLIASHLPSYELRHNQVESIFLSAIDMYGHQFCIENLQKLILSETSIFDVLPNFFYHSNQVVRMAALEVYVRRAYIA
5 gallus_acaca_NP_990836.1        94.7%
ARQVLIASHLPSYELRHNQVESIFLSAIDMYGHQFCIENLQKLILSETSIFDVLPNFFYHSNQVVRMAALEVYVRRAYIA
6 amiss_acaca_XP_014463551.1      92.1%
ARQVLIASHLPSYELRHNQVESIFLSAIDMYGHQFCIENLQKLILSETSIFDVLPNFFYHSNQVVRMAALEVYVRRAYIA

                                               1201       .         .         .         .         :
.         .         . 1280
1 ruby_acaca_i10_HQ_rubyallclust 100.0%      YELNSVQHRQLKDNTCVVEFQFMLPTSHPN-------
-RMSFSSNLNHYGMVHVASVSDVLLDNSFTPPCQRMGGMVSFR
2 canna_acaca_XP_008489475.1      98.6%
YELNSVQHRQLKDNTCVVEFQFMLPTSHPNRGNIPTLNRMSFSSNLNHYGMVHVASVSDVLLDNSFTPPCQRMGGMVSFR
3 cpelagica_acaca_XP_010007346.1  96.9%
YELNSVQHRQLKDNTCVVEFQFMLPTSHPNRGNIPTLNRMSFSSNLNHYGMVHVASVSDVLLDNSFTPPCQRMGGMVSFR
4 hsapiens_acaca_NP_942131.1      88.7%
YELNSVQHRQLKDNTCVVEFQFMLPTSHPNRGNIPTLNRMSFSSNLNHYGMTHVASVSDVLLDNSFTPPCQRMGGMVSFR
5 gallus_acaca_NP_990836.1        94.7%      YELNSVQHRQLKDNTCVVEFQFMLPTSHPN-------
-RMSFSSNLNHYGMVHVASVSDVLLDNSFTPPCQRMGGMVSFR
6 amiss_acaca_XP_014463551.1      92.1%      YELNSVQHRQLKDNTCVVEFQFMLPTSHPN-------
-RMSFSSNLNHYGMVHVASVSDVLLDNSFTPPCQRMGGMVSFR

                                               1281       .         3         .         .         .
.         :         . 1360
1 ruby_acaca_i10_HQ_rubyallclust 100.0%
TFEDFVRIFDEVMGCFCDSPPQSPTFPEAGHASLYDEDKTSREEPIHILNVAIKTDSDVDDDGLAAMFREFTQSKKSVLF
2 canna_acaca_XP_008489475.1      98.6%
TFEDFVRIFDEVMGCFCDSPPQSPTFPEAGHASLYDEDKTSREEPIHILNVAIKTDSDVDDDGLAAMFREFTQSKKSVLF
3 cpelagica_acaca_XP_010007346.1  96.9%
TFEDFVRIFDEVMGCFCDSPPQSPTFPEAGHASLYDEDKAPREEPIHILNVAIKTDSDVDDDGLAAMFREFTQSKKSVLI
4 hsapiens_acaca_NP_942131.1      88.7%
TFEDFVRIFDEVMGCFSDSPPQSPTFPEAGHTSLYDEDKVPRDEPIHILNVAIKTDCDIEDDRLAAMFREFTQQNKATLV
5 gallus_acaca_NP_990836.1        94.7%
TFEDFVRIFDEVMSCFCDSPPQSPTFPEAGHASLYDEDKAAREEPIHILNVAIKTDGDVDDDGLAAMFREFTQSKKSVLI
6 amiss_acaca_XP_014463551.1      92.1%
```

MView

TFDDFVRIFDEVMGCFCDSPPQSPTFREASRTSLYDEDKVAREEPIHILNVAIKTDSDIDDDGLAAMFREFTQSKKSILI

```
                                                  1361           .           .           .           4           .
.           .           . 1440
1 ruby_acaca_i10_HQ_rubyallclust 100.0%      EHGIRRLTFLVAQK--------------
-REFPKFFTFRARDKFEEDRIYRHLEPALAFQLELNRMRNFDLTAIPCANHK
2 canna_acaca_XP_008489475.1       98.6%
EHGIRRLTFLVAQKDFRKQVNYEVDQRFHREFPKFFTFRARDKFEEDRIYRHLEPALAFQLELKXXXXFPLTAIPCANHK
3 cpelagica_acaca_XP_010007346.1  96.9%
EHGIRRLTFLVAQKDFRKQVNYEVDQRFHREFPKFFTFRARDKFEEDRIYRHLEPALAFQLELNRMRNFDLTAIPCANHK
4 hsapiens_acaca_NP_942131.1       88.7%
DHGIRRLTFLVAQKDFRKQVNYEVDRRFHREFPKFFTFRARDKFEEDRIYRHLEPALAFQLELNRMRNFDLTAIPCANHK
5 gallus_acaca_NP_990836.1         94.7%      EHGIRRLTFLVAQK--------------
-REFPKFFTFRARDKFEEDRIYRHLEPALAFQLELNRMRNFDLTAIPCANHK
6 amiss_acaca_XP_014463551.1       92.1%
EHGIRRLTFLVAQKDFRKQVNYEVDQRFHREFPKFFTFRARDKFEEDRIYRHLEPALAFQLELNRMRNFDLTAIPCANHK

                                                  1441           :           .           .           .           .
5           .           . 1520
1 ruby_acaca_i10_HQ_rubyallclust 100.0%
MHLYLGAAKVEVGTEVTDYRFFVRAIIRHSDLVTKEASFEYLQNEGERLLLEAMDELEVAFNNTNVRTDCNHIFLNFVPT
2 canna_acaca_XP_008489475.1       98.6%
MHLYLGAAKVEVGTEVTDYRFFVRAIIRHSDLVTKEASFEYLQNEGERLLLEAMDELEVAFNNTNVRTDCNHIFLNFVPT
3 cpelagica_acaca_XP_010007346.1  96.9%
MHLYLGAAKVEVGTEVTDYRFFVRAIIRHSDLVTKEASFEYLQNEGERLLLEAMDELEVAFNNTNVRTDCNHIFLNFVPT
4 hsapiens_acaca_NP_942131.1       88.7%
MHLYLGAAKVEVGTEVTDYRFFVRAIIRHSDLVTKEASFEYLQNEGERLLLEAMDELEVAFNNTNVRTDCNHIFLNFVPT
5 gallus_acaca_NP_990836.1         94.7%
MHLYLGAAKVEVGTEVTDYRFFVRAIIRHSDLVTKEASFEYLQNEGERLLLEAMDELEVAFNNTNVRTDCNHIFLNFVPT
6 amiss_acaca_XP_014463551.1       92.1%
MHLYLGAAKVEVGTEVTDYRFFVRAIIRHSDLVTKEASFEYLQNEGERLLLEAMDELEVAFNNTNVRTDCNHIFLNFVPT

                                                  1521           .           .           :           .           .
.           .           6 1600
1 ruby_acaca_i10_HQ_rubyallclust 100.0%
VIMDPSKIEESVRSMVMRYGSRLWKLRVLQAELKINIRLTPTGKAIPIRLFLTNESGYYLDISLYKEVTDSRTGQIMFQA
2 canna_acaca_XP_008489475.1       98.6%
VIMDPSKIEESVRSMVMRYGSRLWKLRVLQAELKINIRLTPTGKAIPIRLFLTNESGYYLDISLYKEVTDSRTGQIMFQA
3 cpelagica_acaca_XP_010007346.1  96.9%
VIMDPSKIEESVRSMVMRYGSRLWKLRVLQAELKINIRLTPTGKAIPIRLFLTNESGYYLDISLYKEVTDSRTGQIMFQA
4 hsapiens_acaca_NP_942131.1       88.7%
VIMDPSKIEESVRSMVMRYGSRLWKLRVLQAELKINIRLTPTGKAIPIRLFLTNESGYYLDISLYKEVTDSRTAQIMFQA
5 gallus_acaca_NP_990836.1         94.7%
VIMDPSKIEESVRSMVMRYGSRLWKLRVLQAELKINIRLTPTGKAIPIRLFLTNESGYYLDISLYKEVTDSRTGQIMFQA
6 amiss_acaca_XP_014463551.1       92.1%
VIMDPSKIEESVRSMVMRYGSRLWKLRVLQAELKINIRLTPTGKAIPIRLFLTNESGYYLDISLYKEVTDSRTAQIMFQA

                                                  1601           .           .           .           .           :
.           .           . 1680
1 ruby_acaca_i10_HQ_rubyallclust 100.0%
YGDKQGPLHGMLINTPYVTKDLLQSKRFQAQSLGTSYVYDIPEMFRQSLIKLWGHMNDHAFLPTPPLPSDILTYTELVLD
2 canna_acaca_XP_008489475.1       98.6%
YGDKQGPLHGMLINTPYVTKDLLQSKRFQAQSLGTSYVYDIPEMFRQSLIKLWGHMNDHAFLPTPPLPSDILTYTELVLD
3 cpelagica_acaca_XP_010007346.1  96.9%
YGDKQGPLHGMLINTPYVTKDLLQSKRFQAQSLGTSYVYDIPEMFRQSLIKLWESMNEHAFLPTPPLPSDILTYTELVLD
4 hsapiens_acaca_NP_942131.1       88.7%
YGDKQGPLHGMLINTPYVTKDLLQSKRFQAQSLGTTYIYDIPEMFRQSLIKLWESMSTQAFLPSPPLPSDMLTYTELVLD
5 gallus_acaca_NP_990836.1         94.7%
YGDKQGPLHGMLINTPYVTKDLLQSKRFQAQSLGTSYVYDIPEMFRQSLIKLWDSMNEHAFLPTPPLPSDILTYTELVLD
6 amiss_acaca_XP_014463551.1       92.1%
YGDKQGPLHGMLINTPYVTKDLLQSKRFQAQSLGTTYVYDIPEMFRQSLIKLWDSMNECAVLSAPPLPSDMLTYTELVLD

                                                  1681           .           7           .           .           .
.           :           . 1760
1 ruby_acaca_i10_HQ_rubyallclust 100.0%
DQGQLVHMNRLPGGNEVGMVAWKMTLKTPEYPEGRDIIVIGNDITYKIGSFGPQEDILFLRASELARTHGIPRIYVAANS
2 canna_acaca_XP_008489475.1       98.6%
DQGQLVHMNRLPGGNEVGMVAWKMTLKTPEYPEGRDIIVIGNDITYKIGSFGPQEDILFLRASELARTHGIPRIYVAANS
3 cpelagica_acaca_XP_010007346.1  96.9%
DQGQLVHMNRLPGGNEIGMVAWKMTLKTPEYPEGRDIIVIGNDITYRIGSFGPQEDVLYLRASELARTQGIPRVYVAANS
4 hsapiens_acaca_NP_942131.1       88.7%
DQGQLVHMNRLPGGNEIGMVAWKMTFKSPEYPEGRDIIVIGNDITYRIGSFGPQEDLLFLRASELARAEGIPRIYVSANS
5 gallus_acaca_NP_990836.1         94.7%
DQGQLVHMNRLPGGNEIGMVAWKMTLKTPEYPEGRDIIVIGNDITYRIGSFGPQEDVLFLRASELARTHGIPRIYVAANS
6 amiss_acaca_XP_014463551.1       92.1%
DQGQLVHMNRLPGGNEIGMVAWKMTLKSPEYPEGRDIIVIGNDITYRIGSFGPQEDLLYLRASELARTQGIPRIYVAANS

                                                  1761           .           .           .           8           .
.           .           . 1840
1 ruby_acaca_i10_HQ_rubyallclust 100.0%
GARIGLAEEIRHMFHVAWEDPEDPYKGYKYLYLTPQDYKKVSALNSVHCEHVEDSGESRYKITDIIGKEDGLGIENLRGS
2 canna_acaca_XP_008489475.1       98.6%
GARIGLAEEIRHMFHVAWEDPEDPYKGYKYLYLTPQDYKKVSALNSVHCEHVEDSGESRYKITDIIGKEDGLGIENLRGS
3 cpelagica_acaca_XP_010007346.1  96.9%
GARIGLAEEIRHMFHVAWEDPDDPYKGYKYLYLTPQDYKKVSALNSVHCEHVEENGESRYKITDIIGKEDGLGIENLRGS
```

```
4 hsapiens_acaca_NP_942131.1      88.7%
GARIGLAEEIRHMFHVAWVDPEDPYKGYRYLYLTPQDYKRVSALNSVHCEHVEDEGESRYKITDIIGKEEGIGPENLRGS
5 gallus_acaca_NP_990836.1        94.7%
GARIGLAEEIRHMFHVAWEDPDDPYKGYKYLYLTPQDYKKVSALNSVHCEHVEDNGESRYKITDIIGKEDGLGIENLRGS
6 amiss_acaca_XP_014463551.1      92.1%
GARIGLAEEIRHMFHVAWVEPDDPYKGYKYLYLTPQDYKKVSALNSVHCEHVEDEGESRYKITDIIGKEEGLGVENLRGS

                                  1841          :         .         .         .         .
9         .         . 1920
1 ruby_acaca_i10_HQ_rubyallclust 100.0%
GMIAGETSLAYDSIITINLVTCRAIGIGAYLVRLGQRTLQVENSHIILTGCGALNKVLGREVYTSNNQLGGIQIMHNNGV
2 canna_acaca_XP_008489475.1      98.6%
GMIAGETSLAYDSIITINLVTCRAIGIGAYLVRLGQRTLQVENSHIILTGCGALNKVLGREVYTSNNQLGGIQIMHNNGV
3 cpelagica_acaca_XP_010007346.1  96.9%
GMIAGETSLAYDSIITINLVTCRAIGIGAYLVRLGQRTIQVENSHIILTGCGALNKVLGREVYTSNNQLGGIQIMHNNGV
4 hsapiens_acaca_NP_942131.1      88.7%
GMIAGESSLAYNEIITISLVTCRAIGIGAYLVRLGQRTIQVENSHLILTGAGALNKVLGREVYTSNNQLGGIQIMHNNGV
5 gallus_acaca_NP_990836.1        94.7%
GMIAGESSLAYESIITINLVTCRAIGIGAYLVRLGQRTIQVENSHIILTGCGALNKVLGREVYTSNNQLGGIQIMHNNGV
6 amiss_acaca_XP_014463551.1      92.1%
GMIAGESSLAYDSIITISLVTCRAIGIGAYLVRLGQRTIQVENSHIILTGAGALNKVLGREVYTSNNQLGGIQIMHNNGV

                                  1921          .         .         :         .         .
.         .          0 2000
1 ruby_acaca_i10_HQ_rubyallclust 100.0%
THNTVCDDFEGVYTVLQWLSYMPKSVSSPVPILKVKDPIDRTIDFVPTKAPYDPRWMLAGRPNPSQKGQWLSGFFDNGSF
2 canna_acaca_XP_008489475.1      98.6%
THNTVCDDFEGVYTVLQWLSYMPKSVSSPVPILKVKDPIDRTIDFVPTKAPYDPRWMLAGRPNPSQKGQWLSGFFDNGSF
3 cpelagica_acaca_XP_010007346.1  96.9%
THSTVCDDFEGVYTVLQWISYMPKNVSSPVPILKVKDPIDRTIDFVPTKTPYDPRWMLAGRPNPSQKGQWLSGFFDNGSF
4 hsapiens_acaca_NP_942131.1      88.7%
THCTVCDDFEGVFTVLHWLSYMPKSVHSSVPLLNSKDPIDRIIEFVPTKTPYDPRWMLAGRPHPTQKGQWLSGFFDYGSF
5 gallus_acaca_NP_990836.1        94.7%
THGTVCDDFEGVYTILLWLSYMPKSVYSPVPILKVKDPIDRTIDFVPTKTPYDPRWMLAGRPNPSQKGQWQSGFFDNGSF
6 amiss_acaca_XP_014463551.1      92.1%
THSTVCDDFEGVFTILLWLSYMPKSVSCPVPILSAKDPIDRTIEFVPTKTPYDPRWMLAGRPHPTQKGQWLSGFFDHGSF

                                  2001          .         .         .         .         :
.         .         . 2080
1 ruby_acaca_i10_HQ_rubyallclust 100.0%
LEIMQPWAQTVVVGRARLGGIPVGVVAVETRTVELSIPADPANLDSEAKIIQQAGQVWFPDSAFKTAQAINDFNREGLPL
2 canna_acaca_XP_008489475.1      98.6%
LEIMQPWAQTVVVGRARLGGIPVGVVAVETRTVELSIPADPANLDSEAKIIQQAGQVWFPDSAFKTAQAINDFNREGLPL
3 cpelagica_acaca_XP_010007346.1  96.9%
LEIMQPWAQTVVVGRARLGGIPVGVVAVETRTVELSIPADPANLDSEAKIIQQAGQVWFPDSAFKTAQAVKDFNREGLPL
4 hsapiens_acaca_NP_942131.1      88.7%
SEIMQPWAQTVVVGRARLGGIPVGVVAVETRTVELSIPADPANLDSEAKIIQQAGQVWFPDSAFKTYQAIKDFNREGLPL
5 gallus_acaca_NP_990836.1        94.7%
LEIMQPWAQTVVVGRARLGGIPVGVVAVETRTVELSIPADPANLDSEAKIIQQAGQVWFPDSAFKTAQAINDFNREGLPL
6 amiss_acaca_XP_014463551.1      92.1%
LEVMQPWAQTVVVGRARLGGIPVGVVAVETRTVELSIPADPANLDSEAKIIQQAGQVWFPDSAFKTAQAIKDFNREGLPL

                                  2081          .         1         .         .         .
.         :         . 2160
1 ruby_acaca_i10_HQ_rubyallclust 100.0%
MVFANWRGFSGGMKDMYDQVLKFGAYIVDGLREYHQPVLIYIPPQAELRGGSWVVVDPTINPRHMEMYADRESRGGVLEP
2 canna_acaca_XP_008489475.1      98.6%
MVFANWRGFSGGMKDMYDQVLKFGAYIVDGLREYHQPVLIYIPPQAELRGGSWVVVDPTINPRHMEMYADRESRGGVLEP
3 cpelagica_acaca_XP_010007346.1  96.9%
MVFANWRGFSGGMKDMYDQVLKFGAYIVDGLREYCQPVLIYIPPQAELRGGSWVVVDPTINPRHMEMYADRESRGGILEP
4 hsapiens_acaca_NP_942131.1      88.7%
MVFANWRGFSGGMKDMYDQVLKFGAYIVDGLRECCQPVLVYIPPQAELRGGSWVVIDSSINPRHMEMYADRESRGSVLEP
5 gallus_acaca_NP_990836.1        94.7%
MVFANWRGFSGGMKDMYDQVLKFGAYIVDGLREYRQPVLIYIPPQAELRGGSWAVIDPTINPRHMEMYADRESRGGILEP
6 amiss_acaca_XP_014463551.1      92.1%
MVFANWRGFSGGMKDMYDQILKFGAYIVDGLREYRQPVIVYIPPQAELRGGSWVVIDPTINPRHMEMYADRDSRGGVLEP

                                  2161          .         .         .         2         .
.         .         . 2240
1 ruby_acaca_i10_HQ_rubyallclust 100.0%
EGTVEIKFRRKDLVKTMRRVDPVYIRLAERLGTPELSPAERKELETKLREREEFLIPMYQQVAVQFADLHDTPGRMQEKG
2 canna_acaca_XP_008489475.1      98.6%
EGTVEIKFRRKDLVKTMRRVDPVYIRLAERLGTPELSPAERKELETKLREREEFLIPMYQQVAVQFADLHDTPGRMQEKG
3 cpelagica_acaca_XP_010007346.1  96.9%
EGTVEIKFRRKDLVKTMRRVDPVYIRLAERLGTPELSAADRKDLETKLREREEFLIPIYQQVAVQFADLHDTPGRMQEKG
4 hsapiens_acaca_NP_942131.1      88.7%
EGTVEIKFRRKDLVKTMRRVDPVYIHLAERLGTPELSTAERKELENKLKEREEFLIPIYHQVAVQFADLHDTPGRMQEKG
5 gallus_acaca_NP_990836.1        94.7%
EGTVEIKFRRKDLVKTMRRVDPVYMRLAERLGTPELSAADRKDLESKLKEREEFLIPIYHQVAMQFADLHDTPGRMQEKG
6 amiss_acaca_XP_014463551.1      92.1%
EGTVEIKFRKKDLVKTMRRVDPIYIRLAERLGTPELSPAERKELETKLKEREEFLTPIYHQVAVQFADLHDTPGRMQEKA

                                  2241          :         .         .         .         .
3         .         . 2320
1 ruby_acaca_i10_HQ_rubyallclust 100.0%
```

```
AITDILDWKTSRTFFYWRLRRLLLEEMVKKKIHDANPELTDGQIQAMLRRWFVEAEGTVKAYVWDSNKDVVEWLEKQLTE
2 canna_acaca_XP_008489475.1      98.6%
AITDILDWKTSRTFFYWRLRRLLLEEMVKKKIHDANPELTDGQIQAMLRRWFVEAEGTVKAYVWDSNKDVVEWLEKQLTE
3 cpelagica_acaca_XP_010007346.1  96.9%
AITDILDWKTSRTFFYWRLRRLLLEDVVKKKIHDANPELTDGQIQAMLRRWFVEAEGTVKAYLWDSNKDLVEWLEKQLTE
4 hsapiens_acaca_NP_942131.1      88.7%
VISDILDWKTSRTFFYWRLRRLLLEDLVKKKIHNANPELTDGQIQAMLRRWFVEVEGTVKAYVWDNNKDLAEWLEKQLTE
5 gallus_acaca_NP_990836.1        94.7%
AITDILDWKTSRTFFYWRLRRLLLEDVVKKKIHDANPELTDGQIQAMLRRWFVEVEGTVKAYLWDSNKDLVEWLEKQLME
6 amiss_acaca_XP_014463551.1      92.1%
VITDILEWKTSRTFFYWRLRRLLLEDVVKKKIHDANPELTDGQIQAMLRRWFVEVEGTVKAYLWDNNKDLVEWLEKQLTE

                                        2321          .         .         :         .         .
.         ] 2389
1 ruby_acaca_i10_HQ_rubyallclust 100.0%
EEGVRSVVDENIKYISRDYILKQIRSLVQANPEVAMDSIVHMTQHISPTQRAEIVRILSTMDSPSST--
2 canna_acaca_XP_008489475.1      98.6%
EEGVRSVVDENIKYISRDYILKQIRSLVQANPEVAMDSIVHMTQHISPTQRAEIVRILSTMDSPSST--
3 cpelagica_acaca_XP_010007346.1  96.9%
EEGVRSVVDENIKYISRDYILKQIRSLVQANPEVAMDSIVHMTQHISPTQRAEIVRILSTMDSPSST--
4 hsapiens_acaca_NP_942131.1      88.7%
EDGVHSVIEENIKCISRDYVLKQIRSLVQANPEVAMDSIIHMTQHISPTQRAEVIRILSTMDSPST---
5 gallus_acaca_NP_990836.1        94.7%
EEGVRSVVDENIKYISRDYILKQIRSLVQANPEVAMDSIVHMTQHISPTQRAEIVRILSTMDSPSST--
6 amiss_acaca_XP_014463551.1      92.1%
EEGVRSVVEENIKYISRDYILKQIRSLVQANPEVAMDSIVHMTQHISPTQRAEIVRILSTMDSPPST--
```

MView 1.60.1, Copyright © 1997-2015 Nigel P. Brown

```
Reference sequence (1): ruby_dgat_i1_HQ_rubyallcluster
Identities normalised by aligned length.
Colored by: identity + property

                                          1 [         .         .         .         .         :
.         .         . 80

1 ruby_dgat_i1_HQ_rubyallcluster 100.0%     KTIIAACSQNLSG--------------------------
-SRASVQTALHTLLRAPWPSQRNLRSWMQRLAVLQWVLSFL
2 canna_dgat_XP_008493408.1       59.2%     KTIIAAYSGVLRG---------------------------TGSNILSSLQDLF--
-WLSKSKLEKQLQIISVLQWVLTFL
3 cpelagica_dgat_XP_010005498.1   60.6%     -------------------------------------------------------
-------------------------
4 hsapiens_dgat_NP_115953.2       51.4%
KTLIAAYSGVLRGERQAEADRSQRSHGGPALSREGSGRWGTGSSILSALQDLFSVTWLNRSKVEKQLQVISVLQWVLSFL
5 gallus_dgat_XP_419374.3         58.1%     KTIIAAYSGVLRG---------------------------TGSSILSALQDLF--
-WLSKSKVEKQLQIISVLQWVLTFL
6 amiss_dgat_XP_014456982.1       56.7%     KTLIAAYSGVLRG---------------------------TGSNILSALQDVF--
-WFSKSKFEKQLQIISVLQWVLSFL

                                         81         .         1         .         .         .
.         :         . 160
1 ruby_dgat_i1_HQ_rubyallcluster 100.0%
LLGIVSLLVLIYLVFTSFWAISALYLAWIIFDWDTPERGGRRLACLRGWPIWNHFRDYFPVKLVKTHELSPSHNYIIGSH
2 canna_dgat_XP_008493408.1       59.2%
VMGVACTLILMYILCTDCWAIAALYLAWLVFDWNTPKKGGRRSQWVRNWAIWRYFRDYFPIRLVKTHNLLTTRNYIFGYH
3 cpelagica_dgat_XP_010005498.1   60.6%      -
-GVACTLILMYILCTDCWAIAALYLAWLVFDWNTPKKGGRRSQWVRNWAIWRYFRDYFPIRLVKTHNLLTTRNYIFGYH
4 hsapiens_dgat_NP_115953.2       51.4%
VLGVACSAILMYIFCTDCWLIAVLYFTWLVFDWNTPKKGGRRSQWVRNWAVWRYFRDYFPIQLVKTHNLLTTRNYIFGYH
5 gallus_dgat_XP_419374.3         58.1%
IMGIACTLILMYILCTDCWAIAALYLAWLVFDWNTPKKGGRRSQWVRNWAIWRYFRDYFPIRLVKTHNLLTTRNYIFGYH
6 amiss_dgat_XP_014456982.1       56.7%
VLGVTCSIILVYILCTDCWAIAALYVAWLVFDWDTPMKGGRRSPWVRNWAMWRYFRDYFPIRLVKTHNLPTNRNYIFGYH

                                        161         .         .         .         2         .
.         .         . 240
1 ruby_dgat_i1_HQ_rubyallcluster 100.0%
PHGILCVGAFCNFVTGSTGFGQKFPGIRPSLTTLAGNFRLPLFREYLMSGGLCPVTRSAMGYLLAKNGTGNAVAIVIGGA
2 canna_dgat_XP_008493408.1       59.2%
PHGIMGLGAFCNFSTEATGVGQKFPGIRPYLATLAGNFRMPILRDYLMSGGICPVNRDSIDYILSKNGTGNAIIIVVGGA
3 cpelagica_dgat_XP_010005498.1   60.6%
PHGIMGLGAFCNFSTEATGVSQKFPGIRPYLATLAGNFRMPILRDYLMSGGICPVNRDSIDYILSKNGSGNAIIIVVGGA
4 hsapiens_dgat_NP_115953.2       51.4%
PHGIMGLGAFCNFSTEATEVSKKFPGIRPYLATLAGNFRMPVLREYLMSGGICPVSRDTIDYLLSKNGSGNAIIIVVGGA
5 gallus_dgat_XP_419374.3         58.1%
PHGIMGLGAFCNFSTEATGVSQKFPGIRPYLATLAGNFRMPILRDYLMSGGICPVNRDSIDYILSKNGSGNAIIIVVGGA
6 amiss_dgat_XP_014456982.1       56.7%
PHGIMGFGAFCNFGTEATGVSQKFPGIRPYLATLAGNFRIPILRDYLMSGGICPVNRHSIDFILSKNGTGNAVIIVVGGA

                                        241         :         .         .         .         .
3         .         . 320
```

```
 1 ruby_dgat_i1_HQ_rubyallcluster 100.0%
 AESLSCRPGVTTLILKNRKGFVRMALEHGAYLVPSFTFGENDLYHQVVFKEGSWMRSIQSCFQKLIGFAPCVFYGRALTS
 2 canna_dgat_XP_008493408.1       59.2%
 AESLNCTPGKNSVTLKNRKGFVKLALRHGADLVPVYSFGENEVYKQVIFEEGSWGRWVQKKFQKHIGFAPCIFHGRGLFS
 3 cpelagica_dgat_XP_010005498.1   60.6%
 AESLNCTPGKNSVTLKNRKGFVKLALRHGADLVPVYSFGENEVYKQVIFEEGSWGRWVQKKFQKHIGFAPCIFHGRGLFS
 4 hsapiens_dgat_NP_115953.2       51.4%
 AESLSSMPGKNAVTLRNRKGFVKLALRHGADLVPIYSFGENEVYKQVIFEEGSWGRWVQKKFQKYIGFAPCIFHGRGLFS
 5 gallus_dgat_XP_419374.3         58.1%
 AESLNCTPGKNSVTLRNRKGFVKLALRHGADLVPVYSFGENEVYKQVIFEEGSWGRWVQKKFQKHIGFAPCIFHGRGLFS
 6 amiss_dgat_XP_014456982.1       56.7%
 AESLNCTPGKNLVILKNRKGFVKLALQHGADLVPVYSFGENEVYKQVIFEEGCWGRWVQKKFQKYIGFAPCIFHGRGLFS

                                              321           .           .           :           .           .
 .           .    ] 394
 1 ruby_dgat_i1_HQ_rubyallcluster 100.0%
VQSRGLLPYEKPITTVVGEPLVVPKVRNPSSEMVDTYHQMYISSLIKLFHENKTKYGMLETDELHIL-------
 2 canna_dgat_XP_008493408.1       59.2%
SNTWGLLPYSKPITTVVGEPITIPKVDNPSQEEVDFYHSIYVDSLIKLFDKYKIKFGLPETEVLEVN-------
 3 cpelagica_dgat_XP_010005498.1   60.6%
SNTWGLLPYSKPITTVVGEPITIPKIENPSQQDVDFYHSMYVDSLIKLFDKYKSKFGLRKTDTLIIVCAQAA--
 4 hsapiens_dgat_NP_115953.2       51.4%
SDTWGLVPYSKPITTVVGEPITIPKLEHPTQQDIDLYHTMYMEALVKLFDKHKTKFGLPETEVLEVN-------
 5 gallus_dgat_XP_419374.3         58.1%
SNTWGLLPYSKPITTVVGEPITIPKIDNPSQKEVDFYHSVYVDSLIKLFDKYKGRFGLPETEVLEVN-------
 6 amiss_dgat_XP_014456982.1       56.7%
SNTWGLIPYPNPITTVVGEPITIPKTAHPTQREIDLYHSMYVSSLSKLFDKYKAKFGLPETEILEVN-------
```

MView 1.60.1, Copyright © 1997-2015 Nigel P. Brown

```
 Reference sequence (1): ruby_fasn_i12_HQ_rubyallcluste
 Identities normalised by aligned length.
 Colored by: identity + property

                                                1 [           .           .           .           .           :
 .           .           . 80
 1 ruby_fasn_i12_HQ_rubyallcluste 100.0%
 MEDVVIAGIAGKLPESENLQEFWENLLNGVDMVTEDDRRWKPGIYGLPRRNGKLKDISKFDASFFGVHPKQAHTMDPQLR
 2 canna_FASN_KFP01543.1           99.1%
 -EDVVIAGIAGKLPESENLQEFWENLLNGVDMVTEDDRRWKPGIYGLPRRNGKLKDISKFDASFFGVHPKQAHTMDPQLR
 3 hsapiens_fasn_NP_004095.4       65.6%
 -EEVVIAGMSGKLPESENLQEFWDNLIGGVDMVTDDDRRWKAGLYGLPRRSGKLKDLSRFDASFFGVHPKQAHTMDPQLR
 4 gallus_FASN_NP_990486.2         89.5%
 -EDVVIAGIAGKLPESENLQEFWENLLNGVDMVTEDDRRWKPGIYGLPKRNGKLKDIKKFDASFFGVHPKQAHTMDPQLR
 5 amiss_FASN_XP_006038234.1       73.5%
 -EDVVIAGIAGRLPESESLEEFWENLVGGVDMVTDDERRWKSGLHGLPRRTGKLNDISKFDASFFGTLPKQAAVMDPQLR
 6 cpelagica_FASN_KFU85956.1       89.5%
 MEDVVIAGIAGKLPESENLEEFWENLLNGVDMVTEDDRRWKPGMYGLPKRNGKLKDISKFDASFFGVHPKQAHTMDPQLR

                                               81           .           1           .           .           .
 .           :           . 160
 1 ruby_fasn_i12_HQ_rubyallcluste 100.0%
 LLLEVSYEAILDAGIDPATLRGTDTGVWVGASGSEAAEALSQDPEELLGYSMTGCQRGMFANRISYFYDLTGPSLTIDTA
 2 canna_FASN_KFP01543.1           99.1%
 LLLEVSYEAILDAGIDPATLRGTDTGVWVGASGSEAAEALSQDPEELLGYSMTGCQRGMFANRISYFYDLTGPSLTIDTA
 3 hsapiens_fasn_NP_004095.4       65.6%
 LLLEVTYEAIVDGGINPDSLRGTHTGVWVGVSGSETSEALSRDPETLVGYSMVGCQRAMMANRLSFFFDFRGPSIALDTA
 4 gallus_FASN_NP_990486.2         89.5%
 LLLEVSYEAILDGGINPTALRGTDTGVWVGASGSEAAEALSQDPEELLGYSMTGCQRAMLANRISYFYDFTGPSLTIDTA
 5 amiss_FASN_XP_006038234.1       73.5%
 LLLEVSYEAILDGGINPASLRGSNTGVWIGISGAEAIEVLSQDPETVVGYSMIGSQRAMFANRLSFFYDLKGPSMSVDAA
 6 cpelagica_FASN_KFU85956.1       89.5%
 MLLEVSYEAILDAGINPATLRGTDTGVWIGTSGSEAGEALSQDPEELVGYSMTGCQRAMFSNRISYFLDLTGPSITIDTA

                                              161           .           .           .           2           .
 .           .           . 240
 1 ruby_fasn_i12_HQ_rubyallcluste 100.0%
 CSSSLIALENAYKAIRHGQCSAALVGGVNLLLKPNTSVQFMKLGMLSPEGACKAFDASGNGYCRSEAVVIVLLTKRSMAK
 2 canna_FASN_KFP01543.1           99.1%
 CSSSLIALENAYKAIRHGQCSAALVGGVNLLLKPNTSVQFMKLGMLSPEGACKAFDASGNGYCRSEAVVIVLLTKRSMAK
 3 hsapiens_fasn_NP_004095.4       65.6%
 CSSSLMALQNAYQAIHSGQCPAAIVGGINVLLKPNTSVQFLRLGMLSPEGTCKAFDTAGNGYCRSEGVVAVLLTKKSLAR
 4 gallus_FASN_NP_990486.2         89.5%
 CSSSLMALENAYKAIRHGQCSAALVGGVNILLKPNTSVQFMKLGMLSPDGACKAFDVSGNGYCRSEAVVVVLLTKKSMAK
 5 amiss_FASN_XP_006038234.1       73.5%
 CSSSLLALEVAYKAICHGECDAALVGGCSIMLKPNTSLQFMKLGMLSPDGTCKAFDASGNGYCRSEGVVVVLLTKKSMAK
 6 cpelagica_FASN_KFU85956.1       89.5%
 CSSSLIALENAYKAIRHEQCSAALVGGVNILLKPNTSVQFMKLGMLSPDGACKAFDVSGNGYCRSEAVVVVLLTKRSMAK

                                              241           :           .           .           .           .
```

```
3          .          . 320
1 ruby_fasn_i12_HQ_rubyallcluste 100.0%
RIYATIVNAGTNTDGFKEQGVTFPSGEMQQQLISSLYRESGISPEEVEYVEAHGTGTKAGDPQELNSIVNVFCKCEREPL
2 canna_FASN_KFP01543.1           99.1%
RIYATIVNAGTNTDGFKEQGVTFPSGEMQQQLISSLYRESGISPEEVEYVEAHGTGTKAGDPQELNSIVNVFCKCEREPL
3 hsapiens_fasn_NP_004095.4       65.6%
RVYATILNAGTNTDGFKEQGVTFPSGDIQEQLIRSLYQSAGVAPESFEYIEAHGTGTKVGDPQELNGITRALCATRQEPL
4 gallus_FASN_NP_990486.2         89.5%
RVYATIVNAGSNTDGFKEQGVTFPSGEMQQQLVGSLYRECGIKPGDVEYVEAHGTGTKVGDPQEVNGIVNVFCQCEREPL
5 amiss_FASN_XP_006038234.1       73.5%
RVYATIVNAGTNTDGFKEQGLTFPCGEMHQRLFSSLYTASGILPDEMEYVEAHGTGTKAGDPQEVNAIAKVLCPGRREPL
6 cpelagica_FASN_KFU85956.1       89.5%
RVYATIVNAGSNTDGFKEQGVTFPSGEMQQQLIRSVYRECGVSPGEVEYIEAHGTGTKAGDPQEVNSIVNFFCKCEREPL

                                            321          .          .          :          .          .
.          .          4 400
1 ruby_fasn_i12_HQ_rubyallcluste 100.0%      KIGSTKSNMGHPEPAAGLAALAKVILSLEHGLWAPNLHFNT-
-PNPDIPGLQDGSLEVICKPTPVKGGLVSINSFGFGGA
2 canna_FASN_KFP01543.1           99.1%
KIGSTKSNMGHPEPAAGLAALAKVTGGLRRKIGQGRKHNSVGLVCFDIPGLQDGSLEVICKPTPVKGGLVSINSFGFGGA
3 hsapiens_fasn_NP_004095.4       65.6%      LIGSTKSNMGHPEPASGLAALAKVLLSLEHGLWAPNLHFHS-
-PNPEIPALLDGRLQVVDQPLPVRGGNVGINSFGFGGS
4 gallus_FASN_NP_990486.2         89.5%      LIGSTKSNMGHPEPASGLAALAKVILSLEHGLWAPNLHFND-
-PNPDIPALHDGSLKVVCKPTPVKGGLVSINSFGFGGS
5 amiss_FASN_XP_006038234.1       73.5%      LIGSTKSNMGHPEPASGLVALLKVILSLENGVWAPNLHYNT-
-PNPDIPALQDGSLQVVCKPTPVKGGLVGINSFGFGGS
6 cpelagica_FASN_KFU85956.1       89.5%      KIGSTKSNMGHPEPASGLAALAKVILSLEHGLWAPNLHFNT-
-PNPDIPALQDGTLEVVCKPTPVKGGLVSINSFGFGGA

                                            401          .          .          .          .          :
.          .          . 480
1 ruby_fasn_i12_HQ_rubyallcluste 100.0%
NAHVILRPNENRR-QPLETCNIPRLVQVCGRTQEAVEILIQESRKHGGCSPFVSLLSDISAMPVSSMPYRGYTLVGTESD
2 canna_FASN_KFP01543.1           99.1%
NAHVILRPNENRR-QPPETCNIPRLVQVCGRTQEAVEVLIQESRKHGGCSPFVSLLSDISAMPVSSMPYRGYTLVGTESD
3 hsapiens_fasn_NP_004095.4       65.6%
NVHIILRPNTQPPPAPAPHATLPRLLRASGRTPEAVQKLLEQGLRHSQDLAFLSMLNDIAAVPATAMPFRGYAVLGGERG
4 gallus_FASN_NP_990486.2         89.5%
NAHVILRPNEKKC-QPQETCNLPRLVQVCGRTQEAVEILIEESRKHGGCSPFLSLLSDISAVPVSSMPYRGYTLVGTESD
5 amiss_FASN_XP_006038234.1       73.5%
NVHVILRPHDKKKSQPLEARCLPRLVQVFGRTQEAVEKLLDQSKRLGEDDSFVSLLNDLSAIPASSMPYRGYRLVGSESD
6 cpelagica_FASN_KFU85956.1       89.5%
NAHVILRPNENKR-QPLETCNTPRLVQVCGRTQEAVEKLIQESRRHGGCSPFVSLLSDISAISVSSMPYRGYTLVGTESD

                                            481          .          5          .          .          .
.          :          . 560
1 ruby_fasn_i12_HQ_rubyallcluste 100.0%
IQEIQQVQASGRPLWYICSGMGTQWKGMGLSLMKLDLFRQSILRSDQALKNTGLKVSDLLLQADDNTFDETVHAFVGLAA
2 canna_FASN_KFP01543.1           99.1%
IQEIQQVQASGRPLWYICSGMGTQWKGMGLSLMKLDLFRQSILRSDQALKNTGLKVSDLLLQADDNTFDETVHAFVGLAA
3 hsapiens_fasn_NP_004095.4       65.6%
GPEVQQVPAGERPLWFICSGMGTQWRGMGLSLMRLDRFRDSILRSDEAVKPFGLKVSQLLLSTDESTFDDIVHSFVSLTA
4 gallus_FASN_NP_990486.2         89.5%
ITEIQQVQASGRPLWYICSGMGTQWKGMGLSLMKLDLFRQSILRSDEALKSTGLKVSDLLLNADENTFDDTVHAFVGLAA
5 amiss_FASN_XP_006038234.1       73.5%
VKEVQQIQASGRPLWYICTGMGTQWAGMGRSLMQLELFRQSILRSDEILKGTGLKVSDIILNGDESMFDDAVCSFVGVSA
6 cpelagica_FASN_KFU85956.1       89.5%
IKEVQQVQASGRPLWYICSGMGTQWQGMGLSLMKLDLFRQSILRSDEALKSTRLKVSDLLLQADENTFDDIVHAFVGLAA

                                            561          .          .          .          6          .
.          .          . 640
1 ruby_fasn_i12_HQ_rubyallcluste 100.0%
IQIAQIDMLKAAGLQPDGILGHSVGELACGYADNCLSHEEAILAAYWRGRCVKEAKLPPGGMAAVGLTWEECKQQCPPNV
2 canna_FASN_KFP01543.1           99.1%
IQIAQIDMLKAAGLQPDGILGHSVGELACGYADNCLSHEEAILAAYWRGRCVKEAKLPPGGMAAVGLTWEECKQQCPPNV
3 hsapiens_fasn_NP_004095.4       65.6%
IQIGLIDLLSCMGLRPDGIVGHSLGEVACGYADGCLSQEEAVLAAYWRGQCIKEAHLPPGAMAAVGLSWEECKQRCPPGV
4 gallus_FASN_NP_990486.2         89.5%
IQIAQIDVLKAAGLQPDGILGHSVGELACGYADNSLSHEEAVLAAYWRGRCVKEAKLPPGGMAAVGLTWEECKQRCPPNV
5 amiss_FASN_XP_006038234.1       73.5%
VQVAQIDMLKSMGLQPDGIIGHSVGEVACGYADDSFSHEEAILSAYWRGRCVKEANLPRGKMAAVGLSWEECKIRCPPGV
6 cpelagica_FASN_KFU85956.1       89.5%
IQIAQIDILKAAGLQPDGILGHSVGEVACGYADNSLTHEEAILAAYWRGQCVKENKLPPGGMAAVGLTWEECKQQCPPNV

                                            641          :          .          .          .          .
7          .          . 720
1 ruby_fasn_i12_HQ_rubyallcluste 100.0%
VPACHNSEDTVTISGPLATVSEFVAKLKKAGVFAKEVRSAGVAFHSHYMASIAPVLLSALKKVIPHPKPRSARWISTSIP
2 canna_FASN_KFP01543.1           99.1%
VPACHNSEDTVTISGPLATVSEFVAKLKKAGVFAKEVRSAGVAFHSHYMASIAPVLLSALKKVIPHPKPRSARWISTSIP
3 hsapiens_fasn_NP_004095.4       65.6%
VPACHNSKDTVTISGPQAPVFEFVEQLRKEGVFAKEVRTGGMAFHSYFMEAIAPPLLQELKKVIREPKPRSARWLSTSIP
4 gallus_FASN_NP_990486.2         89.5%
VPACHNSEDTVTVSGPLDSVSEFVTKLKKDGVFAKEVRSAGVAFHSYYMASIAPALLSALKKVIPHPKPRSARWISTSIP
5 amiss_FASN_XP_006038234.1       73.5%
```

```
VPACHNAEDTVTISGPEATMNEFLATLKKEGVFAKEVRSVGVAFHSYFMEALAPMLLSVLRKIIPNPKPRSARWISTSIP
6 cpelagica_FASN_KFU85956.1        89.5%
VPACHNSEDTVTVSGTLASVNEFVAKLKKDGVFAKEVRSAGVAFHSPYMASIAPGLLSALKKVIPHPKPRSARWISTSIP

                                              721          .          .          :          .          .
.          .          8 800
1 ruby_fasn_i12_HQ_rubyallcluste 100.0%
ESQWQSDLAKYSSAEYHVNNLVSPVLFHEGLKHIPENAVVVEIAPHALLQAILKRTLKPTCTILPLMKKDHKNNLEFFLT
2 canna_FASN_KFP01543.1            99.1%
ESQWQSDLAKYSSAEYHVNNLVSPVLFHEGLKHIPENAVVVEIAPHALLQAILKRTLKPTCTILPLMKKDHKNNLEFFLT
3 hsapiens_fasn_NP_004095.4        65.6%
EAQWHSSLARTSSAEYNVNNLVSPVLFQEALWHVPEHAVVLEIAPHALLQAVLKRGLKPSCTIIPLMKKDHRDNLEFFLA
4 gallus_FASN_NP_990486.2          89.5%
ESQWQSDLARNSSAEYHVNNLVNPVLFHEGLKHIPENAVVVEIAPHALLQAILRRTLKPTCTILPLMKKDHKNNLEFFLT
5 amiss_FASN_XP_006038234.1        73.5%
EAQWGSELARYSSAEYHVNNLRSPVLFQEGLQHVPDNAVVVEIAPHALLQAILKRSLKPTCTILPLMKREHKNNLEFFLT
6 cpelagica_FASN_KFU85956.1        89.5%
ENQWQSDLAKNSSAEYYVNNLVSPVLFHEGLKHIPENAVVVEIAPHALLQGVLRRALKPTCTILPLMKKEHKNNLEFFLT

                                              801          .          .          .          .          :
.          .          . 880
1 ruby_fasn_i12_HQ_rubyallcluste 100.0%
QAGKIHLTGINVLGNNLFPPVEYPVPVGTPLISPYIKWDHSQGWDVPKAEDFPSGSKGSASASIYNIDVSPDSPDHYLVG
2 canna_FASN_KFP01543.1            99.1%
QAGKIHLTGINVLGNNLFPPVEYPVPVGTPLISPYIKWDHSQGWDVPKAEDFPSGSKGSASASIYNIDVSPDSPDHYLVG
3 hsapiens_fasn_NP_004095.4        65.6%
GIGRLHLSGIDANPNALFPPVEFPAPRGTPLISPLIKWDHSLAWDVPAAEDFPNGS-GSPSAAIYNIDTSSESPDHYLVD
4 gallus_FASN_NP_990486.2          89.5%
QTGKIHLTGINVLGNNLFPPVEYPVPVGTPLISPYIKWDHSQDWDVPKAEDFPSGSKGSASASVYNIDVSPDSPDHYLVG
5 amiss_FASN_XP_006038234.1        73.5%
HIGKIYLTGINVQSNKLFPPVEYPVPVGTPLISPHILWDHSETWYYPKAEEFPSGSAGSSSASVYNIDVNPESPDHYLIG
6 cpelagica_FASN_KFU85956.1        89.5%
QTGKIHLTGINVLGNNLFPPVEYPVPVGTPLISPYIKWDHSQDWDVPKAGDFPSGSRGSASASIYNIDVSPDSPDHYLVG

                                              881          .          9          .          .          .
.          :          . 960
1 ruby_fasn_i12_HQ_rubyallcluste 100.0%
HCIDGRVLYPATGYLVLAWRTLARSLGMVMEQTAVKFEDVTIHQATILPKNGSVQLEVRLMPASHSFEVSGNGNLAVSGK
2 canna_FASN_KFP01543.1            99.1%
HCIDGRVLYPATGYLVLAWRTLARSLGMVMEQTAVKFEDVTIHQATILPKNGSVQLEVRLMPASHSFEVSGNGNLAVSGK
3 hsapiens_fasn_NP_004095.4        65.6%
HTLDGRVLFPATGYLSIVWKTLARALGLGVEQLPVVFEDVVLHQATILPKTGTVSLEVRLLEASRAFEVSENGNLVVSGK
4 gallus_FASN_NP_990486.2          89.5%
HCIDGRVLYPATGYLVLAWRTLARSLGMVMEQTAVMFEEVTIHQATILPKKGSTQLEVRIMPASHSFEVSGNGNLAVSGK
5 amiss_FASN_XP_006038234.1        73.5%
HCVDGRVLYPATGYLVLAWRTLARSLGAIMEQMPVKFEDVEIHQATILPKKGSVQLEVRLMPASHSFEVSGNGNLSVSGK
6 cpelagica_FASN_KFU85956.1        89.5%
HCIDGRVLYPATGYLVLAWRTLARSLGMTMEKTAVKFEDVTIHQATILPKKGSVQLEVRLMPASHCFEVSGNGNLAVSGK

                                              961          .          .          .          0          .
.          .          . 1040
1 ruby_fasn_i12_HQ_rubyallcluste 100.0%      ISLLENTALK----
-PADFHTRAGMGLKPGLLKEDIYQELHLRGYNYGPTFQGVLECNSEASAAKVLWNGNWVTFLDTLL
2 canna_FASN_KFP01543.1            99.1%      ISLLENTALK----
-PADFHTRAGMGLKPGLLKEDIYQELHLRGYNYGPTFQGVLECNSEASAAKVLWNGNWVTFLDTLL
3 hsapiens_fasn_NP_004095.4        65.6%      VYQWDDPDPR---
LFDHPESPTPNPTEPLFLAQAEVYKELRLRGYDYGPHFQGILEASLEGDSGRLLWKDNWVSFMDTML
4 gallus_FASN_NP_990486.2          89.5%
ISLLENDALKNFHNQLADFQSQANVTAKSGLLMEDVYQELHLRGYNYGPTFQGVLECNSEGSAGKILWNGNWVTFLDTLL
5 amiss_FASN_XP_006038234.1        73.5%
IYLLEDTALNNFHNEQADFDAQPEASSSHPLSKTDIYTELHLRGYNYGPTFQGLLECSSSGSHGKLLWNGNWVTFLDTML
6 cpelagica_FASN_KFU85956.1        89.5%
ISLLENTAQKNFHNQSVDFQTQVDKSSKPGLLKEDIYQELQLRGYNYGPTFQGVLECNSEGNTGKVLWNGNWVTFLDTLL

                                             1041          :          .          .          .          .
1          .          . 1120
1 ruby_fasn_i12_HQ_rubyallcluste 100.0%
HVIILSDTGRSLRLPTRIRSVCVDPVLHQEMVYQYQDNVEVCDAVVDLCLDRLQAGGVQLDGLHASVAPRRQQERMTPTL
2 canna_FASN_KFP01543.1            99.1%
HVIILSDTGRSLRLPTRIRSVCVDPVLHQEMVCQYQDNVEVCDAVVDLCLDRLQAGGVQLDGLHASVAPRRQQERMTPTL
3 hsapiens_fasn_NP_004095.4        65.6%
QMSILGSAKHGLYLPTRVTAIHIDPATHRQKLYTLQDKAQVADVVVSRWLRVTVAGGVHISGLHTESAPRRQQEQQVPIL
4 gallus_FASN_NP_990486.2          89.5%
HLIVLAETGRSLRLPTRIRSVYIDPVLHQEQVYQYQDNVEAFDVVVDRCLDSLKAGGVQINGLHASVAPRRQQERISPTL
5 amiss_FASN_XP_006038234.1        73.5%
HVTVLGYPGRSLRLPTRIRSVCVDPVLHLKQVQTYKDDKQAFDVFYDHCLGSIRSGGVQVAGLHSSTAPRRQQEQGSPLL
6 cpelagica_FASN_KFU85956.1        89.5%
HVIILSETGRSLRLPTRIRSVSIDPVLHQEQVCQYQDNVEAFDVVVDRCLDNLKAGGVQIDGLHASVAPRRQQERNSPTL

                                             1121          .          .          :          .          .
.          .          2 1200
1 ruby_fasn_i12_HQ_rubyallcluste 100.0%
EKFCFVPYTESDCLSSNAHLHDCLDHCKVLIQKMQAKLAVHGVKLVIPGLDTGSTAAKSSPIQKGLQHILTEICRLELNG
2 canna_FASN_KFP01543.1            99.1%
EKFCFVPYTESDCLSSNAHLHDCLDHCKVLIQKMQAKLAVHGVKLVIPGLDTGSTAAKSSPIQKGLQHILTEICRLELNG
```

```
3 hsapiens_fasn_NP_004095.4       65.6%
EKFCFTPHTEEGCLSERAALQEELQLCKGLVQALQTKVTQQGLKMVVPGLDGAQIPRD--PSQQELPRLLSAACRLQLNG
4 gallus_FASN_NP_990486.2         89.5%
EKFSFVPYIESDCLSSSTQLHAYLEHCKGLIQKLQAKMALHGVKLVIHGLETKGAAAGSPPTQKGLQHILTEICRLELNG
5 amiss_FASN_XP_006038234.1       73.5%
EKFCFVPYVEMDCLSSDAQLHTVLEHCKGLIQNLQNKVALHGVRLAIPGLETTGTRAETVPAQKGLLYILDKICHLELNG
6 cpelagica_FASN_KFU85956.1       89.5%
EKFCFVPYTESDCLSSSVHLHDYLNHCKDLIQNLEEKMAVHGVRLVIPGLETEKGVSKSSPIQKGLQHILAEICRLELNG

                                                1201            .           .           .           .           :
.           .           . 1280
1 ruby_fasn_i12_HQ_rubyallcluste 100.0%
NLRSELEQIVTREKMHFHDDPLLSGLLDSSELKSCLDVAVENMTSHRMKIIEALAGSGRLFSRVKSILNTQPLLQVDYIA
2 canna_FASN_KFP01543.1           99.1%
NLRSELEQIVTREKMHFHDDPLLSGLLDSSELKSCLDVAVENMTSHRMKIIEALAGSGRLFSRVKSILNTQPLLQVDYIA
3 hsapiens_fasn_NP_004095.4       65.6%
NLQLELAQVLAQERPKLPEDPLLSGLLDSPALKACLDTAVENMPSLKMKVVEVLAGHGHLYSRIPGLLSPHPLLQLSYTA
4 gallus_FASN_NP_990486.2         89.5%
NLHSELEQIVTQEKMHLQDDPLLNGLLDSSELKTCLDVAKENTTSHRMKIVEALAGSGRLFSRVQSILNTQPLLQLDYIA
5 amiss_FASN_XP_006038234.1       73.5%
NLYSELDEIVTQEKMHLQEDPLLNGLLDSPELKVCLDLTVENTSNIKMKIVEALAGDGCLFPRITPLLGTHPLLEVDYTA
6 cpelagica_FASN_KFU85956.1       89.5%
NLHSELQQIVTREKMHFQDDPLLNGLLDSSELKTCLDVALENMTSHRMKIVEALAGSGRLFSRVKSILNTQPLLQVDYIA

                                                1281            .           3           .           .           .
.           :           . 1360
1 ruby_fasn_i12_HQ_rubyallcluste 100.0%
TDRVLETLSASETELQDAGVSSSQWDPSSLPSGNLTNADLVVYNCATNVLGNTTEILSNLAAAVKEGGFVLLHTLLKGET
2 canna_FASN_KFP01543.1           99.1%
TDRVLETLSASETELQDAGVSSSQWDPSSLPSGNLTNADLVVYNCATNVLGNTTEILSNLAAAVKEGGFVLLHTLLKGET
3 hsapiens_fasn_NP_004095.4       65.6%
TDRHPQALEAAQAELQQHDVAQGQWDPADPAPSALGSADLLVCNCAVAALGDPASALSNMVAALREGGFLLLHTLLRGHP
4 gallus_FASN_NP_990486.2         89.5%
TDCTPETLSDNETELHDAGISFSQWDPSSLPSGNLTNADLAVCNCSTSVLGNTAEIISNLAAAVKEGGFVLLHTLLKEET
5 amiss_FASN_XP_006038234.1       73.5%
TDRAQEVLSAHEKQFQETSVSPGQWDPVRPPPGNLTNADLLVLNCSMKVLEKPAEILSNLVASVKEGGFVLLHTLLGGET
6 cpelagica_FASN_KFU85956.1       89.5%
TDHIQEALSAHETELEDAGVSFSQWDPSSLPSGNLTNADMVVYNCSTSALGNNAEILSNLAAGVKEGGFVLVHTLLKGET

                                                1361            .           .           .           4           .
.           .           . 1440
1 ruby_fasn_i12_HQ_rubyallcluste 100.0%
LGEIVSFLSSPDLQQKHKFLHETQWEDLFSKASLNLVAMKKSFFGSVIFLCRRQVPAKTPIFLPVDETHYKWVESLKEIL
2 canna_FASN_KFP01543.1           99.1%
LGEIVSFLSSPDLQQKHKFLHETQWEDLFSKASLNLVAMKKSFFGSVIFLCRRQVPAKTPIFLPVDETHYKWVESLKEIL
3 hsapiens_fasn_NP_004095.4       65.6%
LGDIVAFLTSTEPQYGQGILSQDAWESLFSRVSLRLVGLKKSFYGSTLFLCRRPTPQDSPIFLPVDDTSFRWVESLKGIL
4 gallus_FASN_NP_990486.2         89.5%
LGEIVSFLTSPDLQQKHSFLSQAQWEELFSKASLNLVAMKRSFFGSVIFLCRRQSPAKAPILLPVDDTHYKWVDSLKEIL
5 amiss_FASN_XP_006038234.1       73.5%
LGETVHFLTALDVQQRPGFLTQAAWEDLFSKTSLNLVAVKRSFFGSVMFLCRRQAPVKPPIFLPVDETHCKWVDFLKNFM
6 cpelagica_FASN_KFU85956.1       89.5%
LGEIVSFLTSPDLQQKHRFLSEAQWEGLFSKASLNLVAIKRSFFGSVIFLCRRQVPAKTPIFLPIDETHYKWVESLKEIL

                                                1441            :           .           .           .           .
5           .           . 1520
1 ruby_fasn_i12_HQ_rubyallcluste 100.0%
AD-SSEQPVWLTATSCGNSGILGMVNCLRLESEGHRIRCVFISNLNPSSAVPPTSHSSLEMQKIVQGDLVMNVYRDGKWG
2 canna_FASN_KFP01543.1           99.1%
AD-SSEQPVWLTATSCGNSGILGMVNCLRLESEGHRIRCVFISNLNPSSAVPPTSHSSLEMQKIVQGDLVMNVYRDGKWG
3 hsapiens_fasn_NP_004095.4       65.6%
ADEDSSRPVWLKAINCATSGVVGLVNCLRREPGGNRLRCVLLSNLSSTSHVPEVDPGSAELQKVLQGDLVMNVYRDGAWG
4 gallus_FASN_NP_990486.2         89.5%
AD-SSEQPLWLTATNCGNSGILGMVNCLRLEAEGHRIRCVFVSNLSPSSTVPATSLSSLEMQKIIERDLVMNVYRDGKWG
5 amiss_FASN_XP_006038234.1       73.5%
AD-SSEQPLWLTATKYPTSGIVGLMTCLRQEPGGHRIRSLFVSNLESSSPAPPTSPSDSEMQKILQRDLVMNVYRDGKWG
6 cpelagica_FASN_KFU85956.1       89.5%      AG-
PSEQPVWLTATSCGNSGILGMVNCLRLEAEGHRIRCVFISNLNPSSAVPPTSPSSLDMQKVVQNDLVMNVYRDGKWG

                                                1521            .           .           :           .           .
.           .           6 1600
1 ruby_fasn_i12_HQ_rubyallcluste 100.0%
SFRHLPLQQAQPQELTEYAYVNVLTRGDLSSLRWIVSPLQHFQANNPDIQLCKVYYASLNFRDIMLATGKLSPDAIPGNW
2 canna_FASN_KFP01543.1           99.1%
SFRHLPLQQAQPQELTEYAYVNVLTRGDLSSLRWIVSPLQHFQANNPDIQLCKVYYASLNFRDIMLATGKLSPDAIPGNW
3 hsapiens_fasn_NP_004095.4       65.6%
AFRHFLLEEDKPEEPTAHAFVSTLTRGDLSSIRWVCSSLRHAQPTCPGAQLCTVYYASLNFRDIMLATGKLSPDAIPGKW
4 gallus_FASN_NP_990486.2         89.5%
SFRHLPLQQAQPQELTEYAYVNVLTRGDLSSLRWIVSPLRHFQTTNPNVQLCKVYYASLNFRDIMLATGKLSPDAIPGNW
5 amiss_FASN_XP_006038234.1       73.5%      SFRHLLLEQAQPQTLTEHACVNTLMRGDLSSLRWIASPLCHYPST----
KLCKVYYASLNFRDIMLASGKLPADAIPGNW
6 cpelagica_FASN_KFU85956.1       89.5%
SFRHLPLQQAQPQELTEYAYVNVLTRGDLSSLRWIVSPLQHFCANNPNVQLCKVYYTSLNFRDIMLATGKLSPDAIPGNW

                                                1601            .           .           .           .           :
```

```
.         .         . 1680
1 ruby_fasn_i12_HQ_rubyallcluste 100.0%
VLQQCMLGMEFSGRDMAGRRVMGLLPAKGLATVVDCEKKFLWEVPKNWTLEEAASVPVVYATAYYALVVRGRMKKGESVL
2 canna_FASN_KFP01543.1           99.1%
VLQQCMLGMEFSGRDMAGRRVMGLLPAKGLATVVDCEKKFLWEVPKNWTLEEAASVPVVYATAYYALVVRGRMKKGESVL
3 hsapiens_fasn_NP_004095.4       65.6%
TSQDSLLGMEFSGRDASGKRVMGLVPAKGLATSVLLSPDFLWDVPSNWTLEEAASVPVVYSTAYYALVVRGRVRPGETLL
4 gallus_FASN_NP_990486.2         89.5%
TLQQCMLGMEFSGRDLAGRRVMGLLPAKGLATVVDCDKRFLWEVPENWTLEEAASVPVVYATAYYALVVRGGMKKGESVL
5 amiss_FASN_XP_006038234.1       73.5%
STKDNLLGMEFSGRDPAGKRVMGLLQAKGLATAVDIDRCCLWDVPENWTLEEAATVPAVYATAYYALISRGGMKRGESVL
6 cpelagica_FASN_KFU85956.1       89.5%
AMQQCMLGMEFSGRDLAGRRVMGLLPAKGLATVVDCDTRFLWEVPKNWTLEEAASVPVVYATAYYALVVRGGMKKGESIL

                                                  1681           .         7         .         .         .
.         :         . 1760
1 ruby_fasn_i12_HQ_rubyallcluste 100.0%
VHSGSGGVGQAAIAIALSMGCRVFTTVGSAEKREYLQARFPQLDANSFASSRDTAFEQHVLRVTNGKG-----VNLVLNS
2 canna_FASN_KFP01543.1           99.1%
VHSGSGGVGQAAIAIALSMGCRVFTTVGSAEKREYLQARFPQLDANSFASSRDTAFEQHVLRVTNGKG-----VNLVLNS
3 hsapiens_fasn_NP_004095.4       65.6%
IHSGSGGVGQAAIAIALSLGCRVFTTVGSAEKRAYLQARFPQLDSTSFANSRDTSFEQHVLWHTGGKG-----VDLVLNS
4 gallus_FASN_NP_990486.2         89.5%
IHSGSGGVGQAAIAIALSMGCRVFATVGSAEKREYLQARFPQLDANSFASSRNTTFEQHILRVTNGKG-----VNLVLNS
5 amiss_FASN_XP_006038234.1       73.5%
IHSGSGGVGQAATIALSMGCRVFTTVGSTEKRKYLQARFPQLDASSFANSRSTAFEQHILRATKGKG-----VDLVLNS
6 cpelagica_FASN_KFU85956.1       89.5%
IHSGSGGVGQAAIAIALSMGCRVFATVGSTEKREYLQARFPQLDANSFASSRSAAFEQHILRVTNGKGKFKECVNLVLNS

                                                  1761           .         .         .         8         .
.         .         . 1840
1 ruby_fasn_i12_HQ_rubyallcluste 100.0%
LAEEKLQASLRCLARHGRFLEIGKFDLSNNSQLGMALFLKNVAFHGILLDAIFEGGNQEWDIVSELLTKGIKDGVVKPLK
2 canna_FASN_KFP01543.1           99.1%
LAEEKLQASLRCLARHGRFLEIGKFDLSNNSQLGMALFLKNVAFHGILLDAIFEGGNQEWDIVSELLTKGIKDGVVKPLK
3 hsapiens_fasn_NP_004095.4       65.6%
LAEEKLQASVRCLATHGRFLEIGKFDLSQNHPLGMAIFLKNVTFHGVLLDAFFNESSADWREVWALVQAGIRDGVVRPLK
4 gallus_FASN_NP_990486.2         89.5%
LAEEKLQASLRCLAQHGRFLEIGKFDLSNNSQLGMALFLKNVAFHGILLDSIFEEGNQEWEVVSELLTKGIKDGVVKPLR
5 amiss_FASN_XP_006038234.1       73.5%
LAEEKLQASLRCLAQHGRFLEIGKFDLSKNNHLGMALFLKNVTFHGILLDAVLKKGSPAWVEVEELVKKGIRDGIVKPLK
6 cpelagica_FASN_KFU85956.1       89.5%
LAEEKLQASLRCLARHGRFLEIGKFDLSNNSQLGMALFLKNVAFHGILLDAIFEDGNQEWDLVSELLTKGIKDGVVKPLR

                                                  1841           :         .         .         .         .
9         .         . 1920
1 ruby_fasn_i12_HQ_rubyallcluste 100.0%
STVFKKEEVEAAFRFMAQGKHIGKVMIQIQEEEKEYSVRRSEPVKISAISRTSCPPSKSYIITGGLGGFGLELAQWLVER
2 canna_FASN_KFP01543.1           99.1%
STVFKKEEVEAAFRFMAQGKHIGKVMIQIQEEEKEYSVRRSEPVKISAISRTSCPPSKSYIITGGLGGFGLELAQWLVER
3 hsapiens_fasn_NP_004095.4       65.6%
CTVFHGAQVEDAFRYMAQGKHIGKVVVQVLAEEPEAVLKGAKPKLMSAISKTFCPAHKSYIIAGGLGGFGLELAQWLIQR
4 gallus_FASN_NP_990486.2         89.5%
TTVFGKEEVEAAFRFMAQGKHIGKVMIKIQEEEKQYPLR-SEPVKLSAISRTSCPPTKSYIITGGLGGFGLELAQWLIER
5 amiss_FASN_XP_006038234.1       73.5%
HTVFGKEELEAAFRFMAQGKHIGKVMVKVREEEKDLPASDSVLTQLPAISRTFCPPTKSYIITGGLGGFGLELAHWLAER
6 cpelagica_FASN_KFU85956.1       89.5%
STVFNKEEVEAAFRFMAQGKHIGKVMIKIQEEEKEYALRRSEPVKISAISRTSCPPTKSYIITGGLGGFGLELAQWLVER

                                                  1921           .         .         :         .         .
.         .         0 2000
1 ruby_fasn_i12_HQ_rubyallcluste 100.0%
GAQKLILTSRSGIRTGYQARRVREWKALGIQVLVSTSDIGTLEGAQRLIEEALQLGPVGGIFNLAVVLRDAMIENQTPEL
2 canna_FASN_KFP01543.1           99.1%
GAQKLILTSRSGIRTGYQARRVREWKALGIQVLVSTSDIGTLEGAQRLIEEALQLGPVGGIFNLAVVLRDAMIENQTPEL
3 hsapiens_fasn_NP_004095.4       65.6%
GVQKLVLTSRSGIRTGYQAKQVRRWRRQGVQVQVSTSNISSLEGARGLIAEAAQLGPVGGVFNLAVVLRDGLLENQTPEF
4 gallus_FASN_NP_990486.2         89.5%
GAQKLVLTSRSGIRTGYQAKCVREWKALGIQVLVSTSDVGTLEGTQLLIEEALKLGPVGGIFNLAVVLKDAMIENQTPEL
5 amiss_FASN_XP_006038234.1       73.5%
GAQKLVLTSRSGVRTGYQAKQIGLWKEMGVQVLVSTSDVGTLEGTQKLLDEAIQLGPVGGIFNLAMVLRDAMMENQTPES
6 cpelagica_FASN_KFU85956.1       89.5%
GAQKLVLTSRSGIRTGYQAKRVREWKALGIQVLVSTSDIGTLEGAQQLIEEALQLGPVGGIFNLAVVLRDAMIENQTPEL

                                                  2001           .         .         .         .         :
.         .         . 2080
1 ruby_fasn_i12_HQ_rubyallcluste 100.0%
FVEVNKPKYSGTLHLDWVTRKKCPDLDYFVVFSSVSCGRGNAGQSNYGFANSTMERICEQRHHDGLPGLAIQWGAIGDVG
2 canna_FASN_KFP01543.1           99.1%
FVEVNKPKYSGTLHLDWVTRKKCPDLDYFVVFSSVSCGRGNAGQSNYGFANSTMERICEQRHHDGLPGLAIQWGAIGDVG
3 hsapiens_fasn_NP_004095.4       65.6%
FQDVCKPKYSGTLNLDRVTREACPELDYFVVFSSVSCGRGNAGQSNYGFANSAMERICEKRRHEGLPGLAVQWGAIGDVG
4 gallus_FASN_NP_990486.2         89.5%
FWEVNKPKYSGTLHLDWVTRKKCPDLDYFVVFSSVSCGRGNAGQSNYGFANSAMERICEQRHHDGLPGLAVQWGAIGDVG
5 amiss_FASN_XP_006038234.1       73.5%
```

```
FQEVCKPKSAGIIHLDRVSRKKCPDLDYFVAFSSVSSGRGNAGQTNYGFANSTMERVCEQRKHDGLPALCVQWGVVGDVG
6 cpelagica_FASN_KFU85956.1        89.5%
FGEVNKPKYSGTLHLDWVTRKKCPDLDYFVVFSSVSCGRGNAGQSNYGFANSTMERICEQRHHDGLP-------------

                                              2081          .          1          .          .          .
.          :          . 2160
1 ruby_fasn_i12_HQ_rubyallcluste 100.0%
ILLKTMGNKEVVVGGTIPQQISSCLEVLDTFLNQPHPVTSSFVLAEKVSVKAEGGSQRDLVEAVAHILGVRDVSSLNADS
2 canna_FASN_KFP01543.1          99.1%
ILLKTMGNKEVVVGGTIPQQISSCLEVLDTFLNQPHPVTSSFVLAEKVSVKAEGGSQRDLVEAVAHILGVRDVSSLNADS
3 hsapiens_fasn_NP_004095.4      65.6%
ILVETMSTNDTIVSGTLPQRMASCLEVLDLFLNQPHMVLSSFVLAEKAAAYRDRDSQRDLVEAVAHILGIRDLAAVNLDS
4 gallus_FASN_NP_990486.2        89.5%      -
ILKAMGNREVVIGGTVLQQISSCLEVLDMFLNQPHPVMSSFVLAEKVSVKSEGGSQRDLVEAVAHILGVRDVSSLNAES
5 amiss_FASN_XP_006038234.1      73.5%
VVIKTRGNDNTVIGGTIPQRIASCLEVLDQFLNQPHPIVSSFVLAEKISTKSEGSGPCDLVEAVAHILGVRDLNSLNAES
6 cpelagica_FASN_KFU85956.1      89.5%      -----
VGNKEIVVGGTIPQPISSCLEVLDTFLNQPHPVTSSFVLAEKVSMKTEGGSQRDLVEAVANILGVP---------

                                              2161          .          .          .          2          .
.          .          . 2240
1 ruby_fasn_i12_HQ_rubyallcluste 100.0%
SLADLGLDSLMGVEVRQTLERDYDIVMTMREIRLLTINKLRELSSKSGTTEELKPSQVMKTGAGEPP-KLDLNNLLVNPE
2 canna_FASN_KFP01543.1          99.1%
SLADLGLDSLMGVEVRQTLERDYDIVMTMREIRLLTINKLRELSSKSGTTEELKPSQVMKTGAGEPP-KLDLNNLLVNPE
3 hsapiens_fasn_NP_004095.4      65.6%
SLADLGLDSLMSVEVRQTLERELNLVLSVREVRQLTLRKLQELSSKADEASELACPTPKEDGLAQQQTQLNLRSLLVNPE
4 gallus_FASN_NP_990486.2        89.5%
SLADLGLDSLMGVEVRQTLERDYDIVMTMREIRLLTINKLRELSSKTGTAEELKPSQVLKTGPGEPP-KLDLNNLLVNPE
5 amiss_FASN_XP_006038234.1      73.5%      TLADLGLDSLMGVEIRQTLERDYDIIMPMREIRGLTINKLRELSTKSGTAEELMP-
FKTTAGQAKHP-ELNLNNLVLNPE
6 cpelagica_FASN_KFU85956.1      89.5%      ---
-LGLDSLMGVEVRQTLERDYDIVMTMREIRLLTINKLRELSSKSRTTEELKPSQVMKTGMGELP-KLDLNNLLVSPE

                                              2241          :          .          .          .          .
3          .          . 2320
1 ruby_fasn_i12_HQ_rubyallcluste 100.0%
GPTITRINDVQSTERPLFLVHPIEGSTAVFKTLASKLHMPCYGFQCTKVAPLDSIQSLATYYINCMKQIQPEGPYRIAGY
2 canna_FASN_KFP01543.1          99.1%
GPTITRINDVQSTERPLFLVHPIEGSTAVFKTLASKLHMPCYGFQCTKVAPLDSIQSLATYYINCMKQIQPEGPYRIAGY
3 hsapiens_fasn_NP_004095.4      65.6%
GPTLMRLNSVQSSERPLFLVHPIEGSTTVFHSLASRLSIPTYGLQCTRAAPLDSIHSLAAYYIDCIRQVQPEGPYRVAGY
4 gallus_FASN_NP_990486.2        89.5%
GPTITRLNEVQSTERPLFLVHPIEGSIAVFYTLASKLHMPCYGLQCTKAAPLDSIQSLASYYIDCMKQIQPEGPYRIAGY
5 amiss_FASN_XP_006038234.1      73.5%
GPTVTRLNEVQSTERPLFLIHPIEGSAAVFQALASRLRIPSYGLQCTRAAPLDSIKSLAAYYIDCMKQVQPEGPYRIAGY
6 cpelagica_FASN_KFU85956.1      89.5%
GPTITRLNDVQSTERPLFFVHPIEGSTAVFHTLASRLHMPCYGFQCTKAAPLDSIQSLASYYLGYMKQIQPEGPYRIAGY

                                              2321          .          .          :          .          .
.          .          4 2400
1 ruby_fasn_i12_HQ_rubyallcluste 100.0%
SFGACVAFEMCSQLQAQHK-GSHALNSLFLFDGSHSFVAAYTQRYRAKLSQGNEAALETEALCAFVQQFTGIEYNKLLEI
2 canna_FASN_KFP01543.1          99.1%
SFGACVAFEMCSQLQAQHK-GSHALNSLFLFDGSHSFVAAYTQRYRAKLSQGNEAALETEALCAFVQQFTGIEYNKLLEI
3 hsapiens_fasn_NP_004095.4      65.6%      SYGACVAFEMCSQLQAQQS-
PAPTHNSLFLFDGSPTYVLAYTQSYRAKLTPGCEAEAETEAICFFVQQFTDMEHNRVLEA
4 gallus_FASN_NP_990486.2        89.5%      SFGACVAFEMCSQLQAQQN-
ASHALNSLFLFDGSHSFVAAYTQSYRAKLTQGNEAALETEALCAFVQQFTGIEYNKLLEI
5 amiss_FASN_XP_006038234.1      73.5%
SFGACVAFEMCSQLQAQQQNPSLGSNSLLLFDGSHSYVAAHTQIYKAKMALGSEAEAEAAAMRAFVQQFTGTEYNKLLET
6 cpelagica_FASN_KFU85956.1      89.5%      SFGACVAFEMCSQLQAQQN-
ASHSLNSLFLFDGSHSFVAAYTRSYRAKLSQGNEAALETEALCAFVQQFTGIEYNKLLEI

                                              2401          .          .          .          .          :
.          .          . 2480
1 ruby_fasn_i12_HQ_rubyallcluste 100.0%
LLPLKDLEARVNAAADLITQVHTHINREALCFAATSFYHKLKAADKYIPESKYHGNVTLVRAKSHNEYEEGLGGDYRLSE
2 canna_FASN_KFP01543.1          99.1%
LLPLKDLEARVNAAADLITQVHTHINREALCFAATSFYHKLKAADKYIPESKYHGNVTLVRAKSHNEYEEGLGGDYRLSE
3 hsapiens_fasn_NP_004095.4      65.6%
LLPLKGLEERVAAAVDLIIKSHQGLDRQELSFAARSFYYKLRAAEQYTPKAKYHGNVMLLRAKTGGAYGEDLGADYNLSQ
4 gallus_FASN_NP_990486.2        89.5%
LLPLEDLEARVNAAADLITQIHKNINREALSFAAASFYHKLKAADKYIPESKYHGNVTLMRAKTHNEYEEGLGGDYRLSE
5 amiss_FASN_XP_006038234.1      73.5%
LLPLQDFDARVSASVDLIARIHRDLNREALSFAAASFYHKLKAADKYVPEFKYHGNVTLLRTKMNDDYGDGLGGDYKLSE
6 cpelagica_FASN_KFU85956.1      89.5%
LLPLKDLEARVNAAADLITQVHTHINREALNFAAASFFHKLKAADKYIPEFKYHGNVTLMRAKSHNEYEEGLGEDYRLSE

                                              2481          .          5          .          .       ] 2527
1 ruby_fasn_i12_HQ_rubyallcluste 100.0%      VCDGKVSVHVVEGDHRTMLEGNGAESIIGIIHSSLAEPRVSVREG--
2 canna_FASN_KFP01543.1          99.1%        VCDGKVSVHVVEGDHRTMLEGNGAESIIGIIHSSLAEPRVSVREG--
3 hsapiens_fasn_NP_004095.4      65.6%        VCDGKVSVHVIEGDHRTLLEGSGLESIISIIHSSLAEPRVSVREG--
4 gallus_FASN_NP_990486.2        89.5%        VCDGKVSVHIIEGDHRTLLEGDGVESIIGIIHGSLAEPRVSVREG--
5 amiss_FASN_XP_006038234.1      73.5%        VCDGKISVHVIEGDHRTFLEADGIETVVGIIHSSLAEPRVSVREG--
```

```
6 cpelagica_FASN_KFU85956.1       89.5%      VCDGKVSVHVVEGDHRTLLEGDGAESIIGIIHSSLAEPRVSVRE---
```

MView 1.60.1, Copyright © 1997-2015 Nigel P. Brown

```
Reference sequence (1): ruby_gpam_i9_HQ_rubyallcluster
Identities normalised by aligned length.
Colored by: identity + property

                                            1 [         .         .         .         .         :
.         .         . 80

1 ruby_gpam_i9_HQ_rubyallcluster 100.0%     --------------------------------
-MDETALSLGTIDVSYLSTSAECSISRCKHSSEEWGECNSRPTLFRSAT
2 canna_gpam_XP_008488364.1       99.9%     ---------------------------------
-DETALSLGTIDVSYLSTSAECSISRCKHSSEEWGECNSRPTLFRSAT
3 cpelagica_gpam_XP_010006217.1   95.3%     ---------------------------------
-DETALSLGTIDVSYLSTSAECGISRCKHSSEEWGECNSRPPLFXXXX
4 hsapiens_gpam_NP_001231878.1    80.6%     ---------------------------------
-DESALTLGTIDVSYLPHSSEYSVGRCKHTSEEWGECGFRPTIFRSAT
5 gallus_gpam_XP_015144456.1      94.1%     ---------------------------------
-DETALSLGTIDVSYLSTSAECSVSRCKHSNEEWGECNSRPTLFRSAT
6 amiss_gpam_KYO40569.1           84.6%
LIGGGNGVSGAGLRSVRTATCESHGSKFNSFVMDEAALSLGTIDVSYLSTSTEYSVGRCKHPSEEWGECNSRPTVFRSAT

                                           81           .         1         .         .         .
.         :         . 160
1 ruby_gpam_i9_HQ_rubyallcluster 100.0%
LRWKEALLSRKRPFVGRCCYVCTPQSRDNFFNASIPSLGLRNVIYINETHTRYRGWLARRLCYVLFVQERDVHKGMFAKN
2 canna_gpam_XP_008488364.1       99.9%
LKWKEALLSRKRPFVGRCCYVCTPQSRDNFFNASIPSLGLRNVIYINETHTRYRGWLARRLCYVLFVQERDVHKGMFAKN
3 cpelagica_gpam_XP_010006217.1   95.3%
FKWKETLLSRKRPFVGRCCYVCTPQSRDNFFNASIPSLGLRNVIYINETHTRYRGWLARRLCYVLFVQERDVHKGMFAKN
4 hsapiens_gpam_NP_001231878.1    80.6%
LKWKESLMSRKRPFVGRCCYSCTPQSWDKFFNPSIPSLGLRNVIYINETHTRHRGWLARRLSYVLFIQERDVHKGMFATN
5 gallus_gpam_XP_015144456.1      94.1%
LKWKET-LSRKRPFVGRCCYVCTPQSRDNFFNASIPSLGLRNVIYINETHTRYRGWLARRLCYVLFVLERDVHKGMFAKN
6 amiss_gpam_KYO40569.1           84.6%
LKWKETLLSRKRPFVGRCCYVCTPQSRDKLFNASIPSLGLRNVIYINETHTRYRGWLARRLCYVLFVQERDVHKGMFAKN

                                          161           .         .         .         2         .
.         .         . 240
1 ruby_gpam_i9_HQ_rubyallcluster 100.0%
LTENVLNNSRVQKAIVDEASEPSTPGSFAQTDPKAISKVKKKARKILQEMVANVSPALIRLTGWVLLKLFNSFFWNIQIH
2 canna_gpam_XP_008488364.1       99.9%
LTENVLNNSRVQKAIVDEASEPSTPGSFAQTDPKAISKVKKKARKILQEMVANVSPALIRLTGWVLLKLFNSFFWNIQIH
3 cpelagica_gpam_XP_010006217.1   95.3%
LTENVLNNSRVQKAIVDEASEPSTPGSFAQTDPKAINKVRKKARKILQEMVANVSPALIRLTGWVLLKLFNSFFWNIQIH
4 hsapiens_gpam_NP_001231878.1    80.6%
VTENVLNSSRVQEAIAEVAAELNPDGS-AQQQSKAVNKVKKKAKRILQEMVATVSPAMIRLTGWVLLKLFNSFFWNIQIH
5 gallus_gpam_XP_015144456.1      94.1%
LTENVLNSSRVQKAIVDEASEPSVPGSFAQMDPKAINKVKKKARKILQEMVANVSPALIRLTGWVLLKLFNSFFWNIQIH
6 amiss_gpam_KYO40569.1           84.6%
LTENVLNSGRVQKAIVEEATETSTSGSFAQVDPKAISKVKKKARKILQEMVATVSPALIRLTGWVLLKLFNSFFWNVQIH

                                          241           :         .         .         .         .
3         .         . 320
1 ruby_gpam_i9_HQ_rubyallcluster 100.0%
RGQIEMVKAATEMNLPLIFLPVHKSHIDYLLLTFILFCHNIKAPYIAAGNNLNIPIFSTLIRKLGGFFIRRKLDQSPDGR
2 canna_gpam_XP_008488364.1       99.9%
RGQIEMVKAATEMNLPLIFLPVHKSHIDYLLLTFILFCHNIKAPYIAAGNNLNIPIFSTLIRKLGGFFIRRKLDQSPDGR
3 cpelagica_gpam_XP_010006217.1   95.3%
RGQIEMVKAATEMNLPLIFLPVHKSHIDYLLLTFILFCHNIKAPYIAAGNNLNIPIFSTLIRKLGGFFIRRKLDQSPDGR
4 hsapiens_gpam_NP_001231878.1    80.6%
KGQLEMVKAATETNLPLLFLPVHRSHIDYLLLTFILFCHNIKAPYIASGNNLNIPIFSTLIHKLGGFFIRRRLDETPDGR
5 gallus_gpam_XP_015144456.1      94.1%
RGQIEMVKAATEMNLPLIFLPVHKSHIDYLLLTFILFCHNIKAPYIAAGNNLNIPIFSTLIRKLGGFFIRRKLDQSSDGR
6 amiss_gpam_KYO40569.1           84.6%
KGQLEMVKAATEMNLPLIFLPVHKSHIDYLLLTFILFCHNIKAPYIAAGNNLNIPIFSTLIRNLGGFFIRRKLDENTNGR

                                          321           .         .         :         .         .
.         .         4 400
1 ruby_gpam_i9_HQ_rubyallcluster 100.0%
KDFLYRALLYVHIEELLRQQQFLEIFLEGTRSRSGKTAGARAGLLSVVVDALFSNATPDVLIIPVGISYDRIIEGHYNSE
2 canna_gpam_XP_008488364.1       99.9%
KDFLYRALLYVHIEELLRQQQFLEIFLEGTRSRSGKTAGARAGLLSVVVDALFSNATPDVLIIPVGISYDRIIEGHYNSE
3 cpelagica_gpam_XP_010006217.1   95.3%
KDFLYRALLYVHIEELLRQQQFLEIFLEGTRSRSGKTSGARAGLLSVVVDALFSNATPDVLIIPVGISYDRIIEGHYNSE
4 hsapiens_gpam_NP_001231878.1    80.6%
KDVLYRALLHGHIVELLRQQQFLEIFLEGTRSRSGKTSCARAGLLSVVVDTLSTNVIPDILIIPVGISYDRIIEGHYNGE
5 gallus_gpam_XP_015144456.1      94.1%
KDFLYRALLYVHIEELLRQQQFLEIFLEGTRSRSGKTSSPRAGLLSVVVDALFSNATPDVLIIPVGISYDRIIEGHYNSE
```

```
6 amiss_gpam_KYO40569.1          84.6%
KDALYRALLYVHIEELLRQQQFLEIFLEGTRSRSGKTSNARAGLLSVVVDALFANATPDVLIIPVGISYDRIIEGHYNSE

                                              401            .          .          .          .          :
.          .          . 480
1 ruby_gpam_i9_HQ_rubyallcluster 100.0%
QLGKPKKNESLWSVARGVFRMLRKNYGCVRVDFAQPFSLKEYVNSQSQKPMPAPLSLEQALLPAILPSRPNDTVHESSEA
2 canna_gpam_XP_008488364.1       99.9%
QLGKPKKNESLWSVARGVFRMLRKNYGCVRVDFAQPFSLKEYVNSQSQKPMPAPLSLEQALLPAILPSRPNDTVHESSEA
3 cpelagica_gpam_XP_010006217.1   95.3%
QLGKPKKNESLWSIARGVFRMLRKNYGCVRVDFAQPFSLKEYINSQSQKSVPAPLSLEQALLPAILPSRPNDMVDEGAEA
4 hsapiens_gpam_NP_001231878.1    80.6%
QLGKPKKNESLWSVARGVIRMLRKNYGCVRVDFAQPFSLKEYLESQSQKPVSALLSLEQALLPAILPSRPSDAADEGRDT
5 gallus_gpam_XP_015144456.1      94.1%
QLGKPKKNESLWSIARGVFRMLRKNYGCVRVDFAQPFSLKEYVNSQSQKTVPAPLSLEQALLPAILPSRPNDTVDEGTEA
6 amiss_gpam_KYO40569.1           84.6%
QLGKPKKNESLWSIARGVFRMLRKNYGCVRVDFAQPFSLKEFVDSQHQKPAPASLSLEQALLPALLPSRPNDVEEEGTEA

                                              481            .          5          .          .          .
.          :          . 560
1 ruby_gpam_i9_HQ_rubyallcluster 100.0%
SQPNSRDITSEPFRRELISNLAEHVLFTANKSCAVMSTYIVACLLLYRHRKGTDLSRLVEDFFSMKEEVLARDYDLGFSG
2 canna_gpam_XP_008488364.1       99.9%
SQPNSRDITSEPFRRELISNLAEHVLFTANKSCAVMSTYIVACLLLYRHRKGTDLSRLVEDFFSMKEEVLARDYDLGFSG
3 cpelagica_gpam_XP_010006217.1   95.3%
SLPNSRDITSEPFRRELISNLAEHILFTANKSCAVMSTHIVACLLLYRHRQGTDLSRLVEDFFSMKEEVLARDFDLGFSG
4 hsapiens_gpam_NP_001231878.1    80.6%
SINESRNATDESLRRRLIANLAEHILFTASKSCAIMSTHIVACLLLYRHRQGIDLSTLVEDFFVMKEEVLARDFDLGFSG
5 gallus_gpam_XP_015144456.1      94.1%
SLPNSKDITSEPYRRELIANLAEHILFTANKSCAVMSTHIVACLLLYRHRQGTDLSRLVEDFFSMKEEVLARDFDLGFSG
6 amiss_gpam_KYO40569.1           84.6%
ALPNSRDISNEHFRRQLIANLAEHILFTANKSCAVMSTHIVACLLLYRHRQGIDLSKLVEDFFSMKEEVLARDFDLGFSG

                                              561            .          .          .          6          .
.          .          . 640
1 ruby_gpam_i9_HQ_rubyallcluster 100.0%
NSDDVVMHAIHLLGNCVNITNTSRNNEFFITPSTEIPAVFELNFYSNGVLHVFIKEAVIACSLHAIQSRRYRNGTNSASP
2 canna_gpam_XP_008488364.1       99.9%
NSDDVVMHAIHLLGNCVNITNTSRNNEFFITPSTEIPAVFELNFYSNGVLHVFIKEAVIACSLHAIQSRRYRNGTNSASP
3 cpelagica_gpam_XP_010006217.1   95.3%
NSDDVVMHAIHLLGNCVNITHASRNNEFFITPSTKIPAVFELNFYSNGVLHVFIKEAVIACSLHAIQSRRCRNGTSGASS
4 hsapiens_gpam_NP_001231878.1    80.6%
NSEDVVMHAIQLLGNCVTITHTSRNDEFFITPSTTVPSVFELNFYSNGVLHVFIMEAIIACSLYAVLNKRGLGGPTSTPP
5 gallus_gpam_XP_015144456.1      94.1%
NSDDVVMHAIHLLGNCVNITNTSRNNEFFITPSTTIPAVFELNFYSNGILHVFIKEAVIACSLHAVQSKRFRNGTNGASP
6 amiss_gpam_KYO40569.1           84.6%
NSEDVVMHAIHLLGNCVTITNTSRNNEFFITPSTTIPAVFELNFYSNGVLHVFINEAIIACSLHAVQSKRLRNGINGTSP

                                              641            :          .          .          .          .
7          .          . 720
1 ruby_gpam_i9_HQ_rubyallcluster 100.0%
GLISQEHLVRKAASLCYLLSNEFTVSLPCQVIYQVCHESVERLIQYGILLVAEQDDQEDVSPSLAEQQWDKKLPEPLSWR
2 canna_gpam_XP_008488364.1       99.9%
GLISQEHLVRKAASLCYLLSNEFTVSLPCQVIYQVCHESVERLIQYGILLVAEQDDQEDVSPSLAEQQWDKKLPEPLSWR
3 cpelagica_gpam_XP_010006217.1   95.3%
SLISQEHLVRKAASLCYLLSNEFTVSLPCQVIYQVCHESVERLIQYGILLVAEQDDQEDVSPSLTEQQWDKKLPEPLSWR
4 hsapiens_gpam_NP_001231878.1    80.6%
NLISQEQLVRKAASLCYLLSNEGTISLPCQTFYQVCHETVGKFIQYGILTVAEHDDQEDISPSLAEQQWDKKLPEPLSWR
5 gallus_gpam_XP_015144456.1      94.1%
SLISQEHLVRKAASLCYLLSNEFTVSLPCQLIYQVCHEAVEKLIQYGILLVAEQDDQEDVSPSLTEQQWNKKLPEPLTWR
6 amiss_gpam_KYO40569.1           84.6%
NMISQEQLIRKAASLCYLLSNEGPVSLPCQMLYQVCHEVVERFIQYGILLVAEQDDQEDLSPSLTEQQWDKKLPEPLSWR

                                              721            .          .          :          .          .
.          .          8 800
1 ruby_gpam_i9_HQ_rubyallcluster 100.0%
SDEEDEDSDFGEEQRDCYLKVSQSKEHQQYITFLQRLLGPLLEAYSSAVIFIHNFSGPVSESEYIQKLHRHLISRTEKKV
2 canna_gpam_XP_008488364.1       99.9%
SDEEDEDSDFGEEQRDCYLKVSQSKEHQQYITFLQRLLGPLLEAYSSAVIFIHNFSGPVSESEYIQKLHRHLISRTEKKV
3 cpelagica_gpam_XP_010006217.1   95.3%
SDEEDEDSDFGEEQRDCYLKVSQSQEHQQYITFLQRLLGPLLEAYSSAVIFVHNFSGPVSESEYLQKLHRHLISRTEKNV
4 hsapiens_gpam_NP_001231878.1    80.6%
SDEEDEDSDFGEEQRDCYLKVSQSKEHQQFITFLQRLLGPLLEAYSSAAIFVHNFSGPVPEPEYLQKLHKYLITRTERNV
5 gallus_gpam_XP_015144456.1      94.1%
SDEEDEDSDFGEEQRDCYLKVSQSQEHQQYITFLQRLLGPLLEAYSSAVIFVHNFSGPVSESEYLQKLHRHLINRTEKNV
6 amiss_gpam_KYO40569.1           84.6%
SDEEDEDSDFGEEQRDCYLKVSQSQEHQQYITFLQRLLGPLLEAYSSAAAFIHNFSGPVSEREYLQKLHKYLISRTEKNV

                                              801            .          .          .          .          :
.  ] 863
1 ruby_gpam_i9_HQ_rubyallcluster 100.0%
AVYAESATYSHVKNAVKVFKEIGVFNQTKQKRDTILELGTTFLPQRNRQKLLEFIMSFMVL--
2 canna_gpam_XP_008488364.1       99.9%
AVYAESATYSHVKNAVKVFKEIGVFNQTKQKRDTILELGTTFLPQRNRQKLLEFIMSFMVL--
3 cpelagica_gpam_XP_010006217.1   95.3%
```

```
AVYAESATYSHVKNAVKVFKEIGVFNQTKQRRDTILELGTTFLPQRNRQKLLEFIMSFMVL--
4 hsapiens_gpam_NP_001231878.1    80.6%
AVYAESATYCLVKNAVKMFKDIGVFKETKQKRVSVLELSSTFLPQCNRQKLLEYILSFVVL--
5 gallus_gpam_XP_015144456.1      94.1%
AVYAESATYSHVKNAVKVFKEIGVFIQTNQKRDTILELSTTFLPQRNRQKLLEFIMSFMVL--
6 amiss_gpam_KYO40569.1           84.6%
AMYAESATYCLVKNAVKVFKDMGVFKETKQ-RETILELSSTFLPQHNRQKLLEFILSFVVL--
```

MView 1.60.1, Copyright © 1997-2015 Nigel P. Brown

```
Reference sequence (1): ruby_gpat4_i9_HQ_rubyallcluste
Identities normalised by aligned length.
Colored by: identity + property

                                          1 [        .         .         .         .         :
.         .         . 80
1 ruby_gpat4_i9_HQ_rubyallcluste 100.0%
VVNLLGISITVLFTLLLVFIIVPAIFGVSFGIRKVYMKTLLKIFQWATLRIERGAKEKNHPLYKPYVNGIIAKEPTSLEE
2 canna_gpat4_KFP06716.1        100.0%     -------------------------------------------------------
----------------------
3 cpelagica_gpat4_KFU86340.1      99.5%     -------------------------------------------------------
----------------------
4 hsapiens_gpat4_NP_848934.1      80.4%     -------------------------------------------------------
----------------------
5 gallus_gpat4_XP_015152890.1     90.4%     -------------------------------------------------------
----------------------
6 amiss_gpat4_KYO28476.1          80.5%     -------------------------------------------------------
----------------------

                                         81          .         1         .         .         .
.         :         . 160
1 ruby_gpat4_i9_HQ_rubyallcluste 100.0%
EIKEIRRSGSGKALDTPEFELSDIFYFCRKGIETIMDDEVTKRFSAEELESWNLLSRTNYNFQYISLRLTVLWGLGVLIR
2 canna_gpat4_KFP06716.1        100.0%     -------------------------------------------------------
----------------------
3 cpelagica_gpat4_KFU86340.1      99.5%     -------------------------------------------------------
----------------------
4 hsapiens_gpat4_NP_848934.1      80.4%     -------------------------------------------------------
----------------------
5 gallus_gpat4_XP_015152890.1     90.4%     -------------------------------------------------------
----------------------
6 amiss_gpat4_KYO28476.1          80.5%     -------------------------------------------------------
----------------------

                                        161          .         .         .         2         .
.         .         . 240
1 ruby_gpat4_i9_HQ_rubyallcluste 100.0%
YCFLLPLRIALAFTGISLLVTGTTVVGYLPNGRCKEFLSKHVHLMCYRICVRALTAIITYHDRENRPRNGGICVANHTSP
2 canna_gpat4_KFP06716.1        100.0%     -------------------------------------------------------
----------------------
3 cpelagica_gpat4_KFU86340.1      99.5%     -------------------------------------------------------
----------------------
4 hsapiens_gpat4_NP_848934.1      80.4%     -------------------------------------------------------
----------------------
5 gallus_gpat4_XP_015152890.1     90.4%     -------------------------------------------------------
----------------------
6 amiss_gpat4_KYO28476.1          80.5%     -------------------------------------------------------
----------------------

                                        241          :         .         .         .         .
3         .         . 320
1 ruby_gpat4_i9_HQ_rubyallcluste 100.0%
IDVIILASDGYYAMVGQIHGGLMGVIQRAMVKACPHVWFERSEVKDRHLVARRLTEHVQDKSKLPILIFPEGTCINNTSV
2 canna_gpat4_KFP06716.1        100.0%     -------------------------------------------------------
----------------------
3 cpelagica_gpat4_KFU86340.1      99.5%     -------------------------------------------------------
----------------------
4 hsapiens_gpat4_NP_848934.1      80.4%     -------------------------------------------------------
----------------------
5 gallus_gpat4_XP_015152890.1     90.4%     -------------------------------------------------------
----------------------
6 amiss_gpat4_KYO28476.1          80.5%     -------------------------------------------------------
----------------------

                                        321          .         .         :         .         .
.         .         4 400
1 ruby_gpat4_i9_HQ_rubyallcluste 100.0%
MMFKKGSFEIGATVYPVAIKYDPQFGDAFWNSSKYGMVTYLLRMMTSWAIVCSVWYMPPMTRQPEEDAVQFANRVKSAIA
2 canna_gpat4_KFP06716.1        100.0%     -------------------------------------------------------
----------------------
```

```
3 cpelagica_gpat4_KFU86340.1      99.5%     ------------------------------------------------------------
-----------------------
4 hsapiens_gpat4_NP_848934.1      80.4%     ----------------------------------------FLL--------------
LP------------FDS-------
5 gallus_gpat4_XP_015152890.1     90.4%     ------------------------------------------------------------
-----------------------
6 amiss_gpat4_KYO28476.1          80.5%     ----------------------------------------FLL--------------
LP------------FDS-------

                                            401          .          .          .          .          :
.          .          . 480
1 ruby_gpat4_i9_HQ_rubyallcluste 100.0%
RQGGLVDLLWDGGLKREKVKDTFKEEQQKLYSKMIVGNVVNLLGISITVLFTLLLVFIIVPAIFGVSFGIRKVYMKTLLK
2 canna_gpat4_KFP06716.1          100.0%     -------------------------------------
-VVNLLGISITVLFTLLLVFIIVPAIFGVSFGIRKVYMKTLLK
3 cpelagica_gpat4_KFU86340.1      99.5%     -------------------------------------
-VVNLLGISITVLFTLLLVFIIVPAIFGVSFGIRKVYMKTLLK
4 hsapiens_gpat4_NP_848934.1      80.4%     -------------------------------------
LIVNLLGISLTVLFTLLLVFIIVPAIFGVSFGIRKLYMKSLLK
5 gallus_gpat4_XP_015152890.1     90.4%     ----FLLLPFDS-------------------------
LVVNLLGISITVLFTLLLVFIIVPAIFGVSFGIRKVYMKTLLK
6 amiss_gpat4_KYO28476.1          80.5%     -------------------------------------
LVANLLGISLTVLFTLLLVFIIVPAVFGVSFGIRRLYMKTLLK

                                            481          .          5          .          .          .
.          :          . 560
1 ruby_gpat4_i9_HQ_rubyallcluste 100.0%
IFQWATLRIERGAKEKNHPLYKPYVNGIIAKEPTSLEEEIKEIRRSGSGKALD-TPEFELSDIFYFCRKGIETIMDDEVT
2 canna_gpat4_KFP06716.1          100.0%
IFQWATLRIERGAKEKNHPLYKPYVNGIIAKEPTSLEEEIKEIRRSGSGKALD-TPEFELSDIFYFCRKGIETIMDDEVT
3 cpelagica_gpat4_KFU86340.1      99.5%
IFQWATLRIERGAKEKNHPLYKPYVNGIIAKEPTSLEEEIKEIRRSGSGKALD-TPEFELSDIFYFCRKGIETIMDDEVT
4 hsapiens_gpat4_NP_848934.1      80.4%
IFAWATLRMERGAKEKNHQLYKPYTNGIIAKDPTSLEEEIKEIRRSGSSKALDNTPEFELSDIFYFCRKGMETIMDDEVT
5 gallus_gpat4_XP_015152890.1     90.4%     IFQWATLRIERGAKEKNHPLYKPYVNGIIAKEPTSLEEEIKEIRRSGSGKALD-
APEFELSDIFYFCRKGIETIMDDEVT
6 amiss_gpat4_KYO28476.1          80.5%     IFQWATLRIERGAKEKNHPVYKPYANGIIAKEPMSLEEEIKEIRRSGSSKALD-
APEFELSDIFYFCRKGIETIMDDEVT

                                            561          .          .          .          6          .
.          .          . 640
1 ruby_gpat4_i9_HQ_rubyallcluste 100.0%
KRFSAEELESWNLLSRTNYNFQYISLRLTVLWGLGVLIRYCFLLPLRIALAFTGISLLVTGTTVVGYLPNGRCKEFLSKH
2 canna_gpat4_KFP06716.1          100.0%
KRFSAEELESWNLLSRTNYNFQYISLRLTVLWGLGVLIRYCFLLPLRIALAFTGISLLVTGTTVVGYLPNGRCKEFLSKH
3 cpelagica_gpat4_KFU86340.1      99.5%
KRFSAEELESWNLLSRTNYNFQYISLRLTVLWGLGVLIRYCFLLPLRIALAFTGISLLVTGTTVVGYLPNGRCKEFLSKH
4 hsapiens_gpat4_NP_848934.1      80.4%
KRFSAEELESWNLLSRTNYNFQYISLRLTVLWGLGVLIRYCFLLPLRIALAFTGISLLVVGTTVVGYLPNGRFKEFMSKH
5 gallus_gpat4_XP_015152890.1     90.4%
KRFSAEELESWNLLSRTNYNFHYISLRLTVLWGLGVLIRYCFLLPLRIALAFTGISLLVTGTTVVGYLPNGRCKEFLSKH
6 amiss_gpat4_KYO28476.1          80.5%
KRFSAEELESWNLLSRTNYNFQYISLRLTVLWGLGVLIRYCFLLPLRIALAFTGVSLLVIGTSVVGLLPNGRCKEFLSKH

                                            641          :          .          .          .          .
7          .          . 720
1 ruby_gpat4_i9_HQ_rubyallcluste 100.0%
VHLMCYRICVRALTAIITYHDRENRPRNGGICVANHTSPIDVIILASDGYYAMVGQIHGGLMGVIQRAMVKACPHVWFER
2 canna_gpat4_KFP06716.1          100.0%
VHLMCYRICVRALTAIITYHDRENRPRNGGICVANHTSPIDVIILASDGYYAMVGQIHGGLMGVIQRAMVKACPHVWFER
3 cpelagica_gpat4_KFU86340.1      99.5%
VHLMCYRICVRALTAIITYHDRENRPRNGGICVANHTSPIDVIILASDGYYAMVGQIHGGLMGVIQRAMVKACPHVWFER
4 hsapiens_gpat4_NP_848934.1      80.4%
VHLMCYRICVRALTAIITYHDRENRPRNGGICVANHTSPIDVIILASDGYYAMVGQVHGGLMGVIQRAMVKACPHVWFER
5 gallus_gpat4_XP_015152890.1     90.4%
VHLMCYRICVRALTAIITYHDRENRPRNGGICVANHTSPIDVIILASDGYYAMVGQVHGGLMGVIQRAMVKACPHVWFER
6 amiss_gpat4_KYO28476.1          80.5%
VHLMCYRICVRALTAIITYHDRENRPRNGGICVANHTSPIDVIILASDGYYAMVGQIHGGLMGVIQRAVVKACPHVWFER

                                            721          .          .          :          .          .
.          .          8 800
1 ruby_gpat4_i9_HQ_rubyallcluste 100.0%
SEVKDRHLVARRLTEHVQDKSKLPILIFPEGTCINNTSVMMFKKGSFEIGATVYPVAIKYDPQFGDAFWNSSKYGMVTYL
2 canna_gpat4_KFP06716.1          100.0%
SEVKDRHLVARRLTEHVQDKSKLPILIFPEGTCINNTSVMMFKKGSFEIGATVYPVAIKYDPQFGDAFWNSSKYGMVTYL
3 cpelagica_gpat4_KFU86340.1      99.5%
SEVKDRHLVAKRLTEHVQDKSKLPILIFPEGTCINNTSVMMFKKGSFEIGATVYPVAIKYDPQFGDAFWNSSKYGMVTYL
4 hsapiens_gpat4_NP_848934.1      80.4%
SEVKDRHLVAKRLTEHVQDKSKLPILIFPEGTCINNTSVMMFKKGSFEIGATVYPVAIKYDPQFGDAFWNSSKYGMVTYL
5 gallus_gpat4_XP_015152890.1     90.4%
SEVKDRHLVAKRLTEHVQDKSKLPILIFPEGTCINNTSVMMFKKGSFEIGATVYPVAIKYDPQFGDAFWNSSKYGMVTYL
6 amiss_gpat4_KYO28476.1          80.5%
SEVKDRHLVAKRLTEHVQDKSKLPILIFPEGTCINNTSVMMFKKGSFEIGATVYPVAIKYDPQFGDAFWNSSKYGMVTYL

                                            801          .          .          .          .          :
```

```
 .         .         . 880
 1 ruby_gpat4_i9_HQ_rubyallcluste 100.0%
 LRMMTSWAIVCSVWYMPPMTRQPEEDAVQFANRVKSAIARQGGLVDLLWDGGLKREKVKDTFKEEQQKLYSKMIVGN---
 2 canna_gpat4_KFP06716.1         100.0%
 LRMMTSWAIVCSVWYMPPMTRQPEEDAVQFANRVKSAIARQGGLVDLLWDGGLKREKVKDTFKEEQQKLYSKMIVGN---
 3 cpelagica_gpat4_KFU86340.1      99.5%
 LRMMTSWAIVCSVWYLPPMTRQPEEDAVQFANRVKSAIARQGGLVDLLWDGGLKREKVKDTFKEEQQKLYSKMIVGN---
 4 hsapiens_gpat4_NP_848934.1      80.4%
 LRMMTSWAIVCSVWYLPPMTREADEDAVQFANRVKSAIARQGGLVDLLWDGGLKREKVKDTFKEEQQKLYSKMIVGNHKD
 5 gallus_gpat4_XP_015152890.1     90.4%
 LRMMTSWAIVCSVWYLPPMTRQCDEDAVQFANRVKSAIARQGGLVDLLWDGGLKREKVKDTFKEEQQKLYSKMIVGNHED
 6 amiss_gpat4_KYO28476.1          80.5%
 LRMMTSWAIVCSVWYLPPMTRQPDEDAVQFANRVKSAIARQGGLVDLLWDGGLKREKVKDAFKEEQQKLYSKMISGNHED

                                          881      ] 886
 1 ruby_gpat4_i9_HQ_rubyallcluste 100.0%    ------
 2 canna_gpat4_KFP06716.1         100.0%    ------
 3 cpelagica_gpat4_KFU86340.1      99.5%    ------
 4 hsapiens_gpat4_NP_848934.1      80.4%    RSRS--
 5 gallus_gpat4_XP_015152890.1     90.4%    RSRS--
 6 amiss_gpat4_KYO28476.1          80.5%    RSRS--
```

MView 1.60.1, Copyright © 1997-2015 Nigel P. Brown

```
 Reference sequence (1): ruby_lpin1_i9_HQ_rubyallcluste
 Identities normalised by aligned length.
 Colored by: identity + property


                                          1 [         .         .         .         .         :
 .         .         . 80


 1 ruby_lpin1_i9_HQ_rubyallcluste 100.0%    -------------------------------------
 -QVVWWWSAEVQTMNYVGQLAGQVFVTVKELYKGLNPATLSGCI
 2 canna_lpin1_XP_008493249.1      95.9%    ---------
 SSEEEDQPETPTGSPWSWIPLMKDPGWIRNVWTRNLNVQTMNYVGQLAGQVFVTVKELYKGLNPATLSGCI
 3 cpelagica_lpin1_XP_010000011.1  88.6%    ---------
 SSEEEDQPETPTGSPWSWIPLMKDPGWIRSVWTRNLNVQTMNYVGQLAGQVFVTVKELYKGLNPATLSGCI
 4 hsapiens_lpin1_NP_663731.1      74.8%    ---------------------------------------------------
 -NYVGQLAGQVFVTVKELYKGLNPATLSGCI
 5 amiss_lpin1_XP_006258935.1      80.4%    ---------------------------------------------------
 -NYVGQLAGQVFVTVKELYKGLNPATLSGCI
 6 gallus_lpin1_XP_015131575.1     82.0%
 MGEKDHFKMSSEDEEQPESPPGSPWSWILLMKDPGWIRNVWTRNLNVQTMNYVGQLAGQVFVTVKELYKGLNPATLSGCI

                                         81          .         1         .         .         .
 .         :         . 160
 1 ruby_lpin1_i9_HQ_rubyallcluste 100.0%
 DIIVVRQPDGNLQCSPFHVRFGKMGVLRSREKVVDIEINGEAVDLHMKLGDNGEAFFVQEMDNDQEVIPFHLATSPILSE
 2 canna_lpin1_XP_008493249.1      95.9%
 DIIVVRQPDGNLQCSPFHVRFGKMGVLRSREKVVDIEINGEAVDLHMKLGDNGEAFFVQEMDNDQEVIPFHLATSPILSE
 3 cpelagica_lpin1_XP_010000011.1  88.6%
 DIIVVRQPDGNLQCSPFHVRFGKMGVLRSREKVVDIEINGEAVDLHMKLGDNGEAFFVQEMDNDQEVIPYHLATSPIRSE
 4 hsapiens_lpin1_NP_663731.1      74.8%
 DIIVIRQPNGNLQCSPFHVRFGKMGVLRSREKVVDIEINGESVDLHMKLGDNGEAFFVQETDNDQEVIPMHLATSPILSE
 5 amiss_lpin1_XP_006258935.1      80.4%
 DIIVVRQPDGNLQCSPFHVRFGKMGVLRSREKVVDIEINGESVDLHMKLGDNGEAFFVQETDNDQEVIPHHLSTSPILSE
 6 gallus_lpin1_XP_015131575.1     82.0%
 DIIVVRQPDGNLQCSPFHVRFGKMGVLRSREKVVDIEINGEAVDLHMKLGDNGEAFFVQEMDNNQEVIPYHLSTSPILSE

                                        161          .         .         .         2         .
 .         .         . 240
 1 ruby_lpin1_i9_HQ_rubyallcluste 100.0%
 GSALMEVQLKRNSIDRIRNLDTSASSQLSPQAYGSQPLTETSPSCSSVKKRRKKRRKSTHKIDSLKREGNGDTSEDEDMF
 2 canna_lpin1_XP_008493249.1      95.9%
 GSALMEVQLKRNSIDRIRNLDTSASSQLSPQAYGSQPLTETSPSCSSVKKRRKKRRKSTHKIDSLKREGNGDTSEDEDMF
 3 cpelagica_lpin1_XP_010000011.1  88.6%
 GTALMEAQLKRNSIDRIRSLDTSASSQLSPQGHGSQPSAETSAACSSVKKRRKKRRKSTHKIDSLKREENGDTSEDEDMF
 4 hsapiens_lpin1_NP_663731.1      74.8%    GASRMECQLKRGSVDRMRGLDPSTPAQVIAP-------
 SETPSSSSVVKKRRKRRRKS--QLDSLKRDDNMNTSEDEDMF
 5 amiss_lpin1_XP_006258935.1      80.4%
 GVALMESQRKRNSIDWVRSLDSSGSSQAQTQVHVSQPAIETSSVCGSVKKRRKKRRKSTHKIDSLKREDNGDTSEDEDMF
 6 gallus_lpin1_XP_015131575.1     82.0%
 GTALMEAQLKRNSIDRIRNLDSSVSSQVPPQAHGSQPGTETSPACSSVKKRRKKRRKSTHKIDSLKREDIGDTSEDEDMF

                                        241          :         .         .         .         .
 3         .         . 320
 1 ruby_lpin1_i9_HQ_rubyallcluste 100.0%    PIEISSEEEKEPLDDSRIPVPDA-
 -FLDDASVMKAPAVTTFSQSASYPNSDGEWSSLQS---------------------
 2 canna_lpin1_XP_008493249.1      95.9%    PIEISSEEEKEPLDDSRIPVPDA-
 -FLDDASVMKAPAVTTFSQSASYPNSDGEWSSLQS---------------------
 3 cpelagica lpin1 XP 010000011.1  88.6%    PIEISSDEEKEPLDSSRVPVPDV--
```

```
LVDDVSDRKAPAVSTFSQSASYPNSDGEWSPLQS---------------------
4 hsapiens_lpin1_NP_663731.1      74.8%
PIEMSSDEAMELLESSRTLPNDIPPFQDDIPEENLSLAVIYPQSASYPNSDREWSPTPS---------------------
5 amiss_lpin1_XP_006258935.1      80.4%       PIEISSDEENDLLDNSRKSVPDV--
CVDEISEKNTSALNTLSQSAPYPHSDGEWSPLQSQPIECTGPSSHLTVPADGGLS
6 gallus_lpin1_XP_015131575.1     82.0%       PIEISSEEEKEQLDNSRILVPDV-
-FVDEVSDIKAPAVSAYSQSSSYPRSDGEWSPIQSKPIDYTGQSSLLTVPADGGLS

                                          321          .          .          :          .          .
.         .         4 400
1 ruby_lpin1_i9_HQ_rubyallcluste 100.0%       --------------
-LSGSRPPTPQSDSELVSKPTDRSGSKNNPHMHWAWGELPQAAKASFLLKAKEPS-IVDVNPSEST
2 canna_lpin1_XP_008493249.1      95.9%       --------------
-LSGSRPPTPQSDSELVSKPTDRSGSKNNPHMHWAWGELPQAAKASFLLKAKEPS-IVDVNPSEST
3 cpelagica_lpin1_XP_010000011.1  88.6%       --------------
-LSGSRPPTPQSDSELVSKPVDRSGLKNNPHMHWAWGELPQAAKATSLLKAKEPS-LVDVNPSEST
4 hsapiens_lpin1_NP_663731.1      74.8%       ---------------
PSGSRPSTPKSDSELVSKSTERTGQKN-PEMLWLWGELPQAAKSSSPHKMKESSPLSSRKICDKS
5 amiss_lpin1_XP_006258935.1      80.4%
SSCPHQSSRFPATDSPSGSRPPTPKSDSELISKTLDRGAQKNNPQMHWAWGELPQAAKSTS-LKAKEAG-VMNVNPSEST
6 gallus_lpin1_XP_015131575.1     82.0%
NSCPHQSSHFSPPDSLSGSRPPTPQSDSELVSKPTDRSGLKN-PHMHWAWGELPQATKASSLIKAKEPN-TVDVNPSEST

                                          401          .          .          .          .          :
.         .         . 480
1 ruby_lpin1_i9_HQ_rubyallcluste 100.0%       HFRVIQSSPVEEFGTVAPLPDLGQAGAATADEREPLP-
-VDANKPELESPGAAVAPLSANEELKQSTACSSQPAGKTDSP
2 canna_lpin1_XP_008493249.1      95.9%       HFRVIQSSPVEEFETVAPLPDLGQAGAATADEREPSP--
ADANKPELESPGAAVAPLSANEELKQSTACSSQPAGKTDSP
3 cpelagica_lpin1_XP_010000011.1  88.6%       HFRVIQSSPSEEFNTVSPLPALEQAGATTAEESEPSP--
AETNKPETESPGAAVPPLSANEELKQAAACSAQPAGKTDSP
4 hsapiens_lpin1_NP_663731.1      74.8%       HFQAIHSESSDTFSDQSPT----
LVGGALLDQNKPQTEMQFVNEEDLETLGAAAPLLPMIEELKPPSASVVQTANKTDSP
5 amiss_lpin1_XP_006258935.1      80.4%
HFRVIQSAPAEEFGSVSLSPALGQADTAAPDETEPQPEPAEINQPEPESAGAVAAPLPANEDIKQAASCLAQPVSKTDSP
6 gallus_lpin1_XP_015131575.1     82.0%       HFRVIQSAPIEEFNGVSPLPALGQTDAATADETEPLP--
AETNKPETESAGAAVPSLPANEEIKQAAACSAQAVGKTDSP

                                          481          .          5          .          .          .
.         :         . 560
1 ruby_lpin1_i9_HQ_rubyallcluste 100.0%
SRKKDKRSRHLGADGVYLDDLTDMDPEVAALYFPKNGDN-VQSKNTNDAGPRSASNSPQSVGSSGVDSGAESTSDGIRDL
2 canna_lpin1_XP_008493249.1      95.9%
SRKKDKRSRHLGADGVYLDDLTDMDPEVAALYFPKNGDN-VQSKNTNDAGPRSASNSPQSVGSSGVESGAESTSDGIRDL
3 cpelagica_lpin1_XP_010000011.1  88.6%
SRKKDKRSRHLGADGVYLDDLTDMDPEVAALYFPKNGES-VQSKHPPEAGPWSAHPSPQSVGSSGVESGAESTSDGARDL
4 hsapiens_lpin1_NP_663731.1      74.8%
SRKRDKRSRHLGADGVYLDDLTDMDPEVAALYFPKNGDPSGLAKHASDNGARSANQSPQSVGSSGVDSGVESTSDGLRDL
5 amiss_lpin1_XP_006258935.1      80.4%
FKKKDKRSRHLGADGVYLDDLTDMDPEVAALYFPQNGDNTLHNKNGNENGPWSANQSPQSVGSSGVDSGVESVSDGIRDL
6 gallus_lpin1_XP_015131575.1     82.0%
SRKKDKRSRHLGADGVYLDDLTDMDPEVAALYFPKNGDN-VQNRNTNDTGPRSATHSPQSFGSSGADSGVESTSDGTRDL

                                          561          .          .          .          6          .
.         .         . 640
1 ruby_lpin1_i9_HQ_rubyallcluste 100.0%
PSIAISLCGGLIDNKEITKEEFLEHAVTYQQFVDNPAIIDDPNLVVKIGNKYYNWTTAGPLLLAMQAFQRPLPKATVESI
2 canna_lpin1_XP_008493249.1      95.9%
PSIAISLCGGLIDNKEITKEEFLEHAVTYQQFVDNPAIIDDPNLVVKIGNKYYNWTTAGPLLLAMQAFQRPLPKATVESI
3 cpelagica_lpin1_XP_010000011.1  88.6%
PSIAISLCGGLTDSKEITQEEFLEHAVTYQQFVDNPAIIDDPNLVVKIGNKYYNWTTAGPLLLAMQAFQRPLPKATVESI
4 hsapiens_lpin1_NP_663731.1      74.8%
PSIAISLCGGLSDHREITKDAFLEQAVSYQQFVDNPAIIDDPNLVVKIGSKYYNWTTAAPLLLAMQAFQKPLPKATVESI
5 amiss_lpin1_XP_006258935.1      80.4%
PSIAISLCGGLSDNKEITKDQFLEHAVTYQQFVDNPAIIDDPNLVVKIGNKYYNWTTAAPLLLAMQAFQKPLPKATVESI
6 gallus_lpin1_XP_015131575.1     82.0%
PSIAISLCGGLTDNKEITKEEFLEHAVTYQQFVDNPAIIDDPNLVVKIGNKYYNWTTAGPLLLAMQAFQKPLPKATVESI

                                          641          :          .          .          .          .
7         .         . 720
1 ruby_lpin1_i9_HQ_rubyallcluste 100.0%       MRDKMPKKGGRWWFSWRGRNSTIKEETKPDQGMSGSGL-TGG-SSQMSMAN------
----------RIKDESSSSDEDP
2 canna_lpin1_XP_008493249.1      95.9%       MRDKMPKKGGRWWFSWRGRNSTIKEETKPDQGMSGSGL-TGG-SSQMSMAN------
----------RIKDESSSSDEDP
3 cpelagica_lpin1_XP_010000011.1  88.6%       MRDKMPKKGGRWWFSWRGRNSTIKEEAKAEQGMSGTGL-TGE-SSQLGMAN------
----------RIKDESSSSDEDP
4 hsapiens_lpin1_NP_663731.1      74.8%       MRDKMPKKGGRWWFSWRGRNTTIKEESKPEQCLAGKAHSTGEQPPQLSLAT------
----------RVKHESSSSDEER
5 amiss_lpin1_XP_006258935.1      80.4%       MRDKMPKKGGRWWFSWRGRNSTIKEETKPELGNGLN---TGEESLQLTITK------
----------RIKDESSSSDEDP
6 gallus_lpin1_XP_015131575.1     82.0%       MRDKMPKKGGRWWFSWRGRNSTIKEETKAEQGMSGSRL-
KGEDSSQMTMANRKFRSLSLPRLIFTRNRIKDESSSSDEDP

                                          721          .          .          :          .          .
.         .         8 800
```

```
1 ruby_lpin1_i9_HQ_rubyallcluste 100.0%
RAAKQNLGSLQSNSSHLSLLSGIGYKKTLRLTSDQLKSLKLKNGPNDVTFSVTTQYQGTCRCEGTIYLWNWDEKVIISDI
2 canna_lpin1_XP_008493249.1       95.9%
RAAKQNLGSLQSNSSHLSLLSGIGYKKTLRLTSDQLKSLKLKNGPNDVTFSVTTQYQGTCRCEGTIYLWNWDEKVIISDI
3 cpelagica_lpin1_XP_010000011.1   88.6%
RAAKQNLGSLQTNSSHLSLLPGISYKKTLRLTSDQLKSLKLKNGPNDVTFSVTTQYQGTCRCEGTIYLWNWDEKVIISDI
4 hsapiens_lpin1_NP_663731.1       74.8%      AAAKP-----
-SNAGHLPLLPNVSYKKTLRLTSEQLKSLKLKNGPNDVVFSVTTQYQGTCRCEGTIYLWNWDDKVIISDI
5 amiss_lpin1_XP_006258935.1       80.4%
RAAKQNIGSLQTNSSHLSLLSGISYKKTLRLTSDQLKSLKLKNGPNDVTFSVTTQYQGTCRCEGTIYFWNWDEKVIISDI
6 gallus_lpin1_XP_015131575.1      82.0%
RAAKQNLGSLQANSSHLSLLSGVSYKKTLRLTSDQLKSLKLKNGPNDVTFSVTTQYQGTCRCEGTIYLWNWDDKVIISDI

                                                  801           .          .          .          .          :
.          .          . 880
1 ruby_lpin1_i9_HQ_rubyallcluste 100.0%
DGTITRSDTLGHILPTLGKDWTHQGIAKLYHKVSQNGYKFLYCSARAIGMADMTRGYLHWVNERGTVLPQGPVLLSPSSL
2 canna_lpin1_XP_008493249.1       95.9%
DGTITRSDTLGHILPTLGKDWTHQGIAKLYHKVSQNGYKFLYCSARAIGMADMTRGYLHWVNERGTVLPQGPVLLSPSSL
3 cpelagica_lpin1_XP_010000011.1   88.6%
DGTITRSDTLGHILPTLGKDWTHQGIAKLYHKVSQNGYKFLYCSARAIGMADMTRGYLHWVNERGTVLPQGPVLLSPSSL
4 hsapiens_lpin1_NP_663731.1       74.8%
DGTITRSDTLGHILPTLGKDWTHQGIAKLYHKVSQNGYKFLYCSARAIGMADMTRGYLHWVNERGTVLPQGPLLLSPSSL
5 amiss_lpin1_XP_006258935.1       80.4%
DGTITRSDTLGHILPTLGKDWTHQGIAKLYHKVSQNGYKFLYCSARAIGMADMTRGYLHWVNERGTVLPQGPVLLSPSSL
6 gallus_lpin1_XP_015131575.1      82.0%
DGTITRSDTLGHILPTLGKDWTHQGIAKLYHKVSQNGYKFLYCSARAIGMADMTRGYLHWVNERGTVLPQGPVLLSPSSL

                                                  881           .          9          .          .          .
.          :          . 960
1 ruby_lpin1_i9_HQ_rubyallcluste 100.0%
FSALHREVIEKKPEKFKVQCLTDIKNLFYPNTEPFYAAFGNRPADVYSYKQVGVSLNRIFTVNPKGELIQEHAKTNISSY
2 canna_lpin1_XP_008493249.1       95.9%
FSALHREVIEKKPEKFKVQCLTDIKNLFYPNTEPFYAAFGNRPADVYSYKQVGVSLNRIFTVNPKGELIQEHAKTNISSY
3 cpelagica_lpin1_XP_010000011.1   88.6%
FSAFHREVIEKKPEKFKVQCLTDIKNLFYPNTEPFYAAFGNRPADVYSYKQVGVSLNRIFTVNPKGELIQEHAKTNISSY
4 hsapiens_lpin1_NP_663731.1       74.8%
FSALHREVIEKKPEKFKVQCLTDIKNLFFPNTEPFYAAFGNRPADVYSYKQVGVSLNRIFTVNPKGELVQEHAKTNISSY
5 amiss_lpin1_XP_006258935.1       80.4%
FSAFHREVIEKKPEKFKVQCLTDIKNLFYPNIEPFYAAFGNRPADVYSYKQVGVSLNRIFTVNPKGELVQEHAKTNISSY
6 gallus_lpin1_XP_015131575.1      82.0%
FSALHREVIEKKPEKFKVQCLTDIKNLFYPNTEPFYAAFGNRPADVYSYKQVGVSLNRIFTVNPKGELIQEHAKTNISSY

                                                  961           .          .          .          0          .  ]
1013
1 ruby_lpin1_i9_HQ_rubyallcluste 100.0%      VRLCEVVDHIFPLLKRSHSSDFPCSDTYSQFTYWREPLPPFETQDETPASS--
2 canna_lpin1_XP_008493249.1       95.9%      VRLCEVVDHIFPLLKRSHSSDFPCSDTYSQFTYWREPLPPFETQDETPASS--
3 cpelagica_lpin1_XP_010000011.1   88.6%      VRLCEVVDHIFPLLKRSHSSDFPCSDTYSQFTYWREPLPPFETQDENPPSS--
4 hsapiens_lpin1_NP_663731.1       74.8%        VRLCEVVDHVFPLLKRSHSSDFPCSDTFSNFTFWREPLPPFENQDIHSASA--
5 amiss_lpin1_XP_006258935.1       80.4%        VRLCEVVDHVFPLLKRSHSSDFPCSDTYSQFTYWREPLPPFENQDINRSLS--
6 gallus_lpin1_XP_015131575.1      82.0%        VRLCEVVDHIFPLLKRSHSSDFPCSDTYSQFTYWREPLPPFETQDVHPDS---
```

MView 1.60.1, Copyright © 1997-2015 Nigel P. Brown

```
Reference sequence (1): ruby_mcat_i2_HQ_rubyallcluster
Identities normalised by aligned length.
Colored by: identity + property

                                                   1 [          .          .          .          .          :
.          .          . 80

1 ruby_mcat_i2_HQ_rubyallcluster 100.0%     -----------------------TRRRGSSRPGDGDRAAILSDLLQSSV-
-EAEEPGAAAAARRARPSPQEGTVLLFPGQG
2 canna_mcat_XP_008488495.1        74.0%     ------------------------------------------------------GAD-
--APRAVPPPRSPSLSPFPRKG
3 cpelagica_mcat_XP_010001330.1    68.6%     ------------------------------------------------------
GKEQRWKPQERKGFRATGISEACQRS
4 gallus_mcat_XP_015146784.1       80.4%
GSWAAATWRLAGCSGRGVLRGAARRRGSSLRGGGERAASLSELLQSSVGDEEPGEGEAEGERRERRSPREGAVLLFPGQG
5 amiss_mcat_KYO45338.1            74.5%     -----------------------------------------------------------
-----------------------
6 amiss_hsapiens_AAH42195.2        61.0%     -----MSVRVARVAWVRGLGASYRRGASSFPVPPPGAQGVAELLRDATG----
AEEEAPWAATERRMPGQCSVLLFPGQG

                                                  81           .          1          .          .          .
.          :          . 160
1 ruby_mcat_i2_HQ_rubyallcluster 100.0%
SQFVGMGRGLLGYPGVRDMYRLAEKVLGYDLLSLCLEGPREELDRTQHCQPAVFVASLAAVEKLNHQQPKVVESCVAAAG
2 canna_mcat_XP_008488495.1        74.0%     P----GGQGAQGRHTMPSPG-----------SSIC----------------------
PRPALPGLGKAMAVPTQGLPGAAG
3 cpelagica_mcat_XP_010001330.1    68.6%     A----LLTWLLSWHTFKSFY-----------CHSCGCWNEEAGS-----------
```

```
SRKVEIPLVTDVLKRVVRSCVAAAG
4 gallus_mcat_XP_015146784.1      80.4%
SQFVGMGRGLLRYPGVRDMYRLAEKVLGYDLLSLCLEGPRAELDRTRHCQPAVFVASLAAVEKLNHLQPKVVESCVAAAG
5 amiss_mcat_KYO45338.1           74.5%     -----
-GRGLLSFPGARDLFVAARDVLGYDLLSLCLHGPQTELDRTVHCQPAVFVSSLAAVEKLSHQRPDVIENCVAAAG
6 amiss_hsapiens_AAH42195.2       61.0%
SQVVGMGRGLLNYPRVRELYAAARRVLGYDLLELSLHGPQETLDRTVHCQPAIFVASLAAVEKLHHLQPSVIENCVAAAG

                                        161          .         .         .         2         .
.         .         . 240
1 ruby_mcat_i2_HQ_rubyallcluster 100.0%
YSVGEFAALVFAGALGFAEALYAVKVRAEAMQKASEAAPSGMLSVIGRREANYKFACLEARKHCESLGIENPVCEISNYL
2 canna_mcat_XP_008488495.1       74.0%
YSVGEFAALVFAGALGFAEALYAVKVRAEAMQKASEAAPSGMLSVIGRREANYKFACLEARKHCESLGIENPVCEISNYL
3 cpelagica_mcat_XP_010001330.1   68.6%
YSVGEFAALVFAGALSFAEALYAVKVRAEAMQKASEAVPSGMLSVVGRREANYKFACLEARQHCESLGVANPVCAVSNYL
4 gallus_mcat_XP_015146784.1      80.4%
YSVGEFAALVFAGAMDFAEALYAVKVRAEAMQAAAEAVPSGMLSVIGGREANYKYACLEARRHCESLGIDNPVCEISNYL
5 amiss_mcat_KYO45338.1           74.5%
FSVGEFAALVFAGALDFEAALYAVKVRAEAMQQASEAVPSGMLSIIGQPKSDFKVACIEAREHCRSLGVEDPVCEVANYL
6 amiss_hsapiens_AAH42195.2       61.0%
FSVGEFAALVFAGAMEFAEGLYAVKIRAEAMQEASEAVPSGMLSVLGQPQSKFNFACLEAREHCKSLGIENPVCEVSNYL

                                        241          :         .         .         .         .
3         .         . 320
1 ruby_mcat_i2_HQ_rubyallcluster 100.0%
FPDSRVIAGHIQALEFLQENARKYYFTRAKLLPVSGAFHTRLMEPAVEPLAEALKSIEIQKPLLCVYSNVDGKKYMHSKH
2 canna_mcat_XP_008488495.1       74.0%
FPDSRVIAGHIQALEFLQENARKYYFTRAKLLPVSGAFHTRLMEPAVEPLAEALKSIEIQKPLLCVYSNVDGKKYMHSKH
3 cpelagica_mcat_XP_010001330.1   68.6%
FPDCRVIAGHLQALEFLQENARKYYFTRTKMLPVSGAFHTRLMEPAVEPLAEVLKSIEIQKPLVCVYSNVDGKKYMHSKH
4 gallus_mcat_XP_015146784.1      80.4%
FPDSRVIAGHIQALEFLQKNAPKFSFTRTKMLPVSGAFHTRLMEPAVEPLAEVLKSIEIQKPLICVYSNVDSKKYMHSKH
5 amiss_mcat_KYO45338.1           74.5%
FPDSRVVAGHLQALKFLEENSRKYYFARTKMLPVSGAFHTRLMESAIEPLAKVLKSVEIRKPLINVYSNVDGKKYMHSKH
6 amiss_hsapiens_AAH42195.2       61.0%
FPDCRVISGHQEALRFLQKNSSKFHFRRTRMLPVSGAFHTRLMEPAVEPLTQALKAVDIKKPLVSVYSNVHGHRYRHPGH

                                        321          .         .         :         .         .
.         .       ] 399
1 ruby_mcat_i2_HQ_rubyallcluster 100.0%
IQKLLVKQVVSPVMWEQTMHSVYERKQGTEFPYTYEVGPGKQLGAILKKCNLKAWKQYNHVDALE-----DEEAAET--
2 canna_mcat_XP_008488495.1       74.0%
IQKLLVKQVVSPVMWEQTMHSVYERKQGTEFPYTYEVGPGKQLGAILKKCNLKAWKQYNHVDALE-----DEEAAET--
3 cpelagica_mcat_XP_010001330.1   68.6%
IQKLLVKQVVSPVMWEQTMHSVYERKQGTEFPYTYEVGPGKQLGAILKKCNLKAWKQYKHVDALE-----DEEAAET--
4 gallus_mcat_XP_015146784.1      80.4%
IQKLLVKQLVSPVLWEQTMHSMYQRKQGMEFPYTYEVGPGKQLGAVLRKCNLKAWRQYSHVDVTE-----EEEAAET--
5 amiss_mcat_KYO45338.1           74.5%
IQHLLVKQLVSPVKWEQTMHEVYERTKRTKFPFTYEVGPGKQLGAMLRNCNLKAWRVYKHIEVSE-----DEEVEEA--
6 amiss_hsapiens_AAH42195.2       61.0%
IHKLLAQQLVSPVKWEQTMHAIYERKKGRGFPQTFEVGPGRQLGAILKSCNMQAWKSYSAVDVLQTLEHVDLDPQEPP-
```

MView 1.60.1, Copyright © 1997-2015 Nigel P. Brown

 Supplemental data 2. Alignments of coding sequence for hepatic lipogenic enzymes for compared species [canna= *Calypte anna*, swift = *Chaetura pelagica*, gallus= *Gallus gallus*, alligator= *Alligator mississippiensis*, human= *Homo sapiens*].

```
Reference sequence (1): canna_DGAT2
Identities normalised by aligned length.
Colored by: identity + property


                              1 [         .         .         .         .         :         .
.         . 80
1 canna_DGAT2     100.0%      --------------------------------------------------------------------
------------
2 swift_DGAT2      91.2%      --------------------------------------------------------------------
------------
3 gallus_DGAT2     89.8%      --------------------------------------------------------------------
------------
4 alligator_DGAT2  82.5%      --------------------------------------------------------------------
------------
5 human_DGAT2      70.5%
ATGAAGACCCTCATAGCCGCCTACTCCGGGGTCCTGCGCGGCGAGCGTCAGGCCGAGGCTGACCGGAGCCAGCGCTCTCA

                             81         .         1         .         .         .         .
:         . 160
1 canna_DGAT2     100.0%
ATGAAAACCATCATCGCTGCTTATTCGGGG-GTGCTGCGAGGCACAGGGTCGAACATTCTTTCTTCTCTGCAGGATTTGT
2 swift_DGAT2      91.2%      --------------------------------------------------------------------
------------
3 gallus_DGAT2     89.8%
ATGAAAACCATCATCGCCGCGTACTCGGGG-GTGCTGCGAGGTACGGGGTCGAGCATTCTCTCTGCTCTGCAGGACTTGT
4 alligator_DGAT2  82.5%
ATGAAGACGCTCATTGCTGCCTACTCGGGC-GTCTTGCGAGGCACTGGGTCAAACATCCTCTCTGCCCTTCAGGATGTAT
5 human_DGAT2      70.5%
CGGAGGACCTGCGCTGTCGCGCGAGGGGTCTGGGAGATGGGGCACTGGATCCAGCATCCTCTCCGCCCTCCAGGACCTCT

                            161         .         .         .         2         .         .
.         . 240
1 canna_DGAT2     100.0%      TCT--------
-GGTTGTCTAAATCCAAGCTAGAGAAACAACTCCAAATCATCTCTGTGCTGCAATGGGTTCTCACTTTC
2 swift_DGAT2      91.2%      --------------------------------------------------------------------
------------
3 gallus_DGAT2     89.8%      TCT--------
-GGCTGTCTAAATCCAAAGTAGAGAAACAACTGCAGATCATCTCCGTGCTGCAATGGGTCCTCACGTTC
4 alligator_DGAT2  82.5%      TCT--------
-GGTTCTCAAAGTCCAAGTTTGAGAAACAGCTCCAGATTATATCCGTGCTGCAATGGGTTCTGTCTTTC
5 human_DGAT2      70.5%
TCTCTGTCACCTGGCTCAATAGGTCCAAGGTGGAAAAGCAGCTACAGGTCATCTCAGTGCTCCAGTGGGTCCTGTCCTTC

                            241         :         .         .         .         .         3
.         . 320
1 canna_DGAT2     100.0%
CTAGTCATGGGTGTTGCTTGCACTTTAATCCTCATGTACATACTGTGCACAGATTGTTGGGCCATTGCTGCTCTTTATTT
2 swift_DGAT2      91.2%      -----
-ATGGGTGTTGCTTGCACTTTAATCCTCATGTACATACTGTGCACAGACTGCTGGGCCATTGCTGCCCTATATTT
3 gallus_DGAT2     89.8%
CTCATCATGGGTATTGCTTGCACTTTAATCCTCATGTACATCCTGTGCACAGATTGCTGGGCGATCGCTGCTCTGTATTT
4 alligator_DGAT2  82.5%
CTTGTCCTGGGTGTTACTTGCTCTATCATACTCGTGTACATCCTCTGCACAGACTGCTGGGCAATTGCAGCACTGTATGT
5 human_DGAT2      70.5%
CTTGTACTGGGAGTGGCCTGCAGTGCCATCCTCATGTACATATTCTGCACTGATTGCTGGCTCATCGCTGTGCTCTACTT

                            321         .         .         :         .         .         .
.         4 400
1 canna_DGAT2     100.0%
AGCCTGGCTGGTATTTGACTGGAATACACCAAAGAAAGGTGGAAGAAGATCCCAATGGGTGAGGAACTGGGCTATATGGA
2 swift_DGAT2      91.2%
AGCCTGGCTGGTTTTTGACTGGAATACACCAAAGAAAGGTGGAAGAAGATCCCAATGGGTGAGAAACTGGGCTATATGGA
3 gallus_DGAT2     89.8%
AGCCTGGCTGGTGTTCGACTGGAATACACCAAAGAAAGGTGGAAGAAGATCCCAGTGGGTGAGAAATTGGGCTATATGGA
4 alligator_DGAT2  82.5%
GGCATGGCTGGTGTTCGATTGGGATACTCCAATGAAGGGTGGAAGAAGATCCCCGTGGGTCAGAAACTGGGCCATGTGGC
5 human_DGAT2      70.5%
CACTTGGCTGGTGTTTGACTGGAACACACCCAAGAAAGGTGGCAGGAGGTCACAGTGGGTCCGAAACTGGGCTGTGTGGC

                            401         .         .         .         .         :         .
.         . 480
1 canna_DGAT2     100.0%
GATACTTCAGGGATTATTTTCCAATAAGACTGGTTAAAACCCACAATCTGCTGACCACAAGGAATTACATTTTTGGGTAC
2 swift_DGAT2      91.2%
GGTACTTCAGGGATTATTTTCCAATTAGACTGGTTAAAACCCACAATCTGCTGACCACCAGGAATTACATTTTTGGGTAC
3 gallus_DGAT2     89.8%
GGTACTTCAGGGATTATTTCCCAATAAGACTGGTGAAAACCCACAACCTGCTGACCACCAGGAATTACATCTTCGGTTAC
```

```
4 alligator_DGAT2  82.5%
GGTACTTCAGAGATTATTTTCCAATACGGCTGGTGAAGACCCACAACTTGCCAACCAATCGGAATTACATATTTGGCTAC
5 human_DGAT2      70.5%
GCTACTTTCGAGACTACTTTCCCATCCAGCTGGTGAAGACACACAACCTGCTGACCACCAGGAACTATATCTTTGGATAC

                           481          .         5         .         .         .         .
:         . 560
1 canna_DGAT2     100.0%
CACCCTCATGGCATCATGGGCTTGGGTGCCTTTTGCAACTTCAGCACAGAGGCCACAGGTGTTGGCCAGAAATTTCCTGG
2 swift_DGAT2      91.2%
CATCCACATGGAATCATGGGCTTGGGTGCCTTTTGCAACTTCAGCACAGAAGCTACAGGTGTCAGCCAGAAATTCCCTGG
3 gallus_DGAT2     89.8%
CATCCGCATGGCATCATGGGCTTGGGAGCCTTTTGCAACTTCAGCACAGAGGCCACGGGAGTCAGCCAGAAATTCCCTGG
4 alligator_DGAT2  82.5%
CACCCACATGGCATCATGGGCTTTGGCGCGTTCTGCAACTTTGGCACAGAAGCCACCGGGGTCAGCCAGAAGTTTCCTGG
5 human_DGAT2      70.5%
CACCCCCATGGTATCATGGGCCTGGGTGCCTTCTGCAACTTCAGCACAGAGGCCACAGAAGTGAGCAAGAAGTTCCCAGG

                           561          .         .         .         6         .         .
.         . 640
1 canna_DGAT2     100.0%
GATCCGACCCTACCTTGCTACCCTGGCTGGGAACTTCAGGATGCCCATTTTGAGGGACTACTTAATGTCTGGTGGTATAT
2 swift_DGAT2      91.2%
GATTCGACCATACCTTGCTACCCTGGCTGGGAACTTCAGGATGCCCATTTTGAGGGACTACTTAATGTCTGGTGGTATAT
3 gallus_DGAT2     89.8%
GATCCGGCCGTACCTGGCCACCCTGGCTGGGAACTTCAGGATGCCCATCCTGAGGGACTACTTAATGTCTGGGGGTATAT
4 alligator_DGAT2  82.5%
CATTAGACCGTATCTTGCTACGTTGGCTGGGAACTTCCGGATACCAATTTTGAGGGACTACTTGATGTCCGGTGGTATTT
5 human_DGAT2      70.5%
CATACGGCCTTACCTGGCTACACTGGCAGGCAACTTCCGAATGCCTGTGTTGAGGGAGTACCTGATGTCTGGAGGTATCT

                           641          :         .         .         .         .         7
.         . 720
1 canna_DGAT2     100.0%
GTCCTGTGAACCGTGACAGCATAGACTACATCTTGTCCAAGAATGGCACTGGCAATGCCATCATCATCGTGGTTGGAGGG
2 swift_DGAT2      91.2%
GTCCTGTGAACCGGGACAGCATAGACTACATCTTGTCCAAGAATGGCAGTGGCAATGCCATCATCATCGTGGTTGGAGGG
3 gallus_DGAT2     89.8%
GTCCCGTGAACCGTGACAGCATAGACTACATCTTGTCCAAGAATGGCAGTGGCAATGCCATCATCATCGTGGTGGGAGGA
4 alligator_DGAT2  82.5%
GCCCTGTGAACCGTCACTCCATAGATTTCATCCTATCCAAGAATGGCACTGGCAACGCCGTGATCATTGTGGTGGGGGGA
5 human_DGAT2      70.5%
GCCCTGTCAGCCGGGACACCATAGACTATTTGCTTTCAAAGAATGGGAGTGGCAATGCTATCATCATCGTGGTCGGGGGT

                           721          .         .         :         .         .         .
.        8 800
1 canna_DGAT2     100.0%
GCAGCAGAGTCCTTGAACTGCACCCCGGGCAAGAACTCGGTGACGCTGAAAAATCGGAAAGGATTTGTGAAGCTGGCTCT
2 swift_DGAT2      91.2%
GCAGCAGAGTCCCTGAACTGCACCCCGGGGAAGAACTCGGTGACGCTGAAGAACAGGAAGGGATTTGTGAAGCTGGCTCT
3 gallus_DGAT2     89.8%
GCAGCGGAGTCCCTCAACTGCACCCCTGGGAAGAACTCGGTGACACTGAGAAACAGGAAGGGATTTGTGAAACTGGCACT
4 alligator_DGAT2  82.5%
GCAGCAGAGTCCCTGAACTGCACGCCTGGGAAGAACTTGGTGATTCTGAAAAATCGGAAAGGATTTGTAAAGCTGGCTCT
5 human_DGAT2      70.5%
GCGGCTGAGTCTCTGAGCTCCATGCCTGGCAAGAATGCAGTCACCCTGCGGAACCGCAAGGGCTTTGTGAAACTGGCCCT

                           801          .         .         .         .         :         .
.         . 880
1 canna_DGAT2     100.0%
GAGACATGGGGCGGACTTGGTTCCTGTCTACTCATTTGGGGAGAACGAAGTGTACAAGCAAGTGATCTTTGAGGAGGGCT
2 swift_DGAT2      91.2%
GAGACATGGGGCGGACTTGGTTCCCGTCTACTCCTTTGGGGAGAACGAGGTGTACAAGCAGGTGATCTTTGAGGAGGGCT
3 gallus_DGAT2     89.8%
GCGGCACGGTGCCGACTTGGTTCCTGTCTACTCTTTTGGGGAGAACGAAGTGTACAAGCAGGTGATCTTTGAGGAGGGCT
4 alligator_DGAT2  82.5%
GCAGCATGGGGCAGATCTAGTTCCCGTGTACTCATTTGGCGAGAACGAGGTGTACAAGCAGGTGATCTTCGAGGAGGGCT
5 human_DGAT2      70.5%
GCGTCATGGAGCTGACCTGGTTCCCATCTACTCCTTTGGAGAGAATGAAGTGTACAAGCAGGTGATCTTCGAGGAGGGCT

                           881          .         9         .         .         .         .
:         . 960
1 canna_DGAT2     100.0%
CCTGGGGAAGATGGGTTCAGAAGAAGTTTCAGAAGCACATTGGATTTGCTCCCTGCATCTTTCATGGCCGTGGCCTCTTC
2 swift_DGAT2      91.2%
CCTGGGGAAGATGGGTTCAGAAGAAGTTTCAGAAGCACATTGGCTTTGCTCCATGCATCTTTCATGGCCGTGGCCTCTTC
3 gallus_DGAT2     89.8%
CCTGGGGAAGATGGGTTCAGAAGAAGTTTCAGAAGCACATTGGCTTTGCTCCATGCATCTTCCATGGCCGTGGGCTCTTC
4 alligator_DGAT2  82.5%
GCTGGGGCAGATGGGTCCAGAAGAAGTTTCAGAAGTACATTGGCTTTGCTCCGTGCATCTTTCATGGTCGTGGCCTCTTC
5 human_DGAT2      70.5%
```

CCTGGGGCCGATGGGTCCAGAAGAAGTTCCAGAAATACATTGGTTTCGCCCCATGCATCTTCCATGGTCGAGGCCTCTTC

```
                          961           .         .         .         0         .         .
.         . 1040
1 canna_DGAT2     100.0%
TCCTCCAACACCTGGGGATTGTTACCTTACTCCAAGCCCATCACTACTGTTGTTGGGGAACCCATCACCATCCCCAAAGT
2 swift_DGAT2      91.2%
TCCTCTAACACCTGGGGGTTGTTACCTTACTCCAAGCCCATCACTACTGTTGTTGGGGAGCCCATCACCATTCCCAAAAT
3 gallus_DGAT2     89.8%
TCCTCGAACACGTGGGGCTTGCTGCCGTACTCCAAGCCCATCACCACTGTTGTGGGCGAACCCATCACCATCCCAAAGAT
4 alligator_DGAT2  82.5%
TCTTCCAACACCTGGGGTTTGATCCCTTACCCTAACCCCATCACCACAGTTGTTGGGGAGCCAATCACCATCCCGAAAAC
5 human_DGAT2      70.5%
TCCTCCGACACCTGGGGGCTGGTGCCCTACTCCAAGCCCATCACCACTGTTGTGGGAGAGCCCATCACCATCCCCAAGCT

                         1041           :         .         .         .         .         1
.         . 1120
1 canna_DGAT2     100.0%
TGATAATCCATCCCAGGAAGAAGTGGATTTCTACCACAGCATCTATGTGGACTCCCTGATCAAACTCTTTGACAAGTACA
2 swift_DGAT2      91.2%
TGAGAATCCATCCCAGCAGGACGTGGACTTCTACCATAGCATGTATGTGGACTCCCTGATCAAACTCTTTGACAAGTACA
3 gallus_DGAT2     89.8%
CGATAACCCATCTCAGAAGGAAGTGGACTTCTACCACAGCGTGTACGTGGACTCCCTGATCAAGCTCTTTGACAAGTACA
4 alligator_DGAT2  82.5%
AGCTCATCCCACCCAGAGGGAAATCGACCTTTATCACAGCATGTACGTGAGCTCGCTGAGTAAGCTCTTTGACAAGTACA
5 human_DGAT2      70.5%
GGAGCACCCAACCCAGCAAGACATCGACCTGTACCACACCATGTACATGGAGGCCCTGGTGAAGCTCTTCGACAAGCACA

                         1121           .         .         :         .         .         .   ]
1184
1 canna_DGAT2     100.0%      AGATCAAATTCGGCCTGCCAGAGACTGAGGTCTTGGAAGTCAACTGA-----------------
2 swift_DGAT2      91.2%      AGAGCAAATTTGGGCTAAGGAAAACTGATACTTTGATTATTGTGTGTGCCCAGGCTGCATAA--
3 gallus_DGAT2     89.8%      AAGGCAGGTTCGGGTTGCCAGAGACTGAGGTCCTGGAAGTCAACTGA-----------------
4 alligator_DGAT2  82.5%      AGGCTAAGTTCGGCCTGCCAGAGACGGAGATCTTGGAAGTCAACTGA-----------------
5 human_DGAT2      70.5%      AGACCAAGTTCGGCCTCCCGGAGACTGAGGTCCTGGAGGTGAACTGA-----------------
```

MView 1.60.1, Copyright © 1997-2015 Nigel P. Brown

```
Reference sequence (1): ruby_ABHD5
Identities normalised by aligned length.
Colored by: identity + property


                            1 [         .         .         .         .         :         .
.         . 80
1 ruby_ABHD5      100.0%
ATGGCCGGTGCCGCCGCTGCGCCTCCCTTCGCTGCCCCACCGGCCACCGCCGACCCACCGCTGACAGCTCCCGGCCGCCG
2 canna_ABHD5      94.2%      ----------------------------------ATGGATTTTGTAATGGTAAT--
GCTGCGGGAAGATGTTAACAAAGA
3 swift_ABHD5      92.2%      --------------------------------------------------------------------
---------ATG
4 human_ABHD5      39.8%      --------------------------------------------------------------------
------------
5 gallus_ABHD5     91.3%      --------------------------------------------------------------------
------------
6 alligator_ABHD5  82.7%
ATGGCCGGGGTCGCGGCGTTCACGCTGTCACTCGCTGCGCTCCCGGCTGCGTCCCAGCCACCCGCTGCCGGCGGCCGCCA

                           81           .         1         .         .         .         .
:         . 160
1 ruby_ABHD5      100.0%      CGCCGCCGCCATGGCCGAGGAGGAGACC-
-TCCAGCGAAGGGTTAGGATGGTTATTCAGCTGGCTTCCTGCCTGGTGTCC
2 canna_ABHD5      94.2%      AGCATTAATTCTTGGCCTAAACAGGACT--T--------
-GGTTAGGATGGTTATTCAGCTGGCTTCCTGCCTGGTGTCC
3 swift_ABHD5      92.2%
GGCACAAGTTGCACCCCAGGGAGATTCCGATTGGACACAAGGTTAGGATGGTTATTCAGCTGGCTTCCTGCCTGGTGTCC
4 human_ABHD5      39.8%      ---------------------ATGGATT-
-TGTGGCCAGGGGCATGGATGCTGCTGCTGCTGCTCTTCCTGCTGCTGCTC
5 gallus_ABHD5     91.3%      ----------ATGGCCGAGGAGGAGGCC-
-TCCAGCGAAAGGTTAGGATGGTTGTTCAGCTGGCTTCCTGCTTGGTGCCC
6 alligator_ABHD5  82.7%      CACGCGCGACATGTCTGACGGCGAGACA--
GCCAGCGAAGGGTCAGGATGGCTGTCCGGTTGGCTTCCTGCATGGTGTCC

                          161           .         .         .         2         .         .
.         . 240
1 ruby_ABHD5      100.0%
CACATCACTGCTACACCTTAAAGAGGCCGAGAACAAAATGCTAAAATGTATTGCAAGCACATACAATAAACGATATGTGT
```

```
 2 canna_ABHD5      94.2%
 CACATCACTGCTACACCTTAAAGAGGCCGAGAACAAAATGCTAAAATGTATTGCAAGCACATACAATAAACGATATGTGT
 3 swift_ABHD5      92.2%
 CACATCACTGCTACACCTTAAAGAAGCTGAGGACAAAATGCTAAAATGTATCACAAGCACATACAATAAACAATATGTGT
 4 human_ABHD5      39.8%      TTC-CTGCTGCC-CACCCTGTGGTT-CTGCAGCCCCAGTGCC-AAGTACTTCTTCAAGATG-
 GCCTTCTACAATGGCTGG
 5 gallus_ABHD5     91.3%
 CACATCACTACTGCACCTTAAAGAAGCTGAGGACAAAATGCTAAAATGTATTACAAGCACATACAATAAGCGGTATGTGT
 6 alligator_ABHD5  82.7%
 CACATCAATGCCACATCTTAAAGACGCAGAAGAGAAAATACTGAAGTGTATCACCAGCACATACAGTAAACAATATGTCT

                           241          :         .         .         .         .         3
 .         . 320
 1 ruby_ABHD5      100.0%
 ATATATCTAATGGAAATAAAATATGGACACTGACATTCTCTCCAGATGTTTCACATAAAACTCCACTTGTTCTCCTGCAT
 2 canna_ABHD5      94.2%
 ATATATCTAATGGAAATAAAATATGGACACTGACATTCTCTCCAGATGTTTCACATAAAACTCCACTTGTTCTCCTGCAT
 3 swift_ABHD5      92.2%
 ATATATCTCATGGAAATAAAATATGGACACTGACATTCTCTCCGGACCTTTCACATAAAACTCCACTTGTTCTCCTGCAT
 4 human_ABHD5      39.8%      ATCCTCTTCCTGGCTGT-----------
 GCTCGCCATCCCTGTGTGTGCCGTGCG-AGGACGCAACGTCGAGAACATGAA
 5 gallus_ABHD5     91.3%
 ATCTAGCTAATGGAAACAAGATATGGACACTGACGTTCTCTCCAGACCTTTCACGTAAAACTCCACTTGTTCTGCTTCAT
 6 alligator_ABHD5  82.7%
 ACATATCCAATGGGAATAAAATATGGACGCTCACATTCTCTCAGGACCTTTCACTTAAAACTCCACTTATTCTCCTTCAT

                           321          .         .         :         .         .         .
 .         4 400
 1 ruby_ABHD5      100.0%
 GGGTTTGGAGGAGGTGTTGGACTCTGGGCTCTCAATTTTGAAGATCTCTGTGAGAACAGGACCGTTCATGCTTTTGACCT
 2 canna_ABHD5      94.2%
 GGGTTTGGAGGAGGTGTTGGACTCTGGGCTCTCAATTTTGAAGATCTCTGTGAGAACAGGACCGTTCATGCTTTTGACCT
 3 swift_ABHD5      92.2%
 GGGTTTGGTGGAGGTGTTGGACTGTGGGCTCTCAATTTTGAAGATCTCTGTGAGAACAGGACCGTTCATGCTTTTGACCT
 4 human_ABHD5      39.8%
 GATCTTGCGTCTAATGCTGCTCCACATCAAATACCTGTACGGGATCCGAGTGGAGGTGCGAGGGGCTCACCACTTCCCTC
 5 gallus_ABHD5     91.3%
 GGATTTGGAGGAGGTGTTGGAATGTGGGCTCTCAATTTTGAAGAGCTCTGTGAAAACAGGACCGTTCATGCTTTCGACCT
 6 alligator_ABHD5  82.7%
 GGATTTGGAGGAGGTGTTGGACTTTGGGCTCTTAATTTTGAAGACCTTTGTGAAAATAGAACCGTTTATGCAATTGACCT

                           401          .         .         .         .         :         .
 .         . 480
 1 ruby_ABHD5      100.0%
 CTTGGGATTTGGACATAGCAGTAGACCACAGTTTCACACTGATGCTCGGGAAGCAGAGAACCAGTTTGTGGAATCCATAG
 2 canna_ABHD5      94.2%
 CTTGGGATTTGGACATAGCAGTAGACCACAGTTTCACACTGATGCTCGGGAAGCAGAGAACCAGTTTGTGGAATCCATAG
 3 swift_ABHD5      92.2%
 CTTGGGATTTGGACGTAGCAGTAGGCCACACTTTCACACTGATGCTCGGGAAGCAGAAAATCAGTTTGTGGAGTCCATAG
 4 human_ABHD5      39.8%      CCTCGCAGCCCTATGTTGTTGTCTCCAACCACCAGA-GCT--CTCTCGATCTGCTTGGGATGA----
 -TGGAGGTACTGC
 5 gallus_ABHD5     91.3%
 CTTGGGATTTGGACGAAGCAGTAGACCACACTTTGATACTGATGCTCGGGAAGCAGAAAATCAGTTTGTGGAATCCATAG
 6 alligator_ABHD5  82.7%
 CTTGGGATTTGGGCGCAGTAGTAGACCACATTTTGACACTGATGCAGAAGAAGCAGAAAATCAATTTGTAGAATCTATAG

                           481          .         5         .         .         .         .
 :         . 560
 1 ruby_ABHD5      100.0%
 AAGAATGGAGAAAGGCGATGGAGATAGAAAAAATGATTTTACTTGGACACAACCTAGGTGGATTCCTGGCTGCTGCTTAC
 2 canna_ABHD5      94.2%
 AAGAATGGAGAAAGGCGATGGAGATAGAAAAAATGATTTTACTTGGACACAACCTAGGTGGATTCCTGGCTGCTGCTTAC
 3 swift_ABHD5      92.2%
 AAGAATGGAGAAAGGTGATGGAGATAGAAAAAATGATTATACTTGGACACAACCTAGGTGGATTCCTGGCAGCTGCTTAT
 4 human_ABHD5      39.8%
 CAGGCCGCTGTGTGCCCATTGCCA-AGCGCGAGCTACTGTGGGCTGGCTCTGCCGGGCTGGCCTGCTGGCTGGCAGGAGT
 5 gallus_ABHD5     91.3%
 AAGAGTGGAGAAAGGAGATGGGGCTAGAAAAAATGATTTTGCTTGGACACAATCTGGGTGGATTCCTGGCTGCTGCTTAC
 6 alligator_ABHD5  82.7%
 AAGAATGGAGACAGGCAGTAGGGTTAGACAAAATGATTTTCCTTGGACACAACCTAGGGGGATTCCTGGCTGCTGCGTAC

                           561          .         .         .         6         .         .
 .         . 640
 1 ruby_ABHD5      100.0%
 TCATTAAAATATCCATCAAGG-GTCAAACATCTTATCTTAGTGGAGCCGTGGGGTTTTCCAGAGAGGCCTGACAATGCTG
 2 canna_ABHD5      94.2%
 TCATTAAAATACCCATCAAGG-GTCAAACATCTTATCTTAGTGGAGCCGTGGGGTTTTCCAGAGAGGCCTGACAATGCTG
 3 swift_ABHD5      92.2%
 TCATTAAAATACCCATCAAGG-GTCAAACATCTTATCTTAGTGGAGCCATGGGGTTTTCCAGAGAGGCCTGACAATGCTG
 4 human_ABHD5      39.8%      CATCTTCATCGACCGGAAGCGCACGGGGGATGCCATCAGTGTCATGTC-TGAGGTCGCCCAGAC--
 -CCTGCTCACCCAG
```

```
 5 gallus_ABHD5     91.3%
TCATTAAAATACCCATCAAGG-GTCAAACATCTTATCTTAGTGGAGCCATGGGGTTTTCCAGAAAGGCCTGACAATGCTG
 6 alligator_ABHD5  82.7%
TCGCTGAAGTACCCATCAAGG-GTAAAACACCTTATCCTAGTGGAGCCGTGGGGTTTTCCAGAGAGGCCGGACAA---TG

                          641          :          .          .          .          .          7
.         . 720
 1 ruby_ABHD5      100.0%
AACACGAGAGACCGATTCCAATCTGGATCAAGGCACTAGGAGCTATACTGAGTCCATTTAACCCATTAGCTGGTCTGAGG
 2 canna_ABHD5      94.2%
AACACGAGAGACCGATTCCAATCTGGATCAAGGCACTAGGAGCTATACTGAGTCCATTTAACCCATTAGCTGGTCTGAGG
 3 swift_ABHD5      92.2%
AGCACGAAAGACCAATTCCAATCTGGATCAAAGCACTAGGAGCTATACTGAGTCCATTTAATCCATTAGCTGGGCTGAGG
 4 human_ABHD5      39.8%      GACGTGAGGGTCTGGGTGTTTCCTG---
-AGGGAACGAGAAACCACAATGGCTCCATGCTGCCCTTCA------------
 5 gallus_ABHD5     91.3%
AACACGAAAGACCAATTCCAATCTGGATCAAAGCACTAGGAGCTATACTGAGTCCATTTAATCCATTGGCTGGGCTGAGG
 6 alligator_ABHD5  82.7%
AACAAGAGAGACCAATTCCAATCTGGATCAAAGCACTAGGAGCTATGCTGAGTCCATTCAATCCATTAGCTGGGCTCCGG

                          721          .          .          :          .          .          .
.         8 800
 1 ruby_ABHD5      100.0%
ATAGCAGGACCCTTTGGATTAAGCCTTGTTCAACGTTTAAGACCAGATTTCAAGCGAAAATATTCATCAATGTTTGATGA
 2 canna_ABHD5      94.2%
ATAGCAGGACCCTTTGGATTAAGCCTTGTTCAACGTTTAAGACCAGATTTCAAGCGAAAATATTCATCAATGTTTGATGA
 3 swift_ABHD5      92.2%
ATAGCAGGACCCTTTGGATTAAGCCTGGTTCAGCGTTTAAGACCAGATTTCAAGCGAAAATATTCATCGATGTTTGATGA
 4 human_ABHD5      39.8%      --AACGTGGCGCCTTCCAT-----CTTG--CAGTGC--AGGCCCAGGTTCCCATTGTCCCCATA-
GTCATGTCCTCCTAC
 5 gallus_ABHD5     91.3%
ATAGCAGGACCCTTTGGTCTAAGCCTTGTTCAGCGTTTAAGACCAGATTTCAAACGAAAATATGCATCGATGTTTGATGA
 6 alligator_ABHD5  82.7%
TTAGCAGGACCCTTTGGTTTAAGCCTTGTACAGCGTTTAAGACCAGACTTCAAGCGAAAGTATTCTTCTATGTTTGATGA

                          801          .          .          .          .          :          .
.         . 880
 1 ruby_ABHD5      100.0%
TAACACTGTGGCTGAGTACATCTATCACTGCAATGTACAGTCCCCTAGTGGTGAAACAGCTTTTAAGAAC-ATGACAATT
 2 canna_ABHD5      94.2%
TAACACTGTGGCTGAGTACATCTATCACTGCAATGTACAGTCCCCTAGTGGTGAAACAGCTTTTAAGAAC-ATGACAATT
 3 swift_ABHD5      92.2%
TAACACTGTGGCTGAATATATCTATCACTGCAATGTACAGTCACCTAGTGGTGAAACAGCTTTCAAGAAC-ATGACTATT
 4 human_ABHD5      39.8%      CAAGACTTCTACTGCAAGAA--------
-GGAGCGTCGCTTCACCTCGGGACAATGTCAGGTGCGGGTGCTGCCCCCAGT
 5 gallus_ABHD5     91.3%
TAACACTGTGACTGAATATATCTATCACTGCAATGTGCAGTCGCCCAGCGGTGAAACAGCTTTCAAGAAC-ATGACTATT
 6 alligator_ABHD5  82.7%
TAACACTGTGGCTGAATATATCTATCACTGCAATGTACAGTCACCCAGCGGTGAAACTGCTTTCAAGAAC-ATGACTATA

                          881          .          9          .          .          .          .
:         . 960
 1 ruby_ABHD5      100.0%
CCTTACGGATGGGCAAAAAGACCGATGCTGCAAAGGATTTCACAGCTGGATCAAGACATTCCTATCACTGTGGTCTATGG
 2 canna_ABHD5      94.2%
CCTTACGGATGGGCAAAAAGACCGATGCTGCAAAGGATTTCACAGCTGGATCAAGACATTCCTATCACTGTGGTCTATGG
 3 swift_ABHD5      92.2%
CCTTATGGATGGGCAAAAAGGCCAATGCTGCAACGGATTTCACAGATGGATCGAGACATTCCTATCACTGTGATCTATGG
 4 human_ABHD5      39.8%
GCCCACGGAAGGGCTGACAC-CAGATGACGTCCCAGCTCTGGCTGACAGAGTCCGGCACTCCATGCTCACTGTTTTCCGG
 5 gallus_ABHD5     91.3%
CCTTATGGATGGGCAAAAAGGCCAATGCTGCAACGGATTCCACAAATGGATCAAGACATTCCTATCACCGTGGTTTATGG
 6 alligator_ABHD5  82.7%
CCTTATGGATGGGCAAAAAGGCCAATGCTACAGCGGATTTCTCTCATGGATCAAGATATTCCTATCACTGTGATTTATGG

                          961          .          .          .          0          .          .
.         . 1040
 1 ruby_ABHD5      100.0%
AGCACGTTCGTGTATAGATGGGAATTCTGGCAGTACTATCCAGTCCCTGAGACCAAATTCA-TATGTGAAGACAATAGCT
 2 canna_ABHD5      94.2%
AGCACGTTCGTGTATAGATGGGAATTCTGGCAGTACTATCCAGTCCCTGAGACCAAATTCA-TATGTGAAGACAATAGCT
 3 swift_ABHD5      92.2%
AGCACGTTCATGTATAGATGGCAATTCTGGCAGCACTATCCAGTCTCTGAGACCAAATTCC-TATGTGAAGACAATAGCT
 4 human_ABHD5      39.8%      GAAATCTCCACTGATGGCCGG-GGTGGTGGTG--ACTATCTGAAG---
AAGCCTGGGGGCGGTGGGTGA-----------
 5 gallus_ABHD5     91.3%
AGCACGTTCATGCATAGACGGCAATTCTGGCAGCACTATCCAGTCTCTGAGACCAAAGTCA-TATGTGAAGACAATAGCT
 6 alligator_ABHD5  82.7%
AGCACGATCTTGTATAGATGGCAATTCTGGTAGCACCATCCAGTCTCTACGACCAAAGTCA-TATGTGAAGACAATAGCT

                         1041          :          .          .          .          .          1
```

```
.          . 1120
1 ruby_ABHD5      100.0%
ATCCTTGGTGCAGGTCATTACGTGTATGCTGATCAACCTGAAGACTTCAATGAGAGAGTGAAAGATATCTGTGACTCCGT
2 canna_ABHD5      94.2%
ATCCTTGGTGCAGGTCATTACGTGTATGCTGATCAACCTGAAGACTTCAATGAGAGAGTGAAAGATATCTGTGACTCCGT
3 swift_ABHD5      92.2%
ATCCTTGGTGCAGGTCATTATGTGTATGCTGATCAACCTGAAGATTTCAATCAGAAAGTGAAAGATATCTGTGACTCTGT
4 human_ABHD5      39.8%      --------------------------------------------------------------------
------------
5 gallus_ABHD5     91.3%
ATCCTTGGTGCAGGTCATTACGTGTACGCTGATCAGCCTGAAGACTTCAATCAGAAAGTGAAAGACATCTGTGATTCTGT
6 alligator_ABHD5  82.7%
ATCCTTGGAGCTGGTCATTATGTATATGCTGATCAACCTGAAGACTTCAATCAGAAAGTAAAAGAGATCTGTGATTCAGT

                          1121         ] 1129
1 ruby_ABHD5      100.0%      GGACTGA--
2 canna_ABHD5      94.2%      GGACTGA--
3 swift_ABHD5      92.2%      GGACTGA--
4 human_ABHD5      39.8%      ---------
5 gallus_ABHD5     91.3%      GGACTGA--
6 alligator_ABHD5  82.7%      GGACTGA--
```

MView 1.60.1, Copyright © 1997-2015 Nigel P. Brown

```
Reference sequence (1): ruby_ACACA
Identities normalised by aligned length.
Colored by: identity + property


                             1 [        .         .         .         .         :         .
.          . 80
1 ruby_ACACA      100.0%      --------------------------------------------------------------------
------------
2 canna_ACACA      98.5%      --------------------------------------------------------------------
------------
3 swift_ACACA      92.4%      --------------------------------------------------------------------
------------
4 human_ACACA      77.6%
ATGTGGTGGTCTACTCTGATGTCAATCTTGAGGGCTAGGTCTTTTTGGAAGTGGATATCTACTCAGACAGTAAGAATTAT
5 gallus_ACACA     89.0%      --------------------------------------------------------------------
------------
6 alligator_ACACA  84.0%      --------------------------------------------------------------------
------------

                            81          .         1         .         .         .         .
:          . 160
1 ruby_ACACA      100.0%      --------------------------------------------------------------------
------------
2 canna_ACACA      98.5%      --------------------------------------------------------------------
------------
3 swift_ACACA      92.4%      --------------------------------------------------------------------
------------
4 human_ACACA      77.6%
AAGAGCTGTAAGAGCTCATTTTGGAGGAATAATGGATGAACCATCTCCCTTGGCCCAACCTCTGGAGCTGAACCAGCACT
5 gallus_ACACA     89.0%      -------------------------------
ATGGAAGAGTCTTCCCAACCTGCTAAACCCCTGGAGATGAACCCTCACT
6 alligator_ACACA  84.0%      -------------------------------
ATGGAGGACTCCTCCCTGCCTGCAAAGCACCTGGAGCTGAGCGCTCATT

                           161          .         .         .         2         .         .
.          . 240
1 ruby_ACACA      100.0%      --------------------------------------------------------------------
------------
2 canna_ACACA      98.5%      --------------------------------------------------------------------
------------
3 swift_ACACA      92.4%      --------------------------------------------------------------------
------------
4 human_ACACA      77.6%
CTCGATTCATAATAGGTTCTGTGTCTGAAGATAACTCAGAGGATGAGATCAGCAACCTGGTGAAGTTGGACCTACTGGAG
5 gallus_ACACA     89.0%
CTCGCTTTATTATTGGTTCCGTGTCAGAGGATAACTCAGAAGATGAGACGAGCTCCTTGGTGAAACTTGACCTGCTGGAG
6 alligator_ACACA  84.0%
CCCGCTTCATCATTGGCTCTGTGTCTGAGGATAACTCAGAAGATGAGACTAGTTCCTTGGTGAAGCTTGATCTGCTGGAG

                           241          :         .         .         .         .         3
.          . 320
1 ruby ACACA      100.0%      --------------------------------------------
```

```
-ATGGAGGATTCTGCAGAGCAGAGT-CAGGAAATGA
2 canna_ACACA      98.5%      --------------------------------------------
-ATGGAGGATTCTGCAGAGCAGAGT-CAGGAAATGA
3 swift_ACACA      92.4%      --------------------------------------------
-ATGGAGGATTCTGCAGAGCAGAGT-CAGGAAATGA
4 human_ACACA      77.6%
GAGAAGGAGGGCTCCTTGTCACCTGCTTCTGTTGGCTCAGATACACTCTCTGATTTGGGGATCTCTAGCCTACAGGATGG
5 gallus_ACACA     89.0%
GAGAAAGAGAGGTCTCTGTCCCCTGTTTCTGTCTGCTCGGATTCCCTTTCGGATTTGGGACTTCCTAGTGCTCAAGATGG
6 alligator_ACACA  84.0%
GAGAAAGAGAGTTCTCTGTCTCCCGTGTCTGTCTGTTCAGAGTCCTTTTCAGACCTGGGACTACCTAACATTCAAGAAGG

                          321          .         .         :         .         .         .
.         4 400
1 ruby_ACACA      100.0%
GATACCACATGCTGCAAAGGCCCAGCATGTCTGGTTTACACCTTGTAAAGCAAGGCCGGGACAGGAAGAAAGTTGATGTG
2 canna_ACACA      98.5%
GATACCACATGCTGCAAAGGCCCAGCATGTCTGGTTTACACCTTGTAAAGCAAGGCCGGGACAGGAAGAAAGTTGATGTG
3 swift_ACACA      92.4%
GATACCACATGCTGCAAAGGCCCAGTATGTCTGGTTTACACCTTGTAAAGCAAGGCCGGGACAGGAAGAAAGTTGACGTG
4 human_ACACA      77.6%
CTTGGCCTTGCACATAA-GGTCCAGCATGTCTGGCTTGCACCTAGTAAAGCAGGGCCGAGACAGAAAGAAAATAGATTCT
5 gallus_ACACA     89.0%
TTTGGCAAACCATATGA-GGCCCAGCATGTCTGGTTTGCACCTCGTAAAGCAAGGCCGGGACAGGAAGAAAGTTGACGTG
6 alligator_ACACA  84.0%
CCTAGCAAGTCACATGA-GGCCCAGTATGTCTGGCTTGCACCTTGTAAAGCAAGGCCGAGACCGGAAGAAAGTTGACTTG

                          401          .         .         .         .         :         .
.         . 480
1 ruby_ACACA      100.0%
CAGAGGGATTTCACTGTGGCTTCTCCAGCAGAGTTTGTTACTCGTTTTGGAGGGAATAAAGTTATTGAAAAGGTCCTGAT
2 canna_ACACA      98.5%
CAGAGGGATTTCACTGTGGCTTCTCCAGCAGAGTTTGTTACTCGTTTTGGAGGGAATAAAGTTATTGAAAAGGTCCTGAT
3 swift_ACACA      92.4%
CAGAGAGATTTCACTGTGGCTTCTCCAGCAGAATTTGTGACTCGTTTTGGAGGGAATAAAGTCATTGAAAAGGTCCTGAT
4 human_ACACA      77.6%
CAACGAGATTTCACTGTGGCTTCTCCAGCAGAATTTGTTACTCGCTTTGGGGGAAATAAAGTGATTGAGAAGGTTCTTAT
5 gallus_ACACA     89.0%
CAGCGGGATTTCACTGTGGCTTCTCCAGCAGAATTTGTTACTCGTTTTGGAGGGAACAGAGTTATTGAGAAGGTCCTGAT
6 alligator_ACACA  84.0%
CAGAGGGATTTCACTGTGGCTTCCCCAGCCGAATTCGTTACTCGTTTTGGGGGGAATAAAGTCATCGAGAAGGTCCTTAT

                          481          .         5         .         .         .         .
:         . 560
1 ruby_ACACA      100.0%
AGCAAACAATGGCATTGCAGCAGTGAAGTGCATGAGGTCCATCCGGCGCTGGTCCTACGAGATGTTTCGGAACGAGCGGG
2 canna_ACACA      98.5%
AGCAAACAATGGCATTGCAGCAGTGAAGTGCATGAGGTCCATCCGGCGCTGGTCCTACGAGATGTTTCGGAACGAGCGGG
3 swift_ACACA      92.4%
AGCAAACAATGGCATTGCAGCAGTGAAGTGCATGAGGTCCATCCGGCGCTGGTCCTACGAGATGTTTCGAAACGAGCGGG
4 human_ACACA      77.6%
TGCTAACAATGGCATTGCAGCAGTGAAATGCATGCGGTCTATCCGTAGGTGGTCTTATGAAATGTTTCGAAATGAACGTG
5 gallus_ACACA     89.0%
AGCCAACAATGGGATTGCAGCAGTGAAATGCATGAGGTCGATCCGGCGCTGGTCCTATGAGATGTTCCGAAACGAGCGGG
6 alligator_ACACA  84.0%
TGCCAATAATGGGATTGCGGCCGTGAAATGCATGAGGTCCATCCGACGCTGGTCCTATGAGATGTTTCGAAATGAGCGTG

                          561          .         .         .         6         .         .
.         . 640
1 ruby_ACACA      100.0%
CCATCAGATTTGTTGTCATGGTGACTCCTGAGGACCTGAAAGCAAATGCAGAGTATATTAAAATGGCAGATCACTACGTC
2 canna_ACACA      98.5%
CCATCAGATTTGTTGTCATGGTGACTCCTGAGGACCTGAAAGCAAATGCAGAGTATATTAAAATGGCAGATCACTACGTC
3 swift_ACACA      92.4%
CGATCAGATTTGTTGTCATGGTGACTCCTGAGGATCTGAAAGCAAATGCAGAGTATATTAAAATGGCAGATCACTACGTC
4 human_ACACA      77.6%
CAATTAGATTCGTTGTCATGGTCACACCTGAAGACCTTAAAGCCAATGCAGAATACATTAAGATGGCAGATCACTATGTG
5 gallus_ACACA     89.0%
CAATCAGATTTGTTGTCATGGTGACTCCTGAGGACCTGAAAGCAAATGCAGAGTACATTAAAATGGCAGATCACTACGTG
6 alligator_ACACA  84.0%
CCATCAGATTCGTTGTCATGGTGACTCCTGAAGATCTGAAAGCGAATGCAGAGTACATTAAAATGGCTGATCACTATGTC

                          641          :         .         .         .         .         7
.         . 720
1 ruby_ACACA      100.0%
CCAGTTCCAGGAGGCCCCAACAATAACAACTATGCAAATGTGGAACTCATTCTGGATATTGCTAAGCGGATTCCAGTGCA
2 canna_ACACA      98.5%
CCAGTTCCAGGAGGCCCCAACAATAACAACTATGCAAATGTGGAACTCATTCTGGATATTGCTAAGCGGATTCCAGTGCA
3 swift_ACACA      92.4%
CCAGTTCCTGGAGGACCAAACAACAACAACTATGCAAATGTGGAACTCATTCTTGATATTGCTAAGCGGATTCCAGTGCA
4 human_ACACA      77.6%
```

```
CCAGTGCCTGGAGGACCAAACAACAACAACTATGCAAATGTGGAATTAATTCTTGATATTGCTAAAAGGATCCCAGTACA
5 gallus_ACACA     89.0%
CCAGTTCCAGGAGGACCAAACAACAACAACTATGCAAATGTGGAACTCATTCTCGATATTGCAAAACGCATTCCAGTGCA
6 alligator_ACACA  84.0%
CCTGTGCCAGGAGGGCCAAACAACAACAACTATGCAAATGTTGAATTGATTCTGGATATCGCAAAACGGATTCCAGTGCA

                          721          .          .          :          .          .          .
.         8 800
1 ruby_ACACA      100.0%
GGCTGTGTGGGCTGGCTGGGGCCATGCATCTGAGAACCCAAAATTACCAGAACTTCTCCACAAAAATGGGATTGCTTTTA
2 canna_ACACA      98.5%
GGCTGTGTGGGCTGGCTGGGGCCATGCATCTGAGAACCCAAAATTACCAGAACTTCTCCACAAAAATGGGATTGCTTTTA
3 swift_ACACA      92.4%
GGCTGTGTGGGCTGGCTGGGGCCATGCATCTGAGAACCCTAAACTACCAGAACTTCTTCACAAAAATGGAATTGCTTTCA
4 human_ACACA      77.6%
AGCAGTGTGGGCTGGCTGGGGTCATGCTTCTGAGAATCCCAAACTACCGGAACTTCTCTTGAAAAATGGCATTGCCTTCA
5 gallus_ACACA     89.0%
GGCTGTTTGGGCTGGCTGGGGCCATGCCTCCGAGAACCCAAAACTACCAGAACTTCTCCACAAAAATGGGATTGCTTTCA
6 alligator_ACACA  84.0%
GGCCGTGTGGGCTGGCTGGGGTCATGCTTCTGAGAACCCCAAACTGCCAGAGCTCCTTCACAAGAATGGAATTGCCTTCA

                          801          .          .          .          .          :          .
.         . 880
1 ruby_ACACA      100.0%
TGGGCCCTCCAAGCCAGGCAATGTGGGCCTTAGGAGATAAAATTGCTTCTTCAATCGTGGCTCAGACTGCAGGCATCCCG
2 canna_ACACA      98.5%
TGGGCCCTCCAAGCCAGGCAATGTGGGCCTTAGGAGATAAAATTGCTTCTTCAATCGTGGCTCAGACTGCGGGCATCCCG
3 swift_ACACA      92.4%
TGGGTCCTCCAAGCCAAGCAATGTGGGCCTTAGGAGATAAAATTGCTTCCTCAATAGTGGCTCAGACTGCTGGCATTCCA
4 human_ACACA      77.6%
TGGGTCCTCCAAGCCAGGCCATGTGGGCTTTAGGGGATAAGATTGCATCTTCCATAGTGGCTCAAACTGCAGGTATCCCA
5 gallus_ACACA     89.0%
TGGGTCCTCCAAGCCAAGCAATGTGGGCTTTAGGAGATAAAATTGCGTCGTCAATAGTGGCTCAGACTGCTGGCATCCCA
6 alligator_ACACA  84.0%
TGGGTCCCCCAAGCCAAGCCATGTGGGCTTTGGGGGATAAAATTGCATCTTCAATAGTGGCTCAAACAGCAGGGATCCCA

                          881          .          9          .          .          .          .
:         . 960
1 ruby_ACACA      100.0%
ACTCTTCCTTGGAGTGGCAGTGGTCTTCGGGTGGACTGGGAGGAAAATGATTTCCAGAAGCGTATCCTGAACGTTCCTCA
2 canna_ACACA      98.5%
ACTCTTCCTTGGAGTGGCAGTGGTCTTCGGGTGGACTGGGAGGAAAATGATTTCCAGAAGCGTATCCTGAACGTTCCTCA
3 swift_ACACA      92.4%
ACTCTTCCTTGGAGTGGCAGTGGTCTTCGAGTGGACTGGCAGGAAAATGATCTTCAGAAGCGTATCTTGAATGTTCCTCA
4 human_ACACA      77.6%
ACTCTTCCCTGGAGCGGCAGTGGTCTTCGTGTGGACTGGCAGGAAAATGATTTTTCAAAACGTATCTTAAATGTTCCCCA
5 gallus_ACACA     89.0%
ACTCTTCCTTGGAATGGCAGTGGTCTTCGAGTGGATTGGCAGGAGAATGATCTTCAGAAGCGTATCCTGAATGTTCCTCA
6 alligator_ACACA  84.0%
ACTCTCCCCTGGAGTGGCAGTGGCCTTCGTGTGGACTGGCAGGAGAACGATCTTCAGAAGCGCATCTTGAACGTCCCGCA

                          961          .          .          .          0          .          .
.         . 1040
1 ruby_ACACA      100.0%
GGAACTCTATGAGAAGGGCTATGTGAAAGATGCAGATGATGGCCTGAGGGCTGCTGAGGAGGTTGGCTACCCTGTCATGA
2 canna_ACACA      98.5%
GGAACTCTATGAAAAGGGCTATGTGAAAGATGCAGATGATGGCCTGAGGGCTGCTGAGGAGGTTGGCTACCCTGTCATGA
3 swift_ACACA      92.4%
GGAGTTATATGAAAAAGGCTACGTGAAAGACGCCGATGATGGCTTGCGGGCTGCTGAGGAGGTTGGCTACCCTGTCATGA
4 human_ACACA      77.6%
GGAGCTATATGAAAAAGGTTATGTGAAAGATGTGGATGATGGGCTACAGGCAGCTGAGGAAGTTGGATATCCAGTAATGA
5 gallus_ACACA     89.0%
GGAGCTGTATGAAAAAGGCTATGTGAAAGATGCAGACGATGGCCTGCGGGCTGCTGAGGAAGTTGGCTACCCTGTCATGA
6 alligator_ACACA  84.0%
GGAGCTGTATGAAAAGGGTTATGTGAAGGATGTGGACGATGGGTTGCGGGCTGCTGAGGAAGTTGGCTACCCAGTCATGA

                         1041          :          .          .          .          .          1
.         . 1120
1 ruby_ACACA      100.0%
TCAAGGCTTCTGAAGGAGGAGGAGGAAAAGGAATCAGGAAAGTTAACAATGCAGATGACTTCTCCAACCTCTTTAGACAG
2 canna_ACACA      98.5%
TCAAGGCTTCTGAAGGAGGAGGAGGAAAAGGAATCAGGAAAGTTAACAATGCAGATGACTTCTCCAACCTCTTTAGACAG
3 swift_ACACA      92.4%
TCAAGGCTTCTGAAGGAGGAGGAGGAAAAGGAATCAGGAAAGTTAACAATGCAGATGACTTCCCTAACCTATTTAGACAG
4 human_ACACA      77.6%
TCAAGGCCTCAGAGGGAGGAGGAGGGAAGGGAATTAGAAAAGTCAACAATGCAGATGACTTCCCTAATCTCTTCAGACAG
5 gallus_ACACA     89.0%
TCAAGGCCTCTGAAGGAGGAGGAGGGAAGGGAATTAGGAAAGTCAATAATGCGGATGACTTCCCCAACCTATTTAGACAG
6 alligator_ACACA  84.0%
TCAAGGCCTCTGAAGGTGGAGGAGGAAAAGGAATCAGGAAAGTTAACAATGCCGATGATTTCCCCAATCTCTTCAGACAG
```

```
                          1121         .         .         :         .         .         .
   .         2 1200
1 ruby_ACACA      100.0%
GTTCAAGCTGAAGTGCCAGGCTCTCCAATCTTTGTCATGAGGCTGGCCAAGCAGTCCCGGCACCTGGAGGTGCAGATCCT
2 canna_ACACA      98.5%
GTTCAAGCTGAAGTGCCAGGCTCTCCAATCTTTGTCATGAGGCTGGCCAAGCAGTCCCGGCACCTGGAGGTGCAGATCCT
3 swift_ACACA      92.4%
GTTCAAGCTGAGGTCCCAGGCTCTCCAATCTTTGTCATGAGACTAGCCAAGCAGTCGCGCCACCTGGAGGTGCAGATCCT
4 human_ACACA      77.6%
GTTCAAGCTGAAGTTCCTGGATCTCCCATATTTGTGATGAGACTAGCCAAACAATCTCGTCATCTGGAGGTGCAGATCTT
5 gallus_ACACA     89.0%
GTTCAGGCTGAAGTCCCAGGCTCTCCGATCTTTGTAATGAGGCTAGCCAAACAGTCCCGCCACTTGGAGGTGCAGATCCT
6 alligator_ACACA  84.0%
GTGCAAGCTGAAGTCCCAGGCTCGCCGATCTTCGTGATGAGACTGGCCAAGCAGTCCCGCCACCTGGAGGTGCAGATCCT

                          1201         .         .         .         .         :         .
   .         . 1280
1 ruby_ACACA      100.0%
GGCTGACCAGTACGGCAACGCCATCTCCCTCTTCGGCCGCGACTGCTCCGTGCAGCGCAGGCACCAGAAGATCATTGAGG
2 canna_ACACA      98.5%
GGCTGACCAGTACGGCAACGCCATCTCCCTCTTCGGCCGCGACTGCTCCGTGCAGCGCAGGCACCAGAAGATCATTGAGG
3 swift_ACACA      92.4%
GGCCGATCAGTACGGCAACGCCATCTCTCTCTTCGGCCGGGATTGCTCCGTGCAGCGCCGGCACCAGAAGATCATCGAGG
4 human_ACACA      77.6%
AGCGGACCAATATGGCAATGCTATCTCTTTGTTTGGTCGTGATTGCTCTGTACAACGCAGGCATCAGAAGATTATTGAAG
5 gallus_ACACA     89.0%
GGCAGACCAGTATGGCAATGCCATCTCTCTCTTTGGTCGGGATTGCTCCGTGCAACGCAGGCATCAGAAGATTATTGAAG
6 alligator_ACACA  84.0%
TGCAGATCAGTATGGCAATGCCATTTCCCTCTTTGGCCGTGATTGCTCAGTGCAGCGCAGGCATCAGAAGATCATTGAAG

                          1281         .         3         .         .         .         .
   :         . 1360
1 ruby_ACACA      100.0%
AAGCTCCTGCTTCTATTGCCACTTCGACAGTCTTTGAGCACATGGAACAGTGTGCAGTGAAGCTTGCCAAGATGGTGGGC
2 canna_ACACA      98.5%
AAGCTCCTGCTTCTATTGCCACTTCGGCGGTCTTTGAGCACATGGAACAGTGTGCAGTGAAGCTTGCCAAGATGGTGGGC
3 swift_ACACA      92.4%
AAGCACCTGCCTCTATTGCCACTTCAGTGGTGTTTGAGCACATGGAGCAGTGTGCAGTGAAGCTTGCCAAAATGGTGGGG
4 human_ACACA      77.6%
AAGCACCTGCTACTATTGCTACTCCAGCAGTATTTGAACACATGGAACAGTGTGCGGTGAAACTTGCCAAAATGGTGGGT
5 gallus_ACACA     89.0%
AAGCACCTGCTTCTATTGCAACTTCGGTGGTATTTGAGCACATGGAACAGTGTGCAGTGAAGCTTGCAAAAATGGTGGGG
6 alligator_ACACA  84.0%
AGGCTCCAGCATCCATTGCTACCATTGCGGTGTTCGAACACATGGAGCAGTGCGCAGTGAAGCTTGCCAAGATGGTGGGG

                          1361         .         .         .         4         .         .
   .         . 1440
1 ruby_ACACA      100.0%
TACGTGAGTGCAGGCACTGTGGAGTACCTGTACAGCCAGGATGGCAGCTTCTACTTTCTGGAGTTGAATCCCCGCCTGCA
2 canna_ACACA      98.5%
TACGTGAGTGCAGGCACTGTGGAGTACCTGTACAGCCAGGATGGCAGCTTCTACTTTCTGGAGTTGAATCCCCGCCTGCA
3 swift_ACACA      92.4%
TACGTGAGTGCAGGCACTGTGGAGTACCTGTACAGCCAGGACGGCAGCTTCTACTTCCTGGAGCTAAATCCCCGCCTGCA
4 human_ACACA      77.6%
TATGTGAGTGCTGGGACTGTGGAATACCTGTACAGCCAGGATGGCAGCTTCTACTTTCTGGAATTGAATCCTCGGCTGCA
5 gallus_ACACA     89.0%
TATGTGAGTGCGGGCACTGTGGAATACCTGTACAGCCAGGATGGCAGCTTCTACTTTCTGGAGTTGAATCCCCGTCTGCA
6 alligator_ACACA  84.0%
TATGTGAGTGCTGGGACAGTGGAGTACCTCTACAGCCAGGACGGGAGCTTCTACTTCCTTGAGCTGAATCCCCGGCTGCA

                          1441         :         .         .         .         .         5
   .         . 1520
1 ruby_ACACA      100.0%
AGTGGAGCACCCCTGCACTGAGATGGTGGCTGATGTCAACCTGCCAGCAGCACAGCTCCAGATTGCCATGGGGATTCCTC
2 canna_ACACA      98.5%
AGTGGAGCACCCCTGCACTGAGATGGTGGCTGATGTCAACCTGCCAGCAGCACAGCTCCAGATTGCCATGGGGATTCCTC
3 swift_ACACA      92.4%
GGTGGAGCACCCCTGCACTGAGATGGTGGCTGATGTCAACCTGCCGGCAGCACAGCTCCAGATTGCCATGGGGATTCCCC
4 human_ACACA      77.6%
GGTAGAGCACCCTTGTACAGAGATGGTGGCTGATGTCAATCTCCCTGCAGCACAGCTCCAGATTGCCATGGGGATTCCTC
5 gallus_ACACA     89.0%
AGTGGAGCACCCCTGCACCGAGATGGTAGCTGATGTTAATCTTCCTGCAGCACAGCTCCAGATTGCCATGGGGATTCCAC
6 alligator_ACACA  84.0%
GGTGGAACACCCCTGCACGGAGATGGTGGCTGACGTGAACCTGCCTGCAGCACAGCTTCAGATTGCCATGGGGATTCCTC

                          1521         .         .         :         .         .         .
   .         6 1600
1 ruby_ACACA      100.0%
TCCACAGGATCAAGGATATCCGGGTGATGTATGGTGTTTCTCCCTGGGGAGATGGAGATATTGATTTTGAGAATTCAGCC
2 canna_ACACA      98.5%
TCCACAGGATCAAGGATATCCGGGTGATGTATGGTGTTTCTCCCTGGGGAGATGGAGATATTGATTTTGAGAATTCAGCC
```

```
3 swift_ACACA      92.4%
TCCACAGAATCAAGGATATCCGGGTGATGTATGGTGTGTCTCCATGGGGAGATACGTCCATTGATTTTGAGAACTCAGCC
4 human_ACACA      77.6%
TATATAGAATCAAGGATATCCGTATGATGTATGGGGTATCTCCCTGGGGTGATTCTCCCATTGATTTTGAAGATTCTGCA
5 gallus_ACACA     89.0%
TCCACCGTATCAAGGATATCCGAGTGATGTATGGTGTTTCCCCATGGGGAGATGGATCTATTGATTTTGAGAATTCAGCC
6 alligator_ACACA  84.0%
TTCACCGGATCAAAGATATCCGAGTTCTGTATAGTGCTTCTCCATGGGGAGACACACCCGTTGACTTTGAGAATTCAGCC

                         1601          .         .         .         .         :         .
.         . 1680
1 ruby_ACACA      100.0%
CATGTCCCCTCTCCTCGTGGCCACGTCATCGCTGCACGGATCACCAGTGAGAATCCTGATGAGGGATTTAAACCCAGTTC
2 canna_ACACA      98.5%
CATGTCCCCTCTCCTCGTGGCCACGTCATTGCTGCACGGATCACCAGTGAGAATCCTGATGAGGGATTTAAACCCAGTTC
3 swift_ACACA      92.4%
CACGTCCCCAGTCCACGCGGCCACGTCATCGCTGCCCGTATCACCAGTGAGAATCCTGATGAAGGATTTAAGCCCAGTTC
4 human_ACACA      77.6%
CACGTTCCTTGTCCAAGGGGCCATGTTATTGCTGCTCGGATCACTAGTGAAAATCCAGATGAGGGTTTTAAGCCCAGCTC
5 gallus_ACACA     89.0%
CATGTCCCCTGTCCACGTGGCCATGTTATTGCTGCACGTATCACCAGTGAGAATCCTGATGAGGGATTTAAGCCCAGTTC
6 alligator_ACACA  84.0%
CATGTCCCCAGTCCACGTGGTCATGTGATTGCTGCTCGTATCACCAGTGAGAATCCAGATGAGGGTTTTAAGCCCAGCTC

                         1681          .         7         .         .         .         .
:         . 1760
1 ruby_ACACA      100.0%
TGGTACAGTTCAGGAACTGAATTTCCGCAGCAATAAGAATGTCTGGGGCTATTTCAGTGTTGCTGCTGCAGGAGGGCTCC
2 canna_ACACA      98.5%
TGGTACAGTTCAGGAACTGAATTTCCGCAGCAATAAGAATGTCTGGGGCTATTTCAGTGTTGCTGCTGCAGGAGGGCTCC
3 swift_ACACA      92.4%
TGGTACAGTTCAGGAGCTGAATTTCCGCAGCAATAAGAATGTCTGGGGCTATTTCAGTGTTGCTGCTGCAGGAGGGCTTC
4 human_ACACA      77.6%
AGGAACAGTTCAGGAGCTAAATTTCCGCAGCAATAAGAATGTTTGGGGATATTTCAGTGTTGCTGCTGCAGGGGGACTTC
5 gallus_ACACA     89.0%
TGGTACAGTCCAGGAACTGAATTTCCGCAGCAATAAGAATGTTTGGGGCTATTTCAGTGTTGCTGCTGCAGGAGGGCTGC
6 alligator_ACACA  84.0%
TGGAACAGTCCAAGAACTGAATTTCCGCAGCAATAAGAATGTCTGGGGTTATTTCAGTGTCGCGGCTGCAGGGGGACTTC

                         1761          .         .         .         8         .         .
.         . 1840
1 ruby_ACACA      100.0%
ATGAATTTGCTGATTCTCAGTTTGGTCACTGCTTCTCCTGGGGAGAAAATCGTGAAGAAGCCATCTCGAACATGGTGGTG
2 canna_ACACA      98.5%
ATGAATTTGCTGATTCTCAGTTTGGTCACTGCTTCTCCTGGGGAGAAAATCGTGAAGAAGCCATCTCGAACATGGTGGTG
3 swift_ACACA      92.4%
ATGAATTTGCTGATTCTCAGTTTGGTCACTGCTTCTCTTGGGGAGAAAATCGTGAAGAAGCCATCTCAAACATGGTAGTG
4 human_ACACA      77.6%
ATGAATTTGCTGATTCTCAGTTTGGTCACTGCTTTTCTTGGGGAGAAAACAGAGAAGAGGCAATTTCAAACATGGTGGTG
5 gallus_ACACA     89.0%
ATGAATTTGCTGATTCTCAGTTTGGTCACTGCTTCTCTTGGGGAGAGAATCGTGAAGAAGCCATCTCAAACATGGTGGTG
6 alligator_ACACA  84.0%
ATGAATTTGCTGACTCTCAGTTTGGGCACTGCTTCTCATGGGGAGAAAATCGTGAAGAAGCCATCTCTAACATGGTGGTG

                         1841          :         .         .         .         .         9
.         . 1920
1 ruby_ACACA      100.0%
GCTTTGAAGGAGCTGTCCATCCGAGGGGATTTCCGAACCACTGTTGAGTACTTGATTAAACTGTTGGAAACAGAAAGCTT
2 canna_ACACA      98.5%
GCTTTGAAGGAGCTGTCCATCCGAGGGGATTTCCGAACCACTGTTGAGTACTTGATTAAACTGTTGGAAACAGAAAGCTT
3 swift_ACACA      92.4%
GCTTTGAAGGAGCTGTCCATCCGAGGGGATTTCCGAACCACTGTTGAGTACTTGATTAAACTGTTGGAAACAGAGAGTTT
4 human_ACACA      77.6%
GCTTTGAAGGAGCTGTCTATTCGGGGTGACTTTCGAACTACAGTTGAATACCTGATCAAATTGTTAGAGACTGAAAGCTT
5 gallus_ACACA     89.0%
GCTTTGAAGGAGCTGTCCATCCGAGGGGATTTCCGAACCACTGTTGAATACTTGATAAAACTGTTGGAAACAGAAAGCTT
6 alligator_ACACA  84.0%
GCTTTGAAGGAACTGTCTATCCGAGGGGATTTCCGAACCACTGTCGAGTACTTGATTAAACTGCTGGAGACAGAAAGCTT

                         1921          .         .         :         .         .         .
.         0 2000
1 ruby_ACACA      100.0%
CCAGCAGAACCGGATCGACACGGGCTGGTTGGATCGGCTGATTGCCGAGAAGGTTCAGGCTGAAAGGCCTGATACCATCC
2 canna_ACACA      98.5%
CCAGCAGAACCGGATCGACACGGGCTGGTTGGATCGGCTGATTGCCGAGAAGGTTCAGGCTGAAAGGCCTGATACCATCC
3 swift_ACACA      92.4%
CCAGCAGAACCGCATCGACACCGGCTGGTTGGATCGGCTCATTGCTGAGAAAGTTCAGGCTGAACGGCCTGACACCATGC
4 human_ACACA      77.6%
TCAGATGAACAGAATTGATACTGGCTGGCTGGACAGACTGATAGCAGAAAAAGTACAGGCTGAGCGACCTGACACCATGT
5 gallus_ACACA     89.0%
CCAGCAGAACCGCATTGACACTGGCTGGTTGGATCGGCTTATTGCTGAGAAAGTGCAGGCTGAAAGGCCTGATACCATGC
```

```
6 alligator_ACACA  84.0%
CCAACACAACCGCATTGACACTGGCTGGCTGGACAGGCTCATTGCAGAGAAAGTTCAGGCAGAGAGGCCTGATACCATGC

                         2001         .         .         .         .         :         .
.        . 2080
1 ruby_ACACA      100.0%
TGGGAGTGGTGTGTGGAGCTCTGCATGTGGCTGATGTGAACTTCAGAAACAGTGTCTCAAACTTCCTGCACTCTCTGGAA
2 canna_ACACA      98.5%
TGGGAGTGGTGTGTGGAGCTCTGCATGTGGCTGATGTGAACTTCAGAAACAGTGTCTCAAACTTCCTGCACTCTCTGGAA
3 swift_ACACA      92.4%
TGGGGGTAGTGTGTGGAGCTCTGCACGTGGCTGATGTGAGCCTCCGAAACAGTGTCTCAAACTTCCTACACTCTCTAGAA
4 human_ACACA      77.6%
TGGGGGTTGTGTGTGGTGCCCTCCACGTGGCAGATGTGAGCCTGCGGAATAGCGTCTCTAACTTCCTTCACTCCTTAGAA
5 gallus_ACACA     89.0%
TAGGAGTGGTATGTGGAGCTCTTCATGTGGCTGATGTGAGCTTTCGAAACAGCGTCTCAAACTTCCTGCACTCTTTAGAA
6 alligator_ACACA  84.0%
TAGGAGTGGTGTGTGGGGCTCTGCATGTGGCTGACGTGAGCTTCCGTAACAGTGTCTCCAACTTCTTGCACTCTCTCGAG

                         2081         .         1         .         .         .         .
:        . 2160
1 ruby_ACACA      100.0%
AGGGGCCAAGTGCTGCCTGCTCATACTCTGCTGAACACTGTGGATGTGGAGCTCATCTATGAAGGACGCAAGTATGTGCT
2 canna_ACACA      98.5%
AGGGGCCAAGTGCTGCCTGCTCATACTCTGCTGAACACTGTGGATGTGGAGCTCATCTATGAAGGACGCAAGTATGTGCT
3 swift_ACACA      92.4%
AGGGGCCAAGTCCTGCCTGCTCATACTCTGCTGAACACTGTGGATGTGGAACTCATATATGAAGGACGGAAGTATGTGCT
4 human_ACACA      77.6%
AGGGGTCAAGTCCTTCCTGCTCATACACTTCTGAATACAGTAGATGTTGAACTTATCTATGAGGGAGTCAAGTATGTACT
5 gallus_ACACA     89.0%
AGGGGCCAAGTCCTGCCTGCTCATACTTTGCTAAACACTGTGGATGTGGAACTCATCTATGAAGGACGGAAATATGTGTT
6 alligator_ACACA  84.0%
AGGGGTCAGGTCCTGCCTGCTCATACTCTGCTGAACACAGTGGATGTGGAACTCATCTACGAAGGACGGAAATATGTGCT

                         2161         .         .         .         2         .         .
.        . 2240
1 ruby_ACACA      100.0%
GAAGGTGACCCGGCAGTCTCCCAATTCCTACGTGGTCATCATGAACAATTCCTGTGTGGAAGTTGATGTGCACCGACTGA
2 canna_ACACA      98.5%
GAAGGTGACCCGGCAGTCTCCCAATTCCTACGTGGTCATCATGAACAACTCCTGTGTGGAAGTTGATGTGCACCGACTGA
3 swift_ACACA      92.4%
GAAGGTGACCCGGCAGTCTCCCAATTCCTACGTGGTGATCATGAACAACTCCTGTGTGGAAGTTGATGTGCACAGACTGA
4 human_ACACA      77.6%
TAAGGTGACTCGACAGTCCCCCAACTCCTATGTGGTGATCATGAATGGCTCATGTGTAGAAGTAGATGTACATCGGCTGA
5 gallus_ACACA     89.0%
GAAGGTGACCCGACAGTCTCCCAATTCCTACGTGGTCATCATGAACAGCTCTTGTGTGGAAGTTGATGTGCACAGACTGA
6 alligator_ACACA  84.0%
GAAGGTGACCAGGCAGTCACCCAATTCCTATGTGGTCATCATGAACAACTCCTGCGTGGAGGTTGATGTGCACCGATTGA

                         2241         :         .         .         .         .         3
.        . 2320
1 ruby_ACACA      100.0%
GTGATGGGGGGCTGCTCCTCTCCTATGATGGGAGCAGCTATACCACCTACATGAAAGAAGAAGTGGACAGGTATCGCATC
2 canna_ACACA      98.5%
GTGATGGGGGGCTGCTCCTCTCCTATGATGGGAGCAGCTATACCACCTACATGAAAGAAGAAGTGGACAGGTATCGCATC
3 swift_ACACA      92.4%
GTGATGGGGGGCTGCTCCTGTCCTATGATGGGAGCAGCTACACTACCTACATGAAAGAAGAAGTGGACAGGTATCGCATC
4 human_ACACA      77.6%
GTGACGGTGGACTGCTCTTGTCCTATGATGGCAGCAGTTATACTACGTATATGAAAGAGGAAGTGGATAGATATCGCATC
5 gallus_ACACA     89.0%
GCGATGGAGGGCTGCTCCTATCTTACGATGGTAGCAGCTACACCACCTACATGAAAGAAGAAGTGGACAGGTATCGCATC
6 alligator_ACACA  84.0%
GTGATGGAGGACTGCTCTTATCCTACGATGGCAGCAGCTACACCACCTACATGAAGGAAGAAGTGGACAGATATCGGATC

                         2321         .         .         :         .         .         .
.        4 2400
1 ruby_ACACA      100.0%
ACCATAGGTAACAAGACCTGTGTGTTTGAGAAGGAGAATGACCCCTCCATTCTGCGCTCCCCTTCGGCCGGGAAGCTCAT
2 canna_ACACA      98.5%
ACCATAGGTAACAAGACCTGTGTGTTTGAGAAGGAGAACGACCCCTCCATTCTGCGCTCCCCTTCGGCCGGGAAGCTCAT
3 swift_ACACA      92.4%
ACTATCGGTAACAAGACCTGTGTGTTTGAGAAGGAGAATGATCCCTCCATTCTGCGCTCGCCTTCGGCTGGGAAGCTTAT
4 human_ACACA      77.6%
ACAATTGGCAATAAAACCTGTGTGTTTGAGAAGGAAAATGACCCATCGGTGATGCGCTCACCTTCTGCTGGGAAGTTAAT
5 gallus_ACACA     89.0%
ACTATAGGTAACAAGACCTGTGTGTTTGAAAAGGAAAATGATCCTTCTATTCTGCGCTCACCTTCGGCTGGGAAGCTTAT
6 alligator_ACACA  84.0%
ACCATAGGTAATAAGACCTGTGTGTTCGAGAAGGAGAATGACCCATCCATCCTGCGCTCGCCTTCTGCTGGGAAGCTCAT

                         2401         .         .         .         .         :         .
.        . 2480
1 ruby_ACACA      100.0%
```

```
CCAGTATGTGGTGGAGGATGGGGGACACGTGTTTGCAGGCCAGTGCTTTGCAGAAATAGAGGTGATGAAAATGGTGATGA
2 canna_ACACA      98.5%
CCAGTATGTGGTGGAGGATGGGGGACACGTGTTTGCAGGCCAGTGCTTTGCAGAAATAGAGGTGATGAAAATGGTGATGA
3 swift_ACACA      92.4%
CCAGTATGTGGTGGAGGATGGGGGACACGTGTTTTCAGGCCAGTGCTTTGCAGAAATAGAGGTGATGAAAATGGTGATGA
4 human_ACACA      77.6%
CCAGTACATTGTAGAAGATGGAGGTCATGTGTTTGCCGGCCAGTGCTATGCTGAGATTGAGGTAATGAAGATGGTAATGA
5 gallus_ACACA     89.0%
CCAGTATGTGGTGGAGGATGGGGGACACGTGTTTGCAGGCCAATGCTTTGCAGAAATAGAGGTGATGAAAATGGTGATGA
6 alligator_ACACA  84.0%
CCAGTACGTGGTGGAGGATGGAGGACACGTGTTTGCAGGCCAGTGCTTTGCGGAAATAGAGGTGATGAAGATGGTGATGA

                         2481          .         5         .         .         .         .
:         . 2560
1 ruby_ACACA      100.0%
CACTAACAGCAGGGGAATCAGGCTGCATCCATTATGTCAAACGCCCAGGGGCTGTCTTGGATCCAGGCTGTGTGATTGCC
2 canna_ACACA      98.5%
CACTAACAGCAGGGGAATCAGGCTGCATCCATTATGTCAAACGCCCAGGGGCTGTCTTGGATCCAGGCTGTGTGATTGCC
3 swift_ACACA      92.4%
CACTAACAGCAGGAGAATCAGGCTGCATCCATTATGTCAAACGCCCAGGGGCAGTCTTGGATCCAGGCTGTGTGATTGCC
4 human_ACACA      77.6%
CCTTAACAGCTGTGGAGTCTGGCTGTATCCATTACGTCAAGCGACCTGGAGCAGCTCTTGACCCTGGCTGTGTACTAGCC
5 gallus_ACACA     89.0%
CACTAACAGCAGGAGAGTCAGGCTGCATCCATTATGTCAAACGCCCCGGGGCAGTGCTGGATCCAGGCTGTGTGATTGCC
6 alligator_ACACA  84.0%
CACTGACAGCAGCAGAATCGGGCTGCATCCACTATGTCAAACGCCCTGGGGCAGCACTGGACCCCGGCTGTGTGATTGCC

                         2561          .         .         .         6         .         .
.         . 2640
1 ruby_ACACA      100.0%      AAGCTCCAGCTGGATGATCCCAGCAGAGTTCAGCA-----------
-GGCTGAGCTGCACACAGGGGCCTTGCCACAGAT
2 canna_ACACA      98.5%      AAGCTCCAGCTGGATGCTGCCAGCAGGGTTCAGCA-----------
-GGCTGAACTGCACACAGGGGCCTTGCCACAGAT
3 swift_ACACA      92.4%
AAGCTCCAGCTGGATGATCCCAGCAGGGTTCAGCAAAAAATTCAGCAGGCTGAGCTGCACACGGGCGCTTTGCCGCAGAT
4 human_ACACA      77.6%      AAAATGCAACTGGACAACCCCAGCAAGGTTCAGCA-----------
-GGCTGAACTTCACACAGGTAGTCTGCCACGGAT
5 gallus_ACACA     89.0%      AAACTCCAGCTGGATGATCCCAGCAGGGTTCAGCA-----------
-GGCTGAACTGCACACAGGCACCTTGCCACAGAT
6 alligator_ACACA  84.0%      AAGCTGCAGTTGGATGATCCCAGCAGGGTTCAGCA-----------
-GGCTGAGCTGCACACTGGTACCTTGCCAAAGAT

                         2641          :         .         .         .         .         7
.         . 2720
1 ruby_ACACA      100.0%
CCAGAGCACAGCACTTCGGGGAGAGAAACTCCATCGCATCTTCCATTACGTCCTGGACAACCTGGTCAATGTGATGAATG
2 canna_ACACA      98.5%
CCAGAGCACAGCACTTCGGGGAGAGAAACTCCATCGCATCTTCCATTACGTCCTGGACAACCTGGTCAATGTGATGAATG
3 swift_ACACA      92.4%
CCAGAGCACAGCGCTTCGAGGCGAGAAACTCCATCGCATCTTCCATTATGTGCTGGATAACCTGGTCAATGTGATGAATG
4 human_ACACA      77.6%
CCAGAGCACGGCACTCAGAGGCGAGAAACTCCATCGAGTGTTCCATTATGTCCTGGATAATCTGGTCAATGTAATGAATG
5 gallus_ACACA     89.0%
CCAGAGCACAGCACTTCGAGGCGAAAAACTCCATCGCATCTTCCATTATGTCCTGGATAACCTGGTCAACGTGATGAATG
6 alligator_ACACA  84.0%
CCAGAGCACAGCACTTCGAGGCGAGAAGCTCCATCGTGTCTTCCATTATGTCCTTGATAACCTGGTCAATGTGATGAATG

                         2721          .         .         :         .         .         .
.         8 2800
1 ruby_ACACA      100.0%
GGTACTGCCTGCCAGAGCCCTTCTTCAGCAGCAAGGTGAAAGGCTGGGTTGAGCGACTGATGAAGACCCTGAGAGATCCC
2 canna_ACACA      98.5%
GGTACTGCCTGCCAGAGCCCTTCTTCAGCAGCAAGGTGAAAGGCTGGGTTGAGCGACTGATGAAGACCCTGAGAGATCCC
3 swift_ACACA      92.4%
GGTACTGCCTGCCAGAGCCCTACTTCAGCAGCAAGGTGAAAGGCTGGGTTGAGCGACTAATGAAGACACTGAGAGATCCA
4 human_ACACA      77.6%
GATACTGCCTTCCAGATCCTTTCTTTAGCAGCAAGGTAAAAGACTGGGTAGAGCGATTGATGAAAACCCTCAGAGATCCC
5 gallus_ACACA     89.0%
GGTACTGCCTGCCAGAGCCCTACTTTAGCAGCAAGGTGAAGGGCTGGGTTGAGCGACTAATGAAGACACTGAGAGATCCA
6 alligator_ACACA  84.0%
GATACTGCCTGCCGGAGCCATACTTTGGCAGGAAGGTGAAGGATTGGGTTGAGCGGCTAATGAAGACCCTGAGAGACCCA

                         2801          .         .         .         .         :         .
.         . 2880
1 ruby_ACACA      100.0%
TCTTTGCCTCTGCTGGAACTTCAGGACATCATGACCAGTGTTTCCGGACGGATCCCACCCAATGTTGAGAAGTCCATCAA
2 canna_ACACA      98.5%
TCTTTGCCTCTGCTGGAACTTCAGGACATCATGACCAGTGTTTCCGGACGGATCCCACCCAATGTTGAGAAGTCCATCAA
3 swift_ACACA      92.4%
TCTTTGCCTCTGCTGGAACTTCAGGACATCATGACCAGTGTCTCTGGACGGATCCCACCCAATGTAGAGAAGTCCATCAA
4 human_ACACA      77.6%
```

```
TCCCTGCCTCTCCTAGAATTGCAAGATATTATGACCAGTGTGTCTGGCCGCATTCCCCCCAATGTGGAGAAGTCTATCAA
5 gallus_ACACA     89.0%
TCTTTGCCTCTGCTGGAACTTCAGGACATCATGACCAGTGTTTCTGGACGGATTCCACCCAATGTGGAGAAGTCCATCAA
6 alligator_ACACA  84.0%
TCTTTGCCCCTCCTGGAGCTTCAGGACATCATGACCAGTGTCTCTGGGCGCATCCCACCCAATGTGGAGAAGTCCATCAA

                         2881          .         9         .         .         .         .
:         . 2960
1 ruby_ACACA      100.0%
GAAGGAGATGGCCCAGTATGCCAGCAACATCACATCAGTTCTTTGCCAGTTTCCCAGCCAACAGATTGCCAATATCTTGG
2 canna_ACACA      98.5%
GAAGGAGATGGCCCAGTATGCTAGCAACATCACATCAGTTCTTTGCCAGTTTCCCAGCCAACAGATTGCCAATATCTTGG
3 swift_ACACA      92.4%
GAAGGAGATGGCCCAATATGCCAGCAACATCACATCAGTCCTTTGCCAGTTTCCCAGCCAACAGATTGCCAACATCTTGG
4 human_ACACA      77.6%
GAAGGAAATGGCTCAGTATGCTAGCAACATCACATCAGTCCTCTGTCAGTTTCCCAGCCAGCAGATTGCAAACATCCTAG
5 gallus_ACACA     89.0%
GAAGGAGATGGCCCAATATGCCAGCAACATCACGTCAGTCCTTTGCCAGTTTCCCAGCCAACAGATTGCCAATATCTTGG
6 alligator_ACACA  84.0%
GAAGGAGATGGCCCAATATGCCAGCAACATCACATCTGTTCTCTGCCAGTTTCCCAGCCAGCAGATTGCCAACATTTTGG

                         2961          .         .         .         0         .         .
.         . 3040
1 ruby_ACACA      100.0%
ATAGCCACGCGGCCACCTTGAACCGCAAGTCGGAACGTGAGGTCTTCTTCATGAACACCCAGAGCATTGTGCAGCTTGTA
2 canna_ACACA      98.5%
ATAGCCACGCGGCCACCTTGAACCGCAAGTCAGAACGTGAGGTCTTCTTCATGAACACCCAGAGCATTGTGCAGCTTGTA
3 swift_ACACA      92.4%
ATAGCCACGCAGCCACCTTGAACCGCAAGTCGGAGCGTGAGGTCTTCTTCATGAACACACAGAGCATTGTGCAGCTTGTA
4 human_ACACA      77.6%
ATAGCCATGCAGCTACATTGAACCGGAAATCTGAACGGGAAGTCTTCTTTATGAATACTCAGAGCATTGTTCAGCTGGTA
5 gallus_ACACA     89.0%
ATAGCCATGCAGCCACCTTGAACCGCAAATCAGAGCGTGAGGTCTTTTTCATGAACACTCAGAGTATTGTGCAGCTTGTA
6 alligator_ACACA  84.0%
ACAGCCATGCAGCCACGCTAAATCGCAAATCTGAGCGGGAGGTCTTCTTTATGAACACTCAGAGTATTGTTCAGTTGGTG

                         3041          :         .         .         .         .         1
.         . 3120
1 ruby_ACACA      100.0%
CAGAGGTACCGGAGTGGCATTCGAGGCCACATGAAAGCTGTGGTGATGGATCTGCTGCGTCAGTATCTGAAGGTGGAGAC
2 canna_ACACA      98.5%
CAGAGGTACCGGAGTGGCATTCGAGGCCACATGAAAGCTGTGGTGATGGATCTGCTGCGTCAGTATCTGAAGGTGGAGAC
3 swift_ACACA      92.4%
CAGAGGTACCGCAGTGGTATTCGGGGTCACATGAAGGCTGTAGTGATGGATCTGCTCCGTCAGTATCTGAAGGTGGAGAC
4 human_ACACA      77.6%
CAGAGGTACCGAAGTGGCATCCGAGGCCACATGAAGGCTGTGGTGATGGATCTGCTCCGGCAGTACCTGCGAGTAGAGAC
5 gallus_ACACA     89.0%
CAGAGGTACCGGAGTGGTATTCGGGGTCACATGAAAGCAGTGGTCATGGATTTGCTCCGTCAATATCTGAAGGTGGAGAC
6 alligator_ACACA  84.0%
CAGAGGTACCGAAGTGGTATTCGAGGCCACATGAAGGCTGTGGTGATGGATCTTCTTCGTCAGTACCTGAAGGTGGAGAC

                         3121          .         .         :         .         .         .
.        2 3200
1 ruby_ACACA      100.0%
TCAATTTCAGCATGGTCACTATGACAAGTGTGTCTTTACCCTTCGGGAAGAGAATAAAAGTGATATGAATGCTGTATTGA
2 canna_ACACA      98.5%
TCAATTTCAGCATGGTCACTATGACAAGTGTGTCTTTACCCTTCGGGAAGAGAATAAAAGTGATATGAATGCTGTATTGA
3 swift_ACACA      92.4%
TCAGTTTCAGCATGGTCACTATGACAAGTGTGTCTTTACCCTTCGGGAAGAGAATAAAAGTGATATGAATGCTGTATTGA
4 human_ACACA      77.6%
ACAATTCCAGAATGGTCACTATGACAAATGTGTATTCGCCCTCCGAGAAGAGAATAAAAGTGACATGAACACTGTACTGA
5 gallus_ACACA     89.0%
TCAGTTTCAGCATGGTCACTATGACAAGTGTGTCTTTGCCCTTCGGGAAGAGAATAAAAGCGACATGAATGCTGTATTGA
6 alligator_ACACA  84.0%
TCAGTTCCAGCACGGTCACTATGACAAGTGTGTCTTTGCCCTTCGGGAAGAGAACAAAAGTGACATGAACACTGTATTGA

                         3201          .         .         .         .         :         .
.         . 3280
1 ruby_ACACA      100.0%
ACTACATCTTCTCACATGCTCAGGTCACCAAGAAGAACCTGCTTGTTACAATGCTCATTGACCAGCTCTGTGGCCGTGAC
2 canna_ACACA      98.5%
ACTACATCTTCTCACATGCTCAGGTCACCAAGAAGAACCTGCTTGTTACAATGCTCATTGACCAGCTCTGTGGCCGTGAC
3 swift_ACACA      92.4%
ACTACATCTTCTCTCATGCTCAAGTCACTAAGAAGAACCTGCTTGTTACAATGCTCATTGACCAGCTATGTGGCCGTGAC
4 human_ACACA      77.6%
ACTACATCTTCTCTCACGCTCAAGTCACCAAGAAGAATCTTCTGGTCACAATGCTTATTGATCAGTTGTGTGGCCGGGAC
5 gallus_ACACA     89.0%
ACTACATCTTCTCACATGCTCAGGTCACCAAGAAGAACCTGCTTGTCACAATGCTCATTGACCAGCTCTGTGGCCGTGAC
6 alligator_ACACA  84.0%
ACTACATCTTCTCCCACGCCCAGGTCACCAAGAAGAACCTGCTTGTAACAATGCTCATTGACCAACTGTGTGGCCGAGAC
```

```
                              3281            .           3           .           .           .           .
  :          . 3360
  1 ruby_ACACA      100.0%
  CCCACCTTGACAGATGAGCTGATTAATATCCTGACAGAGCTGACCCAGCTCAGCAAGACGACCAACGCCAAAGTGGCCCT
  2 canna_ACACA      98.5%
  CCCACCTTGACAGATGAGCTGATTAATATCCTGACAGAGCTGACCCAGCTCAGCAAGACGACCAACGCCAAAGTGGCCCT
  3 swift_ACACA      92.4%
  CCCACCTTGACAGATGAGCTCATTAATATTCTGACTGAGCTGACCCAGCTCAGCAAGACAACCAACGCTAAAGTGGCCCT
  4 human_ACACA      77.6%
  CCTACTCTCACTGATGAGCTGCTGAATATTCTCACAGAGCTAACTCAACTCAGTAAGACCACCAATGCCAAAGTAGCACT
  5 gallus_ACACA     89.0%
  CCCACCCTGACAGATGAGCTGATCAATATTCTGACAGAGCTGACCCAGCTCAGCAAGACAACCAACGCCAAAGTGGCGCT
  6 alligator_ACACA  84.0%
  CCCACCTTGACAGACGAGTTAATTAATATTCTGACTGAGCTGACCCAGCTGAGCAAGACGACCAATGCTAAAGTAGCGCT

                              3361            .           .           .           4           .           .
  .          . 3440
  1 ruby_ACACA      100.0%
  GCGAGCACGGCAGGTTCTCATTGCTTCCCATTTGCCATCCTACGAGCTGAGGCACAACCAGGTGGAGTCCATCTTCCTCT
  2 canna_ACACA      98.5%
  GCGAGCACGGCAGGTTCTCATTGCTTCCCATTTGCCATCCTACGAGCTGAGGCACAACCAGGTGGAGTCCATCTTCCTCT
  3 swift_ACACA      92.4%
  GCGAGCACGGCAGGTTCTCATTGCTTCCCATTTGCCATCCTACGAGCTGCGTCACAACCAGGTGGAGTCCATCTTCCTGT
  4 human_ACACA      77.6%
  TCGAGCACGCCAGGTTCTTATTGCCTCCCATTTGCCATCATATGAGCTTCGCCATAACCAAGTAGAGTCTATCTTCCTAT
  5 gallus_ACACA     89.0%
  GCGGGCACGGCAGGTTCTCATTGCTTCCCATTTGCCGTCCTACGAGCTGCGTCACAACCAGGTGGAGTCCATCTTCCTAT
  6 alligator_ACACA  84.0%
  GCGGGCACGTCAGGTTCTCATTGCTTCCCATTTGCCGTCATACGAGCTGCGTCACAACCAGGTGGAGTCCATCTTCCTAT

                              3441            :           .           .           .           .           5
  .          . 3520
  1 ruby_ACACA      100.0%
  CTGCTATTGACATGTATGGACACCAGTTCTGCATTGAGAACCTCCAGAAACTCATTTTGTCTGAGACATCCATCTTTGAT
  2 canna_ACACA      98.5%
  CTGCTATTGACATGTATGGACACCAGTTCTGCATTGAGAACCTCCAGAAACTCATTTTGTCTGAGACATCCATCTTTGAT
  3 swift_ACACA      92.4%
  CTGCTATCGACATGTATGGACACCAGTTCTGCATTGAGAACTTGCAGAAACTCATTTTGTCTGAGACATCCATCTTTGAT
  4 human_ACACA      77.6%
  CAGCTATTGACATGTATGGACATCAATTTTGCATTGAGAACCTGCAGAAACTCATCCTATCAGAAACATCTATTTTTGAT
  5 gallus_ACACA     89.0%
  CTGCTATTGACATGTATGGACACCAGTTCTGCATTGAGAACCTGCAGAAACTCATTTTGTCAGAGACATCCATCTTTGAT
  6 alligator_ACACA  84.0%
  CAGCCATTGACATGTACGGACACCAGTTCTGCATTGAGAACCTGCAGAAACTCATTCTGTCTGAGACATCCATCTTTGAT

                              3521            .           .           :           .           .           .
  .         6 3600
  1 ruby_ACACA      100.0%
  GTGCTACCCAACTTTTTCTACCACAGTAACCAGGTGGTAAGAATGGCAGCTTTGGAGGTGTATGTGAGGAGAGCCTACAT
  2 canna_ACACA      98.5%
  GTGCTACCCAACTTTTTCTACCACAGTAACCAGGTGGTAAGAATGGCAGCTTTGGAGGTGTATGTGAGGAGAGCCTACAT
  3 swift_ACACA      92.4%
  GTGCTACCCAACTTTTTCTACCACAGTAACCAGGTGGTAAGAATGGCAGCTCTGGAGGTGTATGTTCGAAGGGCATATAT
  4 human_ACACA      77.6%
  GTCCTACCAAACTTCTTCTATCACAGCAACCAAGTAGTGAGGATGGCAGCTCTGGAGGTGTATGTTCGAAGGGCTTATAT
  5 gallus_ACACA     89.0%
  GTGCTACCCAACTTTTTCTACCACAGTAATCAGGTGGTGAGAATGGCAGCTTTGGAGGTGTACGTTCGAAGGGCGTACAT
  6 alligator_ACACA  84.0%
  GTGCTACCAAATTTCTTCTATCACAGTAACCAGGTGGTGAGAATGGCAGCTCTGGAGGTGTATGTTCGAAGAGCATACAT

                              3601            .           .           .           .           :           .
  .          . 3680
  1 ruby_ACACA      100.0%
  TGCCTACGAGTTAAACAGCGTCCAGCACCGCCAGCTGAAGGACAACACCTGCGTGGTGGAGTTCCAGTTCATGCTGCCCA
  2 canna_ACACA      98.5%
  TGCCTACGAGTTAAACAGCGTCCAGCACCGCCAGCTGAAGGACAACACCTGCGTGGTGGAGTTCCAGTTCATGCTGCCCA
  3 swift_ACACA      92.4%
  TGCCTATGAGCTGAACAGTGTCCAGCATCGCCAGCTGAAGGACAACACCTGTGTGGTGGAGTTCCAGTTCATGCTCCCTA
  4 human_ACACA      77.6%
  TGCCTATGAACTTAACAGCGTACAACACCGCCAGCTTAAGGACAACACCTGTGTGGTGGAATTCCAGTTCATGCTGCCCA
  5 gallus_ACACA     89.0%
  TGCCTACGAGCTGAACAGCGTCCAGCACCGCCAGCTGAAGGACAACACCTGCGTGGTGGAGTTCCAGTTCATGCTGCCCA
  6 alligator_ACACA  84.0%
  TGCCTATGAGTTGAACAGTGTCCAGCATCGGCAGCTAAAGGACAACACCTGTGTGGTGGAGTTCCAGTTCATGTTACCCA

                              3681            .           7           .           .           .           .
  :          . 3760
  1 ruby_ACACA      100.0%      CCTCCCACCCTAACAGA-----------------------
  -ATGTCCTTCTCTTCCAACCTCAATCACTACGGGATGGTC
  2 canna_ACACA      98.5%
  CCTCCCACCCTAACAGAGGGAACATCCCCACGCTAAACAGAATGTCCTTCTCTTCCAACCTCAATCACTACGGGATGGTC
```

```
3 swift_ACACA      92.4%
CCTCCCATCCAAACAGAGGGAACATCCCCACGCTAAACAGAATGTCCTTCTCTTCCAACCTCAATCACTATGGGATGGTC
4 human_ACACA      77.6%
CATCTCATCCAAACAGAGGGAACATCCCTACGCTAAACAGAATGTCCTTCTCCTCCAACCTCAACCACTATGGCATGACC
5 gallus_ACACA     89.0%      CCTCCCACCCAAACAGA-----------------------
-ATGTCCTTCTCTTCCAACCTCAATCACTACGGGATGGTC
6 alligator_ACACA  84.0%      CCTCCCATCCAAACAGA-----------------------
-ATGTCCTTCTCTTCCAACCTCAACCACTACGGGATGGTC

                         3761          .         .         .         8         .         .
.         . 3840
1 ruby_ACACA      100.0%
CATGTAGCAAGTGTCAGTGATGTGCTGCTGGACAACTCCTTCACTCCCCCGTGCCAGCGGATGGGGGGCATGGTCTCCTT
2 canna_ACACA      98.5%
CATGTAGCAAGTGTCAGTGATGTGCTGCTGGACAACTCCTTCACTCCCCCGTGCCAGCGGATGGGGGGCATGGTCTCCTT
3 swift_ACACA      92.4%
CATGTAGCTAGTGTGAGTGATGTACTGCTGGACAATTCCTTCACCCCCCCGTGCCAGCGGATGGGCGGGATGGTTTCCTT
4 human_ACACA      77.6%
CATGTAGCTAGTGTCAGCGATGTACTGTTGGACAACTCATTCACTCCACCTTGTCAGCGGATGGGCGGAATGGTCTCTTT
5 gallus_ACACA     89.0%
CACGTAGCCAGTGTGAGTGACGTGCTGCTGGACAACTCGTTCACTCCCCCGTGCCAGCGGATGGGAGGGATGGTCTCTTT
6 alligator_ACACA  84.0%
CACGTGGCCAGCGTGAGTGACGTGCTGCTGGACAACTCATTCACCCCGCCCTGCCAGCGGATGGGCGGGATGGTCTCCTT

                         3841          :         .         .         .         .         9
.         . 3920
1 ruby_ACACA      100.0%
CCGCACCTTTGAAGACTTTGTCAGAATCTTTGATGAAGTGATGGGTTGCTTCTGTGACTCTCCTCCCCAGAGCCCAACCT
2 canna_ACACA      98.5%
CCGCACCTTTGAAGACTTTGTCAGAATCTTTGATGAAGTGATGGGTTGCTTCTGTGACTCTCCTCCCCAGAGCCCAACCT
3 swift_ACACA      92.4%
CCGCACCTTTGAAGATTTTGTCAGGATCTTTGATGAAGTGATGGGTTGCTTCTGTGACTCTCCCCCCCAGAGCCCAACCT
4 human_ACACA      77.6%
TCGGACTTTTGAAGATTTTGTCAGGATCTTTGATGAAGTGATGGGCTGCTTCTCTGACTCCCCACCCCAGAGTCCCACAT
5 gallus_ACACA     89.0%
CCGCACGTTTGAAGATTTTGTCAGAATCTTTGATGAAGTGATGAGTTGTTTTTGCGACTCTCCTCCCCAGAGCCCAACCT
6 alligator_ACACA  84.0%
CAGGACATTTGACGATTTTGTCAGAATCTTCGATGAAGTGATGGGTTGCTTCTGTGACTCTCCTCCCCAAAGCCCGACCT

                         3921          .         .         :         .         .         .
.         0 4000
1 ruby_ACACA      100.0%
TCCCTGAAGCTGGCCATGCTTCCTTGTATGATGAAGACAAGACTTCCCGTGAGGAACCCATTCACATTCTTAATGTTGCT
2 canna_ACACA      98.5%
TCCCTGAAGCTGGCCATGCTTCCTTGTATGATGAAGACAAGACCTCCCGTGAGGAACCCATTCACATTCTTAATGTTGCT
3 swift_ACACA      92.4%
TCCCTGAAGCTGGCCATGCTTCCTTGTATGATGAAGACAAGGCTCCCCGTGAGGAGCCCATCCACATTCTTAATGTTGCT
4 human_ACACA      77.6%
TCCCTGAGGCAGGTCACACGTCTCTTTATGATGAGGATAAGGTTCCCAGGGATGAACCAATTCACATTCTCAATGTGGCT
5 gallus_ACACA     89.0%
TCCCTGAAGCTGGCCATGCTTCCCTCTATGATGAAGACAAGGCTGCCCGTGAGGAGCCCATTCACATTCTTAATGTCGCT
6 alligator_ACACA  84.0%
TCCGTGAAGCCAGCCGCACTTCTTTGTATGATGAAGATAAGGTTGCCAGAGAGGAGCCTATTCACATCTTGAATGTTGCC

                         4001          .         .         .         .         :         .
.         . 4080
1 ruby_ACACA      100.0%
ATTAAAACTGACAGTGATGTTGATGATGATGGGCTGGCAGCCATGTTCAGGGAGTTCACACAAAGCAAGAAATCAGTCCT
2 canna_ACACA      98.5%
ATTAAAACTGACAGTGATGTTGATGATGATGGGCTGGCAGCCATGTTCAGGGAGTTCACACAAAGCAAGAAATCAGTCCT
3 swift_ACACA      92.4%
ATTAAAACTGACAGCGACGTTGATGACGATGGGCTGGCAGCCATGTTCAGGGAGTTCACACAAAGCAAGAAATCAGTCCT
4 human_ACACA      77.6%
ATCAAGACTGACTGTGATATTGAGGATGACAGGCTGGCAGCTATGTTCAGAGAATTTACCCAGCAAAATAAAGCTACCCT
5 gallus_ACACA     89.0%
ATTAAAACTGATGGGGACGTGGATGATGATGGGCTGGCAGCCATGTTCAGAGAGTTCACACAAAGCAAGAAATCAGTCCT
6 alligator_ACACA  84.0%
ATTAAGACCGACAGCGATATTGATGACGATGGGCTGGCAGCCATGTTCAGGGAGTTCACACAAAGCAAGAAATCCATCCT

                         4081          .         1         .         .         .         .
:         . 4160
1 ruby_ACACA      100.0%      GTTTGAACACGGGATTCGGAGGCTGACTTTCCTTGTAGCACAAAAG----------------------
------------
2 canna_ACACA      98.5%
GTTTGAACACGGGATTCGGAGGCTGACTTTCCTTGTAGCACAAAAGGACTTCAGGAAACAGGTCAACTATGAGGTGGATC
3 swift_ACACA      92.4%
GATCGAACATGGGATTCGGAGGCTGACTTTCCTTGTAGCACAAAAGGACTTCAGGAAACAGGTCAACTATGAGGTGGATC
4 human_ACACA      77.6%
GGTTGACCATGGGATCCGGCGCCTTACTTTCCTGGTTGCACAAAAGGATTTCAGAAAGCAGGTCAACTATGAGGTGGATC
5 gallus_ACACA     89.0%      GATTGAGCATGGCATCCGGAGGCTGACATTCCTTGTGGCACAGAAG----------------------
------------
```

```
6 alligator_ACACA  84.0%
GATTGAGCATGGGATCCGGAGGCTGACATTCCTTGTAGCACAAAAGGATTTCAGGAAACAGGTCAACTATGAGGTGGATC

                         4161          .         .         .         2         .         .
.         . 4240
1 ruby_ACACA      100.0%      ----------
-AGAGAATTCCCAAAGTTCTTCACGTTCCGTGCCCGGGATAAGTTTGAGGAGGACAGGATTTACAGGCAC
2 canna_ACACA      98.5%
AGAGATTTCATAGAGAATTCCCAAAGTTCTTCACGTTCCGTGCTCGGGATAAGTTTGAGGAGGACAGGATTTACAGGCAC
3 swift_ACACA      92.4%
AGAGATTCCATAGGGAGTTTCCAAAGTTCTTCACATTCCGTGCCCGGGATAAGTTTGAGGAAGACAGGATTTACCGTCAC
4 human_ACACA      77.6%
GGAGATTTCATAGAGAATTCCCTAAATTTTTTACATTCCGAGCAAGGGATAAGTTTGAGGAGGATCGTATCTATCGTCAT
5 gallus_ACACA     89.0%      ----------
-AGGGAATTTCCAAAGTTCTTCACGTTCCGTGCCAGGGATAAGTTTGAAGAAGACAGAATCTACCGCCAT
6 alligator_ACACA  84.0%
AAAGATTTCATAGGGAATTCCCCAAATTTTTCACATTCCGCGCCAGAGACAAGTTTGAGGAAGATAGGATCTACCGTCAC

                         4241          :         .         .         .         .         3
.         . 4320
1 ruby_ACACA      100.0%
CTGGAGCCAGCTCTGGCTTTTCAGCTGGAGCTGAACCGCATGAGGAACTTCGATCTCACTGCCATCCCCTGTGCCAATCA
2 canna_ACACA      98.5%
CTGGAGCCAGCTCTGGCTTTTCAGCTGGAGCTGAAANNNNNNNNNNNNTTCCCTCTCACTGCCATCCCCTGTGCCAATCA
3 swift_ACACA      92.4%
CTGGAGCCAGCTCTGGCTTTCCAGTTGGAGTTGAACCGAATGCGCAACTTTGATCTCACTGCCATTCCATGTGCCAACCA
4 human_ACACA      77.6%
CTGGAGCCTGCTCTGGCTTTCCAGTTAGAGCTGAACCGGATGAGAAATTTTGACCTCACTGCCATTCCATGTGCTAATCA
5 gallus_ACACA     89.0%
CTGGAGCCAGCTCTGGCTTTCCAGCTGGAGCTGAACCGAATGCGGAACTTTGACCTCACTGCCATTCCATGTGCCAACCA
6 alligator_ACACA  84.0%
TTGGAGCCAGCTCTGGCTTTCCAGTTAGAGCTGAACCGAATGAGAAACTTTGATCTCACTGCCATTCCGTGCGCCAATCA

                         4321          .         .         :         .         .         .
.        4 4400
1 ruby_ACACA      100.0%
CAAAATGCATCTCTACCTGGGAGCAGCTAAAGTTGAAGTGGGAACAGAAGTGACAGACTACAGGTTCTTCGTGAGGGCCA
2 canna_ACACA      98.5%
CAAAATGCATCTCTACCTGGGAGCAGCTAAAGTTGAAGTGGGAACAGAAGTGACAGACTACAGGTTCTTCGTGAGGGCCA
3 swift_ACACA      92.4%
CAAAATGCATCTCTACCTGGGAGCTGCTAAAGTTGAAGTGGGAACAGAAGTGACAGACTACAGGTTCTTCGTGAGGGCTA
4 human_ACACA      77.6%
CAAGATGCACCTGTATCTCGGGGCAGCCAAGGTGGAAGTGGGCACAGAAGTGACAGACTACAGGTTCTTTGTTCGTGCAA
5 gallus_ACACA     89.0%
TAAAATGCATCTCTACCTGGGAGCAGCTAAAGTTGAAGTAGGAACAGAAGTGACAGACTACAGGTTCTTTGTGAGGGCCA
6 alligator_ACACA  84.0%
TAAGATGCACCTGTACCTGGGAGCAGCCAAAGTCGAAGTGGGCACGGAGGTGACGGACTACAGGTTCTTCGTGAGGGCTA

                         4401          .         .         .         .         :         .
.         . 4480
1 ruby_ACACA      100.0%
TTATAAGGCATTCAGACCTGGTCACCAAGGAAGCCTCCTTTGAGTACCTGCAAAATGAGGGAGAACGATTGCTTTTGGAA
2 canna_ACACA      98.5%
TTATAAGGCATTCAGACCTGGTCACCAAGGAAGCCTCCTTTGAGTACCTGCAAAATGAGGGAGAACGATTGCTTTTGGAA
3 swift_ACACA      92.4%
TCATAAGGCATTCAGACCTGGTTACCAAGGAAGCCTCCTTTGAGTATCTGCAAAATGAGGGAGAGCGATTGCTTTTGGAA
4 human_ACACA      77.6%
TCATCAGGCATTCTGATCTGGTCACCAAGGAAGCTTCTTTTGAATATCTGCAAAATGAAGGGGAGCGGCTACTCCTGGAA
5 gallus_ACACA     89.0%
TTATAAGGCATTCAGACCTTGTTACCAAGGAAGCCTCCTTCGAGTACCTGCAAAACGAGGGAGAGCGATTGCTTTTGGAA
6 alligator_ACACA  84.0%
TTATAAGGCATTCAGACTTAGTCACCAAGGAAGCCTCCTTTGAGTATCTTCAGAACGAAGGGGAGCGCTTGCTTTTGGAA

                         4481          .         5         .         .         .         .
:         . 4560
1 ruby_ACACA      100.0%
GCCATGGATGAGCTGGAGGTGGCGTTTAACAACACCAACGTGCGCACAGACTGCAATCACATCTTCTTAAATTTCGTGCC
2 canna_ACACA      98.5%
GCCATGGATGAGCTGGAGGTGGCATTTAACAACACCAACGTGCGCACAGACTGCAATCACATCTTCTTAAATTTCGTGCC
3 swift_ACACA      92.4%
GCCATGGATGAGTTGGAGGTGGCGTTTAATAACACCAACGTGCGCACCGACTGCAATCACATCTTCTTAAATTTCGTGCC
4 human_ACACA      77.6%
GCCATGGATGAGTTGGAAGTTGCTTTTAACAATACAAATGTCCGCACTGACTGTAACCACATCTTCCTCAACTTTGTGCC
5 gallus_ACACA     89.0%
GCCATGGATGAGTTGGAGGTGGCATTTAATAATACCAACGTGCGCACGGACTGCAATCACATCTTCTTAAATTTTGTGCC
6 alligator_ACACA  84.0%
GCCATGGATGAGCTGGAGGTGGCATTTAATAACACCAACGTGCGCACGGACTGCAATCACATCTTCTTAAACTTTGTGCC

                         4561          .         .         .         6         .         .
.         . 4640
1 ruby_ACACA      100.0%
```

```
TACTGTCATCATGGACCCATCAAAGATCGAGGAGTCGGTGAGGAGCATGGTGATGCGCTACGGGAGCCGGCTCTGGAAAC
2 canna_ACACA      98.5%
TACTGTCATCATGGACCCATCGAAGATCGAGGAGTCGGTGAGGAGCATGGTGATGCGCTACGGGAGCCGGCTCTGGAAAC
3 swift_ACACA      92.4%
TACTGTCATCATGGACCCATCTAAGATCGAGGAGTCGGTGCGGAGCATGGTGATGCGCTACGGGAGCCGCCTGTGGAAGC
4 human_ACACA      77.6%
CACGGTTATCATGGACCCATCAAAGATTGAGGAATCCGTGCGGAGCATGGTAATGCGGTATGGAAGTCGCCTGTGGAAAT
5 gallus_ACACA     89.0%
TACTGTCATCATGGACCCATCCAAGATCGAGGAATCCGTGCGGAGCATGGTGATGCGCTACGGGAGCCGCCTGTGGAAGC
6 alligator_ACACA  84.0%
CACTGTCATCATGGATCCATCTAAGATTGAAGAGTCTGTGCGGAGCATGGTGATGCGCTATGGAAGCCGCCTCTGGAAAC

                         4641          :         .         .         .         .         7
.         . 4720
1 ruby_ACACA      100.0%
TCCGCGTCCTCCAGGCAGAGCTGAAGATCAACATTCGGCTGACACCCACAGGGAAGGCAATTCCCATCAGGCTCTTCCTG
2 canna_ACACA      98.5%
TCCGCGTCCTCCAGGCAGAGCTGAAGATCAACATTCGGCTGACACCCACAGGGAAGGCAATTCCCATCAGGCTCTTCCTG
3 swift_ACACA      92.4%
TCCGCGTCCTCCAGGCCGAGCTGAAGATCAACATCCGCCTGACTCCAACTGGGAAGGCCATTCCCATCCGTCTCTTCTTG
4 human_ACACA      77.6%
TGCGCGTCCTCCAGGCAGAACTGAAAATCAACATTCGCCTGACGCCAACTGGAAAAGCAATTCCCATCCGCCTCTTCCTG
5 gallus_ACACA     89.0%
TCCGCGTCCTCCAGGCCGAGCTGAAGATCAACATTCGGCTGACACCGACAGGAAAGGCCATCCCCATTCGTCTCTTCCTG
6 alligator_ACACA  84.0%
TCCGCGTCCTTCAAGCAGAGCTGAAAATCAACATTCGCTTGACGCCAACTGGAAAAGCGATACCTATTCGTCTCTTCCTG

                         4721          .         .         :         .         .         .
.         8 4800
1 ruby_ACACA      100.0%
ACCAATGAGTCAGGCTACTACTTGGACATCAGCCTGTACAAAGAGGTGACAGACTCCAGGACAGGACAGATTATGTTCCA
2 canna_ACACA      98.5%
ACCAATGAGTCAGGCTACTACTTGGACATCAGCCTGTACAAAGAGGTGACAGACTCCAGGACAGGACAGATTATGTTCCA
3 swift_ACACA      92.4%
ACCAACGAGTCAGGCTACTACTTGGACATCAGCTTGTACAAAGAAGTGACAGACTCCAGGACAGGGCAGATTATGTTCCA
4 human_ACACA      77.6%
ACAAACGAGTCTGGCTATTACTTGGATATCAGCCTATACAAGGAAGTGACTGACTCCAGGACAGCACAGATCATGTTTCA
5 gallus_ACACA     89.0%
ACCAACGAGTCGGGCTACTACCTGGACATCAGCCTGTACAAAGAGGTGACGGATTCCAGGACAGGGCAGATTATGTTCCA
6 alligator_ACACA  84.0%
ACCAATGAATCTGGCTACTACTTGGACATCAGCCTCTACAAGGAGGTGACAGATTCCAGGACAGCCCAGATTATGTTCCA

                         4801          .         .         .         .         :         .
.         . 4880
1 ruby_ACACA      100.0%
GGCATACGGTGATAAACAGGGACCACTCCATGGGATGCTGATCAATACCCCCTACGTGACCAAAGACCTGCTTCAGTCCA
2 canna_ACACA      98.5%
GGCATACGGTGATAAACAGGGACCACTCCATGGGATGCTGATCAATACCCCCTACGTGACCAAAGACCTGCTTCAGTCCA
3 swift_ACACA      92.4%
GGCATACGGGGATAAACAGGGACCACTTCATGGGATGCTGATCAATACCCCATATGTGACCAAAGACCTACTTCAGTCCA
4 human_ACACA      77.6%
GGCATATGGAGACAAACAGGGACCACTGCATGGAATGTTAATCAATACTCCATATGTGACCAAAGACCTGCTGCAATCAA
5 gallus_ACACA     89.0%
GGCCTATGGGGATAAACAGGGACCACTTCACGGGATGCTGATAAATACCCCATACGTGACCAAGGACCTTCTTCAGTCCA
6 alligator_ACACA  84.0%
GGCGTATGGAGACAAACAGGGACCACTTCATGGCATGCTGATCAACACCCCATACGTGACAAAAGACCTCCTTCAGTCCA

                         4881          .         9         .         .         .         .
:         . 4960
1 ruby_ACACA      100.0%
AGAGGTTCCAGGCACAGTCTTTAGGGACATCATATGTCTATGACATTCCTGAGATGTTTAGGCAGTCTTTGATTAAACTC
2 canna_ACACA      98.5%
AGAGGTTCCAGGCACAGTCTTTAGGGACATCATATGTCTATGACATTCCTGAGATGTTTAGGCAGTCTTTGATTAAACTC
3 swift_ACACA      92.4%
AGAGATTCCAGGCACAGTCTTTGGGGACATCATATGTCTATGACATTCCTGAGATGTTTCGGCAATCTTTGATTAAACTC
4 human_ACACA      77.6%
AGAGGTTCCAGGCACAATCCTTAGGGACAACATACATATATGATATCCCAGAGATGTTTCGGCAGTCCCTGATCAAACTC
5 gallus_ACACA     89.0%
AGAGATTCCAGGCACAGTCTTTAGGGACATCCTATGTCTATGACATTCCTGAGATGTTTCGGCAGTCTTTAATTAAACTC
6 alligator_ACACA  84.0%
AGAGGTTCCAGGCACAGTCTTTAGGGACAACATATGTCTATGACATTCCTGAAATGTTTCGGCAGTCACTGATTAAACTC

                         4961          .         .         .         0         .         .
.         . 5040
1 ruby_ACACA      100.0%
TGGGGACATATGAACGACCACGCGTTCCTGCCGACACCGCCGCTGCCGTCTGACATCCTGACGTACACTGAGCTGGTGCT
2 canna_ACACA      98.5%
TGGGGACATATGAACGACCACGCGTTCCTGCCGACACCGCCGCTGCCGTCTGACATCCTGACGTACACTGAGCTGGTGCT
3 swift_ACACA      92.4%
TGGGAGTCTATGAATGAACATGCATTCCTGCCAACACCACCGCTGCCTTCCGACATCCTAACATACACTGAATTGGTGCT
4 human_ACACA      77.6%
```

```
TGGGAGTCTATGTCCACTCAAGCATTTCTTCCATCTCCCCCTCTGCCTTCTGACATGCTGACTTACACTGAACTGGTACT
5 gallus_ACACA     89.0%
TGGGATTCTATGAATGAACATGCATTCCTGCCAACACCGCCGCTGCCGTCTGACATACTGACATACACTGAATTGGTGCT
6 alligator_ACACA  84.0%
TGGGATTCCATGAATGAATGTGCAGTTCTGTCTGCACCGCCTCTGCCTTCTGACATGCTGACTTATACTGAACTGGTCCT

                         5041          :         .         .         .         .         1
.         . 5120
1 ruby_ACACA      100.0%
GGATGATCAGGGCCAACTCGTGCACATGAACAGGCTGCCAGGAGGAAATGAGGTTGGCATGGTGGCCTGGAAGATGACTC
2 canna_ACACA      98.5%
GGATGATCAGGGCCAACTCGTGCACATGAACAGGCTGCCAGGAGGAAATGAGGTTGGCATGGTGGCCTGGAAGATGACTC
3 swift_ACACA      92.4%
GGATGATCAGGGCCAACTGGTGCACATGAACAGGCTGCCAGGAGGAAATGAGATTGGGATGGTGGCCTGGAAGATGACTC
4 human_ACACA      77.6%
GGATGATCAAGGTCAGCTGGTCCACATGAACAGGCTTCCAGGAGGAAATGAGATTGGCATGGTAGCTTGGAAAATGACCT
5 gallus_ACACA     89.0%
GGATGATCAGGGCCAGCTCGTGCACATGAACAGGCTGCCAGGAGGAAACGAGATTGGGATGGTAGCCTGGAAAATGACCC
6 alligator_ACACA  84.0%
GGATGATCAAGGCCAGCTGGTGCACATGAACAGGCTACCTGGTGGAAATGAGATTGGCATGGTGGCCTGGAAAATGACCC

                         5121          .         .         :         .         .         .
.         2 5200
1 ruby_ACACA      100.0%
TCAAGACCCCTGAGTATCCTGAAGGCCGTGATATCATTGTCATTGGCAATGACATTACATACAAAATAGGTTCTTTTGGG
2 canna_ACACA      98.5%
TCAAGACCCCTGAGTATCCTGAAGGCCGTGATATCATTGTCATTGGCAATGACATTACATACAAAATAGGTTCTTTTGGG
3 swift_ACACA      92.4%
TCAAGACCCCTGAGTATCCAGAAGGCCGTGATATCATTGTCATTGGCAATGACATCACGTACCGGATAGGTTCTTTTGGG
4 human_ACACA      77.6%
TTAAAAGTCCTGAATATCCAGAAGGCCGAGATATCATTGTTATTGGCAATGACATCACATACCGAATTGGGTCCTTTGGG
5 gallus_ACACA     89.0%
TCAAGACCCCGGAGTATCCCGAAGGCCGTGATATCATCGTCATTGGCAATGACATTACGTACCGGATAGGTTCTTTTGGG
6 alligator_ACACA  84.0%
TCAAGAGCCCCGAGTACCCAGAAGGCCGTGATATCATAGTCATTGGGAATGATATCACGTACCGAATTGGCTCCTTTGGA

                         5201          .         .         .         .         :         .
.         . 5280
1 ruby_ACACA      100.0%
CCTCAGGAGGACATCCTGTTCCTGAGGGCTTCAGAGCTTGCTCGAACACACGGCATCCCCCGGATCTATGTGGCTGCCAA
2 canna_ACACA      98.5%
CCTCAGGAGGACATCCTGTTCCTGAGGGCTTCAGAGCTTGCTCGAACACATGGCATCCCCCGGATCTATGTGGCTGCCAA
3 swift_ACACA      92.4%
CCCCAGGAGGATGTGCTGTACCTGAGGGCTTCAGAGCTTGCTCGAACACAAGGCATCCCCCGTGTCTATGTGGCTGCCAA
4 human_ACACA      77.6%
CCTCAAGAGGATTTGTTATTTCTCAGAGCTTCCGAACTTGCTAGGGCAGAAGGTATTCCACGCATCTATGTATCAGCCAA
5 gallus_ACACA     89.0%
CCTCAGGAGGACGTGCTGTTCCTGAGGGCTTCAGAGCTTGCTCGAACTCATGGCATCCCCCGCATCTACGTGGCTGCCAA
6 alligator_ACACA  84.0%
CCACAGGAGGACCTGCTCTATCTTCGGGCCTCTGAACTTGCTCGAACTCAGGGCATCCCCCGGATTTACGTGGCTGCCAA

                         5281          .         3         .         .         .         .
:         . 5360
1 ruby_ACACA      100.0%
CAGTGGAGCCAGGATTGGTTTGGCTGAGGAGATTCGGCACATGTTCCATGTAGCTTGGGAAGACCCAGAGGATCCATACA
2 canna_ACACA      98.5%
CAGTGGAGCCAGGATTGGTTTGGCTGAGGAGATTCGGCACATGTTCCATGTAGCTTGGGAAGACCCAGAGGATCCATACA
3 swift_ACACA      92.4%
CAGCGGAGCCAGGATTGGTTTGGCTGAGGAGATTCGGCACATGTTCCATGTAGCTTGGGAAGACCCAGATGACCCATACA
4 human_ACACA      77.6%
CAGTGGAGCAAGAATCGGACTGGCAGAAGAAATTCGCCATATGTTTCATGTGGCCTGGGTAGATCCTGAGGATCCTTACA
5 gallus_ACACA     89.0%
CAGCGGAGCCAGGATTGGTTTGGCTGAGGAGATCCGGCACATGTTCCATGTTGCGTGGGAAGATCCAGATGACCCATACA
6 alligator_ACACA  84.0%
CAGTGGAGCGAGGATTGGTTTGGCTGAGGAGATTCGTCATATGTTTCACGTGGCCTGGGTGGAGCCCGATGATCCGTATA

                         5361          .         .         .         4         .         .
.         . 5440
1 ruby_ACACA      100.0%
AGGGATACAAATACTTGTACCTGACTCCTCAAGACTACAAGAAAGTCAGTGCCCTGAACTCTGTTCACTGTGAACACGTG
2 canna_ACACA      98.5%
AGGGATACAAATACTTGTACCTGACTCCTCAAGACTACAAGAAAGTCAGTGCCCTGAACTCTGTTCACTGTGAACACGTG
3 swift_ACACA      92.4%
AGGGATACAAATACTTGTACCTGACTCCTCAAGACTATAAGAAAGTCAGTGCCCTCAACTCAGTTCACTGTGAGCACGTG
4 human_ACACA      77.6%
AGGGATACAGGTATTTATATCTGACTCCTCAAGATTATAAGAGAGTCAGTGCTCTCAACTCTGTCCATTGTGAACACGTG
5 gallus_ACACA     89.0%
AAGGATACAAGTACTTGTATCTGACACCTCAAGACTATAAGAAAGTCAGCGCTCTGAACTCAGTTCACTGTGAACACGTG
6 alligator_ACACA  84.0%
AAGGATACAAATACTTATATCTGACACCTCAAGATTATAAGAAGGTCAGTGCCTTGAACTCAGTCCATTGCGAGCACGTG
```

```
                           5441         :         .         .         .         .         5
   .         . 5520
1 ruby_ACACA      100.0%
GAGGACAGTGGTGAGTCCAGGTATAAGATAACAGATATTATTGGAAAGGAAGATGGACTTGGAATAGAGAACCTCAGAGG
2 canna_ACACA      98.5%
GAGGACAGTGGTGAGTCCAGGTATAAGATAACAGATATTATTGGAAAGGAAGATGGACTTGGAATAGAGAACCTCAGAGG
3 swift_ACACA      92.4%
GAAGAAAATGGAGAGTCCAGGTATAAGATAACAGATATTATCGGAAAGGAAGATGGACTTGGAATAGAGAACCTCAGAGG
4 human_ACACA      77.6%
GAAGATGAAGGAGAATCCAGGTACAAGATAACAGATATTATTGGGAAAGAAGAGGGAATTGGACCCGAGAACCTTCGAGG
5 gallus_ACACA     89.0%
GAGGACAACGGAGAGTCCAGGTATAAGATAACAGATATTATCGGAAAGGAAGACGGACTTGGAATAGAGAACCTCAGAGG
6 alligator_ACACA  84.0%
GAAGACGAAGGAGAGTCCAGGTATAAGATAACAGATATTATTGGAAAGGAAGAAGGACTTGGAGTAGAAAACCTCAGAGG

                           5521         .         .         :         .         .         .
   .         6 5600
1 ruby_ACACA      100.0%
ATCTGGCATGATTGCTGGAGAAACATCTTTAGCCTATGACAGTATTATCACCATCAACTTGGTTACATGTCGGGCAATTG
2 canna_ACACA      98.5%
ATCTGGCATGATTGCTGGAGAAACATCTTTAGCCTATGACAGTATTATCACCATCAACTTGGTTACATGTCGGGCAATTG
3 swift_ACACA      92.4%
ATCTGGCATGATTGCTGGAGAAACGTCTTTAGCCTATGATAGTATTATCACCATCAACTTGGTTACATGTCGGGCAATTG
4 human_ACACA      77.6%
TTCTGGAATGATTGCTGGAGAATCCTCATTGGCCTATAATGAGATCATTACCATCAGCCTGGTGACGTGCCGGGCCATTG
5 gallus_ACACA     89.0%
ATCTGGCATGATTGCTGGAGAATCATCTTTAGCCTACGAGAGTATTATCACCATCAACTTGGTTACGTGTCGGGCAATTG
6 alligator_ACACA  84.0%
GTCTGGCATGATTGCTGGAGAGTCATCATTAGCCTATGACAGTATTATCACCATCAGCCTGGTCACGTGTCGGGCAATTG

                           5601         .         .         .         .         :         .
   .         . 5680
1 ruby_ACACA      100.0%
GGATTGGGGCTTACCTGGTTCGACTGGGGCAGAGGACTCTCCAGGTTGAGAACTCCCATATAATCCTGACTGGCTGTGGA
2 canna_ACACA      98.5%
GGATTGGGGCTTACCTGGTTCGACTGGGGCAGAGGACTCTCCAGGTTGAGAACTCCCATATAATCCTGACTGGCTGTGGA
3 swift_ACACA      92.4%
GAATTGGGGCTTACCTGGTTCGGCTGGGGCAGAGGACTATCCAGGTTGAGAACTCTCATATAATCCTGACTGGCTGTGGA
4 human_ACACA      77.6%
GGATTGGGGCTTACCTTGTCCGGCTGGGACAGAGAACCATCCAGGTTGAGAATTCTCACTTAATTCTAACAGGAGCTGGA
5 gallus_ACACA     89.0%
GAATTGGAGCTTACCTCGTTCGGTTAGGGCAGAGGACTATCCAGGTTGAGAACTCTCACATAATCCTGACTGGCTGTGGA
6 alligator_ACACA  84.0%
GCATTGGGGCTTACCTTGTTCGGCTTGGACAGAGGACCATCCAAGTGGAGAACTCCCATATAATCCTGACCGGAGCCGGA

                           5681         .         7         .         .         .         .
   :         . 5760
1 ruby_ACACA      100.0%
GCCCTCAACAAAGTGCTTGGGAGGGAAGTTTACACCTCCAACAACCAGCTGGGCGGGATCCAGATCATGCACAACAATGG
2 canna_ACACA      98.5%
GCCCTCAACAAAGTGCTTGGGAGGGAAGTTTACACCTCCAACAACCAGCTGGGCGGGATCCAGATCATGCACAACAATGG
3 swift_ACACA      92.4%
GCCCTCAACAAAGTGCTTGGGCGGGAAGTTTATACCTCCAACAACCAGCTGGGTGGGATCCAGATAATGCACAACAACGG
4 human_ACACA      77.6%
GCCCTCAACAAAGTCCTCGGGCGGGAAGTGTACACCTCCAATAACCAGCTGGGGGGCATCCAGATTATGCACAACAATGG
5 gallus_ACACA     89.0%
GCCCTCAACAAGGTGCTGGGACGGGAGGTGTACACCTCCAACAACCAGCTGGGCGGGATCCAGATCATGCACAACAACGG
6 alligator_ACACA  84.0%
GCACTGAACAAGGTGCTTGGACGGGAGGTGTACACCTCAAACAACCAGCTGGGTGGGATCCAGATCATGCACAACAATGG

                           5761         .         .         .         8         .         .
   .         . 5840
1 ruby_ACACA      100.0%
AGTGACACACAACACCGTGTGTGATGATTTTGAAGGAGTCTACACTGTTCTGCAGTGGCTTTCCTACATGCCAAAGAGTG
2 canna_ACACA      98.5%
AGTGACACACAACACCGTGTGTGATGATTTTGAAGGAGTCTACACTGTTCTGCAGTGGCTTTCCTACATGCCAAAGAGTG
3 swift_ACACA      92.4%
GGTGACACACAGCACAGTGTGTGATGATTTTGAAGGAGTCTACACTGTTCTGCAGTGGATTTCCTACATGCCAAAGAATG
4 human_ACACA      77.6%
GGTGACCCACTGCACTGTGTGTGATGACTTTGAAGGGGTTTTCACTGTCCTGCACTGGCTGTCTTACATGCCCAAGAGCG
5 gallus_ACACA     89.0%
GGTGACCCACGGCACCGTGTGCGACGATTTTGAAGGAGTCTACACTATCCTGCTGTGGCTTTCCTACATGCCCAAGAGCG
6 alligator_ACACA  84.0%
AGTGACACACAGCACCGTGTGCGATGATTTTGAAGGTGTCTTCACCATTCTGCTGTGGTTATCCTACATGCCAAAGAGTG

                           5841         :         .         .         .         .         9
   .         . 5920
1 ruby_ACACA      100.0%
TATCCAGCCCTGTTCCTATCCTCAAAGTCAAGGATCCTATAGACAGAACCATAGACTTTGTTCCCACCAAGGCTCCCTAT
2 canna_ACACA      98.5%
TATCCAGCCCTGTTCCTATCCTCAAAGTCAAGGATCCTATAGACAGAACCATAGACTTTGTTCCCACCAAGGCTCCCTAT
```

```
  3 swift_ACACA       92.4%
  TATCCAGCCCTGTTCCTATCCTCAAAGTCAAGGATCCTATAGACAGAACCATAGACTTTGTTCCTACCAAGACTCCCTAT
  4 human_ACACA       77.6%
  TGCACAGTTCAGTTCCTCTTCTGAACTCAAAGGATCCTATAGACAGAATCATCGAGTTTGTTCCCACAAAGACCCCATAC
  5 gallus_ACACA      89.0%
  TATACAGCCCTGTTCCTATCCTCAAGGTCAAGGATCCTATAGACAGAACCATAGACTTCGTTCCTACCAAGACTCCCTAT
  6 alligator_ACACA   84.0%
  TATCCTGCCCGGTTCCTATCCTAAGTGCCAAGGATCCAATAGACAGAACTATTGAGTTTGTTCCCACCAAGACTCCCTAT

                          5921          .         .         :         .         .         .
  .         0 6000
  1 ruby_ACACA       100.0%
  GACCCTCGCTGGATGCTGGCTGGGCGACCAAACCCAAGTCAAAAAGGACAGTGGCTAAGTGGTTTCTTTGACAATGGCTC
  2 canna_ACACA       98.5%
  GACCCTCGCTGGATGCTGGCTGGGCGACCAAACCCAAGTCAAAAAGGACAGTGGCTAAGTGGTTTCTTTGACAATGGCTC
  3 swift_ACACA       92.4%
  GATCCTCGCTGGATGTTGGCTGGACGCCCAAATCCAAGTCAAAAAGGACAGTGGCTAAGTGGTTTCTTTGACAATGGCTC
  4 human_ACACA       77.6%
  GATCCTCGATGGATGCTAGCAGGCCGTCCTCACCCAACCCAAAAAGGTCAGTGGTTGAGTGGCTTTTTTGACTATGGATC
  5 gallus_ACACA      89.0%
  GATCCTCGCTGGATGCTGGCTGGACGCCCAAATCCAAGTCAAAAAGGGCAATGGCAGAGCGGTTTCTTTGACAATGGCTC
  6 alligator_ACACA   84.0%
  GATCCCCGGTGGATGTTGGCTGGACGTCCTCATCCAACTCAAAAAGGCCAGTGGTTAAGTGGGTTCTTTGACCATGGCTC

                          6001          .         .         .         .         :         .
  .         . 6080
  1 ruby_ACACA       100.0%
  ATTCCTGGAGATCATGCAGCCCTGGGCACAGACAGTTGTGGTTGGCAGAGCAAGGCTGGGAGGAATACCTGTAGGAGTCG
  2 canna_ACACA       98.5%
  ATTCCTGGAGATCATGCAGCCCTGGGCACAGACAGTTGTGGTTGGCAGAGCAAGGCTGGGAGGAATACCTGTAGGAGTCG
  3 swift_ACACA       92.4%
  ATTCCTGGAGATCATGCAGCCCTGGGCACAGACAGTTGTGGTTGGCAGAGCAAGGCTGGGAGGAATACCTGTAGGAGTAG
  4 human_ACACA       77.6%
  TTTCTCAGAGATTATGCAGCCCTGGGCACAGACTGTGGTGGTTGGTAGAGCCAGGCTAGGAGGAATACCTGTGGGAGTTG
  5 gallus_ACACA      89.0%
  GTTCCTGGAGATCATGCAGCCCTGGGCACAGACGGTTGTGGTTGGCAGAGCAAGGCTGGGAGGAATACCTGTAGGAGTAG
  6 alligator_ACACA   84.0%
  CTTCCTGGAGGTCATGCAGCCTTGGGCACAAACAGTTGTAGTTGGCAGAGCCAGGCTGGGAGGAATACCTGTAGGAGTAG

                          6081          .         1         .         .         .         .
  :         . 6160
  1 ruby_ACACA       100.0%
  TTGCCGTAGAAACCAGAACAGTGGAGCTCAGCATCCCTGCTGATCCTGCCAACCTGGACTCAGAGGCCAAGATAATCCAG
  2 canna_ACACA       98.5%
  TTGCCGTAGAAACCAGAACAGTGGAGCTCAGCATCCCTGCTGATCCTGCCAACCTGGACTCAGAGGCCAAGATAATCCAG
  3 swift_ACACA       92.4%
  TTGCCGTAGAAACGAGAACAGTGGAGCTCAGCATCCCTGCTGATCCTGCAAACCTGGACTCAGAGGCCAAGATAATCCAG
  4 human_ACACA       77.6%
  TTGCTGTAGAAACCCGAACAGTAGAACTAAGTATCCCAGCTGATCCAGCAAACCTGGATTCTGAAGCCAAGATAATCCAG
  5 gallus_ACACA      89.0%
  TTGCCGTAGAAACCAGAACAGTGGAGCTGAGCATCCCTGCTGATCCCGCCAACCTGGACTCGGAGGCCAAGATAATCCAG
  6 alligator_ACACA   84.0%
  TTGCCGTAGAAACCAGAACTGTGGAGCTAAGTATCCCTGCTGACCCTGCAAACCTGGACTCAGAGGCCAAGATAATCCAG

                          6161          .         .         .         2         .         .
  .         . 6240
  1 ruby_ACACA       100.0%
  CAGGCTGGTCAGGTGTGGTTCCCTGACTCTGCCTTCAAGACTGCTCAGGCTATCAATGACTTCAACAGGGAAGGGCTTCC
  2 canna_ACACA       98.5%
  CAGGCTGGTCAGGTGTGGTTCCCTGACTCTGCCTTCAAGACTGCTCAGGCTATCAATGACTTCAACAGGGAAGGGCTTCC
  3 swift_ACACA       92.4%
  CAGGCTGGTCAGGTCTGGTTCCCTGACTCTGCCTTTAAGACTGCACAGGCTGTCAAGGACTTCAACAGAGAAGGGCTGCC
  4 human_ACACA       77.6%
  CAGGCTGGCCAGGTTTGGTTCCCAGATTCTGCGTTTAAGACGTATCAGGCCATCAAGGACTTCAACCGGGAAGGGCTGCC
  5 gallus_ACACA      89.0%
  CAGGCTGGTCAGGTGTGGTTCCCCGACTCTGCCTTTAAGACAGCCCAGGCCATCAACGACTTCAACAGAGAAGGGCTGCC
  6 alligator_ACACA   84.0%
  CAGGCTGGTCAAGTGTGGTTCCCAGACTCTGCCTTTAAGACAGCCCAAGCTATCAAGGACTTTAACAGGGAGGGACTTCC

                          6241          :         .         .         .         .         3
  .         . 6320
  1 ruby_ACACA       100.0%
  TCTGATGGTCTTTGCCAACTGGAGAGGCTTCTCTGGTGGAATGAAAGACATGTATGACCAGGTGCTCAAGTTTGGTGCCT
  2 canna_ACACA       98.5%
  TCTGATGGTCTTTGCCAACTGGAGAGGCTTCTCTGGTGGAATGAAAGACATGTATGACCAGGTGCTCAAGTTTGGTGCCT
  3 swift_ACACA       92.4%
  TCTGATGGTCTTTGCTAACTGGAGGGGCTTCTCTGGTGGAATGAAAGACATGTATGACCAAGTGCTCAAGTTTGGTGCCT
  4 human_ACACA       77.6%
  TCTGATGGTCTTTGCCAACTGGAGAGGCTTCTCTGGTGGAATGAAAGATATGTACGACCAAGTGCTGAAGTTTGGTGCTT
  5 gallus_ACACA      89.0%
  TCTGATGGTCTTTGCCAACTGGAGAGGCTTCTCTGGTGGCATGAAAGACATGTACGACCAGGTGCTCAAGTTTGGTGCCT
```

```
  6 alligator_ACACA  84.0%
  TCTGATGGTCTTTGCCAACTGGCGAGGCTTCTCCGGTGGCATGAAAGACATGTATGACCAGATACTGAAGTTTGGTGCCT

                            6321          .         .         :         .         .         .
  .         4 6400
  1 ruby_ACACA      100.0%
  ACATTGTGGATGGCCTGAGAGAATACCATCAACCTGTGCTGATTTACATCCCCCCCCAGGCAGAGCTGAGGGGGGGTCC
  2 canna_ACACA      98.5%
  ACATTGTGGATGGCCTGAGAGAATACCATCAACCTGTGCTGATTTACATCCCCCCCCAGGCAGAGCTGAGGGGGGGTCC
  3 swift_ACACA      92.4%
  ACATCGTGGATGGCCTGAGGGAGTATTGTCAGCCCGTGCTTATTTACATCCCACCCCAGGCAGAGCTGAGGGGAGGATCC
  4 human_ACACA      77.6%
  ACATTGTGGATGGCTTGAGGGAGTGCTGCCAGCCTGTGCTGGTTTACATTCCTCCCCAGGCTGAGCTGCGGGGTGGCTCC
  5 gallus_ACACA     89.0%
  ACATCGTGGACGGCCTGCGGGAGTACCGGCAGCCCGTGCTCATCTACATCCCACCGCAGGCGGAGCTCAGGGGCGGCTCC
  6 alligator_ACACA  84.0%
  ACATCGTGGACGGCCTGCGGGAATACAGGCAACCAGTGATTGTTTACATCCCACCCCAGGCAGAGCTGAGAGGAGGATCC

                            6401          .         .         .         .         :         .
  .         . 6480
  1 ruby_ACACA      100.0%
  TGGGTTGTGGTTGACCCCACCATTAACCCCCGTCACATGGAGATGTACGCTGACCGGGAGAGCAGAGGAGGAGTCCTGGA
  2 canna_ACACA      98.5%
  TGGGTTGTGGTTGACCCCACCATTAACCCCCGTCACATGGAGATGTACGCTGACCGGGAGAGCAGAGGAGGAGTCCTGGA
  3 swift_ACACA      92.4%
  TGGGTTGTGGTTGACCCTACCATCAATCCTCGTCATATGGAGATGTACGCGGACCGAGAGAGCAGAGGAGGGATCCTGGA
  4 human_ACACA      77.6%
  TGGGTGGTGATTGACTCCTCCATCAACCCCCGGCACATGGAGATGTATGCTGACCGAGAAAGCAGGGGATCTGTTCTGGA
  5 gallus_ACACA     89.0%
  TGGGCTGTCATCGACCCCACCATCAACCCCAGGCACATGGAGATGTACGCGGACCGTGAAAGCAGAGGGGGAATCCTGGA
  6 alligator_ACACA  84.0%
  TGGGTGGTGATCGACCCCACCATTAACCCTCGACACATGGAGATGTATGCAGACCGGGACAGCAGAGGAGGAGTTCTGGA

                            6481          .         5         .         .         .         .
  :         . 6560
  1 ruby_ACACA      100.0%
  GCCAGAGGGGACGGTGGAGATCAAGTTCCGCAGGAAGGATCTGGTGAAGACGATGCGGCGCGTGGACCCCGTCTACATCC
  2 canna_ACACA      98.5%
  GCCAGAGGGGACAGTGGAGATCAAGTTCCGCAGGAAGGATCTGGTGAAGACGATGCGGCGCGTGGACCCCGTCTACATCC
  3 swift_ACACA      92.4%
  ACCCGAGGGGACAGTGGAAATCAAATTCCGCAGAAAGGATTTGGTGAAGACGATGCGGCGCGTGGACCCCGTCTACATCC
  4 human_ACACA      77.6%
  GCCAGAAGGGACAGTAGAAATCAAATTCCGCAGAAAGGATCTGGTGAAAACCATGCGTCGGGTGGACCCAGTCTACATCC
  5 gallus_ACACA     89.0%
  GCCGGAGGGGACGGTGGAAATCAAGTTCCGCAGGAAGGACCTGGTGAAGACAATGAGGAGAGTGGACCCCGTCTACATGC
  6 alligator_ACACA  84.0%
  GCCCGAAGGGACAGTAGAAATCAAATTCCGCAAGAAAGATTTGGTGAAGACAATGAGGCGAGTGGACCCAATCTACATTC

                            6561          .         .         .         6         .         .
  .         . 6640
  1 ruby_ACACA      100.0%
  GCCTGGCTGAGAGGCTGGGTACCCCTGAGCTGAGCCCCGCGGAGCGGAAGGAGCTGGAGACCAAGCTGAGGGAGAGGGAG
  2 canna_ACACA      98.5%
  GCCTGGCTGAGAGGCTGGGTACCCCTGAGCTGAGCCCCGCGGAGCGGAAGGAGCTGGAGACCAAGCTGAGGGAGAGGGAG
  3 swift_ACACA      92.4%
  GCCTGGCTGAGCGACTGGGTACCCCCGAGCTGAGCGCTGCCGACCGGAAGGACCTGGAGACAAAGCTGAGGGAGCGGGAG
  4 human_ACACA      77.6%
  ACTTGGCTGAGCGATTGGGGACCCCAGAGCTAAGCACAGCTGAGCGGAAGGAGTTGGAGAACAAGTTGAAGGAGCGGGAG
  5 gallus_ACACA     89.0%
  GGCTGGCCGAGCGGCTGGGTACCCCTGAGCTGAGTGCTGCCGACCGAAAAGACCTGGAGAGCAAACTGAAGGAGCGGGAG
  6 alligator_ACACA  84.0%
  GGCTGGCAGAGCGACTAGGCACCCCGGAGCTCAGCCCGGCTGAGCGGAAGGAGCTGGAGACGAAGCTGAAGGAGCGGGAG

                            6641          :         .         .         .         .         7
  .         . 6720
  1 ruby_ACACA      100.0%
  GAATTCCTGATCCCCATGTACCAGCAGGTGGCTGTGCAGTTTGCTGACCTGCACGACACACCAGGACGGATGCAGGAGAA
  2 canna_ACACA      98.5%
  GAATTCCTGATCCCCATGTACCAGCAGGTGGCTGTGCAGTTTGCTGACCTGCACGACACACCAGGACGGATGCAGGAGAA
  3 swift_ACACA      92.4%
  GAATTCCTGATTCCCATCTACCAGCAGGTGGCCGTGCAGTTTGCTGACTTGCACGACACACCCGGCCGGATGCAGGAGAA
  4 human_ACACA      77.6%
  GAATTCCTAATTCCCATTTACCATCAGGTAGCCGTGCAGTTTGCTGACTTGCACGACACACCAGGCCGGATGCAGGAGAA
  5 gallus_ACACA     89.0%
  GAATTCCTGATTCCCATTTACCACCAGGTGGCCATGCAGTTTGCTGACCTGCACGACACACCCGGCCGCATGCAGGAGAA
  6 alligator_ACACA  84.0%
  GAATTCCTGACGCCCATCTATCACCAGGTGGCTGTGCAGTTTGCTGACTTGCATGACACGCCAGGCCGCATGCAGGAGAA

                            6721          .         .         :         .         .         .
  .         8 6800
  1 ruby_ACACA      100.0%
```

```
GGGAGCCATCACTGACATTCTAGACTGGAAAACCTCCCGTACCTTCTTCTACTGGAGATTGAGACGTCTCCTGCTGGAAG
2 canna_ACACA      98.5%
GGGAGCCATCACTGACATTCTAGACTGGAAAACCTCCCGTACCTTCTTCTACTGGAGATTGAGACGTCTCCTGCTGGAAG
3 swift_ACACA      92.4%
GGGTGCCATCACTGACATTCTAGATTGGAAAACTTCCCGTACCTTCTTCTACTGGAGATTAAGACGACTTCTTCTGGAAG
4 human_ACACA      77.6%
GGGTGTTATTAGCGATATCCTGGATTGGAAAACATCCCGTACCTTCTTCTACTGGCGGCTGAGGCGTCTTCTGCTGGAGG
5 gallus_ACACA     89.0%
GGGTGCCATCACGGACATTCTGGACTGGAAAACGTCTCGGACCTTCTTCTACTGGAGGCTGAGACGTCTTCTTCTGGAAG
6 alligator_ACACA  84.0%
AGCTGTCATCACCGACATTCTGGAGTGGAAAACCTCCCGGACTTTCTTCTACTGGAGGTTGCGGCGCCTGCTGCTGGAAG

                         6801            .         .         .         .         :         .
.         . 6880
1 ruby_ACACA      100.0%
AGATGGTGAAAAAGAAAATCCATGATGCCAACCCTGAGCTGACCGACGGGCAGATCCAGGCCATGCTGCGCCGCTGGTTT
2 canna_ACACA      98.5%
AGATGGTGAAAAAGAAAATCCATGATGCCAACCCTGAGCTGACCGACGGGCAGATCCAGGCCATGCTGCGCCGCTGGTTT
3 swift_ACACA      92.4%
ATGTGGTCAAAAAGAAGATCCATGATGCCAACCCTGAGCTGACTGATGGGCAGATCCAGGCTATGCTGAGACGCTGGTTT
4 human_ACACA      77.6%
ACCTGGTCAAGAAGAAAATCCACAATGCCAACCCTGAGCTGACTGATGGCCAGATTCAAGCCATGTTAAGGCGCTGGTTT
5 gallus_ACACA     89.0%
ATGTGGTCAAAAAGAAGATCCATGATGCCAACCCTGAGCTGACCGACGGGCAGATCCAGGCCATGCTGCGACGCTGGTTT
6 alligator_ACACA  84.0%
ACGTGGTCAAGAAAAAGATCCACGATGCCAACCCTGAGCTGACGGATGGGCAGATCCAGGCCATGCTGAGGCGCTGGTTT

                         6881            .         9         .         .         .         .
:         . 6960
1 ruby_ACACA      100.0%
GTGGAGGCCGAGGGGACCGTGAAGGCCTATGTGTGGGACAGCAACAAGGACGTGGTGGAGTGGCTGGAGAAGCAGCTGAC
2 canna_ACACA      98.5%
GTGGAGGCCGAGGGGACCGTGAAGGCCTATGTGTGGGACAGCAACAAGGACGTGGTGGAGTGGCTGGAGAAGCAGCTGAC
3 swift_ACACA      92.4%
GTGGAAGCTGAAGGGACAGTGAAGGCCTACCTGTGGGACAGCAATAAGGACCTGGTGGAGTGGCTGGAGAAACAGCTGAC
4 human_ACACA      77.6%
GTGGAAGTGGAAGGAACAGTGAAGGCTTATGTTTGGGACAATAATAAGGATCTGGCGGAGTGGCTAGAGAAACAGCTGAC
5 gallus_ACACA     89.0%
GTGGAAGTGGAGGGGACGGTAAAGGCGTACCTGTGGGACAGCAATAAGGACCTGGTGGAGTGGCTGGAGAAGCAGCTGAT
6 alligator_ACACA  84.0%
GTGGAAGTCGAGGGGACAGTCAAGGCGTACTTGTGGGATAACAACAAGGACCTGGTGGAGTGGCTGGAGAAACAGCTCAC

                         6961            .         .         .         0         .         .
.         . 7040
1 ruby_ACACA      100.0%
AGAAGAAGAAGGCGTCCGCTCCGTCGTGGATGAAAACATCAAATACATCTCCAGGGACTACATCCTGAAACAGATCCGGA
2 canna_ACACA      98.5%
AGAAGAAGAAGGCGTCCGCTCCGTCGTGGATGAAAACATCAAATACATCTCCAGGGACTACATCCTGAAACAGATCCGGA
3 swift_ACACA      92.4%
GGAAGAAGAAGGTGTTCGCTCTGTTGTGGATGAAAACATCAAGTACATCTCCAGGGACTACATCCTCAAACAGATCCGCA
4 human_ACACA      77.6%
AGAGGAGGATGGTGTTCACTCGGTAATAGAGGAAAACATCAAATGCATCAGCAGAGACTACGTCCTCAAGCAAATCCGCA
5 gallus_ACACA     89.0%
GGAGGAGGAGGGGGTTCGCTCGGTTGTGGATGAGAACATTAAGTACATCTCCAGGGATTACATCCTGAAGCAGATCCGCA
6 alligator_ACACA  84.0%
AGAAGAAGAAGGCGTTCGCTCAGTCGTGGAGGAAAACATCAAATACATCTCCAGAGATTACATCCTCAAGCAGATACGGA

                         7041            :         .         .         .         .         1
.         . 7120
1 ruby_ACACA      100.0%
GCCTGGTCCAGGCCAATCCAGAGGTTGCCATGGATTCGATCGTGCACATGACCCAGCATATATCACCCACCCAGCGAGCA
2 canna_ACACA      98.5%
GCCTGGTCCAGGCCAATCCAGAGGTTGCCATGGATTCGATCGTGCACATGACCCAGCATATATCACCCACCCAGCGAGCA
3 swift_ACACA      92.4%
GCCTGGTCCAGGCCAATCCAGAGGTTGCCATGGATTCCATAGTGCACATGACCCAGCATATATCACCCACCCAGCGAGCT
4 human_ACACA      77.6%
GCTTGGTCCAGGCCAATCCAGAGGTTGCCATGGATTCCATCATCCATATGACGCAGCACATATCACCCACTCAGCGAGCA
5 gallus_ACACA     89.0%
GCCTGGTCCAGGCCAATCCCGAGGTTGCCATGGATTCGATCGTGCACATGACCCAGCATATATCACCCACCCAGCGAGCC
6 alligator_ACACA  84.0%
GTTTGGTACAGGCCAATCCCGAGGTTGCCATGGATTCCATCGTTCACATGACCCAGCATATATCACCGACCCAGCGAGCC

                         7121            .         .         :         .       ] 7168
1 ruby_ACACA      100.0%      GAGATTGTGCGGATCCTCTCCACAATGGATTCACCTTCTTCAACGTAA
2 canna_ACACA      98.5%      GAGATTGTGCGGATCCTCTCCACAATGGATTCACCTTCTTCAACGTAA
3 swift_ACACA      92.4%      GAGATCGTGAGGATCCTCTCCACAATGGACTCGCCTTCTTCAACATAA
4 human_ACACA      77.6%      GAAGTCATACGGATCCTCTCCACAATGGATTCCCCTTCC---ACGTAG
5 gallus_ACACA     89.0%      GAGATCGTGCGGATCCTCTCCACAATGGACTCTCCTTCTTCAACGTAA
6 alligator_ACACA  84.0%      GAGATAGTGCGGATTCTTTCAACAATGGACTCGCCTCCTTCCACGTAA
```

MView 1.60.1, Copyright © 1997-2015 Nigel P. Brown

```
Reference sequence (1): ruby_DGAT2
Identities normalised by aligned length.
Colored by: identity + property


                             1 [        .         .         .         .         :         .
.         . 80
1 ruby_DGAT2      100.0%      --------------------------------------------------------------------
------------
2 canna_DGAT2      61.8%      --------------------------------------------------------------------
------------
3 swift_DGAT2      63.4%      --------------------------------------------------------------------
------------
4 human_DGAT2      58.4%
ATGAAGACCCTCATAGCCGCCTACTCCGGGGTCCTGCGCGGCGAGCGTCAGGCCGAGGCTGACCGGAGCCAGCGCTCTCA
5 gallus_DGAT2     63.4%      --------------------------------------------------------------------
------------
6 alligator_DGAT2  61.2%      --------------------------------------------------------------------
------------

                            81         .         1         .         .         .         .
:         . 160
1 ruby_DGAT2      100.0%
ATGAAAACAATCATTGCAGCCTGCTCCCAA-AACCTCAGTGGCAGCCGTGCCAGTGTCCAGACTGCCCTGCACACCCTGC
2 canna_DGAT2      61.8%      ATGAAAACCATCATCGCTGCTTATTCGGGG-
GTGCTGCGAGGCACAGGGTCGAACATTCTTTCTTCTCTGCAGGATTTGT
3 swift_DGAT2      63.4%      --------------------------------------------------------------------
------------
4 human_DGAT2      58.4%
CGGAGGACCTGCGCTGTCGCGCGAGGGGTCTGGGAGATGGGGCACTGGATCCAGCATCCTCTCCGCCCTCCAGGACCTCT
5 gallus_DGAT2     63.4%      ATGAAAACCATCATCGCCGCGTACTCGGGG-
GTGCTGCGAGGTACGGGGTCGAGCATTCTCTCTGCTCTGCAGGACTTGT
6 alligator_DGAT2  61.2%      ATGAAGACGCTCATTGCTGCCTACTCGGGC-
GTCTTGCGAGGCACTGGGTCAAACATCCTCTCTGCCCTTCAGGATGTAT

                           161         .         .         .         2         .         .
.         . 240
1 ruby_DGAT2      100.0%
TGAGGGCACCGTGGCCTTCCCAGAGGAATCTCCGCTCCTGGATGCAGCGCCTCGCCGTCCTGCAGTGGGTGCTCAGCTTC
2 canna_DGAT2      61.8%      TCT--------
-GGTTGTCTAAATCCAAGCTAGAGAAACAACTCCAAATCATCTCTGTGCTGCAATGGGTTCTCACTTTC
3 swift_DGAT2      63.4%      --------------------------------------------------------------------
------------
4 human_DGAT2      58.4%
TCTCTGTCACCTGGCTCAATAGGTCCAAGGTGGAAAAGCAGCTACAGGTCATCTCAGTGCTCCAGTGGGTCCTGTCCTTC
5 gallus_DGAT2     63.4%      TCT--------
-GGCTGTCTAAATCCAAAGTAGAGAAACAACTGCAGATCATCTCCGTGCTGCAATGGGTCCTCACGTTC
6 alligator_DGAT2  61.2%      TCT--------
-GGTTCTCAAAGTCCAAGTTGAGAAACAGCTCCAGATTATATCCGTGCTGCAATGGGTTCTGTCTTTC

                           241         :         .         .         .         .         3
.         . 320
1 ruby_DGAT2      100.0%
CTGCTGCTGGGAATTGTCAGCCTGCTGGTCCTCATCTACTTGGTGTTCACCAGCTTCTGGGCCATCTCTGCCCTCTACCT
2 canna_DGAT2      61.8%
CTAGTCATGGGTGTTGCTTGCACTTTAATCCTCATGTACATACTGTGCACAGATTGTTGGGCCATTGCTGCTCTTTATTT
3 swift_DGAT2      63.4%      ------
ATGGGTGTTGCTTGCACTTTAATCCTCATGTACATACTGTGCACAGACTGCTGGGCCATTGCTGCCCTATATTT
4 human_DGAT2      58.4%
CTTGTACTGGGAGTGGCCTGCAGTGCCATCCTCATGTACATATTCTGCACTGATTGCTGGCTCATCGCTGTGCTCTACTT
5 gallus_DGAT2     63.4%
CTCATCATGGGTATTGCTTGCACTTTAATCCTCATGTACATCCTGTGCACAGATTGCTGGGCGATCGCTGCTCTGTATTT
6 alligator_DGAT2  61.2%
CTTGTCCTGGGTGTTACTTGCTCTATCATACTCGTGTACATCCTCTGCACAGACTGCTGGGCAATTGCAGCACTGTATGT

                           321         .         .         :         .         .         .
.        4 400
1 ruby_DGAT2      100.0%
GGCCTGGATCATCTTTGACTGGGACACCCCGGAGAGAGGTGGCCGGAGGCTGGCCTGCCTGCGGGGGTGGCCCATCTGGA
2 canna_DGAT2      61.8%
AGCCTGGCTGGTATTTGACTGGAATACACCAAAGAAAGGTGGAAGAAGATCCCAATGGGTGAGGAACTGGGCTATATGGA
3 swift_DGAT2      63.4%
AGCCTGGCTGGTTTTTGACTGGAATACACCAAAGAAAGGTGGAAGAAGATCCCAATGGGTGAGAAACTGGGCTATATGGA
4 human_DGAT2      58.4%
CACTTGGCTGGTGTTTGACTGGAACACACCCAAGAAAGGTGGCAGGAGGTCACAGTGGGTCCGAAACTGGGCTGTGTGGC
5 gallus_DGAT2     63.4%
```

```
AGCCTGGCTGGTGTTCGACTGGAATACACCAAAGAAAGGTGGAAGAAGATCCCAGTGGGTGAGAAATTGGGCTATATGGA
6 alligator_DGAT2  61.2%
GGCATGGCTGGTGTTCGATTGGGATACTCCAATGAAGGGTGGAAGAAGATCCCCGTGGGTCAGAAACTGGGCCATGTGGC

                          401          .          .          .          .          :          .
.         . 480
1 ruby_DGAT2      100.0%
ACCACTTTCGGGATTATTTCCCTGTGAAGCTGGTGAAGACCCACGAGCTGTCCCCCAGCCACAACTACATCATTGGCTCC
2 canna_DGAT2      61.8%
GATACTTCAGGGATTATTTTCCAATAAGACTGGTTAAAACCCACAATCTGCTGACCACAAGGAATTACATTTTTGGGTAC
3 swift_DGAT2      63.4%
GGTACTTCAGGGATTATTTTCCAATTAGACTGGTTAAAACCCACAATCTGCTGACCACCAGGAATTACATTTTTGGGTAC
4 human_DGAT2      58.4%
GCTACTTTCGAGACTACTTTCCCATCCAGCTGGTGAAGACACACAACCTGCTGACCACCAGGAACTATATCTTTGGATAC
5 gallus_DGAT2     63.4%
GGTACTTCAGGGATTATTTCCCAATAAGACTGGTGAAAACCCACAACCTGCTGACCACCAGGAATTACATCTTCGGTTAC
6 alligator_DGAT2  61.2%
GGTACTTCAGAGATTATTTTCCAATACGGCTGGTGAAGACCCACAACTTGCCAACCAATCGGAATTACATATTTGGCTAC

                          481          .          5          .          .          .          .
:         . 560
1 ruby_DGAT2      100.0%
CACCCCCACGGCATCCTCTGCGTCGGCGCCTTCTGCAACTTCGTCACCGGCTCCACGGGATTCGGGCAGAAATTCCCAGG
2 canna_DGAT2      61.8%
CACCCTCATGGCATCATGGGCTTGGGTGCCTTTTGCAACTTCAGCACAGAGGCCACAGGTGTTGGCCAGAAATTTCCTGG
3 swift_DGAT2      63.4%
CATCCACATGGAATCATGGGCTTGGGTGCCTTTTGCAACTTCAGCACAGAAGCTACAGGTGTCAGCCAGAAATTCCCTGG
4 human_DGAT2      58.4%
CACCCCCATGGTATCATGGGCCTGGGTGCCTTCTGCAACTTCAGCACAGAGGCCACAGAAGTGAGCAAGAAGTTCCCAGG
5 gallus_DGAT2     63.4%
CATCCGCATGGCATCATGGGCTTGGAGCCTTTTGCAACTTCAGCACAGAGGCCACGGGAGTCAGCCAGAAATTCCCTGG
6 alligator_DGAT2  61.2%
CACCCACATGGCATCATGGGCTTTGGCGCGTTCTGCAACTTTGGCACAGAAGCCACCGGGGTCAGCCAGAAGTTTCCTGG

                          561          .          .          .          6          .          .
.         . 640
1 ruby_DGAT2      100.0%
CATCCGGCCCTCCCTCACCACGCTGGCCGGCAACTTCCGTCTGCCCCTCTTCAGGGAGTACCTCATGAGTGGGGGGCTGT
2 canna_DGAT2      61.8%
GATCCGACCCTACCTTGCTACCCTGGCTGGGAACTTCAGGATGCCCATTTTGAGGGACTACTTAATGTCTGGTGGTATAT
3 swift_DGAT2      63.4%
GATTCGACCATACCTTGCTACCCTGGCTGGGAACTTCAGGATGCCCATTTTGAGGGACTACTTAATGTCTGGTGGTATAT
4 human_DGAT2      58.4%
CATACGGCCTTACCTGGCTACACTGGCAGGCAACTTCCGAATGCCTGTGTTGAGGGAGTACCTGATGTCTGGAGGTATCT
5 gallus_DGAT2     63.4%
GATCCGGCCGTACCTGGCCACCCTGGCTGGGAACTTCAGGATGCCCATCCTGAGGGACTACTTAATGTCTGGGGGTATAT
6 alligator_DGAT2  61.2%
CATTAGACCGTATCTTGCTACGTTGGCTGGGAACTTCCGGATACCAATTTTGAGGGACTACTTGATGTCCGGTGGTATTT

                          641          :          .          .          .          .          7
.         . 720
1 ruby_DGAT2      100.0%
GCCCGGTGACACGCAGTGCCATGGGGTACCTGCTGGCCAAGAACGGCACCGGTAATGCGGTGGCCATCGTCATCGGGGGG
2 canna_DGAT2      61.8%
GTCCTGTGAACCGTGACAGCATAGACTACATCTTGTCCAAGAATGGCACTGGCAATGCCATCATCATCGTGGTTGGAGGG
3 swift_DGAT2      63.4%
GTCCTGTGAACCGGGACAGCATAGACTACATCTTGTCCAAGAATGGCAGTGGCAATGCCATCATCATCGTGGTTGGAGGG
4 human_DGAT2      58.4%
GCCCTGTCAGCCGGGACACCATAGACTATTTGCTTTCAAAGAATGGGAGTGGCAATGCTATCATCATCGTGGTCGGGGGT
5 gallus_DGAT2     63.4%
GTCCCGTGAACCGTGACAGCATAGACTACATCTTGTCCAAGAATGGCAGTGGCAATGCCATCATCATCGTGGTGGGAGGA
6 alligator_DGAT2  61.2%
GCCCTGTGAACCGTCACTCCATAGATTTCATCCTATCCAAGAATGGCACTGGCAACGCCGTGATCATTGTGGTGGGGGGA

                          721          .          .          :          .          .          .
.         8 800
1 ruby_DGAT2      100.0%
GCGGCCGAGTCCCTGTCCTGCCGCCCCGGTGTCACCACCCTCATCCTCAAGAACCGCAAGGGCTTCGTCCGTATGGCCCT
2 canna_DGAT2      61.8%
GCAGCAGAGTCCTTGAACTGCACCCCGGGCAAGAACTCGGTGACGCTGAAAAATCGGAAAGGATTTGTGAAGCTGGCTCT
3 swift_DGAT2      63.4%
GCAGCAGAGTCCCTGAACTGCACCCCGGGGAAGAACTCGGTGACGCTGAAGAACAGGAAGGGATTTGTGAAGCTGGCTCT
4 human_DGAT2      58.4%
GCGGCTGAGTCTCTGAGCTCCATGCCTGGCAAGAATGCAGTCACCCTGCGGAACCGCAAGGGCTTTGTGAAACTGGCCCT
5 gallus_DGAT2     63.4%
GCAGCGGAGTCCCTCAACTGCACCCCTGGGAAGAACTCGGTGACACTGAGAAACAGGAAGGGATTTGTGAAACTGGCACT
6 alligator_DGAT2  61.2%
GCAGCAGAGTCCCTGAACTGCACGCCTGGGAAGAACTTGGTGATTCTGAAAAATCGGAAAGGATTTGTAAAGCTGGCTCT

                          801          .          .          .          .          :          .
.         . 880
```

```
1 ruby_DGAT2      100.0%
GGAGCACGGGGCCTACCTCGTCCCCTCCTTCACCTTCGGGGAGAATGACCTCTACCACCAGGTGGTCTTCAAGGAGGGCA
2 canna_DGAT2      61.8%
GAGACATGGGGCGGACTTGGTTCCTGTCTACTCATTTGGGGAGAACGAAGTGTACAAGCAAGTGATCTTTGAGGAGGGCT
3 swift_DGAT2      63.4%
GAGACATGGGGCGGACTTGGTTCCCGTCTACTCCTTTGGGGAGAACGAGGTGTACAAGCAGGTGATCTTTGAGGAGGGCT
4 human_DGAT2      58.4%
GCGTCATGGAGCTGACCTGGTTCCCATCTACTCCTTTGGAGAGAATGAAGTGTACAAGCAGGTGATCTTCGAGGAGGGCT
5 gallus_DGAT2     63.4%
GCGGCACGGTGCCGACTTGGTTCCTGTCTACTCTTTTGGGGAGAACGAAGTGTACAAGCAGGTGATCTTTGAGGAGGGCT
6 alligator_DGAT2  61.2%
GCAGCATGGGGCAGATCTAGTTCCCGTGTACTCATTTGGCGAGAACGAGGTGTACAAGCAGGTGATCTTCGAGGAGGGCT

                           881         .         9         .         .         .
:         . 960
1 ruby_DGAT2      100.0%
GCTGGATGAGGAGCATCCAGAGTTGCTTCCAGAAGCTGATTGGCTTTGCTCCTTGTGTCTTCTATGGCCGGGCTCTCACC
2 canna_DGAT2      61.8%
CCTGGGGAAGATGGGTTCAGAAGAAGTTTCAGAAGCACATTGGATTTGCTCCCTGCATCTTTCATGGCCGTGGCCTCTTC
3 swift_DGAT2      63.4%
CCTGGGGAAGATGGGTTCAGAAGAAGTTTCAGAAGCACATTGGCTTTGCTCCATGCATCTTTCATGGCCGTGGCCTCTTC
4 human_DGAT2      58.4%
CCTGGGGCCGATGGGTCCAGAAGAAGTTCCAGAAATACATTGGTTTCGCCCCATGCATCTTCCATGGTCGAGGCCTCTTC
5 gallus_DGAT2     63.4%
CCTGGGGAAGATGGGTTCAGAAGAAGTTTCAGAAGCACATTGGCTTTGCTCCATGCATCTTCCATGGCCGTGGGCTCTTC
6 alligator_DGAT2  61.2%
GCTGGGGCAGATGGGTCCAGAAGAAGTTTCAGAAGTACATTGGCTTTGCTCCGTGCATCTTTCATGGTCGTGGCCTCTTC

                           961         .         .         .         0         .
.         . 1040
1 ruby_DGAT2      100.0%
TCCGTCCAGTCCCGGGGATTACTCCCCTATGAAAAACCCATCACCACCGTGGTGGGGGAGCCCTTGGTGGTGCCCAAGGT
2 canna_DGAT2      61.8%
TCCTCCAACACCTGGGGATTGTTACCTTACTCCAAGCCCATCACTACTGTTGTTGGGGAACCCATCACCATCCCCAAAGT
3 swift_DGAT2      63.4%
TCCTCTAACACCTGGGGGTTGTTACCTTACTCCAAGCCCATCACTACTGTTGTTGGGGAGCCCATCACCATTCCCAAAAT
4 human_DGAT2      58.4%
TCCTCCGACACCTGGGGGCTGGTGCCCTACTCCAAGCCCATCACCACTGTTGTGGGAGAGCCCATCACCATCCCCAAGCT
5 gallus_DGAT2     63.4%
TCCTCGAACACGTGGGGCTTGCTGCCGTACTCCAAGCCCATCACCACTGTTGTGGGCGAACCCATCACCATCCCAAAGAT
6 alligator_DGAT2  61.2%
TCTTCCAACACCTGGGGTTTGATCCCTTACCCTAACCCCATCACCACAGTTGTTGGGGAGCCAATCACCATCCCGAAAAC

                          1041         :         .         .         .         .         1
.         . 1120
1 ruby_DGAT2      100.0%
CAGGAACCCCAGCAGCGAGATGGTGGACACGTACCACCAGATGTACATCAGCTCCCTGATCAAGCTCTTCCACGAGAACA
2 canna_DGAT2      61.8%
TGATAATCCATCCCAGGAAGAAGTGGATTTCTACCACAGCATCTATGTGGACTCCCTGATCAAACTCTTTGACAAGTACA
3 swift_DGAT2      63.4%
TGAGAATCCATCCCAGCAGGACGTGGACTTCTACCATAGCATGTATGTGGACTCCCTGATCAAACTCTTTGACAAGTACA
4 human_DGAT2      58.4%
GGAGCACCCAACCCAGCAAGACATCGACCTGTACCACACCATGTACATGGAGGCCCTGGTGAAGCTCTTCGACAAGCACA
5 gallus_DGAT2     63.4%
CGATAACCCATCTCAGAAGGAAGTGGACTTCTACCACAGCGTGTACGTGGACTCCCTGATCAAGCTCTTTGACAAGTACA
6 alligator_DGAT2  61.2%
AGCTCATCCCACCCAGAGGGAAATCGACCTTTATCACAGCATGTACGTGAGCTCGCTGAGTAAGCTCTTTGACAAGTACA

                          1121         .         .         :         .         .         .   ]
1184
1 ruby_DGAT2      100.0%      AGACCAAGTATGGGATGTTGGAGACGGACGAGCTGCACATCCTCTG-----------------
2 canna_DGAT2      61.8%      AGATCAAATTCGGCCTGCCAGAGACTGAGGTCTTGGAAGTCAACTGA----------------
3 swift_DGAT2      63.4%      AGAGCAAATTTGGGCTAAGGAAAACTGATACTTTGATTATTGTGTGTGCCCAGGCTGCATAA--
4 human_DGAT2      58.4%      AGACCAAGTTCGGCCTCCCGGAGACTGAGGTCCTGGAGGTGAACTGA----------------
5 gallus_DGAT2     63.4%      AAGGCAGGTTCGGGTTGCCAGAGACTGAGGTCCTGGAAGTCAACTGA----------------
6 alligator_DGAT2  61.2%      AGGCTAAGTTCGGCCTGCCAGAGACGGAGATCTTGGAAGTCAACTGA----------------
```

MView 1.60.1, Copyright © 1997-2015 Nigel P. Brown

```
Reference sequence (1): ruby_FASN
Identities normalised by aligned length.
Colored by: identity + property

                            1 [        .         .         .         .         :         .
.         . 80
1 ruby FASN      100.0%
```

ATGGAGGACGTGGTGATTGCAGGCATAGCAGGAAAGCTGCCAGAATCAGAGAACTTGCAAGAGTTTTGGGAGAACCTGCT
2 canna_FASN 99.7%
ATGGAGGACGTGGTGATTGCAGGCATAGCAGGAAAGCTGCCAGAATCAGAGAACTTGCAAGAGTTTTGGGAGAACCTGCT
3 swift_FASN 90.8%
ATGGAGGACGTGGTGATTGCAGGCATAGCAGGAAAGCTGCCAGAATCAGAGAACTTGGAAGAATTTTGGGAGAACCTGCT
4 human_FASN 64.1%
ATGGAGGAGGTGGTGATTGCCGGCATGTCCGGGAAGCTGCCAGAGTCGGAGAACTTGCAGGAGTTCTGGGACAACCTCAT
5 gallus_FASN 88.9%
ATGGAAGACGTGGTGATCGCAGGCATAGCAGGAAAGCTGCCGGAGTCTGAGAACTTACAAGAGTTTTGGGAGAACCTGCT
6 alligator_FASN 75.2%
ATGGAGGACGTGGTGATTGCAGGCATTGCAGGGCGGCTGCCCGAGTCGGAGAGCTTGGAGGAGTTCTGGGAGAACTTGGT

81 . 1 . . . .
: . 160
1 ruby_FASN 100.0%
TAATGGAGTTGATATGGTCACAGAAGATGATCGGAGGTGGAAACCAGGAATTTATGGACTGCCCAGGAGAAATGGAAAGC
2 canna_FASN 99.7%
TAATGGAGTTGATATGGTCACAGAAGATGATCGGAGGTGGAAACCAGGAATTTATGGACTGCCCAGGAGAAATGGAAAGC
3 swift_FASN 90.8%
TAATGGAGTTGATATGGTCACAGAGGATGATCGGAGGTGGAAACCAGGAATGTATGGACTGCCCAAGAGAAATGGAAAGC
4 human_FASN 64.1%
CGGCGGTGTGGACATGGTCACGGACGATGACCGTCGCTGGAAGGCGGGGCTCTACGGCCTGCCCCGGCGGTCCGGCAAGC
5 gallus_FASN 88.9%
CAATGGGGTTGATATGGTCACAGAGGACGATCGGAGGTGGAAGCCAGGGATTTATGGACTGCCCAAAAGAAATGGAAAGC
6 alligator_FASN 75.2%
TGGTGGCGTTGACATGGTCACAGATGACGAGCGGAGGTGGAAGTCAGGACTTCATGGGCTACCCCGTAGAACTGGGAAAC

161 . . . 2 . .
. . 240
1 ruby_FASN 100.0%
TCAAGGACATAAGCAAATTTGATGCATCTTTTTTCGGGGTCCACCCCAAACAAGCTCATACTATGGATCCTCAACTTCGC
2 canna_FASN 99.7%
TCAAGGACATAAGCAAATTTGATGCATCTTTTTTTGGGGTCCACCCCAAACAAGCTCATACTATGGATCCTCAACTTCGC
3 swift_FASN 90.8%
TCAAGGACATAAGCAAATTTGATGCATCCTTTTTTGGGGTCCACCCCAAACAAGCTCATACGATGGACCCTCAACTTCGC
4 human_FASN 64.1%
TGAAGGACCTGTCTAGGTTTGATGCCTCCTTCTTCGGAGTCCACCCCAAGCAGGCACACACGATGGACCCTCAGCTGCGG
5 gallus_FASN 88.9%
TCAAGGACATAAAAAAATTTGATGCCTCCTTCTTTGGGGTCCACCCCAAACAAGCTCATACAATGGATCCTCAGCTTCGC
6 alligator_FASN 75.2%
TGAATGACATCAGCAAATTTGATGCCTCCTTTTTTGGGACTCTGCCCAAACAAGCCGCTGTAATGGACCCTCAACTCCGC

241 : 3
. . 320
1 ruby_FASN 100.0%
TTGCTGTTGGAAGTTTCTTATGAAGCTATTTTGGATGCAGGTATTGATCCAGCCACCCTCCGTGGCACTGACACAGGTGT
2 canna_FASN 99.7%
TTGCTGTTGGAAGTTTCTTATGAAGCTATTTTGGATGCAGGTATTGATCCAGCCACCCTCCGTGGCACTGACACAGGTGT
3 swift_FASN 90.8%
ATGCTGTTGGAAGTTTCTTATGAAGCTATTTTGGATGCAGGTATTAATCCAGCCACGCTCCGTGGCACAGACACAGGTGT
4 human_FASN 64.1%
CTGCTGCTGGAAGTCACCTATGAAGCCATCGTGGACGGAGGCATCAACCCAGATTCACTCCGAGGAACACACACTGGCGT
5 gallus_FASN 88.9%
TTGTTGTTGGAAGTTTCTTATGAAGCTATTTTGGATGGAGGCATTAATCCAACTGCCCTCCGTGGCACAGACACGGGTGT
6 alligator_FASN 75.2%
CTGCTTCTGGAGGTCTCTTATGAAGCCATACTGGATGGAGGTATCAATCCAGCCTCCCTTCGTGGCTCAAACACGGGTGT

321 . . : . . .
. 4 400
1 ruby_FASN 100.0%
ATGGGTTGGTGCCAGTGGGTCAGAAGCTGCGGAAGCTCTTAGCCAAGATCCAGAAGAGCTTTTGGGATACAGTATGACTG
2 canna_FASN 99.7%
ATGGGTTGGTGCCAGTGGGTCAGAAGCTGCGGAAGCTCTTAGCCAAGATCCAGAAGAGCTTTTGGGATACAGTATGACTG
3 swift_FASN 90.8%
ATGGATTGGTACCAGTGGGTCAGAAGCTGGTGAAGCCCTTAGCCAAGATCCAGAAGAGCTGGTGGGATACAGTATGACTG
4 human_FASN 64.1%
CTGGGTGGGCGTGAGCGGCTCTGAGACCTCGGAGGCCCTGAGCCGAGACCCCGAGACACTCGTGGGCTACAGCATGGTGG
5 gallus_FASN 88.9%
ATGGGTTGGTGCAAGTGGCTCAGAAGCTGCTGAAGCCCTTAGCCAAGATCCAGAAGAGCTTTTGGGATACAGTATGACTG
6 alligator_FASN 75.2%
GTGGATTGGTATAAGTGGGGCCGAAGCAATTGAAGTGCTTAGCCAAGACCCTGAGACAGTAGTGGGGTACAGCATGATTG

401 . . . . : .
. . 480
1 ruby_FASN 100.0%
GTTGCCAGCGTGGTATGTTTGCCAACAGGATTTCCTACTTTTATGACTTAACAGGACCAAGTTTAACTATTGACACAGCC
2 canna_FASN 99.7%
GTTGCCAGCGTGGTATGTTTGCCAACAGGATTTCCTACTTTTATGACTTAACAGGACCAAGTTTAACTATTGACACAGCC
3 swift_FASN 90.8%
GCTGCCAGCGTGCCATGTTTCCAACAGGATTTCCTACTTTTTAGACTTAACAGGACCAAGCATAACCATTGACACAGCC
4 human_FASN 64.1%

```
GCTGCCAGCGAGCGATGATGGCCAACCGGCTCTCCTTCTTCTTCGACTTCAGAGGGCCCAGCATCGCACTGGACACAGCC
5 gallus_FASN     88.9%
GCTGCCAGCGTGCTATGCTTGCCAACAGGATTTCTTACTTCTATGATTTTACAGGACCAAGCTTAACTATCGACACAGCA
6 alligator_FASN  75.2%
GCAGCCAGCGTGCCATGTTTGCCAACAGATTGTCCTTCTTCTACGACCTCAAAGGACCCAGCATGTCTGTTGACGCAGCA

                         481          .         5         .         .         .         .
:         . 560
1 ruby_FASN      100.0%
TGCTCCTCTAGTCTCATTGCTCTGGAAAATGCTTATAAGGCAATTCGTCATGGACAGTGCAGTGCAGCCCTAGTAGGAGG
2 canna_FASN      99.7%
TGCTCCTCTAGTCTCATTGCTCTGGAAAATGCTTATAAGGCAATTCGTCATGGACAGTGCAGTGCAGCCCTAGTAGGAGG
3 swift_FASN      90.8%
TGCTCCTCTAGTCTCATCGCTCTGGAAAATGCTTATAAGGCAATTCGTCATGAACAGTGCAGTGCAGCCCTAGTAGGAGG
4 human_FASN      64.1%
TGCTCCTCCAGCCTGATGGCCCTGCAGAACGCCTACCAGGCCATCCACAGCGGGCAGTGCCCTGCCGCCATCGTGGGGGG
5 gallus_FASN     88.9%
TGCTCCTCCAGTCTCATGGCTTTAGAAAATGCTTATAAAGCAATTCGTCACGGACAGTGCAGTGCAGCCCTGGTAGGAGG
6 alligator_FASN  75.2%
TGTTCCTCAAGCCTCCTGGCCCTGGAAGTTGCTTACAAAGCAATCTGTCATGGAGAGTGTGATGCAGCTCTTGTAGGAGG

                         561          .         .         .         6         .         .
.         . 640
1 ruby_FASN      100.0%
CGTCAACCTGTTGCTGAAACCCAACACTTCCGTGCAGTTCATGAAACTGGGTATGCTTAGTCCTGAAGGCGCCTGCAAGG
2 canna_FASN      99.7%
CGTCAACCTGTTGCTGAAACCCAACACTTCCGTGCAGTTCATGAAACTGGGTATGCTTAGTCCTGAAGGCGCCTGCAAGG
3 swift_FASN      90.8%
GGTCAACATTTTGCTGAAACCAAACACTTCTGTGCAGTTCATGAAACTGGGTATGCTTAGTCCTGATGGTGCCTGCAAGG
4 human_FASN      64.1%
CATCAATGTCCTGCTGAAGCCCAACACCTCCGTGCAGTTCTTGAGGCTGGGGATGCTCAGCCCCGAGGGCACCTGCAAGG
5 gallus_FASN     88.9%
GGTCAACATTCTGCTGAAGCCCAACACTTCTGTGCAGTTCATGAAGCTGGGCATGCTTAGTCCTGATGGTGCCTGCAAGG
6 alligator_FASN  75.2%
ATGCAGCATCATGCTGAAACCCAACACATCGTTGCAGTTTATGAAGCTGGGGATGCTCTCCCCTGATGGTACCTGCAAGG

                         641          :         .         .         .         .         7
.         . 720
1 ruby_FASN      100.0%
CTTTTGATGCTTCAGGAAATGGATATTGTCGCTCTGAAGCTGTTGTTATCGTTCTTCTGACCAAACGATCTATGGCTAAG
2 canna_FASN      99.7%
CTTTTGATGCTTCAGGAAATGGATATTGTCGCTCTGAAGCTGTTGTTATCGTTCTTCTGACCAAACGATCTATGGCTAAG
3 swift_FASN      90.8%
CTTTTGATGTTTCAGGAAATGGATATTGTCGCTCTGAAGCTGTTGTCGTTGTTCTCTTGACCAAGCGATCTATGGCCAAG
4 human_FASN      64.1%
CCTTCGACACAGCGGGGAATGGGTACTGCCGCTCGGAGGGTGTGGTGGCCGTCCTGCTGACCAAGAAGTCCCTGGCCCGG
5 gallus_FASN     88.9%
CTTTCGATGTTTCAGGAAATGGGTATTGTCGCTCTGAAGCTGTTGTTGTTGTGCTCTTGACCAAGAAATCCATGGCTAAA
6 alligator_FASN  75.2%
CCTTTGATGCTTCAGGAAATGGATATTGCCGCTCTGAAGGTGTCGTTGTGGTTTTGTTAACCAAGAAATCCATGGCCAAA

                         721          .         .         :         .         .         .
.         8 800
1 ruby_FASN      100.0%
CGGATCTATGCCACAATAGTTAATGCTGGAACTAACACTGATGGCTTTAAGGAGCAAGGTGTGACATTTCCATCTGGAGA
2 canna_FASN      99.7%
CGGATCTATGCCACAATAGTTAATGCTGGAACTAACACTGATGGCTTTAAGGAGCAAGGTGTGACATTTCCATCTGGAGA
3 swift_FASN      90.8%
CGGGTCTACGCCACGATAGTTAATGCTGGAAGCAACACCGACGGCTTTAAGGAGCAAGGTGTGACATTCCCATCTGGAGA
4 human_FASN      64.1%
CGGGTGTACGCCACCATCCTGAACGCCGGCACCAATACAGATGGCTTCAAGGAGCAAGGCGTGACCTTCCCCTCAGGGGA
5 gallus_FASN     88.9%
CGCGTCTATGCCACTATAGTCAATGCTGGGAGTAACACTGATGGCTTTAAGGAGCAAGGTGTGACATTCCCATCTGGAGA
6 alligator_FASN  75.2%
CGGGTTTATGCCACAATAGTCAATGCTGGGACTAACACTGATGGTTTTAAGGAACAAGGTCTAACATTTCCTTGTGGAGA

                         801          .         .         .         .         :         .
.         . 880
1 ruby_FASN      100.0%
GATGCAGCAGCAGTTGATCAGCTCTTTATACAGAGAAAGTGGTATCAGTCCTGAAGAAGTGGAATATGTAGAAGCTCATG
2 canna_FASN      99.7%
GATGCAGCAGCAGTTGATCAGCTCTTTATACAGAGAAAGTGGTATCAGTCCTGAAGAAGTGGAATATGTAGAAGCTCATG
3 swift_FASN      90.8%
GATGCAGCAGCAGTTGATCAGATCTGTGTACAGAGAATGTGGTGTCAGTCCTGGAGAAGTGGAGTACATTGAAGCTCATG
4 human_FASN      64.1%
TATCCAGGAGCAGCTCATCCGCTCGTTGTACCAGTCGGCCGGAGTGGCCCCTGAGTCATTTGAATACATCGAAGCCCACG
5 gallus_FASN     88.9%
GATGCAGCAGCAGCTGGTTGGTTCTCTGTACAGAGAATGTGGTATCAAGCCTGGAGATGTGGAGTATGTTGAAGCTCATG
6 alligator_FASN  75.2%
GATGCATCAGCGGTTGTTCAGCTCCCTGTATACAGCATCTGGGATTCTGCCTGACGAGATGGAGTATGTAGAAGCTCATG
```

```
                           881          .         9         .         .         .         .
:         . 960
1 ruby_FASN      100.0%
GGACAGGCACCAAGGCTGGAGATCCGCAGGAATTAAATAGCATTGTAAATGTCTTCTGTAAATGTGAGAGGGAACCACTG
2 canna_FASN      99.7%
GGACAGGCACCAAGGCTGGAGATCCGCAGGAATTAAATAGCATTGTAAATGTCTTCTGTAAATGTGAGAGGGAACCACTG
3 swift_FASN      90.8%
GGACAGGCACAAAGGCTGGAGATCCACAGGAAGTAAATAGCATTGTAAATTTCTTCTGTAAATGTGAGAGAGAGCCACTG
4 human_FASN      64.1%
GCACAGGCACCAAGGTGGGCGACCCCCAGGAGCTGAATGGCATCACCCGAGCCCTGTGCGCCACCCGCCAGGAGCCGCTG
5 gallus_FASN     88.9%
GGACAGGCACCAAGGTTGGAGATCCGCAAGAAGTAAATGGCATTGTAAATGTCTTCTGCCAGTGTGAGAGAGAGCCTCTG
6 alligator_FASN  75.2%
GCACAGGCACCAAGGCTGGGGATCCGCAAGAGGTGAATGCCATCGCAAAAGTCTTGTGTCCAGGCAGACGAGAGCCACTG

                           961          .         .         .         0         .         .
.         . 1040
1 ruby_FASN      100.0%
AAGATTGGATCCACCAAATCAAACATGGGTCATCCAGAGCCTGCTGCTGGGCTTGCTGCATTAGCCAAGGTCATTCTCTC
2 canna_FASN      99.7%
AAGATTGGATCCACCAAATCAAACATGGGTCATCCAGAGCCTGCTGCTGGGCTTGCTGCATTAGCCAAGGTCATTCTCTC
3 swift_FASN      90.8%
AAGATTGGATCAACAAAGTCAAACATGGGTCATCCAGAGCCTGCTTCTGGGCTTGCTGCATTAGCTAAGGTCATTCTCTC
4 human_FASN      64.1%
CTCATCGGCTCCACCAAGTCCAACATGGGGCACCCGGAGCCAGCCTCGGGGCTGGCAGCCCTGGCCAAGGTGCTGCTGTC
5 gallus_FASN     88.9%
TTAATTGGATCAACCAAGTCAAACATGGGTCATCCAGAGCCTGCCTCTGGGCTTGCTGCATTAGCCAAGGTCATTCTTTC
6 alligator_FASN  75.2%
TTGATTGGATCCACTAAATCCAACATGGGTCACCCCGAACCTGCTTCTGGGCTTGTTGCATTACTCAAGGTGATTCTCTC

                          1041          :         .         .         .         .         1
.         . 1120
1 ruby_FASN      100.0%
TCTGGAACATGGGCTGTGGGCTCCAAATCTTCATTTCAATACTCCGAATCCAGATATTCCTGGTTTACAAGATGGCTCTT
2 canna_FASN      99.7%
TCTGGAACATGGGCTGTGGGCTCCAAATCTTCATTTCAATACTCCGAATCCAGATATTCCTGGTTTACAAGATGGCTCTT
3 swift_FASN      90.8%
TCTGGAACATGGGCTGTGGGCTCCAAACCTTCATTTCAATACCCCAAATCCAGATATCCCTGCCTTACAAGATGGCACTT
4 human_FASN      64.1%
CCTGGAGCACGGGCTCTGGGCCCCCAACCTGCACTTCCATAGCCCCAACCCTGAGATCCCAGCGCTGTTGGATGGGCGGC
5 gallus_FASN     88.9%
TCTGGAACATGGACTGTGGGCTCCAAATCTTCATTTCAATGATCCAAATCCAGATATTCCTGCTTTACACGATGGCTCCT
6 alligator_FASN  75.2%
TCTGGAAAATGGGGTGTGGGCTCCAAACCTCCATTACAACACCCCGAACCCAGACATTCCTGCCTTACAAGATGGCAGCT

                          1121          .         .         :         .         .         .
.         2 1200
1 ruby_FASN      100.0%
TGGAGGTAATTTGTAAGCCAACACCGGTGAAAGGTGGCCTTGTCAGTATCAACTCTTTTGGCTTTGGTGGTGCTAATGCT
2 canna_FASN      99.7%
TGGAGGTAATTTGTAAGCCAACACCGGTGAAAGGTGGCCTTGTCAGTATCAACTCTTTTGGCTTTGGTGGTGCTAATGCT
3 swift_FASN      90.8%
TGGAGGTAGTTTGTAAACCAACACCAGTGAAAGGTGGCCTTGTCAGTATCAACTCTTTTGGCTTTGGAGGTGCTAATGCT
4 human_FASN      64.1%
TGCAGGTGGTGGACCAGCCCCTGCCCGTCCGTGGCGGCAACGTGGGCATCAACTCCTTTGGCTTCGGGGGCTCCAACGTG
5 gallus_FASN     88.9%
TGAAGGTGGTTTGCAAACCAACACCGGTGAAAGGTGGCCTTGTCAGCATCAATTCTTTTGGCTTTGGAGGCTCTAATGCT
6 alligator_FASN  75.2%
TACAGGTGGTCTGTAAGCCGACCCCAGTGAAAGGGGGCCTAGTCGGTATCAATTCTTTTGGCTTTGGTGGTTCCAATGTC

                          1201          .         .         .         .         :         .
.         . 1280
1 ruby_FASN      100.0%      CACGTCATTCTGAGGCCAAATGA--
-GAACAGACGCCAGCCTCTGGAG-ACTTGTAACA-TACCAAGATTGGTTCAAGTT
2 canna_FASN      99.7%      CACGTCATTCTGAGGCCAAATGA--
-GAACAGACGCCAGCCTCCGGAG-ACTTGTAACA-TACCAAGATTGGTTCAAGTT
3 swift_FASN      90.8%      CATGTCATTCTGAGGCCAAATGA---GAACAAACGCCAGCCTCTGGAG-ACATGTAACA-
CACCAAGACTGGTTCAAGTT
4 human_FASN      64.1%      CACATCATCCTGAGGCCCAACAC--
GCAGCCGCCCCCCGCACCCGCCCCACATGCCACCCTGCCCCGTCTGCTGCGGGCC
5 gallus_FASN     88.9%      CATGTTATTCTGAGGCCAAATGA--
-GAAGAAATGTCAGCCTCAAGAG-ACTTGTAACT-TGCCAAGACTGGTTCAAGTT
6 alligator_FASN  75.2%      CATGTCATCCTGAGGCCACATGACAAGAAGAAATCCCAGCCTCTGGAG-
GCACGTTGTT-TGCCACGACTGGTCCAAGTC

                          1281          .         3         .         .         .         .
:         . 1360
1 ruby_FASN      100.0%
TGCGGCAGAACCCAGGAAGCTGTGGAAATACTAATTCAAGAGAGCAGAAAACATGGAGGATGCAGCCCATTTGTAAGCCT
2 canna_FASN      99.7%
TGCGGCAGAACCCAGGAAGCTGTGGAAGTACTAATTCAAGAGAGCAGAAAACATGGAGGATGCAGCCCATTTGTAAGCCT
```

```
3 swift_FASN      90.8%
TGTGGTAGAACCCAGGAAGCTGTGGAAAAACTAATTCAAGAGAGCAGAAGACATGGAGGATGCAGCCCGTTTGTAAGCCT
4 human_FASN      64.1%
AGCGGACGCACCCCTGAGGCCGTGCAGAAGCTGCTGGAGCAGGGCCTCCGGCACAGCCAGGACCTGGCTTTCCTGAGCAT
5 gallus_FASN     88.9%
TGTGGCAGAACACAGGAAGCTGTGGAAATACTAATTGAAGAAAGCAGGAAACATGGAGGATGCAGTCCATTTTTAAGCCT
6 alligator_FASN  75.2%
TTTGGCAGGACGCAAGAAGCTGTGGAGAAGCTACTGGACCAGAGCAAGAGACTGGGGGAAGATGACTCATTTGTGAGCCT

                         1361          .         .         .         4         .         .
.         . 1440
1 ruby_FASN      100.0%
GCTCAGTGATATCTCTGCAATGCCCGTATCCTCCATGCCCTACAGGGGCTACACACTGGTTGGCACTGAGAGTGACATAC
2 canna_FASN      99.7%
GCTCAGTGATATCTCTGCAATGCCCGTATCCTCCATGCCCTACAGGGGCTACACACTGGTTGGCACTGAGAGTGACATAC
3 swift_FASN      90.8%
GCTCAGTGATATCTCTGCAATTTCTGTATCCTCCATGCCTTACAGGGGTTACACACTGGTTGGCACTGAGAGTGACATAA
4 human_FASN      64.1%
GCTGAACGACATCGCGGCTGTCCCCGCCACCGCCATGCCCTTCCGTGGCTACGCTGTGCTGGGTGGTGAGCGCGGTGGCC
5 gallus_FASN     88.9%
GCTCAGTGATATCTCTGCAGTTCCTGTATCTTCCATGCCCTACAGGGGCTACACACTAGTTGGCACTGAGAGTGACATAA
6 alligator_FASN  75.2%
ACTCAACGATTTGTCCGCAATCCCCGCTTCCTCTATGCCGTACCGGGGCTACAGGCTCGTTGGCAGTGAGAGTGACGTGA

                         1441          :         .         .         .         .         5
.         . 1520
1 ruby_FASN      100.0%
AAGAGATTCAGCAAGTTCAAGCGTCTGGTAGACCACTATGGTACATCTGTTCAGGCATGGGAACACAGTGGAAAGGTATG
2 canna_FASN      99.7%
AAGAGATTCAGCAAGTTCAAGCGTCTGGTAGACCACTATGGTACATCTGTTCAGGCATGGGAACACAGTGGAAAGGTATG
3 swift_FASN      90.8%
AAGAGGTTCAGCAGGTTCAAGCATCTGGTAGACCACTCTGGTACATCTGCTCAGGCATGGGAACACAGTGGCAAGGCATG
4 human_FASN      64.1%
CAGAGGTGCAGCAGGTGCCCGCTGGCGAGCGCCCGCTCTGGTTCATCTGCTCTGGGATGGGCACACAGTGGCGCGGGATG
5 gallus_FASN     88.9%
CAGAGATTCAGCAAGTTCAAGCATCTGGTAGACCACTCTGGTACATCTGCTCAGGCATGGGAACACAGTGGAAAGGTATG
6 alligator_FASN  75.2%
AAGAGGTGCAGCAAATCCAGGCATCGGGGAGGCCCCTCTGGTACATCTGCACAGGCATGGGCACGCAGTGGGCAGGGATG

                         1521          .         .         :         .         .         .
.         6 1600
1 ruby_FASN      100.0%
GGCCTGAGCCTTATGAAGTTGGATTTGTTTCGCCAGTCTATACTGCGCTCAGACCAGGCTTTAAAGAACACAGGTCTGAA
2 canna_FASN      99.7%
GGCCTGAGCCTTATGAAGTTGGATTTGTTTCGCCAGTCTATACTGCGCTCAGACCAGGCTTTAAAGAACACAGGTCTGAA
3 swift_FASN      90.8%
GGGCTAAGCCTTATGAAGTTGGATTTGTTCCGCCAATCTATACTGCGCTCAGATGAGGCTTTGAAGAGCACACGCCTGAA
4 human_FASN      64.1%
GGGCTGAGCCTCATGCGCCTGGACCGCTTCCGAGATTCCATCCTACGCTCCGATGAGGCTGTGAAGCCATTCGGCCTGAA
5 gallus_FASN     88.9%
GGCCTGAGCCTTATGAAATTGGATCTGTTTCGCCAGTCTATATTGCGCTCAGATGAGGCTTTGAAGAGCACAGGACTGAA
6 alligator_FASN  75.2%
GGCAGGAGCCTGATGCAGCTGGAGCTGTTCCGCCAGTCCATCTTGCGCTCGGATGAGATCCTGAAGGGCACTGGGCTGAA

                         1601          .         .         .         .         :         .
.         . 1680
1 ruby_FASN      100.0%
GGTCTCAGACCTGCTTCTGCAAGCAGATGACAATACTTTTGATGAGACTGTCCATGCATTTGTTGGACTAGCTGCTATTC
2 canna_FASN      99.7%
GGTCTCAGACCTGCTTCTGCAAGCAGATGACAATACTTTTGATGAGACTGTCCATGCATTTGTTGGACTAGCTGCTATTC
3 swift_FASN      90.8%
GGTCTCAGACTTGCTTCTGCAAGCAGATGAGAACACTTTTGATGATATTGTCCATGCGTTTGTTGGACTAGCTGCAATTC
4 human_FASN      64.1%
GGTGTCACAGCTGCTGCTGAGCACAGACGAGAGCACCTTTGATGACATCGTCCATTCGTTTGTGAGCCTGACTGCCATCC
5 gallus_FASN     88.9%
GGTCTCAGACCTGCTTCTGAATGCAGATGAGAACACTTTTGATGACACTGTCCATGCTTTTGTTGGACTAGCTGCTATAC
6 alligator_FASN  75.2%
GGTCTCTGACATCATTCTGAATGGTGATGAGAGCATGTTTGATGATGCTGTCTGCTCATTTGTTGGAGTCTCTGCAGTTC

                         1681          .         7         .         .         .         .
:         . 1760
1 ruby_FASN      100.0%
AGATTGCCCAGATTGATATGCTGAAGGCTGCAGGTCTGCAACCTGATGGGATTTTGGGCCATTCTGTGGGTGAATTAGCT
2 canna_FASN      99.7%
AGATTGCCCAGATTGATATGCTGAAGGCTGCAGGTCTGCAACCTGATGGGATTTTGGGCCATTCTGTGGGTGAATTAGCT
3 swift_FASN      90.8%
AGATTGCCCAGATTGATATCTTGAAGGCTGCAGGTCTGCAGCCTGATGGGATTTTGGGCCACTCAGTGGGAGAAGTAGCT
4 human_FASN      64.1%
AGATAGGCCTCATAGACCTGCTGAGCTGCATGGGGCTGAGGCCAGATGGCATCGTCGGCCACTCCCTGGGGGAGGTGGCC
5 gallus_FASN     88.9%
AGATTGCCCAAATTGATGTGCTAAAGGCTGCGGGTCTGCAACCTGATGGGATTTTGGGCCACTCAGTGGGAGAACTAGCT
```

```
6 alligator_FASN  75.2%
AGGTGGCACAGATCGACATGTTGAAGTCTATGGGCCTCCAGCCCGATGGGATCATCGGGCACTCGGTGGGAGAGGTGGCC

                        1761           .         .         .         8         .         .
.         . 1840
1 ruby_FASN      100.0%
TGTGGCTATGCAGATAACTGCTTAAGTCATGAAGAAGCCATTCTTGCTGCCTATTGGCGGGGAAGATGTGTTAAAGAGGC
2 canna_FASN      99.7%
TGTGGCTATGCAGATAACTGCTTAAGTCATGAAGAAGCCATTCTTGCTGCCTATTGGCGGGGAAGATGTGTTAAAGAGGC
3 swift_FASN      90.8%
TGTGGATATGCAGATAACTCCTTAACTCATGAAGAAGCCATTCTTGCCGCTTATTGGCGGGGACAATGTGTTAAGGAGAA
4 human_FASN      64.1%
TGTGGCTACGCCGACGGCTGCCTGTCCCAGGAGGAGGCCGTCCTCGCTGCCTACTGGAGGGGACAGTGCATCAAAGAAGC
5 gallus_FASN     88.9%
TGTGGCTATGCAGATAATTCCTTAAGTCATGAAGAAGCTGTTCTTGCTGCTTATTGGAGGGGCCGATGTGTGAAAGAGGC
6 alligator_FASN  75.2%
TGTGGCTATGCCGACGACTCCTTCAGTCACGAAGAAGCCATTCTCTCTGCCTATTGGAGGGGACGGTGCGTCAAAGAGGC

                        1841           :         .         .         .         .         9
.         . 1920
1 ruby_FASN      100.0%
AAAATTGCCCCCAGGAGGAATGGCTGCTGTTGGTCTGACATGGGAGGAATGTAAGCAACAGTGTCCTCCAAATGTGGTAC
2 canna_FASN      99.7%
AAAATTGCCCCCAGGAGGAATGGCTGCTGTTGGTCTGACATGGGAGGAATGTAAGCAACAGTGTCCTCCAAATGTGGTAC
3 swift_FASN      90.8%
CAAATTGCCCCCAGGAGGAATGGCTGCTGTTGGTCTGACATGGGAGGAATGTAAGCAACAGTGTCCTCCAAATGTGGTAC
4 human_FASN      64.1%
CCATCTCCCGCCGGGCGCCATGGCAGCCGTGGGCTTGTCCTGGGAGGAGTGTAAACAGCGCTGCCCCCCGGGCGTGGTGC
5 gallus_FASN     88.9%
CAAATTGCCCCCGGGAGGGATGGCTGCTGTTGGTCTGACATGGGAGGAATGTAAGCAGCGCTGTCCTCCAAACGTGGTAC
6 alligator_FASN  75.2%
GAATCTACCCCGAGGAAAGATGGCTGCTGTTGGTCTGTCGTGGGAGGAATGTAAGATCCGCTGCCCTCCAGGCGTGGTGC

                        1921           .         .         :         .         .         .
.        0 2000
1 ruby_FASN      100.0%
CAGCCTGTCACAACTCTGAAGACACTGTCACTATTTCTGGGCCTCTGGCCACCGTGAGTGAGTTTGTAGCCAAACTGAAA
2 canna_FASN      99.7%
CAGCCTGTCACAACTCTGAAGACACTGTCACTATTTCTGGACCTCTGGCCACCGTGAGTGAGTTTGTAGCCAAACTGAAA
3 swift_FASN      90.8%
CAGCCTGTCACAACTCTGAAGATACTGTCACTGTTTCTGGGACTCTGGCCTCTGTGAATGAGTTTGTAGCCAAGCTGAAA
4 human_FASN      64.1%
CCGCCTGCCACAACTCCAAGGACACAGTCACCATCTCGGGACCTCAGGCCCCGGTGTTTGAGTTCGTGGAGCAGCTGAGG
5 gallus_FASN     88.9%
CAGCATGTCACAACTCTGAGGATACTGTCACTGTTTCGGGGCCTCTGGATTCTGTGTCTGAGTTTGTAACCAAACTGAAG
6 alligator_FASN  75.2%
CTGCCTGTCACAACGCTGAGGACACTGTCACTATCTCGGGGCCAGAGGCCACCATGAATGAGTTCCTAGCCACCCTGAAG

                        2001           .         .         .         .         :         .
.         . 2080
1 ruby_FASN      100.0%
AAGGCTGGTGTTTTCGCAAAGGAGGTGCGCAGTGCTGGAGTTGCGTTTCATTCCCATTATATGGCATCTATTGCACCAGT
2 canna_FASN      99.7%
AAGGCTGGTGTTTTCGCAAAGGAGGTGCGCAGTGCTGGAGTTGCGTTTCATTCCCATTATATGGCATCTATTGCACCAGT
3 swift_FASN      90.8%
AAGGATGGTGTGTTTGCCAAGGAGGTGCGCAGTGCTGGAGTGGCATTTCATTCCCCTTACATGGCATCTATTGCACCAGG
4 human_FASN      64.1%
AAGGAGGGTGTGTTTGCCAAGGAGGTGCGGACCGGCGGTATGGCCTTCCACTCCTACTTCATGGAGGCCATCGCACCCCC
5 gallus_FASN     88.9%
AAAGATGGGGTGTTTGCAAAGGAGGTGCGCAGCGCCGGAGTTGCATTTCATTCCTATTACATGGCATCCATTGCACCAGC
6 alligator_FASN  75.2%
AAGGAAGGTGTGTTTGCTAAGGAAGTGCGCAGTGTCGGGGTCGCATTTCATTCCTATTTCATGGAGGCGCTTGCCCCAAT

                        2081           .         1         .         .         .         .
:         . 2160
1 ruby_FASN      100.0%
GCTGCTCAGTGCCCTGAAAAAGGTAATTCCACACCCTAAACCACGTTCAGCACGATGGATCAGTACATCTATCCCTGAAT
2 canna_FASN      99.7%
GCTGCTCAGTGCCTTGAAAAAGGTAATTCCACACCCTAAACCACGTTCAGCACGATGGATCAGTACATCTATCCCTGAAT
3 swift_FASN      90.8%
GCTGCTCAGTGCCCTGAAAAAGGTAATTCCACATCCTAAACCTCGTTCAGCACGGTGGATCAGTACATCTATCCCTGAAA
4 human_FASN      64.1%
ACTGCTGCAGGAGCTCAAGAAGGTGATCCGGGAGCCGAAGCCACGTTCAGCCCGCTGGCTCAGCACCTCTATCCCCGAGG
5 gallus_FASN     88.9%
ACTACTCAGTGCACTGAAAAAGGTCATTCCACACCCTAAGCCTCGTTCAGCACGGTGGATCAGTACATCTATCCCTGAAT
6 alligator_FASN  75.2%
GTTGCTTAGTGTCCTGCGGAAGATCATTCCAAACCCGAAGCCTCGCTCGGCTCGTTGGATCAGTACATCCATCCCCGAGG

                        2161           .         .         .         2         .         .
.         . 2240
1 ruby_FASN      100.0%
```

```
CTCAGTGGCAGAGTGATCTAGCTAAGTACTCCTCTGCAGAGTATCATGTGAACAACCTAGTGAGTCCAGTGCTGTTCCAC
2 canna_FASN       99.7%
CTCAGTGGCAGAGTGATCTAGCTAAGTACTCCTCTGCAGAGTATCATGTGAACAACCTAGTGAGTCCAGTGCTGTTCCAC
3 swift_FASN       90.8%
ATCAGTGGCAGAGTGATCTAGCTAAGAATTCCTCTGCTGAATATTACGTCAACAACCTAGTGAGTCCCGTGCTGTTCCAT
4 human_FASN       64.1%
CCCAGTGGCACAGCAGCCTGGCACGCACGTCCTCCGCCGAGTACAATGTCAACAACCTGGTGAGCCCTGTGCTGTTCCAG
5 gallus_FASN      88.9%
CTCAGTGGCAGAGTGATCTTGCTAGGAATTCCTCTGCAGAGTATCATGTGAACAACCTAGTGAATCCTGTGCTGTTCCAT
6 alligator_FASN  75.2%
CCCAGTGGGGCAGTGAGCTGGCGCGCTACTCCTCGGCGGAGTATCACGTCAACAACCTGAGGAGCCCGGTGCTGTTCCAG

                        2241          :         .         .         .         .         3
.         . 2320
1 ruby_FASN      100.0%
GAAGGTCTGAAGCATATTCCAGAGAATGCTGTTGTAGTGGAGATTGCTCCACATGCTCTTTTACAGGCTATCTTGAAGAG
2 canna_FASN       99.7%
GAAGGTCTGAAGCATATTCCAGAGAATGCTGTTGTAGTGGAGATTGCTCCACATGCTCTTTTACAGGCTATCTTGAAGAG
3 swift_FASN       90.8%
GAAGGTCTGAAGCATATTCCAGAAAATGCCGTTGTAGTAGAGATTGCTCCACATGCTCTTCTACAGGGTGTCCTGAGGAG
4 human_FASN       64.1%
GAGGCCCTGTGGCACGTGCCTGAGCACGCGGTGGTGCTGGAGATCGCGCCCCACGCCCTGCTGCAGGCTGTCCTGAAGCG
5 gallus_FASN      88.9%
GAAGGCCTGAAGCATATTCCAGAGAATGCTGTTGTAGTGGAGATTGCTCCACATGCTCTCTTACAGGCTATCTTGAGGAG
6 alligator_FASN  75.2%
GAGGGTCTGCAGCACGTTCCGGACAACGCAGTGGTGGTGGAGATCGCGCCCCATGCCCTGCTGCAGGCTATCCTGAAGAG

                        2321          .         .         :         .         .         .
.         4 2400
1 ruby_FASN      100.0%
AACTTTGAAACCAACTTGCACCATTCTACCTTTGATGAAGAAAGACCACAAAAATAATTTGGAGTTCTTCCTGACACAGG
2 canna_FASN       99.7%
AACTTTGAAACCAACTTGCACCATTCTACCTTTGATGAAGAAAGACCACAAAAATAATTTGGAGTTCTTCCTGACACAGG
3 swift_FASN       90.8%
AGCCTTGAAACCAACTTGCACCATTCTGCCTTTGATGAAGAAAGAGCACAAAAACAATTTGGAGTTCTTCCTGACACAGA
4 human_FASN       64.1%
TGGCCTGAAGCCGAGCTGCACCATCATCCCCCTGATGAAGAAGGATCACAGGGACAACCTGGAGTTCTTCCTGGCCGGCA
5 gallus_FASN      88.9%
AACTTTGAAGCCAACTTGCACTATTCTACCTCTGATGAAGAAGGACCACAAAAATAACTTGGAGTTCTTCCTAACGCAGA
6 alligator_FASN  75.2%
AAGCTTGAAACCAACCTGCACCATCCTGCCTTTGATGAAGAGAGAGCACAAAAACAACTTGGAGTTCTTCCTGACGCACA

                        2401          .         .         .         .         :         .
.         . 2480
1 ruby_FASN      100.0%
CTGGAAAGATTCACTTAACTGGGATAAATGTTCTTGGAAATAACTTGTTCCCACCTGTGGAATACCCTGTTCCAGTGGGA
2 canna_FASN       99.7%
CTGGAAAGATTCACTTAACTGGGATAAATGTTCTTGGAAATAACTTGTTCCCACCTGTGGAATACCCTGTTCCAGTGGGA
3 swift_FASN       90.8%
CTGGAAAGATTCACTTAACTGGGATAAATGTTCTTGGAAATAACTTGTTCCCACCTGTGGAATACCCTGTTCCAGTGGGA
4 human_FASN       64.1%
TCGGCAGGCTGCACCTCTCAGGCATCGACGCCAACCCCAATGCCTTGTTCCCACCTGTGGAGTTCCCAGCTCCCCGAGGA
5 gallus_FASN      88.9%
CTGGAAAGATTCATTTAACTGGGATAAATGTTCTTGGAAATAACTTGTTCCCACCTGTGGAATACCCTGTCCCTGTGGGA
6 alligator_FASN  75.2%
TTGGAAAGATCTACTTGACTGGAATAAATGTTCAGTCAAATAAGCTGTTCCCACCTGTTGAATATCCTGTGCCAGTAGGA

                        2481          .         5         .         .         .         .
:         . 2560
1 ruby_FASN      100.0%
ACACCCCTTATTTCTCCATACATCAAATGGGACCACAGTCAGGGCTGGGATGTTCCAAAAGCTGAAGACTTCCCTTCAGG
2 canna_FASN       99.7%
ACACCCCTTATTTCTCCATACATCAAATGGGACCACAGTCAGGGCTGGGATGTTCCAAAAGCTGAAGACTTCCCTTCAGG
3 swift_FASN       90.8%
ACACCCCTTATTTCTCCATACATCAAATGGGACCACAGCCAGGACTGGGATGTTCCGAAAGCTGGAGACTTCCCTTCGGG
4 human_FASN       64.1%
ACTCCCCTCATCTCCCCACTCATCAAGTGGGACCACAGCCTGGCCTGGGACGTGCCGGCCGCCGAGGACTTCCCCAACGG
5 gallus_FASN      88.9%
ACACCTCTCATTTCTCCATATATCAAATGGGACCACAGCCAAGACTGGGATGTTCCAAAAGCTGAAGACTTCCCCTCAGG
6 alligator_FASN  75.2%
ACCCCACTCATCTCTCCCCACATCCTGTGGGATCACAGTGAGACCTGGTACTACCCAAAAGCCGAAGAATTTCCATCTGG

                        2561          .         .         .         6         .         .
.         . 2640
1 ruby_FASN      100.0%
TTCCAAAGGCTCTGCATCTGCTTCAATCTACAATATTGATGTGAGTCCTGACTCTCCTGACCACTACTTGGTTGGTCACT
2 canna_FASN       99.7%
TTCCAAAGGCTCTGCATCTGCTTCAATCTACAATATTGATGTGAGTCCTGACTCTCCTGACCACTACTTGGTTGGTCACT
3 swift_FASN       90.8%
TTCCAGAGGCTCTGCATCTGCTTCCATCTACAATATTGATGTGAGTCCTGACTCTCCTGACCACTACTTGGTTGGTCACT
4 human_FASN       64.1%      TTC--
```

```
-AGGTTCCCCCTCAGCCGCCATCTACAACATCGACACCAGCTCCGAGTCTCCTGACCACTACCTGGTGGACCACA
5 gallus_FASN     88.9%
TTCCAAAGGCTCTGCGTCTGCTTCAGTCTACAACATCGATGTGAGTCCTGACTCTCCTGACCATTACTTGGTTGGCCATT
6 alligator_FASN  75.2%
CTCTGCAGGCTCTTCATCTGCTTCGGTCTACAATATTGATGTGAACCCCGAGTCTCCTGACCATTACTTGATTGGTCACT

                        2641          :         .         .         .         .         7
.           . 2720
1 ruby_FASN      100.0%
GCATTGACGGCAGAGTCCTCTACCCAGCAACTGGGTACCTAGTGCTGGCTTGGCGGACTCTGGCACGGTCCCTTGGCATG
2 canna_FASN      99.7%
GCATTGATGGCAGAGTCCTCTACCCAGCAACTGGGTACCTAGTGCTGGCTTGGCGGACTCTGGCACGGTCCCTTGGCATG
3 swift_FASN      90.8%
GCATTGATGGCAGAGTCCTGTACCCAGCAACTGGGTACCTGGTGCTGGCTTGGCGAACCCTGGCACGGTCTCTTGGCATG
4 human_FASN      64.1%
CCCTCGACGGTCGCGTCCTCTTCCCCGCCACTGGCTACCTGAGCATAGTGTGGAAGACGCTGGCCCGCGCCCTGGGCCTG
5 gallus_FASN     88.9%
GCATTGATGGCAGAGTCCTGTACCCAGCAACTGGGTACTTAGTGCTGGCGTGGCGAACTCTGGCACGATCTCTTGGCATG
6 alligator_FASN  75.2%
GCGTTGATGGCAGAGTCCTCTACCCAGCCACTGGGTACCTGGTGCTAGCCTGGCGAACCCTGGCACGGTCTCTGGGAGCT

                        2721          .         .         :         .         .         .
.          8 2800
1 ruby_FASN      100.0%
GTTATGGAGCAAACAGCTGTTAAGTTTGAAGATGTCACAATTCATCAGGCAACTATACTTCCCAAAAATGGATCAGTACA
2 canna_FASN      99.7%
GTTATGGAGCAAACAGCTGTTAAGTTTGAAGATGTCACAATTCATCAGGCAACTATACTTCCCAAAAATGGATCAGTACA
3 swift_FASN      90.8%
ACTATGGAGAAAACAGCTGTGAAGTTTGAAGATGTCACAATTCATCAAGCAACCATCCTTCCCAAAAAGGGATCAGTACA
4 human_FASN      64.1%
GGCGTCGAGCAGCTGCCTGTGGTGTTTGAGGATGTGGTGCTGCACCAGGCCACCATCCTGCCCAAGACTGGGACAGTGTC
5 gallus_FASN     88.9%
GTCATGGAACAAACAGCTGTTATGTTTGAAGAAGTTACAATCCATCAGGCAACTATCCTTCCCAAAAAGGGATCAACACA
6 alligator_FASN  75.2%
ATCATGGAACAAATGCCTGTCAAGTTTGAGGATGTTGAGATCCACCAGGCAACCATCCTACCCAAGAAAGGTTCTGTGCA

                        2801          .         .         .         .         :         .
.           . 2880
1 ruby_FASN      100.0%
GCTGGAAGTAAGACTTATGCCTGCTTCCCACAGCTTTGAGGTGTCAGGCAATGGGAATCTGGCTGTGAGTGGGAAGATTT
2 canna_FASN      99.7%
GCTGGAAGTAAGACTTATGCCTGCTTCCCACAGCTTTGAGGTGTCAGGCAATGGGAATCTGGCTGTGAGTGGGAAGATTT
3 swift_FASN      90.8%
GCTGGAAGTGAGACTCATGCCTGCTTCCCACTGCTTTGAGGTGTCAGGGAATGGAAATCTGGCTGTGAGTGGGAAGATTT
4 human_FASN      64.1%
CCTGGAGGTACGGCTCCTGGAGGCCTCCCGTGCCTTCGAGGTGTCAGAGAACGGCAACCTGGTAGTGAGTGGGAAGGTGT
5 gallus_FASN     88.9%
GCTGGAAGTACGAATCATGCCTGCTTCTCACAGCTTTGAAGTGTCAGGGAATGGGAATTTGGCTGTGAGTGGGAAGATCT
6 alligator_FASN  75.2%
GCTGGAGGTGAGACTCATGCCTGCTTCCCACAGCTTTGAAGTGTCAGGAAATGGAAACCTGAGTGTAAGTGGGAAAATTT

                        2881          .         9         .         .         .         .
:           . 2960
1 ruby_FASN      100.0%      CCCTCCTAGAAAACACTGCTCTGAAG--------------
-CCAGCTGACTTTCACACTCGAGCAGGCATGGGCTTAAAG
2 canna_FASN      99.7%      CCCTCCTAGAAAACACTGCTCTGAAG--------------
-CCAGCTGACTTTCACACTCGAGCAGGCATGGGCTTAAAG
3 swift_FASN      90.8%
CCCTCCTAGAAAACACTGCTCAGAAGAACTTCCATAACCAGTCAGTTGACTTCCAGACTCAAGTAGACAAGAGCTCAAAG
4 human_FASN      64.1%      ACCAGTGGGATGACCCTGACCCCAGGCTCTTCGACCA---
-CCCGGAAAGCCCCACCCCCAACCCCACGGAGCCCCTCTT
5 gallus_FASN     88.9%
CCCTCCTAGAAAACGATGCTCTGAAGAACTTTCATAACCAGCTGGCTGACTTTCAGAGTCAAGCAAACGTGACTGCGAAG
6 alligator_FASN  75.2%
ACCTTTTGGAAGACACTGCTCTGAATAACTTCCATAATGAGCAAGCTGATTTTGATGCCCAGCCAGAAGCAAGCTCCAGC

                        2961          .         .         .         0         .         .
.           . 3040
1 ruby_FASN      100.0%
CCTGGCCTTTTGAAAGAAGACATTTACCAAGAGCTGCATTTACGTGGATATAATTATGGACCAACTTTCCAGGGCGTTCT
2 canna_FASN      99.7%
CCTGGCCTCTTGAAAGAAGACATTTACCAAGAGCTGCATTTACGTGGATATAATTATGGACCAACTTTCCAGGGCGTTCT
3 swift_FASN      90.8%
CCTGGCCTCTTGAAAGAAGACATTTATCAAGAGCTGCAGCTGCGTGGCTATAATTATGGACCAACTTTCCAGGGTGTTTT
4 human_FASN      64.1%      CCTGGCCC----
-AGGCTGAAGTTTACAAGGAGCTGCGTCTGCGTGGCTACGACTACGGCCCTCATTTCCAGGGCATCCT
5 gallus_FASN     88.9%
TCTGGCCTCTTGATGGAAGATGTTTACCAAGAGCTGCATCTTCGTGGATATAACTATGGACCAACTTTTCAGGGTGTTCT
6 alligator_FASN  75.2%
CACCCCCTCTCCAAGACGGACATTTACACCGAGCTGCATCTCCGTGGGTACAACTATGGGCCAACGTTCCAGGGGCTCCT
```

```
                           3041         :         .         .         .         .         1
  .        . 3120
 1 ruby_FASN      100.0%
 GGAATGCAACAGTGAAGCTAGCGCAGCCAAAGTTCTGTGGAACGGGAACTGGGTGACCTTCCTTGACACCCTGCTACACG
 2 canna_FASN      99.7%
 GGAATGCAACAGTGAAGCTAGCGCAGCCAAGGTTCTGTGGAACGGGAACTGGGTGACCTTCCTTGACACCCTGCTACACG
 3 swift_FASN      90.8%
 GGAATGCAACAGTGAAGGAAACACAGGGAAAGTCCTGTGGAATGGGAATTGGGTGACCTTCCTTGACACCCTGCTACACG
 4 human_FASN      64.1%
 GGAGGCCAGCCTGGAAGGTGACTCGGGGAGGCTGCTGTGGAAGGATAACTGGGTGAGCTTCATGGACACCATGCTGCAGA
 5 gallus_FASN     88.9%
 GGAATGCAACAGTGAAGGAAGTGCAGGGAAAATTCTGTGGAATGGAAACTGGGTAACCTTCCTTGACACCCTGCTACACT
 6 alligator_FASN  75.2%
 CGAATGCAGCAGTTCAGGGAGCCATGGCAAGCTCCTGTGGAATGGGAACTGGGTGACGTTCCTCGACACCATGCTGCACG

                           3121         .         .         :         .         .         .
  .       2 3200
 1 ruby_FASN      100.0%
 TAATAATCTTGTCTGACACTGGGCGCAGTTTACGCTTACCCACCAGGATTCGTTCCGTGTGTGTTGACCCAGTGCTGCAT
 2 canna_FASN      99.7%
 TAATAATCTTGTCTGACACTGGGCGCAGTTTACGCTTACCCACCAGGATTCGTTCCGTGTGTGTTGACCCAGTGCTGCAT
 3 swift_FASN      90.8%
 TGATAATACTGTCTGAGACCGGGCGCAGTCTGCGCTTACCCACCAGGATTCGCTCAGTATCTATTGACCCAGTGCTGCAT
 4 human_FASN      64.1%
 TGTCCATCCTGGGCTCGGCCAAGCACGGCCTGTACCTGCCCACCCGTGTCACCGCCATCCACATCGACCCTGCCACCCAC
 5 gallus_FASN     88.9%
 TGATAGTCTTAGCAGAGACTGGGCGCAGTCTACGATTGCCCACCAGGATTCGCTCAGTGTATATTGACCCTGTGCTTCAT
 6 alligator_FASN  75.2%
 TGACTGTCCTGGGCTATCCTGGGCGCAGCCTGCGCCTACCCACACGCATCCGCTCAGTGTGCGTTGACCCCGTGCTGCAC

                           3201         .         .         .         .         :         .
  .        . 3280
 1 ruby_FASN      100.0%
 CAGGAGATGGTGTACCAGTATCAGGACAATGTAGAAGTTTGTGATGCTGTTGTCGACCTCTGTCTTGATCGCCTTCAAGC
 2 canna_FASN      99.7%
 CAGGAGATGGTGTGCCAGTATCAGGACAATGTAGAAGTTTGTGATGCTGTTGTCGACCTCTGTCTTGATCGCCTTCAAGC
 3 swift_FASN      90.8%
 CAAGAGCAGGTGTGCCAGTACCAGGACAATGTAGAAGCTTTTGATGTTGTTGTGGACCGCTGTCTTGATAACCTTAAAGC
 4 human_FASN      64.1%
 AGGCAGAAGCTGTACACACTGCAGGACAAGGCCCAAGTGGCTGACGTGGTGGTGAGCAGGTGGCTGAGGGTCACAGTGGC
 5 gallus_FASN     88.9%
 CAGGAGCAGGTGTACCAGTACCAGGACAATGTAGAAGCTTTTGATGTTGTTGTTGACCGCTGTCTTGATAGCCTCAAAGC
 6 alligator_FASN  75.2%
 CTGAAGCAAGTGCAGACGTACAAGGATGACAAACAAGCTTTTGATGTGTTCTATGACCACTGCCTTGGCAGCATCAGGTC

                           3281         .         3         .         .         .         .
  :        . 3360
 1 ruby_FASN      100.0%
 AGGGGGTGTTCAGCTCGATGGTCTTCATGCTTCTGTGGCACCACGACGACAACAGGAACGGATGACTCCTACCCTGGAAA
 2 canna_FASN      99.7%
 AGGGGGTGTTCAGCTCGATGGTCTTCATGCTTCTGTGGCACCACGACGACAACAGGAACGGATGACTCCTACCCTGGAAA
 3 swift_FASN      90.8%
 AGGAGGTGTTCAAATCGATGGTCTTCATGCTTCTGTGGCACCACGGCGGCAGCAGGAACGCAACTCTCCCACCCTGGAAA
 4 human_FASN      64.1%
 CGGAGGCGTCCACATCTCCGGGCTCCACACTGAGTCGGCCCCGCGGCGGCAGCAGGAGCAGCAGGTGCCCATCCTGGAGA
 5 gallus_FASN     88.9%
 AGGAGGTGTTCAGATCAATGGACTTCATGCCTCGGTGGCACCACGGCGACAACAGGAGCGGATCTCTCCCACTCTGGAAA
 6 alligator_FASN  75.2%
 AGGAGGTGTGCAGGTTGCTGGGCTCCACAGCTCTACAGCGCCCCGCAGGCAGCAGGAACAGGGTTCTCCACTCCTAGAGA

                           3361         .         .         .         4         .         .
  .        . 3440
 1 ruby_FASN      100.0%
 AGTTCTGCTTTGTGCCCTATACTGAGAGTGACTGTTTGTCTTCCAATGCCCATCTGCATGACTGTTTGGATCACTGCAAA
 2 canna_FASN      99.7%
 AGTTCTGCTTTGTGCCCTATACTGAGAGTGACTGTTTGTCTTCCAATGCCCATCTGCATGACTGTTTGGATCACTGCAAA
 3 swift_FASN      90.8%
 AATTCTGCTTTGTGCCCTATACTGAAAGTGACTGTTTGTCTTCCAGTGTCCATCTGCATGACTATTTGAATCACTGCAAA
 4 human_FASN      64.1%
 AGTTTTGCTTCACTCCCCACACGGAGGAGGGGTGCCTGTCTGAGCGCGCTGCCCTGCAGGAGGAGCTGCAACTGTGCAAG
 5 gallus_FASN     88.9%
 AATTCTCTTTTGTTCCCTATATTGAGAGTGATTGTTTGTCTTCCAGTACCCAGCTTCATGCCTACCTGGAGCACTGCAAA
 6 alligator_FASN  75.2%
 AGTTCTGCTTTGTGCCTTATGTTGAGATGGATTGTCTGTCTTCCGATGCACAGCTTCACACCGTTCTGGAGCATTGCAAA

                           3441         :         .         .         .         .         5
  .        . 3520
 1 ruby_FASN      100.0%
 GTTCTGATCCAGAAGATGCAGGCTAAACTAGCAGTGCATGGGGTCAAGTTAGTCATCCCTGGACTAGACACTGGAAGTAC
 2 canna_FASN      99.7%
 GTTCTGATCCAGAAGATGCAGGCTAAACTAGCAGTGCATGGGGTCAAGTTAGTCATCCCTGGACTAGACACTGGAAGTAC
```

```
  3 swift_FASN      90.8%
  GACCTGATCCAAAACTTAGAGGAAAAAATGGCAGTGCACGGAGTCAGATTAGTCATCCCTGGACTAGAAACTGAAAAGGG
  4 human_FASN      64.1%
  GGGCTGGTGCAGGCACTGCAGACCAAGGTGACCCAGCAGGGGCTGAAGATGGTGGTGCCCGGACTGGATGGGGCCCAGAT
  5 gallus_FASN     88.9%
  GGCCTGATCCAGAAATTACAAGCTAAGATGGCATTGCACGGAGTCAAACTAGTTATCCATGGCCTAGAAACCAAAGGGGC
  6 alligator_FASN  75.2%
  GGTCTGATCCAGAACCTGCAGAACAAGGTAGCGCTGCATGGGGTTAGACTGGCCATCCCTGGGCTAGAGACTACAGGCAC

                          3521          .         .         :         .         .         .
  .         6 3600
  1 ruby_FASN      100.0%
  TGCTGCAAAGTCCTCACCCATACAGAAAGGCCTTCAGCATATCCTTACTGAAATCTGCCGTCTGGAACTGAATGGAAACC
  2 canna_FASN      99.7%
  TGCTGCAAAGTCCTCACCCATACAGAAGGGCCTTCAGCATATCCTTACTGAAATCTGCCGTCTGGAACTGAATGGAAACC
  3 swift_FASN      90.8%
  TGTTTCAAAGTCCTCACCCATACAGAAAGGCCTTCAGCATATCCTTGCTGAAATCTGCCGTCTGGAACTGAATGGAAACC
  4 human_FASN      64.1%      CCC--CCGGGACC---
  -CCTCACAGCAGGAACTGCCCCGGCTGTTGTCGGCTGCCTGCAGGCTTCAGCTCAACGGGAACC
  5 gallus_FASN     88.9%
  TGCTGCAGGATCCCCACCCACACAGAAGGGCCTTCAGCATATCCTTACTGAAATCTGCCGTCTGGAACTGAATGGAAACC
  6 alligator_FASN  75.2%
  CAGGGCAGAGACCGTGCCTGCACAGAAGGGCCTCCTGTACATCCTTGATAAAATCTGCCACCTGGAACTAAATGGAAACC

                          3601          .         .         .         .         :         .
  .         . 3680
  1 ruby_FASN      100.0%
  TCCGTTCTGAGCTGGAACAGATTGTGACTCGAGAGAAAATGCACTTCCATGATGATCCTCTTCTCAGTGGATTGCTAGAT
  2 canna_FASN      99.7%
  TCCGTTCTGAGCTGGAACAGATTGTGACTCGAGAGAAAATGCACTTCCATGATGATCCTCTTCTCAGTGGATTGCTAGAT
  3 swift_FASN      90.8%
  TCCATTCTGAGCTGCAACAGATTGTGACTCGGGAGAAAATGCACTTCCAGGATGATCCTCTTCTCAATGGATTGCTGGAT
  4 human_FASN      64.1%
  TGCAGCTGGAGCTGGCGCAGGTGCTGGCCCAGGAGAGGCCCAAGCTGCCAGAGGACCCTCTGCTCAGCGGCCTCCTGGAC
  5 gallus_FASN     88.9%
  TACATTCTGAGCTGGAACAGATTGTGACTCAGGAGAAGATGCACCTCCAGGACGATCCCCTTCTCAATGGCTTGCTGGAT
  6 alligator_FASN  75.2%
  TGTATTCTGAGCTGGATGAGATTGTGACCCAAGAGAAGATGCATCTCCAGGAGGATCCCCTTCTCAATGGCTTGCTGGAT

                          3681          .         7         .         .         .         .
  :         . 3760
  1 ruby_FASN      100.0%
  TCTTCAGAGCTGAAGAGTTGCCTGGATGTGGCAGTGGAGAACATGACCAGTCACAGAATGAAGATAATAGAGGCTCTTGC
  2 canna_FASN      99.7%
  TCTTCAGAGCTGAAGAGTTGCCTGGATGTGGCAGTGGAGAACATGACCAGTCACAGAATGAAGATAATAGAGGCTCTTGC
  3 swift_FASN      90.8%
  TCTTCAGAGTTGAAGACTTGTCTGGATGTGGCACTGGAGAACATGACCAGTCACAGGATGAAGATAGTAGAGGCTCTTGC
  4 human_FASN      64.1%
  TCCCCGGCACTCAAGGCCTGCCTGGACACTGCCGTGGAGAACATGCCCAGCCTGAAGATGAAGGTGGTGGAGGTGCTGGC
  5 gallus_FASN     88.9%
  TCTTCAGAGTTGAAGACTTGCCTGGATGTGGCAAAGGAGAACACGACCAGTCACAGGATGAAGATAGTGGAGGCTCTGGC
  6 alligator_FASN  75.2%
  TCCCCAGAGCTGAAGGTTTGTCTGGACTTAACCGTGGAGAATACGTCCAATATCAAGATGAAGATTGTGGAGGCACTGGC

                          3761          .         .         .         8         .         .
  .         . 3840
  1 ruby_FASN      100.0%
  AGGAAGTGGACGTCTGTTCTCTCGTGTCAAAAGTATTCTGAATACTCAGCCCTTGTTGCAAGTGGACTACATTGCCACTG
  2 canna_FASN      99.7%
  AGGAAGTGGACGTCTGTTCTCTCGTGTCAAAAGTATTCTGAATACTCAGCCCTTGTTGCAAGTGGACTACATTGCCACTG
  3 swift_FASN      90.8%
  TGGCAGTGGACGTCTGTTCTCTCGTGTCAAAAGTATTCTGAATACTCAGCCCCTGTTGCAGGTGGACTACATTGCCACTG
  4 human_FASN      64.1%
  TGGCCACGGTCACCTGTATTCCCGCATCCCAGGCCTGCTCAGCCCCCATCCCCTGCTGCAGCTGAGCTACACGGCCACCG
  5 gallus_FASN     88.9%
  AGGAAGTGGACGTCTGTTCTCTCGTGTCCAAAGTATTCTGAATACTCAACCCCTGTTGCAGCTGGACTACATTGCCACTG
  6 alligator_FASN  75.2%
  AGGAGATGGATGCCTGTTCCCTCGTATCACTCCTCTTCTAGGAACTCATCCCTTGCTAGAAGTTGACTACACTGCTACTG

                          3841          :         .         .         .         .         9
  .         . 3920
  1 ruby_FASN      100.0%
  ACCGTGTCCTGGAAACTCTTTCAGCTAGTGAAACAGAACTGCAAGATGCTGGAGTTTCCTCTAGCCAGTGGGATCCATCC
  2 canna_FASN      99.7%
  ACCGTGTCCTGGAAACTCTTTCAGCTAGTGAAACAGAGCTGCAAGATGCTGGAGTTTCCTCTAGCCAGTGGGATCCATCC
  3 swift_FASN      90.8%
  ACCACATCCAGGAAGCTCTTTCAGCTCATGAAACAGAGCTAGAAGATGCTGGAGTTTCCTTTAGCCAGTGGGATCCATCT
  4 human_FASN      64.1%
  ACCGCCACCCCCAGGCCCTGGAGGCTGCCCAGGCCGAGCTGCAGCAGCACGACGTTGCCCAGGGCCAGTGGGATCCCGCA
  5 gallus_FASN     88.9%
  ACTGCACCCCTGAAACTCTTTCAGATAATGAAACAGAGCTGCACGATGCTGGAATCTCCTTTAGCCAGTGGGATCCCTCT
```

```
   6 alligator_FASN  75.2%
   ATAGAGCCCAGGAGGTCCTTTCTGCCCATGAGAAGCAGTTTCAGGAAACATCTGTTTCCCCTGGCCAGTGGGACCCTGTC

                           3921          .         .         :         .         .         .
   .         0 4000
   1 ruby_FASN      100.0%
   AGCCTTCCATCGGGAAATCTGACCAATGCTGACCTGGTGGTATACAACTGTGCAACAAACGTTCTAGGGAACACCACGGA
   2 canna_FASN      99.7%
   AGCCTTCCATCGGGAAATCTGACCAATGCTGACCTGGTGGTATACAACTGTGCAACAAACGTTCTAGGGAACACCACGGA
   3 swift_FASN      90.8%
   AGCCTTCCGTCTGGAAATCTGACCAATGCTGACATGGTGGTATACAACTGTTCAACAAGTGCTCTAGGGAACAATGCTGA
   4 human_FASN      64.1%
   GACCCTGCCCCCAGCGCCCTGGGCAGCGCCGACCTCCTGGTGTGCAACTGTGCTGTGGCTGCCCTCGGGGACCCGGCCTC
   5 gallus_FASN     88.9%
   AGCCTTCCCTCTGGAAATCTGACCAATGCTGACCTGGCAGTATGCAACTGTTCAACAAGTGTTCTGGGGAACACAGCTGA
   6 alligator_FASN  75.2%
   CGTCCTCCGCCTGGAAACCTGACCAATGCTGACCTGCTGGTGCTCAACTGCTCCATGAAAGTCTTGGAGAAGCCAGCAGA

                           4001          .         .         .         .         :         .
   .         . 4080
   1 ruby_FASN      100.0%
   AATTCTTTCTAACTTGGCAGCTGCAGTGAAAGAAGGAGGGTTTGTTTTGCTACACACTCTTCTTAAAGGAGAAACTCTTG
   2 canna_FASN      99.7%
   AATTCTTTCTAACTTGGCAGCTGCAGTGAAAGAAGGAGGGTTTGTTTTGCTACACACTCTTCTTAAAGGAGAAACTCTTG
   3 swift_FASN      90.8%
   AATTCTTTCTAATTTGGCAGCTGGAGTGAAAGAAGGAGGGTTTGTTTTGGTACACACCCTTCTTAAAGGAGAAACTCTTG
   4 human_FASN      64.1%
   AGCTCTCAGCAACATGGTGGCTGCCCTGAGAGAAGGGGGCTTTCTGCTCCTGCACACACTGCTCCGGGGGCACCCCCTCG
   5 gallus_FASN     88.9%
   AATTATCTCTAACTTAGCAGCTGCAGTGAAAGAAGGAGGGTTTGTTTTGCTGCACACCCTTCTTAAAGAGGAAACTCTTG
   6 alligator_FASN  75.2%
   GATACTCTCCAACCTGGTGGCCTCCGTGAAGGAAGGAGGGTTTGTGCTACTGCATACTCTTCTGGGAGGAGAAACTCTTG

                           4081          .         1         .         .         .         .
   :         . 4160
   1 ruby_FASN      100.0%
   GAGAAATTGTCAGTTTCCTTTCAAGTCCAGATCTACAGCAGAAACATAAGTTCCTGCATGAGACACAGTGGGAGGACTTA
   2 canna_FASN      99.7%
   GAGAAATTGTCAGTTTCCTTTCAAGTCCAGATCTACAGCAGAAACATAAGTTCCTGCATGAGACACAGTGGGAGGACTTA
   3 swift_FASN      90.8%
   GAGAAATTGTGAGTTTCCTAACAAGTCCAGATCTGCAGCAGAAGCACAGGTTCCTCAGTGAGGCACAGTGGGAGGGCTTA
   4 human_FASN      64.1%
   GGGACATCGTGGCCTTCCTCACCTCCACTGAGCCGCAGTATGGCCAGGGCATCCTGAGCCAGGACGCGTGGGAGAGCCTC
   5 gallus_FASN     88.9%
   GAGAAATTGTCAGCTTTCTTACAAGTCCAGACCTACAGCAGAAGCACAGCTTCCTGTCTCAGGCACAGTGGGAGGAGTTA
   6 alligator_FASN  75.2%
   GAGAAACGGTCCATTTCCTTACAGCACTAGATGTGCAGCAGAGACCAGGCTTCCTGACTCAGGCAGCCTGGGAGGATTTG

                           4161          .         .         .         2         .         .
   .         . 4240
   1 ruby_FASN      100.0%
   TTCAGCAAAGCCTCACTGAATCTGGTTGCCATGAAGAAATCATTCTTTGGCTCAGTTATTTTCTTGTGTCGCCGACAAGT
   2 canna_FASN      99.7%
   TTCAGCAAAGCCTCACTGAATCTGGTTGCCATGAAGAAATCATTCTTTGGCTCAGTTATTTTCTTGTGTCGCCGACAAGT
   3 swift_FASN      90.8%
   TTCAGCAAAGCCTCACTGAATCTGGTTGCCATAAAGAGATCATTCTTTGGCTCAGTTATTTTCCTGTGTCGCCGGCAAGT
   4 human_FASN      64.1%
   TTCTCCAGGGTGTCGCTGCGCCTGGTGGGCCTGAAGAAGTCCTTCTACGGCTCCACGCTCTTCCTGTGCCGCCGGCCCAC
   5 gallus_FASN     88.9%
   TTCAGCAAGGCCTCATTGAATCTGGTTGCAATGAAGAGATCTTTCTTTGGCTCAGTTATTTTCCTGTGTCGACGGCAGTC
   6 alligator_FASN  75.2%
   TTCAGCAAGACCTCGCTGAATCTCGTGGCTGTGAAAAGGTCCTTTTTTGGCTCTGTCATGTTCTTGTGCCGCCGGCAGGC

                           4241          :         .         .         .         .         3
   .         . 4320
   1 ruby_FASN      100.0%
   ACCTGCCAAAACACCCATTTTTCTGCCAGTGGATGAGACCCATTATAAGTGGGTTGAATCTTTAAAGGAGATCTTGGCCG
   2 canna_FASN      99.7%
   ACCTGCCAAAACGCCCATTTTTCTGCCAGTGGATGAGACCCATTATAAGTGGGTTGAATCTTTAAAGGAGATCTTGGCCG
   3 swift_FASN      90.8%
   CCCTGCCAAAACACCCATTTTTCTGCCAATAGATGAGACCCATTATAAGTGGGTTGAATCTTTAAAGGAGATCTTGGCTG
   4 human_FASN      64.1%
   CCCGCAGGACAGCCCCATCTTCCTGCCGGTGGACGATACCAGCTTCCGCTGGGTGGAGTCTCTGAAGGGCATCCTGGCTG
   5 gallus_FASN     88.9%
   CCCTGCCAAAGCACCCATTCTTCTGCCAGTAGATGACACTCATTATAAGTGGGTTGACTCCTTAAAGGAGATCTTGGCTG
   6 alligator_FASN  75.2%
   TCCTGTCAAGCCACCTATTTTCCTGCCTGTGGATGAGACCCATTGCAAGTGGGTGGATTTCCTAAAGAACTTCATGGCTG

                           4321          .         .         :         .         .         .
   .         4 4400
   1 ruby_FASN      100.0%      ATTCA--
```

-TCCGAGCAGCCTGTGTGGTTGACTGCCACCAGTTGTGGGAACTCGGGAATTTTGGGAATGGTGAACTGCCTC
2 canna_FASN   99.7%   ATTCA--
-TCCGAGCAGCCTGTGTGGTTGACTGCCACCAGTTGTGGGAACTCGGGAATTTTGGGAATGGTGAACTGCCTC
3 swift_FASN   90.8%   GCCCA--
-TCAGAACAGCCTGTGTGGTTGACTGCCACCAGTTGTGGGAACTCAGGAATTTTGGGAATGGTGAACTGCCTC
4 human_FASN   64.1%
ACGAAGACTCTTCCCGGCCTGTGTGGCTGAAGGCCATCAACTGTGCCACCTCGGGCGTGGTGGGCTTGGTGAACTGTCTC
5 gallus_FASN   88.9%   ACTCA--
-TCAGAGCAGCCTCTGTGGTTGACTGCCACCAATTGTGGGAACTCTGGAATTTTGGGTATGGTGAACTGCCTC
6 alligator_FASN 75.2%   ATTCA--
-TCTGAGCAGCCTTTGTGGCTGACTGCAACCAAGTATCCAACCTCTGGCATTGTGGGACTGATGACCTGCCTG

4401 . . . . : .
. . 4480
1 ruby_FASN   100.0%
CGCCTTGAATCAGAAGGCCATAGAATCAGGTGTGTGTTCATTTCCAACTTGAACCCTTCATCAGCTGTCCCACCCACAAG
2 canna_FASN   99.7%
CGCCTTGAATCAGAAGGCCATAGAATCAGGTGTGTGTTCATTTCCAACTTGAACCCTTCATCAGCTGTCCCACCCACAAG
3 swift_FASN   90.8%
CGCCTGGAAGCAGAAGGCCACAGAATCAGGTGTGTATTCATTTCCAACTTGAACCCTTCATCAGCTGTCCCACCCACAAG
4 human_FASN   64.1%
CGCCGAGAGCCCGGCGGGAACCGCCTCCGGTGTGTGCTGCTCTCCAACCTCAGCAGCACCTCCCACGTCCCGGAGGTGGA
5 gallus_FASN   88.9%
CGCCTGGAAGCAGAGGGCCACAGAATCAGGTGTGTGTTTGTTTCCAACCTGAGCCCTTCATCAACTGTCCCAGCCACTAG
6 alligator_FASN 75.2%
CGCCAGGAGCCTGGAGGCCACAGGATCAGGTCCCTGTTTGTTTCCAATCTGGAGTCCTCCTCCCCTGCGCCTCCAACCAG

4481 . 5 . . . .
: . 4560
1 ruby_FASN   100.0%
TCATTCTTCCCTGGAGATGCAGAAGATTGTTCAGGGGGATCTGGTCATGAATGTGTATCGTGATGGGAAGTGGGGCTCCT
2 canna_FASN   99.7%
TCATTCTTCCCTGGAGATGCAGAAGATTGTTCAGGGGGATCTGGTCATGAATGTGTATCGTGATGGGAAGTGGGGCTCCT
3 swift_FASN   90.8%
TCCTTCTTCCCTGGATATGCAGAAGGTTGTTCAGAATGACCTGGTGATGAATGTGTATCGTGATGGAAAGTGGGGCTCCT
4 human_FASN   64.1%
CCCGGGCTCCGCAGAACTGCAGAAGGTGTTGCAGGGAGACCTGGTGATGAACGTCTACCGCGACGGGGCCTGGGGGGCTT
5 gallus_FASN   88.9%
TCTTTCTTCCCTGGAGATGCAGAAGATTATTGAGAGAGATCTGGTGATGAATGTGTATCGTGATGGAAAGTGGGGTTCCT
6 alligator_FASN 75.2%
CCCATCAGACTCAGAGATGCAAAAGATCCTGCAGAGAGACCTGGTGATGAATGTGTATCGTGATGGGAAGTGGGGCTCCT

4561 . . . 6 . .
. . 4640
1 ruby_FASN   100.0%
TCAGGCATCTCCCATTGCAGCAAGCACAACCTCAGGAGCTGACAGAATATGCCTATGTAAATGTGTTGACTCGTGGAGAT
2 canna_FASN   99.7%
TCCGGCATCTCCCATTGCAGCAAGCACAACCTCAGGAGTTGACAGAATATGCCTATGTAAATGTGTTGACTCGTGGAGAT
3 swift_FASN   90.8%
TCAGGCACCTCCCATTGCAGCAAGCGCAACCTCAGGAGCTGACAGAATATGCTTATGTAAATGTGTTGACTCGTGGAGAC
4 human_FASN   64.1%
TCCGCCACTTCCTGCTGGAGGAGGACAAGCCTGAGGAGCCGACGGCACATGCCTTTGTGAGCACCCTCACCCGGGGGGAC
5 gallus_FASN   88.9%
TCAGGCATCTCCCATTGCAGCAAGCTCAGCCTCAGGAGCTGACAGAATATGCCTACGTAAATGTGTTGACTCGTGGAGAT
6 alligator_FASN 75.2%
TCCGGCACCTCCTGTTGGAGCAAGCCCAGCCCCAGACTTTGACGGAACATGCCTGTGTGAACACACTGATGCGTGGAGAC

4641 : 7
. . 4720
1 ruby_FASN   100.0%
CTCTCATCTCTTCGTTGGATTGTCTCCCCACTTCAACACTTCCAGGCAAATAATCCTGATATTCAACTCTGCAAAGTCTA
2 canna_FASN   99.7%
CTCTCATCTCTTCGTTGGATTGTCTCCCCACTTCAACACTTCCAGGCAAATAATCCTGATATTCAACTCTGCAAAGTCTA
3 swift_FASN   90.8%
CTGTCATCTCTTCGTTGGATTGTCTCCCCACTTCAGCACTTTTGTGCAAACAACCCAAATGTTCAACTCTGCAAGGTCTA
4 human_FASN   64.1%
CTGTCCTCCATCCGCTGGGTCTGCTCCTCGCTGCGCCATGCCCAGCCCACCTGCCCTGGCGCCCAGCTCTGCACGGTCTA
5 gallus_FASN   88.9%
CTCTCTTCCCTTCGTTGGATTGTTTCCCCACTTCGACACTTCCAAACAACCAATCCAAATGTTCAGCTCTGCAAAGTCTA
6 alligator_FASN 75.2%   CTCTCTTCCCTTCGCTGGATTGCCTCTCCGCTCTGCCACT-----------
-ATCCCAGCACTAAGCTCTGCAAGGTTTA

4721 . . : . . .
. 8 4800
1 ruby_FASN   100.0%
TTATGCGTCTCTCAACTTCCGGGACATTATGCTGGCCACAGGAAAGCTTTCTCCAGATGCAATCCCTGGTAACTGGGTTT
2 canna_FASN   99.7%
TTATGCGTCTCTCAACTTCCGGGACATTATGCTGGCCACAGGAAAGCTTTCTCCAGATGCAATCCCTGGTAACTGGGTTT
3 swift_FASN   90.8%
TTACACGTCTCTCAACTTCCGGGATATCATGCTAGCCACAGGAAAGCTTTCTCCAGATGCTATCCCTGGTAACTGGGCCA
4 human_FASN   64.1%

```
CTACGCCTCCCTCAACTTCCGCGACATCATGCTGGCCACTGGCAAGCTGTCCCCTGATGCCATCCCAGGGAAGTGGACCT
5 gallus_FASN     88.9%
CTATGCATCTCTCAATTTCCGGGACATTATGCTGGCAACAGGAAAGCTTTCTCCAGATGCTATCCCTGGTAACTGGACGT
6 alligator_FASN  75.2%
TTATGCCTCTCTCAATTTCCGTGACATCATGTTGGCCTCGGGGAAGCTCCCCGCAGATGCTATACCAGGTAACTGGAGTA

                        4801          .         .         .         .         :         .
.         . 4880
1 ruby_FASN      100.0%
TGCAGCAGTGCATGCTGGGCATGGAGTTCTCTGGACGGGACATGGCTGGAAGAAGAGTGATGGGATTACTGCCAGCAAAG
2 canna_FASN      99.7%
TGCAGCAGTGCATGCTGGGCATGGAGTTCTCTGGACGGGACATGGCTGGAAGAAGAGTGATGGGATTACTGCCAGCAAAG
3 swift_FASN      90.8%
TGCAGCAGTGCATGCTGGGCATGGAGTTCTCCGGACGGGACCTGGCTGGAAGAAGAGTGATGGGATTGCTGCCAGCAAAG
4 human_FASN      64.1%
CCCAGGACAGCCTGCTAGGTATGGAGTTCTCGGGCCGAGACGCCAGCGGCAAGCGTGTGATGGGACTGGTGCCTGCCAAG
5 gallus_FASN     88.9%
TGCAGCAGTGCATGCTGGGCATGGAGTTCTCAGGACGGGACCTGGCTGGAAGGAGAGTGATGGGATTGCTGCCAGCAAAA
6 alligator_FASN  75.2%
CAAAGGACAACTTGCTGGGAATGGAGTTCTCGGGGCGGGACCCTGCTGGAAAACGGGTTATGGGATTGCTGCAGGCAAAG

                        4881          .         9         .         .         .         .
:         . 4960
1 ruby_FASN      100.0%
GGGCTGGCTACAGTGGTAG-ACTGTGAAAAGAAGTTTCTGTGGGAGGTGCCTAAAAACTGGACTCTGGAAGAAGCAGCTT
2 canna_FASN      99.7%
GGGCTGGCTACAGTGGTAG-ACTGTGAAAAGAAGTTTCTGTGGGAGGTGCCTAAAAACTGGACTCTGGAAGAAGCAGCTT
3 swift_FASN      90.8%
GGGCTGGCTACAGTGGTGG-ACTGTGACACAAGGTTTCTATGGGAGGTGCCCAAGAACTGGACTCTGGAAGAAGCAGCTT
4 human_FASN      64.1%      GGCCTGGCCACCTCTGTCCTGCTGTCACCGGA-
CTTCCTCTGGGATGTGCCTTCCAACTGGACGCTGGAGGAGGCGGCCT
5 gallus_FASN     88.9%
GGGCTGGCTACAGTGGTGG-ACTGTGACAAGAGGTTTCTATGGGAAGTGCCTGAAAACTGGACTCTGGAAGAAGCAGCTT
6 alligator_FASN  75.2%
GGACTGGCTACAGCGGTGG-ACATTGACAGGTGTTGCCTGTGGGATGTGCCAGAGAACTGGACCCTGGAGGAAGCAGCCA

                        4961          .         .         .         0         .         .
.         . 5040
1 ruby_FASN      100.0%
CAGTACCTGTGGTTTATGCCACTGCTTACTATGCTTTGGTGGTTCGAGGTAGAATGAAGAAGGGTGAGAGTGTCCTTGTT
2 canna_FASN      99.7%
CAGTACCTGTGGTTTATGCCACTGCTTACTATGCTTTGGTGGTTCGAGGTAGAATGAAGAAGGGTGAGAGTGTCCTTGTT
3 swift_FASN      90.8%
CAGTGCCTGTGGTTTATGCCACTGCTTATTATGCTTTGGTGGTTCGAGGTGGTATGAAGAAGGGTGAGAGTATCCTTATT
4 human_FASN      64.1%
CGGTGCCTGTCGTCTACAGCACGGCCTACTACGCGCTGGTGGTGCGTGGGCGGGTGCGCCCCGGGGAGACGCTGCTCATC
5 gallus_FASN     88.9%
CGGTGCCTGTGGTTTATGCCACTGCTTATTATGCTTTGGTGGTTCGAGGTGGTATGAAGAAGGGGGAGAGCGTCCTCATT
6 alligator_FASN  75.2%
CCGTGCCCGCGGTCTATGCCACAGCTTATTATGCTTTGATCTCTCGTGGTGGCATGAAGCGGGGGGAGAGCGTGCTCATT

                        5041          :         .         .         .         .         1
.         . 5120
1 ruby_FASN      100.0%
CACTCTGGCTCAGGAGGTGTGGGCCAAGCAGCCATTGCCATTGCCCTGAGTATGGGCTGCCGTGTCTTTACTACTGTAGG
2 canna_FASN      99.7%
CACTCTGGCTCAGGAGGTGTTGGCCAAGCAGCCATTGCCATTGCCCTGAGTATGGGCTGCCGTGTCTTTACTACTGTAGG
3 swift_FASN      90.8%
CACTCTGGCTCAGGGGGTGTGGGCCAAGCAGCCATCGCCATTGCCCTGAGCATGGGCTGCCGTGTCTTTGCTACTGTAGG
4 human_FASN      64.1%
CACTCGGGCTCGGGCGGCGTGGGCCAGGCCGCCATCGCCATCGCCCTCAGTCTGGGCTGCCGCGTCTTCACCACCGTGGG
5 gallus_FASN     88.9%
CACTCTGGCTCAGGAGGTGTGGGCCAAGCAGCTATTGCCATCGCCTTGAGCATGGGCTGCCGTGTTTTTGCTACTGTAGG
6 alligator_FASN  75.2%
CACTCAGGCTCTGGGGGTGTGGGCCAGGCAGCTATCACCATAGCCCTGAGCATGGGCTGCCGTGTCTTCACAACTGTGGG

                        5121          .         .         :         .         .         .
.         2 5200
1 ruby_FASN      100.0%
CTCCGCTGAGAAACGTGAATATCTCCAAGCAAGATTCCCACAGCTGGATGCTAATAGCTTTGCCAGCTCCCGAGATACTG
2 canna_FASN      99.7%
CTCCGCTGAGAAACGTGAATATCTCCAAGCAAGATTCCCACAGCTGGATGCTAATAGCTTTGCCAGCTCCCGAGATACTG
3 swift_FASN      90.8%
CTCCACTGAGAAACGAGAGTATCTCCAAGCAAGATTCCCACAGCTGGATGCTAATAGCTTTGCCAGCTCCCGAAGTGCTG
4 human_FASN      64.1%
GTCGGCTGAGAAGCGGGCGTACCTCCAGGCCAGGTTCCCCCAGCTCGACAGCACCAGCTTCGCCAACTCCCGGGACACAT
5 gallus_FASN     88.9%
CTCTGCTGAGAAACGTGAGTATCTCCAAGCAAGGTTCCCACAGCTGGATGCTAATAGCTTTGCCAGCTCCCGAAATACAA
6 alligator_FASN  75.2%
TTCTACTGAGAAACGCAAGTATCTCCAAGCACGGTTCCCCCAGCTGGATGCAAGCAGTTTTGCCAACTCTAGAAGTACCG
```

```
                        5201           .         .         .         .         :         .
.         . 5280
1 ruby_FASN      100.0%
CCTTTGAGCAACATGTACTGCGAGTTACCAATGGAAAAGGTGTCAACCTAGTGCTAAATTCCTTGGCAGAAGAGAAGCTC
2 canna_FASN      99.7%
CCTTTGAGCAACATGTACTGCGAGTTACCAATGGAAAAGGTGTCAACCTAGTGCTAAATTCCTTGGCAGAAGAGAAGCTC
3 swift_FASN      90.8%
CCTTCGAGCAGCACATCCTGCGAGTTACCAATGGAAAAGGTGTCAACCTAGTGCTAAATTCCTTGGCAGAAGAGAAGCTC
4 human_FASN      64.1%
CCTTCGAGCAGCATGTGCTGTGGCACACGGGCGGGAAGGGCGTTGACCTGGTCTTGAACTCCTTGGCGGAAGAGAAGCTG
5 gallus_FASN     88.9%
CCTTTGAGCAACACATACTGCGAGTTACCAATGGGAAAGGTGTCAACCTTGTGTTAAATTCCTTGGCAGAAGAGAAGCTC
6 alligator_FASN  75.2%
CCTTTGAGCAACATATTCTGCGGGCTACCAAAGGGAAAGGTGTTGACCTCGTGCTAAACTCCTTGGCAGAAGAGAAGCTC

                        5281           .         3         .         .         .         .
:         . 5360
1 ruby_FASN      100.0%
CAAGCCAGTTTGCGTTGTCTCGCTCGACATGGTCGCTTCTTGGAAATAGGCAAATTTGATCTGTCTAACAACAGCCAGCT
2 canna_FASN      99.7%
CAAGCCAGTTTGCGTTGTCTCGCTCGACATGGTCGCTTCTTGGAAATAGGCAAATTTGATCTGTCTAACAACAGCCAGCT
3 swift_FASN      90.8%
CAGGCCAGTTTGCGTTGTCTTGCACGGCACGGGCGCTTTTTGGAAATAGGCAAATTTGATCTGTCTAACAACAGCCAGCT
4 human_FASN      64.1%
CAGGCCAGCGTGAGGTGCTTGGCTACGCACGGTCGCTTCCTGGAAATTGGCAAATTCGACCTTTCTCAGAACCACCCGCT
5 gallus_FASN     88.9%
CAAGCCAGTTTGCGTTGTCTTGCTCAACATGGGCGCTTCTTGGAAATAGGCAAATTTGATCTATCAAACAACAGCCAGCT
6 alligator_FASN  75.2%
CAAGCCAGTCTACGTTGCCTTGCTCAACACGGACGCTTTTTGGAAATCGGCAAATTTGATCTGTCCAAAAACAACCATCT

                        5361           .         .         .         4         .         .
.         . 5440
1 ruby_FASN      100.0%
TGGAATGGCTCTCTTCCTCAAGAATGTGGCATTTCATGGGATCCTACTAGATGCAATCTTTGAGGGTGGAAACCAAGAGT
2 canna_FASN      99.7%
TGGAATGGCTCTCTTCCTCAAGAATGTGGCATTTCATGGGATCCTACTAGATGCAATCTTTGAGGGTGGAAACCAAGAGT
3 swift_FASN      90.8%
TGGAATGGCTCTCTTCCTCAAGAATGTGGCATTTCATGGAATCCTACTAGATGCAATCTTTGAGGATGGAAATCAAGAGT
4 human_FASN      64.1%
CGGCATGGCTATCTTCCTGAAGAACGTGACATTCCACGGGGTCCTACTGGATGCGTTCTTCAACGAGAGCAGTGCTGACT
5 gallus_FASN     88.9%
TGGAATGGCTCTTTTCCTCAAGAATGTGGCGTTTCATGGAATCCTGCTGGATTCAATCTTTGAGGAAGGAAACCAAGAGT
6 alligator_FASN  75.2%
AGGAATGGCTCTCTTCCTGAAGAATGTGACGTTCCATGGGATCCTGCTAGATGCTGTTTTGAAAAAGGGAAGTCCTGCAT

                        5441           :         .         .         .         .         5
.         . 5520
1 ruby_FASN      100.0%
GGGATATAGTATCAGAGTTGTTGACAAAAGGCATAAAAGATGGTGTGGTAAAACCCTTGAAGAGTACAGTCTTCAAAAAA
2 canna_FASN      99.7%
GGGATATAGTATCAGAGTTGTTGACAAAAGGCATAAAAGATGGTGTGGTAAAACCCTTGAAGAGTACAGTCTTCAAAAAA
3 swift_FASN      90.8%
GGGACTTAGTATCAGAGTTGTTGACAAAAGGCATAAAAGATGGTGTGGTAAAGCCCCTGCGGAGTACAGTCTTCAACAAA
4 human_FASN      64.1%
GGCGGGAGGTGTGGGCGCTTGTGCAGGCCGGCATCCGGGATGGGGTGGTACGGCCCCTCAAGTGCACGGTGTTCCATGGG
5 gallus_FASN     88.9%
GGGAGGTGGTATCAGAGTTGTTGACAAAAGGCATAAAAGATGGTGTGGTAAAGCCCCTGAGAACCACAGTCTTCGGTAAA
6 alligator_FASN  75.2%
GGGTGGAAGTGGAAGAGTTGGTGAAGAAGGGGATAAGAGATGGCATAGTGAAACCCTTGAAGCACACTGTCTTTGGCAAA

                        5521           .         .         :         .         .         .
.         6 5600
1 ruby_FASN      100.0%
GAAGAGGTAGAAGCTGCCTTCAGGTTCATGGCTCAAGGAAAACATATTGGCAAAGTTATGATCCAGATCCAAGAAGAGGA
2 canna_FASN      99.7%
GAAGAGGTAGAAGCTGCCTTCAGGTTCATGGCTCAAGGAAAACATATTGGCAAAGTTATGATCCAGATCCAAGAAGAGGA
3 swift_FASN      90.8%
GAAGAGGTAGAAGCTGCCTTTAGGTTCATGGCACAAGGAAAGCATATTGGCAAAGTTATGATCAAGATTCAAGAAGAGGA
4 human_FASN      64.1%
GCCCAGGTGGAGGACGCCTTCCGCTACATGGCCCAAGGGAAGCACATTGGCAAAGTCGTCGTGCAGGTGCTTGCGGAGGA
5 gallus_FASN     88.9%
GAAGAGGTAGAAGCTGCCTTCAGGTTCATGGCGCAAGGAAAACATATTGGCAAAGTTATGATCAAGATCCAAGAAGAGGA
6 alligator_FASN  75.2%
GAGGAGTTGGAAGCTGCCTTTAGGTTCATGGCACAAGGAAAACACATTGGCAAAGTTATGGTTAAGGTCCGGGAAGAAGA

                        5601           .         .         .         .         :         .
.         . 5680
1 ruby_FASN      100.0%
AAAGGAATATTCTGTAAGAAGGTCTGAACCAGTTAAAATCTCAGCCATCTCACGAACCTCCTGTCCACCTTCCAAATCCT
2 canna_FASN      99.7%
AAAGGAATATTCTGTAAGAAGGTCTGAACCAGTTAAAATCTCAGCCATCTCACGAACCTCCTGTCCACCTTCCAAATCCT
```

```
 3 swift_FASN      90.8%
 AAAGGAATATGCTTTAAGAAGATCTGAACCAGTTAAAATCTCGGCCATCTCCCGAACCTCCTGTCCACCTACAAAGTCAT
 4 human_FASN      64.1%
 GCCGGAGGCAGTGCTGAAGGGGGCCAAACCCAAGCTGATGTCGGCCATCTCCAAGACCTTCTGCCCGGCCCACAAGAGCT
 5 gallus_FASN     88.9%      GAAGCAATATCCTTTAAG--
 -GTCTGAACCAGTAAAACTCTCTGCCATCTCCCGAACTTCCTGCCCACCTACCAAGTCTT
 6 alligator_FASN  75.2%
 GAAGGACTTGCCTGCAAGTGATTCTGTGCTAACCCAACTCCCTGCCATCTCCCGCACTTTTTGCCCACCTACCAAATCCT

                         5681          .         7         .         .         .         .
 :         . 5760
 1 ruby_FASN      100.0%
 ACATCATCACAGGGGGCTTAGGAGGATTTGGGCTTGAGTTGGCACAGTGGCTAGTTGAGAGAGGAGCACAGAAGCTTATC
 2 canna_FASN      99.7%
 ACATCATCACAGGGGGCTTAGGAGGATTTGGGCTTGAGTTGGCACAGTGGCTAGTTGAGAGAGGAGCACAGAAGCTTATC
 3 swift_FASN      90.8%
 ACATCATCACAGGGGGCCTGGGAGGATTTGGGCTTGAGTTGGCACAGTGGCTAGTTGAGAGAGGAGCACAGAAGCTTGTA
 4 human_FASN      64.1%
 ACATCATCGCTGGTGGTCTGGGTGGCTTCGGCCTGGAGTTGGCGCAGTGGCTGATACAGCGTGGGGTGCAGAAGCTCGTG
 5 gallus_FASN     88.9%
 ACATCATCACAGGGGGCCTAGGAGGATTTGGGCTTGAGTTGGCACAGTGGCTAATTGAGAGAGGAGCACAGAAGCTTGTA
 6 alligator_FASN  75.2%
 ACATCATCACAGGGGGCCTTGGCGGATTTGGGCTCGAATTGGCACACTGGCTTGCTGAGAGAGGAGCTCAGAAACTCGTG

                         5761          .         .         .         8         .         .
 .         . 5840
 1 ruby_FASN      100.0%
 CTGACATCTCGTTCTGGCATACGAACCGGCTACCAAGCTAGACGTGTTAGAGAGTGGAAGGCACTAGGAATCCAAGTGTT
 2 canna_FASN      99.7%
 CTGACATCTCGTTCTGGCATACGAACCGGCTACCAAGCTAGACGTGTTAGAGAGTGGAAGGCACTAGGAATCCAAGTGTT
 3 swift_FASN      90.8%
 CTGACCTCTCGTTCTGGCATACGAACTGGCTACCAGGCTAAACGTGTTAGAGAGTGGAAGGCACTAGGAATCCAAGTGTT
 4 human_FASN      64.1%
 TTGACTTCTCGCTCCGGGATCCGGACAGGCTACCAGGCCAAGCAGGTCCGCCGGTGGAGGCGCCAGGGCGTACAGGTGCA
 5 gallus_FASN     88.9%
 CTGACATCTCGATCTGGCATACGAACTGGCTACCAGGCTAAATGTGTTAGAGAATGGAAGGCGCTGGGAATCCAAGTGTT
 6 alligator_FASN  75.2%
 CTGACATCTCGTTCTGGCGTAAGAACTGGCTACCAAGCTAAACAAATCGGTCTATGGAAGGAGATGGGAGTCCAGGTCTT

                         5841          :         .         .         .         .         9
 .         . 5920
 1 ruby_FASN      100.0%
 GGTATCTACCAGTGATATTGGAACACTAGAAGGAGCACAGCGATTGATAGAAGAAGCCTTGCAGCTTGGCCCAGTTGGAG
 2 canna_FASN      99.7%
 GGTATCTACCAGTGATATTGGAACACTAGAAGGAGCACAGCGATTGATAGAAGAAGCCTTGCAGCTTGGCCCAGTTGGAG
 3 swift_FASN      90.8%
 GGTATCTACCAGTGATATTGGAACACTAGAAGGAGCACAGCAACTGATAGAAGAAGCTTTGCAGCTTGGCCCAGTTGGAG
 4 human_FASN      64.1%
 GGTGTCCACCAGCAACATCAGCTCACTGGAGGGGGCCCGGGGCCTCATTGCCGAGGCGGCGCAGCTTGGGCCCGTGGGCG
 5 gallus_FASN     88.9%
 GGTCTCTACCAGTGATGTTGGAACTCTAGAAGGAACGCAGCTTTTGATAGAAGAGGCTTTGAAGCTCGGACCAGTTGGGG
 6 alligator_FASN  75.2%
 GGTGTCAACCAGCGATGTTGGCACTTTAGAGGGAACACAGAAGTTACTAGATGAAGCGATACAGCTTGGACCAGTTGGGG

                         5921          .         .         :         .         .         .
 .         0 6000
 1 ruby_FASN      100.0%
 GCATCTTTAATTTGGCTGTGGTCCTTAGAGATGCCATGATTGAAAATCAGACCCCAGAATTATTTGTGGAGGTCAACAAG
 2 canna_FASN      99.7%
 GCATCTTTAATTTGGCTGTGGTCCTTAGAGATGCCATGATTGAAAATCAGACCCCAGAATTATTTGTGGAGGTCAACAAG
 3 swift_FASN      90.8%
 GCATCTTTAATTTGGCTGTGGTCCTTAGAGATGCCATGATTGAAAATCAGACTCCAGAATTATTCGGGGAGGTCAACAAG
 4 human_FASN      64.1%
 GCGTCTTCAACCTGGCCGTGGTCTTGAGAGATGGCTTGCTGGAGAACCAGACCCCAGAGTTCTTCCAGGACGTCTGCAAG
 5 gallus_FASN     88.9%
 GCATCTTTAATTTGGCTGTGGTCCTTAAAGATGCCATGATTGAAAATCAGACCCCGGAATTATTCTGGGAGGTCAACAAG
 6 alligator_FASN  75.2%
 GAATCTTTAACTTGGCTATGGTTCTCAGAGATGCCATGATGGAGAACCAGACACCAGAATCCTTCCAAGAGGTCTGCAAA

                         6001          .         .         .         .         :         .
 .         . 6080
 1 ruby_FASN      100.0%
 CCCAAGTATTCAGGCACCCTTCATTTGGATTGGGTGACTCGTAAGAAGTGTCCAGACCTGGACTATTTTGTTGTGTTCTC
 2 canna_FASN      99.7%
 CCCAAGTATTCAGGCACCCTTCATTTGGATTGGGTGACTCGTAAGAAGTGTCCAGACCTGGACTATTTTGTTGTGTTCTC
 3 swift_FASN      90.8%
 CCCAAGTATTCAGGCACCCTTCATTTGGATTGGGTGACTCGTAAGAAGTGTCCAGACCTGGACTATTTTGTTGTGTTCTC
 4 human_FASN      64.1%
 CCCAAGTACAGCGGCACCCTGAACCTGGACAGGGTGACCCGAGAGGCGTGCCCTGAGCTGGACTACTTTGTGGTCTTCTC
 5 gallus_FASN     88.9%
 CCCAAGTATTCAGGCACCCTTCATTTGGACTGGGTGACTCGTAAGAAGTGCCCAGACCTGGACTATTTTGTTGTATTCTC
```

```
   6 alligator_FASN  75.2%
   CCCAAGTCTGCAGGCATCATTCATCTGGACCGAGTGAGTCGTAAGAAGTGTCCGGACCTGGACTACTTTGTTGCTTTCTC

                            6081          .         1         .         .         .         .
   :         . 6160
   1 ruby_FASN      100.0%
   CTCTGTAAGCTGTGGAAGAGGAAATGCTGGACAAAGTAATTATGGTTTTGCCAATTCTACCATGGAGCGTATCTGTGAGC
   2 canna_FASN      99.7%
   CTCTGTAAGCTGTGGAAGAGGAAATGCTGGACAAAGTAATTATGGTTTTGCCAATTCTACCATGGAGCGTATCTGTGAGC
   3 swift_FASN      90.8%
   CTCTGTAAGCTGTGGAAGAGGAAATGCTGGGCAAAGTAATTATGGCTTTGCCAATTCTACCATGGAGCGTATCTGTGAGC
   4 human_FASN      64.1%
   CTCTGTGAGCTGCGGGCGTGGCAATGCGGGACAGAGCAACTACGGCTTTGCCAATTCCGCCATGGAGCGTATCTGTGAGA
   5 gallus_FASN     88.9%
   CTCTGTAAGCTGTGGAAGAGGAAATGCTGGGCAAAGTAATTATGGCTTTGCTAATTCTGCCATGGAGCGTATCTGTGAGC
   6 alligator_FASN  75.2%
   ATCTGTAAGCTCTGGAAGAGGGAATGCCGGACAGACTAACTATGGCTTTGCCAACTCCACCATGGAGCGTGTCTGTGAGC

                            6161          .         .         .         2         .         .
   .         . 6240
   1 ruby_FASN      100.0%
   AGCGGCATCATGATGGACTCCCCGGTCTGGCAATCCAGTGGGGAGCCATTGGTGATGTGGGTATCCTTCTGAAGACAATG
   2 canna_FASN      99.7%
   AGCGGCATCATGATGGACTCCCCGGTCTGGCAATCCAGTGGGGAGCCATTGGTGATGTGGGTATCCTTCTGAAGACAATG
   3 swift_FASN      90.8%      AGCGACATCATGATGGACTCCCAGGTATTCTCCTCCAG--
   -GCCTNNNNNNNNNNNNNNNNNNNNNNNNNNNNNNNNNNAATG
   4 human_FASN      64.1%
   AACGCCGGCACGAAGGCCTCCCAGGCCTGGCCGTGCAGTGGGGCGCCATCGGCGACGTGGGCATTTTGGTGGAGACGATG
   5 gallus_FASN     88.9%      AGCGGCATCACGATGGGCTCCCAGGCCTGGCAGTCCAGTGGGGAGCCATTGGTGATGTGGGCATC--
   -CTGAAGGCAATG
   6 alligator_FASN  75.2%
   AGCGGAAGCATGATGGGCTTCCAGCATTGTGTGTGCAGTGGGGTGTCGTTGGCGATGTGGGTGTCGTCATAAAGACACGG

                            6241          :         .         .         .         .         3
   .         . 6320
   1 ruby_FASN      100.0%
   GGAAACAAAGAGGTTGTGGTTGGGGGGACCATTCCCCAGCAAATCAGCTCATGCCTGGAGGTGCTCGATACCTTCCTGAA
   2 canna_FASN      99.7%
   GGAAACAAAGAGGTTGTGGTTGGGGGGACCATTCCCCAGCAAATCAGCTCATGCCTGGAGGTGCTCGATACCTTCCTGAA
   3 swift_FASN      90.8%
   GGAAACAAGGAGATTGTTGTTGGAGGAACCATTCCCCAGCCAATCAGTTCGTGCCTGGAAGTGCTTGATACCTTCCTGAA
   4 human_FASN      64.1%
   AGCACCAACGACACGATCGTCAGTGGCACGCTGCCCCAGCGCATGGCGTCCTGCCTGGAGGTGCTGGACCTCTTCCTGAA
   5 gallus_FASN     88.9%
   GGAAACAGGGAGGTTGTGATTGGGGGAACCGTTCTCCAGCAAATCAGCTCCTGCCTGGAGGTGCTCGATATGTTCCTGAA
   6 alligator_FASN  75.2%
   GGCAATGACAACACTGTGATTGGCGGGACCATCCCACAGCGAATTGCCTCGTGCCTGGAGGTTCTGGACCAGTTCCTGAA

                            6321          .         .         :         .         .         .
   .        4 6400
   1 ruby_FASN      100.0%
   TCAGCCTCATCCTGTCACATCCAGTTTTGTCCTAGCAGAGAAGGTCTCTGTGAAAGCTGAAGGAGGCAGTCAGCGGGATC
   2 canna_FASN      99.7%
   TCAGCCTCATCCTGTCACATCCAGTTTTGTCCTAGCAGAGAAGGTCTCTGTGAAAGCTGAAGGAGGCAGTCAGCGGGATC
   3 swift_FASN      90.8%
   TCAACCTCATCCTGTCACATCCAGTTTTGTCCTAGCAGAGAAGGTGTCTATGAAAACTGAAGGAGGCAGTCAACGGGATC
   4 human_FASN      64.1%
   CCAGCCCCACATGGTCCTGAGCAGCTTTGTGCTGGCTGAGAAGGCTGCGGCCTATAGGGACAGGGACAGCCAGCGGGACC
   5 gallus_FASN     88.9%
   TCAACCTCATCCTGTTATGTCCAGTTTTGTCCTAGCAGAGAAGGTCTCTGTGAAAAGTGAAGGCGGAAGTCAACGGGATC
   6 alligator_FASN  75.2%
   TCAGCCACATCCCATTGTGTCCAGCTTTGTCCTGGCAGAAAAGATTTCTACAAAGAGCGAAGGAAGTGGCCCTTGTGACC

                            6401          .         .         .         .         :         .
   .         . 6480
   1 ruby_FASN      100.0%      TGGTAGAAGCTGTTGCTCACATCCTGGGTG---TCCGTGATG-------
   -TGAGTAGTCTGAACGCTGATAGTTCCTTGG
   2 canna_FASN      99.7%      TGGTAGAAGCTGTTGCTCACATCCTGGGTG---TCCGTGATG-------
   -TGAGTAGTCTGAACGCTGATAGTTCCTTGG
   3 swift_FASN      90.8%
   TGGTAGAAGCTGTTGCTAACATCCTGGGTGAGTTCTCAGACAAGCACTACTGGGCAGTGTCTGCCATCTCAGTGCAGTGG
   4 human_FASN      64.1%      TGGTGGAGGCCGTGGCACACATCCTGGGCA---TCCGCGACT-------
   -TGGCTGCTGTCAACCTGGACAGCTCACTGG
   5 gallus_FASN     88.9%      TTGTAGAAGCTGTTGCTCATATCCTTGGTG---TTCGTGACG-------
   -TGAGCAGTCTGAATGCTGAGAGCTCCCTAG
   6 alligator_FASN  75.2%      TCGTGGAGGCCGTGGCCCACATCCTGGGTG---TCCGTGACT-------
   -TGAACAGTCTGAACGCTGAGAGCACCCTGG

                            6481          .         5         .         .         .         .
   :         . 6560
   1 ruby_FASN      100.0%      CAGA------
```

```
-CTTGGGCTTGGATTCCTTAATGGGTGTCGAGGTGCGCCAGACACTGGAGAGAGACTATGACATTGTTAT
2 canna_FASN      99.7%      CAGA------
-CTTGGGCTTGGATTCCTTAATGGGTGTCGAGGTGCGCCAGACACTGGAGAGAGACTATGACATTGTTAT
3 swift_FASN      90.8%
TGCTGTGTCCCTTTGGGCTTGGATTCCCTGATGGGTGTCGAGGTGCGCCAGACACTGGAGAGAGACTATGACATTGTTAT
4 human_FASN      64.1%      CGGA------
-CCTGGGCCTGGACTCGCTCATGAGCGTGGAGGTGCGCCAGACGCTGGAGCGTGAGCTCAACCTGGTGCT
5 gallus_FASN     88.9%      CAGA------
-CTTGGGCCTGGATTCCTTGATGGGTGTGGAGGTGCGCCAGACGCTGGAGAGAGACTATGACATCGTAAT
6 alligator_FASN  75.2%      CCGA-------
TCTGGGCTTGGACTCCCTCATGGGCGTGGAGATCCGCCAGACCCTGGAGAGGGACTATGACATCATCAT

                        6561          .         .         .         6         .         .
.         . 6640
1 ruby_FASN      100.0%
GACCATGAGAGAAATTAGACTCCTTACAATCAACAAGCTGCGTGAACTTTCTTCCAAGTCTGGGACAACAGAGGAGCTGA
2 canna_FASN      99.7%
GACCATGAGAGAAATTAGACTCCTTACAATCAACAAGCTGCGTGAACTTTCTTCCAAGTCTGGGACAACAGAGGAGCTGA
3 swift_FASN      90.8%
GACCATGAGAGAAATTCGTCTCCTTACAATCAACAAACTGCGTGAACTTTCTTCCAAGTCCAGGACAACAGAGGAGCTGA
4 human_FASN      64.1%
GTCCGTGCGCGAGGTGCGGCAACTCACGCTCCGGAAACTGCAGGAGCTGTCCTCAAAGGCGGATGAGGCCAGCGAGCTGG
5 gallus_FASN     88.9%
GACCATGAGGGAGATCCGACTCCTCACCATCAACAAACTGCGTGAACTGTCCTCCAAGACTGGGACAGCAGAGGAGCTGA
6 alligator_FASN  75.2%
GCCGATGAGAGAGATTCGAGGGCTTACCATTAACAAACTGCGGGAGCTTTCCACCAAATCTGGAACAGCAGAGGAGCTGA

                        6641          :         .         .         .         .         7
.         . 6720
1 ruby_FASN      100.0%      AACCATCCCAAGTGATGAAGACAGGTGCTGGCGAACCTCCAA--
-AACTAGATTTGAACAACTTGTTGGTGAACCCAGAA
2 canna_FASN      99.7%      AACCATCCCAAGTGATGAAGACAGGTGCTGGCGAACCTCCAA--
-AACTAGATTTGAACAACTTGTTGGTGAACCCAGAA
3 swift_FASN      90.8%      AGCCATCTCAAGTGATGAAGACAGGCATGGGCGAACTTCCAA--
-AACTGGATTTGAACAACTTATTGGTGAGCCCTGAA
4 human_FASN      64.1%
CATGCCCCACGCCCAAGGAGGATGGTCTGGCCCAGCAGCAGACTCAGCTGAACCTGCGCTCCCTGCTGGTGAACCCGGAG
5 gallus_FASN     88.9%      AGCCATCACAAGTGTTGAAGACAGGCCCAGGTGAGCCTCCAA--
-AACTGGATTTGAACAACTTGCTGGTGAATCCAGAA
6 alligator_FASN  75.2%      TGCCATTTAA---GACTACAGCAGGCCAGGCCAAGCACCCAG--
-AACTGAACCTGAACAACCTGGTGCTGAATCCCGAG

                        6721          .         .         :         .         .         .
.        8 6800
1 ruby_FASN      100.0%
GGGCCAACGATCACCCGTATCAACGATGTTCAGAGCACTGAGCGCCCTCTTTTCCTTGTTCACCCCATTGAAGGATCCAC
2 canna_FASN      99.7%
GGGCCAACGATCACCCGTATCAACGATGTTCAGAGCACTGAGCGCCCTCTTTTCCTTGTTCACCCCATTGAAGGATCCAC
3 swift_FASN      90.8%
GGACCAACAATCACCCGTCTCAATGATGTTCAGAGCACAGAGCGTCCTCTTTTCTTTGTTCACCCCATTGAAGGATCCAC
4 human_FASN      64.1%
GGCCCCACCCTGATGCGGCTCAACTCCGTGCAGAGCTCGGAGCGGCCCCTGTTCCTGGTGCACCCAATCGAGGGCTCCAC
5 gallus_FASN     88.9%
GGACCAACGATTACCCGTCTCAATGAAGTTCAGAGCACAGAACGCCCTCTTTTCCTTGTTCACCCCATTGAGGGATCCAT
6 alligator_FASN  75.2%
GGGCCGACCGTCACCCGACTCAACGAGGTGCAAAGCACGGAGCGCCCGCTATTCCTCATCCACCCCATCGAGGGATCCGC

                        6801          .         .         .         .         :         .
.         . 6880
1 ruby_FASN      100.0%
TGCTGTTTTCAAAACTCTTGCCTCCAAACTTCATATGCCCTGCTATGGGTTCCAGTGCACAAAAGTTGCTCCCTTGGACA
2 canna_FASN      99.7%
TGCTGTTTTCAAAACTCTTGCCTCCAAACTTCATATGCCCTGCTATGGGTTCCAGTGCACAAAAGTTGCTCCCTTGGACA
3 swift_FASN      90.8%
TGCAGTTTTCCACACTCTTGCCTCCAGACTTCATATGCCCTGCTATGGGTTCCAGTGCACAAAAGCTGCTCCCTTGGACA
4 human_FASN      64.1%
CACCGTGTTCCACAGCCTGGCCTCCCGGCTCAGCATCCCCACCTATGGCCTGCAGTGCACCCGAGCTGCGCCCCTTGACA
5 gallus_FASN     88.9%
TGCAGTCTTCTATACTCTTGCCTCCAAACTTCATATGCCCTGCTATGGACTCCAGTGCACAAAAGCTGCTCCTTTGGACA
6 alligator_FASN  75.2%
TGCCGTGTTCCAGGCCCTGGCCTCCAGGCTCAGAATCCCCAGCTACGGGCTCCAGTGCACACGAGCTGCTCCCTTGGACA

                        6881          .         9         .         .         .         .
:         . 6960
1 ruby_FASN      100.0%
GCATACAGAGCCTGGCAACCTATTACATTAACTGCATGAAGCAGATACAGCCTGAAGGACCTTACCGCATTGCTGGATAT
2 canna_FASN      99.7%
GCATACAGAGCCTGGCAACCTATTACATTAACTGCATGAAGCAGATACAGCCTGAAGGACCTTACCGCATTGCTGGATAT
3 swift_FASN      90.8%
GCATACAGAGCCTGGCAAGCTATTACCTTGGCTATATGAAGCAGATACAGCCTGAAGGACCTTACCGCATTGCTGGATAT
4 human_FASN      64.1%
```

```
GCATCCACAGCCTGGCTGCCTACTACATCGACTGCATCAGGCAGGTGCAGCCCGAGGGCCCCTACCGCGTGGCCGGCTAC
5 gallus_FASN     88.9%
GCATACAGAGCCTGGCATCCTATTATATTGACTGTATGAAGCAGATACAGCCTGAAGGACCTTATCGCATTGCTGGATAC
6 alligator_FASN  75.2%
GCATAAAGAGCCTGGCAGCCTACTACATTGACTGCATGAAGCAGGTGCAGCCGGAGGGACCCTACCGCATTGCTGGATAC

                        6961          .         .         .         0         .         .
.         . 7040
1 ruby_FASN      100.0%      TCTTTCGGTGCCTGTGTAGCCTTTGAAATGTGCTCCCAACTGCAAGC--
-ACAACATAAAGGTTCCCATGCGCTCAACAG
2 canna_FASN      99.7%      TCTTTCGGTGCCTGTGTAGCCTTTGAAATGTGCTCCCAACTGCAAGC--
-ACAACATAAAGGTTCCCATGCGCTCAACAG
3 swift_FASN      90.8%      TCCTTTGGTGCCTGTGTAGCCTTTGAAATGTGCTCCCAGCTGCAAGC--
-ACAACAAAATGCTTCCCATTCGCTCAACAG
4 human_FASN      64.1%      TCCTACGGGGCCTGCGTGGCCTTTGAAATGTGCTCCCAGCTGCAGGC---
CCAGCAGAGCCCAGCCCCCACCCACAACAG
5 gallus_FASN     88.9%      TCTTTTGGTGCCTGCGTAGCCTTTGAAATGTGCTCCCAGCTGCAAGC--
-ACAACAAAATGCTTCCCATGCACTCAACAG
6 alligator_FASN  75.2%
TCCTTTGGAGCCTGCGTCGCCTTTGAGATGTGCTCCCAGCTGCAAGCCCAGCAGCAGAACCCCTCCCTGGGGTCCAACAG

                        7041          :         .         .         .         .         1
.         . 7120
1 ruby_FASN      100.0%
TCTATTCCTCTTCGATGGATCTCATTCCTTTGTGGCAGCATACACTCAGAGATACAGAGCCAAGCTAAGCCAAGGAAATG
2 canna_FASN      99.7%
TCTATTCCTCTTTGATGGATCTCATTCCTTTGTGGCAGCATACACTCAGAGATACAGAGCCAAGCTAAGCCAAGGAAATG
3 swift_FASN      90.8%
TCTGTTCCTCTTTGATGGGTCTCATTCCTTTGTGGCAGCTTACACTCGGAGCTACAGAGCCAAGCTGAGCCAAGGAAATG
4 human_FASN      64.1%
CCTCTTCCTGTTCGACGGCTCGCCCACCTACGTACTGGCCTACACCCAGAGCTACCGGGCAAAGCTGACCCCAGGCTGTG
5 gallus_FASN     88.9%
TTTATTCCTCTTTGATGGGTCTCATTCCTTTGTGGCAGCATACACTCAGAGCTACAGAGCAAAGCTGACCCAAGGAAATG
6 alligator_FASN  75.2%
CCTCCTCCTGTTCGACGGCTCCCACTCCTACGTGGCTGCGCACACTCAGATCTACAAAGCCAAGATGGCCCTGGGAAGTG

                        7121          .         .         :         .         .         .
.         2 7200
1 ruby_FASN      100.0%
AGGCTGCATTGGAGACTGAAGCATTGTGTGCCTTTGTTCAGCAATTTACAGGCATTGAATACAATAAGCTGCTGGAGATT
2 canna_FASN      99.7%
AGGCTGCATTGGAGACTGAAGCATTGTGTGCCTTTGTTCAGCAATTTACAGGCATTGAATACAATAAGCTGCTGGAGATT
3 swift_FASN      90.8%
AGGCTGCCTTGGAGACTGAAGCATTGTGTGCCTTTGTTCAGCAATTTACAGGCATTGAATACAATAAGCTGCTGGAGATT
4 human_FASN      64.1%
AGGCTGAGGCTGAGACGGAGGCCATATGCTTCTTCGTGCAGCAGTTCACGGACATGGAGCACAACAGGGTGCTGGAGGCG
5 gallus_FASN     88.9%
AGGCTGCGTTGGAGACAGAAGCACTGTGTGCCTTTGTTCAGCAGTTTACAGGCATTGAATACAATAAGTTGTTGGAGATT
6 alligator_FASN  75.2%
AGGCCGAGGCAGAGGCTGCAGCAATGCGTGCCTTTGTTCAGCAGTTCACAGGCACCGAATACAATAAGTTGCTGGAGACT

                        7201          .         .         .         .         :         .
.         . 7280
1 ruby_FASN      100.0%
CTCCTGCCCCTGAAAGATCTGGAGGCTCGTGTGAATGCTGCTGCTGACCTGATAACTCAGGTTCATACACACATCAATCG
2 canna_FASN      99.7%
CTCCTGCCCCTGAAAGATCTGGAGGCTCGTGTGAATGCTGCTGCTGACCTGATAACTCAGGTTCATACACACATCAATCG
3 swift_FASN      90.8%
CTTCTGCCCCTGAAAGATCTGGAGGCTCGTGTGAATGCTGCTGCAGACCTGATAACTCAAGTTCATACACACATCAATCG
4 human_FASN      64.1%
CTGCTGCCGCTGAAGGGCCTAGAGGAGCGTGTGGCAGCCGCCGTGGACCTGATCATCAAGAGCCACCAGGGCCTGGACCG
5 gallus_FASN     88.9%
CTTCTGCCCTTGGAAGATCTGGAGGCTCGTGTCAATGCTGCTGCAGACCTTATAACTCAGATTCATAAAAACATCAACCG
6 alligator_FASN  75.2%
CTGCTGCCCCTGCAAGACTTCGATGCTCGTGTCAGTGCTTCTGTGGACCTAATAGCTCGGATTCACAGAGACCTCAATCG

                        7281          .         3         .         .         .         .
:         . 7360
1 ruby_FASN      100.0%
TGAAGCACTCTGCTTTGCTGCTACTTCCTTTTACCATAAACTGAAGGCTGCCGACAAGTATATACCCGAGTCCAAGTATC
2 canna_FASN      99.7%
TGAAGCACTCTGCTTTGCTGCTACTTCCTTTTACCATAAACTGAAGGCTGCCGACAAGTATATACCCGAGTCCAAGTATC
3 swift_FASN      90.8%
TGAAGCACTCAACTTTGCTGCTGCTTCCTTTTTCCACAAACTGAAAGCTGCTGACAAGTATATACCAGAGTTCAAGTACC
4 human_FASN      64.1%
CCAGGAGCTGAGCTTTGCGGCCCGGTCCTTCTACTACAAGCTGCGTGCCGCTGAGCAGTACACACCCAAGGCCAAGTACC
5 gallus_FASN     88.9%
TGAAGCACTCAGCTTTGCTGCTGCTTCCTTTTACCATAAGCTGAAGGCTGCTGACAAGTATATACCAGAATCCAAGTATC
6 alligator_FASN  75.2%
TGAGGCCCTCAGCTTTGCTGCTGCCTCCTTCTACCACAAACTGAAGGCTGCCGACAAGTATGTACCAGAGTTCAAGTACC
```

```
                           7361          .         .         .         4         .         .
.         . 7440
1 ruby_FASN      100.0%
ATGGGAATGTGACACTAGTGCGGGCTAAGTCTCACAATGAATATGAAGAAGGTCTGGGTGGAGACTACCGACTCTCGGAG
2 canna_FASN      99.7%
ATGGGAATGTGACGCTAGTGCGGGCTAAGTCTCACAATGAATATGAAGAAGGTCTGGGTGGAGACTACCGACTCTCGGAG
3 swift_FASN      90.8%
ACGGGAATGTGACACTGATGCGGGCCAAGTCTCACAATGAATATGAAGAAGGTCTGGGTGAAGACTACAGACTCTCAGAG
4 human_FASN      64.1%
ATGGCAACGTGATGCTACTGCGCGCCAAGACGGGTGGCGCCTACGGCGAGGACCTGGGCGCGGACTACAACCTCTCCCAG
5 gallus_FASN     88.9%
ATGGGAACGTGACACTGATGCGGGCAAAGACTCACAATGAGTATGAAGAAGGTCTGGGTGGAGACTACAGACTCTCAGAG
6 alligator_FASN  75.2%
ATGGCAATGTGACGCTGCTGAGAACCAAGATGAACGATGACTATGGAGACGGCCTGGGTGGAGATTACAAACTCTCAGAG

                           7441          :         .         .         .         .         5
.         . 7520
1 ruby_FASN      100.0%
GTATGCGATGGGAAAGTATCTGTCCACGTCGTTGAAGGGGATCACCGCACTATGTTGGAGGGAAATGGCGCTGAATCAAT
2 canna_FASN      99.7%
GTATGCGATGGGAAAGTATCTGTCCACGTCGTTGAAGGGGATCACCGCACTATGTTGGAGGGAAATGGCGCTGAATCAAT
3 swift_FASN      90.8%
GTATGTGATGGGAAAGTCTCTGTCCACGTTGTCGAAGGGGATCACCGCACCCTATTGGAGGGAGATGGTGCTGAATCAAT
4 human_FASN      64.1%
GTATGCGACGGGAAAGTATCCGTCCACGTCATCGAGGGTGACCACCGCACGCTGCTGGAGGGCAGCGGCCTGGAGTCCAT
5 gallus_FASN     88.9%
GTATGCGATGGAAAAGTATCAGTCCACATCATTGAAGGAGATCACCGCACCTTATTGGAGGGAGATGGTGTTGAATCAAT
6 alligator_FASN  75.2%
GTCTGTGATGGGAAAATCTCAGTCCATGTCATCGAAGGAGATCACCGCACCTTCCTGGAGGCAGACGGCATTGAGACTGT

                           7521          .         .         :         .         .         ] 7580
1 ruby_FASN      100.0%      TATTGGCATCATCCACAGCTCACTGGCAGAACCACGTGTCAGTGTCAGAGAGGGTTA---
2 canna_FASN      99.7%      TATTGGGATCATCCACAGCTCACTGGCAGAACCACGTGTCAGTGTCAGAGAGGGTTAA--
3 swift_FASN      90.8%      TATTGGGATCATCCACAGCTCACTGGCAGAACCACGTGTCAGTGTCAGGGAGGGTTAA--
4 human_FASN      64.1%      CATCAGCATCATCCACAGCTCCCTGGCTGAGCCACGCGTGAGCGTGCGGGAGGGCTAG--
5 gallus_FASN     88.9%      CATTGGGATCATCCATGGCTCACTGGCAGAGCCACGTGTCAGTGTCAGAGAAGGTTAA--
6 alligator_FASN  75.2%      TGTCGGGATCATCCACAGCTCGCTGGCAGAGCCCCGGGTCAGCGTCCGAGAGGGTTAG--
```

MView 1.60.1, Copyright © 1997-2015 Nigel P. Brown

```
Reference sequence (1): ruby_GPAM
Identities normalised by aligned length.
Colored by: identity + property


                              1 [        .         .         .         .         :         .
.         . 80
1 ruby_GPAM      100.0%
ATGTGTGTGGATACGTGTTTCTGTGTGTGTGTATATGTATGTTATTATTTTTCAGCTTCTGATCTTCACTTCAACTTGTG
2 canna_GPAM      99.6%      ---------------------------------------------------------------------
-----------
3 swift_GPAM      94.4%      ---------------------------------------------------------------------
-----------
4 human_GPAM      76.1%      ---------------------------------------------------------------------
-----------
5 gallus_GPAM     92.3%      ---------------------------------------------------------------------
-----------
6 alligator_GPAM  83.5%      ---------------------------------------------------------------------
-----------

                             81          .         1         .         .         .         .
:         . 160
1 ruby_GPAM      100.0%
TGTCATGGATGAAACTGCTTTGTCCCTTGGAACAATAGATGTTTCCTATTTGTCCACCTCAGCAGAGTGCAGTATCAGTA
2 canna_GPAM      99.6%      ---
-ATGGATGAAACTGCTTTGTCCCTTGGAACAATAGATGTTTCCTATTTGTCCACCTCAGCAGAGTGCAGTATCAGTA
3 swift_GPAM      94.4%      ---
-ATGGATGAAACTGCTTTGTCCCTTGGAACAATAGATGTTTCCTATTTGTCCACCTCAGCAGAGTGCGGTATCAGTA
4 human_GPAM      76.1%      ---
-ATGGATGAATCTGCACTGACCCTTGGTACAATAGATGTTTCTTATCTGCCACATTCATCAGAATACAGTGTTGGTC
5 gallus_GPAM     92.3%      ---
-ATGGATGAAACTGCTTTGTCGCTTGGAACAATAGATGTTTCTTATTTGTCCACCTCCGCAGAGTGCAGTGTCAGTA
6 alligator_GPAM  83.5%      ---
-ATGGATGAGGCGGCTCTGTCCCTGGGAACAATAGATGTTTCCTATTTGTCCACTTCCACGGAATACAGTGTGGGGA

                            161          .         .         .         2         .         .
.         . 240
```

```
 1 ruby_GPAM      100.0%
 GATGTAAGCATTCCAGTGAAGAATGGGGAGAGTGTAACTCTAGGCCCACTCTCTTCAGATCTGCCACTTTAAGATGGAAG
 2 canna_GPAM      99.6%
 GATGTAAGCATTCCAGTGAAGAATGGGGAGAGTGTAACTCTAGGCCCACTCTCTTCAGATCTGCCACTTTAAAATGGAAG
 3 swift_GPAM      94.4%
 GATGTAAGCATTCCAGTGAGGAATGGGGGGAGTGTAACTCTAGGCCCCTCTCTTCNNNNNNNNNNNTTTCAAATGGAAA
 4 human_GPAM      76.1%
 GATGTAAGCACACAAGTGAGGAATGGGGTGAGTGTGGCTTTAGACCCACCATCTTCAGATCTGCAACTTTAAAATGGAAA
 5 gallus_GPAM     92.3%
 GATGTAAGCATTCCAATGAAGAATGGGGAGAGTGTAACTCTAGGCCTACTCTCTTCCGATCTGCCACTTTAAAATGGAAG
 6 alligator_GPAM  83.5%
 GATGTAAGCACCCCAGTGAAGAGTGGGGAGAGTGCAATTCCAGGCCTACCGTCTTTCGATCTGCCACTTTAAAATGGAAG

                           241          :         .         .         .         .         3
 .         . 320
 1 ruby_GPAM      100.0%
 GAGGCTCTACTGAGCAGAAAAAGGCCATTTGTGGGGCGATGCTGCTACGTGTGCACTCCCCAGAGCCGGGATAACTTCTT
 2 canna_GPAM      99.6%
 GAGGCTCTGCTGAGCAGAAAAAGGCCATTTGTGGGGCGATGCTGCTACGTGTGCACTCCCCAGAGCCGGGATAACTTCTT
 3 swift_GPAM      94.4%
 GAGACTCTGCTGAGCCGGAAAAGGCCATTCGTGGGACGATGTTGCTATGTGTGCACTCCCCAGAGCCGGGATAACTTCTT
 4 human_GPAM      76.1%
 GAAAGCCTAATGAGTCGGAAAAGGCCATTTGTTGGAAGATGTTGTTACTCCTGCACTCCCCAGAGCTGGGACAAATTTTT
 5 gallus_GPAM     92.3%      GAGACT--
 -CTGAGCAGAAAAAGGCCGTTTGTGGGACGATGTTGCTACGTATGCACTCCCCAGAGCCGGGATAACTTCTT
 6 alligator_GPAM  83.5%
 GAGACCCTGCTAAGCAGAAAAAGGCCATTTGTGGGGCGCTGTTGCTATGTGTGCACTCCTCAAAGCCGGGATAAGCTCTT

                           321          .         .         :         .         .         .
 .         4 400
 1 ruby_GPAM      100.0%
 CAATGCAAGTATTCCTTCTTTGGGTCTACGTAATGTCATATACATCAATGAAACACATACAAGGTACAGAGGGTGGCTTG
 2 canna_GPAM      99.6%
 CAATGCAAGTATTCCTTCTTTGGGTCTACGTAATGTCATATACATCAATGAAACACATACAAGGTACAGAGGGTGGCTTG
 3 swift_GPAM      94.4%
 CAATGCCAGTATTCCTTCCCTGGGTCTACGTAATGTCATATATATCAATGAAACGCATACAAGGTACAGAGGGTGGCTTG
 4 human_GPAM      76.1%
 CAACCCCAGTATCCCGTCTTTGGGTTTGCGGAATGTTATTTATATCAATGAAACTCACACAAGACACCGCGGATGGCTTG
 5 gallus_GPAM     92.3%
 CAATGCTAGTATTCCTTCTTTGGGTCTACGTAATGTGATATATATCAATGAAACACACACCAGGTACAGGGGGTGGCTTG
 6 alligator_GPAM  83.5%
 CAATGCTAGTATTCCTTCTTTGGGCTTACGCAATGTCATCTACATCAATGAAACACACACAAGGTACAGAGGGTGGCTCG

                           401          .         .         .         .         :         .
 .         . 480
 1 ruby_GPAM      100.0%
 CAAGACGCCTTTGTTATGTCCTTTTTGTGCAAGAGAGAGATGTTCACAAGGGTATGTTTGCCAAGAATCTGACAGAAAAT
 2 canna_GPAM      99.6%
 CAAGACGCCTTTGTTATGTCCTTTTTGTGCAAGAGAGAGATGTTCACAAGGGTATGTTTGCCAAGAATCTGACAGAAAAT
 3 swift_GPAM      94.4%
 CAAGACGCCTTTGTTATGTCCTTTTTGTGCAAGAGAGGGATGTTCACAAGGGTATGTTTGCCAAGAATCTGACAGAAAAT
 4 human_GPAM      76.1%
 CAAGACGCCTTTCTTACGTTCTTTTTATTCAAGAGCGAGATGTGCATAAGGGCATGTTTGCCACCAATGTGACTGAAAAT
 5 gallus_GPAM     92.3%
 CAAGACGACTTTGTTATGTTCTTTTTGTGCTAGAGAGGGATGTTCATAAGGGAATGTTTGCCAAGAACCTGACAGAAAAT
 6 alligator_GPAM  83.5%
 CAAGACGTCTCTGTTACGTTCTCTTTGTGCAAGAACGTGATGTGCATAAGGGTATGTTTGCCAAGAATCTGACGGAAAAT

                           481          .         5         .         .         .         .
 :         . 560
 1 ruby_GPAM      100.0%
 GTGCTGAATAACAGCAGAGTCCAGAAGGCCATTGTAGATGAAGCTTCTGAACCAAGTACTCCAGGCAGTTTTGCTCAGAC
 2 canna_GPAM      99.6%
 GTGCTGAATAACAGCAGAGTCCAGAAGGCCATTGTAGATGAAGCTTCTGAACCAAGTACTCCAGGCAGTTTTGCTCAGAC
 3 swift_GPAM      94.4%
 GTGCTGAATAACAGCAGAGTCCAGAAGGCCATTGTAGATGAAGCTTCTGAACCAAGTACTCCAGGTAGTTTTGCTCAGAC
 4 human_GPAM      76.1%      GTGCTGAACAGCAGTAGAGTACAAGAGGCAATTGCAGAAGTGGCTGCTGAATTAAAC--
 -CCTGATGGTTCTGCCCAGCA
 5 gallus_GPAM     92.3%
 GTGCTGAATAGCAGTAGGGTACAGAAGGCCATCGTAGATGAAGCTTCTGAGCCAAGTGTTCCAGGTAGCTTTGCTCAGAT
 6 alligator_GPAM  83.5%
 GTTTTAAACAGTGGCAGAGTGCAGAAGGCCATTGTAGAGGAGGCTACTGAAACAAGTACATCAGGGAGCTTCGCTCAGGT

                           561          .         .         .         6         .         .
 .         . 640
 1 ruby_GPAM      100.0%
 AGATCCTAAAGCTATCAGCAAAGTGAAGAAAAAAGCTAGGAAGATTCTCCAGGAAATGGTAGCAAATGTGTCACCTGCTT
 2 canna_GPAM      99.6%
 AGATCCTAAAGCTATCAGCAAAGTGAAGAAAAAAGCTAGGAAGATTCTCCAGGAAATGGTAGCAAATGTGTCACCTGCTT
 3 swift_GPAM      94.4%
 GGATCCTAAAGCTATCAACAAAGTGAGGAAAAAAGCTAGGAAGATTCTCCAGGAAATGGTAGCAAATGTGTCACCTGCTT
```

```
4 human_GPAM      76.1%
GCAATCAAAAGCCGTTAACAAAGTGAAAAAGAAAGCTAAAAGGATTCTTCAAGAAATGGTTGCCACTGTCTCACCGGCAA
5 gallus_GPAM     92.3%
GGATCCTAAGGCTATCAACAAAGTGAAAAAAAAGGCTAGGAAAATTCTCCAGGAAATGGTAGCAAATGTGTCACCGGCTT
6 alligator_GPAM  83.5%
GGACCCCAAGGCCATCAGCAAGGTGAAGAAGAAAGCAAGGAAGATTCTGCAGGAAATGGTAGCAACTGTCTCACCTGCTT

                         641          :         .         .         .         .         7
.         . 720
1 ruby_GPAM      100.0%
TAATCAGGTTGACTGGCTGGGTGTTACTGAAGTTATTTAACAGCTTTTTCTGGAATATTCAGATTCACAGAGGTCAAATA
2 canna_GPAM      99.6%
TAATCAGGTTGACTGGCTGGGTGTTACTGAAGTTATTTAACAGCTTTTTCTGGAATATTCAGATTCACAGAGGTCAAATA
3 swift_GPAM      94.4%
TAATCAGGTTGACTGGCTGGGTGTTACTGAAGTTATTTAACAGCTTTTTCTGGAATATTCAGATTCACAGAGGTCAAATA
4 human_GPAM      76.1%
TGATCAGACTGACTGGGTGGGTGCTGCTAAAACTGTTCAACAGCTTCTTTTGGAACATTCAAATTCACAAAGGTCAACTT
5 gallus_GPAM     92.3%
TAATCAGGTTGACCGGCTGGGTGTTACTGAAGTTGTTTAACAGCTTTTTCTGGAATATTCAGATCCACAGAGGTCAAATA
6 alligator_GPAM  83.5%
TGATCAGGTTGACTGGTTGGGTGCTGCTGAAATTATTTAACAGCTTCTTTTGGAACGTTCAGATCCACAAAGGTCAACTG

                         721          .         .         :         .         .         .
.        8 800
1 ruby_GPAM      100.0%
GAAATGGTCAAAGCAGCAACAGAGATGAATTTGCCTCTTATCTTCCTGCCTGTTCACAAATCCCACATTGACTACCTGCT
2 canna_GPAM      99.6%
GAAATGGTCAAAGCAGCAACAGAGATGAATTTGCCTCTTATCTTCCTGCCTGTTCACAAATCCCACATTGACTACCTGCT
3 swift_GPAM      94.4%
GAAATGGTCAAAGCAGCAACAGAGATGAATTTGCCTCTTATCTTCTTGCCTGTTCACAAATCCCACATTGACTACCTGCT
4 human_GPAM      76.1%
GAGATGGTTAAAGCTGCAACTGAGACGAATTTGCCGCTTCTGTTTCTACCAGTTCATAGATCCCATATTGACTATCTGCT
5 gallus_GPAM     92.3%
GAAATGGTCAAAGCAGCAACAGAGATGAATTTGCCTCTTATTTTCTTGCCTGTTCATAAATCTCACATTGATTACCTGCT
6 alligator_GPAM  83.5%
GAAATGGTCAAAGCCGCAACTGAGATGAATTTACCTCTCATCTTCTTGCCTGTTCACAAATCTCACATTGACTACCTGCT

                         801          .         .         .         .         :         .
.         . 880
1 ruby_GPAM      100.0%
TCTTACATTCATTCTTTTCTGCCATAACATCAAAGCACCCTACATTGCTGCAGGGAACAATCTCAACATCCCCATCTTCA
2 canna_GPAM      99.6%
TCTTACATTCATTCTTTTCTGCCATAACATCAAAGCACCCTACATTGCTGCAGGGAACAATCTAAACATCCCCATCTTCA
3 swift_GPAM      94.4%
CCTTACATTCATTCTTTTCTGCCATAACATCAAAGCACCCTACATTGCTGCAGGGAACAATCTCAACATCCCCATCTTCA
4 human_GPAM      76.1%
GCTCACTTTCATTCTCTTCTGCCATAACATCAAAGCACCATACATTGCTTCAGGCAATAATCTCAACATCCCAATCTTCA
5 gallus_GPAM     92.3%
CCTTACATTCATACTTTTCTGCCATAACATCAAAGCACCCTATATTGCTGCAGGGAATAATCTCAACATCCCCATCTTCA
6 alligator_GPAM  83.5%
CCTCACATTCATTCTTTTCTGCCATAACATCAAAGCACCATACATTGCTGCAGGGAATAACCTCAATATCCCCATCTTCA

                         881          .         9         .         .         .         .
:         . 960
1 ruby_GPAM      100.0%
GCACATTGATCCGCAAGCTGGGAGGATTTTTCATTCGTCGAAAGTTAGATCAGAGTCCTGATGGTCGTAAGGACTTCCTG
2 canna_GPAM      99.6%
GCACATTGATCCGCAAGCTGGGAGGATTTTTCATTCGTCGAAAGTTAGATCAGAGTCCTGATGGTCGTAAGGACTTCCTG
3 swift_GPAM      94.4%
GCACCTTGATCCGCAAGCTGGGAGGATTTTTCATTCGTCGAAAGTTAGATCAGAGTCCTGATGGCCGTAAGGATTTCCTC
4 human_GPAM      76.1%
GTACCTTGATCCATAAGCTTGGGGGCTTCTTCATACGACGAAGGCTCGATGAAACACCAGATGGACGGAAAGATGTTCTC
5 gallus_GPAM     92.3%
GCACACTGATCCGTAAGCTGGGAGGATTTTTCATTCGTCGAAAGTTGGATCAGAGTTCTGATGGTCGTAAGGACTTCCTC
6 alligator_GPAM  83.5%
GCACGTTAATCCGCAACCTTGGAGGCTTTTTTATTCGGCGGAAGTTGGATGAGAACACTAATGGACGTAAAGATGCCCTA

                         961          .         .         .         0         .         .
.         . 1040
1 ruby_GPAM      100.0%
TACAGAGCTTTACTCTATGTGCACATAGAAGAATTGTTGAGACAGCAGCAGTTTTTAGAAATATTCTTGGAAGGCACACG
2 canna_GPAM      99.6%
TACAGAGCTTTACTCTATGTGCACATAGAAGAATTGTTGAGACAGCAGCAGTTTTTAGAAATATTCTTAGAAGGCACACG
3 swift_GPAM      94.4%
TACAGAGCATTGCTCTATGTGCACATAGAAGAACTGTTGAGACAGCAACAGTTTTTAGAGATATTCTTGGAAGGCACACG
4 human_GPAM      76.1%
TATAGAGCTTTGCTCCATGGGCATATAGTTGAATTACTTCGACAGCAGCAATTCTTGGAGATCTTCCTGGAAGGCACACG
5 gallus_GPAM     92.3%
TACAGAGCTTTGCTGTATGTGCACATAGAAGAATTGTTGAGACAGCAGCAGTTTTTAGAAATATTCTTGGAAGGCACACG
6 alligator_GPAM  83.5%
TATAGAGCATTACTCTATGTGCACATTGAGGAATTGCTCAGACAGCAGCAGTTCCTAGAGATATTCCTGGAAGGTACACG
```

```
                        1041          :         .         .         .         .         1
.        . 1120
1 ruby_GPAM      100.0%
ATCGCGAAGTGGAAAGACAGCTGGAGCTAGAGCAGGTCTTTTATCTGTGGTGGTGGATGCTCTCTTCTCAAATGCTACAC
2 canna_GPAM      99.6%
ATCGCGAAGTGGAAAGACAGCTGGAGCTAGAGCAGGTCTTTTATCTGTGGTGGTGGATGCTCTCTTCTCAAATGCTACAC
3 swift_GPAM      94.4%
ATCTCGAAGTGGGAAGACATCTGGAGCTAGAGCAGGTCTTTTATCTGTGGTGGTGGATGCTCTCTTCTCAAATGCTACTC
4 human_GPAM      76.1%
TTCTAGGAGTGGAAAAACCTCTTGTGCTCGGGCAGGACTTTTGTCAGTTGTGGTAGATACTCTGTCTACCAATGTCATCC
5 gallus_GPAM     92.3%
GTCTCGAAGTGGAAAGACATCCAGTCCTAGAGCGGGTCTTTTATCTGTGGTAGTGGATGCTCTCTTCTCAAATGCTACTC
6 alligator_GPAM  83.5%
GTCTCGCAGTGGAAAAACATCCAATGCCAGAGCAGGTCTCCTGTCTGTGGTTGTAGATGCTCTTTTTGCAAATGCCACCC

                        1121          .         .         :         .         .         .
.        2 1200
1 ruby_GPAM      100.0%
CTGATGTCCTAATTATACCTGTGGGAATCTCCTATGATCGCATAATTGAAGGTCACTATAACAGTGAACAGCTGGGCAAG
2 canna_GPAM      99.6%
CTGATGTCCTAATTATACCTGTGGGAATCTCCTATGATCGCATAATTGAAGGTCACTATAACAGTGAACAGCTGGGCAAG
3 swift_GPAM      94.4%
CTGACGTCCTAATTATACCTGTGGGAATCTCCTATGATCGCATAATTGAAGGTCACTACAACAGTGAGCAGCTGGGCAAA
4 human_GPAM      76.1%
CAGACATCTTGATAATACCTGTTGGAATCTCCTATGATCGCATTATCGAAGGTCACTACAATGGTGAACAACTGGGCAAA
5 gallus_GPAM     92.3%
CTGATGTCCTAATTATACCTGTGGGAATCTCCTACGATCGCATAATTGAAGGTCACTATAACAGTGAACAGCTGGGCAAG
6 alligator_GPAM  83.5%
CTGATGTACTAATTATACCTGTGGGAATTTCGTATGATCGCATCATTGAAGGTCACTATAACAGTGAACAGCTGGGCAAA

                        1201          .         .         .         .         :         .
.        . 1280
1 ruby_GPAM      100.0%
CCTAAGAAGAATGAAAGTCTTTGGAGTGTAGCTAGAGGAGTCTTCAGAATGCTGCGGAAGAATTATGGGTGTGTCAGAGT
2 canna_GPAM      99.6%
CCTAAGAAGAATGAAAGTCTTTGGAGTGTAGCTAGAGGAGTCTTCAGAATGCTGAGGAAGAATTATGGGTGTGTCAGAGT
3 swift_GPAM      94.4%
CCTAAGAAGAATGAAAGTCTTTGGAGTATAGCTAGAGGAGTCTTCAGAATGCTGCGGAAGAATTATGGCTGTGTTAGAGT
4 human_GPAM      76.1%
CCTAAGAAGAATGAGAGCCTGTGGAGTGTAGCAAGAGGTGTTATTAGAATGTTACGAAAAACTATGGTTGTGTCCGAGT
5 gallus_GPAM     92.3%
CCTAAGAAGAATGAAAGTCTTTGGAGTATAGCAAGAGGAGTCTTCAGAATGCTGCGGAAGAATTATGGGTGTGTCAGAGT
6 alligator_GPAM  83.5%
CCTAAGAAGAATGAAAGCCTTTGGAGCATAGCTAGAGGGGTTTTCAGAATGCTGCGGAAGAATTATGGGTGTGTCAGAGT

                        1281          .         3         .         .         .         .
:        . 1360
1 ruby_GPAM      100.0%
AGATTTTGCACAACCATTCTCCTTAAAAGAATATGTGAACAGCCAAAGCCAAAAGCCCATGCCTGCACCTCTTTCTTTGG
2 canna_GPAM      99.6%
AGATTTTGCACAACCATTCTCCTTAAAAGAATATGTGAACAGCCAAAGCCAAAAGCCCATGCCTGCACCTCTTTCTTTGG
3 swift_GPAM      94.4%
AGATTTTGCACAACCATTTTCCTTAAAAGAATATATAAACAGCCAAAGCCAAAAATCTGTGCCTGCTCCTCTTTCATTGG
4 human_GPAM      76.1%
GGATTTTGCACAGCCATTTTCCTTAAAGGAATATTTAGAAAGCCAAAGTCAGAAACCGGTGTCTGCTCTACTTTCCCTGG
5 gallus_GPAM     92.3%
AGATTTTGCACAACCATTTTCCTTAAAAGAATATGTGAACAGCCAAAGTCAAAAAACTGTGCCTGCTCCTCTTTCTCTTG
6 alligator_GPAM  83.5%
AGACTTTGCACAGCCATTTTCTCTAAAAGAATTTGTAGACAGCCAACATCAGAAACCTGCACCTGCTTCACTTTCTTTGG

                        1361          .         .         .         4         .         .
.        . 1440
1 ruby_GPAM      100.0%
AACAAGCTTTGTTACCAGCTATACTTCCATCAAGACCTAATGATACTGTGCATGAAAGTTCAGAGGCTTCACAGCCAAAC
2 canna_GPAM      99.6%
AACAAGCTTTGTTACCAGCTATACTTCCATCAAGACCTAATGATACTGTGCATGAAAGTTCAGAGGCTTCACAGCCAAAC
3 swift_GPAM      94.4%
AACAAGCTTTGTTACCAGCTATACTTCCATCAAGACCTAATGATATGGTGGATGAAGGTGCAGAGGCCTCACTGCCCAAC
4 human_GPAM      76.1%
AGCAAGCGTTGTTACCAGCTATACTTCCTTCAAGACCCAGTGATGCTGCTGATGAAGGTAGAGACACGTCCATTAATGAG
5 gallus_GPAM     92.3%
AACAAGCTTTGTTACCAGCTATACTTCCATCGAGACCTAATGATACTGTGGATGAAGGTACTGAGGCTTCACTGCCTAAC
6 alligator_GPAM  83.5%
AACAAGCTCTATTACCAGCTCTACTTCCATCAAGGCCTAATGATGTTGAGGAAGAAGGTACAGAGGCTGCACTGCCAAAC

                        1441          :         .         .         .         .         5
.        . 1520
1 ruby_GPAM      100.0%
TCCAGGGATATAACCAGTGAACCCTTCAGAAGAGAGCTGATTTCCAACTTAGCTGAGCATGTTTTGTTCACTGCTAACAA
2 canna_GPAM      99.6%
```

```
TCCAGGGATATAACCAGTGAACCTTTCAGAAGAGAGCTGATTTCCAACTTAGCTGAGCATGTTTTGTTCACTGCTAACAA
3 swift_GPAM      94.4%
TCCAGGGATATCACCAGTGAACCCTTCAGAAGAGAGCTGATCTCCAACTTGGCTGAGCATATTTTATTCACTGCGAACAA
4 human_GPAM      76.1%
TCCAGAAATGCAACAGATGAATCCCTACGAAGGAGGTTGATTGCAAATCTGGCTGAGCATATTCTATTCACTGCTAGCAA
5 gallus_GPAM     92.3%
TCCAAGGATATAACCAGTGAACCCTACAGAAGAGAGCTGATAGCCAATTTGGCTGAGCATATTTTATTCACTGCTAACAA
6 alligator_GPAM  83.5%
TCCAGGGACATAAGTAATGAACATTTTAGAAGACAGTTGATAGCCAATTTGGCTGAGCATATATTGTTCACTGCTAACAA

                           1521          .         .         :         .         .         .
.         6 1600
1 ruby_GPAM      100.0%
GTCCTGTGCTGTGATGTCTACCTACATTGTTGCCTGTTTGCTGCTGTACAGACACAGGAAGGGAACTGATCTTTCCAGAT
2 canna_GPAM      99.6%
GTCCTGTGCTGTGATGTCTACCTACATTGTTGCCTGTTTGCTGCTGTACAGACACAGGAAGGGAACTGATCTTTCCAGAT
3 swift_GPAM      94.4%
ATCCTGTGCTGTGATGTCTACCCACATTGTTGCCTGTTTGTTGCTGTACAGACACAGGCAGGGAACAGATCTTTCCAGGT
4 human_GPAM      76.1%
GTCCTGTGCCATTATGTCCACACACATTGTGGCTTGCCTGCTCCTCTACAGACACAGGCAGGGAATTGATCTCTCCACAT
5 gallus_GPAM     92.3%
GTCCTGTGCTGTGATGTCTACCCACATTGTTGCCTGTTTGCTGCTGTACAGACATAGGCAGGGGACTGATCTTTCCAGGT
6 alligator_GPAM  83.5%
GTCATGTGCTGTGATGTCAACGCATATAGTTGCCTGTTTGCTGCTCTACCGACATAGGCAGGGAATTGATCTGTCCAAGT

                           1601          .         .         .         .         :         .
.         . 1680
1 ruby_GPAM      100.0%
TAGTAGAAGATTTCTTTTCCATGAAGGAGGAAGTCTTAGCCCGTGACTATGACTTGGGATTTTCAGGGAACTCAGATGAT
2 canna_GPAM      99.6%
TAGTAGAAGATTTCTTTTCCATGAAGGAGGAAGTCTTAGCCCGTGACTATGACTTGGGATTTTCAGGGAACTCAGATGAT
3 swift_GPAM      94.4%
TGGTAGAAGATTTCTTTTCCATGAAGGAGGAGGTCTTAGCCCGTGACTTTGACTTGGGATTTTCAGGGAACTCGGATGAT
4 human_GPAM      76.1%
TGGTCGAAGACTTCTTTGTGATGAAAGAGGAAGTCCTGGCTCGTGATTTTGACCTGGGGTTCTCAGGAAATTCAGAAGAT
5 gallus_GPAM     92.3%
TGGTGGAAGATTTCTTCTCCATGAAGGAAGAGGTCTTAGCCCGTGACTTCGACTTGGGATTTTCAGGGAACTCAGATGAT
6 alligator_GPAM  83.5%
TGGTGGAAGACTTCTTCTCCATGAAGGAAGAGGTCCTGGCCCGTGACTTTGACTTGGGATTTTCCGGGAATTCTGAGGAT

                           1681          .         7         .         .         .         .
:         . 1760
1 ruby_GPAM      100.0%
GTTGTCATGCATGCCATCCACTTGTTGGGGAACTGTGTAAATATCACAAACACCAGTCGAAACAATGAGTTCTTCATCAC
2 canna_GPAM      99.6%
GTTGTCATGCATGCCATCCACTTGTTGGGGAACTGTGTAAATATCACAAACACCAGTCGAAACAATGAGTTCTTCATCAC
3 swift_GPAM      94.4%
GTTGTCATGCATGCCATCCACTTGTTGGGGAACTGTGTAAATATCACACACGCTAGTCGAAACAACGAGTTCTTCATCAC
4 human_GPAM      76.1%
GTAGTAATGCATGCCATACAGCTGCTGGGAAATTGTGTCACAATCACCCACACTAGCAGGAACGATGAGTTTTTATCAC
5 gallus_GPAM     92.3%
GTTGTCATGCATGCCATCCACTTGTTGGGGAACTGTGTAAATATCACAAACACTAGTCGAAACAACGAGTTCTTCATCAC
6 alligator_GPAM  83.5%
GTTGTCATGCATGCTATCCACCTGCTGGGGAACTGTGTCACTATCACGAATACCAGCCGAAACAATGAGTTCTTCATCAC

                           1761          .         .         .         8         .         .
.         . 1840
1 ruby_GPAM      100.0%
TCCCAGCACAGAGATACCTGCTGTCTTTGAACTCAACTTCTACAGCAATGGAGTACTTCATGTATTCATTAAAGAGGCTG
2 canna_GPAM      99.6%
TCCCAGCACAGAGATACCTGCTGTCTTTGAACTCAACTTCTACAGCAATGGAGTACTTCATGTATTCATTAAAGAGGCTG
3 swift_GPAM      94.4%
TCCCAGCACCAAGATACCTGCTGTCTTTGAACTCAACTTCTACAGCAATGGAGTACTTCATGTATTCATTAAAGAGGCTG
4 human_GPAM      76.1%
CCCCAGCACAACTGTCCCATCAGTCTTCGAACTCAACTTCTACAGCAATGGGGTACTTCATGTCTTTATCATGGAGGCCA
5 gallus_GPAM     92.3%
TCCCAGCACAACGATCCCTGCTGTCTTTGAACTCAATTTCTACAGCAATGGAATTCTTCATGTATTCATTAAAGAGGCAG
6 alligator_GPAM  83.5%
TCCCAGCACAACTATCCCTGCTGTCTTTGAGCTCAACTTCTACAGCAATGGAGTACTCCACGTCTTCATTAATGAAGCCA

                           1841          :         .         .         .         .         9
.         . 1920
1 ruby_GPAM      100.0%
TTATTGCCTGCAGTCTCCATGCGATTCAGAGCAGAAGGTACAGAAATGGTACCAATAGTGCTTCTCCTGGTTTGATTAGT
2 canna_GPAM      99.6%
TTATTGCCTGCAGTCTCCATGCGATTCAGAGCAGAAGGTACAGAAATGGTACCAATAGTGCTTCTCCTGGTTTGATTAGT
3 swift_GPAM      94.4%
TTATAGCCTGCAGTCTTCATGCGATTCAGAGTAGGAGGTGCAGAAATGGTACCAGTGGTGCTTCTTCCAGTTTGATTAGT
4 human_GPAM      76.1%
TCATAGCTTGCAGCCTTTATGCAGTTCTGAACAAGAGGGGACTGGGGGGTCCCACTAGCACCCCACCTAACCTGATCAGC
5 gallus_GPAM     92.3%
```

```
TCATTGCCTGCAGTCTTCATGCAGTTCAGAGTAAGAGGTTCAGAAATGGTACCAATGGTGCTTCTCCCAGCTTGATTAGT
6 alligator_GPAM  83.5%
TTATTGCCTGCAGTCTTCACGCAGTACAAAGTAAAAGGCTTCGAAATGGAATCAATGGTACTTCTCCCAATATGATTAGC

                        1921          .         .         :         .         .         .
.         0 2000
1 ruby_GPAM      100.0%
CAGGAACACCTCGTCCGAAAAGCTGCCAGCTTGTGTTACTTGCTTTCTAATGAATTTACTGTATCTTTGCCTTGCCAGGT
2 canna_GPAM      99.6%
CAAGAACACCTGGTCCGAAAAGCTGCCAGCTTATGTTACTTGCTTTCTAATGAATTTACTGTATCTTTGCCTTGCCAGGT
3 swift_GPAM      94.4%
CAAGAACACTTGGTCCGAAAAGCTGCTAGCTTGTGTTACTTGCTTTCTAATGAATTTACTGTATCTTTGCCTTGCCAGGT
4 human_GPAM      76.1%
CAGGAGCAGCTGGTGCGGAAGGCGGCCAGCCTGTGCTACCTTCTCTCCAATGAAGGCACCATCTCACTGCCTTGCCAGAC
5 gallus_GPAM     92.3%
CAAGAGCACCTTGTCCGCAAAGCTGCCAGCTTGTGCTACTTGCTTTCTAATGAATTTACTGTATCTTTGCCTTGTCAGTT
6 alligator_GPAM  83.5%
CAAGAACAACTGATCCGCAAAGCTGCCAGCTTGTGTTACTTGCTTTCCAATGAAGGCCCTGTCTCCCTGCCCTGCCAGAT

                        2001          .         .         .         .         :         .
.         . 2080
1 ruby_GPAM      100.0%
GATATATCAAGTTTGCCATGAATCTGTGGAAAGGCTGATACAATATGGTATTCTTCTGGTGGCTGAGCAGGATGATCAGG
2 canna_GPAM      99.6%
GATATATCAAGTTTGCCATGAATCTGTGGAAAGGCTGATACAATATGGTATTCTTCTGGTGGCTGAGCAGGATGATCAGG
3 swift_GPAM      94.4%
GATTTATCAAGTTTGCCATGAATCAGTGGAAAGGCTGATACAGTACGGTATTCTTCTGGTGGCCGAGCAGGATGATCAGG
4 human_GPAM      76.1%
ATTTTACCAAGTCTGCCATGAAACAGTAGGAAAGTTTATCCAGTATGGCATTCTTACAGTGGCAGAGCACGATGACCAGG
5 gallus_GPAM     92.3%
GATATATCAAGTTTGCCATGAAGCAGTGGAAAAGCTGATACAATATGGTATTCTTCTGGTGGCTGAGCAGGATGATCAGG
6 alligator_GPAM  83.5%
GTTATATCAAGTTTGCCATGAGGTAGTCGAAAGGTTTATACAGTATGGAATTCTTTTGGTGGCTGAGCAGGATGATCAGG

                        2081          .         1         .         .         .         .
:         . 2160
1 ruby_GPAM      100.0%
AGGATGTTAGCCCCAGTCTTGCAGAGCAGCAGTGGGATAAAAAGCTCCCTGAGCCATTATCTTGGAGAAGTGATGAGGAA
2 canna_GPAM      99.6%
AGGATGTTAGCCCCAGTCTTGCAGAGCAGCAGTGGGATAAAAAGCTCCCTGAGCCATTATCTTGGAGAAGTGATGAGGAA
3 swift_GPAM      94.4%
AGGATGTTAGTCCTAGTCTTACAGAGCAGCAGTGGGATAAAAAGCTCCCTGAGCCATTATCTTGGCGAAGTGATGAGGAA
4 human_GPAM      76.1%
AAGATATCAGTCCTAGTCTTGCTGAGCAGCAGTGGGACAAGAAGCTTCCAGAACCTTTGTCTTGGAGAAGTGATGAAGAA
5 gallus_GPAM     92.3%
AGGATGTTAGTCCCAGTCTTACAGAGCAGCAGTGGAATAAAAAACTCCCTGAACCATTAACTTGGAGAAGTGATGAGGAA
6 alligator_GPAM  83.5%
AGGATCTTAGCCCAAGTCTTACAGAGCAGCAATGGGATAAAAAACTTCCTGAACCTTTGTCTTGGAGGAGTGATGAGGAG

                        2161          .         .         .         2         .         .
.         . 2240
1 ruby_GPAM      100.0%
GATGAAGACAGTGATTTTGGAGAAGAGCAGAGAGATTGCTACCTGAAGGTGAGCCAGTCTAAAGAGCACCAGCAGTACAT
2 canna_GPAM      99.6%
GATGAAGACAGTGATTTTGGAGAAGAGCAGAGAGATTGCTACCTGAAGGTGAGCCAGTCTAAAGAGCACCAGCAGTACAT
3 swift_GPAM      94.4%
GATGAAGACAGTGATTTTGGAGAGGAGCAGAGAGATTGCTACCTGAAGGTGAGCCAGTCTCAAGAGCACCAGCAGTACAT
4 human_GPAM      76.1%
GATGAAGACAGTGACTTTGGGGAGGAACAGCGAGATTGCTACCTGAAGGTGAGCCAATCCAAGGAGCACCAGCAGTTTAT
5 gallus_GPAM     92.3%
GATGAAGATAGTGATTTTGGAGAAGAGCAAAGAGATTGCTACCTGAAGGTCAGTCAGTCTCAAGAGCACCAGCAGTACAT
6 alligator_GPAM  83.5%
GATGAAGATAGTGACTTCGGAGAAGAACAAAGGGACTGTTATCTCAAGGTGAGCCAGTCTCAGGAACACCAGCAGTACAT

                        2241          :         .         .         .         .         3
.         . 2320
1 ruby_GPAM      100.0%
CACTTTCCTGCAGAGGCTGTTGGGACCTTTACTGGAGGCATATAGCTCTGCTGTTATCTTCATCCACAATTTTAGTGGTC
2 canna_GPAM      99.6%
CACTTTCCTGCAGAGGCTGTTGGGACCTTTACTGGAGGCATATAGCTCTGCTGTTATCTTCATCCACAATTTTAGTGGTC
3 swift_GPAM      94.4%
CACTTTCCTGCAGAGGTTGTTGGGACCTTTACTGGAGGCATATAGCTCTGCTGTTATCTTCGTGCACAATTTTAGTGGTC
4 human_GPAM      76.1%
CACCTTCTTACAGAGACTCCTTGGGCCTTTGCTGGAGGCCTACAGCTCTGCTGCCATCTTTGTTCACAACTTCAGTGGTC
5 gallus_GPAM     92.3%
CACTTTCCTGCAGAGGTTACTGGGACCTTTACTGGAGGCATATAGCTCTGCTGTCATCTTTGTACACAATTTTAGTGGTC
6 alligator_GPAM  83.5%
CACCTTCCTGCAAAGGCTGTTGGGGCCTTTATTGGAGGCGTACAGCTCTGCTGCTGCCTTTATCCACAACTTTAGCGGCC

                        2321          .         .         :         .         .         .
.         4 2400
```

```
 1 ruby_GPAM      100.0%
 CTGTTTCAGAGTCTGAATACATTCAAAAGCTGCACAGGCACTTAATAAGCAGGACGGAGAAGAAGGTTGCTGTATATGCT
 2 canna_GPAM      99.6%
 CTGTTTCAGAGTCTGAATACATTCAAAAGCTGCACAGGCACTTAATAAGCAGGACGGAGAAGAAGGTTGCTGTATATGCT
 3 swift_GPAM      94.4%
 CTGTTTCAGAGTCTGAATACCTTCAGAAGTTACACAGGCACTTAATAAGCAGGACGGAGAAGAATGTTGCTGTATATGCT
 4 human_GPAM      76.1%
 CTGTTCCAGAACCTGAGTATCTGCAAAAGTTGCACAAATACCTAATAACCAGAACAGAAAGAAATGTTGCAGTATATGCT
 5 gallus_GPAM     92.3%
 CTGTTTCAGAGTCTGAATACCTTCAAAAGTTACACAGGCATTTAATAAACAGGACAGAGAAGAATGTTGCTGTGTATGCT
 6 alligator_GPAM  83.5%
 CTGTATCAGAAAGGGAGTATCTACAAAAGCTACACAAATACTTAATAAGCAGGACAGAGAAAAATGTTGCGATGTATGCT

                         2401         .         .         .         .         :         .
 .         . 2480
 1 ruby_GPAM      100.0%
 GAGAGTGCTACCTACAGTCATGTGAAAAATGCAGTGAAAGTCTTTAAGGAAATTGGAGTTTTCAATCAGACGAAACAAAA
 2 canna_GPAM      99.6%
 GAGAGTGCTACCTACAGTCATGTGAAAAATGCAGTGAAAGTCTTTAAGGAAATTGGAGTTTTCAATCAGACGAAACAAAA
 3 swift_GPAM      94.4%
 GAGAGTGCTACCTACAGTCATGTGAAAAATGCAGTGAAAGTCTTCAAGGAAATTGGGGTTTTCAATCAGACGAAACAAAG
 4 human_GPAM      76.1%
 GAGAGTGCCACATATTGTCTTGTGAAGAATGCTGTGAAAATGTTTAAGGATATTGGGGTTTTCAAGGAGACCAAACAAAA
 5 gallus_GPAM     92.3%
 GAGAGTGCTACATACAGCCATGTGAAAAATGCAGTGAAAGTCTTTAAGGAAATTGGGGTTTTCATTCAAACAAATCAAAA
 6 alligator_GPAM  83.5%
 GAGAGTGCTACCTATTGCCTTGTGAAAAATGCTGTGAAAGTCTTCAAGGATATGGGGGTTTTCAAGGAGACCAAACA---

                         2481         .         5         .         .         .         .
 :         . 2560
 1 ruby_GPAM      100.0%
 AAGAGACACCATTCTGGAACTAGGTACCACCTTCCTACCTCAGCGCAACAGGCAAAAAACTACTGGAATTCATAATGAGC
 2 canna_GPAM      99.6%
 AAGAGACACCATTCTGGAACTAGGTACCACCTTCCTACCTCAGCGCAACAGGCAAAAA-CTACTGGAATTCATAATGAGC
 3 swift_GPAM      94.4%
 AAGAGACACCATTTTGGAACTAGGTACTACGTTCCTACCTCAGCGCAACAGGCAAAAA-CTACTGGAATTCATCATGAGT
 4 human_GPAM      76.1%
 GAGAGTGTCTGTTTTAGAACTGAGCAGCACTTTTCTACCTCAATGCAACCGACAAAAA-CTTCTAGAATATATTCTGAGT
 5 gallus_GPAM     92.3%
 GAGAGACACTATTTTGGAGCTTAGCACTACGTTCCTACCTCAGCGCAACAGGCAAAAA-CTACTGGAATTCATCATGAGC
 6 alligator_GPAM  83.5%
 GAGGGAGACTATTTTGGAACTGAGCAGTACTTTCCTACCTCAGCACAACCGACAAAAG-CTGCTGGAATTCATTTTGAGC

                         2561         .         .         .         6        ] 2609
 1 ruby_GPAM      100.0%      TTCATGGTGTTATAGATGACACAAAGCACCTTTGCCCAGGACCCTAA--
 2 canna_GPAM      99.6%      TTCATGGTGTTATAG----------------------------------
 3 swift_GPAM      94.4%      TTCATGGTGTTATAG----------------------------------
 4 human_GPAM      76.1%      TTTGTGGTGCTGTAG----------------------------------
 5 gallus_GPAM     92.3%      TTCATGGTATTATAG----------------------------------
 6 alligator_GPAM  83.5%      TTTGTTGTACTGTAA----------------------------------
```

MView 1.60.1, Copyright © 1997-2015 Nigel P. Brown

```
 Reference sequence (1): ruby_LPIN1
 Identities normalised by aligned length.
 Colored by: identity + property

                             1 [         .         .         .         .         :         .
 .         . 80
 1 ruby_LPIN1      100.0%      --------------------------------------------------------------------
 -ACCGCGGCGGTG
 2 gallus_LPIN1     82.2%
 ATGGGGGAAAAGGACCACTTCAAGATGTCGTCTGAGGATGAAGAGCAGCCTGAGAGCCCTCCTGGCTCCCCTTGGTCTTG
 3 canna_LPIN1      96.4%      ------------------------
 ATGTCGTCTGAGGAGGAAGACCAGCCAGAGACCCCAACTGGCTCCCCTTGGTCTTG
 4 swift_LPIN1      87.4%      ------------------------
 ATGTCGTCTGAGGAGGAAGACCAGCCAGAGACCCCAACTGGCTCCCCCTGGTCGTG
 5 human_LPIN1      70.6%      ---------------------------------------------------------------------
 ------------
 6 alligator_LPIN1  79.7%      ---------------------------------------------------------------------
 ------------

                            81         .         1         .         .         .         .
 :         . 160
 1 ruby_LPIN1      100.0%
 TGCCCGCACAAAGCAGCGGCGAGCGCAGTCTCCAGGTGGTGGTGGTGGTCGGC-AGAGGTCCAAACCATGAATTACGTTG
```

```
  2 gallus_LPIN1     82.2%
  GATTCTATTGATGAAAGATCCTGGTTGGATTCGAAATGTCTGGACTCGCAATCTAAACGTCCAAACCATGAACTACGTTG
  3 canna_LPIN1      96.4%
  GATTCCGTTGATGAAAGATCCTGGTTGGATTCGAAATGTCTGGACTCGCAATCTAAATGTCCAAACCATGAATTACGTTG
  4 swift_LPIN1      87.4%
  GATTCCATTGATGAAAGATCCTGGTTGGATTCGAAGCGTCTGGACTCGCAATCTCAATGTGCAAACCATGAATTACGTTG
  5 human_LPIN1      70.6%      ------------------------------------------------------------------
  -ATGAATTACGTGG
  6 alligator_LPIN1  79.7%      ------------------------------------------------------------------
  -ATGAATTACGTCG

                              161          .          .          .          2          .          .
  .          . 240
  1 ruby_LPIN1      100.0%
  GACAGTTAGCAGGGCAAGTATTTGTAACTGTGAAGGAGCTCTATAAGGGACTAAACCCAGCCACATTGTCAGGATGTATA
  2 gallus_LPIN1     82.2%
  GACAGTTAGCAGGGCAAGTGTTTGTAACTGTGAAGGAGCTCTATAAGGGACTAAACCCAGCCACGCTGTCGGGATGTATC
  3 canna_LPIN1      96.4%
  GACAGTTAGCAGGGCAAGTATTTGTAACTGTGAAGGAGCTCTATAAGGGACTAAACCCAGCCACATTGTCAGGATGTATA
  4 swift_LPIN1      87.4%
  GACAGCTAGCAGGGCAGGTGTTTGTGACTGTGAAGGAGCTCTACAAGGGACTAAACCCAGCCACCCTCTCAGGATGCATC
  5 human_LPIN1      70.6%
  GGCAGTTAGCCGGCCAGGTGTTTGTCACCGTGAAGGAGCTCTACAAGGGGCTGAATCCCGCCACACTCTCAGGGTGCATT
  6 alligator_LPIN1  79.7%
  GGCAGTTAGCAGGCCAAGTATTTGTCACTGTGAAGGAGCTCTACAAGGGATTAAATCCTGCTACATTGTCTGGATGCATT

                              241          :          .          .          .          .          3
  .          . 320
  1 ruby_LPIN1      100.0%
  GATATAATTGTTGTACGACAGCCAGATGGAAATCTTCAGTGTTCCCCTTTCCATGTACGCTTTGGGAAAATGGGAGTTCT
  2 gallus_LPIN1     82.2%
  GATATAATTGTTGTACGTCAGCCAGATGGGAATCTTCAGTGTTCTCCATTCCATGTACGCTTTGGGAAAATGGGAGTTCT
  3 canna_LPIN1      96.4%
  GATATAATTGTTGTACGACAGCCAGATGGAAATCTTCAGTGTTCCCCTTTCCATGTACGCTTTGGGAAAATGGGAGTTCT
  4 swift_LPIN1      87.4%
  GATATCATCGTGGTGCGGCAGCCGGATGGGAACCTGCAGTGCTCCCCCTTCCACGTGCGCTTTGGGAAGATGGGGGTTCT
  5 human_LPIN1      70.6%
  GACATCATTGTCATCCGCCAGCCCAATGGAAACCTCCAATGCTCCCCTTTCCACGTCCGCTTTGGGAAGATGGGGGTCCT
  6 alligator_LPIN1  79.7%
  GATATAATTGTTGTACGCCAGCCAGATGGAAACCTCCAGTGTTCCCCTTTTCATGTGCGCTTCGGGAAAATGGGGGTTCT

                              321          .          .          :          .          .          .
  .          4 400
  1 ruby_LPIN1      100.0%
  ACGTTCTAGGGAGAAAGTGGTTGACATAGAAATTAACGGAGAGGCTGTAGACTTGCACATGAAACTAGGAGACAATGGAG
  2 gallus_LPIN1     82.2%
  GCGTTCCAGGGAGAAAGTGGTTGACATAGAAATTAATGGAGAGGCTGTAGATTTGCACATGAAGCTAGGAGACAATGGAG
  3 canna_LPIN1      96.4%
  ACGTTCTAGGGAGAAAGTGGTTGACATAGAAATTAACGGAGAGGCTGTAGACTTGCACATGAAACTAGGAGACAATGGAG
  4 swift_LPIN1      87.4%
  GCGCTCCAGGGAGAAAGTGGTTGACATAGAAATTAATGGAGAGGCTGTAGACTTGCACATGAAACTGGGAGACAATGGAG
  5 human_LPIN1      70.6%
  GCGCTCCCGAGAGAAAGTGGTTGACATAGAAATCAATGGGGAATCTGTGGATTTGCATATGAAATTGGGAGATAATGGAG
  6 alligator_LPIN1  79.7%
  GCGTTCTAGGGAGAAAGTGGTTGACATAGAAATTAATGGAGAATCTGTAGATTTGCACATGAAACTAGGGGACAATGGAG

                              401          .          .          .          .          :          .
  .          . 480
  1 ruby_LPIN1      100.0%
  AGGCTTTCTTTGTGCAGGAGATGGATAATGATCAGGAGGTAATTCCTTTTCATCTTGCCACATCTCCCATTCTGTCTGAG
  2 gallus_LPIN1     82.2%
  AAGCCTTTTTCGTCCAGGAGATGGATAACAATCAGGAGGTAATTCCTTATCATCTGTCTACGTCCCCCATCCTGTCTGAG
  3 canna_LPIN1      96.4%
  AGGCTTTCTTTGTGCAGGAGATGGATAATGATCAGGAGGTAATTCCTTTTCATCTTGCCACATCTCCCATTCTGTCTGAG
  4 swift_LPIN1      87.4%
  AGGCCTTTTTTGTGCAGGAGATGGATAACGATCAGGAGGTAATTCCATATCATCTTGCCACATCTCCCATTCGGTCTGAG
  5 human_LPIN1      70.6%
  AAGCATTTTTTGTTCAAGAAACAGATAATGATCAGGAAGTTATCCCTATGCACCTGGCCACCTCCCCCATCCTGTCAGAA
  6 alligator_LPIN1  79.7%
  AAGCATTTTTCGTACAGGAAACAGATAATGATCAGGAGGTAATTCCTCACCACCTTTCCACATCCCCCATTCTATCAGAG

                              481          .          5          .          .          .          .
  :          . 560
  1 ruby_LPIN1      100.0%
  GGAAGTGCTTTAATGGAAGTTCAGCTGAAGAGGAACTCCATAGACAGGATAAGAAACCTGGACACCAGTGCATCCTCACA
  2 gallus_LPIN1     82.2%
  GGAACTGCGTTAATGGAAGCTCAGCTGAAGAGAAACTCAATTGACAGGATAAGAAACCTGGACAGCAGTGTATCTTCACA
  3 canna_LPIN1      96.4%
  GGAAGTGCTTTAATGGAAGTTCAGCTGAAGAGGAACTCCATAGACAGGATAAGAAACCTGGACACCAGTGCATCCTCACA
  4 swift_LPIN1      87.4%
  GGAACGGCTTTGATGGAAGCTCAGCTGAAGAGGAACTCTATAGACAGGATAAGAAGTCTGGACACCAGTGCATCCTCACA
```

```
5 human_LPIN1      70.6%
GGAGCTTCGAGAATGGAATGCCAGCTGAAAAGGGGCTCTGTGGACAGGATGAGAGGCCTGGACCCCAGCA----------
6 alligator_LPIN1  79.7%
GGAGTTGCTCTAATGGAATCACAGCGGAAGAGGAACTCAATAGACTGGGTCAGGAGCTTGGACAGTAGTGGATCCTCACA

                          561         .         .         .         6         .         .
.         . 640
1 ruby_LPIN1      100.0%
GCTATCACCCCAAGCCTATGGATCCCAGCCTTTGACTGAAACATCTCCATCCTGTAGCTCTGTGAAAAAAAGGAGGAAAA
2 gallus_LPIN1     82.2%
AGTACCACCCCAAGCTCATGGATCCCAGCCTGGTACTGAAACATCTCCAGCCTGTAGCTCTGTGAAAAAGAGGAGGAAAA
3 canna_LPIN1      96.4%
GCTATCACCCCAAGCCTATGGATCCCAGCCTTTGACTGAAACATCTCCATCCTGTAGCTCTGTGAAAAAAAGGAGGAAAA
4 swift_LPIN1      87.4%
ACTGTCACCCCAAGGCCATGGATCCCAGCCTTCTGCTGAGACATCTGCAGCCTGTAGCTCTGTGAAAAAAAGGAGGAAGA
5 human_LPIN1      70.6%      --CGCCAGCCCAAGTG-ATCGCTCCCAGC-------
-GAGACGCCGTCAAGCAGCTCTGTAGTAAAGAAGAGAAGAAAAA
6 alligator_LPIN1  79.7%
AGCACAAACTCAAGTACATGTTTCTCAGCCTGCTATAGAAACCTCTTCAGTTTGTGGCTCCGTGAAGAAAAGAAGAAAAA

                          641         :         .         .         .         .         7
.         . 720
1 ruby_LPIN1      100.0%
AGAGGAGGAAGTCTACCCATAAAATAGACAGTTTAAAACGAGAAGGCAATGGAGACACCTCAGAAGATGAAGATATGTTT
2 gallus_LPIN1     82.2%
AGAGGAGGAAGTCTACCCACAAAATAGACAGCTTAAAAAGAGAAGACATTGGAGATACATCAGAAGATGAAGACATGTTT
3 canna_LPIN1      96.4%
AGAGGAGGAAGTCTACCCATAAAATAGATAGCTTAAAACGAGAAGGCAATGGAGACACCTCAGAAGATGAAGATATGTTT
4 swift_LPIN1      87.4%
AGAGGAGGAAGTCTACCCATAAAATAGACAGCTTAAAAAGAGAAGAGAATGGAGACACATCAGAAGATGAAGACATGTTT
5 human_LPIN1      70.6%      GGAGGAGAAAGTC-AC----
-AGCTGGACAGCCTGAAGAGAGATGACAACATGAACACATCTGAGGATGAGGACATGTTC
6 alligator_LPIN1  79.7%
AGAGGAGAAAATCTACCCATAAAATAGACAGCTTGAAAAGAGAAGACAATGGAGACACTTCAGAAGATGAAGACATGTTT

                          721         .         .         :         .         .         .
.         8 800
1 ruby_LPIN1      100.0%
CCTATAGAGATTAGCTCAGAGGAAGAAAAAGAACCATTGGATGATTCAAGAATCCCTGTTCCAGATGCGTTT--CTT---
2 gallus_LPIN1     82.2%
CCTATAGAGATTAGCTCAGAGGAAGAAAAGGAACAATTGGACAATTCAAGGATTCTTGTTCCAGATGTGTTT--GTT---
3 canna_LPIN1      96.4%
CCTATAGAGATTAGCTCAGAGGAAGAAAAAGAACCATTGGATGATTCAAGAATCCCTGTTCCAGATGCGTTT--CTT---
4 swift_LPIN1      87.4%
CCTATAGAGATAAGCTCAGATGAAGAAAAAGAACCATTGGACAGTTCCAGGGTCCCTGTTCCAGATGTGCTC--GTT---
5 human_LPIN1      70.6%
CCCATCGAGATGAGCTCGGATGAGGCCATGGAGCTGCTGGAGAGCAGCAGAACTCTTCCTAATGATATACCTCCATTCCA
6 alligator_LPIN1  79.7%
CCTATAGAGATTAGTTCAGATGAAGAGAATGACCTATTGGACAACAGCAGGAAGTCAGTTCCTGATGTGTGT--GTT---

                          801         .         .         .         .         :         .
.         . 880
1 ruby_LPIN1      100.0%
-GATGATGCATCTGTCATGAAGGCTCCAGCAGTTACTACATTTTCTCAGTCTGCATCTTACCCTAATTCAGATGGAGAAT
2 gallus_LPIN1     82.2%
-GATGAAGTATCTGATATAAAGGCTCCTGCTGTTTCTGCTTATTCTCAGTCTTCATCTTACCCTCGTTCGGATGGAGAAT
3 canna_LPIN1      96.4%
-GATGATGCATCTGTCATGAAGGCTCCAGCAGTTACTACATTTTCTCAGTCTGCATCTTACCCTAATTCAGATGGAGAAT
4 swift_LPIN1      87.4%
-GATGATGTATCTGACAGAAAGGCTCCAGCAGTTTCTACATTTTCTCAGTCTGCATCTTACCCTAATTCAGATGGAGAAT
5 human_LPIN1      70.6%
AGATGATATTCCTGAGGAAAACCTCTCCCTGGCTGTGATTTACCCTCAGTCAGCCTCATACCCTAATTCGGATAGAGAGT
6 alligator_LPIN1  79.7%
-GACGAAATATCAGAGAAGAATACTTCTGCGCTCAATACCCTTTCTCAGTCTGCACCATATCCCCATTCAGATGGGGAAT

                          881         .         9         .         .         .         .
:         . 960
1 ruby_LPIN1      100.0%      GGTCGTCTCTTCAAAG----------------------------------------------------
------------
2 gallus_LPIN1     82.2%
GGTCACCCATTCAAAGTAAGCCCATAGATTACACAGGGCAATCCTCTCTTCTCACTGTTCCAGCAGATGGAGGCCTATCT
3 canna_LPIN1      96.4%      GGTCGTCTCTTCAAAG----------------------------------------------------
------------
4 swift_LPIN1      87.4%      GGTCACCTCTTCAAAG----------------------------------------------------
------------
5 human_LPIN1      70.6%      GGTCACCCACTCCCAG----------------------------------------------------
------------
6 alligator_LPIN1  79.7%
GGTCACCCCTTCAAAGTCAGCCTATAGAATGCACAGGGCCATCCTCTCATCTCACAGTTCCAGCAGATGGAGGCCTATCT

                          961         .         .         .         0         .         .
```

```
.           . 1040
1 ruby_LPIN1      100.0%      ------------------------------------------
-CCTATCAGGTTCACGCCCTCCTACTCCTCAAAGTGA
2 gallus_LPIN1     82.2%
AACTCTTGTCCTCATCAGTCTTCTCACTTTTCTCCTCCAGACAGCCTGTCAGGCTCACGTCCTCCTACTCCTCAAAGTGA
3 canna_LPIN1      96.4%      ------------------------------------------
-CCTATCAGGTTCACGCCCTCCTACTCCTCAAAGTGA
4 swift_LPIN1      87.4%      ------------------------------------------
-CCTCTCAGGTTCCCGCCCTCCTACTCCTCAGAGTGA
5 human_LPIN1      70.6%      -------------------------------------------
TCCTTCCGGTTCCCGACCTTCAACACCTAAAAGTGA
6 alligator_LPIN1  79.7%
AGTTCTTGTCCTCATCAATCTTCTCGCTTTCCTGCGACAGACAGCCCCTCAGGTTCACGGCCTCCCACTCCTAAAAGTGA

                         1041         :         .         .         .         .         1
.           . 1120
1 ruby_LPIN1      100.0%
TTCAGAATTAGTCAGTAAACCTACAGACAGAAGTGGATCGAAGAATAATCCCCACATGCACTGGGCATGGGGAGAGCTAC
2 gallus_LPIN1     82.2%      TTCAGAATTAGTCAGTAAACCTACAGACAGGAGTGGACTAAAGAAT--
-CCACACATGCACTGGGCATGGGGAGAGCTAC
3 canna_LPIN1      96.4%
TTCAGAATTAGTCAGTAAACCTACAGACAGAAGTGGATCGAAGAATAATCCCCACATGCACTGGGCATGGGGAGAGCTAC
4 swift_LPIN1      87.4%
CTCAGAATTAGTCAGTAAACCTGTGGACAGGAGTGGACTGAAGAATAATCCTCACATGCACTGGGCATGGGGAGAGCTAC
5 human_LPIN1      70.6%      TTCAGAATTGGTCAGCAAGTCCACGGAAAGGACAGGGCAGAAGAAC--
-CCAGAAATGCTTTGGCTGTGGGGAGAGCTGC
6 alligator_LPIN1  79.7%
TTCAGAATTAATCAGTAAAACTTTGGACCGGGGAGCACAGAAGAATAACCCACAAATGCATTGGGCCTGGGGAGAACTAC

                         1121         .         .         :         .         .         .
.           2 1200
1 ruby_LPIN1      100.0%      CCCAGGCTGCAAAGGCCAGCTTTCTGCTCAAAGCAAAGGAACCCAGCATAGTGGA--
-TGTAAATCCTTCTGAAAGCACT
2 gallus_LPIN1     82.2%      CACAGGCTACAAAGGCAAGTTCCTTGATCAAAGCTAAGGAACCCAACACAGTAGA--
-TGTAAATCCTTCAGAAAGCACT
3 canna_LPIN1      96.4%      CCCAGGCTGCAAAGGCCAGCTTTCTGCTCAAAGCAAAGGAACCCAGCATAGTGGA--
-TGTAAATCCTTCTGAAAGCACT
4 swift_LPIN1      87.4%      CCCAGGCTGCAAAGGCCACTTCTCTGCTCAAAGCAAAGGAACCCAGCCTGGTGGA--
-TGTAAATCCCTCTGAAAGCACT
5 human_LPIN1      70.6%
CGCAGGCTGCTAAGTCTTCTTCTCCACACAAGATGAAAGAGTCCAGCCCATTGAGCAGTAGAAAAATTTGTGATAAAAGT
6 alligator_LPIN1  79.7%      CTCAAGCCGCAAAGTCCACTTC---ACTCAAGGCTAAAGAAGCTGGTGTGATGAA--
-TGTAAATCCTTCTGAAAGCACT

                         1201         .         .         .         .         :         .
.           . 1280
1 ruby_LPIN1      100.0%
CACTTTCGGGTCATCCAGAGTTCTCCTGTAGAGGAGTTTGGGACTGTAGCTCCTCTACCTGACCTTGGACAAGCAGGTGC
2 gallus_LPIN1     82.2%
CACTTTCGGGTCATCCAGAGTGCTCCTATAGAGGAGTTTAATGGCGTGTCTCCTCTACCTGCCCTTGGACAGACAGATGC
3 canna_LPIN1      96.4%
CACTTTCGGGTCATCCAGAGTTCTCCTGTAGAGGAGTTTGAGACTGTAGCTCCTCTACCTGACCTTGGACAAGCAGGTGC
4 swift_LPIN1      87.4%
CACTTTCGGGTCATCCAGAGTTCTCCTAGTGAGGAGTTTAACACTGTGTCCCCTCTACCTGCCCTTGAACAAGCAGGTGC
5 human_LPIN1      70.6%      CACTTTCAGGCCATTCACAGCGAATCTTCAGACACTTTTAGTGACCAATC------GCCAACTCT---
---GGTCGGTGG
6 alligator_LPIN1  79.7%
CACTTTCGGGTCATACAAAGTGCTCCTGCAGAAGAGTTTGGCAGTGTATCTCTTTCACCTGCTCTTGGACAGGCAGATAC

                         1281         .         3         .         .         .         .
:           . 1360
1 ruby_LPIN1      100.0%      AGCAACTGCTGATGAAAGGGAGCCCTT-----
-ACCTGTTGATGCAAATAAGCCAGAGTTAGAGTCTCCAGGAGCAGCTG
2 gallus_LPIN1     82.2%      AGCAACTGCTGATGAAACTGAGCCCTT------
GCCAGCTGAGACAAATAAGCCAGAGACAGAATCTGCAGGAGCAGCTG
3 canna_LPIN1      96.4%      AGCAACTGCTGATGAAAGGGAGCCCTC-----
-ACCTGCTGATGCAAATAAGCCAGAGTTAGAGTCTCCAGGAGCAGCTG
4 swift_LPIN1      87.4%      AACAACTGCTGAGGAAAGTGAACCCTC-----
-ACCAGCTGAGACAAATAAGCCAGAGACTGAGTCTCCAGGAGCAGCTG
5 human_LPIN1      70.6%
GGCACTTTTGGACCAGAACAAGCCTCAGACAGAAATGCAGTTTGTGAATGAAGAAGACCTGGAGACCTTAGGAGCAGCAG
6 alligator_LPIN1  79.7%
TGCAGCTCCTGATGAAACCGAGCCCCAGCCAGAACCAGCTGAGATAAATCAACCAGAGCCTGAGTCTGCAGGAGCAGTTG

                         1361         .         .         .         4         .         .
.           . 1440
1 ruby_LPIN1      100.0%
TGGCACCATTGTCTGCCAATGAAGAATTAAAACAATCTACAGCCTGCTCATCCCAACCAGCTGGCAAGACAGATTCCCCT
2 gallus_LPIN1     82.2%
TACCATCATTGCCTGCCAATGAGGAAATAAAACAAGCTGCTGCTTGCTCAGCCCAGGCAGTTGGCAAGACAGATTCCCCT
3 canna_LPIN1      96.4%
```

```
TGGCACCATTGTCTGCCAATGAAGAATTAAAACAATCTACAGCCTGCTCATCCCAACCAGCTGGCAAGACAGATTCCCCT
4 swift_LPIN1       87.4%
TACCACCCTTGTCTGCCAATGAAGAACTAAAACAAGCTGCAGCTTGTTCAGCCCAACCAGCTGGCAAGACAGATTCCCCT
5 human_LPIN1       70.6%
CGCCACTCTTGCCCATGATCGAGGAGCTCAAACCCCCCTCTGCCAGTGTAGTCCAGACAGCAAACAAGACGGATTCTCCT
6 alligator_LPIN1   79.7%
CAGCACCTCTGCCTGCGAATGAAGACATAAAACAAGCTGCATCTTGCTTAGCTCAGCCAGTTAGCAAAACAGATTCACCC

                         1441          :         .         .         .         .         5
.         . 1520
1 ruby_LPIN1       100.0%
TCCAGAAAAAAAGACAAACGAAGCCGGCATCTTGGTGCTGATGGTGTCTATTTAGATGACCTCACTGACATGGATCCAGA
2 gallus_LPIN1      82.2%
TCCAGAAAGAAAGACAAACGAAGCCGGCATCTTGGTGCTGATGGCGTCTATTTAGATGACCTTACTGACATGGATCCAGA
3 canna_LPIN1       96.4%
TCCAGAAAAAAAGACAAACGAAGCCGGCATCTTGGTGCTGATGGTGTCTATTTAGATGACCTCACTGACATGGATCCAGA
4 swift_LPIN1       87.4%
TCCAGAAAGAAAGACAAACGAAGCCGGCATCTTGGTGCTGATGGTGTCTATTTAGATGACCTCACTGACATGGATCCAGA
5 human_LPIN1       70.6%
TCCAGGAAAAGAGATAAACGAAGCCGACATCTTGGTGCTGACGGCGTCTACTTGGATGACCTCACAGACATGGATCCTGA
6 alligator_LPIN1   79.7%
TTCAAAAAGAAAGATAAACGAAGTCGGCATCTTGGTGCCGATGGTGTCTATTTAGATGACCTCACCGACATGGATCCAGA

                         1521          .         .         :         .         .         .
.         6 1600
1 ruby_LPIN1       100.0%      AGTTGCTGCACTTTATTTCCCCAAAAATGGGGATAATG--
-TCCAAAGCAAGAACACAAACGATGCGGGGCCTCGGTCTG
2 gallus_LPIN1      82.2%      AGTTGCTGCACTTTATTTCCCCAAAAATGGGGATAATG--
-TACAAAACAGAAACACAAATGACACAGGGCCACGGTCTG
3 canna_LPIN1       96.4%      AGTTGCTGCACTTTATTTCCCCAAAAATGGGGATAATG--
-TCCAAAGCAAGAACACGAACGATGCGGGGCCTCGGTCTG
4 swift_LPIN1       87.4%      AGTTGCTGCACTTTATTTCCCCAAGAACGGGGAGAGTG--
-TCCAGAGCAAGCACCCTCCTGAGGCAGGGCCCTGGTCTG
5 human_LPIN1       70.6%
AGTGGCGGCCCTGTATTTTCCCAAAAACGGAGATCCTTCCGGACTCGCAAAACATGCAAGCGACAACGGAGCCCGGTCAG
6 alligator_LPIN1   79.7%
AGTTGCTGCACTTTATTTCCCCCAAAATGGTGATAATACTTTACATAATAAGAATGGAAATGAAAATGGACCATGGTCCG

                         1601          .         .         .         .         :         .
.         . 1680
1 ruby_LPIN1       100.0%
CCAGCAACTCCCCACAGTCTGTTGGGAGCTCAGGTGTTGACAGTGGAGCTGAAAGCACCTCGGATGGAATTCGAGATTTG
2 gallus_LPIN1      82.2%
CCACTCATTCTCCACAGTCTTTTGGGAGCTCAGGCGCTGACAGTGGTGTTGAAAGCACCTCAGATGGAACCAGAGATTTG
3 canna_LPIN1       96.4%
CCAGCAACTCCCCACAGTCTGTTGGGAGCTCAGGTGTTGAAAGTGGAGCTGAAAGCACCTCGGATGGAATTCGAGATTTG
4 swift_LPIN1       87.4%
CCCACCCGTCCCCGCAGTCCGTGGGGAGCTCAGGTGTGGAAAGTGGAGCTGAGAGCACCTCAGATGGAGCACGGGACTTG
5 human_LPIN1       70.6%
CCAACCAGTCCCCGCAGTCGGTGGGCAGCTCGGGCGTGGACAGTGGCGTGGAGAGCACCTCGGACGGGCTGAGGGACCTC
6 alligator_LPIN1   79.7%
CCAATCAGTCTCCTCAGTCTGTTGGAAGTTCAGGTGTTGACAGTGGTGTTGAAAGTGTCTCTGATGGAATAAGAGATTTG

                         1681          .         7         .         .         .         .
:         . 1760
1 ruby_LPIN1       100.0%
CCTTCCATTGCCATTTCTCTCTGCGGAGGCCTTATTGACAACAAAGAAATAACCAAAGAAGAATTTTTAGAACATGCAGT
2 gallus_LPIN1      82.2%
CCTTCGATTGCCATCTCTCTCTGTGGAGGCCTCACGGACAACAAAGAGATAACCAAAGAAGAATTCTTAGAACATGCAGT
3 canna_LPIN1       96.4%
CCTTCCATTGCCATTTCTCTCTGCGGAGGCCTTATTGACAACAAAGAAATAACCAAAGAAGAATTTTTAGAACATGCAGT
4 swift_LPIN1       87.4%
CCCTCCATTGCCATCTCTCTCTGTGGAGGCCTCACAGACAGCAAGGAGATCACCCAAGAAGAATTCTTGGAACATGCAGT
5 human_LPIN1       70.6%
CCTTCCATCGCCATCTCCCTCTGCGGGGGCCTCAGCGACCACCGGGAGATCACGAAAGATGCATTCCTGGAGCAAGCTGT
6 alligator_LPIN1   79.7%
CCCTCCATTGCCATTTCACTCTGTGGTGGTCTTAGTGACAACAAAGAAATAACAAAAGATCAGTTCTTAGAGCATGCAGT

                         1761          .         .         .         8         .         .
.         . 1840
1 ruby_LPIN1       100.0%
AACATATCAGCAGTTTGTGGACAATCCTGCTATAATTGATGACCCTAACCTTGTGGTTAAGATTGGAAATAAGTACTACA
2 gallus_LPIN1      82.2%
AACGTATCAACAGTTTGTGGACAATCCTGCTATCATTGATGACCCTAACCTTGTGGTTAAGATTGGAAATAAGTACTACA
3 canna_LPIN1       96.4%
AACATATCAGCAGTTTGTGGACAATCCTGCTATAATTGATGACCCTAACCTTGTGGTTAAGATTGGAAATAAATACTACA
4 swift_LPIN1       87.4%
AACGTATCAGCAATTTGTGGACAATCCTGCTATCATTGATGACCCTAACCTCGTGGTTAAGATTGGAAATAAGTACTACA
5 human_LPIN1       70.6%
GTCATATCAACAGTTTGTGGACAACCCCGCTATTATCGATGACCCCAATCTCGTGGTAAAGATTGGGAGTAAATATTATA
6 alligator_LPIN1   79.7%
```

```
  AACATATCAGCAGTTTGTGGACAATCCTGCCATCATTGATGACCCCAATCTTGTGGTTAAGATTGGAAACAAATACTACA

                            1841          :         .         .         .         .         9
  .         . 1920
  1 ruby_LPIN1      100.0%
  ACTGGACAACAGCTGGTCCCCTTCTGCTGGCAATGCAGGCATTCCAGAGACCTTTGCCAAAGGCTACTGTGGAATCTATA
  2 gallus_LPIN1     82.2%
  ACTGGACAACAGCTGGTCCCCTTCTGCTGGCAATGCAGGCATTCCAGAAACCTTTGCCAAAGGCCACTGTGGAATCTATA
  3 canna_LPIN1      96.4%
  ACTGGACAACAGCTGGTCCCCTTCTGCTGGCAATGCAGGCATTCCAGAGACCTTTGCCAAAGGCTACTGTGGAATCTATA
  4 swift_LPIN1      87.4%
  ACTGGACAACAGCTGGTCCCCTTCTGCTGGCAATGCAGGCATTCCAGAGACCTTTGCCAAAGGCTACTGTGGAATCTATC
  5 human_LPIN1      70.6%
  ACTGGACAACAGCAGCACCCCTCCTCCTGGCAATGCAGGCCTTCCAGAAACCTTTGCCAAAGGCCACTGTGGAATCTATC
  6 alligator_LPIN1  79.7%
  ACTGGACAACAGCTGCTCCACTCCTCCTGGCAATGCAAGCATTTCAGAAACCTTTGCCAAAGGCCACTGTGGAATCTATA

                            1921          .         .         :         .         .         .
  .         0 2000
  1 ruby_LPIN1      100.0%
  ATGAGGGACAAGATGCCCAAAAAGGGTGGAAGGTGGTGGTTTTCATGGCGAGGGAGGAACAGCACTATTAAAGAGGAAAC
  2 gallus_LPIN1     82.2%
  ATGAGAGACAAGATGCCCAAAAAAGGTGGAAGATGGTGGTTCTCTTGGAGAGGGAGAAACAGCACTATTAAAGAGGAAAC
  3 canna_LPIN1      96.4%
  ATGAGGGACAAGATGCCCAAAAAGGGTGGAAGGTGGTGGTTTTCATGGCGAGGGAGGAACAGCACTATTAAAGAGGAAAC
  4 swift_LPIN1      87.4%
  ATGAGGGACAAGATGCCCAAAAAAGGTGGAAGGTGGTGGTTTTCCTGGCGAGGGAGGAACAGCACTATCAAAGAGGAAGC
  5 human_LPIN1      70.6%
  ATGAGGGATAAAATGCCCAAAAAGGGAGGAAGATGGTGGTTTTCATGGAGGGGAAGAAACACCACAATCAAGGAGGAAAG
  6 alligator_LPIN1  79.7%
  ATGAGGGATAAGATGCCCAAGAAAGGTGGAAGATGGTGGTTTTCATGGCGAGGGAGGAATAGCACTATAAAAGAGGAAAC

                            2001          .         .         .         .         :         .
  .         . 2080
  1 ruby_LPIN1      100.0%      AAAGCCAGACCAAGGTATGAGTGG---GAGTGGACTCACAGGAGG--
  -CTCTTCACAGATGAGCATGGCAAACAG-----
  2 gallus_LPIN1     82.2%      AAAGGCAGAACAAGGTATGAGTGG--
  -GAGTAGACTTAAAGGAGAAGACTCTTCACAGATGACCATGGCAAACAGGAAGT
  3 canna_LPIN1      96.4%      AAAGCCAGACCAAGGTATGAGTGG---GAGTGGACTCACAGGAGG--
  -CTCTTCACAGATGAGCATGGCAAACAG-----
  4 swift_LPIN1      87.4%      AAAGGCAGAGCAAGGAATGAGTGG---GACTGGACTGACAGGAGA---
  GTCTTCACAGCTGGGCATGGCAAACAG-----
  5 human_LPIN1      70.6%
  TAAGCCAGAGCAGTGCTTGGCTGGCAAGGCCCATAGCACCGGAGAGCAACCGCCGCAGCTCAGCTTGGCCACCAG-----
  6 alligator_LPIN1  79.7%      CAAGCCAGAACTCGG----AAACG---GACTTAAT-
  -ACAGGAGAGGAGTCATTACAGCTGACCATTACAAAGAG-----

                            2081          .         1         .         .         .         .
  :         . 2160
  1 ruby_LPIN1      100.0%      ------------------------------------------
  -AATAAAAGATGAATCTTCTTCAAGTGACGAAGACCCC
  2 gallus_LPIN1     82.2%
  TCAGGAGCCTATCACTTCCCAGGCTTATTTTCACACGCAATAGAATAAAAGATGAATCTTCTTCGAGCGATGAAGACCCT
  3 canna_LPIN1      96.4%      -------------------------------------------
  GATAAAAGATGAATCTTCTTCAAGTGACGAAGACCCC
  4 swift_LPIN1      87.4%      ------------------------------------------
  -AATAAAAGATGAATCTTCTTCAAGTGATGAAGACCCT
  5 human_LPIN1      70.6%      -------------------------------------------
  GGTAAAGCATGAATCATCCTCCAGTGATGAGGAGCGC
  6 alligator_LPIN1  79.7%      ------------------------------------------
  -AATAAAGGATGAATCTTCTTCAAGTGATGAAGACCCT

                            2161          .         .         .         2         .         .
  .         . 2240
  1 ruby_LPIN1      100.0%
  AGAGCTGCTAAACAAAACCTTGGGTCATTACAAAGCAACTCAAGTCATCTCTCATTATTGTCTGGAATTGGTTACAAAAA
  2 gallus_LPIN1     82.2%
  AGAGCTGCCAAACAAAACCTTGGGTCATTACAAGCCAACTCGAGTCATCTCTCATTATTGTCTGGAGTCAGTTACAAAAA
  3 canna_LPIN1      96.4%
  AGAGCTGCTAAACAAAACCTTGGGTCATTACAAAGCAACTCAAGTCATCTCTCATTATTGTCTGGAATTGGTTACAAAAA
  4 swift_LPIN1      87.4%
  AGAGCTGCCAAACAGAACCTGGGGTCATTACAAACCAACTCAAGTCACCTTTCATTACTGCCTGGAATCAGCTACAAAAA
  5 human_LPIN1      70.6%      GCAGCTGCCAAGC------------CATCA-----
  -AACGCAGGCCACCTCCCTCTTCTGCCTAATGTCAGCTACAAGAA
  6 alligator_LPIN1  79.7%
  AGAGCTGCCAAACAAAATATCGGGTCATTACAAACAAACTCAAGTCACCTCTCATTATTGTCTGGAATCAGTTACAAGAA

                            2241          :         .         .         .         .         3
  .         . 2320
  1 ruby_LPIN1      100.0%
  AACACTTCGACTCACTTCTGACCAGCTTAAAAGCTTGAAACTCAAGAATGGCCCCAATGATGTCACCTTTAGTGTTACAA
```

```
  2 gallus_LPIN1     82.2%
  AACACTTCGACTAACTTCTGACCAGCTTAAAAGCTTAAAGCTGAAGAATGGTCCCAATGATGTGACTTTCAGTGTTACAA
  3 canna_LPIN1      96.4%
  AACACTTCGACTCACTTCTGACCAGCTTAAAAGCTTGAAACTCAAGAATGGCCCCAATGATGTCACCTTTAGTGTTACAA
  4 swift_LPIN1      87.4%
  GACACTGCGACTCACTTCTGACCAGCTCAAAAGCTTAAAGCTGAAGAATGGCCCCAATGATGTGACCTTCAGTGTCACCA
  5 human_LPIN1      70.6%
  GACTCTCCGGCTGACTTCCGAGCAGCTTAAAAGCTTGAAGTTGAAGAATGGCCCCAACGACGTGGTTTTCAGTGTCACCA
  6 alligator_LPIN1  79.7%
  GACCCTTCGACTCACTTCTGACCAGCTTAAAAGCCTGAAGCTGAAGAATGGCCCCAATGATGTGACCTTCAGTGTTACAA

                            2321         .         .         :         .         .         .
  .         4 2400
  1 ruby_LPIN1      100.0%
  CACAGTATCAAGGCACTTGCCGCTGTGAAGGAACTATTTACCTGTGGAATTGGGATGAGAAAGTTATTATTTCTGACATC
  2 gallus_LPIN1     82.2%
  CGCAATATCAAGGTACTTGCCGCTGTGAAGGCACCATTTACCTATGGAATTGGGATGATAAGGTTATTATTTCTGATATC
  3 canna_LPIN1      96.4%
  CACAGTATCAAGGCACTTGCCGCTGTGAAGGAACTATTTACCTGTGGAATTGGGATGAGAAAGTTATTATTTCTGACATC
  4 swift_LPIN1      87.4%
  CACAGTACCAAGGCACCTGCCGCTGTGAAGGCACCATCTACCTGTGGAACTGGGATGAGAAAGTGATTATTTCTGACATT
  5 human_LPIN1      70.6%
  CGCAGTACCAAGGCACGTGCCGCTGTGAGGGCACCATCTATCTGTGGAACTGGGATGATAAAGTCATCATTTCTGATATT
  6 alligator_LPIN1  79.7%
  CACAATATCAAGGCACTTGCCGTTGTGAAGGCACAATCTACTTCTGGAACTGGGATGAAAAGGTCATTATTTCTGACATT

                            2401         .         .         .         .         :         .
  .         . 2480
  1 ruby_LPIN1      100.0%
  GATGGAACAATCACACGGTCGGATACTTTAGGTCATATTTTGCCTACTCTTGGAAAAGACTGGACACACCAAGGGATTGC
  2 gallus_LPIN1     82.2%
  GATGGAACAATTACACGGTCAGATACTTTAGGTCATATTTTGCCTACTCTCGGCAAAGACTGGACACACCAAGGGATTGC
  3 canna_LPIN1      96.4%
  GATGGAACAATCACACGGTCGGATACTTTAGGTCATATTTTGCCTACTCTTGGAAAAGACTGGACACACCAAGGAATTGC
  4 swift_LPIN1      87.4%
  GATGGAACAATCACACGGTCAGATACTTTAGGTCATATTTTGCCAACTCTTGGCAAAGACTGGACACACCAAGGGATTGC
  5 human_LPIN1      70.6%
  GATGGGACAATTACCAGATCAGATACTCTTGGCCACATTTTGCCCACCCTTGGGAAGGATTGGACCCATCAGGGCATCGC
  6 alligator_LPIN1  79.7%
  GATGGAACTATCACAAGGTCTGATACATTAGGCCATATCTTGCCCACTCTTGGCAAGGACTGGACGCACCAAGGGATTGC

                            2481         .         5         .         .         .         .
  :         . 2560
  1 ruby_LPIN1      100.0%
  AAAGTTGTACCATAAAGTGAGCCAAAATGGGTATAAATTTTTGTATTGCTCAGCACGTGCTATTGGCATGGCAGACATGA
  2 gallus_LPIN1     82.2%
  AAAGTTGTACCATAAAGTGAGCCAAAATGGATATAAGTTTTTGTATTGCTCAGCACGTGCTATTGGAATGGCAGACATGA
  3 canna_LPIN1      96.4%
  AAAGTTGTACCATAAAGTGAGCCAAAATGGGTATAAATTTTTGTATTGCTCAGCACGTGCTATTGGCATGGCAGACATGA
  4 swift_LPIN1      87.4%
  AAAGTTGTACCATAAAGTGAGCCAAAATGGCTACAAGTTTTTGTACTGCTCAGCACGAGCCATTGGGATGGCAGACATGA
  5 human_LPIN1      70.6%
  TAAGCTGTACCATAAAGTGAGCCAGAATGGATATAAATTTCTCTACTGTTCTGCCCGTGCCATCGGGATGGCGGACATGA
  6 alligator_LPIN1  79.7%
  AAAATTGTACCATAAAGTGAGCCAAAATGGGTATAAATTTCTGTACTGCTCAGCACGTGCTATAGGAATGGCAGACATGA

                            2561         .         .         .         6         .         .
  .         . 2640
  1 ruby_LPIN1      100.0%
  CCAGAGGATACTTGCACTGGGTCAATGAGCGAGGAACTGTGCTGCCTCAGGGGCCAGTGTTGCTGAGTCCAAGCAGCTTA
  2 gallus_LPIN1     82.2%
  CCAGAGGATACTTGCACTGGGTCAATGAACGTGGAACTGTGCTGCCACAGGGGCCAGTGTTGCTGAGTCCAAGCAGCTTA
  3 canna_LPIN1      96.4%
  CCAGAGGATACTTGCACTGGGTCAATGAGCGAGGAACTGTGCTGCCTCAGGGGCCAGTGTTGCTGAGTCCAAGCAGCTTA
  4 swift_LPIN1      87.4%
  CCAGGGGCTACTTGCACTGGGTGAACGAGCGGGGCACGGTGCTGCCGCAGGGGCCGGTGCTGCTGAGCCCCAGCAGCTTG
  5 human_LPIN1      70.6%
  CGCGGGGCTACCTGCACTGGGTCAACGAGAGGGGCACGGTGCTGCCCCAGGGGCCCCTGCTGCTGAGTCCCAGCAGCCTC
  6 alligator_LPIN1  79.7%
  CCAGAGGATATTTACATTGGGTCAACGAACGAGGGACAGTACTACCTCAGGGACCTGTGCTATTAAGTCCAAGCAGCTTG

                            2641         :         .         .         .         .         7
  .         . 2720
  1 ruby_LPIN1      100.0%
  TTCTCAGCTTTACACAGGGAAGTGATAGAAAAGAAGCCAGAAAAATTTAAAGTCCAGTGTTTGACAGACATAAAAAATTT
  2 gallus_LPIN1     82.2%
  TTTTCAGCTTTGCACAGGGAAGTGATAGAGAAAAAGCCAGAAAAATTCAAAGTTCAGTGTTTGACAGACATAAAAAATTT
  3 canna_LPIN1      96.4%
  TTCTCAGCTTTACACAGGGAGGTGATAGAAAAGAAGCCAGAAAAATTTAAAGTCCAGTGTTTGACAGACATCAAAAATTT
  4 swift_LPIN1      87.4%
  TTCTCAGCTTTCCACAGGGAGGTGATAGAAAAGAAACCAGAAAAATTTAAAGTTCAGTGTTTGACAGACATCAAAAATTT
```

```
5 human_LPIN1      70.6%
TTCTCTGCCCTGCACAGAGAAGTGATTGAAAAGAAGCCAGAAAAGTTTAAAGTCCAGTGTTTGACAGACATCAAAAACCT
6 alligator_LPIN1  79.7%
TTCTCAGCCTTTCACAGGGAAGTGATAGAGAAGAAGCCAGAAAAATTTAAAGTTCAGTGTCTGACAGACATAAAAAATTT

                         2721          .         .         :         .         .         .
.         8 2800
1 ruby_LPIN1      100.0%
GTTTTATCCTAACACAGAACCCTTTTATGCTGCTTTTGGAAACAGACCTGCTGATGTTTATTCATACAAACAGGTGGGAG
2 gallus_LPIN1     82.2%
GTTTTATCCTAACACAGAACCCTTTTATGCTGCTTTTGGAAACAGACCTGCTGATGTTTATTCATACAAACAAGTGGGTG
3 canna_LPIN1      96.4%
GTTTTATCCTAACACAGAACCCTTTTATGCTGCTTTTGGAAACAGACCTGCTGATGTTTATTCATACAAACAGGTGGGAG
4 swift_LPIN1      87.4%
GTTTTATCCTAACACAGAACCCTTTTATGCTGCTTTTGGAAACAGACCTGCTGATGTTTATTCATACAAACAGGTGGGAG
5 human_LPIN1      70.6%
GTTTTTCCCCAACACAGAACCTTTTATGCTGCTTTTGGAAACCGACCAGCTGATGTGTATTCATACAAGCAAGTAGGAG
6 alligator_LPIN1  79.7%
GTTTTATCCTAACATAGAGCCCTTCTATGCTGCCTTTGGAAACAGACCTGCCGATGTTTACTCATACAAGCAAGTAGGGG

                         2801          .         .         .         .         :         .
.         . 2880
1 ruby_LPIN1      100.0%
TTTCTTTAAACAGGATATTTACAGTTAACCCCAAAGGAGAACTTATACAAGAGCATGCAAAGACAAACATCTCATCTTAC
2 gallus_LPIN1     82.2%
TTTCTTTAAACCGAATATTTACCGTCAACCCCAAAGGAGAACTTATACAAGAACATGCAAAGACAAATATCTCATCCTAT
3 canna_LPIN1      96.4%
TTTCTTTAAACAGGATATTTACAGTTAACCCCAAAGGAGAGCTTATACAAGAGCATGCAAAGACAAACATCTCATCTTAC
4 swift_LPIN1      87.4%
TATCTTTAAACAGGATATTTACAGTTAACCCCAAAGGAGAACTTATCCAAGAACATGCAAAGACAAACATCTCATCCTAT
5 human_LPIN1      70.6%
TGTCTTTGAATAGAATATTTACCGTCAACCCTAAAGGAGAGCTGGTACAGGAACATGCAAAGACCAACATCTCTTCGTAT
6 alligator_LPIN1  79.7%
TCTCTTTAAATAGAATATTTACAGTTAATCCCAAAGGAGAACTTGTACAAGAACATGCAAAGACCAACATCTCTTCCTAC

                         2881          .         9         .         .         .         .
:         . 2960
1 ruby_LPIN1      100.0%
GTCAGACTGTGTGAAGTGGTAGATCACATTTTCCCGTTGCTGAAAAGAAGCCATTCTTCAGACTTCCCTTGTTCTGATAC
2 gallus_LPIN1     82.2%
GTCAGACTGTGTGAAGTGGTAGACCATATTTTCCCTTTGCTGAAAAGAAGCCATTCTTCAGATTTCCCTTGTTCTGATAC
3 canna_LPIN1      96.4%
GTCAGACTGTGTGAAGTGGTAGATCACATTTTCCCGTTGCTGAAAAGAAGCCATTCTTCAGACTTCCCTTGTTCTGATAC
4 swift_LPIN1      87.4%
GTGAGACTGTGTGAAGTAGTAGATCACATTTTTCCTTTGCTGAAAAGAAGCCATTCTTCAGACTTCCCTTGTTCTGACAC
5 human_LPIN1      70.6%
GTGAGACTCTGTGAAGTAGTCGACCACGTTTTCCCGTTGCTGAAAAGAAGCCATTCTTCAGACTTTCCCTGTTCGGATAC
6 alligator_LPIN1  79.7%
GTCAGACTATGTGAGGTGGTTGATCATGTGTTTCCTTTGCTGAAAAGAAGCCATTCTTCAGATTTTCCTTGTTCTGACAC

                         2961          .         .         .         0         .         .
.       ] 3038
1 ruby_LPIN1      100.0%
CTACAGTCAGTTCACATACTGGAGAGAACCTCTGCCACCTTTTGAAACTCAGGATGAAACTCCAGCCTCATCTTAA--
2 gallus_LPIN1     82.2%
CTACAGTCAGTTTACCTACTGGAGAGAACCTCTGCCACCTTTTGAAACTCAGGATGTACATCCAGATTCATCTTAA--
3 canna_LPIN1      96.4%
CTACAGTCAGTTCACATACTGGAGAGAACCTCTGCCACCTTTTGAAACTCAGGATGAAACTCCAGCCTCATCTTAA--
4 swift_LPIN1      87.4%
CTACAGTCAGTTCACCTACTGGAGAGAACCTCTACCACCTTTTGAAACTCAGGATGAAAATCCACCCTCATCTTAG--
5 human_LPIN1      70.6%
CTTCAGTAACTTCACCTTTTGGAGAGAGCCACTGCCACCTTTTGAAAACCAGGACATTCATTCTGCCTCAGCGTAA--
6 alligator_LPIN1  79.7%
ATATAGTCAGTTCACATACTGGAGAGAACCATTACCACCTTTTGAAAACCAGGATATAAATCGATCTTTATCTTAA--
```

MView 1.60.1, Copyright © 1997-2015 Nigel P. Brown

```
Reference sequence (1): ruby_MCAT
Identities normalised by aligned length.
Colored by: identity + property


                           1 [         .         .         .         .         :         .
.         . 80
1 ruby_MCAT      100.0%      ---------------------------------------------------------------------
---------AC
2 canna MCAT      85.4%      ---------------------------------------------------------------------
```

----
3 swift_MCAT   78.6%   ---
----
4 human_MCAT   58.9%   ATGAGCGTCCGGGTCGCACGGGTAGCGTGGGTCAGGGGCTTGGGCGC---
CAGCTACCGCCGCGGCGCCTCG-AGCTTCC
5 gallus_MCAT   73.5%
ATGGGCAGCTGGGCCGCGGCAACATGGCGGCTCGCTGGCTGCAGCGGGCGCGGCGTCCTGCGCGGCGCAGCGCGGCGGCG
6 alligator_MCAT 67.1%   ATGTGCAGCTGGGTGGCGGCGGCGGGATGG-CGACGGGCT--AGCTG---CAGCG--
CGCTGCGGTCCGGCG-GGGGGCG

81 . 1 . . . .
: . 160
1 ruby_MCAT 100.0%
GCGGCGGCGGGGCAGCTCCCGCCCCGGCGATGGGGACCGGGCGGCGATCCTGAGCGACCTGCTGCAGAGCTCGGTGGAGG
2 canna_MCAT   85.4%   ---
----
3 swift_MCAT   78.6%   ---
----
4 human_MCAT   58.9%   CGGTGCCTC---------CGCCGGGCGCCCAG-----
GGTGTAGCGGAGCTGCTGCGAGATGCGACCGGGGCGGAGGAGG
5 gallus_MCAT   73.5%   GGGCAGCTCGCTCCGAGGCGGTGGGGAACGGGCGGCGAGCCTGAGCGAGCTGCTGCAGAGCTCGGT---
GGGGGACGAGG
6 alligator_MCAT 67.1%   ----GCCGC---------CGCCGGGCAGCCTG-----AGCG------ACTTGCTGCAGAGCTCGGT---
GACGGGCGAAG

161 . . . 2 . .
. . 240
1 ruby_MCAT 100.0%
CCGAGGAGCCGGGCGCGGCAGCGGCGGCGAGGCGGGCGCGGCCGTCCCCCCAGGAGGGCACGGTGCTGCTCTTTCCCGGG
2 canna_MCAT   85.4%   ---
----
3 swift_MCAT   78.6%   ---
----
4 human_MCAT   58.9%   AGGCGCCCTGGGCGGCGACGGAGCGGCGAATGCCGGGCCAG------T--------
-GCTCCGTGCTGCTCTTCCCGGGC
5 gallus_MCAT   73.5%
AGCCGGGCGAGGGGGAGGCGGAGGGGGAGAGGCGGGAGCGGCGGTCTCCGCGGGAGGGCGCGGTGCTGCTGTTCCCCGGG
6 alligator_MCAT 67.1%   ACACGGGCACGGAGGCGGCGGCGCGGCGGGGGCGGCGCCTGGACGCCT---------
CCTCGGTGCTGCTGTTCCCGGGC

241 : 3
. . 320
1 ruby_MCAT 100.0%
CAGGGCAGCCAGTTCGTGGGGATGGGCCGCGGGCTGCTGGGGTACCCCGGCGTGCGGGACATGTACCGCCTGGCCGAGAA
2 canna_MCAT   85.4%   -----------
ATGGGTGCCGATGCCCCCCGGGC-GGTGCCTCCACCCCGCAGCCCGTCCCTGTCCCCGTTCCCCCGAAA
3 swift_MCAT   78.6%   ATGGGGAAAGAGCAGCGGTGGAAGCCTCAGGAGA-GAAAAGGTTTTAGAGCAACCGGTATTT-
CAGAGGCTTGTCAAAGA
4 human_MCAT   58.9%
CAGGGCAGCCAGGTGGTGGGCATGGGCCGCGGTCTGCTCAACTACCCGCGCGTCCGCGAACTCTACGCCGCCGCCCGCCG
5 gallus_MCAT   73.5%
CAGGGCAGCCAGTTCGTGGGGATGGGCCGCGGGCTGCTGCGCTACCCCGGCGTGCGGGACATGTACCGCCTGGCCGAGAA
6 alligator_MCAT 67.1%
CAGGGCAGCCAGCGGGCGGGGATGGGCCGGGGCCTGCTGAGCTTCCCCGGTGCGAGGGACCTGTTCGTCGCGGCCCGCGA

321 . . : . . .
. 4 400
1 ruby_MCAT 100.0%
GGTGCTGGGCTACGACCTGCTCTCCCTCTGCCTGGAGGGGCCGCGGGAAGAGCTGGACCGCACCCAGCACTGCCAGCCCG
2 canna_MCAT   85.4%   GGGACC---CGGAGGACAGGGCGCGCAGGGCCGCCACACGAT----GCCTTCCCCAGGCTCCTCCAT-
-CTGCCCGC---
3 swift_MCAT   78.6%   AGTGCC---TTATTGACTTGGC-
TGTTGTCTTGGCATACGTTTAAAAGCTTTTATTGCCATAGTTGTGGCTGCTGGAATG
4 human_MCAT   58.9%
CGTGCTGGGCTACGACCTGCTGGAACTGAGCCTGCACGGGCCGCAGGAGACCCTGGACCGCACCGTGCACTGTCAGCCCG
5 gallus_MCAT   73.5%
GGTGCTGGGCTACGACCTGCTTTCGCTCTGCCTGGAGGGGCCGCGGGCCGAGCTGGACCGCACCCGGCACTGCCAGCCCG
6 alligator_MCAT 67.1%
CGTGTTAGGCTACGACCTGCTATCGCTCTGCCTGCACGGGCCGCAGACGGAGCTGGATCGCACAGTGCACTGCCAGCCCG

401 . . . . : .
. . 480
1 ruby_MCAT 100.0%   CCGTGTTCGTCGCCTCCCTGGCCGCCGTGGAGAAGCTCAACCACCAGCAGCCTAAAGTGGT---
-GGAGAGCTGCGTGGC
2 canna_MCAT   85.4%   ----GGCCCGCCCTGCCCGGGCTGGGGAAG---------GCGATGGCGGTCCCCACGCAGG--------
GGCTGCCCGG-
3 swift_MCAT   78.6%   AGGAGGCAGGGAGTAGTAGGAAAGTGGAAATTCCACTT-
GTAACAGATGTGCTTAAACGGGTGGTGCGGAGCTGCGTGGC
4 human_MCAT   58.9%   CGATCTTCGTGGCATCGCTGGCCGCTGTCGAGAAACTACATCACCTGCAGCCCTCGGTGATTG---
-AGAACTGTGTTGC
5 gallus_MCAT   73.5%   CCGTGTTCGTCGCCTCCCTGGCCGCCGTAGAGAAGCTCAACCACCTGCAGCCTAAGGTGGTGG---

```
-AGAGCTGCGTGGC
6 alligator_MCAT  67.1%      CTGTCTTCGTGTCCTCCCTGGCCGCCGTGGAGAAGCTCAGCCACCAGCGGCCTGATGTGATAG---
-AGAACTGTGTTGC

                             481          .         5         .         .         .         .
:         . 560
1 ruby_MCAT      100.0%
GGCGGCGGGGTACAGCGTGGGGGAGTTCGCGGCGCTGGTCTTCGCTGGAGCCCTGGGCTTTGCGGAAGCGCTGTACGCGG
2 canna_MCAT      85.4%
GGCGGCGGGGTACAGCGTGGGGGAGTTCGCGGCGCTGGTCTTCGCTGGAGCCCTGGGCTTTGCGGAAGCGCTGTACGCGG
3 swift_MCAT      78.6%
GGCCGCGGGGTACAGCGTGGGGGAGTTTGCGGCGCTGGTCTTCGCTGGAGCCCTCAGCTTTGCCGAAGCGCTGTACGCGG
4 human_MCAT      58.9%
TGCTGCTGGATTCAGTGTGGGAGAGTTTGCAGCCCTAGTGTTTGCCGGAGCCATGGAATTTGCTGAAGGTTTGTATGCAG
5 gallus_MCAT     73.5%
CGCCGCCGGCTACAGCGTGGGCGAGTTCGCGGCGCTGGTGTTCGCCGGAGCCATGGACTTCGCGGAAGCACTCTATGCTG
6 alligator_MCAT  67.1%
AGCCGCCGGGTTCAGCGTGGGCGAGTTTGCTGCCCTGGTGTTTGCTGGAGCCTTAGACTTTGAAGCAGCCTTGTATGCAG

                             561          .         .         .         6         .         .
.         . 640
1 ruby_MCAT      100.0%
TGAAAGTGCGCGCCGAAGCCATGCAAAAGGCGTCGGAAGCTGCCCCCAGTGGAATGCTATCGGTTATCGGTCGGCGAGAG
2 canna_MCAT      85.4%
TGAAAGTGCGCGCCGAAGCCATGCAAAAGGCGTCGGAAGCTGCCCCCAGTGGAATGCTATCGGTTATCGGTCGGCGAGAG
3 swift_MCAT      78.6%
TGAAAGTCCGCGCCGAAGCCATGCAAAAGGCGTCGGAAGCCGTCCCCAGCGGAATGCTGTCGGTTGTCGGTCGGCGAGAG
4 human_MCAT      58.9%
TGAAAATCCGAGCTGAGGCCATGCAGGAAGCTTCAGAAGCTGTCCCCAGTGGGATGCTGTCTGTCCTCGGCCAGCCTCAG
5 gallus_MCAT     73.5%
TGAAGGTGCGTGCTGAGGCCATGCAGGCGGCGGCGGAGGCCGTCCCCAGTGGGATGCTGTCGGTCATCGGCGGACGAGAG
6 alligator_MCAT  67.1%
TGAAAGTGCGTGCTGAAGCAATGCAACAGGCATCGGAAGCAGTCCCAAGTGGGATGTTATCAATTATTGGGCAACCCAAG

                             641          :         .         .         .         .         7
.         . 720
1 ruby_MCAT      100.0%
GCAAATTACAAATTTGCCTGCTTGGAAGCCCGTAAACACTGTGAATCGCTGGGTATAGAAAACCCTGTCTGTGAAATTTC
2 canna_MCAT      85.4%
GCAAATTACAAATTTGCCTGCTTGGAAGCTCGTAAACACTGTGAATCGCTGGGTATAGAAAACCCTGTCTGTGAAATTTC
3 swift_MCAT      78.6%
GCAAATTACAAATTTGCCTGCTTGGAAGCCCGTCAGCACTGTGAATCGCTGGGCGTAGCAAACCCGGTGTGTGCTGTTTC
4 human_MCAT      58.9%
TCCAAGTTCAACTTCGCCTGTTTGGAAGCCCGGGAACACTGCAAGTCTTTAGGCATAGAGAACCCCGTATGTGAAGTGTC
5 gallus_MCAT     73.5%
GCCAACTACAAATACGCCTGCCTGGAAGCCCGTAGGCACTGTGAGTCGCTGGGCATTGACAACCCCGTGTGCGAGATCTC
6 alligator_MCAT  67.1%
TCAGATTTCAAGGTTGCCTGCATAGAAGCTCGTGAACACTGTAGATCATTGGGTGTAGAAGATCCTGTTTGTGAAGTTGC

                             721          .         .         :         .         .         .
.         8 800
1 ruby_MCAT      100.0%
AAACTATTTGTTTCCAGACAGCAGAGTCATTGCAGGACACATACAGGCTTTGGAGTTTTTGCAGGAGAATGCCCGAAAAT
2 canna_MCAT      85.4%
AAACTATTTGTTTCCAGACAGCAGAGTCATTGCAGGACACATACAGGCTTTGGAGTTTTTGCAGGAGAATGCCCGAAAAT
3 swift_MCAT      78.6%
AAACTACTTGTTTCCAGACTGCAGAGTCATTGCAGGACACTTACAGGCTTTGGAGTTTTTGCAGGAGAATGCCCGAAAAT
4 human_MCAT      58.9%
CAACTACCTCTTTCCAGATTGCAGGGTGATTTCAGGACACCAAGAGGCTCTACGGTTTCTCCAGAAGAATTCCTCTAAGT
5 gallus_MCAT     73.5%
CAACTACCTGTTCCCGGATAGCAGAGTCATCGCAGGGCACATACAGGCTTTGGAGTTTTTGCAGAAGAACGCCCCAAAAT
6 alligator_MCAT  67.1%
AAATTACCTGTTTCCAGATAGCAGAGTTGTTGCAGGTCATTTACAGGCCTTGAAGTTTTTAGAGGAGAACTCCCGAAAAT

                             801          .         .         .         .         :         .
.         . 880
1 ruby_MCAT      100.0%
ATTATTTTACACGTGCAAAATTGCTTCCGGTCAGTGGGGCTTTTCATACCAGACTTATGGAACCAGCAGTAGAACCACTG
2 canna_MCAT      85.4%
ATTATTTTACACGTGCAAAATTGCTTCCGGTCAGTGGGGCGTTTCATACCAGACTTATGGAACCAGCAGTAGAACCACTG
3 swift_MCAT      78.6%
ACTATTTTACACGTACCAAAATGCTTCCGGTCAGTGGGGCTTTTCATACCAGACTTATGGAACCAGCAGTAGAGCCTTTG
4 human_MCAT      58.9%
TTCATTTCAGACGCACCAGGATGTTGCCGGTTAGTGGCGCATTCCACACCCGCCTCATGGAGCCAGCCGTGGAGCCCCTG
5 gallus_MCAT     73.5%
TCAGCTTCACACGTACCAAAATGCTTCCGGTCAGTGGTGCCTTCCACACACGACTCATGGAACCAGCAGTAGAGCCGCTG
6 alligator_MCAT  67.1%
ATTATTTTGCACGTACAAAAATGCTGCCAGTCAGTGGAGCTTTTCACACAAGACTTATGGAATCAGCCATAGAGCCCTTG

                             881          .         9         .         .         .         .
:         . 960
```

```
1 ruby_MCAT      100.0%
GCTGAAGCCCTAAAATCGATTGAGATTCAGAAACCACTGCTCTGTGTCTATTCCAATGTTGATGGCAAAAAGTACATGCA
2 canna_MCAT      85.4%
GCTGAAGCCCTAAAATCGATTGAGATTCAGAAACCACTGCTCTGTGTCTATTCCAATGTTGATGGCAAAAAGTACATGCA
3 swift_MCAT      78.6%
GCTGAAGTCCTAAAATCGATTGAGATTCAGAAACCTCTGGTCTGTGTCTATTCCAATGTTGATGGCAAAAAGTACATGCA
4 human_MCAT      58.9%
ACGCAAGCTTTAAAGGCAGTCGACATTAAGAAGCCTCTGGTTTCTGTCTACTCCAACGTCCACGGGCATAGATACAGGCA
5 gallus_MCAT     73.5%
GCTGAAGTCCTGAAGTCCATCGAAATTCAGAAACCACTGATTTGTGTCTATTCCAACGTCGACAGCAAAAAGTACATGCA
6 alligator_MCAT  67.1%
GCAAAAGTACTCAAATCAGTTGAGATTCGGAAACCACTCATAAATGTCTATTCAAACGTCGATGGCAAAAAATACATGCA

                         961         .         .         .         0         .         .
.         . 1040
1 ruby_MCAT      100.0%
CTCAAAGCACATTCAGAAGCTGTTAGTGAAGCAAGTGGTTTCTCCTGTTATGTGGGAACAGACCATGCATTCTGTTTATG
2 canna_MCAT      85.4%
CTCAAAGCACATTCAGAAGCTGTTAGTGAAGCAAGTGGTTTCTCCTGTTATGTGGGAACAGACCATGCATTCTGTTTATG
3 swift_MCAT      78.6%
TTCAAAGCACATTCAGAAGCTGTTAGTGAAGCAGGTGGTTTCACCTGTTATGTGGGAACAGACCATGCATTCTGTATATG
4 human_MCAT      58.9%
TCCCGGGCACATCCACAAGCTGCTGGCCCAGCAGCTGGTCTCCCCAGTGAAGTGGGAGCAGACGATGCATGCCATATACG
5 gallus_MCAT     73.5%
CTCAAAGCACATCCAGAAACTGCTGGTGAAGCAGCTCGTCTCACCCGTGCTGTGGGAGCAGACTATGCATTCCATGTACC
6 alligator_MCAT  67.1%
TTCAAAACATATTCAACATTTGTTAGTAAAGCAGCTTGTTTCACCTGTAAAATGGGAACAGACTATGCATGAAGTATATG

                        1041         :         .         .         .         .         1
.         . 1120
1 ruby_MCAT      100.0%
AAAGAAAGCAAGGAACAGAATTTCCTTACACATATGAAGTGGGGCCTGGGAAGCAACTAGGAGCCATTCTCAAAAAATGT
2 canna_MCAT      85.4%
AAAGAAAGCAAGGAACAGAATTTCCTTACACATATGAAGTGGGGCCTGGGAAGCAACTAGGAGCCATTCTCAAAAAATGT
3 swift_MCAT      78.6%
AAAGAAAGCAAGGAACAGAATTTCCTTACACGTATGAAGTGGGACCCGGGAAGCAACTGGGAGCCATTCTCAAAAAATGT
4 human_MCAT      58.9%
AAAGGAAAAAGGGCAGGGGGTTCCCCCAAACTTTCGAAGTAGGCCCTGGCAGGCAGCTGGGAGCCATCCTGAAGAGCTGT
5 gallus_MCAT     73.5%
AGAGGAAGCAGGGAATGGAATTTCCATACACGTATGAAGTGGGTCCTGGGAAACAGCTGGGAGCGGTTCTCCGAAAGTGC
6 alligator_MCAT  67.1%
AAAGAACAAAGAGAACGAAGTTTCCTTTTACATATGAAGTGGGACCTGGGAAACAACTAGGAGCAATGCTCAGAAACTGC

                        1121         .         .         :         .         .         .
.         2 1200
1 ruby_MCAT      100.0%      AATTTAAAGGCCTGGAAACAATATAACCATGTAGATGCTTTGGAAGATGAGGAAGCAGCAGAAACCTA-
-----------
2 canna_MCAT      85.4%
AATTTAAAGGCCTGGAAACAATATAACCATGTAGATGCTTTGGAAGATGAGGAAGCAGCAGAAACCTAA-----------
3 swift_MCAT      78.6%
AATTTAAAGGCCTGGAAGCAGTATAAACATGTAGATGCTCTGGAAGATGAGGAAGCAGCAGAGACCTAA-----------
4 human_MCAT      58.9%      AACATGCAGGCCTGGAAGTCCTACAGCGCCGTGGATGTG-
CTGCAGACCCTCGAACATGTGGACCTGGACCCTCAGGAGC
5 gallus_MCAT     73.5%
AATCTGAAGGCCTGGAGGCAGTACAGCCACGTGGATGTCACAGAGGAGGAGGAAGCGGCAGAGACATAA-----------
6 alligator_MCAT  67.1%
AATTTAAAGGCTTGGAGAGTCTATAAACATATTGAAGTATCAGAAGATGAGGAAGTAGAGGAGGCATGA-----------

                        1201         .  ] 1213
1 ruby_MCAT      100.0%      -------------
2 canna_MCAT      85.4%      -------------
3 swift_MCAT      78.6%      -------------
4 human_MCAT      58.9%      CCCCGAGATGA--
5 gallus_MCAT     73.5%      -------------
6 alligator_MCAT  67.1%      -------------
```

MView 1.60.1, Copyright © 1997-2015 Nigel P. Brown
