# Supplementary material for: Single-molecule, full-length transcript sequencing provides insight into the extreme metabolism of the ruby-throated hummingbird Archilochus colubris
Source: Gigascience. 2018 Feb 15;7(3):giy009. doi: 10.1093/gigascience/giy009 (PMC5869288; doi:10.1093/gigascience/giy009)
Supplement: Supplemental material [file giy009_supp.zip › Supplemental_methods_workman_180102.pdf]

Data processing, isoform clustering and sorting using the DNANexus Pipeline

The SMRTanalysis 3.1 software (https://github.com/PacificBiosciences/SMRT-Analysis) and IsoSeq pipeline were employed using a DNANexus interface. Raw sequence files produced from the Pacbio RSII (bax.h5, bas) were converted into BAM files using bax2bam, zmws_per_split 3. Circular consensus sequence (CCS) was generated from subread BAM files, parameters: min_length 300, max_drop_fraction 0.8, no_polish TRUE, min_zscore -9999, min_passes 1, min_predicted_accuracy 0.8, max_length 15000. CCS.BAM files were output, which were then classified into full length and non-full length reads using pbclassify.py, ignorepolyA false, minSeqLength 300. Non-full length and full-length fasta files produced were then fed into the cluster step, which does isoform-level clustering (ICE), followed by final Arrow polishing, hq_quiver_min_accuracy 0.99, bin_by_primer false, bin_size_kb 1, qv_trim_5p 100, qv_trim_3p 30. Four size fractions (1-2kb, 2-3kb, 3-6kb, 5-10kb) of male liver mRNA sample were sequenced on 40 SMRT Cells, producing 440.75 Gb of raw data, 3.4Gb of full-length non-chimeric reads after circular consensus sequence (CCS) generation and filtering for full-length read classification. Of the four size-selected bins, our average CCS length was 1533, 2464, 3650, and 5444 bp, respectively (Figure 1B).

Preparation of Illumina libraries for polishing

2ug total liver RNA (260/280=2.0, 260/230=1.16) in nuclease free water was input into Lexogen mRNA sense v2 Illumina library preparation kit. 100bp insert molecule size was targeted using 14ul PB and 2ul PS (Lexogen kit), and 12 cycles PCR were performed. The library was sequenced on a single rapid-run flowcell of a Hiseq 4000 using 2x100bp paired end reads, for a total yield of 153M reads.

Filtering for contamination using blast

BLASTn was performed using an NCBI AMI on AWS, and was run with the following settings: evalue = 1e-10, max_target_seqs 1, default scoring parameters.

Aligning to reference using GMAP

We aligned with GMAP (Wu and Watanabe 2005) version 2016-09-23 with parameters -f samse -n 0 -z senseforce against Calypte anna genome (Zhang et al. 2014a).

Assessing transcriptome completion using BUSCO

To determine completeness of our transcriptome assembly relative to established transcriptomes, as well as relative retention of transcripts between multiple data processing steps, we used benchmarking software BUSCO (Benchmarking Universal Single Copy Orthologs). BUSCO searches for a list of conserved orthologous genes assumed to be present in all completed transcriptome assemblies for members of the given clade. We utilized the Metazoan and Aves lineage datasets (Simão et al. 2015) to determine assembly completeness for not only our ASD (all-sequence data) dataset, but also the HQD (High quality dataset) and COGENT-collapsed dataset (CCD, described below) in order to ensure that sequence diversity

was not lost when filtering. We then compared this result with the chicken transcriptome (*Gallus gallus)* (ftp://ftp.ncbi.nih.gov/genomes/Gallus_gallus), the predicted transcriptome from the recently published Anna's hummingbird (*Calypte anna*) genome (http://gigadb.org/dataset/101004), and *Gallus gallus* from a single-tissue Pacbio Iso-Seq dataset (http://journals.plos.org/plosone/article?id=10.1371/journal.pone.0094650). Busco v2.0 BETA was used (downloaded 9/1/2016, https://gitlab.com/ezlab/busco), and results are summarized in Figure 2C and Supplemental Table 1. Notably, the NCBI *Gallus gallus* transcriptome is nearly complete for both Aves and Metazoan sets, with the predicted transcriptome from *Calypte anna* not far behind. In contrast, our transcriptome contains only about half of the Aves set and slightly more of the metazoan set; many genes are not substantially expressed in a single tissue. Notably our ortholog detection is dramatically improved over the other single-tissue Pacbio data available, (embryonic heart tissue, Thomas et al. 2014), which only captured 6% of the Aves set.

ORF prediction and protein translation using ANGEL

The ANGEL pipeline (https://github.com/PacificBiosciences/ANGEL), a long read implementation of ANGLE (Shimizu et al. 2006) was used to determine protein coding sequences from cDNAs. We performed this analysis on both our HQ polished isoform (HQD) and all sequences datasets (ASD). This resulted in 119,292 HQD and 1,061,147 ASD peptide sequences, with a size distribution comparable to human, chicken, swift and alligator, with a mean amino acid length of roughly 500 AA.

Gene family prediction and reducing transcript redundancy using Cogent

Cogent (coding genome reconstruction tool) v1.3 (https://github.com/Magdoll/Cogent) uses k-mer similarity profiles in order to partition full length coding sequences into gene families, after which it reconstructs contigs containing the full coding region.

Illumina data alignment and transcriptome polishing using Pilon

Paired end Illumina data was trimmed using Atropos(Didion et al. 2017) (https://github.com/jdidion/atropos) and aligned to the Cogent gene families (CCD) dataset using Bowtie2(Langmead and Salzberg 2012) (http://bowtie-bio.sourceforge.net/bowtie2/index.shtml). Polishing to correct reads was performed from these alignments using Pilon(Walker et al. 2014) (https://github.com/broadinstitute/pilon/wiki). We performed ten rounds of polishing and alignment, and polished CCD reads were used in downstream analysis which required single nucleotide accuracy (dN/dS, details below). Multiple rounds are necessary as read alignment improves with each round of error correction - we have found more errors are corrected through multiple rounds. After the first round of pilon error correction which found 72,178 errors to be corrected, subsequent errors found rapidly dropped until only 132 errors were found on the tenth round (Supplementary Figure 4)

ORF prediction and protein translation using ANGEL

ANGEL consists of three primary stages: dumb ORF prediction, classifier training, and prediction. Dumb open reading frame (ORF) prediction, which produces all six possible open reading frames per given transcript, was run for all transcripts with a minimum length of 300 amino acids. Training involves the creation of a random subset of non-redundant transcripts, which is then used to create a classifier pickle file implemented in the prediction stage. Prediction then outputs the most likely ORF (minimum peptide length of 50 amino acids) based on length and coding potential of each given sequence.

Orthologous gene prediction using OrthoMCL

OrthoMCL works by performing an all-vs-all BlastP comparison of input sequences, and determining reciprocal best hit of all input pairings (cut off at e-5 and >50% match). Putative orthologs are the reciprocal best hits between species, while putative paralogs constitute reciprocal better hits within species. A normalized similarity matrix, followed by Markov clustering, produces ortholog groups. We compared our ruby-throated hummingbird *Archilochus colubris* to five other birds- *Calypte anna* (Anna's hummingbird, release date 2014-04-24, ftp://climb.genomics.cn/pub/10.5524/101001_102000/101004/Calypte_anna.pep.gz), *Gallus gallus* (chicken, Galgal5, ftp://ftp.ncbi.nih.gov/genomes/Gallus_gallus/protein/protein.fa.gz), *Chaetura pelagica* (chimney swift, release date 2014-04-24,ftp://ftp.ncbi.nih.gov/genomes/Chaetura_pelagica/protein/protein.fa.gz), *Taeniopygia guttata* (zebra finch, taeGut3.2.4, ftp://ftp.ensembl.org/pub/release-85/fasta/taeniopygia_guttata/pep/Taeniopygia_guttata.taeGut3.2.4.pep.all.fa.gz), and *Melopsittacus undulatus* (budgeriger, melUnd1, ftp://climb.genomics.cn/pub/10.5524/100001_101000/100059/BGIMUN1.120628.gene.withUTR.pep), as well as human (*Homo sapiens*, hg38, ftp://ftp.ensembl.org/pub/release-85/fasta/homo_sapiens/pep/Homo_sapiens.GRCh38.pep.all.fa.gz) and American alligator (*Alligator mississippiens,* allMis0.2/1, ftp://ftp.ncbi.nih.gov/genomes/Alligator_mississippiensis/protein/protein.fa.gz).

GO analysis using PANTHER

Orthologs shared between *Calypte anna* and *Archilochus colubris*, but not with other birds included in OrthoMCL analysis, were examined more closely using gene ontology (GO) analysis. BlastP with default alignment settings was used to determine the top three putative hits for each ortholog. Genbank accession numbers were converted in a universal gene symbol using BioDB (https://biodbnet-abcc.ncifcrf.gov/db/db2db.php, Mudunuri et al. 2009). Gene symbols were then run through Panther (http://pantherdb.org/, Mi et al. 2017), producing GO terms for the input orthologs.

Hepatic lipogenesis pathway analysis

Accessions of genes of interest were obtained from NCBI and are listed in supplemental table 5. BLASTP was performed against the database of HQD isoforms to obtain a representative sequence with highest homology to query, and counts of these enzymes were normalized to

produce relative transcript abundances. Representative protein sequences aligned with ClustalW using default settings.

Conservation analysis using PAML

PAML4.9c (Yang 2007) was used in order to estimate pairwise conservation between ruby-throated hummingbird and the five species (*Archilochus colubris, Calypte anna, Gallus Gallus, Chaetura pelagica*, *Alligator mississippiensis* and *Homo sapiens*) used as comparators in pathway analysis. Pairwise alignment was performed using CLUSTALW with PHYLIP output. Codeml module was used, runmode= -2, and default settings. dN/dS was estimated using Nei and Gojobori (Nei and Gojobori 1986). The mRNA sequences were then translated to protein using ExPasY, and these proteins were aligned using CLUSTAL, alignment scores were recorded.
