# Supplementary material for: Single-molecule, full-length transcript sequencing provides insight into the extreme metabolism of the ruby-throated hummingbird Archilochus colubris
Source: Gigascience. 2018 Feb 15;7(3):giy009. doi: 10.1093/gigascience/giy009 (PMC5869288; doi:10.1093/gigascience/giy009)
Supplement: Response_to_Reviewer_Comments_Original_Submission.pdf [file giy009_response_to_reviewer_comments_original_submission.pdf]

Author's Response To Reviewer Comments

Below please find our revisions to the manuscript entitled “Single molecule, full-length transcript sequencing provides insight into the extreme metabolism of ruby-throated hummingbird Archilochus colubris”.

We thank the editors and reviewers for their thoughtful comments on the initial submission. We have addressed all of the reviewers’ concerns and are submitting what we believe to be an improved version of our manuscript. The revised manuscript includes careful reanalysis as well as a new Illumina RNA-seq data set for quantification and correction of errors in the PacBio data.

Reviewer reports:

<<<<<

Reviewer #1: Workman et al. have presented a manuscript on a very relevant topic - third generation sequencing on an fascinating bird, the ruby-throated hummingbird. While I wholeheartedly agree that long read sequencing will address several assembly, and downstream, problems - resulting in a better understanding of several genetic aspects of any organism, there are several inaccuracies in the current manuscript that need to be addressed before publication.

1. There are several transcripts (about 155) from viruses - SRR5237173.540969 is one such example. It encodes a 823 long ORF, which has Identities = 702/824 (85%) with a FBS protein (NP 955606.1) from Fujinami sarcoma virus (FSV). The relation of FSV to avian genomes has been long known (http://www.pnas.org/content/77/4/2018). This should modify, however minimally, the statement 'resulted in 119,292 HQD and 1,061,147 ASD peptide sequences'. Similar techniques should be used for eliminating bacterial and fungal transcripts. Methods for quickly detecting metagenomic transcripts have been elucidated in http://biorxiv.org/content/early/2016/10/04/079186. Also, ORF-based annotation help in filtering bacterial transcripts from PacBio reads (http://biorxiv.org/content/early/2017/01/17/100974).

=====

Thank you for performing this check. We have performed BLAST searches to filter out these contaminating reads (details in supplemental methods). We have removed contaminating sequences from the HQD and LQD consensus isoforms set, then re-run ANGEL to produce the corresponding 93,469 HQD and 679,956 ASD peptide sequences. The resulting lower counts than previous runs were a result of both contaminant removal and changing minimal acceptable protein length settings (details in supplemental methods). Filtered data has been reuploaded to Zenodo [DOI:10.5281/zenodo.781592].

<<<<<

2. Figure 5A, and the associated analysis ('poor pairwise protein alignment between A. colubris and all examined species, such as with DGAT2, is suggestive of misannotation or splice

variation in our transcriptome, cases with variable alignment identities provide interesting targets for further investigation') is incorrect. The DGAT2 from C. anna (XP 008493408.1) is 358 aa long, and is 357 aa identical to the ORF encoded by SRR5237173.336808. The color coding in Fig5A suggests about 60% identity, based on other transcripts (SRR5237173.185657, SRR5237173.22637, etc - there are 200 homologous transcripts). So, there seems to be two genes for DGAT2, mapping to two different scaffolds in the C. anna genome - an interesting observation is that one gene has very low expression (a single transcript), while the other has several.

=====

Yes, it appears the initial alignment was based on a sequence that is “DGAT2-like” and not DGAT2, and that they do in fact map to different scaffolds in the genome. When substituting the SRR5237173.336808 sequence for MSA, we obtain much higher percent identities as expected. The heatmap plot has been updated correspondingly.

<<<<<

3. The PAML numbers, and their evolutionary connotations are not properly explained. Finding the number of genes (ACACA seems to have only one), and possibly quantifying them (roughly, ACACA has about 400 homologous transcripts: some complete - some fragmented) would provide interesting insights in the pathway.

=====

We have added language in the main text to further explain PAML analysis and the implications of it [“In order to...metric of positive selection”]. We have also added a heatmap of relative abundances of transcripts [Figure 5], which has indeed provided interesting insights that we touch on briefly in the discussion [“Relative abundances of enzymes of interest...of the animals”].

<<<<<

Reviewer #2: This manuscript describes the sequencing of full length cDNA (RNA-Seq) from a hummingbird using PacBio technology. In general I find this a well written manuscript describing an important avian genomic resource. It should, however, be noted that the format of this manuscript is not following the journal guide lines for a "Data Note". I have a few questions and suggestions for improvement as outlined below.

Throughout I'm very confused by the mixed use of "hepatopancreas" and "liver" to describe the tissue being sampled and sequenced. My understanding is that the term "hepatopancreas" is mainly used for invertebrates and fish. I thus suggest changing to "liver" throughout.

=====

We agree and have updated to “liver” throughout the manuscript.

<<<<<

The main methodological novelty with this work is the use of PacBio data only, for a transcriptome characterisation in a non-model organism. While I applaud this initiative, and especially the detailed description of the downstream bioinformatics pipeline, it also raises some questions regarding data quality. The standard way of using PacBio data for a species without a reference genome (or transcriptome) is to complement the long reads with a substantial amount of short read sequences (for example Illumina) in order to correct the high level of sequencing errors in the PacBio reads. It is unclear (and not well described in the current manuscript) how the lack of such error correcting affect the quality of the resulting transcriptome sequence. This is especially problematic for inference of variable sites (SNPs and InDels) and the molecular evolution type analyses presented at the end of the results section here. The dN/dS analyses in particular are especially sensitive to sequencing and alignment errors that may be abundant in this dataset. I suggest to investigate occurrence of sequencing errors more formally and to omit any molecular evolution analyses until the transcriptome sequence variation has been validated using complementary sequencing.

=====

Our pipeline includes several error correction methods to address the inherent high error rate associated with single molecule sequencing. Specifically, the circular consensus reads generated by Pacbio are the result of consensus generation/error correction, and the following Arrow polishing step of our data analysis pipeline is purported to produce reads accurate to 99.999% with 50-fold coverage. However, due to the sporadic coverage nature of RNAseq data, not all of our reads will be this accurate. While we are confident the accuracy of our data as sufficient for tasks such as homology identification and redundancy reduction, we agree that Illumina validation should be employed before making claims that involve single nucleotide resolution.

So, to determine the level of remaining error, and in response to the reviewer's criticism, we performed Illumina sequencing on liver mRNA, and used pilon to determine and correct errors which remain at the end of our pipeline. We found a relatively small number of errors were corrected, and repeated our dN/dS analysis with the corrected dataset and updated Figure 5 to reflect this. The dN/dS values did not change substantially, with the exception of the chicken value which was significantly reduced. The manuscript and methods sections have also been updated accordingly.

<<<<<

It is repeatedly stated that this is "the first high-coverage transcriptome of any single avian tissue". This is a pretty bold statement, given the large amount of transcriptome studies of several model bird species (such as chicken, zebra finch, flycatchers, crows and others). It is also completely un-necessary in this context. This study is interesting as it is, without any need to try to exaggerate the novelty with this kind of dubious statements.

=====

This has been changed, thank you for the suggestion.

<<<<<

With transcriptome sequencing (RNA-Seq) it is possible to get information on relative transcription rates for the identified expressed genes (through read depth quantification). I'm puzzled why there are no such inferences reported anywhere in this manuscript.

=====

We compared overrepresentation of genes in specific GO pathways using Panther; additionally, we have included abundance estimates for our hepatic lipogenesis pathway analysis to address this concern. Also, we have included information regarding the most abundant transcripts in our liver dataset in supplemental table 2D and reference briefly in the main text [Data is further summarized...Supplemental table 2].

<<<<<

The results section is full of rather detailed methods descriptions. I would have opted to keep these in the methods section only.

=====

In revising our MS to a data note format, we have pooled methods and related results into Data Description, and moved detailed methodology to a Supplemental Methods section.

<<<<<

In the fifth section of the results, it would have been useful to have some information of the divergence time between the Anna's Hummingbird and the focal species.

=====

We have added in this information along with the alignment section of data description.

<<<<<

Last section of the fourth results page: "a higher degree of divergence within this class of enzymes than would be predicted statistically". Please explain what statistical test was used here and report the test statistic, sample size and p-value.

=====

In order to test for overrepresentation of lipid metabolic process enzymes, we used the statistical overrepresentation test employed by Panther and described in Box 3 of their paper (http://www.nature.com/nprot/journal/v8/n8/box/nprot.2013.092_BX3.html). These results

(sample size, p value) are reported in Supplemental table 4, and a more detailed explanation of this was included.

<<<<<

First section of the Methods: How many bird samples were sequenced? In the first sentence it only says "ruby-throated hummingbirds" (plural without any specific numbers). Later it says that tissue was collected from one bird. Please be more specific here. Also please provide more specific information about the one bird individual sequenced (age, sex, time and place of sampling etc.). A lot of effort have been made on this one individual - it is important to include as much meta data as possible for this.

=====

We sequenced a single sample from one bird. Standard hummingbird aging techniques only allow us to resolve the age of wild-captured hummingbirds as either a “hatch year” individual (i.e. one born within the last 12 months) or an “after hatch year” individual (at least one year old, though maybe more). Given the bird was sacrificed approximately one year after capture, and was found to be “after hatch year” when captured, we can only confidently conclude that it was 2+ years old at the time of sacrifice. We have amended the text to explicitly state this age range.

<<<<<

Data Accession: It would be very useful to also have analyses scripts and pipelines placed in a public repository for future reference.

=====

It has been added to our availability and requirements section.

<<<<<

Legends to figure 1 and 2 are in the wrong order.

Please check format of reference list.

=====

Reference list has been updated to Nature format and links have been incorporated into citations.

<<<<<

Figure 1. Details here need to be much clearer explained in the figure caption. For example please provide detail about abbreviations used, and axis lables. For B I think "5000Mb of sequencing data was larger than 2000bp" should read "4000Mb of sequencing data was larger than 2000bp". Or am I reading the figure wrong?

Figure 2. Very clear and useful description of the work flow and analysis pipeline. Maybe you could add details about the amount of data in- and outputted at each stage of the analyses?
Figure 3. Again the caption is lacking in clarity and detail. The reader should not need to be familiar with the specific software and output terminology in order to understand what is done. The figure with caption should also be understandable without having to read the main text.
Figure 4. Not sure how important this information is (maybe better placed in a supplement). Also it is unclear what kind of statistical analyses that is being presented in 4B. Please elaborate on what was done here. What does the stars represent?
Figure 5. Again caption is unclear. What does the right heat map in 5B represent?

=====

Figure headings, captions and legends have been updated for clarity. Data into and out of each stage of analysis added to Figure 2.
