# Supplementary material for: Single-molecule, full-length transcript sequencing provides insight into the extreme metabolism of the ruby-throated hummingbird Archilochus colubris
Source: Gigascience. 2018 Feb 15;7(3):giy009. doi: 10.1093/gigascience/giy009 (PMC5869288; doi:10.1093/gigascience/giy009)
Supplement: Response_to_Reviewer_Comments_Revision_1.pdf [file giy009_response_to_reviewer_comments_revision_1.pdf]

Author's Response To Reviewer Comments

Below please find our revisions and response to the reviewers concerns on the manuscript entitled “Single molecule, full-length transcript sequencing provides insight into the extreme metabolism of ruby-throated hummingbird Archilochus colubris”
We thank the editors and reviewers for their thoughtful comments on the revision. We have addressed all of the reviewers’ concerns and are submitting a detailed response along with an improved version of the manuscript.
>>>
Reviewer #1:
I am satisfied with the changes made in response to suggestions (from me and another reviewer) previously, and pleased with the way the manuscript has shaped with the additional Illumina sequences.
However, the paper still has inadequacies that need to be fixed. While, genes that should not have been included have been fixed, others that are bona-fide genes have been left out.
Several genes that map to the Calypte anna and Aquila chrysaetos transcripts are missing in the acolubris HQD or cogent CDS. An example is SRR5237173.100014 (953 nt). This is 99% similar to 'Calypte anna zinc finger CCCH-type containing 6 (ZC3H6), transcript variant X1, mRNA' (XM 008499961.1). Another example is SRR5237173.117510 (904 nt), which matches 88% identity to the golden eagle (XM 011575986.1) "vesicle transport through interaction with t-SNAREs 1A (VTI1A) transcript variant X2, mRNA'. Its acceptable to miss a few genes, but my analysis shows 6000 genes (corresponding to about 22,000 PacBio transcripts) missing by just using Calypte anna and Aquila chrysaetos (not including other avian species). Given the quality and depth of the Pacbio transcriptome, the coverage of known genes should be better.
===
Thank you for your analysis. First, it should be noted that, although this is a high quality and (relatively) high depth dataset, it originated from a single tissue of a single animal, which naturally limits genes detected. As we stated in the paper, “our A. colubris transcriptome only captured around half of this diversity, likely due to our sample being single tissue, collection time point and individual.” Furthermore, our filtering steps are somewhat conservative; there is a filter in place in the Quiver/Arrow step of the pipeline, which requires that a putative isoform have at least two independent, full-length sequences, with predicted accuracy scores of >99%. These very stringent parameters were put in place to preclude the inclusion of potentially spurious transcripts into further analysis. This means that low abundance genes in this tissue and sample may be lost; though Supplemental Figure 1 suggests that we have largely saturated sequencing depth on this particular sample.
<<<
>>>
Another aspect that has not been touched upon is the quantification of the Illumina transcriptome, which provides an unbiased view of highly expressed genes, as compared to known genes in the hepatic lipogenic pathway. The availability of the sequences from two different technologies provides a platform for comparing
and contrasting them.
===
See “Looking at Pacbio reads…” response below for rationale against comparative quantification.

<<<

>>>

Using Salmon (https://www.nature.com/nmeth/journal/v14/n4/abs/nmeth.4197.html) taking the acolubris HQD.cds as target, I have quantified the Illumina transcriptome (SRR6148275). The top ten genes are provided below - and the corresponding annotation based on the the BLAST 'nt' database.

I1.C1447.F29P0.1522.M.5511 XM 008499421.1 Calypte anna acidic mammalian chitinase-like (LOC103533552)

I1.C3140.F2P0.1756.M.20042 XM 010008736.1 Chaetura pelagica apolipoprotein A-I (LOC104398601)

I0.C76367.F48P0.832.M.3544 XM 008491568.1 Calypte anna lipocalin-like 1 (LCNL1)

I0.C68578.F2P0.585.M.3451 XM 008498154.1 Calypte anna avidin-like (LOC103532403)

I2.C288336.F2P0.1193.M.25884 KP875235.1 Pygoscelis antarcticus 18S ribosomal RNA gene

I0.C104457.F3P0.427.M.4404 XM 014961892.1 Calidris pugnax apolipoprotein A-II-like (LOC106899953)

I0.C102059.F3P0.428.M.4043 XM 014961892.1 Calidris pugnax apolipoprotein A-II-like (LOC106899953)

I1.C554372.F3P0.1606.M.14434 HM033221.1 Archilochus colubris voucher BIOUG:BIBS RTHU cytochrome

I0.C16075.F5P0.700.M.4006 XM 008504716.1 Calypte anna serum amyloid A protein-like (LOC103538359)

I3.C3772.F3P0.2060.M.41994 XM 008499863.1 Calypte anna apolipoprotein A-I (LOC103533949)

The mammalian chitinase-like is an interesting gene, which seems to have little or no reference in the current literature for hummingbirds. This might be a serum chitinase (https://www.ncbi.nlm.nih.gov/pubmed/11591385)associated with the I0 C16075.F5P0.700.M.4006 transcript (a serum amyloid A protein), another highly transcribed gene. Another possibility is it is a gut-chitinase(https://www.ncbi.nlm.nih.gov/pubmed/12133911),although I am not sure whether a liver chitinase can make its way to the gut.

===

From the Suzuki [PMID: 11591385] paper, "Avian species have dual sources for chitinase production in the stomach and liver, and mammals may have selected the major source, either the alimentary canal or liver, through evolution. The chitinase produced in hepatocytes circulates in the bloodstream and specifically defends against chitin containing microorganisms via its chitin-binding and chitin-fragmenting abilities." While humans only express chitinase in the gut (and also through certain white blood cells), chickens express the enzyme in both the gut and liver, and other mammals(cows) express the enzyme only in the liver. Suzuki et al. hypothesize that the ancestral state is expression of chitinase in both tissues. While the gut chitinase is used for digestion, expression in liver is believed to contribute to serum chitinase levels and to act as a defense against chitin-containing pathogens. The chitinase-like isoform in our dataset is highly homologous to the chicken liver chitinase. As we have not found any literature describing chitinase expression in the pancreas, nor of liver chitinase being transported to the gut for digestion, it seems likely that the hummingbird liver expresses this protein for circulation and

defense against pathogens.
>>>
<<<
The high expression levels of the chitinase gene is not apparent from the Pacbio sequences (SRR5237173), probably since they are not raw reads(?). Do the Pacbio raw reads corroborate with the Illumina results?
===
Looking at the Pacbio raw reads, it appears that this chitinase-like gene is in low abundance, around 100 putative hits as opposed to 80,000 for the most abundant transcripts (acyl-CoA desaturase, steroyl CoA desaturase). This large discrepancy in expression relative to Illumina is likely due to two things; 1) differences in library preparation methodologies, and 2; inter-individual differences. cDNA production methodology is completely different (full length reverse transcription vs random priming, different amounts of PCR cycles) which could be skewing abundance drastically. Additionally, different individuals were used for the two types of sequencing; though this is reasonable for sequence correction using Illumina, it's likely not ideal for quantitation validation. In considering future experiments, we will be sure to perform such an assay to measure the relative quantitation between Illumina and Pacbio (long-read) quantitation; there seems to be too many confounding factors to confidently report this analysis. We are also planning incorporation of spike-in standards (Lexogen SIRVs) for future studies to have an additional control.
>>>
<<<
Minor comment: It would be also useful to annotate the known cds based on (high) homology with other known avian genes (I had to BLAST to 'nt' in order to obtain the annotation.
===
This has been done and uploaded to Zenodo (10.5281/zenodo.1054086), thank you for the suggestion.
>>>
