# Supplementary material for: Single-molecule, full-length transcript sequencing provides insight into the extreme metabolism of the ruby-throated hummingbird Archilochus colubris
Source: Gigascience. 2018 Feb 15;7(3):giy009. doi: 10.1093/gigascience/giy009 (PMC5869288; doi:10.1093/gigascience/giy009)
Supplement: Response_to_Reviewer_Comments_Revision_2.pdf [file giy009_response_to_reviewer_comments_revision_2.pdf]

Author's Response To Reviewer Comments

As per editorial instructions, we have:

1) Included citations to the GigaDB and ZENODO datasets wehre appropriate
2) Removed the sentence "All other data available upon request"
3) Added the MIT license to our github repo
4) Added a statement to the manuscript involving the approval for research with animals (Candian Wildlife Service permit and UToronto AUP).

2/5/18
5) Fixed the references 28, 29 and 38
