# Supplementary material for: Single-molecule, full-length transcript sequencing provides insight into the extreme metabolism of the ruby-throated hummingbird Archilochus colubris
Source: Gigascience. 2018 Feb 15;7(3):giy009. doi: 10.1093/gigascience/giy009 (PMC5869288; doi:10.1093/gigascience/giy009)
Supplement: Reviewer_1_Report_(Original_Submission) -- Sandeep Chakraborty — 02 May 2017 Reviewed [file giy009_reviewer_1_report_(original_submission).pdf]

Reviewer Report

Title: Single molecule, full-length transcript sequencing provides insight into the extreme metabolism of ruby-throated hummingbird Archilochus colubris

Version: Original Submission **Date:** 02 May 2017

Reviewer name: Sandeep Chakraborty

Reviewer Comments to Author:

Workman et al. have presented a manuscript on a very relevant topic - third generation sequencing on an
fascinating bird, the ruby-throated hummingbird. While I wholeheartedly agree that long read sequencing
will address several assembly, and downstream, problems - resulting in a better understanding of several
genetic aspects of any organism, there are several inaccuracies in the current manuscript that need to be
addressed before publication.

1. There are several transcripts (about 155) from viruses - SRR5237173.540969 is one such example. It encodes a 823 long ORF, which has Identities = 702/824 (85%) with a FBS protein (NP 955606.1) from Fujinami sarcoma virus (FSV). The relation of FSV to avian genomes has been long known (http://www.pnas.org/content/77/4/2018). This should modify, however minimally, the statement 'resulted in 119,292 HQD and 1,061,147 ASD peptide sequences'. Similar techniques should be used for eliminating bacterial and fungal transcripts. Methods for quickly detecting metagenomic transcripts have been elucidated in http://biorxiv.org/content/early/2016/10/04/079186. Also, ORF-based annotation help in filtering bacterial transcripts from PacBio reads (http://biorxiv.org/content/early/2017/01/17/100974).

2. Figure 5A, and the associated analysis ('poor pairwise protein alignment between A. colubris and all examined species, such as with DGAT2, is suggestive of misannotation or splice variation in our transcriptome, cases with variable alignment identities provide interesting targets for further investigation') is incorrect. The DGAT2 from C. anna (XP 008493408.1) is 358 aa long, and is 357 aa identical to the ORF encoded by SRR5237173.336808. The color coding in Fig5A suggests about 60% identity, based on other transcripts (SRR5237173.185657, SRR5237173.22637, etc - there are 200 homologous transcripts). So, there seems to be two genes for DGAT2, mapping to two different scaffolds in the C. anna genome - an interesting observation is that one gene has very low expression (a single transcript), while the other has several.

3. The PAML numbers, and their evolutionary connotations are not properly explained. Finding the number of genes (ACACA seems to have only one), and possibly quantifying them (roughly, ACACA

has about 400 homologous transcripts: some complete - some fragmented) would provide interesting insights in the pathway.

Based on the above mentioned issues, my verdict will be "major revision".

Level of Interest

Please indicate how interesting you found the manuscript: An article of importance in its field

Quality of Written English

Please indicate the quality of language in the manuscript: Acceptable

Declaration of Competing Interests

Please complete a declaration of competing interests, considering the following questions:

- Have you in the past five years received reimbursements, fees, funding, or salary from an organisation that may in any way gain or lose financially from the publication of this manuscript, either now or in the future?
- Do you hold any stocks or shares in an organisation that may in any way gain or lose financially from the publication of this manuscript, either now or in the future?
- Do you hold or are you currently applying for any patents relating to the content of the manuscript?
- Have you received reimbursements, fees, funding, or salary from an organization that holds or has applied for patents relating to the content of the manuscript?
- Do you have any other financial competing interests?
- Do you have any non-financial competing interests in relation to this paper?

If you can answer no to all of the above, write 'I declare that I have no competing interests' below. If your reply is yes to any, please give details below.

I declare that I have no competing interests.

I agree to the open peer review policy of the journal. I understand that my name will be included on my report to the authors and, if the manuscript is accepted for publication, my named report including any attachments I upload will be posted on the website along with the authors' responses. I agree for my report to be made available under an Open Access Creative Commons CC-BY license

(http://creativecommons.org/licenses/by/4.0/). I understand that any comments which I do not wish to be included in my named report can be included as confidential comments to the editors, which will not be published.

I agree to the open peer review policy of the journal

To further support our reviewers, we have joined with Publons, where you can gain additional credit to further highlight your hard work (see: https://publons.com/journal/530/gigascience). On publication of this paper, your review will be automatically added to Publons, you can then choose whether or not to claim your Publons credit. I understand this statement.

Yes
