# Supplementary material for: Single-molecule, full-length transcript sequencing provides insight into the extreme metabolism of the ruby-throated hummingbird Archilochus colubris
Source: Gigascience. 2018 Feb 15;7(3):giy009. doi: 10.1093/gigascience/giy009 (PMC5869288; doi:10.1093/gigascience/giy009)
Supplement: Reviewer_1_Report_(Revision_1) -- Sandeep Chakraborty — 03 Nov 2017 Reviewed [file giy009_reviewer_1_report_(revision_1).pdf]

Reviewer Report

Title: Single molecule, full-length transcript sequencing provides insight into the extreme metabolism of ruby-throated hummingbird Archilochus colubris

Version: Revision 1 **Date:** 03 Nov 2017

Reviewer name: Sandeep Chakraborty

Reviewer Comments to Author:

I am satisfied with the changes made in response to suggestions (from me and another reviewer) previously,
and pleased with the way the Ms has shaped with the additional Illumina sequences.

However, the paper still has inadequacies that need to be fixed. While, genes that should not have been included have been fixed, others that are bona-fide genes have been left out.

Several genes that map to the Calypte anna and Aquila chrysaetos transcripts are missing in the acolu-bris HQD or cogent CDS. An example is SRR5237173.100014 (953 nt). This is 99% similar to 'Calypte anna zinc finger CCCH-type containing 6 (ZC3H6), transcript variant X1, mRNA' (XM 008499961.1). Another example is SRR5237173.117510 (904 nt), which matches 88% identity to the golden eagle (XM 011575986.1)
"vesicle transport through interaction with t-SNAREs 1A (VTI1A) transcript variant X2, mRNA'.

Its acceptable to miss a few genes, but my analysis shows 6000 genes (corresponding to about 22,000 PacBio transcripts) missing by just using Calypte anna and Aquila chrysaetos (not including other avian species). Given the quality and depth of the Pacbio transcriptome, the coverage of known genes should be
better.

Another aspect that has not been touched upon is the quantification of the Illumina transcriptome, which
provides an unbiased view of highly expressed genes, as compared to known genes in the hepatic lipogenic
pathway. The availability of the sequences from two different technologies provides a platform for comparing
and contrasting them.

Using Salmon (https://www.nature.com/nmeth/journal/v14/n4/abs/nmeth.4197.html) taking the acol-ubris HQD.cds as target, I have quantified the Illumina transcriptome (SRR6148275). The top ten genes

are provided below - and the corresponding annotation based on the the BLAST 'nt' database.

I1.C1447.F29P0.1522.M.5511 XM 008499421.1 Calypte anna acidic mammalian chitinase-like (LOC103533552)
I1.C3140.F2P0.1756.M.20042 XM 010008736.1 Chaetura pelagica apolipoprotein A-I (LOC104398601)
I0.C76367.F48P0.832.M.3544 XM 008491568.1 Calypte anna lipocalin-like 1 (LCNL1)
I0.C68578.F2P0.585.M.3451 XM 008498154.1 Calypte anna avidin-like (LOC103532403)
I2.C288336.F2P0.1193.M.25884 KP875235.1 Pygoscelis antarcticus 18S ribosomal RNA gene
I0.C104457.F3P0.427.M.4404 XM 014961892.1 Calidris pugnax apolipoprotein A-II-like (LOC106899953)
I0.C102059.F3P0.428.M.4043 XM 014961892.1 Calidris pugnax apolipoprotein A-II-like (LOC106899953)
I1.C554372.F3P0.1606.M.14434 HM033221.1 Archilochus colubris voucher BIOUG:BIBS RTHU cytochrome
I0.C16075.F5P0.700.M.4006 XM 008504716.1 Calypte anna serum amyloid A protein-like (LOC103538359)
I3.C3772.F3P0.2060.M.41994 XM 008499863.1 Calypte anna apolipoprotein A-I (LOC103533949)

The mammalian chitinase-like is an interesting gene, which seems to have little or no reference in the cur-
rent literature for hummingbirds. This might be a serum chitinase (https://www.ncbi.nlm.nih.gov/pubmed/11591385)
associated with the I0 C16075.F5P0.700.M.4006 transcript (a serum amyloid A protein), another highly tran-
scribed gene. Another possibility is it is a gut chitinase (https://www.ncbi.nlm.nih.gov/pubmed/12133911),
although I am not sure whether a liver chitinase can make its way to the gut.

The high expression levels of the chitinase gene is not apparent from the Pacbio sequences (SRR5237173),
probably since they are not raw reads(?). Do the Pacbio raw reads corroborate with the Illumina results?

Minor comment: It would be also useful to annotate the known cds based on (high) homology with other
known avian genes (I had to BLAST to 'nt' in order to obtain the annotation.

Level of Interest

Please indicate how interesting you found the manuscript: An article of importance in its field

Quality of Written English

Please indicate the quality of language in the manuscript: Acceptable

Declaration of Competing Interests

Please complete a declaration of competing interests, considering the following questions:

- Have you in the past five years received reimbursements, fees, funding, or salary from an organisation that may in any way gain or lose financially from the publication of this manuscript, either now or in the future?
- Do you hold any stocks or shares in an organisation that may in any way gain or lose financially from the publication of this manuscript, either now or in the future?
- Do you hold or are you currently applying for any patents relating to the content of the manuscript?
- Have you received reimbursements, fees, funding, or salary from an organization that holds or has applied for patents relating to the content of the manuscript?
- Do you have any other financial competing interests?
- Do you have any non-financial competing interests in relation to this paper?

If you can answer no to all of the above, write 'I declare that I have no competing interests' below. If your reply is yes to any, please give details below.

I declare that I have no competing interests

I agree to the open peer review policy of the journal. I understand that my name will be included on my report to the authors and, if the manuscript is accepted for publication, my named report including any attachments I upload will be posted on the website along with the authors' responses. I agree for my report to be made available under an Open Access Creative Commons CC-BY license (http://creativecommons.org/licenses/by/4.0/). I understand that any comments which I do not wish to be included in my named report can be included as confidential comments to the editors, which will not be published.

I agree to the open peer review policy of the journal

To further support our reviewers, we have joined with Publons, where you can gain additional credit to further highlight your hard work (see: https://publons.com/journal/530/gigascience). On publication of this paper, your review will be automatically added to Publons, you can then choose whether or not to claim your Publons credit. I understand this statement.

Yes
