# Supplementary material for: Single-molecule, full-length transcript sequencing provides insight into the extreme metabolism of the ruby-throated hummingbird Archilochus colubris
Source: Gigascience. 2018 Feb 15;7(3):giy009. doi: 10.1093/gigascience/giy009 (PMC5869288; doi:10.1093/gigascience/giy009)
Supplement: Reviewer_2_Report_(Original_Submission) -- Robert Ekblom — 23 May 2017 Reviewed [file giy009_reviewer_2_report_(original_submission).pdf]

Reviewer Report

Title: Single molecule, full-length transcript sequencing provides insight into the extreme metabolism of ruby-throated hummingbird Archilochus colubris

Version: Original Submission **Date:** 23 May 2017

Reviewer name: Robert Ekblom

Reviewer Comments to Author:

This manuscript describes the sequencing of full length cDNA (RNA-Seq) from a hummingbird using PacBio technology. In general I find this a well written manuscript describing an important avian genomic resource. It should, however, be noted that the format of this manuscript is not following the journal guide lines for a "Data Note". I have a few questions and suggestions for improvement as outlined below.

Throughout I'm very confused by the mixed use of "hepatopancreas" and "liver" to describe the tissue being sampled and sequenced. My understanding is that the term "hepatopancreas" is mainly used for invertebrates and fish. I thus suggest changing to "liver" throughout.

The main methodological novelty with this work is the use of PacBio data only, for a transcriptome characterisation in a non-model organism. While I applaud this initiative, and especially the detailed description of the downstream bioinformatics pipeline, it also raises some questions regarding data quality. The standard way of using PacBio data for a species without a reference genome (or transcriptome) is to complement the long reads with a substantial amount of short read sequences (for example Illumina) in order to correct the high level of sequencing errors in the PacBio reads. It is unclear (and not well described in the current manuscript) how the lack of such error correcting affect the quality of the resulting transcriptome sequence. This is especially problematic for inference of variable sites (SNPs and InDels) and the molecular evolution type analyses presented at the end of the results section here. The dN/dS analyses in particular are especially sensitive to sequencing and alignment errors that may be abundant in this dataset. I suggest to investigate occurrence of sequencing errors more formally and to omit any molecular evolution analyses until the transcriptome sequence variation has been validated using complementary sequencing.

It is repeatedly stated that this is "the first high-coverage transcriptome of any single avian tissue". This is a pretty bold statement, given the large amount of transcriptome studies of several model bird species (such as chicken, zebra finch, flycatchers, crows and others). It is also completely un-necessary in this context. This study is interesting as it is, without any need to try to exaggerate the novelty with this kind of dubious statements.

With transcriptome sequencing (RNA-Seq) it is possible to get information on relative transcription rates for the identified expressed genes (through read depth quantification). I'm puzzled why there are no

such inferences reported anywhere in this manuscript.

The results section is full of rather detailed methods descriptions. I would have opted to keep these in the methods section only.

In the fifth section of the results, it would have been useful to have some information of the divergence time between the Anna's Humming bird and the focal species.

Last section of the fourth results page: "a higher degree of divergence within this class of enzymes than would be predicted statistically". Please explain what statistical test was used here and report the test statistic, sample size and p-value.

First section of the Methods: How many bird samples were sequenced? In the first sentence it only says "ruby-throated hummingbirds" (plural without any specific numbers). Later it says that tissue was collected from one bird. Please be more specific here. Also please provide more specific information about the one bird individual sequenced (age, sex, time and place of sampling etc.). A lot of effort have been made on this one individual - it is important to include as much meta data as possible for this.

Data Accession: It would be very useful to also have analyses scripts and pipelines placed in a public repository for future reference.

Legends to figure 1 and 2 are in the wrong order.

Please check format of reference list.

Figure 1. Details here need to be much clearer explained in the figure caption. For example please provide detail about abbreviations used, and axis lables. For B I think "5000Mb of sequencing data was larger than 2000bp" should read "4000Mb of sequencing data was larger than 2000bp". Or am I reading the figure wrong?

Figure 2. Very clear and useful description of the work flow and analysis pipeline. Maybe you could add details about the amount of data in- and outputted at each stage of the analyses?

Figure 3. Again the caption is lacking in clarity and detail. The reader should not need to be familiar with the specific software and output terminology in order to understand what is done. The figure with caption should also be understandable without having to read the main text.

Figure 4. Not sure how important this information is (maybe better placed in a supplement). Also it is unclear what kind of statistical analyses that is being presented in 4B. Please elaborate on what was done here. What does the stars represent?

Figure 5. Again caption is unclear. What does the right heat map in 5B represent?

Level of Interest

Please indicate how interesting you found the manuscript: An article of importance in its field

Quality of Written English

Please indicate the quality of language in the manuscript: Acceptable

Declaration of Competing Interests

Please complete a declaration of competing interests, considering the following questions:

- Have you in the past five years received reimbursements, fees, funding, or salary from an organisation that may in any way gain or lose financially from the publication of this manuscript, either now or in the future?
- Do you hold any stocks or shares in an organisation that may in any way gain or lose financially from the publication of this manuscript, either now or in the future?
- Do you hold or are you currently applying for any patents relating to the content of the manuscript?
- Have you received reimbursements, fees, funding, or salary from an organization that holds or has applied for patents relating to the content of the manuscript?
- Do you have any other financial competing interests?
- Do you have any non-financial competing interests in relation to this paper?

If you can answer no to all of the above, write 'I declare that I have no competing interests' below. If your reply is yes to any, please give details below.

I declare that I have no competing interests

I agree to the open peer review policy of the journal. I understand that my name will be included on my report to the authors and, if the manuscript is accepted for publication, my named report including any attachments I upload will be posted on the website along with the authors' responses. I agree for my report to be made available under an Open Access Creative Commons CC-BY license (http://creativecommons.org/licenses/by/4.0/). I understand that any comments which I do not wish to be included in my named report can be included as confidential comments to the editors, which will not be published.

I agree to the open peer review policy of the journal

To further support our reviewers, we have joined with Publons, where you can gain additional credit to further highlight your hard work (see: https://publons.com/journal/530/gigascience). On publication of this paper, your review will be automatically added to Publons, you can then choose whether or not to claim your Publons credit. I understand this statement.

Yes
